# Activation of alcohols as sulfonium salts in the photocatalytic hetero-difunctionalization of alkenes

**DOI:** 10.1038/s41557-025-02003-7

**Published:** 2025-11-26

**Authors:** Huaibo Zhao, Dario Filippini, Yiding Chen, Albert Gallego-Gamo, Louise S. Natrajan, Loïc R. E. Pantaine, Ciro Romano, David J. Procter

**Affiliations:** 1 Department of Chemistry, University of Manchester, Manchester, UK; 2 Process and Analytical Chemistry, Pharmaron UK Ltd., Hoddesdon, UK

**Keywords:** Photocatalysis, Synthetic chemistry methodology, Synthetic chemistry methodology

## Abstract

Motifs related to 1,2-diols and 1,2-amino alcohols are found widely in bioactive natural products, drugs and agrochemicals. These highly sought-after substructures would ideally be constructed by the direct addition of alcohols to the C=C bond of alkenes, both common substrate classes in chemical synthesis. However, their direct union is only possible if one of the pair can be rendered electron-deficient through derivatization; such approaches typically require stoichiometric amounts of strong oxidants and often lack generality. Here we describe a straightforward process in which both simple and complex alcohols can be converted under photocatalytic conditions to the corresponding alkoxy radicals—via the formation of alkoxy sulfonium salts—that react with alkenes en route to 1,2-diol and 1,2-amino-alcohol derivatives. The method can be easily adapted from laboratory to industrial, kilogram scale using a photoflow system. Spectroscopic analysis and control experiments have been used to probe the underpinning mechanism.

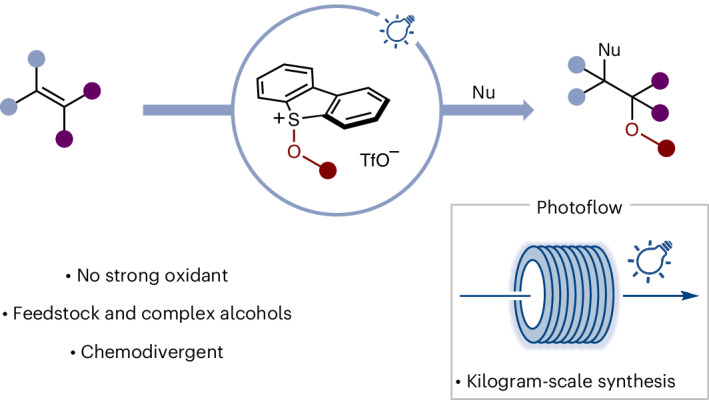

## Abstract (web summary)

1,2-Diols and 1,2-amino alcohols are widely found in bioactive compounds. Now it has been shown that alcohols can be converted, via alkoxy sulfonium salts, to alkoxy radicals that add to alkenes to give 1,2-diol and 1,2-amino-alcohol derivatives. The photocatalytic method can be run on a kilogram scale using a photoflow system.

## Main

**Fig. 1:**
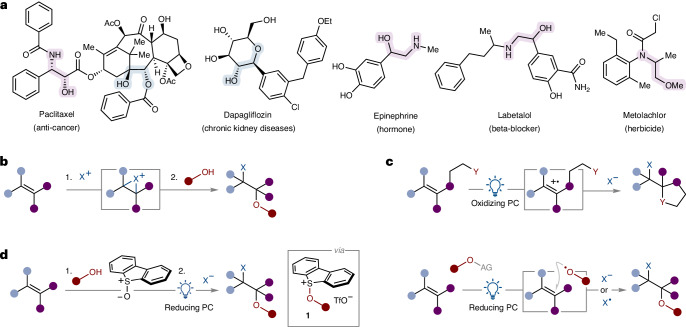
The importance of 1,2-diol and 1,2-amino-alcohol motifs and approaches for their construction. **a**, Selected natural products, pharmaceuticals and agrochemicals containing 1,2-diols or 1,2-amino-alcohol motifs. **b**, General approaches to achieve the hetero-difunctionalization of alkenes use polar chemistry, involve strong oxidants and show low functional group tolerance. **c**, General approaches to achieve the hetero-difunctionalization of alkenes using radical chemistry can have limited scope. Alkoxy radical approaches involve precursors that are not formed directly from alcohols. **d**, Our approach towards the photocatalytic hetero-difunctionalization of alkenes uses readily accessible, yet little-explored, alkoxysulfonium salts as bench-stable alkoxy radical precursors formed from both simple and complex alcohols. Ac, acetyl; AG, activating group; Et, ethyl; Me, methyl; PC, photocatalyst; Tf, trifluoromethanesulfonyl.

The widespread presence of 1,2-diol and 1,2-amino-alcohol motifs in molecules of societal importance—including bioactive natural products (for example, paclitaxel and adrenaline), drugs (for example, labetalol and dapagliflozin) and agrochemicals (for example, metolachlor)^1–4^ (Fig. 1a)—continues to spur efforts to develop new methods for their construction. The highly sought-after installation of two heteroatom-containing groups across an alkene C=C bond would ideally be triggered by the direct reaction of alkenes with alcohols (or other heteroatom containing nucleophiles)—both common classes of feedstock. However, owing to the electron-rich nature of both alcohols and alkenes, their direct union is only possible if one of the reactants can be rendered electron-deficient through derivatization—for example, using epoxides/halonium ions formed from the alkene partners^5–10^. These approaches typically require the use of stoichiometric amounts of strong oxidants and, thus, often lack generality, owing to functional group incompatibility, and lead to longer synthetic routes that involve protecting group manipulation (Fig. 1b). Alternative approaches involve the use of transition metal reagents and catalysts to activate alkenes towards nucleophile addition or to activate partners towards addition across alkene C=C bonds followed by trapping of organometallic intermediates with electrophilic alcohol derivatives^11–17^.

With the advent of photocatalysis, new methods for the hetero-difunctionalization of alkenes have emerged^18–30^ (Fig. 1c). Of note, the oxidation of alkenes to the corresponding radical cations under photocatalytic conditions prompts the addition of heteroatom nucleophiles. This approach, however, is mostly limited to particularly electron-rich alkenes, and internal nucleophiles are typically required^31–34^. An alternative radical approach exploiting polarity reversal involves the generation of electrophilic alkoxy radicals and their engagement with the electron-rich highest occupied molecular orbital of alkene partners^35,36^. Unfortunately, this approach is limited by the forcing conditions required for the generation of alkoxy radicals and their high reactivity, which result in side-reactions (for example, intra- and intermolecular hydrogen atom transfer (HAT) and fragmentation). Photocatalysis provides more user-friendly approaches to alkoxy radicals from a range of stable precursors^37–49^. Elegant photocatalytic methods for the direct generation of alkoxy radicals from alcohols, under mild and generally applicable conditions, exploit proton-coupled electron transfer^49^ and ligand-to-metal charge transfer^50^; alkene functionalization using alkoxy radicals generated using these approaches currently leads to monofunctionalized products of hydroetherification rather than difunctionalized products. For alkene difunctionalization, alkoxy radicals are not typically prepared from alcohols and instead arise from alkyl (pseudo)halides by nucleophilic substitution in multi-step syntheses, thus they are subject to a limited palette of structural variability that reflects the requirements of S_N_2 or S_N_1 reactions. A more convenient approach involves the direct utilization of alcohol feedstocks that are converted in situ to the corresponding alkoxy radicals by photocatalysed proton-coupled electron transfer before alkene addition^36^. This approach requires the combination of a base with an oxidizing photocatalyst. As the reduced photocatalyst must be oxidized in a later step to close the photocatalytic cycle, after the addition of the alkoxy radical to the alkene, hetero-difunctionalization of alkenes by two nucleophile partners using this approach has not been described.

Our interest in the activation of substrates using sulfoxides in Pummerer-type processes, and the application of the resulting sulfonium salts in photochemistry^51–57^, led us to consider alkoxy sulfonium salts (for example, **1**)^58–66^ as readily accessible, stable precursors of alkoxy radicals, which can be formed directly from a range of both simple and complex alcohols and that can be activated under mild reducing photocatalytic conditions (Fig. 1d). Upon addition of the resulting alkoxy radicals to an alkene, the resulting carbon radicals can close the photocatalytic cycle by reducing the oxidized form of the photocatalyst, setting the stage for the addition of a second nucleophile. By judicious choice of the nucleophile, for example, water, alcohol or nitrile, products of alkene 1,2-alkoxy-hydroxylation, 1,2-dialkoxylation and 1,2-alkoxy-amidation can be accessed, respectively. Of note, the strategy enables the direct use of alcohols as alkoxy radical precursors, after straightforward activation as alkoxy sulfonium salts, which can either be isolated and characterized or used in convenient telescoped processes for alkene difunctionalization. Given the low redox potential of alkoxy sulfonium salts (*E*_p_ of −0.14 eV versus Ag/AgCl), the use of strong reductants (or oxidants) is avoided, and alcohol and alkene partners bearing a range of functional groups can be engaged. Importantly, both the synthesis of alkoxy sulfonium salts and the photocatalytic alkene hetero-difunctionalization are amenable to being scaled-up; the process has been carried out on a kilogram scale in an industrial setting using a photoflow approach.

## Results and discussion

### Synthesis of alkoxy sulfonium salts

**Fig. 2:**
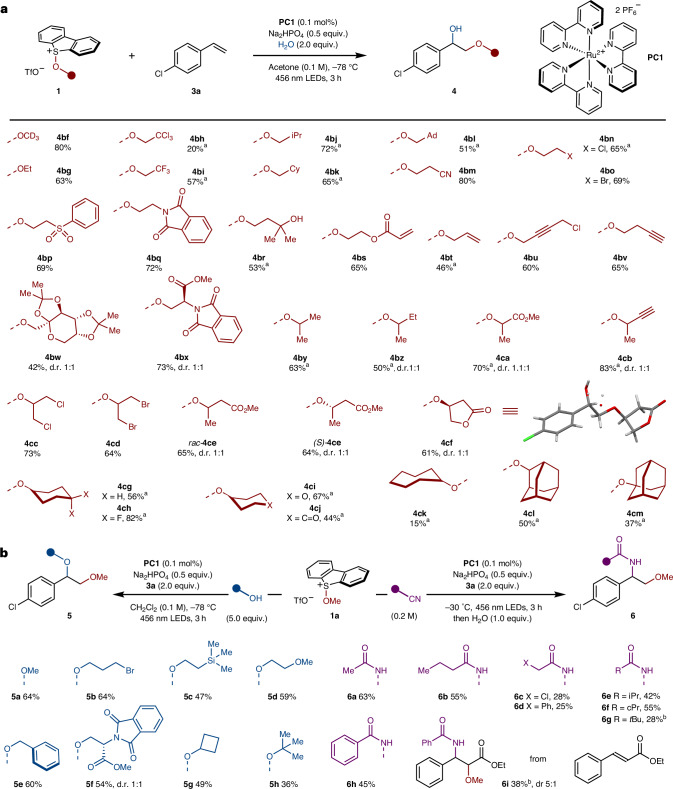
Alkoxy sulfonium salts in the photocatalytic difunctionalization of alkenes. **a**, The alkoxy sulfonium salt scope in the photocatalysed 1,2-alkoxy-hydroxylation of alkenes. **b**, The substrate scope for the photocatalytic 1,2-dialkoxylation (left) and the 1,2-alkoxy-amidation (right) of alkenes. ^a^Here, 5.0 equiv. **3a** was used. ^b^Reaction run at 0 °C. Ad, 1-adamantyl; cPr, cyclopropyl; Cy, cyclohexyl; iPr, isopropyl.

Using commercial dibenzothiophene *S*-oxide **2** and adapting straightforward conditions used widely for the synthesis of more common aryl sulfonium salts^54^, we have developed a robust protocol for the preparation of alkoxy sulfonium salts from a range of alcohols (Table 1). The alkoxy sulfonium salts were readily isolated and characterized, and the process was amenable to larger scale; 3.15 g of salt **1a** was prepared in 86% yield. Furthermore, **1a** was prepared on a 1-kg scale in an industrial setting (see ‘Large-scale, continuous 1,2-alkoxy-hydroxylation under photoflow conditions’ section). A range of alkoxy sulfonium salts—derived from primary and secondary alcohols—were prepared in high yield, although only a few tertiary alcohols could be engaged (for example, the formation of **1ai**), thus this is an area for future refinement of the approach. Crucially, products of alcohol oxidation were not observed^67^. The process embraced both inexpensive, simple feedstock alcohols (**1a**–**h**)—including a deuterated alcohol (**1b**)—and complex alcohols derived from an amino acid (**1s**) and a sugar (**1t**), or alcohols from the chiral pool (**1aa** and **1ab**). The mild reaction conditions allowed the presence of a number of important functional groups in the alcohol substrates to be tolerated, for example, halide (**1d**,**e**,**i**,**j**,**o**,**y,z** and **ad**), nitrile (**1k**), ester (**1s**,**x**,**aa**,**ab**), sulfone (**1l**), alkene (**1m**), alkyne (**1n**,**o**,**u**), tertiary alcohol (**1q**), acrylate (**1r**) and phthalimido (**1p**,**s**) motifs were compatible with alkoxy sulfonium salt synthesis, while a cyclic ketal delivered a sulfonium salt bearing the corresponding ketone functionality (**1af**). Crucially, enantiomerically pure alcohols, with the stereochemistry residing at (**1aa**,**ab**), or remote from the hydroxyl group (**1s**,**t**), gave alkoxy sulfonium salts with no loss of stereochemical integrity (Fig. 2).

**Table 1:**
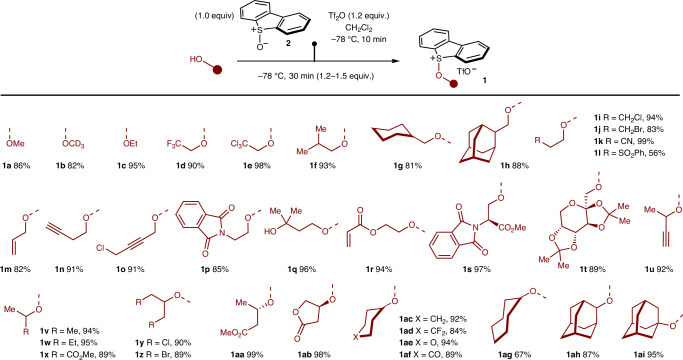
Alkoxy sulfonium salts prepared from alcohols

Tf_2_O, trifluoromethanesulfonic anhydride.

### Photocatalytic 1,2-alkoxy-hydroxylation of alkenes

With alkoxy sulfonium salts in hand, we optimized the photocatalytic selective construction of two C–O bonds across an alkene (Table 2). Using alkoxy sulfonium salt **1a** and 4-chlorostyrene **3a**, we arrived at the optimized conditions highlighted in entry 1; **4a** was obtained in 83% yield when using the photocatalyst **PC1** (0.1 mol%) in acetone, in the presence of water (2.0 equiv.) and Na_2_HPO_4_ (50 mol%), under light irradiation at 456 nm. The formation of **2**—probably by hydrolysis of **1a**—and **2′**—by direct homolysis of **1a**—were minimized by reducing the reaction temperature and using a photocatalyst. At higher reaction temperatures, in the presence of more alkoxy sulfonium salt, the formation of the aryl ketone product arising from oxidation of **4a** was observed. We ascribe this to HAT from the benzylic position of **4a** by the alkoxy radical. Under our optimized conditions, only traces of aryl ketone byproduct are observed (Supplementary Table S1). The reaction is only marginally sensitive to the reaction temperature (entry 2) and the presence of a base (entry 3)—aspects that we later exploited in the development of a photoflow process. In contrast, the reaction shuts down in solvents other than acetone (entry 4). Using alternative alkoxy sulfonium salts, conveniently derived from other sulfoxides (entry 5), or photocatalysts (entry 7), gave lower yields of product. Addition of water was not essential—probably owing to adventitious water in the acetone solvent—and higher loadings of water did not improve the yield (entry 6). A low yield of **4a** was obtained in the absence of a photocatalyst (entry 9), and no product was observed without light (entry 10).

**Table 2:**
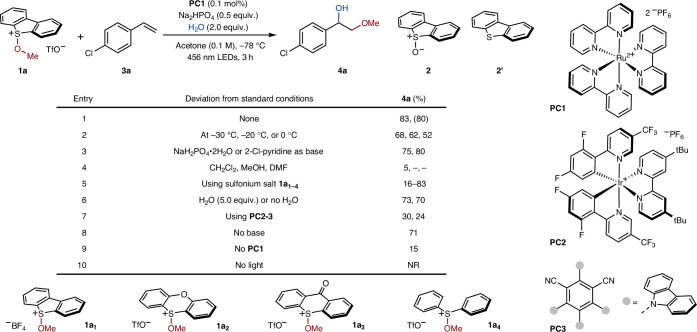
Optimization of the 1,2-alkoxy-hydroxylation of alkenes using alkoxy sulfonium salts

General reaction conditions involved **1a** (0.1 mmol), **3a** (0.2 mmol), **PC1** (0.1 mol%), Na_2_HPO_4_ (0.5 equiv.) and H_2_O (2.0 equiv.) dissolved in acetone (0.1 M) under irradiation by blue light-emitting diodes (LEDs; *λ*_max_ centred at 456 nm) at –78 °C for 3 h. Yields were determined by ^1^H NMR spectroscopy using mesitylene as an internal standard. Isolated yields are reported in parentheses. DMF, *N*,*N*-dimethylformamide; NR, no reaction; tBu, *tert*-butyl.

**Fig. 3:**
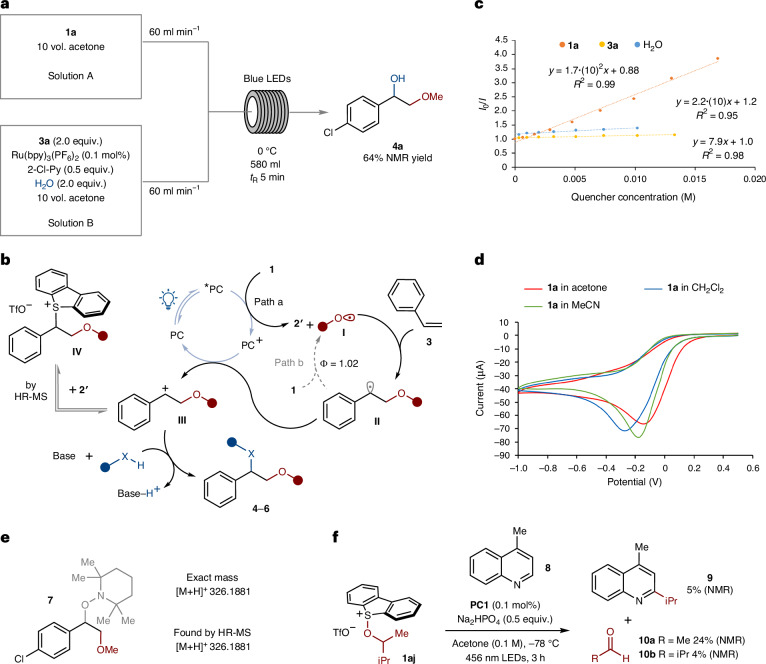
Reaction scale-up under photoflow conditions, proposed mechanism of alkene 1,2-alkoxy-hydroxylation and mechanistic studies supporting the proposed catalytic cycle. **a**, The optimized photocatalytic 1,2-alkoxy-hydroxylation of alkenes for a photoflow reactor. **b**, The proposed catalytic cycle. **c**, Stern–Volmer fluorescence quenching studies. **d**, Cyclic voltammetry studies. **e**, A radical intermediate trapping experiment. **f**, A control experiment supporting the formation of an alkoxy radical intermediate. bpy, 2,2′-bipyridine; Py, pyridine; *t*_R_, retention time.

We next assessed the scope of the photocatalysed alkoxy-hydroxylation of alkenes using methoxy sulfonium salt **1a** and varying the alkene partner (Table 3). Styrene derivatives with electron-withdrawing and electron-donating groups at the *para-* (**4a**–**m**,**z** and **4aa**–**ac**), *meta*- (**4n**–**t**) and *ortho*-positions (**4u**–**y**) were effective substrates. Products with polyfunctionalized aromatic rings were obtained in good to high yields regardless of the position and electronic properties of the substituents (**4ad**–**ah**). The benzocyclobutane motif (**4ai**) and both 1- and 2-substituted naphthyl-units were also tolerated (**4aj** and **4ak**). Engaging heteroaryl-containing substrates in photocatalysis often proves challenging owing to the sensitivity of heteroaromatic motifs to light. Pleasingly, the use of heteroaryl-containing alkenes delivers the corresponding medicinally relevant products (**4al**–**aq**), albeit in lower yields. The protocol extends to more challenging polysubstituted alkenes (**4ar**–**be**), for example, compound **4ar** was obtained in 83% yield despite the presence of weak allylic C–H bonds, which could be prone to competing HAT by the alkoxy radical intermediate. Other 1,2-disubstituted alkenes could be effectively engaged, including those bearing primary alkyl bromide (**4as**), ester (**4at**), ketone (**4au**), tertiary alkyl hydroxyl (**4ay**) and nitrile (**4az**) motifs. Cyclic 1,2-disubstituted alkenes afforded the corresponding products **4aw** and **4ax** in good yields. The process also proved effective for the 1,2-hydroxy-alkoxy functionalization of trisubstituted alkenes (**4av**). The formation of **4ay** as a single diasteroisomer may be rationalized by invoking the interaction of the pendant hydroxyl group with one face of the intermediate benzylic carbocation (Fig. 3b), with subsequent nucleophilic attack by water on the opposite face^68^. Interestingly, **4bc** was obtained in a moderate yield with no sign of radical ring-opening of the cyclopropyl substituent. This suggests a rapid oxidation of the intermediate benzyl radical to the corresponding carbocation in the catalytic cycle^69^. Finally, the benzazepine motif present in arginine vasopressine receptor antagonists^70^ can be conveniently decorated; **4be** was prepared in good yield and its structure confirmed by X-ray crystallographic analysis. Overall, this method tolerates various substitution patterns and functional groups, highlighting its wide applicability. The process is, however, currently specific for styrene-type alkenes, and no product formation was observed when alkyl substituted alkenes were employed; this may be because of parasitic HAT from allylic C–H bonds outcompeting the addition of the alkoxy radical to the C=C bond.

**Table 3:**
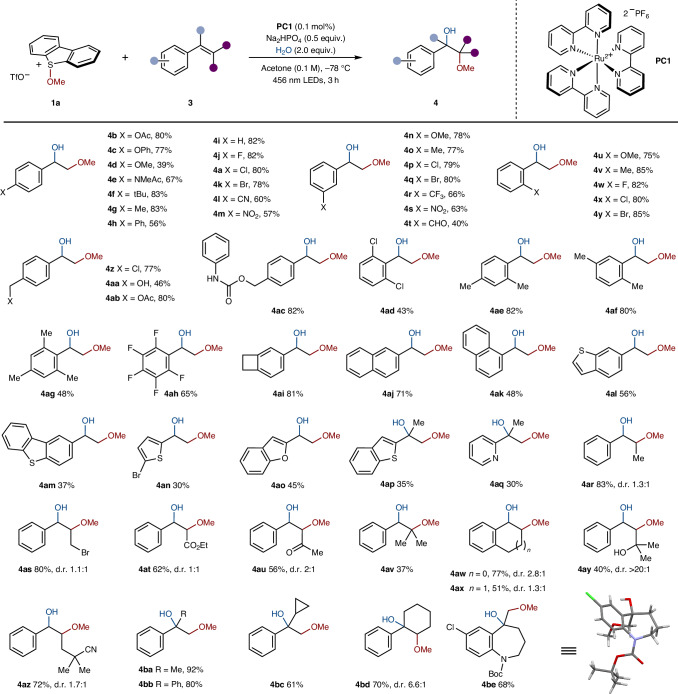
Alkene scope in the photocatalytic 1,2-alkoxy-hydroxylation of alkenes

Boc, *tert*-butoxycarbonyl; d.r., diastereoisomeric ratio; Ph, phenyl.

We next explored the scope of the photocatalytic process with respect to the alkoxy sulfonium salt using alkene **3a** (Fig. 2a). The reaction tolerated wide variation in the activated alcohol partner. Derivatives of simple alcohols, such as deuterated methanol (**4bf**) and ethanol (**4bg**), or more functionalized β-substituted derivatives (**4bh**–**bx**) were well-tolerated in the alkene difunctionalization. The reaction is not limited to primary alcohols; secondary (**4by**–**cl**) and tertiary alcohol derivatives (**4cm**) were also viable substrates. Importantly, products were obtained in good-to-high yield throughout, despite the known propensity of β-branched alkoxy radicals to undergo fragmentation^71^. A range of functional groups in the alcohol-derived partner were tolerated: for example, primary halides (**4bn**,**bo**,**bu**,**cc**,**cd**), nitriles (**4bm**), sulfones (**4bp**), phthalimides (**4bq** and protected amino acid **4bx**), hydroxyls (**4br**), ketones (**4cj**), α,β-unsaturated esters (**4bs**), alkenes (**4bt**), alkynes (**4bu**,**bv**,**cb**), esters (**4bx**,**ca**,**ce**), lactones (**4cf**) and ketals (in protected sugar **4bw**).

### Photocatalysed 1,2-dialkoxylation and 1,2-alkoxy-amidation of alkenes

By varying the nucleophile used to quench the carbocation intermediate, we expanded our approach to other 1,2-difunctionalization reactions. Pleasingly, minimal tuning of the reaction conditions delivered an alkene dialkoxylation procedure. The synthesis of unsymmetrical 1,2-diethers—with different alkyl groups on the two oxygen atoms—from diols, is non-trivial owing to the similar reactivity of the two hydroxy groups and the need for efficient and toxic alkylating agents^72^ (Fig. 2b, left). Using methoxy sulfonium salt **1a** and alkene **3a**, with a range of alcohol nucleophile partners, allowed selective access to several 1,2-diethers. Careful drying of the alcohol nucleophile ensured the formation of only traces of the 1,2-alkoxy-hydroxylation products. The alcohols used ranged from feedstock alcohols—such as methanol (**5a**), benzyl alcohol (**5e**), cyclobutanol (**5g**), *tert*-butanol (**5h**) and 2-methoxyethanol (**5d**), through alcohols containing functionality—such as bromoethanol (**5b**) and 2-(trimethylsilyl)ethanol (**5c**)—to complex alcohols such as a protected serine (**5f**). Highlighting the generality of this platform, the use of inexpensive nitriles as co-solvents resulted in the 1,2-alkoxy-amidation of alkene **3a** using alkoxy sulfonium salt **1a**^21,73^; amides **6a**–**h** were isolated in moderate yields alongside traces of the 1,2-alkoxy-hydroxylation products. Finally, an analogue of the paclitaxel side chain **6i** (Fig. 1a) was prepared from ethyl cinnamate when using benzonitrile as co-solvent (Fig. 2b, right).

### Large-scale, continuous 1,2-alkoxy-hydroxylation under photoflow conditions

Given the potential impact of developing a process that can be applied on industrial scale, the reaction was adapted from a batch process to an easily scaled photoflow process—a flow chemistry setting under light irradiation. We selected the reaction between methoxy sulfonium salt **1a** and styrene **3a** as a model system. Firstly, the synthesis of methoxy sulfonium salt **1a** was optimized for a 1-kg scale (Supplementary Information). Secondly, a rapid optimization campaign was performed taking into consideration key aspects of the method to make it compatible with the process chemistry flow reactor used and fulfilling requirements for its use in the pharmaceutical industry (Fig. 3a). Switching from an inorganic to an organic base removed any solubility concerns and ensured reaction homogeneity, while a 5-min residence time was found sufficient to achieve a good conversion and an excellent yield. The reaction temperature was raised from –30 °C to 0 °C, thus avoiding cryogenic conditions and unnecessary light diffraction from frost build-up on the reactor surface. As a stability study on the starting mixture indicated a slow decay of **1a** in the reaction mixture over 24 h, a two-feed system was established that consisted of two equal volume solutions of **1a** in acetone and styrene **3a** with photocatalyst, base and water, respectively. Both streams were mixed at the same flow rate and cooled to 0 °C, allowing for fresh mixing and pre-cooling of the active reagents before irradiation. Process safety experiments (differential scanning calorimetry, and accelerating rate calorimetry) for starting mixtures did not reveal safety concerns (Supplementary Information). With these minor modifications to the standard conditions, we achieved good yields of **4a** in the gram-scale photoflow reactor. The reaction was then scaled up to the kilogram-scale photoflow reactor, starting with 1.0 kg of **1a**, and obtaining **4a** in a 64% yield over 3.5 h.

### Mechanistic studies and the proposed catalytic cycle

Building on previous studies on the photochemistry of sulfonium salts by our team^51–57^ and by others^74–76^, we propose the catalytic cycle set out in Fig. 3b. Photoexcitation of **PC1** (${E}_{1/2}^{* \mathrm{II}/{\rm{I}}}$ of –0.81 eV versus saturated calomel electrode (SCE))^77^ under blue light triggers single electron transfer (SET) reduction of the alkoxy sulfonium salts **1** with the generation of alkoxy radicals **I**. The highly reactive alkoxy radicals add to the alkenes, generating benzylic radicals **II**, which are oxidized to the corresponding carbocations **III** (*E*_1/2_of +0.74 eV versus SCE)^78^ by **PC1**^**+**^ (${E}_{1/2}^{\mathrm{III}/\mathrm{II}}$ +1.29 eV versus SCE)^77^, closing the photocatalytic cycle (path a). Interception of the carbocation intermediates **III** by a nucleophile—water, alcohol or nitrile—furnishes the products of alkene 1,2-difunctionalization **4**–**6**. Mechanistic studies were conducted to probe the proposed catalytic cycle (Fig. 3c,d). Stern–Volmer fluorescence quenching studies revealed that the alkoxy sulfonium salt (for example, **1a**) is the only quencher of the excited state of **PC1**, supporting the SET reduction of the salt in the first step of the reaction. Cyclic voltammetry studies on **1a** found a more positive reduction potential (*E*_p_of −0.14 eV versus Ag/AgCl) than the photoexcited **PC1**, further supporting the proposed SET process. Addition of alkoxy radical **I** to the alkene was supported by a radical trapping experiment conducted under the standard reaction conditions in the presence of the radical scavenger 2,2,6,6-tetramethylpiperidine-1-oxyl. The 2,2,6,6-tetramethylpiperidine-1-oxyl-adduct **7**, resulting from interception of radical intermediates **II**, was detected by high-resolution mass spectrometry (HR-MS). The involvement of alkoxy radicals is also supported by the results obtained using sulfonium salt **1aj**, bearing a β-branched alkoxy motif; the introduction of 4-methyl-quinoline **8**, under otherwise standard reaction conditions, gave Minisci-type coupling product **9** and aldehydes **10a**,**b** arising from radicals formed by β-scission of the alkoxy radical species^71^. The intermediacy of benzylic carbocations **III** is also corroborated by the detection by HR-MS of a sulfonium salt corresponding to **IV** formed by reaction of **III** with dibenzothiophene **2′** liberated in the early stages of the catalytic cycle. A quantum yield (*ø*) of 1.02 was measured for the coupling of **1a** and **3a** to give **4a**, thus suggesting the involvement—at least partially—of a radical-chain mechanism, in which **II** reduces the starting alkoxy sulfonium salt **1a** (Fig. 3b, path b).

## Conclusions

Convenient activation of both simple and complex alcohols as alkoxy sulfonium salts provides a platform for alkoxy radical generation from alcohols under mild photocatalytic conditions. Crucially, our alcohol activation involves addition of the alcohol hydroxyl group to the sulfur of a commercial sulfoxide in an interrupted Pummerer-type process, rather than displacement of alkyl halides by O-containing activating agents in S_N_1 and S_N_2 processes. Thus, alcohol activation is more general in that greater structural variation and functionality is tolerated in the alkoxy radical precursors. We have applied the approach in an efficient, modular photocatalytic difunctionalization of alkenes, using alcohols activated directly as alkoxy sulfonium salts, that delivers 1,2-hydroxy ethers, symmetrical and unsymmetrical 1,2-diethers and 1,2-amido ethers. The mild nature of the process results in good functional group tolerance and scope, and the method can be easily adapted from laboratory to industrial scale using a photoflow system; a product of alkene difunctionalization was obtained in 3.5 h from a 1-kg scale process. Preliminary mechanistic studies suggest a photocatalytic cycle involving reduction of the alkoxy sulfonium salts to give alkoxy radicals that undergo intermolecular addition to alkenes. Subsequent oxidation of the carbon-centred radical adducts regenerates the photocatalyst and closes the catalytic cycle. We believe that straightforward activation of alcohols for use in photochemistry, through alkoxysulfonium salt formation, will facilitate future developments in chemical synthesis.

## Methods

### General procedure for the photocatalytic 1,2-alkoxy-hydroxylation of alkenes

A dry tube equipped with a stirring bar was charged with alkoxy sulfonium salt (0.10 mmol, 1.0 equiv.), Na_2_HPO_4_ (7 mg, 0.05 mmol, 0.5 equiv.) and alkene (if solid) (0.20 mmol, 2.0 equiv.). The tube was capped with an aluminium crimp seal with septum (polytetrafluoroethylene (PTFE)/butyl), then evacuated under high vacuum and backfilled with N_2_ (three times). Degassed Ru(bpy)_3_(PF_6_)_2_ in an acetone stock solution (1.0 ml, 0.1 mol%), and alkene (if liquid) (0.20 mmol, 2.0 equiv.) and H_2_O (4 µl, 0.20 mmol, 2.0 equiv.) were sequentially added. The vial was purged with a stream of N_2_ and the lid sealed with parafilm and placed approximately 5 cm from blue LEDs. The blue LEDs were switched on and the mixture was stirred under irradiation at −78 °C for 3 h. The tube was allowed to warm to room temperature and was opened, and the reaction mixture was concentrated and purified by column chromatography on silica gel, eluting with hexane–ethyl acetate.

### General procedure for the photocatalytic 1,2-dialkoxylation of alkenes

A dry tube equipped with a stirring bar was charged with alkoxy sulfonium salt (0.10 mmol, 1.0 equiv.), Na_2_HPO_4_ (7 mg, 0.05 mmol, 0.5 equiv.) and alkene (if solid) (0.20 mmol, 2.0 equiv.). The tube was capped with an aluminium crimp seal with septum (PTFE/butyl), then evacuated under high vacuum and backfilled with N_2_ (three times). Degassed Ru(bpy)_3_(PF_6_)_2_ in a CH_2_Cl_2_ stock solution (1.0 ml, 0.1 mol%), and alkene (if liquid) (0.20 mmol, 2.0 equiv.) and alcohol (0.50 mmol, 5.0 equiv.) were sequentially added. The vial was purged with a stream of N_2_ and the lid sealed with parafilm and placed approximately 5 cm from blue LEDs. The blue LEDs were switched on and the mixture was stirred under irradiation at −78 °C for 3 h. The tube was allowed to warm to room temperature and was opened, and the reaction mixture was concentrated and purified by column chromatography on silica gel, eluting with hexane–ethyl acetate.

### General procedure for the photocatalytic 1,2-alkoxy-amidation of alkenes

A dry tube equipped with a stirring bar was charged with alkoxy sulfonium salt (0.10 mmol, 1.0 equiv.), Na_2_HPO_4_ (7 mg, 0.05 mmol, 0.5 equiv.) and alkene (if solid) (0.20 mmol, 2.0 equiv.). The tube was capped with an aluminium crimp seal with septum (PTFE/butyl), then evacuated under high vacuum and backfilled with N_2_ (three times). Degassed Ru(bpy)_3_(PF_6_)_2_ in an alkylnitrile stock solution (0.5 ml, 0.1 mol%), and alkene (if liquid) (0.20 mmol, 2.0 equiv.) and H_2_O (2 µl, 0.10 mmol, 1.0 equiv.) were sequentially added. The vial was purged with a stream of N_2_ and the lid sealed with parafilm and placed approximately 5 cm from blue LEDs. The blue LEDs were switched on and the mixture was stirred under irradiation at −30 °C for 3 h. The tube was allowed to warm to room temperature and was opened, and the reaction mixture was concentrated and purified by column chromatography on silica gel, eluting with hexane–ethyl acetate.

## Online content

Any methods, additional references, Nature Portfolio reporting summaries, source data, extended data, supplementary information, acknowledgements, peer review information; details of author contributions and competing interests; and statements of data and code availability are available at 10.1038/s41557-025-02003-7.

## Supplementary information

Supplementary Information Supplementary Figs. 1–20, Supplementary Tables 1–13 and the synthesis of starting materials and products, data and NMR spectra.

## Data Availability

All data are provided in the main text or Supplementary Information, including experimental procedures, copies of NMR spectra and X-ray structure reports. Structural parameters are available from the Cambridge Crystallographic Data Centre (CCDC) under reference numbers 2432415 (**4be**) and 2432693 (**4cf**).

## References

[1] Kanda, Y. et al. Two-phase synthesis of taxol. *J. Am. Chem. Soc.* **142**, 10526–10533 (2020). 32406238 10.1021/jacs.0c03592 PMC8349513

[2] Yu, J., Cao, Y., Yu, H. Z. & Wang, J. J. A concise and efficient synthesis of dapagliflozin. *Org. Process Res. Dev.* **23**, 1458–1461 (2019).

[3] Gold, E. H. et al. Synthesis and comparison of some cardiovascular properties of the stereoisomers of labetalol. *J. Med. Chem.* **25**, 1363–1370 (1982). 6128421 10.1021/jm00353a017

[4] Yang, P. et al. Optimized synthetic route for enantioselective preparation of (*S*)-metolachlor from commercially available (*R*)-propylene oxide. *Org. Process Res. Dev.* **21**, 1682–1688 (2017).

[5] Carey, F. A. & Sundberg, R. J. *Advanced Organic Chemistry, Part B: Reaction and Synthesis* 5th edn (Springer, 2007).

[6] Grigoropoulou, G., Clark, J. H. & Elings, J. A. Recent developments on the epoxidation of alkenes using hydrogen peroxide as an oxidant. *Green Chem.* **5**, 1–7 (2003).

[7] Zhang, P., Wang, T. & Gong, J. Advances in electrochemical oxidation of olefins to epoxides. *CCS Chem.* **5**, 1028–1042 (2023).

[8] Shellhamer, D. F. et al. Ionic reaction of halogens with terminal alkenes: the effect of electron-withdrawing fluorine substituents on the bonding of halonium ions. *J. Org. Chem.* **68**, 3932–3937 (2003). 12737574 10.1021/jo030030v

[9] Hennecke, U. New catalytic approaches towards the enantioselective halogenation of alkenes. *Chem. Asian J.* **7**, 456–465 (2012). 22315237 10.1002/asia.201100856

[10] Bodkina, J. A. & McLeod, M. D. The sharpless asymmetric aminohydroxylation. *Perkin Trans. 1* **2002**, 2733–2746 (2002).

[11] Yin, G., Mu, X. & Liu, G. Palladium(II)-catalyzed oxidative difunctionalization of alkenes: bond forming at a high-valent palladium center. *Acc. Chem. Res.* **49**, 2413–2423 (2016). 27739689 10.1021/acs.accounts.6b00328

[12] Dhungana, R. K., Shekhar, K. C., Basnet, P. & Giri, R. Transition metal-catalyzed dicarbofunctionalization of unactivated olefins. *Chem. Rec.* **18**, 1314–1340 (2018). 29517841 10.1002/tcr.201700098

[13] Lan, X.-W., Wang, N.-X. & Xing, Y. Recent advances in radical difunctionalization of simple alkenes. *Eur. J. Org. Chem.* **2017**, 5821–5851 (2017).

[14] Lee, J. H., Choi, S. & Hong, K. B. Alkene difunctionalization using hypervalent iodine reagents: progress and developments in the past ten years. *Molecules* **24**, 2634 (2019). 31331092 10.3390/molecules24142634 PMC6680546

[15] Jiang, H. & Studer, A. Intermolecular radical carboamination of alkenes. *Chem. Soc. Rev.* **49**, 1790–1811 (2020). 32055811 10.1039/c9cs00692c

[16] Patel, M., Desai, B., Sheth, A., Dholakiya, B. Z. & Naveen, T. Recent advances in mono- and difunctionalization of unactivated olefins. *Asian J. Org. Chem.* **10**, 3201–3232 (2021).

[17] Wu, Z., Hu, M., Li, J., Wu, W. & Jiang, H. Recent advances in aminative difunctionalization of alkenes. *Org. Biomol. Chem.* **19**, 3036–3054 (2021). 33734255 10.1039/d0ob02446e

[18] Erchinger, J. E. et al. EnT-mediated N–S bond homolysis of a bifunctional reagent leading to aliphatic sulfonyl fluorides. *J. Am. Chem. Soc.* **145**, 2364–2374 (2023). 36652725 10.1021/jacs.2c11295

[19] Tan, G. et al. Energy transfer-enabled unsymmetrical diamination using bifunctional nitrogen-radical precursors. *Nat. Catal.* **5**, 1120–1130 (2022).

[20] Liu, Y., Liu, H., Liu, X. & Chen, Z. Recent advances in photoredox-catalyzed difunctionalization of alkenes. *Catalysts* **13**, 1056 (2023).

[21] Cao, M.-Y., Ren, X. & Lu, Z. Olefin difunctionalizations via visible light photocatalysis. *Tetrahedron Lett.* **56**, 3732–3742 (2015).

[22] Zhou, W.-J., Yang, Z. & Xiao, B. Redox-neutral photocatalytic germylative difunctionalization of unactivated olefins via selective radical capture by Ge(II). *ACS Catal.* **15**, 2150–2157 (2025).

[23] Dong, C. et al. Photocatalytic dihydroxylation of light olefins to glycols by water. *Nat. Commun.* **15**, 8210 (2024). 39294117 10.1038/s41467-024-52461-9 PMC11410969

[24] Lai, S.-Q., Wei, B.-Y., Wang, J.-W., Yu, W. & Han, B. Photocatalytic anti-Markovnikov radical hydro- and aminooxygenation of unactivated alkenes tuned by ketoxime carbonates. *Angew. Chem. Int. Ed.* **60**, 21997–22003 (2021). 10.1002/anie.202107118 34255913

[25] Luo, X.-L. et al. Photocatalytic 1,2-iminosulfonylation and remote 1,6-iminosulfonylation of olefins. *Org. Lett.* **25**, 1742–1747 (2023). 36883883 10.1021/acs.orglett.3c00437

[26] Sookezian, A. & Molander, G. A. Photoinduced vicinal 1,2-difunctionalization of olefins for the synthesis of alkyl sulfonamides. *Org. Lett.* **25**, 1014–1019 (2023). 36745531 10.1021/acs.orglett.3c00182

[27] Nan, G. & Yue, H. Visible-light-promoted difunctionalization of olefins leading to α-thiocyanato ketones. *Synlett* **29**, 1340–1345 (2018).

[28] Miyazawa, K., Koike, T. & Akita, M. Aminohydroxylation of olefins with iminopyridinium ylides by dual Ir photocatalysis and Sc(OTf)_3_ catalysis. *Tetrahedron* **72**, 7813–782 (2016).

[29] Hampton, C., Simonetti, M. & Leonori, D. Olefin dihydroxylation using nitroarenes as photoresponsive oxidants. *Angew. Chem. Int. Ed.* **62**, e202214508 (2023). 10.1002/anie.202214508 PMC10107662 36509705

[30] Govaerts, S. et al. Photoinduced olefin diamination with alkylamines. *Angew. Chem. Int. Ed.* **59**, 15021–15028 (2020). 10.1002/anie.202005652 PMC7497254 32432808

[31] Margrey, K. A. & Nicewicz, D. A. A general approach to catalytic alkene anti-Markovnikov hydrofunctionalization reactions via acridinium photoredox catalysis. *Acc. Chem. Res.* **49**, 1997–2006 (2016). 27588818 10.1021/acs.accounts.6b00304

[32] Asano, Y., Nagasawa, Y., Yamaguchi, E. & Itoh, A. Aerobic photooxidative synthesis of β-alkoxy monohydroperoxides using an organo photoredox catalyst controlled by a base. *Chem. Asian J.* **13**, 409–412 (2018). 29327427 10.1002/asia.201701742

[33] Luo, M.-J., Xiao, Q. & Li, J.-H. Electro-/photocatalytic alkene-derived radical cation chemistry: recent advances in synthetic applications. *Chem. Soc. Rev.* **51**, 7206–7237 (2022). 35880555 10.1039/d2cs00013j

[34] Zhang, S. et al. Substrate-dependent electrochemical dimethoxylation of olefins. *Adv. Synth. Catal.* **361**, 485–489 (2019).

[35] Barthelemy, A.-L., Tuccio, B., Magnier, E. & Dagousset, G. Alkoxyl radicals generated under photoredox catalysis: a strategy for anti-markovnikov alkoxylation reactions. *Angew. Chem. Int. Ed.* **57**, 13790–13794 (2018). 10.1002/anie.201806522 30084188

[36] Tsui, E., Wang, H. & Knowles, R. R. Catalytic generation of alkoxy radicals from unfunctionalized alcohols. *Chem. Sci.* **11**, 11124–11141 (2020). 33384861 10.1039/d0sc04542j PMC7747465

[37] Chang, L., An, Q., Duan, L., Feng, K. & Zuo, Z. Alkoxy radicals see the light: new paradigms of photochemical synthesis. *Chem. Rev.* **122**, 2429–2486 (2022). 34613698 10.1021/acs.chemrev.1c00256

[38] Ali, M., Sewell, S., Li, J. & Wang, T. Recent advances in application of alkoxy radical in organic synthesis. *Organics* **4**, 459–489 (2023). 38084108 10.3390/org4040033 PMC10712650

[39] El Gehani, A. A. M. A., Maashi, H. A., Harnedy, J. & Morrill, L. C. Electrochemical generation and utilization of alkoxy radicals. *Chem. Commun.* **59**, 3655–3664 (2023). 10.1039/d3cc00302g 36877137

[40] Wu, X. et al. Metal-free alcohol-directed regioselective heteroarylation of remote unactivated C(*sp*^3^)–H bonds. *Nat. Commun.* **9**, 3343 (2018). 30131495 10.1038/s41467-018-05522-9 PMC6104081

[41] Xiong, Y., Zhang, X., Guoa, H.-M. & Wu, X. Photoredox/persistent radical cation dual catalysis for alkoxy radical generation from alcohols. *Org. Chem. Front.* **9**, 3532–3539 (2022).

[42] Rivero, A. R., Fodran, P., Ondrejková, A. & Wallentin, C. J. Alcohol etherification via alkoxy radicals generated by visible-light photoredox catalysis. *Org. Lett.* **22**, 8436–8440 (2020). 33040526 10.1021/acs.orglett.0c03058 PMC7653678

[43] Yang, Z. et al. Tuning the reactivity of alkoxyl radicals from 1,5-hydrogen atom transfer to 1,2-silyl transfer. *Nat. Commun.* **12**, 2131 (2021). 33837201 10.1038/s41467-021-22382-y PMC8035221

[44] Hareram, M. D. et al. Electrochemical deconstructive functionalization of cycloalkanols via alkoxy radicals enabled by proton-coupled electron transfer. *Org. Lett.* **24**, 3890–3895 (2022). 35604008 10.1021/acs.orglett.2c01552 PMC9171832

[45] Minisci, F. & Galli, R. Reactivity of hydroxy and alkoxy radicals in presence of olefins and oxidation-reduction systems. introduction of azido, chloro and acyloxy groups in allylic position and azido-chlorination of olefins. *Tetrahedron Lett.* **4**, 357–360 (1963).

[46] Zhang, J. et al. Visible-light-induced alkoxyl radicals enable α-C(*sp*^3^)-H bond allylation. *iScience* **23**, 100755 (2020). 31884167 10.1016/j.isci.2019.100755 PMC6941871

[47] Gallego-Gamo, A. et al. Direct synthesis of 2-hydroxytrifluoroethylacetophenones via organophotoredox-mediated net-neutral radical/polar crossover. *J. Org. Chem.* **89**, 11682–11692 (2024). 39087492 10.1021/acs.joc.4c01419 PMC11334190

[48] Finis, D. S. & Nicewicz, D. A. Alkoxy radical generation mediated by sulfoxide cation radicals for alcohol-directed aliphatic C–H functionalization. *J. Am. Chem. Soc.* **146**, 16830–16837 (2024). 10.1021/jacs.4c05052 PMC11624318 38847590

[49] Tsui, E., Metrano, A. J., Tsuchiya, Y. & Knowles, R. R. Catalytic hydroetherification of unactivated alkenes enabled by proton-coupled electron transfer. *Angew. Chem. Int. Ed.* **59**, 11845–11849 (2020). 10.1002/anie.202003959 PMC7451027 32227658

[50] Li, P., Duan, L., Lin, Y., Chu, L. & Zuo, Z. Modulating electron transfer via cerium photocatalysis for alkoxy radical-mediated selective hydroetherification. *Angew. Chem. Int. Ed.* **64**, e202501949 (2025). 10.1002/anie.202501949 40033774

[51] Péter, A., Perry, G. J. P. & Procter, D. J. Radical C–C bond formation using sulfonium salts and light. *Adv. Synth. Catal.* **362**, 2135–2142 (2020).

[52] Pulis, A. P. & Procter, D. J. C–H coupling reactions directed by sulfoxides: teaching an old functional group new tricks. *Angew. Chem. Int. Ed.* **55**, 9842–9860 (2016). 10.1002/anie.201601540 27409984

[53] Smith, L. H. S., Coote, S. C., Sneddon, H. F. & Procter, D. J. Beyond the Pummerer reaction: recent developments in thionium ion chemistry. *Angew. Chem. Int. Ed.* **49**, 5832–5844 (2010). 10.1002/anie.201000517 20583014

[54] Dewanji, A. et al. A general arene C–H functionalization strategy via electron donor-acceptor complex photoactivation. *Nat. Chem.* **15**, 43–52 (2023). 36471045 10.1038/s41557-022-01092-y

[55] van Dalsen, L., Brown, R. E., Rossi-Ashton, J. A. & Procter, D. J. Sulfonium salts as acceptors in electron donor-acceptor complexes. *Angew. Chem. Int. Ed.* **62**, e202303104 (2023). 10.1002/anie.202303104 PMC10952135 36959098

[56] Zhao, H., Cuomo, V. D., Rossi-Ashton, J. A. & Procter, D. J. Aryl sulfonium salt electron donor-acceptor complexes for halogen atom transfer: isocyanides as tuneable coupling partners. *Chem* **10**, 1240–1251 (2024).

[57] Aukland, M. H., Šiaučiulis, M., West, A., Perry, G. J. P. & Procter, D. J. Metal-free photoredox-catalysed formal C–H/C–H coupling of arenes enabled by interrupted Pummerer activation. *Nat. Catal.* **3**, 163–169 (2020).

[58] Johnson, C. R. & Phillips, W. G. Reactions of alkoxides with alkoxysulfonium salts. *Tetrahedron Lett.* **6**, 2101–2104 (1965).

[59] Johnson, C. R. & Phillips, W. G. Reactions of alkoxysulfonium salts with alkoxides. *J. Org. Chem.* **32**, 1926–1931 (1967).

[60] Johnson, C. R. & Jones, M. P. Preparation of alkoxysulfonium salts by oxidation of sulfides with positive halogen compounds. *J. Org. Chem.* **32**, 2014–2016 (1967).

[61] Khuddus, M. A. & Swern, D. Chemistry of epoxides. XXIX. Alkoxysulfonium salts from dimethyl sulfoxide and epoxides. preparation, characterization, reactions, and mechanistic studies. *J. Am. Chem. Soc.* **95**, 8393–8402 (1973).

[62] Glass, R. S., Hojjatie, M., Setzer, W. N. & Wilson, G. S. Stereoselective synthesis, structural studies, and hydrolysis of tricyclic alkoxysulfonium salts. *J. Org. Chem.* **51**, 1815–1820 (1986).

[63] Dupont Durst, H., Zubrick, J. W. & Kieczykowski, G. R. A mild, selective reduction of alkoxysulfonium salts: reductions of sulfoxides using sodium cyanohydridoborate and crown ethers. *Tetrahedron Lett.* **15**, 1777–1780 (1974).

[64] Andersen, K. K., Cinquini, M. & Papanikolaou, N. E. Synthesis of triaryalsulfonium salts from diarylethoxysulfonium salts. *Tetrahedron Lett.* **45**, 5445–5449 (1966).

[65] Andersen, K. K., Cinquini, M. & Papanikolaou, N. E. The synthesis and stereochemistry of triarylsulfonium salts. *J. Org. Chem.* **35**, 706–710 (1970).

[66] Andersen, K. K., Caret, R. L. & Ladd, D. L. Synthesis of optically active dialkylarylsulfonium salts from alkyl aryl sulfoxides. *J. Org. Chem.* **41**, 3096–3100 (1976).

[67] Omura, K. & Swern, D. Oxidation of alcohols by “activated” dimethyl sulfoxide. A preparative, steric and mechanistic study. *Tetrahedron* **34**, 1651–1660 (1978).

[68] Ramdular, A. & Woerpel, K. A. Diastereoselective substitution reactions of acyclic β-alkoxy acetals via electrostatically stabilized oxocarbenium ion intermediates. *Org. Lett.* **24**, 3217–3222 (2022). 35446592 10.1021/acs.orglett.2c01004 PMC9817112

[69] Newcomb, M. *Radical Kinetics and Clocks. Encyclopedia of Radicals in Chemistry, Biology and Materials* (Wiley, 2012).

[70] Kondo, K. et al. 7-Chloro-5-hydroxy-1-[2-methyl-4-(2-methylbenzoylamino)benzoyl]-2,3,4,5-tetrahydro-1*H*-1-benzazepine (OPC-41061): a potent, orally active nonpeptide arginine vasopressin V2 receptor antagonist. *Bioorg. Med. Chem.* **7**, 1743–1754 (1999). 10482466 10.1016/s0968-0896(99)00101-7

[71] Murakami, M. & Ishida, N. β-Scission of alkoxy radicals in synthetic transformations. *Chem. Lett.* **46**, 1692–1700 (2017).

[72] Ren, B., Yana, N. & Gan, L. Regioselective alkylation of carbohydrates and diols: a cheaper iron catalyst, new applications and mechanism. *RSC Adv.* **7**, 46257–46262 (2017).

[73] Ritter, J. J. & Minieri, P. P. A new reaction of nitriles. I. Amides from alkenes and mononitriles. *J. Am. Chem. Soc.* **70**, 4045–4048 (1948). 18105932 10.1021/ja01192a022

[74] Dektar, J. L. & Hacker, N. P. Photochemistry of triarylsulfonium salts. *J. Am. Chem. Soc.* **112**, 6004–6015 (1990).

[75] Kozhushkov, S. I. & Alcarazo, M. Synthetic applications of sulfonium salts. *Eur. J. Inorg. Chem.* **2020**, 2486–2500 (2020). 32742188 10.1002/ejic.202000249 PMC7386937

[76] Zhao, H., Cuomo, V. D., Tian, W., Romano, C. & Procter, D. J. Light-assisted functionalisation of aryl radicals towards metal-free cross-coupling. *Nat. Rev. Chem.* **9**, 61–80 (2025). 39548311 10.1038/s41570-024-00664-5

[77] Prier, C. K., Rankic, D. A. & MacMillan, D. W. C. Visible light photoredox catalysis with transition metal complexes: applications in organic synthesis. *Chem. Rev.* **113**, 5322–5363 (2013). 23509883 10.1021/cr300503r PMC4028850

[78] Grampp, G., Mureşanu, C. & Landgraf, S. Photomodulated voltammetry investigations on the benzyl radical. *Electrochim. Acta* **53**, 3149–3155 (2008).

