# Supplementary material for: Activation of alcohols as sulfonium salts in the photocatalytic hetero-difunctionalization of alkenes
Source: Nat Chem. 2025 Nov 26;18(2):398–406. doi: 10.1038/s41557-025-02003-7 (PMC12872449; doi:10.1038/s41557-025-02003-7)
Supplement: Supplementary file 1 — Supplementary Figs. 1–20, Supplementary Tables 1–13 and the synthesis of starting materials and products, data and NMR spectra. [file 41557_2025_2003_MOESM1_ESM.pdf]

nature chemistry

Supplementary information

https://doi.org/10.1038/s41557-025-02003-7

Activation of alcohols as sulfonium salts in the photocatalytic hetero-difunctionalization of alkenes

In the format provided by the authors and unedited

Table of Contents

1 General Experimental Details 2

2 Starting Material Synthesis 2

3 Reaction Optimization 30

4 Substrate Scope 34

5 Picture of Reaction Set-up 79

6 Scale-up Experiment 80

6.1 Synthesis of Alkoxy Sulfonium Salt 1a 80

6.2 Stability Studies on 1a under the Reaction Conditions 84

6.3 General Procedures for Photoflow Reactions 86

6.4 Optimization of Batch Conditions to Continuous Flow Setup 88

6.5 Analysis Methods and Process Safety Assessment 92

6.6 Large Scale Photoflow Development 95

7 Mechanistic Considerations 100

7.1 Detection of Carbon Radical Intermediate Experiment 100

7.2 O^{18} Isotope Labeling Studies 102

7.3 Evidence for the Fragmentation of Alkoxy Radicals 103

7.4 Cyclic Voltammetry Studies 106

7.5 Emission Quenching Studies 108

7.6 Quantum Yield (Φ) Determination 111

7.7 X-Ray Structures 115

8 NMR Spectra 119

9 References 315

1 General Experimental Details

All required fine chemicals were used directly without purification unless stated otherwise. All air and moisture sensitive reactions were carried out under nitrogen atmosphere using standard Schlenk manifold techniques. All solvents were purchased at 99.8% purity. ^{1}H and ^{13}C Nuclear Magnetic Resonance (NMR) spectra were acquired at various field strengths as indicated and were referenced to $CHCl_3$ (7.26 and 77.2 ppm for ^{1}H and ^{13}C, respectively). ^{1}H NMR coupling constants are reported in Hertz. Data are reported as follows: chemical shift, integration, multiplicity (s = singlet, br s = broad singlet, d = doublet, t = triplet, q = quartet, qi = quintet, sx = sextet, sp = septet, m = multiplet, dd = doublet of doublets, etc.), proton assignment (determined by 2D NMR experiments: NOESY, HSQC and HMBC) where possible and diagnostic. High-resolution mass spectra were obtained using a JEOL JMS-700 spectrometer or a Fissions VG Trio 2000 quadrupole mass spectrometer. Spectra were obtained using electron impact ionization (EI) and chemical ionization (CI) techniques, or positive electrospray (ES). Analytical TLC was carried out using aluminum backed plates pre-coated (0.25 mm) with Merck Silica Gel 60 F254. Compounds were visualized by exposure to UV-light or by dipping the plates in permanganate ($KMnO_4$) stain followed by heating. Column chromatography was performed using Merck Silica Gel 60 (40–63 µm). All mixed solvent eluents are reported as v/v solutions. Absorption and emission spectra were obtained using a Horiba Duetta spectrometer and 1 mm High Precision Cells made of quartz from Hellma Analytics. The LEDs used are Kessil PR 160 456 nm. All the reactions were conducted in CEM 10 mL glass microwave tubes.

2 Starting Material Synthesis

General Procedure for the Preparation of Alkoxy Sulfonium Salts – GP1

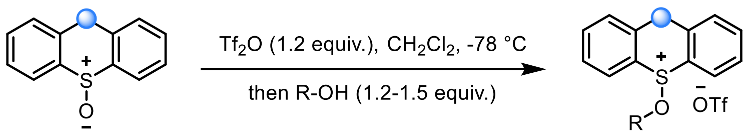

To a solution of sulfoxide (1.0 equiv.) in anhydrous CH_2Cl_2 (0.1 M) at –78 °C in the dark was added triflic anhydride (1.2 equiv.), and the reaction mixture was stirred for 10 min before alcohol (1.2 or 1.5 equiv.) was added. After 30 min, the reaction mixture was slowly warmed to room temperature, and stirred for another 30 min, then the solvent was removed in vacuo (keeping the water bath temperature around 30 °C) and to the residue was added Et_2O. The

resulting precipitate was decanted, washed with Et_2O twice, and dried in high vacuo to afford the alkoxy sulfonium salt.

5-Methoxy-5*H*-dibenzo[*b*,*d*]thiophen-5-ium triflate (1a)

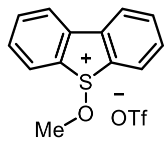

Following **GP1**, dibenzo[*b*,*d*]thiophene 5-oxide (2.00 g, 10.0 mmol, 1.0 equiv.) and methanol (0.61 mL, 15.0 mmol, 1.5 equiv.) gave **1a** (3.15 g, 86%) as a solid. ^{1}H NMR (400 MHz, acetone-d_6) δ 8.59 (2H, d, *J* = 7.9 Hz), 8.38 (2H, d, *J* = 7.8 Hz), 8.07 (2H, td, *J* = 7.7, 1.1 Hz), 7.87 (2H, td, *J* = 7.8, 1.1 Hz), 3.70 (3H, s); ^{13}C NMR (101 MHz, acetone-d_6) δ 142.0, 138.4, 132.7, 131.7, 130.4, 125.2, 58.7; ^{19}F NMR (376 MHz, acetone-d_6) δ −78.8; HRMS (ESI): Found $(M{-}OTf)^+$ 215.0518, $C_{13}H_{11}OS$ requires 215.0525.
The quaternary carbon corresponding to the CF_3 in the triflate counter anion was not observed, though its presence was confirmed by ^{19}F NMR.

5-Methoxy-5*H*-dibenzo[*b*,*d*]thiophen-5-ium tetrafluoroborate (1a$_1$)

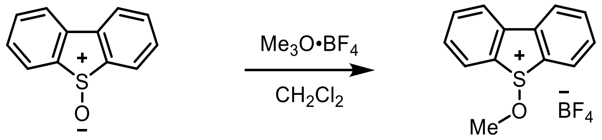

To a solution of dibenzo[*b*,*d*]thiophene 5-oxide (0.40 g, 2.0 mmol, 1.0 equiv.) in anhydrous CH_2Cl_2 (0.2 M) at room temperature was added trimethyloxonium tetrafluoroborate (0.33 g, 2.2 mmol, 1.1 equiv.), and the reaction mixture was stirred for 2 h at room temperature in the dark. The solvent was removed in vacuo (keeping the water bath temperature around 30 °C) and to the residue was added Et_2O (10 mL). The resulting precipitate was decanted, washed with Et_2O (2 × 10 mL), and dried in high vacuo to afford the methoxysulfonium salt **1a$_1$** (0.50 g, 83%) as a solid. ^{1}H NMR (500 MHz, acetone-d_6) δ 8.58 (2H, d, *J* = 7.9 Hz), 8.37 (2H, d, *J* = 7.8 Hz), 8.07 (2H, td, *J* = 7.6, 1.1 Hz), 7.87 (2H, td, *J* = 7.7, 1.1 Hz), 3.70 (3H, s); ^{13}C NMR (126 MHz, acetone-d_6) δ 141.9, 138.5, 132.8, 131.7, 130.3, 125.2, 58.8; ^{19}F NMR (471 MHz, acetone-d_6) δ −151.3; HRMS (ESI): Found $(M{-}BF_4)^+$ 215.0516, $C_{13}H_{11}OS$ requires 215.0525.

10-Methoxy-10*H*-phenoxathiin-10-ium triflate (1a$_2$)

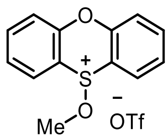

Following **GP1**, phenoxathiine 10-oxide (0.54 g, 2.5 mmol, 1.0 equiv.) and methanol (0.15 mL, 3.75 mmol, 1.5 equiv.) gave **1a$_2$** (0.89 g, 94%) as a solid. ^{1}H NMR (500 MHz, acetone-d_6) δ 8.58 (2H, dd, J = 8.1, 1.6 Hz), 8.26 (2H, ddd, J = 8.8, 7.3, 1.6 Hz), 7.95 (2H, dd, J = 8.6, 1.1 Hz), 7.86 (2H, ddd, J = 8.3, 7.3, 1.1 Hz), 3.63 (3H, s); ^{13}C NMR (126 MHz, acetone-d_6) δ 152.7, 140.5, 133.7, 128.2, 120.8, 106.7, 57.8; ^{19}F NMR (471 MHz, acetone-d_6) δ −78.9; HRMS (ESI): Found (M−OTf)$^+$ 231.0468, $C_{13}H_{11}O_2S$ requires 231.0474.
The quaternary carbon corresponding to the CF_3 in the triflate counter anion was not observed, though its presence was confirmed by ^{19}F NMR.

10-Methoxy-9-oxo-9,10-dihydrothioxanthylium triflate (1a$_3$)

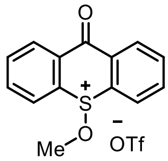

Following **GP1**, 9*H*-thioxanthen-9-one 10-oxide (0.46 g, 2.0 mmol, 1.0 equiv.) and methanol (0.12 mL, 3.0 mmol, 1.5 equiv.) gave **1a$_3$** (0.68 g, 87%) as a solid. ^{1}H NMR (500 MHz, acetone-d_6) δ 8.71–8.62 (4H, m), 8.33–8.27 (4H, m), 3.89 (3H, s); ^{13}C NMR (126 MHz, acetone-d_6) δ 177.9, 138.2, 137.0, 133.9, 132.2, 131.7, 127.3, 60.4; ^{19}F NMR (471 MHz, acetone-d_6) δ −79.0; HRMS (ESI): Found (M−OTf)$^+$ 243.0464, $C_{14}H_{11}O_2S$ requires 243.0474.
The quaternary carbon corresponding to the CF_3 in the triflate counter anion was not observed, though its presence was confirmed by ^{19}F NMR.

Methoxydiphenylsulfonium triflate (1a$_4$)

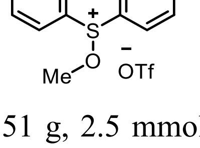

Following **GP1**, diphenyl sulfoxide (0.51 g, 2.5 mmol, 1.0 equiv.) and methanol (0.15 mL, 3.75 mmol, 1.5 equiv.) gave **1a$_4$** (0.78 g, 85%) as an oil. ^{1}H NMR (400 MHz, acetone-d_6) δ 8.16–8.09 (4H, m), 8.01–7.92 (2H, m), 7.91–7.82 (4H, m), 4.46 (3H, s); ^{13}C NMR (101 MHz, acetone-d_6) δ 136.9, 131.9, 130.7, 130.3, 121.6 (q, $J_{C\text{-}F}$ = 319.8 Hz), 65.2; ^{19}F NMR (376 MHz, acetone-d_6) δ −79.2; HRMS (ESI): Found (M−OTf)$^+$ 217.0682, $C_{13}H_{13}OS$ requires 217.0682.

5-(Methoxy-d_3)-5*H*-dibenzo[*b*,*d*]thiophen-5-ium triflate (1b)

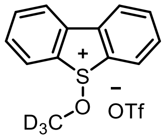

Following **GP1**, dibenzo[*b*,*d*]thiophene 5-oxide (0.50 g, 2.5 mmol, 1.0 equiv.) and methanol-d_4 (0.15 mL, 3.75 mmol, 1.5 equiv.) gave **1b** (0.75 g, 82%) as a solid. ^{1}H NMR (500 MHz, acetone-d_6) δ 8.59 (2H, d, J = 7.9 Hz), 8.38 (2H, d, J = 7.9 Hz), 8.08 (2H, td, J = 7.7, 1.1 Hz), 7.88 (2H, td, J = 7.7, 1.1 Hz); ^{13}C NMR (126 MHz, acetone-d_6) δ 142.0, 138.4, 132.8, 131.7, 130.4, 125.2; ^{19}F NMR (471 MHz, acetone-d_6) δ −78.9; ^{2}H NMR (61 MHz, acetone-d_6) δ 2.1; HRMS (ESI): Found (M−OTf)$^+$ 218.0719, $C_{13}H_8D_3OS$ requires 218.0713.
The quaternary carbon corresponding to the CF_3 in the triflate counter anion was not observed, though its presence was confirmed by ^{19}F NMR.

5-Ethoxy-5*H*-dibenzo[*b*,*d*]thiophen-5-ium triflate (1c)

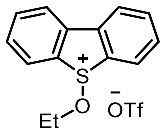

Following **GP1**, dibenzo[*b*,*d*]thiophene 5-oxide (0.20 g, 1.0 mmol, 1.0 equiv.) and ethanol (88 μL, 1.5 mmol, 1.5 equiv.) gave **1c** (0.36 g, 95%) as a solid. ^{1}H NMR (400 MHz, acetone-d_6) δ 8.58 (2H, d, J = 8.2 Hz), 8.35 (2H, d, J = 7.8 Hz), 8.03 (2H, td, J = 7.6, 1.1 Hz), 7.84 (2H, td, J = 7.7, 1.1 Hz), 4.11 (2H, q, J = 7.0 Hz), 1.30 (3H, t, J = 7.0 Hz); ^{13}C NMR (101 MHz, acetone-d_6) δ 141.4, 138.1, 132.6, 131.5, 131.4, 125.2, 122.1 (q, $J_{C\text{-}F}$ = 321.4 Hz), 71.4, 15.4; ^{19}F NMR (376 MHz, acetone-d_6) δ −78.8; HRMS (ESI): Found (M−OTf)$^+$ 229.0672, $C_{14}H_{13}OS$ requires 229.0682.

5-(2,2,2-Trifluoroethoxy)-5*H*-dibenzo[*b*,*d*]thiophen-5-ium triflate (1d)

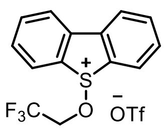

Following **GP1**, dibenzo[*b*,*d*]thiophene 5-oxide (1.00 g, 5.0 mmol, 1.0 equiv.) and 2,2,2-trifluoroethanol (0.55 mL, 7.5 mmol, 1.5 equiv.) gave **1d** (1.94 g, 90%) as a solid. ^{1}H NMR (500 MHz, acetone-d_6) δ 8.66 (2H, d, J = 7.9 Hz), 8.36 (2H, d, J = 7.8 Hz), 8.07 (2H, td, J = 7.7, 1.1 Hz), 7.86 (2H, td, J = 7.8, 1.1 Hz), 4.97 (2H, q, J = 8.1 Hz); ^{13}C NMR (126 MHz, acetone-d_6) δ 141.6, 138.9, 132.9, 132.2, 132.0, 125.5, 123.2 (q, $J_{C\text{-}F}$ = 277.2 Hz), 68.6 (q, $J_{C\text{-}F}$

= 37.2 Hz); ^{19}F NMR (471 MHz, acetone-d_6) δ −74.0, −78.9; HRMS (ESI): Found (M−OTf)$^+$ 283.0398, $C_{14}H_{10}F_3OS$ requires 283.0399.

The quaternary carbon corresponding to the CF_3 in the triflate counter anion was not observed, though its presence was confirmed by ^{19}F NMR.

5-(2,2,2-Trichloroethoxy)-5*H*-dibenzo[*b*,*d*]thiophen-5-ium triflate (1e)

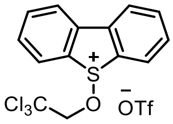

Following **GP1**, dibenzo[*b*,*d*]thiophene 5-oxide (0.20 g, 1.0 mmol, 1.0 equiv.) and 2,2,2-trichloroethanol (0.14 mL, 1.5 mmol, 1.5 equiv.) gave **1e** (0.48 g, 98%) as a solid. ^{1}H NMR (400 MHz, acetone-d_6) δ 8.71 (2H, d, J = 8.0 Hz), 8.37 (2H, d, J = 7.8 Hz), 8.08 (2H, td, J = 7.7, 1.1 Hz), 7.87 (2H, td, J = 7.8, 1.1 Hz), 5.12 (2H, s); ^{13}C NMR (101 MHz, acetone-d_6) δ 141.5, 138.8, 132.8, 132.4, 132.1, 125.5, 94.0, 81.4; ^{19}F NMR (376 MHz, acetone-d_6) δ −78.9; HRMS (ESI): Found (M−OTf)$^+$ 330.9511, $C_{14}H_{10}Cl_3OS$ requires 330.9512.

The quaternary carbon corresponding to the CF_3 in the triflate counter anion was not observed, though its presence was confirmed by ^{19}F NMR.

5-Isobutoxy-5*H*-dibenzo[*b*,*d*]thiophen-5-ium triflate (1f)

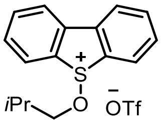

Following **GP1**, dibenzo[*b*,*d*]thiophene 5-oxide (1.00 g, 5.0 mmol, 1.0 equiv.) and 2-methyl-1-propanol (0.69 mL, 7.5 mmol, 1.5 equiv.) gave **1f** (1.88 g, 93%) as a solid. ^{1}H NMR (500 MHz, acetone-d_6) δ 8.61 (2H, d, J = 7.9 Hz), 8.37 (2H, d, J = 7.8 Hz), 8.07 (2H, td, J = 7.6, 1.5 Hz), 7.87 (2H, td, J = 7.7, 1.5 Hz), 3.75 (2H, d, J = 6.3 Hz), 2.02–1.91 (1H, m), 0.85 (6H, d, J = 6.8 Hz); ^{13}C NMR (126 MHz, acetone-d_6) δ 141.7, 138.3, 132.7, 131.6, 131.1 (d, J = 4.4 Hz), 125.2, 79.3, 29.2, 18.6; ^{19}F NMR (471 MHz, acetone-d_6) δ −78.9; HRMS (ESI): Found (M−OTf)$^+$ 257.0998, $C_{16}H_{17}OS$ requires 257.0995.

The quaternary carbon corresponding to the CF_3 in the triflate counter anion was not observed, though its presence was confirmed by ^{19}F NMR.

5-(Cyclohexylmethoxy)-5*H*-dibenzo[*b*,*d*]thiophen-5-ium triflate (1g)

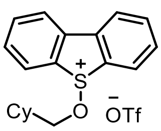

Following **GP1**, dibenzo[*b*,*d*]thiophene 5-oxide (0.50 g, 2.5 mmol, 1.0 equiv.) and cyclohexanemethanol (0.46 mL, 3.75 mmol, 1.5 equiv.) gave **1g** (0.90 g, 81%) as a solid. ^{1}H NMR (400 MHz, acetone-d_6) δ 8.60 (2H, d, J = 7.9 Hz), 8.37 (2H, d, J = 7.7 Hz), 8.06 (2H, td, J = 7.7, 1.1 Hz), 7.87 (2H, td, J = 7.7, 1.1 Hz), 3.76 (2H, d, J = 6.1 Hz), 1.75–1.53 (6H, m), 1.24–1.02 (3H, m), 0.96–0.82 (2H, m); ^{13}C NMR (101 MHz, acetone-d_6) δ 141.7, 138.3, 132.7, 131.5, 131.1, 125.2, 78.4, 38.4, 29.4, 26.6, 26.0; ^{19}F NMR (376 MHz, acetone-d_6) δ −78.9; HRMS (ESI): Found (M−OTf)$^+$ 297.1302, $C_{19}H_{21}OS$ requires 297.1308.
The quaternary carbon corresponding to the CF_3 in the triflate counter anion was not observed, though its presence was confirmed by ^{19}F NMR.

5-((Adamantan-1-yl)methoxy)-5*H*-dibenzo[*b*,*d*]thiophen-5-ium triflate (1h)

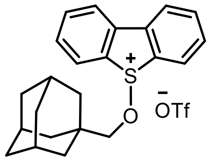

Following **GP1**, dibenzo[*b*,*d*]thiophene 5-oxide (0.50 g, 2.5 mmol, 1.0 equiv.) and 1-adamantanemethanol (0.62 g, 3.75 mmol, 1.5 equiv.) gave **1h** (1.10 g, 88%) as a solid. ^{1}H NMR (400 MHz, acetone-d_6) δ 8.61 (2H, d, J = 7.9 Hz), 8.37 (2H, d, J = 7.8 Hz), 8.07 (2H, td, J = 7.7, 1.1 Hz), 7.87 (2H, td, J = 7.7, 1.1 Hz), 3.47 (2H, s), 1.94–1.87 (3H, m), 1.73–1.64 (3H, m), 1.62–1.54 (3H, m), 1.49–1.44 (6H, m); ^{13}C NMR (101 MHz, acetone-d_6) δ 141.8, 138.3, 132.6, 131.6, 130.9, 125.2, 81.8, 38.9, 37.2, 34.8, 28.6; ^{19}F NMR (376 MHz, acetone-d_6) δ −78.8; HRMS (ESI): Found (M−OTf)$^+$ 349.1611, $C_{23}H_{25}OS$ requires 349.1621.
The quaternary carbon corresponding to the CF_3 in the triflate counter anion was not observed, though its presence was confirmed by ^{19}F NMR.

5-(3-Chloropropoxy)-5*H*-dibenzo[*b*,*d*]thiophen-5-ium triflate (1i)

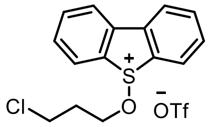

Following **GP1**, dibenzo[*b*,*d*]thiophene 5-oxide (0.10 g, 0.5 mmol, 1.0 equiv.) and 3-chloropropan-1-ol (62 μL, 0.75 mmol, 1.5 equiv.) gave **1i** (0.20 g, 94%) as a solid. ^{1}H NMR (500 MHz, acetone-d_6) δ 8.62 (2H, d, J = 7.9 Hz), 8.37 (2H, d, J = 7.7 Hz), 8.05 (2H, td, J =

7.7, 1.1 Hz), 7.85 (2H, td, J = 7.9, 1.1 Hz), 4.07 (2H, t, J = 5.7 Hz), 3.66 (2H, t, J = 6.2 Hz), 2.14 (2H, pent, J = 6.0 Hz); ^{13}C NMR (101 MHz, acetone-d_6) δ 141.7, 138.3, 132.7, 131.7, 130.9, 125.2, 70.4, 41.0, 32.5; ^{19}F NMR (376 MHz, acetone-d_6) δ −79.0; IR (neat, cm^{-1}): 3089, 1713, 1583, 1462, 1448, 1422, 1290, 1255, 1221, 1156; HRMS (ESI): Found (M−OTf)$^+$ 277.0448, $C_{15}H_{14}OCl$ requires 277.0448.
The quaternary carbon corresponding to the CF_3 in the triflate counter anion was not observed, though its presence was confirmed by ^{19}F NMR.

5-(3-Bromopropoxy)-5*H*-dibenzo[*b*,*d*]thiophen-5-ium triflate (1j)

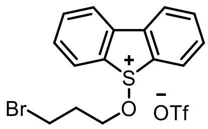

Following **GP1**, dibenzo[*b*,*d*]thiophene 5-oxide (0.20 g, 1.0 mmol, 1.0 equiv.) and 3-bromopropan-1-ol (0.13 mL, 1.5 mmol, 1.5 equiv.) gave **1j** (0.39 g, 83%) as a solid. ^{1}H NMR (500 MHz, acetone-d_6) δ 8.64 (2H, d, J = 7.9 Hz), 8.38 (2H, d, J = 7.7 Hz), 8.07 (2H, t, J = 7.7 Hz), 7.87 (2H, t, J = 7.7 Hz), 4.07 (2H, t, J = 5.7 Hz), 3.53 (2H, td, J = 6.4 Hz), 2.22 (1H, pent, J = 6.1 Hz); ^{13}C NMR (126 MHz, acetone-d_6) δ 141.7, 138.4, 132.8, 131.8, 131.0, 125.2, 71.3, 32.6, 29.4; ^{19}F NMR (471 MHz, acetone-d_6) δ −78.9; IR (neat, cm^{-1}): 3087, 3010, 2947, 1685, 1579, 1482, 1447, 1415, 1271, 1247, 1223, 1148; HRMS (ESI): Found (M−OTf)$^+$ 320.9928, $C_{15}H_{14}OBrS$ requires 320.9930.
The quaternary carbon corresponding to the CF_3 in the triflate counter anion was not observed, though its presence was confirmed by ^{19}F NMR.

5-(2-Cyanoethoxy)-5*H*-dibenzo[*b*,*d*]thiophen-5-ium triflate (1k)

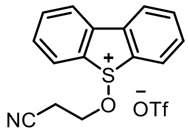

Following **GP1**, dibenzo[*b*,*d*]thiophene 5-oxide (0.20 g, 1.0 mmol, 1.0 equiv.) and 3-hydroxypropionitrile (0.10 mL, 1.5 mmol, 1.5 equiv.) gave **1k** (0.40 g, 99%) as a solid. ^{1}H NMR (400 MHz, acetone-d_6) δ 8.65 (2H, d, J = 7.9 Hz), 8.37 (2H, d, J = 7.8 Hz), 8.06 (2H, td, J = 7.7, 1.1 Hz), 7.86 (2H, td, J = 7.8, 1.1 Hz), 4.37 (2H, t, J = 5.7 Hz), 3.03 (2H, t, J = 5.7 Hz); ^{13}C NMR (101 MHz, acetone-d_6) δ 141.6, 138.5, 132.8, 131.8, 131.5, 125.3, 117.3, 69.3, 19.2; ^{19}F NMR (376 MHz, acetone-d_6) δ −78.9; HRMS (ESI): Found (M−OTf)$^+$ 254.0627, $C_{15}H_{12}NOS$ requires 254.0634.

The quaternary carbon corresponding to the CF_3 in the triflate counter anion was not observed, though its presence was confirmed by ^{19}F NMR.

5-(2-(Phenylsulfonyl)ethoxy)-5*H*-dibenzo[*b*,*d*]thiophen-5-ium triflate (1l)

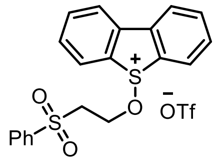

Following **GP1**, dibenzo[*b*,*d*]thiophene 5-oxide (0.10 g, 0.5 mmol, 1.0 equiv.) and 2-(phenylsulfonyl)ethan-1-ol (87 µL, 0.75 mmol, 1.5 equiv.) gave **1l** (0.29 g, 56%) as a solid. ^{1}H NMR (400 MHz, acetone-*d6*) δ 8.55 (2H, d, *J* = 7.9 Hz), 8.31 (2H, d, *J* = 7.9 Hz), 8.04 (2H, td, *J* = 7.7, 1.1 Hz), 7.86–7.79 (4H, m), 7.71–7.63 (1H, m), 7.56 (2H, t, *J* = 7.8 Hz), 4.55 (2H, t, *J* = 5.4 Hz), 3.80 (2H, t, *J* = 5.4 Hz); ^{13}C NMR (101 MHz, acetone-*d6*) δ 141.3, 140.3, 138.4, 135.0, 132.7, 131.8, 131.7, 130.3, 128.7, 125.2, 68.4, 55.8; ^{19}F NMR (376 MHz, acetone-*d6*) δ −79.0; IR (neat, cm^{-1}): 3095, 2929, 1447, 1294, 1271, 1255, 1223, 1188, 1161, 1141; HRMS (ESI): Found (M−OTf)$^+$ 369.0599, $C_{20}H_{17}O_3S_2$ requires 369.0614.

The quaternary carbon corresponding to the CF_3 in the triflate counter anion was not observed, though its presence was confirmed by ^{19}F NMR.

5-(Allyloxy)-5*H*-dibenzo[*b*,*d*]thiophen-5-ium triflate (1m)

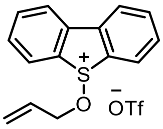

Following **GP1**, dibenzo[*b*,*d*]thiophene 5-oxide (0.20 g, 1.0 mmol, 1.0 equiv.) and prop-2-en-1-ol (0.10 mL, 1.5 mmol, 1.5 equiv.) gave **1m** (0.32 g, 82%) as a solid. ^{1}H NMR (400 MHz, acetone-*d6*) δ 8.59 (2H, d, *J* = 7.8 Hz), 8.36 (2H, d, *J* = 7.8 Hz), 8.06 (2H, td, *J* = 7.7, 1.1 Hz), 7.85 (2H, dq, *J* = 7.8, 1.1 Hz), 5.96 (1H, ddt, *J* = 16.9, 10.3, 6.4 Hz), 5.45 (1H, dq, *J* = 16.9, 1.3 Hz), 5.40 (1H, dd, *J* = 10.3, 1.0 Hz), 4.64 (2H, dt, *J* = 6.4, 1.1 Hz); ^{13}C NMR (101 MHz, acetone-*d6*) δ 141.6, 138.3, 132.7, 131.9, 131.7, 131.3, 125.3, 124.0, 75.3; ^{19}F NMR (376 MHz, acetone-*d6*) δ −78.8; IR (neat, cm^{-1}): 3090, 1583, 1448, 1425, 1250, 1221, 1144; HRMS (ESI): Found (M−OTf)$^+$ 241.0666, $C_{15}H_{13}OS$ requires 214.0682.

The quaternary carbon corresponding to the CF_3 in the triflate counter anion was not observed, though its presence was confirmed by ^{19}F NMR.

5-(But-3-yn-1-yloxy)-5*H*-dibenzo[*b*,*d*]thiophen-5-ium triflate (1n)

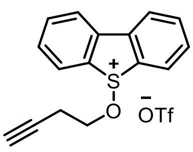

Following **GP1**, dibenzo[*b*,*d*]thiophene 5-oxide (0.20 g, 1.0 mmol, 1.0 equiv.) and but-3-yn-1-ol (92 μL, 1.5 mmol, 1.5 equiv.) gave **1n** (0.37 g, 91%) as a solid. ^{1}H NMR (500 MHz, acetone-d_6) δ 8.62 (2H, d, J = 7.9 Hz), 8.37 (2H, d, J = 7.2 Hz), 8.07 (2H, td, J = 7.7, 1.1 Hz), 7.97 (2H, td, J = 7.7, 1.1 Hz), 4.17 (2H, d, J = 6.0 Hz), 2.66 (2H, td, J = 6.0, 2.6 Hz), 2.56 (1H, t, J = 2.6 Hz); ^{13}C NMR (126 MHz, acetone-d_6) δ 141.6, 138.4, 132.7, 131.8, 131.5, 125.2, 79.7, 72.7, 72.5, 20.2; ^{19}F NMR (471 MHz, acetone-d_6) δ −78.9; IR (neat, cm^{-1}): 3092, 1582, 1451, 1432, 1260, 1221, 1148; HRMS (ESI): Found (M−OTf)$^+$ 253.0667, $C_{16}H_{13}OS$ requires 253.0682. The quaternary carbon corresponding to the CF_3 in the triflate counter anion was not observed, though its presence was confirmed by ^{19}F NMR.

5-((4-Chlorobut-2-yn-1-yl)oxy)-5*H*-dibenzo[*b*,*d*]thiophen-5-ium triflate (1o)

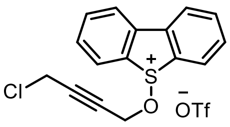

Following **GP1**, dibenzo[*b*,*d*]thiophene 5-oxide (0.20 g, 1.0 mmol, 1.0 equiv.) and 4-chlorobut-2-yn-1-ol (0.13 mL, 1.5 mmol, 1.5 equiv.) gave **1o** (0.40 g, 91%) as an oil. ^{1}H NMR (400 MHz, acetone-d_6) δ 8.60 (2H, d, J = 8.0 Hz), 8.33 (2H, d, J = 7.8 Hz), 8.04 (2H, td, J = 7.7, 1.1 Hz), 7.83 (2H, dd, J = 7.7, 1.2 Hz), 5.13 (2H, t, J = 2.0 Hz), 4.42 (2H, t, J = 2.0 Hz); ^{13}C NMR (101 MHz, acetone-d_6) δ 141.3, 138.5, 132.6, 132.0, 131.9, 125.3, 121.9 (q, $J_{C\text{-}F}$ = 310.7 Hz), 88.8, 79.2, 63.0, 30.4; ^{19}F NMR (376 MHz, acetone-d_6) δ −79.0; HRMS (ESI): Found (M−OTf)$^+$ 287.0288, $C_{16}H_{12}OClS$ requires 287.0292.

5-(2-(1,3-Dioxoisoindolin-2-yl)ethoxy)-5*H*-dibenzo[*b*,*d*]thiophen-5-ium triflate (1p)

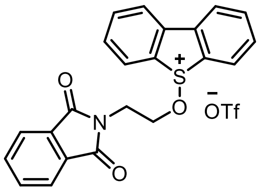

Following **GP1**, dibenzo[*b*,*d*]thiophene 5-oxide (0.10 g, 0.5 mmol, 1.0 equiv.) and 2-(2-hydroxyethyl)isoindoline-1,3-dione (0.14 g, 0.75 mmol, 1.5 equiv.) gave **1p** (0.22 g, 85%) as a solid. ^{1}H NMR (500 MHz, acetone-d_6) δ 8.46 (2H, d, J = 8.0 Hz), 8.34 (2H, d, J = 7.8 Hz), 8.02 (2H, td, J = 7.7, 1.1 Hz), 7.96–7.80 (4H, m), 7.70 (2H, td, J = 7.7, 1.2 Hz), 4.26 (2H, t, J

= 5.1 Hz), 3.97 (2H, t, J = 5.1 Hz); ^{13}C NMR (101 MHz, acetone-d_6) δ 168.3, 141.6, 138.4, 135.4, 132.8, 132.7, 131.5, 131.3, 125.3, 124.1, 70.6, 37.8; ^{19}F NMR (376 MHz, acetone-d_6) δ −78.9; IR (neat, cm^{-1}): 3095, 1772, 1709, 1581, 1449, 1427, 1392, 1352, 1295, 1256, 1223, 1190, 1145; HRMS (ESI): Found (M–OTf)$^+$ 374.0840, $C_{22}H_{16}O_3NS$ requires 374.0850.
The quaternary carbon corresponding to the CF_3 in the triflate counter anion was not observed, though its presence was confirmed by ^{19}F NMR.

5-(3-Hydroxy-3-methylbutoxy)-5*H*-dibenzo[*b*,*d*]thiophen-5-ium triflate (1q)

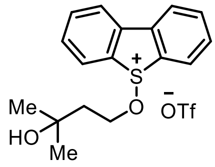

Following **GP1**, dibenzo[*b*,*d*]thiophene 5-oxide (0.20 g, 1.0 mmol, 1.0 equiv.) and 3-methyl-1,3-butanediol (0.16 mL, 1.5 mmol, 1.5 equiv.) gave **1q** (0.42 g, 96%) as an oil. ^{1}H NMR (400 MHz, acetone-d_6) δ 8.56 (2H, d, J = 8.2 Hz), 8.30 (2H, d, J = 7.8 Hz), 8.00 (2H, td, J = 7.7, 1.1 Hz), 7.81 (3H, td, J = 7.7, 1.1 Hz), 4.22 (2H, t, J = 6.6 Hz), 1.90 (2H, t, J = 6.7 Hz), 1.13 (6H, s); ^{13}C NMR (101 MHz, acetone-d_6) δ 141.3, 138.1, 132.5, 131.5, 131.4, 125.1, 121.5 (q, $J_{C\text{-}F}$ = 319.8 Hz), 72.4, 69.9, 42.7, 29.6; ^{19}F NMR (376 MHz, acetone-d_6) δ −79.1; HRMS (ESI): Found (M−OTf)$^+$ 287.1114, $C_{17}H_{19}O_2S$ requires 287.1100.

5-(2-(Acryloyloxy)ethoxy)-5*H*-dibenzo[*b*,*d*]thiophen-5-ium triflate (1r)

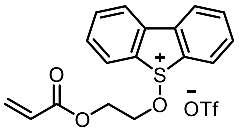

Following **GP1**, dibenzo[*b*,*d*]thiophene 5-oxide (0.20 g, 1.0 mmol, 1.0 equiv.) and 2-hydroxyethyl acrylate (0.13 mL, 1.5 mmol, 1.2 equiv.) gave **1r** (0.42 g, 94%) as an oil. ^{1}H NMR (400 MHz, acetone-d_6) δ 8.61 (2H, d, J = 7.9 Hz), 8.36 (2H, d, J = 7.8 Hz), 8.06 (2H, td, J = 7.7, 1.1 Hz), 7.86 (2H, td, J = 7.7, 1.2 Hz), 6.36 (1H, dd, J = 17.3, 1.5 Hz), 6.15 (1H, dd, J = 17.3, 10.4 Hz), 5.96 (1H, dd, J = 10.4, 1.5 Hz), 4.43–4.37 (2H, m), 4.37–4.33 (2H, m); ^{13}C NMR (101 MHz, acetone-d_6) δ 165.8, 141.6, 138.4, 132.8, 132.4, 131.7, 131.6, 128.7, 125.3, 72.4, 62.7; ^{19}F NMR (376 MHz, acetone-d_6) δ −79.0; HRMS (ESI): Found (M−OTf)$^+$ 299.0730, $C_{17}H_{15}O_3S$ requires 299.0736.
The quaternary carbon corresponding to the CF_3 in the triflate counter anion was not observed, though its presence was confirmed by ^{19}F NMR.

(*S*)-5-(2-(1,3-Dioxoisoindolin-2-yl)-3-methoxy-3-oxopropoxy)-5*H*-dibenzo[*b*,*d*]thiophen-5-ium triflate (1s)

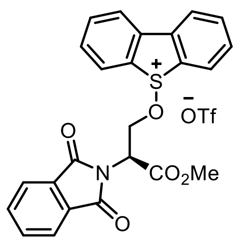

Following **GP1**, dibenzo[*b*,*d*]thiophene 5-oxide (0.10 g, 0.5 mmol, 1.0 equiv.) and methyl (*S*)-2-(1,3-dioxoisoindolin-2-yl)-3-hydroxypropanoate (0.19 g, 0.75 mmol, 1.5 equiv.) gave **1s** (0.28 g, 97%) as a solid. ^{1}H NMR (400 MHz, acetone-d_6) δ 8.59 (1H, d, J = 7.9 Hz), 8.40 (1H, d, J = 7.9 Hz), 8.36 (1H, d, J = 8.4 Hz), 8.34 (1H, d, J = 8.4 Hz), 8.09–8.01 (2H, m), 8.01–7.91 (4H, m), 7.84 (1H, td, J = 7.7, 1.2 Hz), 7.67 (1H, td, J = 7.7, 1.2 Hz), 5.41 (1H, dd, J = 8.8, 4.6 Hz), 4.70 (1H, dd, J = 10.1, 4.6 Hz), 4.52 (1H, dd, J = 10.1, 8.8 Hz), 3.66 (3H, s); ^{13}C NMR (101 MHz, acetone-d_6) δ 167.5, 167.1, 141.7, 141.5, 138.6 (2 × C), 136.0, 132.8 (2 × C), 132.4, 131.9, 131.7, 131.2 (2 × C), 125.4, 125.2, 124.6, 69.4, 53.6, 51.2; ^{19}F NMR (376 MHz, acetone-d_6) δ −79.0; IR (neat, cm^{-1}): 3091, 1779, 1755, 1712, 1465, 1449, 1437, 1387, 1273, 1256, 1221, 1163, 1142, 1106; HRMS (ESI): Found (M–OTf)$^+$ 432.0912, $C_{24}H_{18}NO_5S$ requires 432.0900.

The quaternary carbon corresponding to the CF_3 in the triflate counter anion was not observed, though its presence was confirmed by ^{19}F NMR.

5-(2-(1,3-Dioxoisoindolin-2-yl)-3-methoxy-3-oxopropoxy)-5*H*-dibenzo[*b*,*d*]thiophen-5-ium triflate (1s')

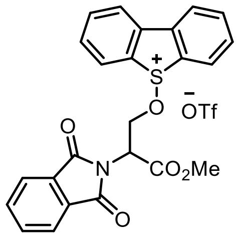

Following **GP1**, dibenzo[*b*,*d*]thiophene 5-oxide (0.20 g, 1.0 mmol, 1.0 equiv.) and methyl 2-(1,3-dioxoisoindolin-2-yl)-3-hydroxypropanoate (0.30 g, 1.2 mmol, 1.2 equiv.) gave **1s'** (0.55 g, 95%) as a solid. ^{1}H NMR (500 MHz, acetone-d_6) δ 8.60 (1H, d, J = 7.9 Hz), 8.40 (1H, d, J = 7.9 Hz), 8.36 (2H, t, J = 7.8 Hz), 8.07 (1H, td, J = 7.2, 1.1 Hz), 8.04 (1H, td, J = 7.2, 1.1 Hz), 8.00–7.92 (4H, m), 7.85 (1H, td, J = 7.7, 1.2 Hz), 7.68 (1H, td, J = 7.7, 1.2 Hz), 5.41 (1H, dd, J = 8.8, 4.6 Hz), 4.69 (1H, dd, J = 10.2, 4.6 Hz), 4.51 (1H, dd, J = 10.2, 8.8 Hz), 3.66 (3H, s); ^{13}C NMR (126 MHz, acetone-d_6) δ 167.5, 167.1, 141.7, 141.5, 138.6 (2 × C), 136.0, 132.8 (2

× C), 132.4, 131.9, 131.7, 131.2 (2 × C), 125.4, 125.2, 124.6, 69.4, 53.6, 51.2; ^{19}F NMR (471 MHz, acetone-*d₆*) δ −78.9; IR (neat, cm^{-1}): 3091, 1779, 1755, 1712, 1465, 1449, 1437, 1387, 1273, 1256, 1221, 1163, 1142, 1106; HRMS (ESI): Found (M−OTf)$^+$ 432.0910, $C_{24}H_{18}NO_5S$ requires 432.0900.

The quaternary carbon corresponding to the CF_3 in the triflate counter anion was not observed, though its presence was confirmed by ^{19}F NMR.

5-(((3a*S*,5a*R*,8a*R*,8b*S*)-2,2,7,7-Tetramethyltetrahydro-3a*H*-bis([1,3]dioxolo)[4,5-*b*:4',5'-*d*]pyran-3a-yl)methoxy)-5*H*-dibenzo[*b*,*d*]thiophen-5-ium triflate (1t)

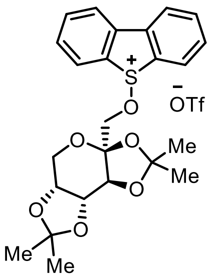

Following **GP1**, dibenzo[*b*,*d*]thiophene 5-oxide (0.10 g, 0.5 mmol, 1.0 equiv.) and ((3a*S*,5a*R*,8a*R*,8b*S*)-2,2,7,7-tetramethyltetrahydro-3a*H*-bis([1,3]dioxolo)[4,5-*b*:4',5'-*d*]pyran-3a-yl)methanol (0.19 g, 0.75 mmol, 1.5 equiv.) gave **1t** (0.26 g, 89%) as a solid. ^{1}H NMR (400 MHz, acetone-*d₆*) δ 8.60 (2H, d, *J* = 7.9 Hz), 8.34 (2H, d, *J* = 7.8 Hz), 8.05 (2H, td, *J* = 7.7, 4.5 Hz), 7.85 (2H, td, *J* = 7.7, 4.6 Hz), 4.60 (1H, dd, *J* = 8.0, 2.6 Hz), 4.28 (2H, dd, *J* = 15.6, 2.8 Hz), 4.24 (1H, s), 4.19 (1H, d, *J* = 10.8 Hz), 3.88 (1H, dd, *J* = 13.0, 1.9 Hz), 3.64 (1H, d, *J* = 13.0 Hz), 1.52 (3H, s), 1.39 (3H, s), 1.25 (3H, s), 1.16 (3H, s); ^{13}C NMR (101 MHz, acetone-*d₆*) δ 141.4, 141.2, 138.4 (2 × C), 132.7 (2 × C), 132.2, 132.1, 132.0, 131.7, 125.2, 125.1, 110.4, 109.4, 101.4, 75.2, 71.3, 71.2, 70.4, 62.1, 26.5, 26.1, 25.3, 24.2; ^{19}F NMR (376 MHz, acetone-*d₆*) δ −79.0; IR (neat, cm^{-1}): 2991, 2940, 1710, 1449, 1388, 1296, 1164; HRMS (ESI): Found (M−OTf)$^+$ 433.1523, $C_{24}H_{27}O_6S$ requires 443.1506.

The quaternary carbon corresponding to the CF_3 in the triflate counter anion was not observed, though its presence was confirmed by ^{19}F NMR.

5-(But-3-yn-2-yloxy)-5*H*-dibenzo[*b*,*d*]thiophen-5-ium triflate (1u)

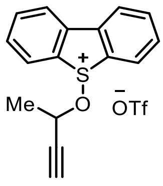

Following **GP1**, dibenzo[*b*,*d*]thiophene 5-oxide (0.20 g, 1.0 mmol, 1.0 equiv.) and 3-butyn-2-ol (0.12 mL, 1.5 mmol, 1.5 equiv.) gave **1u** (0.37 g, 92%) as a solid. ^{1}H NMR (400 MHz, acetone-d_6) δ 8.62 (1H, d, J = 7.9 Hz), 8.53 (1H, d, J = 8.1 Hz), 8.36–8.31 (2H, m), 8.06–8.01 (2H, m), 7.86–7.79 (2H, m), 5.67 (1H, qd, J = 6.5, 2.1 Hz), 3.85 (1H, d, J = 2.1 Hz), 1.67 (3H, d, J = 6.5 Hz); ^{13}C NMR (101 MHz, acetone-d_6) δ 141.1, 140.9, 138.2 (2 × C), 133.2, 132.9, 132.6, 132.5, 131.9, 131.5, 125.3, 125.2, 82.8, 81.0, 74.9, 23.2; ^{19}F NMR (376 MHz, $CDCl_3$) δ −78.9; HRMS (ESI): Found $(M{-}OTf)^+$ 253.0686, $C_{16}H_{13}OS$ requires 253.0682.
The quaternary carbon corresponding to the CF_3 in the triflate counter anion was not observed, though its presence was confirmed by ^{19}F NMR.

5-Isopropoxy-5*H*-dibenzo[*b*,*d*]thiophen-5-ium triflate (1v)

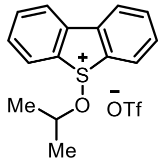

Following **GP1**, dibenzo[*b*,*d*]thiophene 5-oxide (0.40 g, 2.0 mmol, 1.0 equiv.) and isopropanol (0.23 mL, 3.0 mmol, 1.5 equiv.) gave **1v** (0.74 g, 94%) as a solid. ^{1}H NMR (400 MHz, acetone-d_6) δ 8.61 (2H, d, J = 8.0 Hz), 8.36 (2H, d, J = 7.7 Hz), 8.05 (2H, td, J = 7.6, 1.1 Hz), 7.86 (2H, td, J = 7.8, 1.1 Hz), 4.95 (1H, hept, J = 6.2 Hz), 1.39 (6H, d, J = 6.2 Hz); ^{13}C NMR (101 MHz, acetone-d_6) δ 141.1, 138.1, 133.1, 132.6, 131.4, 125.3, 85.5, 23.7; ^{19}F NMR (376 MHz, $CDCl_3$) δ −78.8; HRMS (ESI): Found $(M{-}OTf)^+$ 243.0831, $C_{15}H_{15}OS$ requires 243.0838.
The quaternary carbon corresponding to the CF_3 in the triflate counter anion was not observed, though its presence was confirmed by ^{19}F NMR.

5-(*sec*-Butoxy)-5*H*-dibenzo[*b*,*d*]thiophen-5-ium triflate (1w)

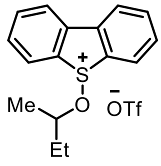

Following **GP1**, dibenzo[*b*,*d*]thiophene 5-oxide (0.20 g, 1.0 mmol, 1.0 equiv.) and 2-butanol (0.14 mL, 3.0 mmol, 1.5 equiv.) gave **1w** (0.39 g, 95%) as a solid. ^{1}H NMR (400 MHz, acetone-

d_6) δ 8.62 (1H, d, J = 8.1 Hz), 8.59 (1H, d, J = 7.9 Hz), 8.36 (2H, d, J = 7.7 Hz), 8.05 (2H, td, J = 7.6, 1.1 Hz), 7.85 (2H, td, J = 7.9, 1.1 Hz), 4.81 (1H, h, J = 6.2 Hz), 1.83–1.61 (2H, m), 1.38 (3H, d, J = 6.2 Hz), 0.87 (3H, t, J = 7.4 Hz); ^{13}C NMR (101 MHz, acetone-d_6) δ 141.1, 141.0, 138.1, 133.2 (2 × C), 132.6 (2 × C), 131.5 (2 × C), 125.3, 125.2, 89.8, 30.6, 21.1, 9.4; ^{19}F NMR (376 MHz, $CDCl_3$) δ −78.9; HRMS (ESI): Found (M−OTf)$^+$ 257.0986, $C_{16}H_{17}OS$ requires 257.0995.

The quaternary carbon corresponding to the CF_3 in the triflate counter anion was not observed, though its presence was confirmed by ^{19}F NMR.

5-((1-Methoxy-1-oxopropan-2-yl)oxy)-5*H*-dibenzo[*b*,*d*]thiophen-5-ium triflate (1x)

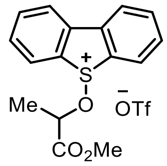

Following **GP1**, dibenzo[*b*,*d*]thiophene 5-oxide (0.20 g, 1.0 mmol, 1.0 equiv.) and methyl lactate (0.14 mL, 1.5 mmol, 1.5 equiv.) gave **1x** (0.39 g, 89%) as a solid. ^{1}H NMR (500 MHz, acetone-d_6) δ 8.63 (1H, d, J = 8.0 Hz), 8.63 (1H, d, J = 8.0 Hz), 8.34–8.27 (2H, m), 8.03 (1H, td, J = 7.6, 1.1 Hz), 8.00 (1H, td, J = 7.6, 1.1 Hz), 7.84 (1H, td, J = 7.7, 1.1 Hz), 7.80 (1H, td, J = 7.7, 1.1 Hz), 5.84 (1H, q, J = 7.0 Hz), 3.96 (3H, s), 1.70 (3H, d, J = 7.0 Hz); ^{13}C NMR (101 MHz, acetone-d_6) δ 172.1, 140.8, 140.4, 138.2, 138.0, 134.6, 134.4, 132.6, 132.5, 131.8, 131.5, 125.1 (2 × C), 122.1 (q, $J_{C\text{-}F}$ = 321.3 Hz), 80.7, 54.2, 18.7; ^{19}F NMR (471 MHz, $CDCl_3$) δ −78.9; HRMS (ESI): Found (M−OTf)$^+$ 301.0887, $C_{17}H_{17}O_3S$ requires 301.0893.

5-((1,3-Dichloropropan-2-yl)oxy)-5*H*-dibenzo[*b*,*d*]thiophen-5-ium triflate (1y)

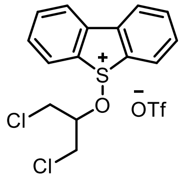

Following **GP1**, dibenzo[*b*,*d*]thiophene 5-oxide (0.20 g, 1.0 mmol, 1.0 equiv.) and 1,3-dichloro-2-propanol (0.14 mL, 1.5 mmol, 1.5 equiv.) gave **1y** (0.42 g, 90%) as a solid. ^{1}H NMR (400 MHz, acetone-d_6) δ 8.67 (2H, d, J = 7.9 Hz), 8.34 (2H, d, J = 7.8 Hz), 8.06 (2H, td, J = 7.7, 1.1 Hz), 7.85 (2H, td, J = 7.7, 1.1 Hz), 5.63 (1H, tt, J = 6.1, 3.9 Hz), 4.16 (2H, dd, J = 12.8, 3.9 Hz), 4.05 (2H, dd, J = 12.8, 6.2 Hz); ^{13}C NMR (101 MHz, acetone-d_6) δ 140.9, 138.5, 134.2, 132.7, 131.7, 125.4, 87.7, 44.6; ^{19}F NMR (376 MHz, $CDCl_3$) δ −78.9; HRMS (ESI): Found (M−OTf)$^+$ 311.0060, $C_{15}H_{13}OCl_2S$ requires 311.0059.

The quaternary carbon corresponding to the CF_3 in the triflate counter anion was not observed, though its presence was confirmed by ^{19}F NMR.

5-((1,3-Dibromopropan-2-yl)oxy)-5*H*-dibenzo[*b*,*d*]thiophen-5-ium triflate (1z)

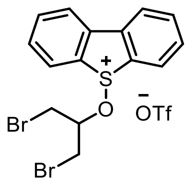

Following **GP1**, dibenzo[*b*,*d*]thiophene 5-oxide (0.20 g, 1.0 mmol, 1.0 equiv.) and 1,3-dibromo-2-propanol (0.33 g, 1.5 mmol, 1.5 equiv.) gave **1z** (0.49 g, 89%) as a solid. ^{1}H NMR (400 MHz, acetone-d_6) δ 8.70 (2H, d, J = 7.9 Hz), 8.33 (2H, d, J = 7.8 Hz), 8.04 (2H, t, J = 7.6 Hz), 7.84 (2H, t, J = 7.8 Hz), 5.62 (1H, tt, J = 5.8, 4.4 Hz), 4.02 (2H, dd, J = 12.0, 4.4 Hz), 3.94 (2H, dd, J = 12.0, 5.8 Hz); ^{13}C NMR (101 MHz, acetone-d_6) δ 140.8, 138.5, 134.2, 132.6, 131.7, 125.4, 86.4, 32.8; ^{19}F NMR (376 MHz, $CDCl_3$) δ −78.8; HRMS (ESI): Found $(M-OTf)^+$ 398.9051, $C_{15}H_{13}OBr_2S$ requires 398.9048.
The quaternary carbon corresponding to the CF_3 in the triflate counter anion was not observed, though its presence was confirmed by ^{19}F NMR.

(*S*)-5-((4-Methoxy-4-oxobutan-2-yl)oxy)-5*H*-dibenzo[*b*,*d*]thiophen-5-ium triflate (1aa)

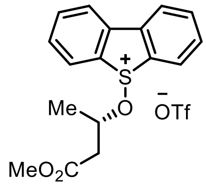

Following **GP1**, dibenzo[*b*,*d*]thiophene 5-oxide (0.20 g, 1.0 mmol, 1.0 equiv.) and Methyl (*S*)-(+)-3-hydroxybutyrate (0.17 mL, 1.5 mmol, 1.5 equiv.) gave **1aa** (0.45 g, 99%) as an oil. ^{1}H NMR (400 MHz, acetone-d_6) δ 8.61 (1H, d, J = 7.9 Hz), 8.45 (1H, d, J = 7.9 Hz), 8.31 (2H, dt, J = 7.8, 1.4 Hz), 8.00 (2H, tt, J = 7.6, 1.1 Hz), 7.85–7.79 (2H, m), 5.56 (1H, dqd, J = 8.3, 6.4, 3.7 Hz), 3.75 (3H, s), 2.99 (1H, dd, J = 17.9, 3.8 Hz), 2.91 (1H, dd, J = 17.9, 8.4 Hz), 1.56 (3H, d, J = 6.4 Hz); ^{13}C NMR (101 MHz, acetone-d_6) δ 172.0, 140.6, 140.3, 138.1, 137.9, 134.7, 133.9, 132.6, 132.5, 131.4, 131.1, 125.1 (2 × C), 121.9 (q, J_{C-F} = 320.8 Hz), 86.0, 52.5, 41.5, 21.8; ^{19}F NMR (376 MHz, $CDCl_3$) δ −79.0; HRMS (ESI): Found $(M-OTf)^+$ 301.0887, $C_{17}H_{17}O_3S$ requires 301.0893.
The quaternary carbon corresponding to the CF_3 in the triflate counter anion was not observed, though its presence was confirmed by ^{19}F NMR.

5-((4-Methoxy-4-oxobutan-2-yl)oxy)-5*H*-dibenzo[*b*,*d*]thiophen-5-ium triflate (1aa')

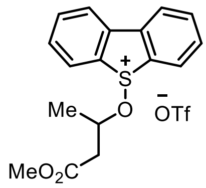

Following **GP1**, dibenzo[*b*,*d*]thiophene 5-oxide (0.20 g, 1.0 mmol, 1.0 equiv.) and methyl 3-hydroxybutyrate (0.18 g, 1.5 mmol, 1.5 equiv.) gave **1aa'** (0.43 g, 95%) as an oil. ^{1}H NMR (400 MHz, acetone-d_6) δ 8.60 (1H, d, J = 8.1 Hz), 8.44 (1H, d, J = 7.9 Hz), 8.29 (2H, d, J = 7.8 Hz), 7.99 (2H, t, J = 7.6 Hz), 7.83–7.78 (2H, m), 5.61–5.49 (1H, m), 3.75 (3H, s), 2.98 (1H, dd, J = 17.9, 3.7 Hz), 2.91 (1H, dd, J = 17.9, 8.5 Hz), 1.55 (3H, d, J = 6.4 Hz); ^{13}C NMR (101 MHz, acetone-d_6) δ 172.0, 140.5, 140.3, 138.1, 137.9, 134.6, 133.8, 132.5 (2 × C), 131.4, 131.1, 125.1 (2 × C), 121.8 (q, $J_{C\text{-}F}$ = 320.2 Hz), 86.0, 52.5, 41.5, 21.8; ^{19}F NMR (376 MHz, $CDCl_3$) δ −78.9; HRMS (ESI): Found (M−OTf)$^+$ 301.0887, $C_{17}H_{17}O_3S$ requires 301.0893.

The quaternary carbon corresponding to the CF_3 in the triflate counter anion was not observed, though its presence was confirmed by ^{19}F NMR.

(*S*)-5-((5-Oxotetrahydrofuran-3-yl)oxy)-5*H*-dibenzo[*b*,*d*]thiophen-5-ium triflate (1ab)

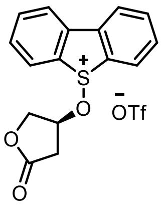

Following **GP1**, dibenzo[*b*,*d*]thiophene 5-oxide (0.10 g, 0.5 mmol, 1.0 equiv.) and (*S*)-4-hydroxydihydrofuran-2(3*H*)-one (61 µL, 0.75 mmol, 1.5 equiv.) gave **1ab** (0.22 g, 98%) as a solid. ^{1}H NMR (500 MHz, acetone-d_6) δ 8.68 (1H, d, J = 8.0 Hz), 8.57 (1H, d, J = 8.0 Hz), 8.33 (1H, d, J = 7.8 Hz), 8.32 (1H, d, J = 7.8 Hz), 8.06–8.02 (2H, m), 7.86–7.81 (2H, m), 6.04 (1H, dd, J = 9.9, 8.9 Hz), 4.56 (1H, td, J = 9.2, 1.7 Hz), 4.43 (1H, ddd, J = 10.2, 9.0, 6.1 Hz), 2.88 (1H, dddd, J = 13.0, 9.0, 6.1, 1.7 Hz), 2.56 (1H, dq, J = 13.0, 9.9 Hz); ^{13}C NMR (126 MHz, acetone-d_6) δ 174.2, 141.1, 140.7, 138.4 (2 × C), 134.1, 133.9, 132.7, 132.6, 132.1, 131.9, 125.3, 125.2, 79.7, 66.8, 30.2; ^{19}F NMR (471 MHz, acetone-d_6) δ −78.9; IR (neat, cm^{-1}): 3086, 3000, 2922, 1768, 1581, 1483, 1449, 1428, 1401, 1379, 1341, 1253, 1222, 1150, 1073; HRMS (ESI): Found (M−OTf)$^+$ 285.0579, $C_{16}H_{13}O_3S$ requires 285.0580.

The quaternary carbon corresponding to the CF_3 in the triflate counter anion was not observed, though its presence was confirmed by ^{19}F NMR.

5-(Cyclohexyloxy)-5*H*-dibenzo[*b*,*d*]thiophen-5-ium triflate (1ac)

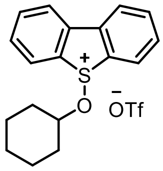

Following **GP1**, dibenzo[*b*,*d*]thiophene 5-oxide (0.20 g, 2.5 mmol, 1.0 equiv.) and cyclohexanol (0.34 mL, 3.75 mmol, 1.5 equiv.) gave **1ac** (0.99 g, 92%) as a solid. ^{1}H NMR (500 MHz, $CDCl_3$) δ 8.27 (2H, d, J = 7.8 Hz), 7.98 (2H, d, J = 7.7 Hz), 7.83 (2H, td, J = 7.6, 1.1 Hz), 7.64 (2H, td, J = 7.8, 1.1 Hz), 4.96 (1H, tt, J = 8.9, 4.0 Hz), 2.10–2.02 (2H, m), 1.79–1.69 (2H, m), 1.64–1.47 (3H, m), 1.47–1.36 (2H, m), 1.27–1.15 (1H, m); ^{13}C NMR (126 MHz, $CDCl_3$) δ 139.6, 137.2, 132.3, 131.9, 130.8, 124.1, 90.2, 33.5, 24.6, 23.7; ^{19}F NMR (471 MHz, $CDCl_3$) δ −78.2; HRMS (ESI): Found $(M-OTf)^+$ 283.1138, $C_{18}H_{19}OS$ requires 283.1151.
The quaternary carbon corresponding to the CF_3 in the triflate counter anion was not observed, though its presence was confirmed by ^{19}F NMR.

5-((4,4-Difluorocyclohexyl)oxy)-5*H*-dibenzo[*b*,*d*]thiophen-5-ium triflate (1ad)

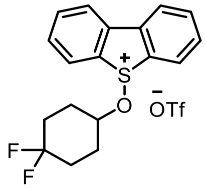

Following **GP1**, dibenzo[*b*,*d*]thiophene 5-oxide (0.20 g, 1.0 mmol, 1.0 equiv.) and 4,4-difluorocyclohexanol (0.16 g, 1.2 mmol, 1.2 equiv.) gave **1ad** (0.37 g, 84%) as an oil. ^{1}H NMR (500 MHz, acetone-d_6) δ 8.67 (2H, d, J = 8.0 Hz), 8.36 (2H, d, J = 7.8 Hz), 8.05 (2H, td, J = 7.6, 1.1 Hz), 7.84 (2H, td, J = 7.8, 1.1 Hz), 5.07–4.99 (1H, m), 2.15–2.06 (2H, m), 2.03–1.91 (6H, m); ^{13}C NMR (126 MHz, acetone-d_6) δ 141.2, 138.2, 133.1, 132.7, 131.5, 125.3, 123.0 (t, $J_{C\text{-}F}$ = 240.6 Hz), 84.6, 30.4 (t, $J_{C\text{-}F}$ = 30.3 Hz), 30.3 (t, $J_{C\text{-}F}$ = 30.3 Hz), 29.6 (t, $J_{C\text{-}F}$ = 6.7 Hz), 29.5 (t, $J_{C\text{-}F}$ = 6.7 Hz); ^{19}F NMR (471 MHz, $CDCl_3$) δ −78.8, −95.6 (d, J = 238.4 Hz), −101.1 (d, J = 238.4 Hz); HRMS (ESI): Found $(M-OTf)^+$ 319.0951, $C_{18}H_{17}OF_2S$ requires 319.0963.
The quaternary carbon corresponding to the CF_3 in the triflate counter anion was not observed, though its presence was confirmed by ^{19}F NMR.

5-((Tetrahydro-2*H*-pyran-4-yl)oxy)-5*H*-dibenzo[*b*,*d*]thiophen-5-ium triflate (1ae)

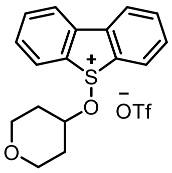

Following **GP1**, dibenzo[*b*,*d*]thiophene 5-oxide (0.20 g, 1.0 mmol, 1.0 equiv.) and tetrahydro-4-pyranol (0.14 mL, 1.5 mmol, 1.5 equiv.) gave **1ae** (0.41 g, 94%) as a solid. ^{1}H NMR (400 MHz, acetone-*d*$_6$) δ 8.62 (2H, d, *J* = 7.9 Hz), 8.34 (2H, d, *J* = 7.7 Hz), 8.03 (2H, t, *J* = 7.7 Hz), 7.83 (2H, t, *J* = 7.7 Hz), 5.08 (1H, tt, *J* = 9.0, 4.3 Hz), 3.83 (2H, dt, *J* = 11.9, 4.4 Hz), 3.42 (2H, ddd, *J* = 11.9, 9.4, 2.7 Hz), 2.13–2.07 (2H, m), 1.82–1.72 (2H, m); ^{13}C NMR (101 MHz, acetone-*d*$_6$) δ 141.0, 138.1, 133.4, 132.6, 131.4, 125.3, 85.8, 65.4, 34.3; ^{19}F NMR (376 MHz, $CDCl_3$) δ −78.9; HRMS (ESI): Found (M−OTf)$^+$ 285.0937, $C_{17}H_{17}O_2S$ requires 285.0944.
The quaternary carbon corresponding to the CF_3 in the triflate counter anion was not observed, though its presence was confirmed by ^{19}F NMR.

5-((4-Oxocyclohexyl)oxy)-5*H*-dibenzo[*b*,*d*]thiophen-5-ium triflate (1af)

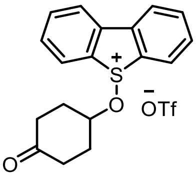

Following **GP1**, dibenzo[*b*,*d*]thiophene 5-oxide (0.20 g, 1.0 mmol, 1.0 equiv.) and 1,4-dioxaspiro[4.5]decan-8-ol (0.19 g, 1.2 mmol, 1.2 equiv.) gave **1af** (0.40 g, 89%) as an oil. ^{1}H NMR (400 MHz, acetone-*d*$_6$) δ 8.70 (2H, d, *J* = 7.9 Hz), 8.37 (2H, d, *J* = 7.9 Hz), 8.06 (2H, td, *J* = 7.7, 1.1 Hz), 7.87 (2H, td, *J* = 7.7, 1.1 Hz), 5.20 (1H, tt, *J* = 6.4, 3.5 Hz), 2.53–2.43 (2H, m), 2.38–2.27 (2H, m), 2.24–2.13 (4H, m); ^{13}C NMR (101 MHz, acetone-*d*$_6$) δ 206.5, 141.2, 138.2, 133.1, 132.7, 131.5, 125.4, 85.2, 36.9, 32.3; ^{19}F NMR (376 MHz, $CDCl_3$) δ −79.0; HRMS (ESI): Found (M−OTf)$^+$ 297.0944, $C_{18}H_{17}O_2S$ requires 297.0944.
The quaternary carbon corresponding to the CF_3 in the triflate counter anion was not observed, though its presence was confirmed by ^{19}F NMR.

5-(Cycloheptyloxy)-5*H*-dibenzo[*b*,*d*]thiophen-5-ium triflate (1ag)

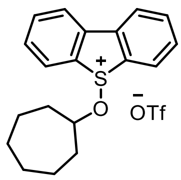

Following **GP1**, dibenzo[*b*,*d*]thiophene 5-oxide (0.20 g, 1.0 mmol, 1.0 equiv.) and cycloheptanol (0.18 mL, 1.5 mmol, 1.5 equiv.) gave **1ag** (0.30 g, 67%) as a solid. ^{1}H NMR (400 MHz, acetone-d_6) δ 8.60 (2H, d, J = 8.1 Hz), 8.35 (2H, d, J = 7.8 Hz), 8.05 (2H, td, J = 7.7, 1.1 Hz), 7.85 (2H, td, J = 7.8, 1.2 Hz), 4.90 (1H, tt, J = 7.7, 4.7 Hz), 2.03–1.95 (2H, m), 1.94–1.82 (2H, m), 1.69–1.57 (2H, m), 1.54–1.45 (4H, m), 1.43–1.31 (2H, m); ^{13}C NMR (101 MHz, acetone-d_6) δ 141.1, 138.1, 133.2, 132.6, 131.3, 125.2, 92.1, 36.0, 28.7, 22.8; ^{19}F NMR (376 MHz, $CDCl_3$) δ −79.0; HRMS (ESI): Found (M−OTf)$^+$ 297.1297, $C_{19}H_{21}OS$ requires 297.1308.

The quaternary carbon corresponding to the CF_3 in the triflate counter anion was not observed, though its presence was confirmed by ^{19}F NMR.

5-((Adamantan-2-yl)oxy)-5*H*-dibenzo[*b*,*d*]thiophen-5-ium triflate (1ah)

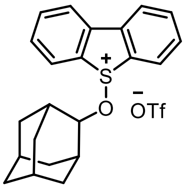

Following **GP1**, dibenzo[*b*,*d*]thiophene 5-oxide (0.20 g, 1.0 mmol, 1.0 equiv.) and 2-adamantanol (0.18 mg, 1.2 mmol, 1.2 equiv.) gave **1ah** (0.42 g, 87%) as a solid. ^{1}H NMR (500 MHz, acetone-d_6) δ 8.60 (2H, d, J = 7.9 Hz), 8.35 (2H, d, J = 7.7 Hz), 8.03 (2H, td, J = 7.7, 1.1 Hz), 7.83 (2H, td, J = 7.7, 1.1 Hz), 5.01 (1H, t, J = 3.6 Hz), 2.20–2.14 (2H, m), 1.96 (2H, d, J = 13.3 Hz), 1.89–1.82 (2H, m), 1.81–1.72 (2H, m), 1.73–1.67 (4H, m), 1.63–1.56 (2H, m); ^{13}C NMR (126 MHz, acetone-d_6) δ 140.9, 138.0, 133.5, 132.6, 131.3, 125.3, 122.3 (q, $J_{C\text{-}F}$ = 321.8 Hz), 93.8, 37.3, 36.9, 34.6, 31.5, 27.6, 27.2; ^{19}F NMR (471 MHz, $CDCl_3$) δ −78.8; HRMS (ESI): Found (M−OTf)$^+$ 335.1456, $C_{22}H_{23}OS$ requires 335.1464.

The quaternary carbon corresponding to the CF_3 in the triflate counter anion was not observed, though its presence was confirmed by ^{19}F NMR.

5-((Adamantan-1-yl)oxy)-5*H*-dibenzo[*b*,*d*]thiophen-5-ium triflate (1ai)

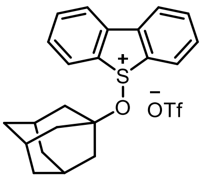

Following **GP1**, dibenzo[*b*,*d*]thiophene 5-oxide (0.20 g, 1.0 mmol, 1.0 equiv.) and 1-adamantanol (0.18 mg, 1.2 mmol, 1.2 equiv.) gave **1ai** (0.46 g, 95%) as a solid. ^{1}H NMR (400 MHz, acetone-d_6) δ 8.38 (2H, d, J = 7.9 Hz), 8.33 (2H, d, J = 7.9 Hz), 8.01 (2H, td, J = 7.6, 1.1 Hz), 7.83 (2H, td, J = 7.7, 1.1 Hz), 2.48–2.42 (3H, m), 2.41–2.37 (6H, m), 1.82–1.76 (6H, m); ^{13}C NMR (126 MHz, acetone-d_6) δ 140.5, 137.8, 134.5, 132.7, 130.9, 125.4, 94.2, 43.7, 35.6, 33.3; ^{19}F NMR (471 MHz, $CDCl_3$) δ −79.0; HRMS (ESI): Found (M−OTf)$^+$ 335.1454, $C_{22}H_{23}OS$ requires 335.1464.
The quaternary carbon corresponding to the CF_3 in the triflate counter anion was not observed, though its presence was confirmed by ^{19}F NMR.

5-((3-Methylbutan-2-yl)oxy)-5*H*-dibenzo[*b*,*d*]thiophen-5-ium triflate (1aj)

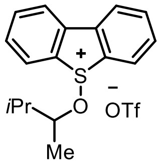

Following **GP1**, dibenzo[*b*,*d*]thiophene 5-oxide (0.50 g, 2.5 mmol, 1.0 equiv.) and 3-Methyl-2-butanol (0.40 mL, 3.75 mmol, 1.5 equiv.) gave **1aj** (1.00 g, 95%) as a solid. ^{1}H NMR (500 MHz, acetone-d_6) δ 8.62 (1H, d, J = 7.9 Hz), 8.58 (1H, d, J = 7.9 Hz), 8.38–8.32 (2H, m), 8.05 (2H, td, J = 7.7, 1.2 Hz), 7.85 (2H, td, J = 7.8, 1.3 Hz), 4.80–4.71 (1H, m), 2.01–1.91 (1H, m), 1.36 (3H, d, J = 6.3 Hz), 0.90 (3H, d, J = 6.8 Hz), 0.86 (3H, d, J = 6.8 Hz); ^{13}C NMR (126 MHz, acetone-d_6) δ 141.2, 141.0, 138.1, 133.3, 133.1, 132.6, 132.5, 131.6, 131.5, 125.3, 125.2, 92.6, 34.7, 18.0, 17.9, 17.3; ^{19}F NMR (471 MHz, $CDCl_3$) δ −78.8; HRMS (ESI): Found (M−OTf)$^+$ 271.1165, $C_{17}H_{19}OS$ requires 271.1151.
The quaternary carbon corresponding to the CF_3 in the triflate counter anion was not observed, though its presence was confirmed by ^{19}F NMR.

General Procedure for the Preparation of Alkenes via Pd Catalyzed Cross-Coupling Reaction – GP2

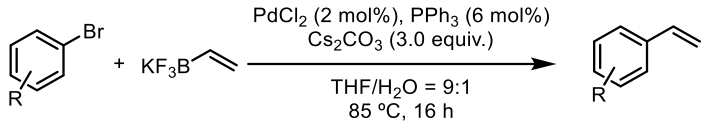

A Schlenk flask equipped with a stirring bar was charged with potassium vinyltrifluoroborate (0.15 g, 1.1 mmol, 1.1 equiv.), $PdCl_2$ (3.5 mg, 0.02 mmol, 2 mol%), PPh_3 (16.0 mg, 0.06 mmol, 6 mol%) and Cs_2CO_3 (0.98 g, 3.0 mmol, 3.0 equiv.). The corresponding bromobenzene derivative (1.0 mmol, 1.0 equiv.) was dissolved in 2 mL of THF/water (9:1), and added to the reaction mixture followed by heating at 85 °C for 16 h. Then the reaction mixture was cooled to room temperature and diluted with H_2O (5 mL). The layers were separated and the aqueous layer was extracted with CH_2Cl_2 (3 × 15 mL). The combined organic layers were washed with brine (30 mL), dried ($MgSO_4$), filtered and evaporated. Purification by column chromatography on silica gel eluting with hexane–EtOAc to give the products.

General Procedure for the Preparation of Alkenes Using the Wittig Reaction – GP3

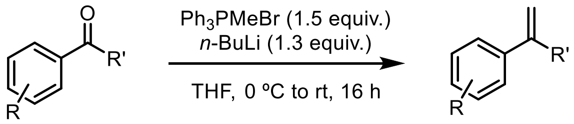

An oven dried round-bottomed flask equipped with a stirring bar was charged with PPh_3MeBr (1.34 g, 3.75 mmol, 1.5 equiv.). Dry THF (20 mL) was added and the reaction was cooled to 0 °C. *n*-BuLi (2.00 mL, 1.6 M in hexane, 3.25 mmol, 1.3 equiv.) was slowly added via syringe and the reaction mixture was stirred at the same temperature for 1 h. To the resulting mixture, a benzaldehyde/acetophenone (2.5 mmol, 1.0 equiv.) in THF (5 mL) was added dropwise at 0 °C. The reaction mixture was stirred for 16 h at room temperature, quenched with H_2O (20 mL) and extracted with Et_2O (3 × 20 mL). The combined organic layers were washed with brine (30 mL), dried ($MgSO_4$), filtered and evaporated. Purification by column chromatography on silica gel eluting with hexane–EtOAc to give the products.

_N_-Methyl-_N_-(4-vinylphenyl)acetamide (3b)

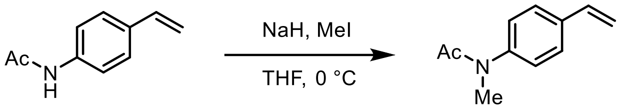

NaH (60% in mineral oil, 0.16 g, 4.0 mmol, 2.0 equiv.) was added to a solution of *N*-(4-vinylphenyl)acetamide (0.32 g, 2.0 mmol, 1.0 equiv.) in dry THF (20 ml, 0.1 M) at 0 °C under argon atmosphere. After stirring at room temperature for 30 min, MeI (0.25 ml, 4.0 mmol, 2.0 equiv.) was added and the reaction mixture was stirred overnight. The reaction mixture was then quenched with H_2O (10 ml), and the aqueous layer extracted with EtOAc (3 × 10 mL). The combined organic layers were washed with brine (10 mL), dried ($MgSO_4$), filtered and evaporated. The residue was purified by column chromatography on silica gel eluting with hexane–EtOAc to give **3b** (0.35 g, 99%) as a solid. ^{1}H NMR (500 MHz, $CDCl_3$) δ 7.43 (2H, d, J = 8.2 Hz), 7.13 (2H, d, J = 8.0 Hz), 6.71 (1H, dd, J = 17.6, 10.9 Hz), 5.76 (1H, d, J = 17.6 Hz), 5.30 (1H, d, J = 10.9 Hz), 3.25 (3H, s), 1.88 (3H, s); ^{13}C NMR (126 MHz, $CDCl_3$) δ 170.6, 144.1, 137.1, 135.8, 127.5, 127.3, 115.1, 37.2, 22.5. Data in accordance with the literature.[1]

4-Vinylbenzyl acetate (3c)

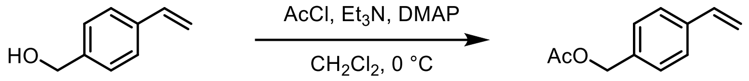

To a solution of (4-vinylphenyl)methanol (0.27 g, 2.0 mmol, 1.0 equiv.) in CH_2Cl_2 (10 mL, 0.2 M) was added Et_3N (0.55 mL, 4.0 mmol, 2.0 equiv.) and 4-(dimethylamino)pyridine (24 mg, 0.2 mmol, 0.1 equiv.). The resulting mixture was cooled down to 0 °C and acetyl chloride (0.16 mL, 2.2 mmol, 1.1 equiv.) was added slowly. The reaction mixture was allowed to warm up to room temperature and stirred overnight. A saturated solution of $NaHCO_3$ (10 mL) was added, and the aqueous layer was extracted with CH_2Cl_2 (3 × 10 mL). The combined organic layers were washed with brine (10 mL), dried ($MgSO_4$), filtered and evaporated. The residue was purified by column chromatography on silica gel eluting with hexane–EtOAc to give **3c** (0.32 g, 91%) as an oil. ^{1}H NMR (400 MHz, $CDCl_3$) δ 7.41 (2H, d, J = 8.2 Hz), 7.32 (2H, d, J = 8.2 Hz), 6.72 (1H, dd, J = 17.6, 10.9 Hz), 5.76 (1H, dd, J = 17.6, 1.0 Hz), 5.27 (1H, dd, J = 10.8, 1.0 Hz), 5.09 (2H, s), 2.10 (3H, s); ^{13}C NMR (101 MHz, $CDCl_3$) δ 171.0, 137.7, 136.5, 135.5, 128.7, 126.5, 114.5, 66.2, 21.2. Data in accordance with the literature.[2]

4-Vinylbenzyl phenylcarbamate (3d)

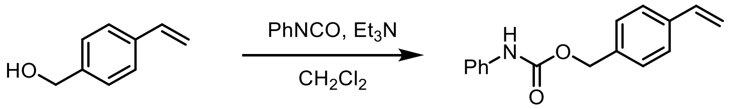

To a solution of (4-vinylphenyl)methanol (0.27 g, 2.0 mmol, 1.0 equiv.) in CH_2Cl_2 (10 mL, 0.2 M) was added phenyl isocyanate (0.24 mL, 2.2 mol, 1.1 equiv.), and Et_3N (0.28 mL, 2.0 mmol, 1.0 equiv.) at room temperature. The resulting reaction mixture was stirred at room temperature until complete consumption of the starting material (monitored by TLC). The solvent was removed under reduced pressure. The residue was purified by column chromatography on silica gel eluting with hexane–EtOAc to give **3d** (0.48 g, 95%) as a solid. R_f 0.47 [hexane–EtOAc (4:1)]; ^{1}H NMR (500 MHz, $CDCl_3$) δ 7.42 (2H, d, *J* = 8.2 Hz), 7.41–7.38 (2H, m), 7.36 (2H, d, *J* = 8.2 Hz), 7.35–7.28 (2H, m), 7.08 (1H, tt, *J* = 7.3, 1.2 Hz), 6.79 (1H, br s), 6.73 (1H, dd, *J* = 17.6, 10.9 Hz), 5.78 (1H, dd, *J* = 17.6, 0.9 Hz), 5.29 (1H, dd, *J* = 10.9, 0.9 Hz), 5.19 (2H, s); ^{13}C NMR (126 MHz, $CDCl_3$) δ 153.5, 137.9, 137.8, 136.4, 135.6, 129.2, 128.7, 126.5, 123.6, 118.8, 114.5, 66.8; HRMS (APCI): Found $(M+Na)^+$ 276.0996, $C_{16}H_{15}NO_2Na$ requires 276.0995.

6-Vinylbenzo[*b*]thiophene (3e)

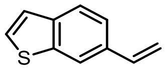

Following **GP2**, 6-bromobenzo[*b*]thiophene (0.21 g, 1.0 mmol) gave **3e** (0.11 g, 66%) as a solid. ^{1}H NMR (400 MHz, $CDCl_3$) δ 7.81–7.72 (2H, m), 7.43 (1H, d, *J* = 8.4, 1.7 Hz), 7.38 (1H, d, *J* = 5.4 Hz), 7.27 (1H, d, *J* = 5.4 Hz), 6.81 (1H, dd, *J* = 17.6, 10.9 Hz), 5.79 (1H, d, *J* = 17.6 Hz), 5.26 (1H, d, *J* = 10.9 Hz); ^{13}C NMR (101 MHz, $CDCl_3$) δ 140.1, 139.2, 137.0, 134.1, 127.0, 124.1, 122.5, 122.3, 121.8, 113.5. Data in accordance with literature.[3]

2-Vinyldibenzo[*b*,*d*]thiophene (3f)

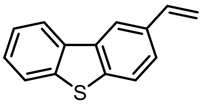

Following **GP2**, 2-bromodibenzo[*b*,*d*]thiophene (0.26 g, 1.0 mmol) gave **3f** (0.16 g, 77%) as a solid. ^{1}H NMR (400 MHz, $CDCl_3$) δ 8.20–8.14 (2H, m), 7.88–7.83 (1H, m), 7.80 (1H, d, *J* = 8.3 Hz), 7.56 (1H, dd, *J* = 8.4, 1.7 Hz), 7.50–7.44 (2H, m), 6.90 (1H, dd, *J* = 17.6, 10.9 Hz), 5.88 (1H, d, *J* = 17.6 Hz), 5.34 (1H, d, *J* = 10.9 Hz); ^{13}C NMR (101 MHz, $CDCl_3$) δ 140.0, 139.0, 136.9, 136.0, 135.6, 134.4, 127.0, 124.9, 124.6, 123.0, 121.7, 122.9, 119.6, 113.8. Data in accordance with literature.[4]

2-Bromo-5-vinylthiophene (3g)

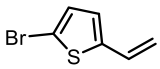

Following **GP3**, 5-bromothiophene-2-carbaldehyde (0.48 g, 2.5 mmol) gave **3g** (0.35 g, 73%) as an oil. ^{1}H NMR (400 MHz, $CDCl_3$) δ 6.92 (1H, d, J = 3.8 Hz), 6.76–6.58 (2H, m), 5.47 (1H, d, J = 17.3 Hz), 5.15 (1H, d, J = 10.9 Hz); ^{13}C NMR (101 MHz, $CDCl_3$) δ 144.8, 130.3, 129.5, 126.2, 113.9, 111.4. Data in accordance with literature.[5]

2-(Prop-1-en-2-yl)benzo[*b*]thiophene (3h)

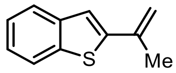

Following **GP3**, 1-(benzo[*b*]thiophen-2-yl)ethan-1-one (0.44 g, 2.5 mmol) gave **3h** (0.30 g, 70%) as a solid. ^{1}H NMR (400 MHz, $CDCl_3$) δ 7.80–7.75 (1H, m), 7.74–7.69 (1H, m), 7.36–7.28 (2H, m), 7.23 (1H, s), 5.52 (1H, s), 5.15 (1H, s), 2.25 (3H, s); ^{13}C NMR (101 MHz, $CDCl_3$) δ 145.7, 140.5, 139.2, 137.7, 124.8, 124.4, 123.6, 122.2, 120.7, 114.2, 21.5. Data in accordance with literature.[6]

2-(Prop-1-en-2-yl)pyridine (3i)

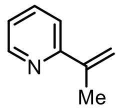

Following **GP3**, 2-acetylpyridine (0.28 mL, 2.5 mmol) gave **3i** (0.18 g, 60%) as an oil. ^{1}H NMR (500 MHz, $CDCl_3$) δ 8.59 (1H, d, J = 4.8 Hz), 7.65 (1H, td, J = 7.8, 1.9 Hz), 7.48 (1H, d, J = 8.3 Hz), 7.16 (1H, dd, J = 7.5, 4.8 Hz), 5.88–5.84 (1H, m), 5.33–5.29 (1H, m), 2.22 (3H, s); ^{13}C NMR (126 MHz, $CDCl_3$) δ 158.3, 149.0, 143.3, 136.5, 122.2, 119.9, 115.9, 20.6. Data in accordance with literature.[7]

(2-Methylprop-1-en-1-yl)benzene (3j)

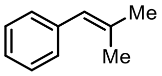

Following **GP3**, benzaldehyde (0.21 g, 2.0 mmol) and isopropyltriphenylphosphonium iodide (1.56 g, 3.6 mmol) gave **3j** (0.22 g, 84%) as an oil. ^{1}H NMR (400 MHz, $CDCl_3$) δ 7.35–7.28 (2H, m), 7.25–7.21 (2H, m), 7.21–7.15 (1H, m), 6.27 (1H, s), 1.91 (3H, d, J = 1.4 Hz), 1.87 (3H, d, J = 1.4 Hz); ^{13}C NMR (101 MHz, $CDCl_3$) δ 138.8, 135.6, 128.9, 128.2, 125.9, 125.2, 27.0, 19.5. Data in accordance with literature.[8]

(*E*)-2-Methyl-4-phenylbut-3-en-2-ol (3k)

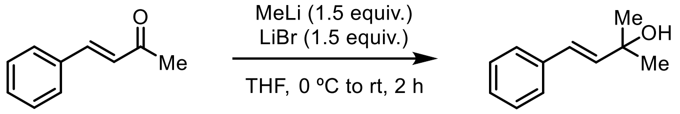

To a solution of (*E*)-4-phenylbut-3-en-2-one (0.29 g, 2.0 mmol, 1.0 equiv.) and LiBr (0.26 g, 3.0 mmol, 1.5 equiv.) in THF (10 mL, 0.2 M) was added MeLi (1.88 mL, 1.6 M in Et_2O, 3.0 mmol, 1.5 equiv.) dropwise while stirring at 0 °C. The reaction was further stirred and allowed to warm up to room temperature for 2 h. Then the reaction mixture was quenched with NH_4Cl (10 mL, saturated), and the aqueous layer was extracted with EtOAc (3 × 10 mL). The combined organic layers were washed with brine (20 mL), dried ($MgSO_4$), filtered and evaporated. The residue was purified by column chromatography on silica gel eluting with hexane–EtOAc to give **3k** (0.30 g, 93%) as a solid. ^{1}H NMR (400 MHz, $CDCl_3$) δ 7.42–7.36 (2H, m), 7.35–7.28 (2H, m), 7.25–7.20 (1H, m), 6.59 (1H, d, *J* = 16.1 Hz), 6.36 (1H, d, *J* = 16.1 Hz), 1.43 (6H, s); ^{13}C NMR (101 MHz, $CDCl_3$) δ 137.6, 137.0, 128.7, 127.6, 126.5 (2 × C), 71.2, 30.0. Data in accordance with literature.[9]

(*E*)-2,2-Dimethyl-5-phenylpent-4-enenitrile (3l)

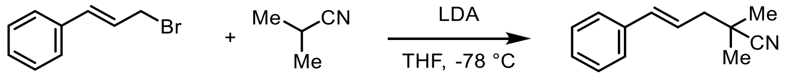

Isobutyronitrile (0.90 mL, 10.0 mmol, 1.0 equiv.) was added dropwise to a solution of lithium diisopropylamide (6.0 mL, 2.0 M in THF, 12.0 mmol, 1.2 equiv.) in dry THF (20 mL) at –78 °C and the solution was stirred at –78 °C for a further 30 min. Then, a solution of 3-bromo-1-phenyl-1-propene (2.36 g, 12.0 mmol, 1.2 equiv.) in dry THF (10 mL) was added dropwise to the reaction mixture. Upon completion of the addition, the reaction was allowed to warm to room temperature and stirred for 2 h. The reaction mixture was then quenched with NH_4Cl (20 mL, saturated), and the aqueous layer was extracted with Et_2O (3 × 30 mL). The combined organic layers were washed with brine (30 mL), dried ($MgSO_4$), filtered and evaporated. The residue was purified by column chromatography on silica gel eluting with hexane–EtOAc to give (*E*)-2,2-dimethyl-5-phenylpent-4-enenitrile **3l** (1.76 g, 95%) as a solid. ^{1}H NMR (500 MHz, $CDCl_3$) δ 7.39 (2H, d, *J* = 7.3 Hz), 7.33 (2H, t, *J* = 7.6 Hz), 7.25 (1H, t, *J* = 7.3 Hz), 6.52 (1H, d, *J* = 15.6 Hz), 6.26 (1H, dt, *J* = 15.4, 7.4 Hz), 2.44 (2H, d, *J* = 7.5 Hz), 1.39 (6H, s) ^{13}C NMR (126 MHz, $CDCl_3$) δ 136.8, 134.9, 128.7, 127.8, 126.5, 124.9, 123.7, 44.5, 32.7, 26.5. Data in accordance with the literature.[10]

(1-Cyclopropylvinyl)benzene (3m)

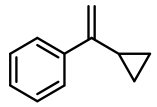

Following **GP3**, 4-chloro-1-phenylbutan-1-one (0.46 g, 2.5 mmol) gave **3m** (0.11 g, 31%) as an oil. ^{1}H NMR (500 MHz, $CDCl_3$) δ 7.62–7.58 (2H, m), 7.37–7.32 (2H, m), 7.30–7.27 (1H, m), 5.28 (1H, s), 4.94 (1H, s), 1.66 (1H, dddd, J = 13.7, 8.3, 5.4, 1.2 Hz), 0.86–0.82 (2H, m), 0.62–0.58 (2H, m); ^{13}C NMR (126 MHz, $CDCl_3$) δ 149.5, 141.8, 128.3, 127.6, 126.3, 109.2, 15.8, 6.8. Data in accordance with literature.[11]

***tert*-Butyl 7-chloro-5-methylene-2,3,4,5-tetrahydro-1*H*-benzo[*b*]azepine-1-carboxylate (3n)**

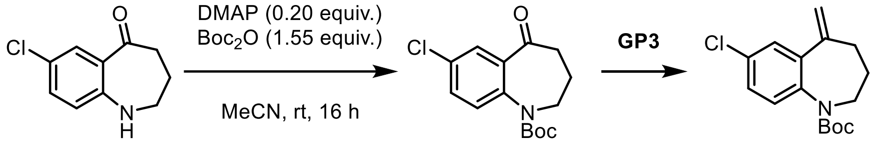

Step 1: A round-bottom flask equipped with a stirring bar was charged with 7-chloro-1,2,3,4-tetrahydro-5*H*-benzo[*b*]azepin-5-one (0.98 g, 5.0 mmol, 1.0 equiv.), DMAP (0.13 g, 1.02 mmol, 0.20 equiv.) and Boc_2O (1.69 g, 7.75 mmol, 1.55 equiv.). Dry MeCN (17.5 mL) was added and the reaction was stirred for 16 h. Then, the solvent was evaporated and the residue diluted with EtOAc (20 mL), washed with brine (3 × 10 mL), dried ($MgSO_4$), filtered and evaporated. The residue was used in the next step without further purification.

Step 2: Following **GP3**, *tert*-butyl 7-chloro-5-oxo-2,3,4,5-tetrahydro-1*H*-benzo[*b*]azepine-1-carboxylate (0.59 g, 2.0 mmol) gave **3n** (0.33 g, 57%) as a solid. ^{1}H NMR (400 MHz, DMSO-d_6) δ 7.36 (1H, br s), 7.30 (1H, dd, J = 8.5, 2.5 Hz), 7.22 (1H, d, J = 8.5 Hz), 5.22 (1H, br s), 5.19 (1H, s), 3.51 (2H, br s), 2.36 (2H, t, J = 6.2 Hz), 1.88–1.75 (2H, m), 1.35 (9H, br s); ^{13}C NMR (101 MHz, DMSO-d_6) δ 153.3, 146.7, 138.9, 130.2, 130.1, 127.5, 127.2, 116.5, 79.7, 47.6, 33.3, 27.9. Data in accordance with literature.[12]

Some of the carbon peaks in the ^{13}C NMR spectrum are missing due to the overlapping signals resulting from the rotation of Boc group.

2-(2-Hydroxyethyl)isoindoline-1,3-dione (3o)

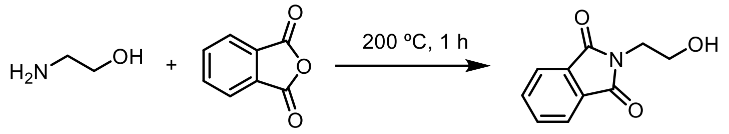

A mixture of 2-aminoethan-1-ol (0.75 g, 10.0 mmol, 1.0 equiv.) and phthalic anhydride (1.77 g, 12.0 mmol, 1.0 equiv.) was heated at 200 °C for 1 hour. The reaction mixture was cooled down to room temperature, diluted with CH_2Cl_2 (25 mL) and stirred for 30 minutes. After filtration, the combined organic layers were washed with H_2O (10 mL), brine (10 mL), dried ($MgSO_4$), filtrated and evaporated to give **3o** (1.89 g, 99%) as a solid. ^{1}H NMR (400 MHz, $CDCl_3$) δ 7.85 (2H, dd, J = 5.4, 3.1 Hz), 7.72 (2H, dd, J = 5.4, 3.1 Hz), 3.92–3.83 (4H, m), 2.32 (1H, br s); ^{13}C NMR (101 MHz, $CDCl_3$) δ 169.0, 134.3, 132.1, 123.5, 61.2, 41.0. Data in accordance with literature.[13]

Methyl (*S*)-2-(1,3-dioxoisoindolin-2-yl)-3-hydroxypropanoate (3p)

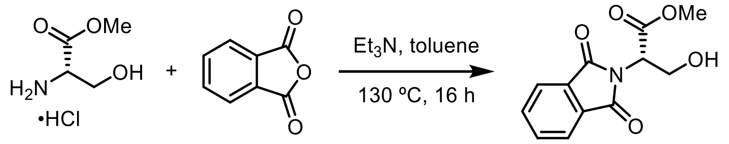

To a solution of methyl L-serinate hydrochloride (1.00 g, 6.4 mmol, 1.0 equiv.) and phthalic anhydride (0.95 g, 6.4 mmol, 1.0 equiv.) in toluene (20 mL) was added triethylamine (0.90 mL, 6.4 mmol, 1.0 equiv.). The reaction mixture was heated to reflux with a Dean-Stark set-up for 16 h. Then, the solvent was evaporated and the resulting mixture diluted in EtOAc (30 mL). The combined organic layers were washed with H_2O (15 mL), brine (15 mL), dried ($MgSO_4$), filtered and evaporated. Purification by column chromatography on silica gel eluting with hexane-EtOAc to give **3p** (1.24 g, 78%) as an oil. ^{1}H NMR (500 MHz, $CDCl_3$) δ 7.88 (2H, dd, J = 5.5, 3.1 Hz), 7.76 (2H, dd, J = 5.5, 3.1 Hz), 5.03 (1H, dd, J = 5.7, 4.3 Hz), 4.22 (1H, dd, J = 12.2, 5.7 Hz), 4.19 (1H, dd, J = 12.2, 4.3 Hz), 3.78 (3H, s), 3.47 (1H, br s); ^{13}C NMR (126 MHz, $CDCl_3$) δ 168.6, 168.2, 134.6, 131.8, 123.9, 61.2, 54.9, 53.1. Data in accordance with literature.[14]

Methyl 2-(1,3-dioxoisoindolin-2-yl)-3-hydroxypropanoate (3p')

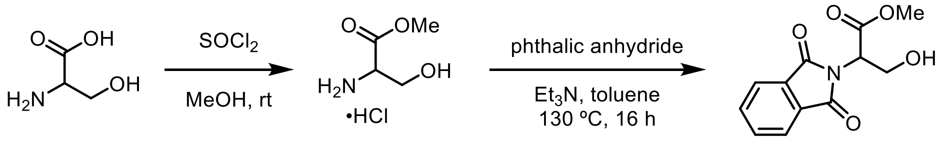

Step 1: $SOCl_2$ (1.38 mL, 19.1 mmol, 2.0 equiv.) was added to a solution of DL-serine (1.00 g, 9.53 mmol, 1.0 equiv.) in MeOH (20 mL, 0.48 M) at 0 °C. Then, the mixture was stired at room

temperature overnight and evaporated to dryness to give the corresponding methyl serinate hydrochloride which was directly used without further purification.

Step 2: Following the procedure described for the synthesis of **3p** but using methyl serinate hydrochloride (9.53 mmol, 1.0 equiv.) and phthalic anhydride (1.41 g, 9.53 mmol, 1.0 equiv.) gave **3p'** (1.36 g, 57%) as a solid. ^{1}H NMR (500 MHz, $CDCl_3$) δ 7.85 (2H, dd, *J* = 5.5, 3.1 Hz), 7.74 (2H, dd, *J* = 5.5, 3.1 Hz), 5.01 (1H, dd, *J* = 6.0, 4.4 Hz), 4.21 (1H, dd, *J* = 12.2, 6.0 Hz), 4.17 (1H, dd, *J* = 12.2, 4.4 Hz), 3.76 (3H, s), 3.29 (1H, br s); ^{13}C NMR (126 MHz, $CDCl_3$) δ 168.6, 168.2, 134.6, 131.7, 123.8, 61.0, 54.8, 53.0. Data in accordance with literature.[14]

3 Reaction Optimization

General Procedure for the 1,2-Alkoxy-Hydroxylation Reaction Optimization – GP4

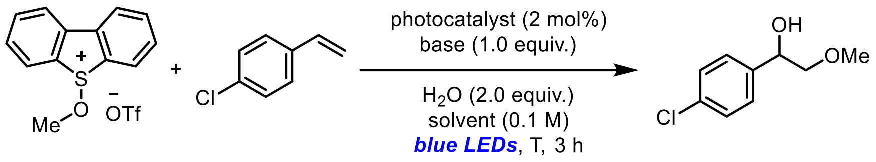

A dry tube equipped with a stirring bar was charged with the photocatalyst (2 µmol, 2 mol%), methoxysulfonium salt **1a** (36 mg, 0.10 mmol, 1.0 equiv.), and base (if solid) (0.10 mmol, 1.0 equiv.) The tube was capped with a Supelco aluminium crimp seal with septum (PTFE/butyl), then evacuated under high vacuum and backfilled with N_2 (3 times). Degassed solvent (1 mL, 0.1 M), base (if liquid) (0.1 mmol, 1.0 equiv.), 4-chlorostyrene (24 µL, 0.20 mmol, 2.0 equiv.), and H_2O (4 µL, 0.20 mmol, 2.0 equiv.) were sequentially added. The vial was purged with a stream of N_2 and the lid sealed with parafilm and placed approximately 5 cm from blue LEDs. The blue LEDs were switched on and the mixture was stirred under irradiation at the specified temperature for 3 h. The tube was opened, and mesitylene (14 µL, 0.1 mmol, 1.0 equiv.) was added as an NMR standard. $CDCl_3$ (1.0 mL) was then introduced. A 1.0 mL aliquot of the resulting mixture was filtered through a short pad of anhydrous $MgSO_4$ directly into an NMR tube for analysis by ^1H NMR spectroscopy to determine the NMR yield.

Table S1. Reaction optimization for the 1,2-alkoxy-hydroxylation reaction: the effect of base, solvent, and photocatalyst.

Entry	photocatalyst	Sulfonium Salt	Base	Solvent	Temperature (°C)	Yield (%)
**1**	4CzIPN	**1a**	$NaH_2PO_4{\cdot}2H_2O$	acetone	room temperature	35
**2**	4CzIPN	**1a**	$NaH_2PO_4{\cdot}2H_2O$	acetone	-20	55
**3**	4CzIPN	**1a**	Na_2HPO_4	acetone	-20	55
**4**	4CzIPN	**1a**	K_3PO_4	acetone	-20	49
**5**	4CzIPN	**1a**	Na_2CO_3	acetone	-20	41
**6**	4CzIPN	**1a**	K_2CO_3	acetone	-20	32
**7**	4CzIPN	**1a**	NaOAc	acetone	-20	18
**8**	4CzIPN	**1a**	2-Cl-pyridine	acetone	-20	51
**9**	4CzIPN	**1a**	pyridine	acetone	-20	11
**10**	4CzIPN	**1a**	$NaH_2PO_4{\cdot}2H_2O$	CH_2Cl_2	-20	5
**11**	4CzIPN	**1a**	$NaH_2PO_4{\cdot}2H_2O$	EtOAc	-20	11
**12**	4CzIPN	**1a**	$NaH_2PO_4{\cdot}2H_2O$	THF	-20	9
**13**	4CzIPN	**1a**	$NaH_2PO_4{\cdot}2H_2O$	MeCN	-20	8
**14**	4CzIPN	**1a**	$NaH_2PO_4{\cdot}2H_2O$	MeOH	-20	-
**15**	4CzIPN	**1a**	$NaH_2PO_4{\cdot}2H_2O$	DMF	-20	trace
**16**	*fac*-$Ir(ppy)_3$	**1a**	$NaH_2PO_4{\cdot}2H_2O$	acetone	-20	32
**17**	$Ir[dF(CF_3)ppy]_2(dtbbpy)PF_6$	**1a**	$NaH_2PO_4{\cdot}2H_2O$	acetone	-20	30
**18**	$Ir(ppy)_2(dtbbpy)PF_6$	**1a**	$NaH_2PO_4{\cdot}2H_2O$	acetone	-20	55
**19**	$Ru(bpy)_3(PF_6)_2$	**1a**	$NaH_2PO_4{\cdot}2H_2O$	acetone	-20	66
**20**	$Ru(bpy)_3Cl_2{\bullet}6H_2O$	**1a**	$NaH_2PO_4{\cdot}2H_2O$	acetone	-20	43
**21**	$Ru(bpz)_3(PF_6)_2$	**1a**	$NaH_2PO_4{\cdot}2H_2O$	acetone	-20	7

Further optimization of the 1,2-alkoxy-hydroxylation reaction was carried out by screening the photocatalyst loading and amount of base at different temperature (**Table S2**).

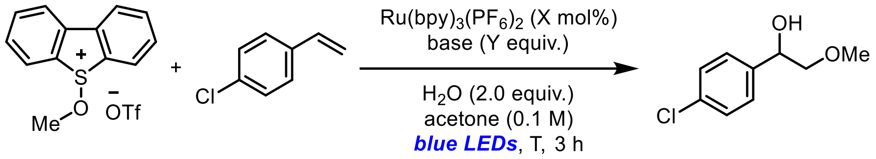

Table S2. Further optimization of the 1,2-alkoxy-hydroxylation reaction: the effect of photocatalyst and base loading.

Entry	Photocatalyst (mol%)	Base	Temperature (°C)	Yield (%)
**1**	$Ru(bpy)_3(PF_6)_2$ (2)	$NaH_2PO_4 \cdot 2H_2O$	-20	66
**2**	$Ru(bpy)_3(PF_6)_2$ (0.1)	$NaH_2PO_4 \cdot 2H_2O$	-20	66
**3**	$Ru(bpy)_3(PF_6)_2$ (0.01)	$NaH_2PO_4 \cdot 2H_2O$	-20	58
**4**	$Ru(bpy)_3(PF_6)_2$ (0.1)	$NaH_2PO_4 \cdot 2H_2O$	-78	75
**5**	$Ru(bpy)_3(PF_6)_2$ (0.1)	Na_2HPO_4	-78	81
**6**[a]	$Ru(bpy)_3(PF_6)_2$ (0.1)	Na_2HPO_4	-78	83

a: 0.5 equiv. Na_2HPO_4 was used as base.

Further optimization and control experiments for the 1,2-alkoxy-hydroxylation reaction were set up based on the optimized reaction (**Table S3**).

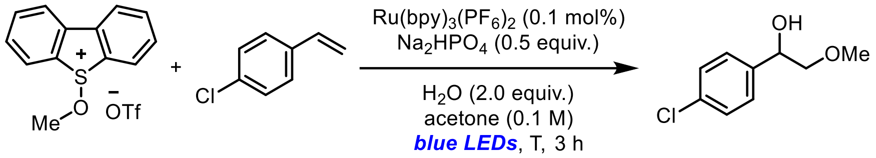

Table S3. Further optimization and control experiments for the 1,2-alkoxy-hydroxylation reaction.

Entry	photocatalyst	Sulfonium Salt	Base	Solvent	Temperature (°C)	Yield (%)
**1**	$Ru(bpy)_3(PF_6)_2$	**1a**	Na_2HPO_4	acetone	-78	83
**2**	$Ru(bpy)_3(PF_6)_2$	**1a**	Na_2HPO_4	acetone	-45	70
**3**	$Ru(bpy)_3(PF_6)_2$	**1a**	Na_2HPO_4	acetone	-30	68
**4**	$Ru(bpy)_3(PF_6)_2$	**1a**	Na_2HPO_4	acetone	-20	62

Entry	photocatalyst	Sulfonium Salt	Base	Solvent	Temperature (°C)	Yield (%)
**5**	$Ru(bpy)_3(PF_6)_2$	**1a**	2-Cl-pyridine	acetone	-78	80
**6**	4CzIPN	**1a**	Na_2HPO_4	acetone	-78	24
**7**	*fac*-$Ir(ppy)_3$	**1a**	Na_2HPO_4	acetone	-78	18
**8**	$Ir[dF(CF_3)ppy]_2(dtbbpy)PF_6$	**1a**	Na_2HPO_4	acetone	-78	30
**9**	$Ru(bpy)_3(PF_6)_2$	**1a**	Na_2HPO_4	CH_2Cl_2	-78	5
**10**	$Ru(bpy)_3(PF_6)_2$	**1a**	Na_2HPO_4	MeOH	-78	-
**11**	$Ru(bpy)_3(PF_6)_2$	**1a**	Na_2HPO_4	DMF	-78	-
**12**	$Ru(bpy)_3(PF_6)_2$	**$1a_1$**	Na_2HPO_4	acetone	-78	83
**13**	$Ru(bpy)_3(PF_6)_2$	**$1a_2$**	Na_2HPO_4	acetone	-78	66
**14**	$Ru(bpy)_3(PF_6)_2$	**$1a_3$**	Na_2HPO_4	acetone	-78	62
**15**	$Ru(bpy)_3(PF_6)_2$	**$1a_4$**	Na_2HPO_4	acetone	-78	18
**16**[a]	$Ru(bpy)_3(PF_6)_2$	**1a**	Na_2HPO_4	acetone	-78	73
**17**[b]	$Ru(bpy)_3(PF_6)_2$	**1a**	Na_2HPO_4	acetone	-78	74
Control Experiments						
**18**	-	**1a**	Na_2HPO_4	acetone	-78	15
**19**	$Ru(bpy)_3(PF_6)_2$	**1a**	-	acetone	-78	71
**20**[c]	$Ru(bpy)_3(PF_6)_2$	**1a**	Na_2HPO_4	acetone	-78	73
**21**[c]	$Ru(bpy)_3(PF_6)_2$	**1a**	-	acetone	-78	53
**22**[d]	$Ru(bpy)_3(PF_6)_2$	**1a**	Na_2HPO_4	acetone	-78	-(80)

[a] reaction run with 5.0 equiv. H_2O; [b] reaction run with **1a** (1.2 equiv.), **3a** (0.1 mmol); [c] reaction run without H_2O. [d] reaction run in the dark. Starting material recovered in parentheses.

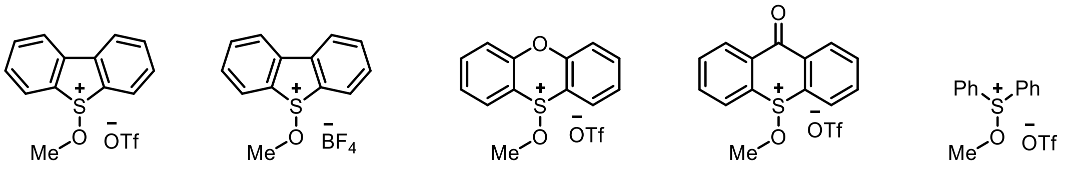

1a **$1a_1$** **$1a_2$** **$1a_3$** **$1a_4$**

4 Substrate Scope

General Procedure for the Photocatalytic 1,2-Alkoxy-Hydroxylation of Alkenes – GP5

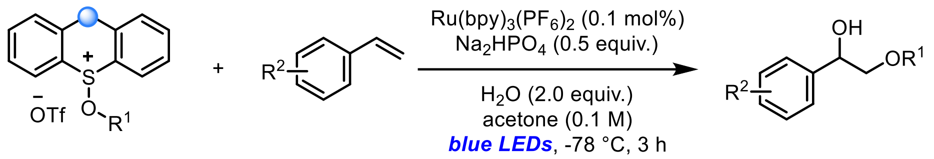

A dry tube equipped with a stirring bar was charged with alkoxy sulfonium salt (0.10 mmol, 1.0 equiv.), Na_2HPO_4 (7 mg, 0.05 mmol, 0.5 equiv.) and alkene (if solid) (0.20 mmol, 2.0 equiv.). The tube was capped with a Supelco aluminium crimp seal with septum (PTFE/butyl), then evacuated under high vacuum and backfilled with N_2 (3 times). Degassed $Ru(bpy)_3(PF_6)_2$ in acetone stock solution (1.0 mL, 0.1 mol%) and alkene (if liquid) (0.20 mmol, 2.0 equiv.), and H_2O (4 µL, 0.20 mmol, 2.0 equiv.) were sequentially added. The vial was purged with a stream of N_2 and the lid sealed with parafilm and placed approximately 5 cm from blue LEDs. The blue LEDs were switched on and the mixture was stirred under irradiation at −78 °C for 3 h. The tube was allowed to warm to room temperature, opened, and the reaction mixture was concentrated and purified by column chromatography on silica gel eluting with hexane–EtOAc.

General Procedure for the Photocatalytic 1,2-Dialkoxylation of Alkenes – GP6

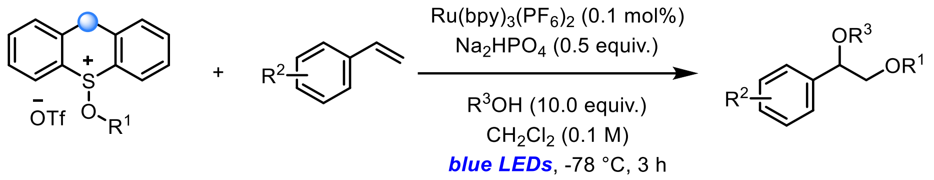

A dry tube equipped with a stirring bar was charged with alkoxy sulfonium salt (0.10 mmol, 1.0 equiv.), Na_2HPO_4 (7 mg, 0.05 mmol, 0.5 equiv.) and alkene (if solid) (0.20 mmol, 2.0 equiv.). The tube was capped with a Supelco aluminium crimp seal with septum (PTFE/butyl), then evacuated under high vacuum and backfilled with N_2 (3 times). Degassed $Ru(bpy)_3(PF_6)_2$ in CH_2Cl_2 stock solution (1.0 mL, 0.1 mol%), alkene (if liquid) (0.20 mmol, 2.0 equiv.), and alcohol (0.50 mmol, 5.0 equiv.) were sequentially added. The vial was purged with a stream of N_2 and the lid sealed with parafilm and placed approximately 5 cm from blue LEDs. The blue LEDs were switched on and the mixture was stirred under irradiation at −78 °C for 3 h. The tube was allowed to warm to room temperature, opened, and the reaction mixture was concentrated and purified by column chromatography on silica gel eluting with hexane–EtOAc.

General Procedure for the Photocatalytic 1,2-Alkoxy-Amidation of Alkenes – GP7

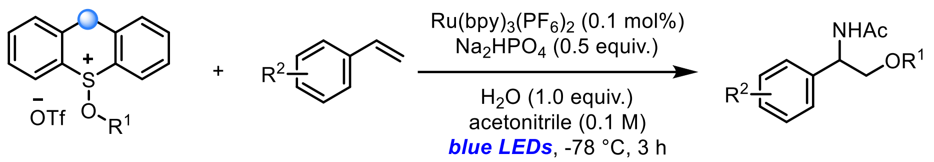

A dry tube equipped with a stirring bar was charged with alkoxy sulfonium salt (0.10 mmol, 1.0 equiv.), Na_2HPO_4 (7 mg, 0.05 mmol, 0.5 equiv.) and alkene (if solid) (0.20 mmol, 2.0 equiv.). The tube was capped with a Supelco aluminium crimp seal with septum (PTFE/butyl), then evacuated under high vacuum and backfilled with N_2 (3 times). Degassed $Ru(bpy)_3(PF_6)_2$ in nitrile stock solution (0.5 mL, 0.1 mol%) and alkene (if liquid) (0.20 mmol, 2.0 equiv.), and H_2O (2 µL, 0.10 mmol, 1.0 equiv.) were sequentially added. The vial was purged with a stream of N_2 and the lid sealed with parafilm and placed approximately 5 cm from blue LEDs. The blue LEDs were switched on and the mixture was stirred under irradiation at −30 °C for 3 h. The tube was allowed to warm to room temperature, opened, and the reaction mixture was concentrated and purified by column chromatography on silica gel eluting with hexane–EtOAc.

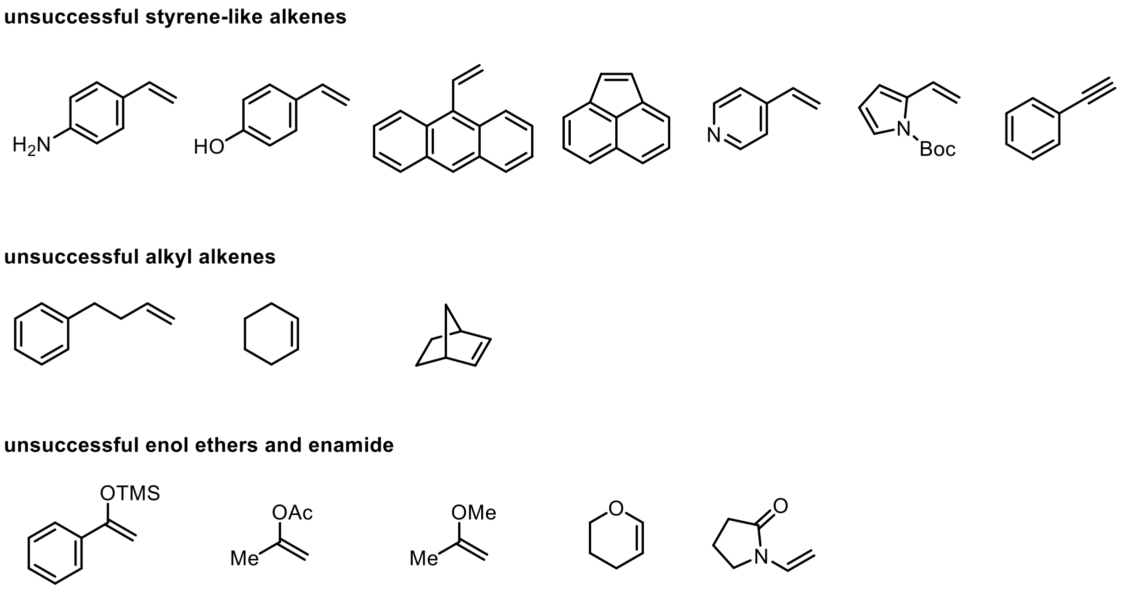

Scheme S1. Currently unsuccessful substrates.

Substrate scope of 1,2-alkoxy-hydroxylation

1-(4-Chlorophenyl)-2-methoxyethan-1-ol (4a)

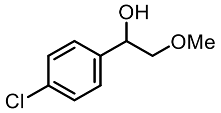

Following **GP5**, 5-methoxy-5*H*-dibenzo[*b*,*d*]thiophen-5-ium triflate **1a** (36 mg, 0.10 mmol) and 4-chlorostyrene (24 µL, 0.20 mmol) gave **4a** (14.9 mg, 80%) as an oil. ^{1}H NMR (400 MHz, $CDCl_3$) δ 7.32 (4H, s), 4.86 (1H, dd, *J* = 8.8, 3.3 Hz), 3.51 (1H, dd, *J* = 9.8, 3.3 Hz), 3.42 (3H, s), 3.38 (1H, dd, *J* = 9.8, 8.8 Hz), 2.88 (1H, br s); ^{13}C NMR (101 MHz, $CDCl_3$) δ 138.9, 133.7, 128.7, 127.6, 78.0, 72.1, 59.2. Data in accordance with the literature.[15]

4-(1-Hydroxy-2-methoxyethyl)phenyl acetate (4b)

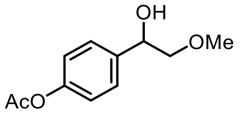

Following **GP5**, 5-methoxy-5*H*-dibenzo[*b*,*d*]thiophen-5-ium triflate **1a** (36 mg, 0.10 mmol) and 4-vinylphenyl acetate (35 mg, 0.20 mmol) gave **4b** (16.8 mg, 80%) as an oil. R*f* 0.26 [hexane–EtOAc (1:1)]; ^{1}H NMR (400 MHz, $CDCl_3$) δ 7.39 (2H, d, *J* = 8.5 Hz), 7.07 (2H, d, *J* = 8.5 Hz), 4.89 (1H, dd, *J* = 9.0, 3.2 Hz), 3.53 (1H, dd, *J* = 9.8, 3.2 Hz), 3.43 (3H, s), 3.40 (1H, dd, *J* = 9.8, 9.0 Hz), 2.79 (1H, br s), 2.29 (3H, s); ^{13}C NMR (101 MHz, $CDCl_3$) δ 169.6, 150.3, 137.9, 127.4, 121.7, 78.2, 72.3, 59.2, 21.3; HRMS (APCI): Found $(M+Na)^+$ 233.0789, $C_{11}H_{14}O_4Na$ requires 233.0784.

2-Methoxy-1-(4-phenoxyphenyl)ethan-1-ol (4c)

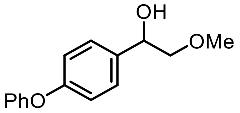

Following **GP5**, 5-methoxy-5*H*-dibenzo[*b*,*d*]thiophen-5-ium triflate **1a** (36 mg, 0.10 mmol) and 4-phenoxystyrene (37 µL, 0.20 mmol) gave **4c** (18.8 mg, 77%) as an oil. R*f* 0.26 [hexane–EtOAc (2:1)]; ^{1}H NMR (400 MHz, $CDCl_3$) δ 7.38–7.28 (4H, m), 7.15–7.06 (1H, m), 7.04–6.95 (4H, m), 4.88 (1H, dd, *J* = 8.9, 3.2 Hz), 3.54 (1H, dd, *J* = 9.7, 3.2 Hz), 3.45 (3H, s), 3.44 (1H, dd, *J* = 9.7, 8.9 Hz), 2.80 (1H, br s); ^{13}C NMR (101 MHz, $CDCl_3$) δ 157.3, 157.1, 135.2, 129.9, 127.8, 123.4, 119.0, 118.9, 78.2, 72.3, 59.2; HRMS (APCI): Found $(M+Na)^+$ 267.0996, $C_{15}H_{16}O_3Na$ requires 267.0992.

2-Methoxy-1-(4-methoxyphenyl)ethan-1-ol (4d)

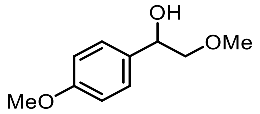

Following **GP5**, 5-methoxy-5*H*-dibenzo[*b*,*d*]thiophen-5-ium triflate **1a** (36 mg, 0.10 mmol) and 4-methoxystyrene (27 µL, 0.20 mmol) gave **4d** (7.1 mg, 39%) as an oil. ^{1}H NMR (400 MHz, $CDCl_3$) δ 7.31 (2H, d, *J* = 8.7 Hz), 6.89 (2H, d, *J* = 8.7 Hz), 4.85 (1H, dd, *J* = 8.7, 3.3 Hz), 3.80 (3H, s), 3.51 (1H, dd, *J* = 9.7, 3.3 Hz), 3.44 (3H, s), 3.43 (1H, dd, *J* = 9.7, 8.7 Hz), 2.68 (1H, br s); ^{13}C NMR (101 MHz, $CDCl_3$) δ 159.4, 132.5, 127.6, 114.0, 78.3, 72.4, 59.2, 55.4. Data in accordance with the literature.[15]

***N*-(4-(1-Hydroxy-2-methoxyethyl)phenyl)-*N*-methylacetamide (4e)**

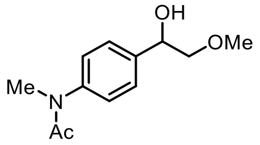

Following **GP5**, 5-methoxy-5*H*-dibenzo[*b*,*d*]thiophen-5-ium triflate **1a** (36 mg, 0.10 mmol) and *N*-methyl-*N*-(4-vinylphenyl)acetamide **3b** (35 mg, 0.20 mmol) gave **4e** (15.0 mg, 67%) as a solid. ^{1}H NMR (400 MHz, $CDCl_3$) δ 7.44 (2H, d, *J* = 8.0 Hz), 7.18 (2H, d, *J* = 8.0 Hz), 4.91 (1H, dd, *J* = 8.8, 3.3 Hz), 3.57 (1H, dd, *J* = 9.7, 3.3 Hz), 3.45 (3H, s), 3.43 (1H, dd, *J* = 9.7, 8.8 Hz), 3.25 (3H, s), 2.90 (1H, br s), 1.88 (3H, s); ^{13}C NMR (101 MHz, $CDCl_3$) δ 171.1, 144.0, 140.2, 127.7, 127.1, 78.0, 72.2, 59.2, 37.4, 22.5. Data in accordance with the literature.[15]

1-(4-(*tert*-Butyl)phenyl)-2-methoxyethan-1-ol (4f)

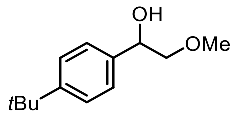

Following **GP5**, 5-methoxy-5*H*-dibenzo[*b*,*d*]thiophen-5-ium triflate **1a** (36 mg, 0.10 mmol) and 4-*tert*-butylstyrene (37 µL, 0.20 mmol) gave **4f** (17.3 mg, 83%) as an oil. ^{1}H NMR (400 MHz, $CDCl_3$) δ 7.38 (2H, d, *J* = 8.4 Hz), 7.31 (2H, d, *J* = 8.4 Hz), 4.87 (1H, dd, *J* = 8.9, 3.3 Hz), 3.55 (1H, dd, *J* = 9.8, 3.3 Hz), 3.47 (1H, dd, *J* = 9.8, 8.9 Hz), 3.44 (3H, s), 2.71 (1H, br s), 1.32 (9H, s); ^{13}C NMR (101 MHz, $CDCl_3$) δ 151.0, 137.4, 126.0, 125.5, 78.3, 72.6, 59.2, 34.7, 31.5. Data in accordance with the literature.[15]

2-Methoxy-1-(*p*-tolyl)ethan-1-ol (4g)

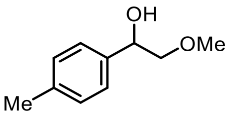

Following **GP5**, 5-methoxy-5*H*-dibenzo[*b*,*d*]thiophen-5-ium triflate **1a** (36 mg, 0.10 mmol) and 4-methylstyrene (26 µL, 0.20 mmol) gave **4g** (13.8 mg, 83%) as an oil. ^{1}H NMR (400 MHz, $CDCl_3$) δ 7.27 (2H, d, *J* = 8.0 Hz), 7.16 (2H, d, *J* = 8.0 Hz), 4.86 (1H, dd, *J* = 8.9, 3.3 Hz), 3.52 (1H, dd, *J* = 9.8, 3.3 Hz), 3.43 (3H, s), 3.43 (1H, dd, *J* = 9.8, 8.9 Hz), 2.71 (1H, br s), 2.34 (3H, s); ^{13}C NMR (101 MHz, $CDCl_3$) δ 137.7, 137.4, 129.2, 126.2, 78.3, 72.6, 59.2, 21.3. Data in accordance with the literature.[15]

1-([1,1'-Biphenyl]-4-yl)-2-methoxyethan-1-ol (4h)

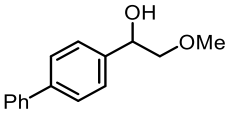

Following **GP5**, 5-methoxy-5*H*-dibenzo[*b*,*d*]thiophen-5-ium triflate **1a** (36 mg, 0.10 mmol) and 4-phenylstyrene (36 mg, 0.20 mmol) gave **4h** (12.8 mg, 56%) as a solid. ^{1}H NMR (400 MHz, $CDCl_3$) δ 7.62–7.57 (4H, m), 7.48–7.41 (4H, m), 7.38–7.32 (1H, m), 4.95 (1H, dd, *J* = 8.9, 3.3 Hz), 3.60 (1H, dd, *J* = 9.8, 3.3 Hz), 3.49 (1H, dd, *J* = 9.8, 8.9 Hz), 3.46 (3H, m), 2.81 (1H, br s); ^{13}C NMR (101 MHz, $CDCl_3$) δ 141.0, 139.4, 128.9, 127.4, 127.3, 127.2, 126.7, 78.2, 72.6, 59.2. Data in accordance with the literature.[15]

2-Methoxy-1-phenylethan-1-ol (4i)

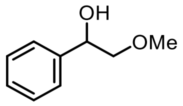

Following **GP5**, 5-methoxy-5*H*-dibenzo[*b*,*d*]thiophen-5-ium triflate **1a** (36 mg, 0.10 mmol) and styrene (23 µL, 0.20 mmol) gave **4i** (12.5 mg, 82%) as an oil. ^{1}H NMR (400 MHz, $CDCl_3$) δ 7.41–7.33 (4H, m), 7.32–7.27 (1H, m), 4.90 (1H, dd, *J* = 8.9, 3.2 Hz), 3.55 (1H, dd, *J* = 9.8, 3.2 Hz), 3.44 (3H, s), 3.44 (1H, dd, *J* = 9.8, 8.9 Hz), 2.75 (1H, br s); ^{13}C NMR (101 MHz, $CDCl_3$) δ 140.3, 128.6, 128.0, 126.3, 78.3, 72.8, 59.2. Data in accordance with the literature.[16]

1-(4-Fluorophenyl)-2-methoxyethan-1-ol (4j)

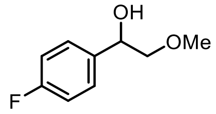

Following **GP5**, 5-methoxy-5*H*-dibenzo[*b*,*d*]thiophen-5-ium triflate **1a** (36 mg, 0.10 mmol) and 4-fluorostyrene (24 μL, 0.20 mmol) gave **4j** (14.0 mg, 82%) as an oil. ^{1}H NMR (400 MHz, $CDCl_3$) δ 7.39–7.32 (2H, m), 7.08–7.00 (2H, m), 4.87 (1H, dd, *J* = 8.9, 3.2 Hz), 3.52 (1H, dd, *J* = 9.8, 3.2 Hz), 3.43 (3H, s), 3.39 (1H, dd, *J* = 9.8, 8.9 Hz), 2.75 (1H, br s); ^{13}C NMR (101 MHz, $CDCl_3$) δ 162.6 (d, $J_{C\text{-}F}$ = 245.8 Hz), 136.1 (d, $J_{C\text{-}F}$ = 3.0 Hz), 128.0 (d, $J_{C\text{-}F}$ = 8.0 Hz), 115.4 (d, $J_{C\text{-}F}$ = 21.3 Hz), 78.2, 72.2, 59.2; ^{19}F NMR (376 MHz, $CDCl_3$) δ –114.7. Data in accordance with the literature.[15]

1-(4-Bromophenyl)-2-methoxyethan-1-ol (4k)

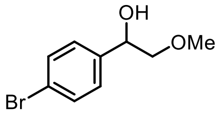

Following **GP5**, 5-methoxy-5*H*-dibenzo[*b*,*d*]thiophen-5-ium triflate **1a** (36 mg, 0.10 mmol) and 4-bromostyrene (26 μL, 0.20 mmol) gave **4k** (18.0 mg, 78%) as an oil. R_f 0.12 [hexane–EtOAc (4:1)]; ^{1}H NMR (500 MHz, $CDCl_3$) δ 7.48 (2H, d, *J* = 8.3 Hz), 7.26 (2H, d, *J* = 8.3 Hz), 4.85 (1H, dd, *J* = 8.8, 3.3 Hz), 3.52 (1H, dd, *J* = 9.8, 3.3 Hz), 3.42 (3H, s), 3.38 (1H, dd, *J* = 9.8, 8.8 Hz), 2.82 (1H, br s); ^{13}C NMR (126 MHz, $CDCl_3$) δ 139.4, 131.6, 128.0, 121.8, 78.0, 72.2, 59.2; HRMS (APCI): Found $(M+Na)^+$ 252.9838, $C_9H_{11}O_2BrNa$ requires 252.9835.

4-(1-Hydroxy-2-methoxyethyl)benzonitrile (4l)

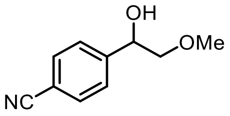

Following **GP5**, 5-methoxy-5*H*-dibenzo[*b*,*d*]thiophen-5-ium triflate **1a** (36 mg, 0.10 mmol) and 4-cyanostyrene (24 μL, 0.20 mmol) gave **4l** (10.6 mg, 60%) as an oil. R_f 0.24 [hexane–EtOAc (4:1)]; ^{1}H NMR (400 MHz, $CDCl_3$) δ 7.64 (2H, d, *J* = 8.2 Hz), 7.50 (2H, d, *J* = 8.2 Hz), 4.93 (1H, dt, *J* = 8.5, 2.7 Hz), 3.56 (1H, dd, *J* = 9.7, 3.4 Hz), 3.43 (3H, s), 3.38 (1H, dd, *J* = 9.7, 8.5 Hz), 2.94 (1H, br d, *J* = 2.7 Hz); ^{13}C NMR (101 MHz, $CDCl_3$) δ 145.8, 132.4, 126.9, 118.9, 111.7, 77.6, 72.1, 59.3; HRMS (APCI): Found $(M+Na)^+$ 200.0688, $C_{10}H_{11}NO_2Na$ requires 200.0682.

2-Methoxy-1-(4-nitrophenyl)ethan-1-ol (4m)

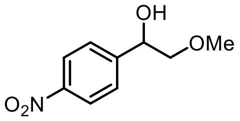

Following **GP5**, 5-methoxy-5*H*-dibenzo[*b*,*d*]thiophen-5-ium triflate **1a** (36 mg, 0.10 mmol) and 4-nitrostyrene (30 mg, 0.20 mmol) gave **4m** (11.2 mg, 57%) as a solid. R_f 0.11 [hexane–EtOAc (2:1)]; ^{1}H NMR (500 MHz, $CDCl_3$) δ 8.21 (2H, d, *J* = 8.6 Hz), 7.57 (2H, d, *J* = 8.6 Hz), 4.99 (1H, dd, *J* = 8.4, 3.4 Hz), 3.59 (1H, dd, *J* = 9.7, 3.4 Hz), 3.44 (3H, s), 3.40 (1H, dd, *J* = 9.7, 8.4 Hz), 2.96 (1H, br s); ^{13}C NMR (126 MHz, $CDCl_3$) δ 147.7 (2 × C), 127.0, 123.8, 77.6, 72.0, 59.3; HRMS (ESI): Found ($M-H^+$) 196.0607, $C_9H_{11}NO_4$ requires 196.0615.

2-Methoxy-1-(3-methoxyphenyl)ethan-1-ol (4n)

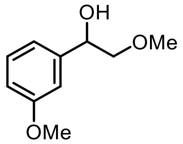

Following **GP5**, 5-methoxy-5*H*-dibenzo[*b*,*d*]thiophen-5-ium triflate **1a** (36 mg, 0.10 mmol) and 3-vinylanisole (28 µL, 0.20 mmol) gave **4n** (14.2 mg, 78%) as an oil. ^{1}H NMR (400 MHz, $CDCl_3$) δ 7.26 (1H, t, *J* = 7.8 Hz), 6.99–6.92 (2H, m), 6.87–6.80 (1H, m), 4.88 (1H, dd, *J* = 8.9, 3.2 Hz), 3.81 (3H, s), 3.55 (1H, dd, *J* = 9.8, 3.2 Hz), 3.43 (3H, s), 3.42 (1H, dd, *J* = 9.8, 8.9 Hz), 2.78 (1H, br s); ^{13}C NMR (101 MHz, $CDCl_3$) δ 159.9, 142.0, 129.6, 118.6, 113.5, 111.7, 78.2, 72.7, 59.2, 55.4. Data in accordance with the literature.[16]

2-Methoxy-1-(*m*-tolyl)ethan-1-ol (4o)

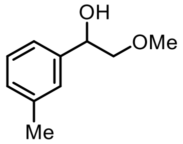

Following **GP5**, 5-methoxy-5*H*-dibenzo[*b*,*d*]thiophen-5-ium triflate **1a** (36 mg, 0.10 mmol) and 3-methylstyrene (26 µL, 0.20 mmol) gave **4o** (12.8 mg, 77%) as an oil. ^{1}H NMR (400 MHz, $CDCl_3$) δ 7.24 (1H, t, *J* = 7.6 Hz), 7.21 (1H, s), 7.17 (1H, d, *J* = 7.7 Hz), 7.11 (1H, d, *J* = 7.3 Hz), 4.86 (1H, dd, *J* = 8.9, 3.2 Hz), 3.54 (1H, dd, *J* = 9.8, 3.2 Hz), 3.44 (3H, s), 3.43 (1H, dd, *J* = 9.8, 8.9 Hz), 2.75 (1H, br s), 2.36 (3H, s); ^{13}C NMR (101 MHz, $CDCl_3$) δ 140.3, 138.2, 128.8, 128.4, 126.9, 123.3, 78.3, 72.8, 59.2, 21.6. Data in accordance with the literature.[15]

1-(3-Chlorophenyl)-2-methoxyethan-1-ol (4p)

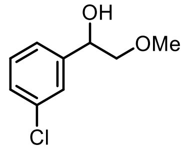

Following **GP5**, 5-methoxy-5*H*-dibenzo[*b*,*d*]thiophen-5-ium triflate **1a** (36 mg, 0.10 mmol) and 3-chlorostyrene (25 μL, 0.20 mmol) gave **4p** (14.7 mg, 79%) as an oil. R_f 0.29 [hexane–EtOAc (2:1)]; ^{1}H NMR (400 MHz, $CDCl_3$) δ 7.40 (1H, s), 7.30–7.23 (3H, m), 4.86 (1H, dd, *J* = 8.8, 3.2 Hz), 3.54 (1H, dd, *J* = 9.8, 3.3 Hz), 3.43 (3H, s), 3.39 (1H, dd, *J* = 9.8, 8.8 Hz), 2.83 (1H, br s); ^{13}C NMR (101 MHz, $CDCl_3$) δ 142.5, 134.5, 129.8, 128.1, 126.5, 124.4, 78.0, 72.2, 59.2; HRMS (APCI): Found $(M+Na)^+$ 209.0342, $C_9H_{11}O_2ClNa$ requires 209.0340.

1-(3-Bromophenyl)-2-methoxyethan-1-ol (4q)

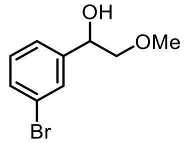

Following **GP5**, 5-methoxy-5*H*-dibenzo[*b*,*d*]thiophen-5-ium triflate **1a** (36 mg, 0.10 mmol) and 3-bromostyrene (26 μL, 0.20 mmol) gave **4q** (18.5 mg, 80%) as an oil. ^{1}H NMR (400 MHz, $CDCl_3$) δ 7.56 (1H, s), 7.42 (1H, d, *J* = 8.1 Hz), 7.30 (1H, d, *J* = 7.8 Hz), 7.22 (1H, t, *J* = 7.8 Hz), 4.86 (1H, dd, *J* = 8.8, 3.3 Hz), 3.54 (1H, dd, *J* = 9.8, 3.3 Hz), 3.43 (3H, s), 3.39 (1H, dd, *J* = 9.8, 8.8 Hz), 2.82 (1H, br s); ^{13}C NMR (101 MHz, $CDCl_3$) δ 142.7, 131.0, 130.1, 129.4, 124.9, 122.7, 78.0, 72.1, 59.2. Data in accordance with the literature.[15]

2-Methoxy-1-(3-(trifluoromethyl)phenyl)ethan-1-ol (4r)

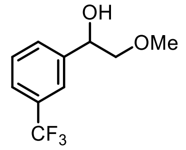

Following **GP5**, 5-methoxy-5*H*-dibenzo[*b*,*d*]thiophen-5-ium triflate **1a** (36 mg, 0.10 mmol) and 3-(trifluoromethyl)styrene (30 μL, 0.20 mmol) gave **4r** (14.5 mg, 66%) as an oil. R_f 0.34 [hexane–EtOAc (2:1)]; ^{1}H NMR (400 MHz, $CDCl_3$) δ 7.67 (1H, s), 7.59–7.51 (2H, m), 7.47 (1H, t, *J* = 7.7 Hz), 4.95 (1H, dt, *J* = 8.6, 2.8 Hz), 3.57 (1H, dd, *J* = 9.8, 3.3 Hz), 3.44 (3H, s), 3.41 (1H, dd, *J* = 9.8, 8.6 Hz), 2.88 (1H, br d, *J* = 2.5 Hz); ^{13}C NMR (101 MHz, $CDCl_3$) δ 141.4, 130.9 (q, $J_{C\text{-}F}$ = 32.1 Hz), 129.7, 129.0, 124.8 (q, $J_{C\text{-}F}$ = 3.8 Hz), 124.3 (q, $J_{C\text{-}F}$ = 272.4 Hz), 123.1 (q, $J_{C\text{-}F}$ = 3.8 Hz), 122.9, 77.9, 72.2, 59.2; ^{19}F NMR (376 MHz, $CDCl_3$) δ −62.6; HRMS (APCI): Found $(M+Na)^+$ 243.0600, $C_{10}H_{11}O_2F_3Na$ requires 243.0603.

2-Methoxy-1-(3-nitrophenyl)ethan-1-ol (4s)

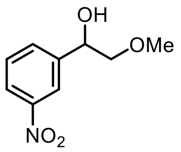

Following **GP5**, 5-methoxy-5*H*-dibenzo[*b*,*d*]thiophen-5-ium triflate **1a** (36 mg, 0.10 mmol) and 3-nitrostyrene (28 μL, 0.20 mmol) gave **4s** (12.4 mg, 63%) as an solid. R_f 0.13 [hexane–EtOAc (2:1)]; ^{1}H NMR (400 MHz, $CDCl_3$) δ 8.27 (1H, s), 8.15 (1H, d, *J* = 8.2 Hz), 7.73 (1H, d, *J* = 8.0 Hz), 7.53 (1H, t, *J* = 7.9 Hz), 4.99 (1H, dt, *J* = 8.4, 2.8 Hz), 3.60 (1H, dd, *J* = 9.7, 3.3 Hz), 3.44 (3H, s), 3.43 (1H, dd, *J* = 9.7, 8.4 Hz), 2.99 (1H, br d, *J* = 2.7 Hz); ^{13}C NMR (101 MHz, $CDCl_3$) δ 148.5, 142.7, 132.4, 129.5, 122.9, 121.4, 77.6, 71.8, 59.3; HRMS (ESI): Found (M−H$^+$) 196.0608, $C_9H_{11}NO_4$ requires 196.0615.

3-(1-Hydroxy-2-methoxyethyl)benzaldehyde (4t)

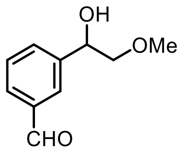

Following **GP5**, 5-methoxy-5*H*-dibenzo[*b*,*d*]thiophen-5-ium triflate **1a** (36 mg, 0.10 mmol) and 3-vinylbenzaldehyde (25 μL, 0.20 mmol) gave **4t** (7.2 mg, 40%) as an oil. R_f 0.13 [hexane–EtOAc (2:1)]; ^{1}H NMR (400 MHz, $CDCl_3$) δ 10.03 (1H, s), 7.92 (1H, s), 7.82 (1H, d, *J* = 7.7 Hz), 7.68 (1H, d, *J* = 7.7 Hz), 7.53 (1H, t, *J* = 7.7 Hz), 4.98 (1H, dd, *J* = 8.7, 3.3 Hz), 3.59 (1H, dd, *J* = 9.8, 3.3 Hz), 3.45 (3H, s), 3.43 (1H, dd, *J* = 9.8, 8.7 Hz), 2.89 (1H, br s); ^{13}C NMR (101 MHz, $CDCl_3$) δ 192.4, 141.6, 136.7, 132.4, 129.3, 129.3, 127.5, 77.9, 72.2, 59.3; HRMS (APCI): Found (M+Na)$^+$ 203.0684, $C_{10}H_{12}O_3Na$ requires 203.0679.

2-Methoxy-1-(2-methoxyphenyl)ethan-1-ol (4u)

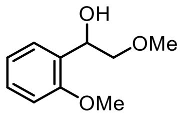

Following **GP5**, 5-methoxy-5*H*-dibenzo[*b*,*d*]thiophen-5-ium triflate **1a** (36 mg, 0.10 mmol) and 2-vinylanisole (27 μL, 0.20 mmol) gave **4u** (13.7 mg, 75%) as an oil. ^{1}H NMR (400 MHz, $CDCl_3$) δ 7.47 (1H, dd, *J* = 7.5, 1.7 Hz), 7.26 (1H, td, *J* = 7.2, 1.7 Hz), 6.98 (1H, t, *J* = 7.5 Hz), 6.87 (1H, d, *J* = 8.3 Hz), 5.21 (1H, dt, *J* = 8.3, 3.4 Hz), 3.84 (3H, s), 3.62 (1H, dd, *J* = 9.8, 3.2 Hz), 3.43 (3H, s), 3.42 (1H, dd, *J* = 9.8, 8.3 Hz), 2.86 (1H, br d, *J* = 3.7 Hz); ^{13}C NMR (101 MHz, $CDCl_3$) δ 156.4, 128.7, 128.6, 127.1, 120.9, 110.4, 76.8, 68.3, 59.0, 55.4. Data in accordance with the literature.[16]

2-Methoxy-1-(*o*-tolyl)ethan-1-ol (4v)

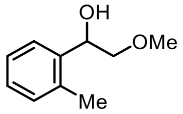

Following **GP5**, 5-methoxy-5*H*-dibenzo[*b*,*d*]thiophen-5-ium triflate **1a** (36 mg, 0.10 mmol) and 2-methylstyrene (26 µL, 0.20 mmol) gave **4v** (14.1 mg, 85%) as an oil. ^{1}H NMR (400 MHz, $CDCl_3$) δ 7.53 (1H, dd, J = 7.4, 1.8 Hz), 7.24 (1H, td, J = 7.4, 1.8 Hz), 7.19 (1H, td, J = 7.2, 1.7 Hz), 7.14 (1H, dd, J = 7.2, 1.7 Hz), 5.14 (1H, dd, J = 9.0, 2.7 Hz), 3.51 (1H, dd, J = 10.0, 2.9 Hz), 3.45 (3H, s), 3.38 (1H, dd, J = 10.0, 9.0 Hz), 2.69 (1H, br s), 2.35 (3H, s); ^{13}C NMR (101 MHz, $CDCl_3$) δ 138.2, 134.9, 130.4, 127.7, 126.4, 126.0, 77.2, 69.5, 59.1, 19.2. Data in accordance with the literature.[16]

1-(2-Fluorophenyl)-2-methoxyethan-1-ol (4w)

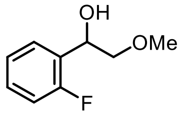

Following **GP5**, 5-methoxy-5*H*-dibenzo[*b*,*d*]thiophen-5-ium triflate **1a** (36 mg, 0.10 mmol) and 2-fluorostyrene (24 µL, 0.20 mmol) gave **4w** (14.0 mg, 82%) as an oil. R_f 0.22 [hexane–EtOAc (4:1)]; ^{1}H NMR (400 MHz, $CDCl_3$) δ 7.56 (1H, td, J = 7.5, 1.8 Hz), 7.30–7.23 (1H, m), 7.17 (1H, td, J = 7.5, 1.2 Hz), 7.02 (1H, ddd, J = 10.5, 8.2, 1.2 Hz), 5.23 (1H, dt, J = 8.4, 3.0 Hz), 3.62 (1H, dd, J = 9.8, 3.1 Hz), 3.44 (3H, s), 3.43 (1H, dd, J = 9.8, 8.4 Hz), 2.78 (1H, br d, J = 2.9 Hz); ^{13}C NMR (101 MHz, $CDCl_3$) δ 159.9 (d, $J_{C\text{-}F}$ = 245.9 Hz), 129.3 (d, $J_{C\text{-}F}$ = 8.3 Hz), 127.8 (d, $J_{C\text{-}F}$ = 4.4 Hz), 127.4 (d, $J_{C\text{-}F}$ = 13.2 Hz), 124.4 (d, $J_{C\text{-}F}$ = 3.4 Hz), 115.3 (d, $J_{C\text{-}F}$ = 21.6 Hz), 76.8 (d, $J_{C\text{-}F}$ = 0.8 Hz), 66.9 (d, J = 2.7 Hz), 59.1; ^{19}F NMR (376 MHz, $CDCl_3$) δ −119.4; HRMS (APCI): Found $(M+Na)^+$ 193.0641, $C_9H_{11}O_2FNa$ requires 193.0635.

1-(2-Chlorophenyl)-2-methoxyethan-1-ol (4x)

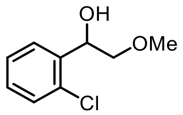

Following **GP5**, 5-methoxy-5*H*-dibenzo[*b*,*d*]thiophen-5-ium triflate **1a** (36 mg, 0.10 mmol) and 2-chlorostyrene (25 µL, 0.20 mmol) gave **4x** (14.9 mg, 80%) as an oil. R_f 0.39 [hexane–EtOAc (2:1)]; ^{1}H NMR (500 MHz, $CDCl_3$) δ 7.63 (1H, dd, J = 7.8, 1.8 Hz), 7.35–7.28 (2H, m), 7.22 (1H, td, J = 7.6, 1.8 Hz), 5.32 (1H, dt, J = 8.6, 2.8 Hz), 3.67 (1H, dd, J = 10.0, 2.8 Hz), 3.45 (3H, s), 3.33 (1H, dd, J = 10.0, 8.6 Hz); ^{13}C NMR (126 MHz, $CDCl_3$) δ 137.8, 132.0,

129.4, 129.0, 127.8, 127.2, 76.3, 69.5, 59.1; HRMS (APCI): Found $(M+Na)^+$ 209.0347, $C_9H_{11}O_2ClNa$ requires 209.0340.

1-(2-Bromophenyl)-2-methoxyethan-1-ol (4y)

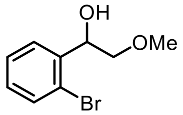

Following **GP5**, 5-methoxy-5*H*-dibenzo[*b*,*d*]thiophen-5-ium triflate **1a** (36 mg, 0.10 mmol) and 2-bromostyrene (25 µL, 0.20 mmol) gave **4y** (19.6 mg, 85%) as an oil. R_f 0.39 [hexane–EtOAc (2:1)]; ^{1}H NMR (400 MHz, $CDCl_3$) δ 7.62 (1H, dd, J = 7.7, 1.8 Hz), 7.52 (1H, dd, J = 8.0, 1.2 Hz), 7.35 (1H, td, J = 7.5, 1.2 Hz), 7.15 (1H, td, J = 7.7, 1.8 Hz), 5.27 (1H, dd, J = 8.6, 2.9 Hz), 3.68 (1H, dd, J = 10.0, 2.9 Hz), 3.46 (3H, s), 3.31 (1H, dd, J = 10.0, 8.6 Hz), 2.88 (1H, br s); ^{13}C NMR (101 MHz, $CDCl_3$) δ 139.3, 132.7, 129.3, 128.1, 127.8, 122.1, 76.3, 71.6, 59.1; HRMS (APCI): Found $(M+Na)^+$ 252.9840, $C_9H_{11}O_2BrNa$ requires 252.9835.

1-(4-(Chloromethyl)phenyl)-2-methoxyethan-1-ol (4z)

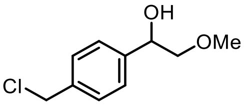

Following **GP5**, 5-methoxy-5*H*-dibenzo[*b*,*d*]thiophen-5-ium triflate **1a** (36 mg, 0.10 mmol) and 4-(chloromethyl)styrene (28 µL, 0.20 mmol) gave **4z** (15.5 mg, 77%) as an oil. R_f 0.13 [hexane–EtOAc (4:1)]; ^{1}H NMR (500 MHz, $CDCl_3$) δ 7.38 (4H, s), 4.90 (1H, dd, J = 9.0, 3.3 Hz), 4.58 (2H, s), 3.54 (1H, dd, J = 9.8, 3.3 Hz), 3.43 (3H, s), 3.41 (1H, dd, J = 9.8, 9.0 Hz), 2.81 (1H, br s); ^{13}C NMR (126 MHz, $CDCl_3$) δ 140.7, 137.2, 128.8, 126.7, 78.1, 72.4, 59.2, 46.1; HRMS (APCI): Found $(M+Na)^+$ 223.0499, $C_{10}H_{13}O_2ClNa$ requires 223.0496.

1-(4-(Hydroxymethyl)phenyl)-2-methoxyethan-1-ol (4aa)

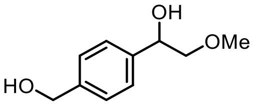

Following **GP5**, 5-methoxy-5*H*-dibenzo[*b*,*d*]thiophen-5-ium triflate **1a** (36 mg, 0.10 mmol) and (4-vinylphenyl)methanol (26 µL, 0.20 mmol) gave **4aa** (8.4 mg, 46%) as an oil. R_f 0.13 [hexane–EtOAc (1:2)]; ^{1}H NMR (400 MHz, $CDCl_3$) δ 7.38 (2H, d, J = 8.4 Hz), 7.35 (2H, d, J = 8.4 Hz), 4.89 (1H, dd, J = 8.9, 3.3 Hz), 4.68 (2H, s), 3.54 (1H, dd, J = 9.8, 3.3 Hz), 3.43 (3H, s), 3.42 (1H, dd, J = 9.8, 8.9 Hz), 2.79 (1H, br s), 1.73 (1H, br s); ^{13}C NMR (101 MHz, $CDCl_3$)

δ 140.6, 139.8, 127.2, 126.5, 78.2, 72.6, 65.2, 59.2; HRMS (APCI): Found $(M+Na)^+$ 205.0839, $C_{10}H_{14}O_3Na$ requires 205.0835.

4-(1-Hydroxy-2-methoxyethyl)benzyl acetate (4ab)

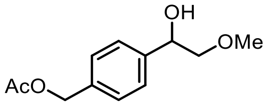

Following **GP5**, 5-methoxy-5*H*-dibenzo[*b*,*d*]thiophen-5-ium triflate **1a** (36 mg, 0.10 mmol) and 4-vinylbenzyl acetate **3c** (35 mg, 0.20 mmol) gave **4ab** (17.9 mg, 80%) as an oil. R_f 0.29 [hexane–EtOAc (1:1)]; ^{1}H NMR (400 MHz, $CDCl_3$) δ 7.38 (2H, d, *J* = 8.2 Hz), 7.34 (2H, d, *J* = 8.2 Hz), 5.09 (2H, s), 4.89 (1H, dd, *J* = 9.3, 3.2 Hz), 3.53 (1H, dd, *J* = 9.8, 3.2 Hz), 3.43 (3H, s), 3.41 (1H, dd, *J* = 9.8, 9.3 Hz), 2.83 (1H, br s), 2.09 (3H, s); ^{13}C NMR (101 MHz, $CDCl_3$) δ 171.0, 140.5, 135.6, 128.5, 126.5, 78.2, 72.5, 66.2, 59.2, 21.1; HRMS (APCI): Found $(M+Na)^+$ 247.0944, $C_{12}H_{16}O_4Na$ requires 247.0941.

4-(1-Hydroxy-2-methoxyethyl)benzyl acetate (4ac)

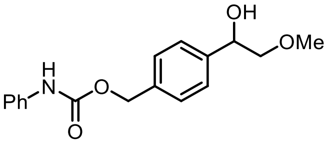

Following **GP5**, 5-methoxy-5*H*-dibenzo[*b*,*d*]thiophen-5-ium triflate **1a** (36 mg, 0.10 mmol) and 4-vinylbenzyl phenylcarbamate **3d** (51 mg, 0.20 mmol) gave **4ac** (24.7 mg, 82%) as a solid. R_f 0.22 [hexane–EtOAc (1:1)]; ^{1}H NMR (500 MHz, $CDCl_3$) δ 7.41–7.34 (6H, m), 7.33–7.27 (2H, m), 7.10–7.03 (1H, m), 6.78 (1H, br s), 5.18 (2H, s), 4.90 (1H, dd, *J* = 8.3, 3.3 Hz), 3.53 (1H, dd, *J* = 9.8, 3.3 Hz), 3.43 (3H, s), 3.42 (1H, dd, *J* = 9.8, 8.3 Hz), 2.86 (1H, br s); ^{13}C NMR (126 MHz, $CDCl_3$) δ 153.4, 140.6, 137.9, 135.8, 129.2, 128.5, 126.5, 123.7, 118.8, 78.1, 72.5, 66.8, 59.2; HRMS (APCI): Found $(M+Na)^+$ 324.1212, $C_{17}H_{19}NO_4Na$ requires 324.1217.

1-(2,6-Dichlorophenyl)-2-methoxyethan-1-ol (4ad)

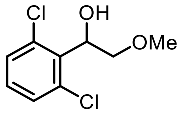

Following **GP5**, 5-methoxy-5*H*-dibenzo[*b*,*d*]thiophen-5-ium triflate **1a** (36 mg, 0.10 mmol) and 2,6-dichlorostyrene (27 μL, 0.20 mmol) gave **4ad** (9.5 mg, 43%) as an oil. R_f 0.32 [hexane–EtOAc (4:1)]; ^{1}H NMR (400 MHz, $CDCl_3$) δ 7.31 (2H, d, *J* = 8.0 Hz), 7.16 (1H, dd, *J* = 8.6, 7.5 Hz), 5.68 (1H, ddd, *J* = 8.7, 7.8, 4.2 Hz), 3.98 (1H, dd, *J* = 10.3, 8.7 Hz), 3.60 (1H, dd, *J* = 10.3, 4.2 Hz), 3.45 (3H, s), 3.00 (1H, d, *J* = 7.8 Hz); ^{13}C NMR (101 MHz, $CDCl_3$) δ 135.1,

134.8, 129.6, 129.5, 74.1, 71.0, 59.2; HRMS (APCI): Found $(M+Na)^+$ 242.9956, $C_9H_{10}O_2Cl_2Na$ requires 242.9950.

1-(2,4-Dimethylphenyl)-2-methoxyethan-1-ol (4ae)

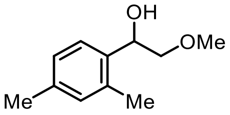

Following **GP5**, 5-methoxy-5*H*-dibenzo[*b*,*d*]thiophen-5-ium triflate **1a** (36 mg, 0.10 mmol) and 2,4-dimethylstyrene (29 µL, 0.20 mmol) gave **4ae** (14.8 mg, 82%) as an oil. R_f 0.32 [hexane–EtOAc (2:1)]; ^{1}H NMR (400 MHz, $CDCl_3$) δ 7.40 (1H, d, *J* = 7.9 Hz), 7.04 (1H, d, *J* = 7.9 Hz), 6.96 (1H, s), 5.10 (1H, dd, *J* = 9.0, 3.0 Hz), 3.49 (1H, dd, *J* = 10.0, 3.0 Hz), 3.44 (3H, s), 3.38 (1H, dd, *J* = 10.0, 9.0 Hz), 2.65 (1H, br s), 2.31 (3H, s), 2.30 (3H, s); ^{13}C NMR (101 MHz, $CDCl_3$) δ 137.4, 135.3, 134.8, 131.2, 127.1, 125.9, 77.3, 69.4, 59.1, 21.1, 19.1; HRMS (APCI): Found $(M+Na)^+$ 203.1046, $C_{11}H_{16}O_2Na$ requires 203.1043.

1-(2,5-Dimethylphenyl)-2-methoxyethan-1-ol (4af)

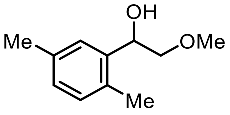

Following **GP5**, 5-methoxy-5*H*-dibenzo[*b*,*d*]thiophen-5-ium triflate **1a** (36 mg, 0.10 mmol) and 2,5-dimethylstyrene (29 µL, 0.20 mmol) gave **4af** (14.4 mg, 80%) as an oil. R_f 0.13 [hexane–EtOAc (4:1)]; ^{1}H NMR (400 MHz, $CDCl_3$) δ 7.34 (1H, s), 7.06–6.96 (2H, m), 5.11 (1H, dt, *J* = 9.2, 2.3 Hz), 3.50 (1H, dd, *J* = 10.0, 2.9 Hz), 3.45 (3H, s), 3.38 (1H, t, *J* = 9.5 Hz), 2.71 (1H, br d, *J* = 2.0 Hz), 2.33 (3H, s), 2.30 (3H, s); ^{13}C NMR (101 MHz, $CDCl_3$) δ 137.9, 135.9, 131.7, 130.4, 128.5, 126.6, 77.3, 69.5, 59.1, 21.2, 18.7 HRMS (APCI): Found $(M+Na)^+$ 203.1047, $C_{11}H_{16}O_2Na$ requires 203.1043.

1-Mesityl-2-methoxyethan-1-ol (4ag)

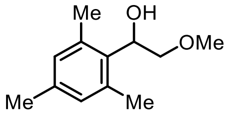

Following **GP5**, 5-methoxy-5*H*-dibenzo[*b*,*d*]thiophen-5-ium triflate **1a** (36 mg, 0.10 mmol) and 2,4,6-trimethylstyrene (32 µL, 0.20 mmol) gave **4ag** (9.3 mg, 48%) as an oil. R_f 0.34 [hexane–EtOAc (3:1)]; ^{1}H NMR (400 MHz, $CDCl_3$) δ 6.82 (2H, s), 5.34 (1H, dd, *J* = 9.8, 2.6 Hz), 3.76 (1H, t, *J* = 9.9 Hz), 3.45 (3H, s), 3.40 (1H, dd, *J* = 10.1, 3.2 Hz), 2.41 (1H, br s), 2.41

(6H, s), 2.24 (3H, s); ^{13}C NMR (101 MHz, $CDCl_3$) δ 137.2, 136.9, 132.4, 130.3, 75.0, 70.7, 59.1, 20.9 (2 × C); HRMS (APCI): Found $(M+Na)^+$ 217.1204, $C_{12}H_{18}O_2Na$ requires 217.1199.

2-Methoxy-1-(perfluorophenyl)ethan-1-ol (4ah)

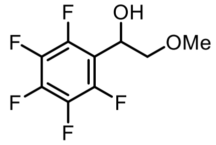

Following **GP5**, 5-methoxy-5*H*-dibenzo[*b*,*d*]thiophen-5-ium triflate **1a** (36 mg, 0.10 mmol) and 2,3,4,5,6-pentafluorostyrene (28 µL, 0.20 mmol) gave **4ah** (15.7 mg, 65%) as a solid. R_f 0.30 [hexane–EtOAc (4:1)]; ^1H NMR ($CDCl_3$, 400 MHz) δ 5.25 (1H, ddd, J = 7.2, 5.8, 4.2 Hz), 3.81–3.71 (1H, m), 3.61 (1H, dd, J = 9.7, 4.2 Hz), 3.43 (3H, s), 2.83 (1H, br d, J = 5.8 Hz); ^{13}C NMR (101 MHz, $CDCl_3$) δ 147.3–146.0 (m), 144.7–143.0 (m), 142.7–139.3 (m), 139.1–138.7 (m), 137.3–136.0 (m), 114.6–113.0 (m), 74.8, 65.4, 59.4; ^{19}F NMR ($CDCl_3$, 376 MHz) δ −142.6 (m), −154.4 (m), −161.8 (m); HRMS (ESI): Found $(M-H^+)$ 241.0289, $C_9H_6F_5O_2$ requires 241.0293.

1-(Bicyclo[4.2.0]octa-1,3,5-trien-3-yl)-2-methoxyethan-1-ol (4ai)

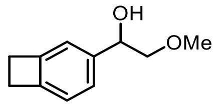

Following **GP5**, 5-methoxy-5*H*-dibenzo[*b*,*d*]thiophen-5-ium triflate **1a** (36 mg, 0.10 mmol) and 3-vinylbicyclo[4.2.0]octa-1,3,5-triene (27 µL, 0.20 mmol) gave **4ai** (14.4 mg, 81%) as an oil. R_f 0.29 [hexane–EtOAc (2:1)]; ^1H NMR (400 MHz, $CDCl_3$) δ 7.20 (1H, d, J = 7.6 Hz), 7.10 (1H, s), 7.03 (1H, d, J = 7.6 Hz), 4.85 (1H, dd, J = 8.9, 3.3 Hz), 3.51 (1H, dd, J = 9.8, 3.3 Hz), 3.43 (3H, s), 3.40 (1H, dd, J = 9.8, 8.9 Hz), 3.16 (4H, s), 2.70 (1H, br s); ^{13}C NMR (101 MHz, $CDCl_3$) δ 146.1, 145.7, 139.0, 125.0, 122.6, 120.6, 78.6, 73.4, 59.1, 29.6, 29.5; HRMS (APCI): Found $(M+Na)^+$ 201.0890, $C_{11}H_{14}O_2Na$ requires 201.0886.

2-Methoxy-1-(naphthalen-2-yl)ethan-1-ol (4aj)

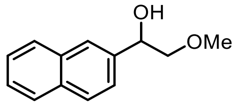

Following **GP5**, 5-methoxy-5*H*-dibenzo[*b*,*d*]thiophen-5-ium triflate **1a** (36 mg, 0.10 mmol) and 2-vinylnaphthalene (27 µL, 0.20 mmol) gave **4aj** (14.4 mg, 71%) as a solid. ^1H NMR (400 MHz, $CDCl_3$) δ 7.87 (1H, s), 7.86–7.81 (3H, m), 7.53–7.44 (3H, m), 5.07 (1H, dd, J = 8.8, 3.2 Hz), 3.64 (1H, dd, J = 9.8, 3.2 Hz), 3.52 (1H, dd, J = 9.8, 8.8 Hz), 3.46 (3H, s), 2.92 (1H, br

s); ^{13}C NMR (101 MHz, $CDCl_3$) δ 137.8, 133.4, 133.2, 128.3, 128.1, 127.8, 126.3, 126.1, 125.2, 124.2, 78.2, 72.9, 59.2. Data in accordance with the literature.[15]

2-Methoxy-1-(naphthalen-1-yl)ethan-1-ol (4ak)

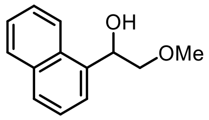

Following **GP5**, 5-methoxy-5*H*-dibenzo[*b*,*d*]thiophen-5-ium triflate **1a** (36 mg, 0.10 mmol) and 1-vinylnaphthalene (31 mg, 0.20 mmol) gave **4ak** (9.7 mg, 48%) as a solid. R_f 0.24 [hexane–EtOAc (2:1)]; ^1H NMR (400 MHz, $CDCl_3$) δ 8.07 (1H, d, J = 8.3 Hz), 7.88 (1H, dd, J = 7.6, 1.8 Hz), 7.80 (1H, d, J = 8.2 Hz), 7.75 (1H, d, J = 7.1 Hz), 7.57–7.45 (3H, m), 5.72 (1H, dd, J = 8.9, 2.8 Hz), 3.78 (1H, dd, J = 10.0, 2.8 Hz), 3.54 (1H, dd, J = 10.0, 8.9 Hz), 3.48 (3H, s), 2.93 (1H, br s); ^{13}C NMR (101 MHz, $CDCl_3$) δ 135.8, 133.8, 130.6, 129.1, 128.4, 126.3, 125.7, 123.8, 122.8, 77.7, 77.5, 69.8, 59.2; HRMS (APCI): Found $(M+Na)^+$ 225.0890, $C_{13}H_{14}O_2Na$ requires 225.0886. Data in accordance with the literature.[17]

1-(Benzo[*b*]thiophen-6-yl)-2-methoxyethan-1-ol (4al)

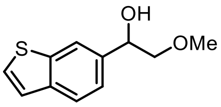

Following **GP5**, 5-methoxy-5*H*-dibenzo[*b*,*d*]thiophen-5-ium triflate **1a** (36 mg, 0.10 mmol) and 6-vinylbenzo[*b*]thiophene **3e** (32 mg, 0.20 mmol) gave **4al** (11.6 mg, 56%) as an oil. R_f 0.27 [hexane-EtOAc (1:1)]; ^1H NMR (400 MHz, $CDCl_3$) δ 7.87 (1H, s), 7.86 (1H, d, J = 8.2 Hz), 7.46 (1H, d, J = 5.4 Hz), 7.36 (1H, dd, J = 8.3, 1.7 Hz), 7.33 (1H, d, J = 5.4 Hz), 5.03 (1H, dd, J = 8.8, 3.3 Hz), 3.60 (1H, dd, J = 9.8, 3.3 Hz), 3.51 (1H, dd, J = 9.8, 8.8 Hz), 3.46 (3H, s); ^{13}C NMR (101 MHz, $CDCl_3$) δ 139.9, 139.4, 136.7, 127.1, 124.0, 122.7, 122.6, 121.3, 78.5, 72.9, 59.2; HRMS (APCI): Found $(M+Na)^+$ 231.0451, $C_{11}H_{12}O_2NaS$ requires 231.0450.

1-(Dibenzo[*b*,*d*]thiophen-2-yl)-2-methoxyethan-1-ol (4am)

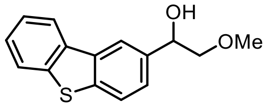

Following **GP5**, 5-methoxy-5*H*-dibenzo[*b*,*d*]thiophen-5-ium triflate **1a** (36 mg, 0.10 mmol) and 2-vinyldibenzo[*b*,*d*]thiophene **3f** (42 mg, 0.20 mmol) gave **4am** (9.6 mg, 37%) as an oil. R_f 0.24 [hexane-EtOAc (1:1)]; ^1H NMR (400 MHz, $CDCl_3$) δ 8.21 (1H, s), 8.20–8.17 (1H, m), 7.87–7.81 (2H, m), 7.49–7.43 (3H, m), 5.09 (1H, dd, J = 8.9, 3.3 Hz), 3.64 (1H, dd, J = 9.8,

3.3 Hz), 3.52 (1H, dd, J = 9.8, 8.9 Hz), 3.48 (3H, s); ^{13}C NMR (101 MHz, $CDCl_3$) δ 140.0, 139.1, 136.9, 135.5, 127.0, 125.1, 124.5, 123.0, 122.9, 121.8, 119.4, 78.5, 72.9, 59.3, 31.1; IR (neat, cm^{-1}): 3427, 3059, 2923, 2853, 1715, 1591, 1468, 1430, 1391, 1316, 1268, 1229, 1193, 1176, 1156, 1117; HRMS (APCI): Found $(M+Na)^+$ 281.0612, $C_{15}H_{14}O_2NaS$ requires 281.0607.

1-(5-Bromothiophen-2-yl)-2-methoxyethan-1-ol (4an)

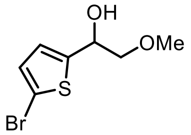

Following **GP3**, 5-methoxy-5*H*-dibenzo[*b*,*d*]thiophen-5-ium triflate **1a** (36 mg, 0.10 mmol) and 2-bromo-5-vinylthiophene **3g** (38 mg, 0.20 mmol) gave **4an** (7.1 mg, 30%) as an oil. R$_f$ 0.32 [hexane-EtOAc (4:1)]; ^{1}H NMR (400 MHz, $CDCl_3$) δ 6.92 (1H, d, J = 3.8 Hz), 6.76 (1H, d, J = 3.8 Hz), 5.02 (1H, dd, J = 7.6, 3.5 Hz), 3.61 (1H, dd, J = 9.7, 3.5 Hz), 3.51 (1H, dd, J = 9.7, 7.6 Hz), 3.44 (3H, s); ^{13}C NMR (101 MHz, $CDCl_3$) δ 145.6, 129.5, 124.8, 112.0, 77.3, 69.3, 59.3. HRMS (ESI): Found $(M-2H+Na)^+$ $C_7H_7BrO_2SNa$, 256.9244 requires 256.9242.

1-(Benzofuran-2-yl)-2-methoxyethan-1-ol (4ao)

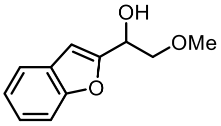

Following **GP5**, 5-methoxy-5*H*-dibenzo[*b*,*d*]thiophen-5-ium triflate **1a** (36 mg, 0.10 mmol) and 2-vinylbenzofuran (29 mg, 0.20 mmol) gave **4ao** (8.6 mg, 45%) as an oil. ^{1}H NMR (400 MHz, $CDCl_3$) δ 7.55 (1H, dd, J = 7.2, 1.3 Hz), 7.46 (1H, dd, J = 7.9, 1.0 Hz), 7.27 (1H, td, J = 7.7, 1.4 Hz), 7.22 (1H, td, J = 7.4, 1.4 Hz), 6.72 (1H, s), 5.03 (1H, q, J = 5.0 Hz), 3.81–3.74 (2H, m), 3.46 (3H, s), 2.84 (1H, br d, J = 4.7 Hz); ^{13}C NMR (101 MHz, $CDCl_3$) δ 156.4, 154.9, 128.2, 124.4, 122.9, 121.2, 111.4, 103.8, 74.9, 67.4, 59.4. Data in accordance with the literature.[15]

2-(Benzo[*b*]thiophen-2-yl)-1-methoxypropan-2-ol (4ap)

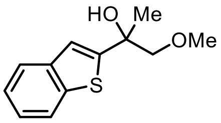

Following **GP5**, 5-methoxy-5*H*-dibenzo[*b*,*d*]thiophen-5-ium triflate **1a** (36 mg, 0.10 mmol) and 2-(prop-1-en-2-yl)benzo[*b*]thiophene **3h** (34.8 mg, 0.20 mmol) gave **4ap** (7.8 mg, 35%) as

an oil. R_f 0.21 [hexane-EtOAc (4:1)]; ^{1}H NMR (400 MHz, $CDCl_3$) δ 7.80 (1H, dd, *J* = 7.7, 1.5 Hz), 7.71 (1H, dd, *J* = 7.4, 1.7 Hz), 7.32 (1H, td, *J* = 7.5, 1.4 Hz), 7.28 (1H, td, *J* = 7.7, 1.5 Hz), 7.20 (1H, s), 3.69 (1H, d, *J* = 9.2 Hz), 3.56 (1H, d, *J* = 9.3 Hz), 3.43 (3H, s), 1.65 (3H, s); ^{13}C NMR (101 MHz, $CDCl_3$) δ 151.3, 140.0, 139.5, 124.3, 124.1, 123.5, 122.4, 119.5, 80.8, 73.5, 59.6, 27.3; IR (neat, cm^{-1}): 3432, 3056, 2978, 2926, 2890, 2828, 1710, 1666, 1456, 1435, 1376, 1329, 1305, 1273, 1250, 1181, 1151, 1105; HRMS (APCI): Found $(M+Na)^+$ 245.0607, $C_{12}H_{14}O_2NaS$ requires 245.0607.

1-Methoxy-2-(pyridin-2-yl)propan-2-ol (4aq)

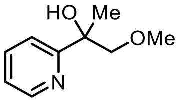

Following **GP5**, 5-methoxy-5*H*-dibenzo[*b*,*d*]thiophen-5-ium triflate **1a** (36 mg, 0.10 mmol) and 2-(prop-1-en-2-yl)pyridine **3i** (23.8 mg, 0.20 mmol) gave **4aq** (5.0 mg, 30%) as an oil. R_f 0.20 [hexane-EtOAc (4:1)]; ^{1}H NMR (400 MHz, $CDCl_3$) δ 8.55 (1H, d, *J* = 4.5 Hz), 7.74 (1H, td, *J* = 7.7, 1.8 Hz), 7.53 (1H, d, *J* = 8.0 Hz), 7.23 (1H, ddd, *J* = 7.6, 5.0, 1.2 Hz), 3.71 (1H, d, *J* = 9.3 Hz), 3.51 (1H, d, *J* = 9.3 Hz), 3.33 (3H, s), 1.53 (3H, s); ^{13}C NMR (101 MHz, $CDCl_3$) δ 163.4, 147.4, 137.4, 122.4, 120.3, 80.4, 74.1, 59.7, 25.6; HRMS (APCI): Found $(M+H)^+$ 168.1018, $C_9H_{14}O_2N$ requires 168.1019.

2-Methoxy-1-phenylpropan-1-ol (4ar)

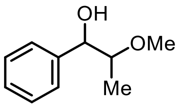

Following **GP5**, 5-methoxy-5*H*-dibenzo[*b*,*d*]thiophen-5-ium triflate **1a** (36 mg, 0.10 mmol) and *trans*-β-methylstyrene (26 μL, 0.20 mmol) gave **4ar** (13.8 mg, 83%, d.r. = 1.3:1) as an inseparable mixture. ^{1}H NMR (400 MHz, $CDCl_3$) δ 7.38–7.25 (5H, m), 4.91 (0.44H, d, *J* = 3.6 Hz), 4.40 (0.58H, d, *J* = 7.9 Hz), 3.54 (0.44H, qd, *J* = 6.3, 3.6 Hz), 3.43 (1.70H, s), 3.42 (1.30H, s), 3.40–3.33 (0.63H, m), 3.25 (0.58H, br s), 2.53 (0.44 H, br s), 0.98 (3H, d, *J* = 6.3 Hz); ^{13}C NMR (101 MHz, $CDCl_3$) δ 140.6 (2 × C), 128.5, 128.3, 128.1, 127.4, 126.4, 81.9, 80.9, 78.5, 74.6, 56.8 (2 × C), 14.8, 12.7. Data in accordance with the literature.[16]

3-Bromo-2-methoxy-1-phenylpropan-1-ol (4as)

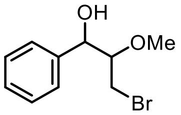

Following **GP5**, 5-methoxy-5*H*-dibenzo[*b*,*d*]thiophen-5-ium triflate **1a** (36 mg, 0.10 mmol) and 3-bromo-1-phenyl-1-propene (39 mg, 0.20 mmol) gave **4as** (19.6 mg, 80%, d.r. = 1.1:1) as an inseparable mixture. R_f 0.26 [hexane–EtOAc (4:1)]; ^{1}H NMR (400 MHz, $CDCl_3$) δ 7.44–7.31 (5H, m), 4.91 (0.47H, dd, *J* = 5.2, 3.2 Hz), 4.83 (0.53H, dd, *J* = 6.4, 3.4 Hz), 3.60–3.52 (1.53H, m), 3.47 (1.60H, s), 3.46–3.43 (0.47H, m), 3.41 (1.40H, s), 3.41–3.34 (0.47H, m), 3.18 (0.53H, dd, *J* = 11.1, 3.8 Hz), 2.87 (0.53 H, br d, *J* = 3.7 Hz), 2.41 (0.47H, br d, *J* = 3.6 Hz); ^{13}C NMR (101 MHz, $CDCl_3$) δ 140.1, 140.0, 128.7, 128.6, 128.4, 128.2, 126.9, 126.7, 84.7, 84.3, 74.5, 73.6, 59.0, 58.7, 31.9, 30.9; HRMS (APCI): Found $(M+Na)^+$ 266.9996, $C_{10}H_{13}O_2BrNa$ requires 266.9991.

Ethyl 3-hydroxy-2-methoxy-3-phenylpropanoate (4at)

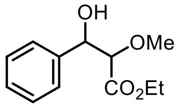

Following **GP5**, 5-methoxy-5*H*-dibenzo[*b*,*d*]thiophen-5-ium triflate **1a** (36 mg, 0.10 mmol) and ethyl cinnamate (24 µL, 0.20 mmol) gave **4at** (13.9 mg, 62%, d.r. = 1:1) as an inseparable mixture. R_f 0.32 [hexane–EtOAc (2:1)]; ^{1}H NMR (400 MHz, $CDCl_3$) δ 7.39–7.28 (5H, m), 5.00 (0.5H, t, *J* = 5.2 Hz), 4.89 (0.5H, t, *J* = 5.2 Hz), 4.18–4.03 (2H, m), 3.98 (0.5H, d, *J* = 5.6 Hz), 3.88 (0.5H, d, *J* = 5.9 Hz), 3.43 (1.5H, s), 3.40 (1.5H, s), 3.00 (0.5H, br d, *J* = 4.7 Hz), 2.93 (0.5H, br d, *J* = 5.1 Hz), 1.15 (1.5H, t, *J* = 7.2 Hz), 1.10 (1.5H, t, *J* = 7.2 Hz); ^{13}C NMR (101 MHz, $CDCl_3$) δ 170.4 (2 × C), 139.5, 139.1, 128.5, 128.4, 128.2, 126.8, 126.7, 85.5, 84.5, 75.0, 74.2, 61.2, 61.2, 59.0, 58.9, 14.2, 14.1; HRMS (APCI): Found $(M+Na)^+$ 247.0946, $C_{12}H_{16}O_4Na$ requires 247.0941. Data in accordance with the literature.[18]

4-Hydroxy-3-methoxy-4-phenylbutan-2-one (4au)

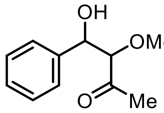

Following **GP5**, 5-methoxy-5*H*-dibenzo[*b*,*d*]thiophen-5-ium triflate **1a** (36 mg, 0.10 mmol) and 4-phenyl-3-buten-2-one (29 mg, 0.20 mmol) gave **4au** (10.9 mg, 56%, d.r. = 2:1) as an inseparable mixture. R_f 0.29 [hexane–EtOAc (2:1)]; ^{1}H NMR (500 MHz, $CDCl_3$) δ 7.40–7.27 (5H, m), 4.90 (0.33H, dd, *J* = 6.2, 4.2 Hz), 4.88 (0.67H, t, *J* = 5.2 Hz), 3.77 (0.67H, d, *J* = 4.8

Hz), 3.77 (0.33H, d, J = 6.2 Hz), 3.35 (2H, s), 3.30 (1H, s), 2.95 (0.67H, br d, J = 5.8 Hz), 2.89 (0.33H, br d, J = 4.2 Hz), 2.08 (2H, s), 2.06 (1H, s); ^{13}C NMR (126 MHz, $CDCl_3$) δ 210.2, 210.0, 139.7, 139.6, 128.6, 128.5, 128.3 (2 × C), 126.8, 126.5, 91.0, 90.3, 74.4, 74.3, 59.5, 59.4, 27.9, 27.5; HRMS (APCI): Found $(M+Na)^+$ 217.0840, $C_{11}H_{14}O_3Na$ requires 217.0835. Data in accordance with the literature.[19]

2-Methoxy-2-methyl-1-phenylpropan-1-ol (4av)

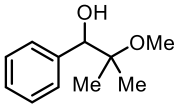

Following **GP3**, 5-methoxy-5*H*-dibenzo[*b*,*d*]thiophen-5-ium triflate **1a** (36 mg, 0.10 mmol) and (2-methylprop-1-en-1-yl)benzene **3j** (26.4 mg, 0.20 mmol) gave **4av** (6.7 mg, 37%) as an oil. ^{1}H NMR (400 MHz, $CDCl_3$) δ 7.41–7.36 (2H, m), 7.36–7.26 (3H, m), 4.62 (1H, d, J = 2.0 Hz), 3.31 (3H, s), 3.08 (1H, d, J = 2.0 Hz), 1.13 (3H, s), 1.03 (3H, s); ^{13}C NMR (101 MHz, $CDCl_3$) δ 140.1, 127.9, 127.8, 127.7, 79.3, 78.3, 49.6, 21.2, 18.7. Data in accordance with literature.[16]

2-Methoxy-2,3-dihydro-1*H*-inden-1-ol (4aw)

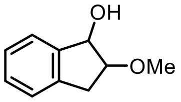

Following **GP5**, 5-methoxy-5*H*-dibenzo[*b*,*d*]thiophen-5-ium triflate **1a** (36 mg, 0.10 mmol) and indene (23 μL, 0.20 mmol) gave **4aw** (12.6 mg, 77%, d.r. = 2.8:1) as an inseparable mixture. ^{1}H NMR (500 MHz, $CDCl_3$) δ 7.48–7.35 (1H, m), 7.30–7.17 (3H, m), 5.12 (0.26H, d, J = 5.6 Hz), 5.05 (0.74H, d, J = 4.8 Hz), 4.09 (0.74H, td, J = 5.4, 4.1 Hz), 4.00 (0.26H, td, J = 7.2, 5.6 Hz), 3.52 (0.78H, s), 3.48 (2.22H, s), 3.29 (0.26H, dd, J = 15.6, 7.2 Hz), 3.07 (0.74H, dd, J = 16.0, 4.1 Hz), 3.00 (0.74H, dd, J = 16.0, 5.5 Hz), 2.90 (0.74H, br d, J = 6.9 Hz), 2.78 (0.26H, dd, J = 15.6, 7.2 Hz), 2.30 (0.24H, br s); ^{13}C NMR (126 MHz, $CDCl_3$) δ 142.8, 142.1, 139.8, 139.0, 128.7 (2 × C), 127.4, 127.3, 125.3, 125.1 (2 × C), 124.2, 90.4, 82.5, 80.5, 75.0, 57.8, 57.6, 35.5, 35.1. Data in accordance with the literature.[15]

2-Methoxy-1,2,3,4-tetrahydronaphthalen-1-ol (4ax)

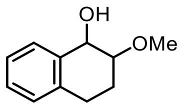

Following **GP5**, 5-methoxy-5*H*-dibenzo[*b*,*d*]thiophen-5-ium triflate **1a** (36 mg, 0.10 mmol) and 1,2-dihydronaphthalene (26 µL, 0.20 mmol) gave **4ax** (9.1 mg, 51%, d.r. = 1.3:1) as an inseparable mixture. ^{1}H NMR (400 MHz, $CDCl_3$) δ 7.58–7.46 (1H, m), 7.25–7.16 (2H, m), 7.13–7.07 (1H, m), 4.78 (0.56H, d, *J* = 3.7 Hz), 4.67 (0.44H, d, *J* = 7.3 Hz), 3.66 (0.56H, dt, *J* = 9.0, 3.0 Hz), 3.51–3.48 (3H, m), 3.45 (0.44H, ddd, *J* = 10.3, 7.3, 3.3 Hz), 3.01–2.70 (2H, m), 2.66 (1H, br s), 2.27–2.10 (1H, m), 1.90 (0.56H, dtd, *J* = 13.0, 6.3, 2.7 Hz), 1.75 (0.44H, dtd, *J* = 13.0, 9.9, 6.0 Hz); ^{13}C NMR (101 MHz, $CDCl_3$) δ 136.8, 136.7, 136.3, 135.7, 129.8, 128.5, 128.3, 127.9, 127.8, 127.5, 126.5, 126.4, 82.1, 78.5, 72.6, 68.5, 56.8, 56.5, 27.3, 26.5, 24.3, 22.8. Data in accordance with the literature.[16]

2-Methoxy-3-methyl-1-phenylbutane-1,3-diol (4ay)

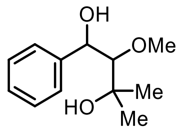

Following **GP5**, 5-methoxy-5*H*-dibenzo[*b*,*d*]thiophen-5-ium triflate **1a** (36 mg, 0.10 mmol) and (*E*)-2-methyl-4-phenylbut-3-en-2-ol **3k** (32.4 mg, 0.20 mmol) gave **4ay** (8.4 mg, 40%) as an oil (single diastereomer). R_f 0.12 [hexane-EtOAc (4:1)]; ^{1}H NMR (400 MHz, $CDCl_3$) δ 7.43 (2H, d, *J* = 7.3 Hz), 7.36 (2H, t, *J* = 8.4 Hz), 7.29–7.24 (1H, m), 5.04 (1H, dd, *J* = 4.2, 2.0 Hz), 3.82 (1H, d, *J* = 4.2 Hz), 3.07 (1H, d, *J* = 2.0 Hz), 3.06 (3H, s), 2.54 (1H, br s), 1.40 (3H, s), 1.31 (3H, s); ^{13}C NMR (101 MHz, $CDCl_3$) δ 142.8, 128.4, 127.4, 126.1, 89.9, 74.5, 72.7, 62.4, 27.6, 26.0; IR (neat, cm^{-1}): 3408, 3062, 3027, 2974, 2929, 2831, 1495, 1450, 1382, 1199, 1161, 1104; HRMS (APCI): Found $(M+Na)^+$ 233.1147, $C_{12}H_{18}O_3Na$ requires 233.1148.

5-Hydroxy-4-methoxy-2,2-dimethyl-5-phenylpentanenitrile (4az)

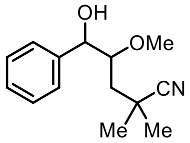

Following **GP5**, 5-methoxy-5*H*-dibenzo[*b*,*d*]thiophen-5-ium triflate **1a** (36 mg, 0.10 mmol) and (*E*)-2,2-dimethyl-5-phenylpent-4-enenitrile **3l** (37 mg, 0.20 mmol) gave **4az** (16.8 mg, 72%, d.r. = 1.7:1) as an inseparable mixture. R_f 0.16 [hexane–EtOAc (3:1)]; ^{1}H NMR (400 MHz, $CDCl_3$) δ 7.41–7.27 (5H, m), 5.06 (0.63H, dd, *J* = 3.5, 1.7 Hz), 4.68 (0.37H, t, *J* = 5.0

Hz), 3.65 (0.37H, ddd, J = 8.2, 4.7, 3.3 Hz), 3.56 (0.63H, ddd, J = 9.8, 3.5, 2.0 Hz), 3.50 (1.90H, s), 3.41 (1.10H, s), 2.63 (0.37H, br d, J = 5.0 Hz), 2.48 (0.63H, br d, J = 1.7 Hz), 1.75 (1H, dd, J = 14.8, 9.8 Hz), 1.68–1.62 (0.74H, m), 1.42 (1H, dd, J = 14.8, 2.0 Hz), 1.37 (1.10H, s), 1.33 (1.90H, s), 1.29 (1.10H, s), 1.17 (1.90H, s); ^{13}C NMR (101 MHz, $CDCl_3$) δ 141.1, 139.6, 128.6 (2 × C), 128.0, 127.7, 126.5, 125.9, 125.2, 125.1, 83.4, 83.0, 75.7, 72.3, 59.4, 57.6, 42.4, 39.0, 31.0, 30.8, 28.0, 27.9, 27.6, 27.4; HRMS (APCI): Found $(M+Na)^+$ 256.1313, $C_{14}H_{19}NO_2Na$ requires 256.1308.

1-Methoxy-2-phenylpropan-2-ol (4ba)

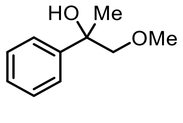

Following **GP5**, 5-methoxy-5*H*-dibenzo[*b*,*d*]thiophen-5-ium triflate **1a** (36 mg, 0.10 mmol) and α-methylstyrene (26 μL, 0.20 mmol) gave **4ba** (15.3 mg, 92%) as an oil. ^{1}H NMR (400 MHz, $CDCl_3$) δ 7.50–7.44 (2H, m), 7.39–7.31 (2H, m), 7.28–7.23 (1H, m), 3.59 (1H, d, J = 9.2 Hz), 3.48 (1H, d, J = 9.2 Hz), 3.38 (3H, s), 2.85 (1H, s), 1.51 (3H, s); ^{13}C NMR (101 MHz, $CDCl_3$) δ 145.6, 128.3, 127.1, 125.1, 80.9, 74.0, 59.6, 26.9. Data in accordance with the literature.[15]

2-Methoxy-1,1-diphenylethan-1-ol (4bb)

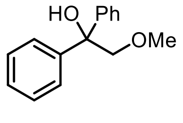

Following **GP5**, 5-methoxy-5*H*-dibenzo[*b*,*d*]thiophen-5-ium triflate **1a** (36 mg, 0.10 mmol) and 1,1-diphenylethylene (35 μL, 0.20 mmol) gave **4bb** (18.3 mg, 80%) as an oil. ^{1}H NMR (400 MHz, $CDCl_3$) δ 7.47–7.40 (4H, m), 7.36–7.29 (4H, m), 7.27–7.22 (2H, m), 3.95 (2H, s), 3.46 (3H, s), 3.43 (1H, br s); ^{13}C NMR (101 MHz, $CDCl_3$) δ 144.5, 128.3, 127.3, 126.5, 79.0, 77.8, 59.5. Data in accordance with the literature.[16]

1-Cyclopropyl-2-methoxy-1-phenylethan-1-ol (4bc)

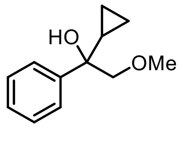

Following **GP5**, 5-methoxy-5*H*-dibenzo[*b*,*d*]thiophen-5-ium triflate **1a** (36 mg, 0.10 mmol) and (1-cyclopropylvinyl)benzene **3m** (28.8 mg, 0.20 mmol) gave **4bc** (11.8 mg, 61%) as an oil. R$_f$ 0.21 [hexane-EtOAc (8:1)]; ^{1}H NMR (400 MHz, $CDCl_3$) δ 7.51–7.45 (2H, m), 7.38–

7.32 (2H, m), 7.28–7.23 (1H, m), 3.80 (1H, d, J = 9.3 Hz), 3.62 (1H, d, J = 9.3 Hz), 3.37 (3H, s), 2.67 (1H, br s), 1.16 (1H, tt, J = 8.4, 5.4 Hz), 0.55–0.42 (2H, m), 0.40–0.27 (2H, m); ^{13}C NMR (101 MHz, $CDCl_3$) δ 144.6, 128.1, 127.0, 125.7, 80.2, 74.2, 59.6, 19.2, 1.3, 0.1; IR (neat, cm^{-1}): 3484, 3086, 3059, 3007, 2924, 2892, 2823, 1493, 1471, 1447, 1392, 1331, 1263, 1181, 1112; HRMS (APCI): Found $(M+Na)^+$ 215.1043, $C_{12}H_{16}O_2Na$ requires 215.1043.

2-Methoxy-1-phenylcyclohexan-1-ol (4bd)

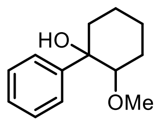

Following **GP5**, 5-methoxy-5*H*-dibenzo[*b*,*d*]thiophen-5-ium triflate **1a** (36 mg, 0.10 mmol) and 1-phenyl-1-cyclohexene (32 μL, 0.20 mmol) gave **4bd** (14.4 mg, 70%, d.r. = 6.6:1) as an inseparable mixture. ^{1}H NMR (400 MHz, $CDCl_3$) δ 7.62–7.47 (2H, m), 7.39–7.31 (2H, m), 7.28–7.19 (1H, m), 3.58 (0.88H, dd, J = 11.0, 4.7 Hz), 3.31 (0.13H, t, J = 3.6 Hz), 3.09 (2.6H, s), 3.03 (0.4H, s), 2.67 (1H, br s), 2.07–1.97 (1H, m), 1.91–1.80 (2H, m), 1.72–1.51 (4H, m), 1.40–1.28 (1H, m); ^{13}C NMR (101 MHz, $CDCl_3$) (major isomer) δ 147.9, 128.2, 126.6, 125.0, 83.1, 75.7, 57.3, 39.5, 26.8, 24.3, 21.5; (minor isomer) δ 146.8, 128.0, 127.1, 126.4, 83.4, 74.3, 57.5, 33.0, 25.3, 21.3, 19.9. Data in accordance with the literature.[20]

***tert*-Butyl 7-chloro-5-hydroxy-5-(methoxymethyl)-2,3,4,5-tetrahydro-1*H*-benzo[*b*]azepine-1-carboxylate (4be)**

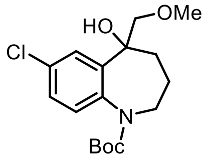

Following **GP5**, 5-methoxy-5*H*-dibenzo[*b*,*d*]thiophen-5-ium triflate **1a** (36 mg, 0.10 mmol) and *tert*-butyl 7-chloro-5-methylene-2,3,4,5-tetrahydro-1*H*-benzo[*b*]azepine-1-carboxylate **3n** (58.8 mg, 0.20 mmol) gave **4be** (23.4 mg, 68%) as a solid (mixture of rotamers). R_f 0.22 [hexane-EtOAc (4:1)]; ^{1}H NMR (500 MHz, $CDCl_3$) δ 7.82–7.73 (1H, m), 7.23–7.10 (1.4H, m), 7.00 (0.6H, d, J = 8.4 Hz), 4.38 (0.7H, dt, J = 14.3, 3.5 Hz), 4.21 (0.3H, d, J = 14.0 Hz), 3.62 (1H, d, J = 9.3 Hz), 3.50–3.33 (1H, m), 3.30 (3H, s), 3.20 (0.7H, br s) and 3.12 (0.3H, br s), 2.72 (0.3H, t, J = 13.3 Hz), 2.63 (0.7H, t, J = 13.3 Hz), 2.20–1.85 (2H, m), 1.74–1.66 (2H, m), 1.52 (2.7H, s), 1.38 (6.3H, s); ^{13}C NMR (126 MHz, $CDCl_3$) δ Major rotamer: 153.7, 142.4, 138.1, 132.8, 130.6, 127.6, 127.3, 80.6, 75.8, 75.7, 59.4, 47.6, 36.9, 28.5, 24.7, Minor rotamer: 154.2, 142.7, 138.2, 133.1, 130.9, 128.1, 127.5, 80.7, 76.0, 75.8, 59.5, 48.8, 36.9, 28.5, 25.4; IR (neat, cm^{-1}): 3442, 2976, 2922, 2857, 2821, 1683, 1478, 1456, 1444, 1392, 1368, 1356,

1214, 1296, 1254, 1223, 1191, 1152, 1103; HRMS (APCI): Found $(M+Na)^+$ 364.1290, $C_{17}H_{24}O_4NClNa$ requires 364.1286.
4be was further characterised by X-ray crystallographic analysis. CCDC: 2432415.

1-(4-Chlorophenyl)-2-(methoxy-*d*3)ethan-1-ol (4bf)

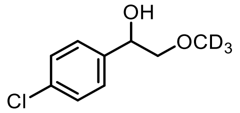

Following **GP5**, 5-(methoxy-d_3)-5*H*-dibenzo[*b*,*d*]thiophen-5-ium triflate **1b** (37 mg, 0.10 mmol) and 4-chlorostyrene (24 μL, 0.20 mmol) gave **4bf** (15.2 mg, 80%) as an oil. ^{1}H NMR (400 MHz, $CDCl_3$) δ 7.32 (4H, s), 4.86 (1H, ddd, *J* = 8.8, 3.3, 1.8 Hz), 3.51 (1H, dd, *J* = 9.8, 3.3 Hz), 3.38 (1H, dd, *J* = 9.8, 8.8 Hz), 2.85 (1H, br d, *J* = 2.4 Hz); ^{13}C NMR (101 MHz, $CDCl_3$) δ 138.9, 133.7, 128.7, 127.6, 78.0, 72.1, 59.1–57.4 (m); ^{2}H NMR (61 MHz, $CDCl_3$) δ 3.4; HRMS (APCI): Found $(M+Na)^+$ 212.0531, $C_9H_8D_3ClO_2Na$ requires 212.0528.

1-(4-Chlorophenyl)-2-ethoxyethan-1-ol (4bg)

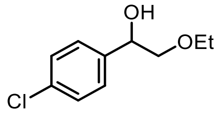

Following **GP5**, 5-ethoxy-5*H*-dibenzo[*b*,*d*]thiophen-5-ium triflate **1c** (38 mg, 0.10 mmol) and 4-chlorostyrene (24 μL, 0.20 mmol) gave **4bg** (12.6 mg, 63%) as an oil. R_f 0.18 [hexane–EtOAc (4:1)]; ^{1}H NMR (500 MHz, $CDCl_3$) δ 7.31 (4H, s), 4.85 (1H, dt, *J* = 9.0, 2.6 Hz), 3.64–3.50 (3H, m), 3.38 (1H, dd, *J* = 9.8, 8.9 Hz), 2.97 (1H, br d, *J* = 2.2 Hz), 1.23 (3H, t, *J* = 7.0 Hz); ^{13}C NMR (126 MHz, $CDCl_3$) δ 139.0, 133.6, 128.6, 127.7, 76.0, 72.2, 66.9, 15.2; HRMS (APCI): Found $(M+Na)^+$ 223.0500, $C_{10}H_{13}O_2ClNa$ requires 223.0496.
Following **GP5**, but with 4-chlorostyrene (60 μL, 0.50 mmol, 5.0 equiv.) gave **4bg** (14.4 mg, 72%) as an oil.

1-(4-Chlorophenyl)-2-(2,2,2-trichloroethoxy)ethan-1-ol (4bh)

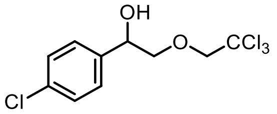

Following **GP5**, 5-(2,2,2-trichloroethoxy)-5*H*-dibenzo[*b*,*d*]thiophen-5-ium triflate **1e** (48 mg, 0.10 mmol) and 4-chlorostyrene (60 μL, 0.50 mmol, 5.0 equiv.) gave **4bh** (6.0 mg, 20%) as an oil. R_f 0.30 [hexane–EtOAc (4:1)]; ^{1}H NMR (400 MHz, $CDCl_3$) δ 7.34 (4H, s), 4.97 (1H, dt, *J* = 8.6, 2.7 Hz), 4.18 (2H, s), 3.94 (1H, dd, *J* = 9.9, 3.2 Hz), 3.76 (1H, dd, *J* = 9.9, 8.6 Hz), 2.78

(1H, br d, J = 2.7 Hz); ^{13}C NMR (101 MHz, $CDCl_3$) δ 138.1, 134.0, 128.9, 127.7, 96.9, 83.6, 78.2, 72.5; HRMS (APCI): Found $(M+Na)^+$ 324.9322, $C_{10}H_{10}O_2Cl_4Na$ requires 324.9327.

1-(4-Chlorophenyl)-2-(2,2,2-trifluoroethoxy)ethan-1-ol (4bi)

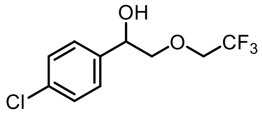

Following **GP5**, 5-(2,2,2-trifluoroethoxy)-5*H*-dibenzo[*b*,*d*]thiophen-5-ium triflate **1d** (43 mg, 0.10 mmol) and 4-chlorostyrene (24 µL, 0.20 mmol) gave **4bi** (11.5 mg, 45%) as an oil. R_f 0.24 [hexane–EtOAc (4:1)]; ^{1}H NMR (400 MHz, $CDCl_3$) δ 7.34 (2H, d, J = 8.8 Hz), 7.31 (2H, d, J = 8.8 Hz), 4.91 (1H, dt, J = 8.6, 2.7 Hz), 4.02–3.82 (2H, m), 3.77 (1H, dd, J = 9.8, 3.2 Hz), 3.60 (1H, t, J = 9.2 Hz), 2.69 (1H, br d, J = 2.7 Hz); ^{13}C NMR (101 MHz, $CDCl_3$) δ 138.0, 134.1, 128.9, 127.7, 123.9 (q, $J_{C\text{-}F}$ = 279.6 Hz), 77.9, 72.4, 68.9 (q, $J_{C\text{-}F}$ = 34.2 Hz); ^{19}F NMR (376 MHz, $CDCl_3$) δ −74.1; HRMS (APCI): Found $(M+Na)^+$ 277.0217, $C_{10}H_{10}O_2ClF_3Na$ requires 277.0214.

Following **GP5**, but with 4-chlorostyrene (60 µL, 0.50 mmol, 5.0 equiv.) gave **4bi** (14.5 mg, 57%) as an oil.

1-(4-Chlorophenyl)-2-isobutoxyethan-1-ol (4bj)

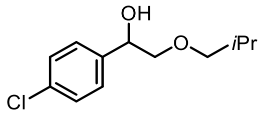

Following **GP5**, 5-isobutoxy-5*H*-dibenzo[*b*,*d*]thiophen-5-ium triflate **1f** (41 mg, 0.10 mmol) and 4-chlorostyrene (24 µL, 0.20 mmol) gave **4bj** (14.2 mg, 62%) as an oil. R_f 0.25 [hexane–EtOAc (8:1)]; ^{1}H NMR (400 MHz, $CDCl_3$) δ 7.32 (4H, s), 4.86 (1H, ddd, J = 8.9, 3.3, 2.2 Hz), 3.55 (1H, dd, J = 9.7, 3.3 Hz), 3.37 (1H, dd, J = 9.7, 8.9 Hz), 3.30 (1H, dd, J = 9.2, 6.6 Hz), 3.24 (1H, dd, J = 9.2, 6.7 Hz), 2.92 (1H, br d, J = 2.2 Hz), 1.98–1.80 (1H, m), 0.92 (6H, d, J = 6.9 Hz); ^{13}C NMR (101 MHz, $CDCl_3$) δ 139.0, 133.6, 128.6, 127.7, 78.4, 76.4, 72.2, 28.5, 19.4; HRMS (APCI): Found $(M+Na)^+$ 251.0814, $C_{12}H_{17}O_2ClNa$ requires 251.0809.

Following **GP5**, but with 4-chlorostyrene (60 µL, 0.50 mmol, 5.0 equiv.) gave **4bj** (16.5 mg, 72%) as an oil.

1-(4-Chlorophenyl)-2-(cyclohexylmethoxy)ethan-1-ol (4bk)

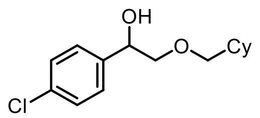

Following **GP5**, 5-(cyclohexylmethoxy)-5*H*-dibenzo[*b*,*d*]thiophen-5-ium triflate **1g** (45 mg, 0.10 mmol) and 4-chlorostyrene (24 µL, 0.20 mmol) gave **4bk** (13.7 mg, 51%) as an oil. R_f 0.25 [hexane–EtOAc (8:1)]; ^{1}H NMR (400 MHz, $CDCl_3$) δ 7.31 (4H, s), 4.85 (1H, ddd, *J* = 9.0, 3.3, 2.1 Hz), 3.54 (1H, dd, *J* = 9.8, 3.3 Hz), 3.35 (1H, dd, *J* = 9.8, 9.0 Hz), 3.33 (1H, dd, *J* = 9.3, 6.8 Hz), 3.27 (1H, dd, *J* = 9.3, 6.5 Hz), 2.91 (1H,br d, *J* = 2.1 Hz), 1.77–1.55 (6H, m), 1.31–1.10 (3H, m), 1.02–0.87 (2H, m); ^{13}C NMR (101 MHz, $CDCl_3$) δ 139.0, 133.6, 128.6, 127.7, 77.4, 76.4, 72.2, 38.1, 30.1, 26.7, 26.0; HRMS (APCI): Found $(M+Na)^+$ 291.1126, $C_{15}H_{21}O_2ClNa$ requires 291.1122.

Following **GP5**, but with 4-chlorostyrene (60 µL, 0.50 mmol, 5.0 equiv.) gave **4bk** (17.5 mg, 65%) as an oil.

2-(2-(Adamantan-1-yl)ethoxy)-1-(4-chlorophenyl)ethan-1-ol (4bl)

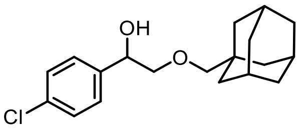

Following **GP5**, 5-((adamantan-1-yl)methoxy)-5*H*-dibenzo[*b*,*d*]thiophen-5-ium triflate **1h** (50 mg, 0.10 mmol) and 4-chlorostyrene (24 µL, 0.20 mmol) gave **4bl** (10.9 mg, 34%) as a solid. R_f 0.25 [hexane–EtOAc (8:1)]; ^{1}H NMR (400 MHz, $CDCl_3$) δ 7.32 (4H, s), 4.86 (1H, ddd, *J* = 9.0, 3.3, 2.0 Hz), 3.53 (1H, dd, *J* = 9.8, 3.3 Hz), 3.35 (1H, t, *J* = 9.4 Hz), 3.11 (1H, d, *J* = 8.9 Hz), 3.03 (1H, d, *J* = 8.9 Hz), 2.90 (1H, br d, *J* = 2.0 Hz), 2.01–1.94 (3H, m), 1.78–1.70 (3H, m), 1.69–1.62 (3H, m), 1.58–1.53 (6H, m); ^{13}C NMR (101 MHz, $CDCl_3$) δ 139.0, 133.5, 128.6, 127.7, 82.5, 76.9, 72.1, 39.8, 37.3, 34.3, 28.3; HRMS (APCI): Found $(M+Na)^+$ 343.1438, $C_{19}H_{25}O_2ClNa$ requires 343.1435.

Following **GP5**, but with 4-chlorostyrene (60 µL, 0.50 mmol, 5.0 equiv.) gave **4bl** (16.4 mg, 51%) as a solid.

3-(2-(4-Chlorophenyl)-2-hydroxyethoxy)propanenitrile (4bm)

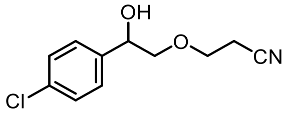

Following **GP5**, 5-(2-cyanoethoxy)-5*H*-dibenzo[*b*,*d*]thiophen-5-ium triflate **1k** (40 mg, 0.10 mmol) and 4-chlorostyrene (24 µL, 0.20 mmol) gave **4bm** (18.1 mg, 80%) as an oil. R_f 0.18

[hexane–EtOAc (1:1)]; ^{1}H NMR (400 MHz, $CDCl_3$) δ 7.31 (4H, s), 4.88 (1H, dd, *J* = 8.5, 3.3 Hz), 3.73 (2H, t, *J* = 6.2 Hz), 3.63 (1H, dd, *J* = 9.8, 3.3 Hz), 3.49 (1H, dd, *J* = 9.8, 8.5 Hz), 2.88 (1H, br s), 2.63 (2H, t, *J* = 6.2 Hz); ^{13}C NMR (101 MHz, $CDCl_3$) δ 138.5, 133.8, 128.7, 127.6, 117.8, 76.6, 72.2, 66.0, 19.0; HRMS (APCI): Found $(M+Na)^+$ 248.0452, $C_{11}H_{12}O_2NClNa$ requires 248.0449.

1-(4-Chlorophenyl)-2-(3-chloropropoxy)ethan-1-ol (4bn)

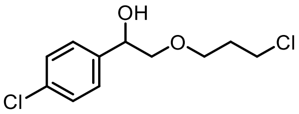

Following **GP5**, 5-(3-chloropropoxy)-5*H*-dibenzo[*b*,*d*]thiophen-5-ium triflate **1i** (42.7 mg, 0.10 mmol) and 4-chlorostyrene (60 μL, 0.50 mmol, 5.0 equiv.) gave **4bn** (16.1 mg, 65%) as an oil. R_f 0.32 [hexane-EtOAc (7:3)]; ^{1}H NMR (500 MHz, $CDCl_3$) δ 7.32 (4H, s), 4.86 (1H, dd, *J* = 8.8, 3.2 Hz), 3.77–3.61 (4H, m), 3.58 (1H, dd, *J* = 9.8, 3.2 Hz), 3.43 (1H, t, *J* = 9.8 Hz), 2.42 (1H, br s), 2.05 (2H, pent, *J* = 6.1 Hz); ^{13}C NMR (126 MHz, $CDCl_3$) δ 138.8, 133.7, 128.7, 127.7, 76.4, 72.2, 67.9, 41.9, 32.6; IR (neat, cm^{-1}): 3423, 2869, 1597, 1490, 1447, 1405, 1356, 1301, 1221, 1194, 1172, 1114; HRMS (APCI): Found $(M+Na)^+$ 271.0261, $C_{11}H_{14}O_2Cl_2Na$ requires 271.0263.

2-(3-Bromopropoxy)-1-(4-chlorophenyl)ethan-1-ol (4bo)

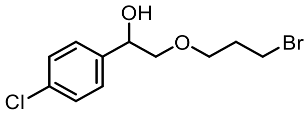

Following **GP5**, 5-(3-bromopropoxy)-5*H*-dibenzo[*b*,*d*]thiophen-5-ium **1j** (47.1 mg, 0.10 mmol) and 4-chlorostyrene (24 μL, 0.20 mmol) gave **4bo** (20.3 mg, 69%) as an oil. R_f 0.26 [hexane-EtOAc (4:1)]; ^{1}H NMR (500 MHz, $CDCl_3$) δ 7.32 (4H, s), 4.87 (1H, dd, *J* = 8.7, 3.3 Hz), 3.73–3.61 (2H, m), 3.58 (1H, dd, *J* = 9.8, 3.3 Hz), 3.51 (2H, t, *J* = 6.4 Hz), 3.44 (1H, dd, *J* = 9.8, 8.7 Hz), 2.13 (2H, p, *J* = 6.4 Hz); ^{13}C NMR (126 MHz, $CDCl_3$) δ 138.8, 133.7, 128.7, 127.7, 76.4, 72.2, 68.8, 32.6, 30.5; IR (neat, cm^{-1}): 3424, 2866, 1597, 1490, 1434, 1405, 1358, 1308, 1285, 1257, 1214, 1195, 1113; HRMS (APCI): Found $(M+Na)^+$ 314.9759, $C_{11}H_{14}O_2BrClNa$ requires 314.9758.

1-(4-Chlorophenyl)-2-(2-(phenylsulfonyl)ethoxy)ethan-1-ol (4bp)

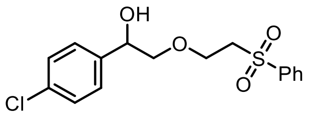

Following **GP5**, 5-(2-(phenylsulfonyl)ethoxy)-5*H*-dibenzo[*b*,*d*]thiophen-5-ium triflate **1l** (51.8 mg, 0.10 mmol) and 4-chlorostyrene (24 μL, 0.20 mmol) gave **4bp** (23.5 mg, 69%) as an oil. R*f* 0.09 [hexane-EtOAc (7:3)]; ^{1}H NMR (400 MHz, $CDCl_3$) δ 7.96–7.91 (2H, m), 7.71–7.65 (1H, m), 7.61–7.56 (2H, m), 7.29 (2H, d, *J* = 8.6 Hz), 7.24 (2H, d, *J* = 8.6 Hz), 4.69 (1H, dd, *J* = 8.7, 3.1 Hz), 3.98–3.79 (2H, m), 3.50 (1H, dd, *J* = 9.7, 3.1 Hz), 3.41 (2H, t, *J* = 5.8 Hz), 3.34 (1H, dd, *J* = 9.7, 8.7 Hz), 2.45 (1H, br s); ^{13}C NMR (101 MHz, $CDCl_3$) δ 139.9, 138.4, 134.1, 133.7, 129.5, 128.7, 128.1, 127.6, 76.6, 71.9, 64.6, 56.3; IR (neat, cm^{-1}): 3490, 3059, 2915, 1585, 1491, 1446, 1402, 1360, 1306, 1291, 1266, 1192, 1142, 1119; HRMS (ESI): Found $(M+Na)^+$ 363.0411, $C_{16}H_{17}O_3ClSNa$ requires 363.0400.

2-(2-(2-(4-Chlorophenyl)-2-hydroxyethoxy)ethyl)isoindoline-1,3-dione (4bq)

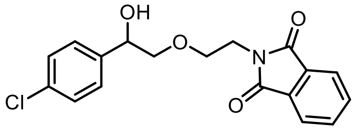

Following **GP5**, 5-(2-(1,3-dioxoisoindolin-2-yl)ethoxy)-5*H*-dibenzo[*b*,*d*]thiophen-5-ium triflate **1p** (52.3 mg, 0.10 mmol) and 4-chlorostyrene (24 μL, 0.20 mmol) and gave **4bq** (24.9 mg, 72%) as an oil. R*f* 0.2 [hexane-EtOAc (4:1)]; ^{1}H NMR (400 MHz, $CDCl_3$) δ 7.84 (2H, dd, *J* = 5.5, 3.1 Hz), 7.73 (2H, dd, *J* = 5.5, 3.1 Hz), 7.27 (2H, d, *J* = 8.6 Hz), 7.23 (2H, d, *J* = 8.6 Hz), 4.79 (1H, dd, *J* = 8.3, 3.3 Hz), 3.91 (2H, t, *J* = 5.4 Hz), 3.81–3.69 (2H, m), 3.65 (1H, dd, *J* = 9.5, 3.3 Hz), 3.44 (1H, dd, *J* = 9.5, 8.3 Hz), 2.75 (1H, br s); ^{13}C NMR (101 MHz, $CDCl_3$) δ 168.6, 138.9, 134.2, 133.5, 132.1, 128.6, 127.6, 123.5, 76.3, 72.1, 68.6, 37.6; IR (neat, cm^{-1}): 3471, 3055, 2915, 1772, 1706, 1615, 1491, 1468, 1427, 1393, 1356, 1321, 1265, 1189, 1172, 1120; HRMS (APCI): Found $(M+Na)^+$ 368.0661, $C_{18}H_{16}O_4NClNa$ requires 368.0660.

4-(2-(4-Chlorophenyl)-2-hydroxyethoxy)-2-methylbutan-2-ol (4br)

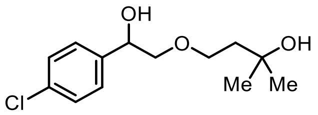

Following **GP5**, 5-(3-hydroxy-3-methylbutoxy)-5*H*-dibenzo[*b*,*d*]thiophen-5-ium triflate **1q** (44 mg, 0.10 mmol) and 4-chlorostyrene (24 μL, 0.20 mmol) gave **4br** (9.3 mg, 36%) as an oil. R*f* 0.13 [hexane–EtOAc (1:1)]; ^{1}H NMR (400 MHz, $CDCl_3$) δ 7.32 (4H, s), 4.86 (1H, dd, *J* = 8.5, 3.4 Hz), 3.82–3.68 (2H, m), 3.60 (1H, dd, *J* = 9.9, 3.4 Hz), 3.46 (1H, dd, *J* = 9.9, 8.5

Hz), 2.87 (1H, br s), 2.59 (1H, br s), 1.82–1.77 (2H, m), 1.25 (6H, s); ^{13}C NMR (101 MHz, $CDCl_3$) δ 138.9, 133.8, 128.8, 127.6, 76.6, 72.2, 70.7, 68.9, 41.7, 29.7, 29.6; HRMS (APCI): Found $(M+Na)^+$ 281.0920, $C_{13}H_{19}O_3ClNa$ requires 281.0915.

Following **GP5**, but with 4-chlorostyrene (60 μL, 0.50 mmol, 5.0 equiv.) gave **4br** (13.7 mg, 53%) as an oil.

2-(2-(4-Chlorophenyl)-2-hydroxyethoxy)ethyl acrylate (4bs)

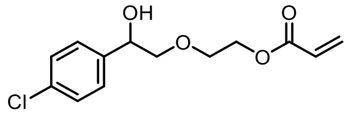

Following **GP5**, 5-(2-(acryloyloxy)ethoxy)-5*H*-dibenzo[*b*,*d*]thiophen-5-ium triflate **1r** (45 mg, 0.10 mmol) and 4-chlorostyrene (24 μL, 0.20 mmol) gave **4bs** (17.6 mg, 65%) as an oil. R_f 0.24 [hexane–EtOAc (2:1)]; ^{1}H NMR (400 MHz, $CDCl_3$) δ 7.31 (4H, s), 6.44 (1H, dd, *J* = 17.3, 1.4 Hz), 6.15 (1H, dd, *J* = 17.3, 10.4 Hz), 5.86 (1H, dd, *J* = 10.4, 1.4 Hz), 4.87 (1H, dd, *J* = 8.8, 3.3 Hz), 4.34 (2H, t, *J* = 4.7 Hz), 3.84–3.70 (2H, m), 3.64 (1H, dd, *J* = 9.8, 3.3 Hz), 3.46 (1H, dd, *J* = 9.8, 8.8 Hz), 2.92 (1H, br s); ^{13}C NMR (101 MHz, $CDCl_3$) δ 166.3, 138.7, 133.7, 131.5, 128.7, 128.2, 127.7, 76.7, 72.2, 69.4, 63.5; HRMS (APCI): Found $(M+Na)^+$ 293.0546, $C_{13}H_{15}O_4ClNa$ requires 293.0551.

Following **GP5**, but with 4-chlorostyrene (60 μL, 0.50 mmol, 5.0 equiv.) gave **4bs** (19.2 mg, 71%) as an oil.

2-(Allyloxy)-1-(4-chlorophenyl)ethan-1-ol (4bt)

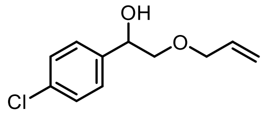

Following **GP5**, 5-(allyloxy)-5*H*-dibenzo[*b*,*d*]thiophen-5-ium triflate **1m** (39.0 mg, 0.10 mmol) and 4-chlorostyrene (60 μL, 0.50 mmol, 5.0 equiv.) and gave **4bt** (9.8 mg, 46%) as an oil. R_f 0.32 [hexane-EtOAc (7:3)]; ^{1}H NMR (400 MHz, $CDCl_3$) δ 7.32 (4H, s), 5.92 (1H, ddt, *J* = 17.1, 10.2, 5.7 Hz), 5.29 (1H, dq, *J* = 17.1, 1.5 Hz), 5.22 (1H, dq, *J* = 10.3, 1.5 Hz), 4.88 (1H, dd, *J* = 8.9, 3.2 Hz), 4.06 (2H, dt, *J* = 5.7, 1.4 Hz), 3.58 (1H, dd, *J* = 9.8, 3.2 Hz), 3.41 (1H, dd, *J* = 9.8, 8.9 Hz); ^{13}C NMR (101 MHz, $CDCl_3$) δ 138.8, 134.3, 133.7, 128.7, 127.7, 117.8, 75.6, 72.4, 72.3; IR (neat, cm^{-1}): 3429, 2921, 2856, 1491, 1464, 1405, 1349, 1318, 1216, 1195; HRMS (APCI): Found $(M+Na)^+$ 235.0501, $C_{11}H_{13}O_2ClNa$ requires 235.0496.

2-((4-Chlorobut-2-yn-1-yl)oxy)-1-(4-chlorophenyl)ethan-1-ol (4bu)

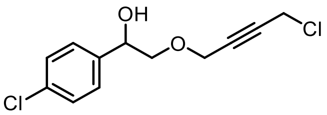

Following **GP5**, 5-((4-chlorobut-2-yn-1-yl)oxy)-5*H*-dibenzo[*b*,*d*]thiophen-5-ium triflate **1o** (44 mg, 0.10 mmol) and 4-chlorostyrene (24 μL, 0.20 mmol) gave **4bu** (15.5 mg, 60%) as an oil. R_f 0.13 [hexane–EtOAc (4:1)]; ^{1}H NMR (400 MHz, $CDCl_3$) δ 7.33 (4H, s), 4.89 (1H, dt, *J* = 8.7, 2.6 Hz), 4.34–4.22 (2H, m), 4.17 (2H, t, *J* = 2.0 Hz), 3.68 (1H, dd, *J* = 9.7, 3.2 Hz), 3.50 (1H, dd, *J* = 9.7, 8.7 Hz), 2.77 (1H, br d, *J* = 2.6 Hz); ^{13}C NMR (101 MHz, $CDCl_3$) δ 138.6, 133.8, 128.7, 127.7, 82.1, 81.8, 75.4, 72.2, 58.9, 30.3; HRMS (APCI): Found $(M+Na)^+$ 281.0108, $C_{12}H_{12}O_2Cl_2Na$ requires 281.0107.

Following **GP5**, but with 4-chlorostyrene (60 μL, 0.50 mmol, 5.0 equiv.) gave **4bu** (16.6 mg, 64%) as an oil.

2-(But-3-yn-1-yloxy)-1-(4-chlorophenyl)ethan-1-ol (4bv)

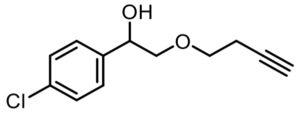

Following **GP5**, 5-(but-3-yn-1-yloxy)-5*H*-dibenzo[*b*,*d*]thiophen-5-ium triflate **1n** (40.2 mg, 0.10 mmol) and 4-chlorostyrene (24 μL, 0.20 mmol) gave **4bv** (14.6 mg, 65%) as an oil. R_f 0.32 [hexane-EtOAc (4:1)]; ^{1}H NMR (400 MHz, $CDCl_3$) δ 7.32 (4H, s), 4.88 (1H, dd, *J* = 8.8, 3.2 Hz), 3.79–3.50 (3H, m), 3.44 (1H, dd, *J* = 9.8, 8.8 Hz), 2.50 (2H, td, *J* = 6.6, 2.7 Hz), 2.02 (1H, t, *J* = 2.7 Hz); ^{13}C NMR (101 MHz, $CDCl_3$) δ 138.6, 133.7, 128.7, 127.7, 81.3, 76.4, 72.2, 69.8, 69.4, 20.0; IR (neat, cm^{-1}): 3435, 3031, 3053, 2915, 2870, 1702, 1596, 1492, 1405, 1360, 1317, 1265, 1222, 1195, 1116; HRMS (APCI): Found $(M+Na)^+$ 247.0498, $C_{12}H_{13}O_2ClNa$ requires 247.0496.

1-(4-Chlorophenyl)-2-(((3a*S*,5a*R*,8a*R*,8b*S*)-2,2,7,7-tetramethyltetrahydro-3a*H*-bis([1,3]dioxolo)[4,5-*b*:4',5'-*d*]pyran-3a-yl)methoxy)ethan-1-ol (4bw)

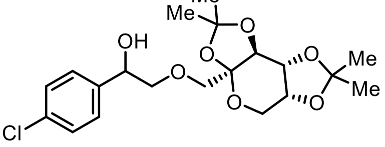

Following **GP5**, 5-(((3a*S*,5a*R*,8a*R*,8b*S*)-2,2,7,7-tetramethyltetrahydro-3a*H*-bis([1,3]dioxolo)[4,5-*b*:4',5'-*d*]pyran-3a-yl)methoxy)-5*H*-dibenzo[*b*,*d*]thiophen-5-ium triflate **1t** (59.3 mg, 0.10 mmol) and 4-chlorostyrene (24 μL, 0.20 mmol) gave **4bw** (17.4 mg, 42%,

d.r. = 1:1) as an oil. R_f 0.17 [hexane-EtOAc (7:3)]; ^{1}H NMR (400 MHz, $CDCl_3$) δ 7.31 (4H, s), 4.93–4.86 (1H, m), 4.61 (1H, dt, *J* = 7.9, 2.0 Hz), 4.35 (1H, dd, *J* = 10.3, 2.5 Hz), 4.28–4.18 (1H, m), 3.92 (1H, dd, *J* = 13.0, 2.0 Hz), 3.82 (0.5H, d, *J* = 10.0 Hz), 3.80–3.71 (2H, m), 3.71 – 3.65 (1H, m), 3.57 (0.5H, d, *J* = 10.4 Hz), 3.51 (0.5H, dd, *J* = 10.3, 9.0 Hz), 3.39 (0.5H, t, *J* = 9.7 Hz), 1.55 (1.5H, s), 1.54 (1.5H, s), 1.52 (1.5H, s), 1.49 (1.5H, s), 1.41 (1.5H, s), 1.39 (1.5H, s), 1.36 (1.5H, s), 1.35 (1.5H, s); ^{13}C NMR (101 MHz, $CDCl_3$) δ 138.6, 138.4, 133.7, 133.6, 128.7 (2 × C), 127.7, 127.6, 109.2, 108.8, 108.6, 102.6 (2 × C), 78.1, 74.2, 73.1, 72.1, 71.8, 71.3, 71.2, 71.0, 70.9, 70.8, 70.7, 70.3, 70.2 (2 × C), 65.7, 61.4, 61.3, 61.2, 26.6 (2 × C), 26.0, 25.9, 25.5, 25.3, 24.1, 24.0; IR (neat, cm^{-1}): 3472, 2988, 2931, 1492, 1456, 1381, 1315, 1251, 1207, 1184, 1165, 1117; HRMS (APCI): Found $(M+Na)^+$ 437.1340, $C_{20}H_{27}O_7ClNa$ requires 437.1338.

Methyl (2*S*)-3-(2-(4-chlorophenyl)-2-hydroxyethoxy)-2-(1,3-dioxoisoindolin-2-yl)propanoate (4bx)

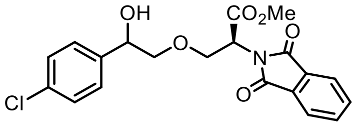

Following **GP5**, (*S*)-5-(2-(1,3-dioxoisoindolin-2-yl)-3-methoxy-3-oxopropoxy)-5*H*-dibenzo[*b*,*d*]thiophen-5-ium triflate **1s** (58.2 mg, 0.10 mmol) and 4-chlorostyrene (24 μL, 0.20 mmol) gave **4bx** (29.5 mg, 73%, d.r. = 1:1) as an oil. R_f 0.17 [hexane-EtOAc (2:1)]; ^{1}H NMR (400 MHz, $CDCl_3$) δ 7.86 (2H, dd, *J* = 5.5, 3.1 Hz), 7.76 (2H, dd, *J* = 5.5, 3.1 Hz), 7.24–7.13 (4H, m), 5.16 (0.5H, t, *J* = 5.2 Hz), 5.13 (0.5H, t, *J* = 5.4 Hz), 4.78–4.71 (1H, m), 4.30–4.24 (0.5H, m), 4.22–4.17 (1H, m), 4.12 (0.5H, dd, *J* = 10.4, 5.1 Hz), 3.75 (3H, s), 3.70 (0.5H, dd, *J* = 9.8, 3.4 Hz), 3.58 (0.5H, dd, *J* = 9.7, 3.9 Hz), 3.52 (0.5H, dd, *J* = 9.7, 7.9 Hz), 3.43 (0.5H, dd, *J* = 9.8, 7.8 Hz), 2.63 (1H, br s); ^{13}C NMR (101 MHz, $CDCl_3$) δ 167.9 (2 × C), 167.7, 167.6, 138.8 (2 × C), 134.5, 133.5, 133.4, 131.8, 128.5 (2 × C), 127.6, 127.5, 123.8, 76.4, 76.2, 72.0, 71.9, 67.9, 67.7, 53.0, 51.4 (2 × C); IR (neat, cm^{-1}): 3479, 2953, 2923, 1775, 1744, 1710, 1612, 1491, 1468, 1436, 1390, 1289, 1259, 1222, 1173, 1121; HRMS (APCI): Found $(M)^+$ 403.0825, $C_{20}H_{18}O_6NCl$ requires 403.0817.

Methyl 3-(2-(4-chlorophenyl)-2-hydroxyethoxy)-2-(1,3-dioxoisoindolin-2-yl)propanoate (4bx')

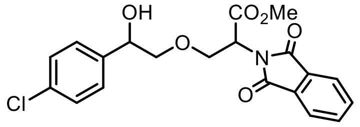

Following **GP5**, 5-(2-(1,3-dioxoisoindolin-2-yl)-3-methoxy-3-oxopropoxy)-5*H*-dibenzo[*b*,*d*]thiophen-5-ium triflate **1s** (58.2 mg, 0.10 mmol) and 4-chlorostyrene (24 μL, 0.20 mmol) gave **4bx'** (30.2 mg, 75%, d.r. = 1:1) as an oil. R_f 0.17 [hexane-EtOAc (2:1)]; ^{1}H NMR (400 MHz, $CDCl_3$) δ 7.86 (2H, dd, J = 5.5, 3.1 Hz), 7.76 (2H, dd, J = 5.5, 3.1 Hz), 7.24–7.13 (4H, m), 5.15 (0.5H, t, J = 5.2 Hz), 5.13 (0.5H, t, J = 5.4 Hz), 4.79–4.70 (1H, m), 4.30–4.24 (0.5H, m), 4.22–4.16 (1H, m), 4.12 (0.5H, dd, J = 10.4, 5.1 Hz), 3.75 (3H, s), 3.69 (0.5H, dd, J = 9.8, 3.4 Hz), 3.57 (0.5H, dd, J = 9.7, 3.9 Hz), 3.52 (0.5H, dd, J = 9.7, 7.9 Hz), 3.43 (0.5H, dd, J = 9.8, 7.8 Hz), 2.62 (1H, br s); ^{13}C NMR (101 MHz, $CDCl_3$) δ 167.9 (2 × C), 167.7, 167.6, 138.8 (2 × C), 134.5, 133.5, 133.4, 131.8, 128.5 (2 × C), 127.6, 127.5, 123.8, 76.4, 76.2, 72.0, 71.9, 67.9, 67.7, 53.0, 51.4 (2 × C); IR (neat, cm^{-1}): 3479, 2953, 2923, 1775, 1744, 1710, 1612, 1491, 1468, 1436, 1390, 1289, 1259, 1222, 1173, 1121; HRMS (APCI): Found $(M)^+$ 403.0823, $C_{20}H_{18}O_6NCl$ requires 403.0817.

1-(4-Chlorophenyl)-2-isopropoxyethan-1-ol (4by)

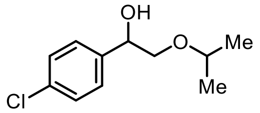

Following **GP5**, 5-isopropoxy-5*H*-dibenzo[*b*,*d*]thiophen-5-ium triflate **1v** (39 mg, 0.10 mmol) and 4-chlorostyrene (24 μL, 0.20 mmol) gave **4by** (10.3 mg, 48%) as an oil. R_f 0.24 [hexane–EtOAc (4:1)]; ^{1}H NMR (400 MHz, $CDCl_3$) δ 7.32 (4H, s), 4.81 (1H, ddd, J = 9.1, 3.3, 2.0 Hz), 3.65 (1H, pent, J = 6.2 Hz), 3.58 (1H, dd, J = 9.5, 3.2 Hz), 3.32 (1H, t, J = 9.3 Hz), 2.94 (1H, br d, J = 2.0 Hz), 1.19 (3H, d, J = 6.2 Hz), 1.18 (3H, d, J = 6.2 Hz); ^{13}C NMR (101 MHz, $CDCl_3$) δ 139.0, 133.6, 128.6, 127.7, 73.8, 72.5, 72.5, 22.2; HRMS (APCI): Found $(M+Na)^+$ 237.0656, $C_{11}H_{15}O_2ClNa$ requires 237.0653.

Following **GP5**, but with 4-chlorostyrene (60 μL, 0.50 mmol, 5.0 equiv.) gave **4by** (13.5 mg, 63%) as an oil.

2-(*sec*-Butoxy)-1-(4-chlorophenyl)ethan-1-ol (4bz)

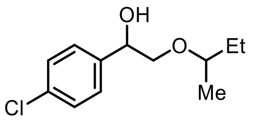

Following **GP5**, 5-(*sec*-butoxy)-5*H*-dibenzo[*b*,*d*]thiophen-5-ium triflate **1w** (41 mg, 0.10 mmol) and 4-chlorostyrene (24 μL, 0.20 mmol) gave **4bz** (9.1 mg, 40%, d.r. = 1:1) as an inseparable mixture. R_f 0.36 [hexane–EtOAc (4:1)]; ^{1}H NMR (400 MHz, $CDCl_3$) δ 7.32 (4H, s), 4.84–4.79 (1H, m), 3.64 (0.5H, dd, *J* = 9.5, 3.3 Hz), 3.53 (0.5H, dd, *J* = 9.6, 3.4 Hz), 3.46–3.36 (1H, m), 3.39 (0.5H, t, *J* = 9.5 Hz), 3.28 (0.5H, t, *J* = 9.3 Hz), 2.97 (0.5H, br d, *J* = 2.1 Hz), 2.94 (0.5H, br d, *J* = 2.1 Hz), 1.62–1.52 (1H, m), 1.52–1.40 (1H, m), 1.15 (3H, d, *J* = 6.1 Hz), 0.92 (1.5H, t, *J* = 7.5 Hz), 0.91 (1.5H, t, *J* = 7.5 Hz); ^{13}C NMR (101 MHz, $CDCl_3$) δ 139.1, 139.0, 133.5, 128.6, 127.7 (2 × C), 77.8, 77.6, 74.0, 73.8, 72.6, 72.4, 29.3, 29.2, 19.4, 19.2, 9.9, 9.8; HRMS (APCI): Found $(M+Na)^+$ 251.0813, $C_{12}H_{17}O_2ClNa$ requires 251.0809.

Following **GP5**, but with 4-chlorostyrene (60 μL, 0.50 mmol, 5.0 equiv.) gave **4bz** (11.4 mg, 50%, d.r. = 1:1).

Methyl 2-(2-(4-chlorophenyl)-2-hydroxyethoxy)propanoate (4ca)

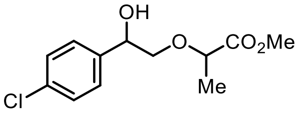

Following **GP5**, 5-((1-methoxy-1-oxopropan-2-yl)oxy)-5*H*-dibenzo[*b*,*d*]thiophen-5-ium triflate **1x** (44 mg, 0.10 mmol) and 4-chlorostyrene (60 μL, 0.50 mmol, 5.0 equiv.) gave **4ca** (18.1 mg, 70%) as an oil. R_f 0.24 [hexane–EtOAc (2:1)]; ^{1}H NMR (400 MHz, $CDCl_3$) δ 7.37–7.28 (4H, m), 4.92–4.84 (1H, m), 4.06 (1H, q, *J* = 6.9 Hz), 3.77 (1.5H, s), 3.76 (1.5H, s), 3.74–3.68 (1H, m), 3.43 (0.5H, dd, *J* = 9.8, 8.6 Hz), 3.37 (0.5H, t, *J* = 9.5 Hz), 1.45 (3H, d, *J* = 6.9 Hz); ^{13}C NMR (101 MHz, $CDCl_3$) δ 174.3, 173.9, 138.6 (2 × C), 133.7, 133.6, 128.6, 127.7 (2 × C), 76.4, 75.8, 75.4, 75.1, 72.4, 72.1, 52.4, 52.3, 18.8, 18.7; HRMS (APCI): Found $(M+Na)^+$ 281.0550, $C_{12}H_{15}O_4ClNa$ requires 281.0551.

2-(But-3-yn-2-yloxy)-1-(4-chlorophenyl)ethan-1-ol (4cb)

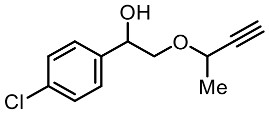

Following **GP5**, 5-(but-3-yn-2-yloxy)-5*H*-dibenzo[*b*,*d*]thiophen-5-ium triflate **1u** (40 mg, 0.10 mmol) and 4-chlorostyrene (24 μL, 0.20 mmol) gave **4cb** (15.3 mg, 68%, d.r. = 1:1) as an oil. R_f 0.28 [hexane–EtOAc (4:1)]; ^{1}H NMR (400 MHz, $CDCl_3$) δ 7.38–7.28 (4H, m), 4.94–4.83

(1H, m), 4.31–4.18 (1H, m), 3.86 (0.5H, dd, J = 9.7, 3.0 Hz), 3.69 (0.5H, dd, J = 9.7, 8.8 Hz), 3.57 (0.5H, dd, J = 9.7, 3.3 Hz), 3.36 (0.5H, t, J = 9.3 Hz), 2.82 (1H, br d, J = 2.3 Hz), 2.45 (1H, br t, J = 2.3 Hz), 1.48 (1.5H, d, J = 6.6 Hz), 1.48 (1.5H, d, J = 6.6 Hz); ^{13}C NMR (101 MHz, $CDCl_3$) δ 138.7 (2 × C), 133.7 (2 × C), 128.7 (2 × C), 127.8, 127.7, 83.3, 83.2, 74.4, 73.9, 73.8, 73.7, 72.4, 72.1, 66.2, 65.7, 22.1 (2 × C); HRMS (APCI): Found $(M+Na)^+$ 247.0499, $C_{12}H_{13}O_2ClNa$ requires 247.0496.

Following **GP5**, but with 4-chlorostyrene (60 μL, 0.50 mmol, 5.0 equiv.) gave **4cb** (18.6 mg, 80%, d.r. = 1:1) as an oil.

1-(4-Chlorophenyl)-2-((1,3-dichloropropan-2-yl)oxy)ethan-1-ol (4cc)

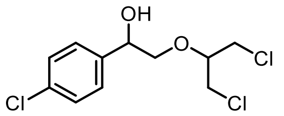

Following **GP5**, 5-((1,3-dichloropropan-2-yl)oxy)-5*H*-dibenzo[*b*,*d*]thiophen-5-ium triflate **1y** (46 mg, 0.10 mmol) and 4-chlorostyrene (24 μL, 0.20 mmol) gave **4cc** (20.7 mg, 73%) as an oil. R_f 0.24 [hexane–EtOAc (4:1)]; ^{1}H NMR (400 MHz, $CDCl_3$) δ 7.32 (4H, s), 4.89 (1H, dt, J = 8.8, 2.7 Hz), 3.86–3.79 (1H, m), 3.77 (1H, dd, J = 9.7, 3.1 Hz), 3.74–3.62 (4H, m), 3.53 (1H, dd, J = 9.7, 8.8 Hz), 2.96 (1H, br d, J = 2.4 Hz); ^{13}C NMR (101 MHz, $CDCl_3$) δ 138.2, 133.9, 128.8, 127.7, 79.8, 76.1, 72.4, 43.5, 43.4; HRMS (APCI): Found $(M+Na)^+$ 304.9874, $C_{11}H_{13}O_2Cl_3Na$ requires 304.9873.

Following **GP5**, but with 4-chlorostyrene (60 μL, 0.50 mmol, 5.0 equiv.) gave **4cc** (22.7 mg, 80%) as an oil.

1-(4-Chlorophenyl)-2-((1,3-dibromopropan-2-yl)oxy)ethan-1-ol (4cd)

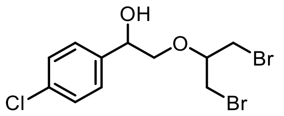

Following **GP5**, 5-((1,3-dibromopropan-2-yl)oxy)-5*H*-dibenzo[*b*,*d*]thiophen-5-ium triflate **1z** (55 mg, 0.10 mmol) and 4-chlorostyrene (24 μL, 0.20 mmol) gave **4cd** (23.8 mg, 64%) as an oil. R_f 0.21 [hexane–EtOAc (4:1)]; ^{1}H NMR (400 MHz, $CDCl_3$) δ 7.33 (4H, s), 4.90 (1H, dt, J = 8.8, 2.8 Hz), 3.83–3.75 (2H, m), 3.57 (2H, d, J = 5.4 Hz), 3.54 (2H, d, J = 5.4 Hz), 3.52 (1H, dd, J = 9.6, 8.8 Hz), 2.96 (1H, br d, J = 2.5 Hz); ^{13}C NMR (101 MHz, $CDCl_3$) δ 138.1, 133.9, 128.8, 127.7, 79.0, 76.0, 72.4, 32.7, 32.4; HRMS (APCI): Found $(M+Na)^+$ 392.8869, $C_{11}H_{13}O_2ClBr_2Na$ requires 392.8863.

Methyl 3-(2-(4-chlorophenyl)-2-hydroxyethoxy)butanoate (*rac*-4ce)

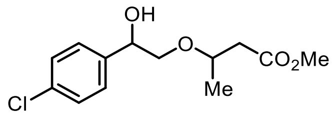

Following **GP5**, 5-((4-methoxy-4-oxobutan-2-yl)oxy)-5*H*-dibenzo[*b*,*d*]thiophen-5-ium triflate **1aa'** (45 mg, 0.10 mmol) and 4-chlorostyrene (24 µL, 0.20 mmol) gave *rac*-**4ce** (17.7 mg, 65%, d.r. = 1.1:1) as an oil. R_f 0.21 [hexane–EtOAc (2:1)]; ^{1}H NMR (400 MHz, $CDCl_3$) δ 7.35–7.28 (4H, m), 4.84 (0.52H, dt, *J* = 8.0, 2.7 Hz), 4.79 (0.48H, dd, *J* = 9.4, 2.9 Hz), 4.07–3.93 (1H, m), 3.80 (0.48H, br s), 3.74 (0.48H, dd, *J* = 10.0, 2.9 Hz), 3.71 (1.4H, s), 3.69 (1.6H, s), 3.57 (0.52H, dd, *J* = 10.3, 3.4 Hz), 3.49 (0.52H, dd, *J* = 10.3, 8.2 Hz), 3.38 (0.52H, br d, *J* = 3.2 Hz), 3.24 (0.48H, t, *J* = 9.7 Hz), 2.58 (1H, dd, *J* = 16.0, 8.6 Hz), 2.47 (0.52H, dd, *J* = 12.0, 4.2 Hz), 2.43 (0.48H, dd, *J* = 12.0, 4.4 Hz), 1.21 (1.6H, d, *J* = 5.9 Hz), 1.21 (1.4H, d, *J* = 5.9 Hz); ^{13}C NMR (101 MHz, $CDCl_3$) δ 172.6, 172.4, 139.1, 138.7, 133.5, 133.4, 128.6, 127.7, 127.6, 75.3, 73.7, 73.5, 72.8, 71.9, 71.6, 52.0, 51.9, 41.5, 41.4, 19.8, 19.5; HRMS (APCI): Found $(M+Na)^+$ 295.0714, $C_{13}H_{17}O_4ClNa$ requires 295.0708.

Methyl (3*S*)-(2-(4-chlorophenyl)-2-hydroxyethoxy)butanoate ((*S*)-4ce)

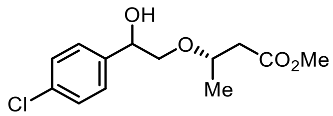

Following **GP5**, (*S*)-5-((4-methoxy-4-oxobutan-2-yl)oxy)-5*H*-dibenzo[*b*,*d*]thiophen-5-ium triflate **1aa** (45 mg, 0.10 mmol) and 4-chlorostyrene (24 µL, 0.20 mmol) gave (*S*)-**4ce** (17.5 mg, 64%, d.r. = 1.1:1) as an oil. R_f 0.21 [hexane–EtOAc (2:1)]; ^{1}H NMR (400 MHz, $CDCl_3$) δ 7.36–7.27 (4H, m), 4.83 (0.52H, dt, *J* = 8.4, 2.7 Hz), 4.79 (0.48H, dd, *J* = 9.4, 2.9 Hz), 4.07–3.93 (1H, m), 3.81 (0.48H, br s), 3.73 (0.48H, dd, *J* = 10.0, 2.9 Hz), 3.71 (1.4H, s), 3.69 (1.6H, s), 3.57 (0.52H, dd, *J* = 10.3, 3.4 Hz), 3.49 (0.52H, dd, *J* = 10.3, 8.2 Hz), 3.39 (0.52H, br s), 3.24 (0.48H, t, *J* = 9.7 Hz), 2.58 (1H, dd, *J* = 16.0, 8.7 Hz), 2.47 (0.52H, dd, *J* = 12.0, 4.2 Hz), 2.43 (0.48H, dd, *J* = 12.0, 4.4 Hz), 1.21 (1.6H, d, *J* = 5.8 Hz), 1.21 (1.4H, d, *J* = 5.8 Hz); ^{13}C NMR (101 MHz, $CDCl_3$) δ 172.6, 172.4, 139.1, 138.7, 133.4 (2 × C), 128.6, 127.7, 127.6, 75.3, 73.7, 73.5, 72.8, 71.9, 71.6, 52.0, 51.9, 41.5, 41.4, 19.8, 19.5; HRMS (APCI): Found $(M+Na)^+$ 295.0709, $C_{13}H_{17}O_4ClNa$ requires 295.0708.

Following **GP5**, but with 4-chlorostyrene (60 µL, 0.50 mmol, 5.0 equiv.) gave (*S*)-**4ce** (21.3 mg, 78%, d.r. = 1.1:1) as an oil.

(4*S*)-4-(2-(4-Chlorophenyl)-2-hydroxyethoxy)dihydrofuran-2(3*H*)-one (4cf)

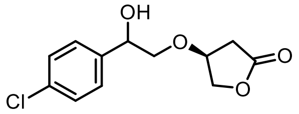

Following **GP5**, (*S*)-5-((5-oxotetrahydrofuran-3-yl)oxy)-5*H*-dibenzo[*b*,*d*]thiophen-5-ium triflate **1ab** (43.4 mg, 0.10 mmol) and 4-chlorostyrene (24 μL, 0.20 mmol) gave **4cf** (15.7 mg, 61%, d.r. = 1:1) as a solid. R_f 0.20 [hexane-EtOAc (1:3)]; ^{1}H NMR (400 MHz, $CDCl_3$) δ 7.33 (2H, d, *J* = 8.7 Hz), 7.30 (2H, d, *J* = 8.7 Hz), 4.85 (1H, dd, *J* = 8.4, 3.3 Hz), 4.44–4.32 (3H, m), 3.59 (1H, dd, *J* = 9.6, 3.3 Hz), 3.44 (1H, td, *J* = 9.1, 3.6 Hz), 2.70 (1H, dt, *J* = 17.9, 5.3 Hz), 2.60 (1H, ddd, *J* = 17.9, 11.0, 2.0 Hz), 2.37 (1H, br s); ^{13}C NMR (101 MHz, $CDCl_3$) δ 175.3 (2 × C), 138.4, 138.3, 134.0, 128.8, 127.6, 75.5, 74.7, 74.6, 73.2, 72.9, 72.3 (2 × C), 35.1, 35.0; IR (neat, cm^{-1}): 3433, 2921, 1770, 1597, 1490, 1465, 1403, 1376, 1342, 1227, 1166; HRMS (APCI): Found $(M+Na)^+$ 279.0400, $C_{12}H_{13}O_4ClNa$ requires 279.0395.
The stereocentre in the alcohol portion of the lactone in **4cf** is a single absolute stereochemistry, as characterised by X-ray crystallographic analysis. CCDC: 2432693.

1-(4-Chlorophenyl)-2-(cyclohexyloxy)ethan-1-ol (4cg)

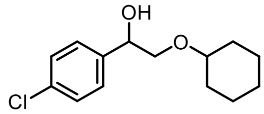

Following **GP5**, 5-(cyclohexyloxy)-5*H*-dibenzo[*b*,*d*]thiophen-5-ium triflate **1ac** (43 mg, 0.10 mmol) and 4-chlorostyrene (24 μL, 0.20 mmol) gave **4cg** (10.2 mg, 40%) as an oil. R_f 0.25 [hexane–EtOAc (7:1)]; ^{1}H NMR (500 MHz, $CDCl_3$) δ 7.31 (4H, s), 4.81 (1H, dd, *J* = 9.1, 3.3 Hz), 3.61 (1H, dd, *J* = 9.6, 3.3 Hz), 3.35 (1H, t, *J* = 9.3 Hz), 3.35–3.29 (1H, m), 3.03 (1H, br s), 1.95–1.85 (2H, m), 1.76–1.69 (2H, m), 1.58–1.49 (1H, m), 1.36–1.19 (5H, m); ^{13}C NMR (126 MHz, $CDCl_3$) δ 139.1, 133.5, 128.6, 127.7, 78.3, 73.5, 72.4, 32.3, 32.2, 25.8, 24.1; HRMS (APCI): Found $(M+Na)^+$ 277.0970, $C_{14}H_{19}O_2ClNa$ requires 277.0966.
Following **GP5**, but with 4-chlorostyrene (60 μL, 0.50 mmol, 5.0 equiv.) gave **4cg** (14.3 mg, 56%) as an oil.

1-(4-Chlorophenyl)-2-((4,4-difluorocyclohexyl)oxy)ethan-1-ol (4ch)

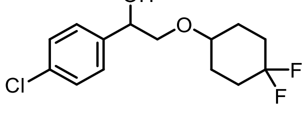

Following **GP5**, 5-((4,4-difluorocyclohexyl)oxy)-5*H*-dibenzo[*b*,*d*]thiophen-5-ium triflate **1ad** (47 mg, 0.10 mmol) and 4-chlorostyrene (24 μL, 0.20 mmol) gave **4ch** (18.3 mg, 63%) as an

oil. R_f 0.16 [hexane–EtOAc (4:1)]; ^{1}H NMR (400 MHz, $CDCl_3$) δ 7.32 (4H, s), 4.83 (1H, ddd, *J* = 8.7, 3.4, 2.0 Hz), 3.58 (1H, dd, *J* = 9.4, 3.4 Hz), 3.57–3.52 (1H, m), 3.38 (1H, dd, *J* = 9.4, 8.7 Hz), 2.83 (1H, br d, *J* = 2.0 Hz), 2.12–1.93 (2H, m), 1.92–1.74 (6H, m); ^{13}C NMR (101 MHz, $CDCl_3$) δ 138.8, 133.7, 128.7, 127.6, 123.1 (t, $J_{C\text{-}F}$ = 241.0 Hz), 73.9, 73.8, 72.5, 30.0 (t, $J_{C\text{-}F}$ = 24.7 Hz), 29.9 (t, $J_{C\text{-}F}$ = 24.7 Hz), 27.3 (td, $J_{C\text{-}F}$ = 6.8 Hz), 27.2 (t, $J_{C\text{-}F}$ = 6.8 Hz); ^{19}F NMR (376 MHz, $CDCl_3$) δ −93.9 (d, *J* = 236.8 Hz), −100.5 (d, *J* = 236.8 Hz); HRMS (APCI): Found $(M+Na)^+$ 313.0781, $C_{14}H_{17}O_2ClF_2Na$ requires 313.0777.

Following **GP5**, but with 4-chlorostyrene (60 μL, 0.50 mmol, 5.0 equiv.) gave **4ch** (23.8 mg, 82%) as an oil.

1-(4-Chlorophenyl)-2-((tetrahydro-2H-pyran-4-yl)oxy)ethan-1-ol (4ci)

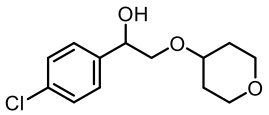

Following **GP5**, 5-((tetrahydro-2*H*-pyran-4-yl)oxy)-5*H*-dibenzo[*b*,*d*]thiophen-5-ium triflate **1ae** (43 mg, 0.10 mmol) and 4-chlorostyrene (24 μL, 0.20 mmol) gave **4ci** (15.4 mg, 60%) as an oil. R_f 0.10 [hexane–EtOAc (1:1)]; ^{1}H NMR (400 MHz, $CDCl_3$) δ 7.32 (4H, s), 4.83 (1H, dd, *J* = 9.0, 3.3 Hz), 3.92 (2H, dt, *J* = 11.5, 4.5 Hz), 3.62 (1H, dd, *J* = 9.5, 3.3 Hz), 3.55 (1H, tt, *J* = 8.7, 4.0 Hz), 3.48–3.41 (2H, m), 3.40 (1H, t, *J* = 9.2 Hz), 2.93 (1H, br s), 1.96–1.83 (2H, m), 1.66–1.53 (2H, m); ^{13}C NMR (101 MHz, $CDCl_3$) δ 138.9, 133.7, 128.7, 127.7, 74.8, 73.3, 72.4, 65.7, 65.7, 32.5, 32.4; HRMS (APCI): Found $(M+Na)^+$ 279.0759, $C_{13}H_{17}O_3ClNa$ requires 279.0758.

Following **GP5**, but with 4-chlorostyrene (60 μL, 0.50 mmol, 5.0 equiv.) gave **4ci** (17.2 mg, 67%) as an oil.

4-(2-(4-Chlorophenyl)-2-hydroxyethoxy)cyclohexan-1-one (4cj)

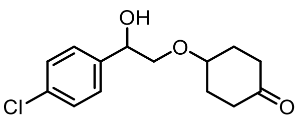

Following **GP5**, 5-((4-oxocyclohexyl)oxy)-5*H*-dibenzo[*b*,*d*]thiophen-5-ium triflate **1af** (45 mg, 0.10 mmol) and 4-chlorostyrene (24 μL, 0.20 mmol) gave **4cj** (9.4 mg, 35%) as an oil. R_f 0.24 [hexane–EtOAc (1:1)]; ^{1}H NMR (400 MHz, $CDCl_3$) δ 7.33 (4H, s), 4.88 (1H, dd, *J* = 8.5, 3.4 Hz), 3.79 (1H, tt, *J* = 5.9, 3.0 Hz), 3.66 (1H, dd, *J* = 9.5, 3.4 Hz), 3.47 (1H, dd, *J* = 9.5, 8.5 Hz), 2.84 (1H, br s), 2.53 (2H, ddd, *J* = 15.5, 10.1, 5.8 Hz), 2.27 (2H, dt, *J* = 14.9, 5.7 Hz), 2.14–2.04 (2H, m), 2.02–1.91 (2H, m); ^{13}C NMR (101 MHz, $CDCl_3$) δ 210.8, 138.9, 133.8,

128.7, 127.6, 74.0, 73.8, 72.5, 37.2, 37.1, 30.6, 30.5; HRMS (APCI): Found $(M+Na)^+$ 291.0762, $C_{14}H_{17}O_3ClNa$ requires 291.0758.

Following **GP5**, but with 4-chlorostyrene (60 μL, 0.50 mmol, 5.0 equiv.) gave **4cj** (11.8 mg, 44%) as an oil.

1-(4-Chlorophenyl)-2-(cycloheptyloxy)ethan-1-ol (4ck)

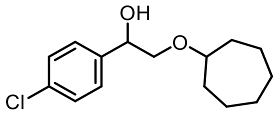

Following **GP5**, 5-(cycloheptyloxy)-5*H*-dibenzo[*b*,*d*]thiophen-5-ium triflate **1ag** (45 mg, 0.10 mmol) and 4-chlorostyrene (60 μL, 0.50 mmol, 5.0 equiv.) gave **4ck** (4.0 mg, 15%) as an oil. R_f 0.24 [hexane–EtOAc (10:1)]; ^{1}H NMR (400 MHz, $CDCl_3$) δ 7.32 (4H, s), 4.80 (1H, ddd, *J* = 9.0, 3.3, 1.7 Hz), 3.58 (1H, dd, *J* = 9.6, 3.3 Hz), 3.50 (1H, tt, *J* = 7.9, 4.2 Hz), 3.31 (1H, t, *J* = 9.3 Hz), 2.94 (1H, br d, *J* = 1.7 Hz), 1.95–1.81 (2H, m), 1.70–1.56 (4H, m), 1.57–1.50 (4H, m), 1.44–1.33 (2H, m); ^{13}C NMR (101 MHz, $CDCl_3$) δ 139.1, 133.5, 128.6, 127.7, 81.0, 73.9, 72.5, 34.0 (2 × C), 28.5 (2 × C), 23.0 (2 × C); HRMS (APCI): Found $(M+Na)^+$ 291.1123, $C_{15}H_{21}O_2ClNa$ requires 291.1122.

2-((Adamantan-2-yl)oxy)-1-(4-chlorophenyl)ethan-1-ol (4cl)

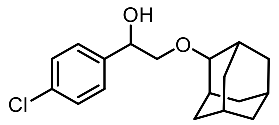

Following **GP5**, 5-((adamantan-2-yl)oxy)-5*H*-dibenzo[*b*,*d*]thiophen-5-ium triflate **1ah** (49 mg, 0.10 mmol) and 4-chlorostyrene (24 μL, 0.20 mmol) gave **4cl** (10.4 mg, 34%) as a solid. R_f 0.24 [hexane–EtOAc (10:1)]; ^{1}H NMR (400 MHz, $CDCl_3$) δ 7.34 (2H, d, *J* = 8.8 Hz), 7.31 (2H, d, *J* = 8.8 Hz), 4.85 (1H, ddd, *J* = 8.9, 3.3, 2.0 Hz), 3.60 (1H, dd, *J* = 9.4, 3.3 Hz), 3.50 (1H, t, *J* = 3.3 Hz), 3.37 (1H, t, *J* = 9.2 Hz), 3.05 (1H, br d, *J* = 2.0 Hz), 2.06–1.96 (4H, m), 1.90–1.76 (4H, m), 1.74–1.69 (2H, m), 1.68–1.61 (2H, m), 1.54–1.45 (2H, m); ^{13}C NMR (101 MHz, $CDCl_3$) δ 139.2, 133.5, 128.6, 127.7, 82.5, 73.1, 72.5, 39.4, 37.6, 36.5, 32.1, 31.8, 31.7 (2 × C), 27.5 (2 × C); HRMS (APCI): Found $(M+Na)^+$ 329.1281, $C_{18}H_{23}O_2ClNa$ requires 329.1279.

Following **GP5**, but with 4-chlorostyrene (60 μL, 0.50 mmol, 5.0 equiv.) gave **4cl** (15.3 mg, 50%) as a solid.

2-((Adamantan-1-yl)oxy)-1-(4-chlorophenyl)ethan-1-ol (4cm)

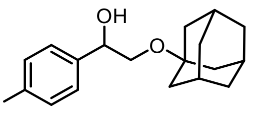

Following **GP5**, 5-((adamantan-1-yl)oxy)-5*H*-dibenzo[*b*,*d*]thiophen-5-ium triflate **1ai** (49 mg, 0.10 mmol) and 4-chlorostyrene (24 μL, 0.20 mmol) gave **4cm** (7.4 mg, 24%) as a solid. R_f 0.33 [hexane–EtOAc (4:1)]; ^{1}H NMR (400 MHz, $CDCl_3$) δ 7.32 (4H, s), 4.76 (1H, dd, *J* = 9.1, 3.3 Hz), 3.54 (1H, dd, *J* = 9.2, 3.3 Hz), 3.33 (1H, t, *J* = 9.2 Hz), 3.03 (1H, br s), 2.20–2.12 (3H, m), 1.81–1.69 (6H, m), 1.67–1.56 (6H, m); ^{13}C NMR (101 MHz, $CDCl_3$) δ 139.3, 133.5, 128.6, 127.7, 73.1, 72.7, 65.9, 41.8, 36.5, 30.6; HRMS (APCI): Found $(M+Na)^+$ 329.1281, $C_{18}H_{23}O_2ClNa$ requires 329.1279.

Following **GP5**, but with 4-chlorostyrene (60 μL, 0.50 mmol, 5.0 equiv.) gave **4cm** (11.4 mg, 37%) as a solid.

Substrate scope of 1,2-dialkoxylation

1-Chloro-4-(1,2-dimethoxyethyl)benzene (5a)

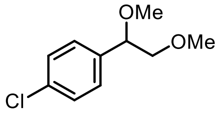

Following **GP6**, 5-methoxy-5*H*-dibenzo[*b*,*d*]thiophen-5-ium triflate **1a** (36 mg, 0.10 mmol), 4-chlorostyrene (24 μL, 0.20 mmol) and MeOH (41 μL, 0.50 mmol) gave **5a** (12.8 mg, 64%) as an oil. ^{1}H NMR (400 MHz, $CDCl_3$) δ 7.33 (2H, d, *J* = 8.5 Hz), 7.26 (2H, d, *J* = 8.5 Hz), 4.35 (1H, dd, *J* = 7.9, 3.7 Hz), 3.55 (1H, dd, *J* = 10.4, 7.9 Hz), 3.38 (1H, dd, *J* = 10.4, 3.7 Hz), 3.37 (3H, s), 3.28 (3H, s); ^{13}C NMR (101 MHz, $CDCl_3$) δ 137.6, 133.9, 128.8, 128.5, 82.4, 77.1, 59.4, 57.2. Data in accordance with the literature.[15]

1-(1-(3-Bromopropoxy)-2-methoxyethyl)-4-chlorobenzene (5b)

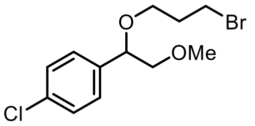

Following **GP6**, 5-methoxy-5*H*-dibenzo[*b*,*d*]thiophen-5-ium triflate **1a** (36 mg, 0.10 mmol), 4-chlorostyrene (24 μL, 0.20 mmol) and 3-bromopropan-1-ol (45 μL, 0.50 mmol) gave **5b** (19.6 mg, 64%) as an oil. R_f 0.34 [hexane-EtOAc (4:1)]; ^{1}H NMR (400 MHz, $CDCl_3$) δ 7.33 (2H, d, *J* = 8.5 Hz), 7.26 (2H, d, *J* = 8.5 Hz), 4.45 (1H, dd, *J* = 7.5, 4.0 Hz), 3.64–3.45 (5H, m), 3.41 (1H, dd, *J* = 10.5, 4.0 Hz), 3.37 (3H, s), 2.26–1.97 (2H, m); ^{13}C NMR (101 MHz, $CDCl_3$) δ 138.0, 133.9, 128.8, 128.4, 81.1, 77.1, 66.8, 59.5, 33.0, 30.7; IR (neat, cm^{-1}): 2922, 2875,

2825, 1595, 1489, 1450, 1411, 1341, 1285, 1257, 1198; HRMS (APCI): Found $(M+Na)^+$ 328.9917, $C_{12}H_{16}O_2BrClNa$ requires 328.9914.

(2-(1-(4-Chlorophenyl)-2-methoxyethoxy)ethyl)trimethylsilane (5c)

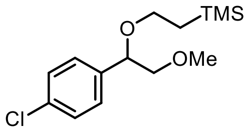

Following **GP6**, 5-methoxy-5*H*-dibenzo[*b*,*d*]thiophen-5-ium triflate **1a** (36 mg, 0.10 mmol), 4-chlorostyrene (24 μL, 0.20 mmol) and 2-(trimethylsilyl)ethan-1-ol (71 μL, 0.50 mmol) gave **5c** (13.5 mg, 47%) as an oil. R_f 0.20 [hexane-EtOAc (10:1)]; ^{1}H NMR (400 MHz, $CDCl_3$) δ 7.32 (2H, d, *J* = 8.5 Hz), 7.27 (2H, d, *J* = 8.5 Hz), 4.44 (1H, dd, *J* = 7.4, 4.2 Hz), 3.56 (1H, dd, *J* = 10.2, 7.4 Hz), 3.45–3.36 (3H, m), 3.36 (3H, s), 1.02 (1H, ddd, *J* = 13.8, 9.8, 7.0 Hz), 0.91 (1H, ddd, *J* = 13.8, 9.7, 7.1 Hz), −0.04 (9H, s); ^{13}C NMR (101 MHz, $CDCl_3$) δ 138.7, 133.6, 128.7, 128.4, 80.2, 77.3, 66.6, 59.4, 18.6, −1.3; IR (neat, cm^{-1}): 2952, 2918, 2889, 1489, 1248, 1195, 1129; HRMS (APCI): Found $(M+Na)^+$ 309.1044, $C_{14}H_{23}O_2ClNaSi$ requires 309.1048.

1-Chloro-4-(2-methoxy-1-(2-methoxyethoxy)ethyl)benzene (5d)

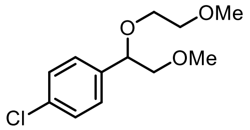

Following **GP6**, 5-methoxy-5*H*-dibenzo[*b*,*d*]thiophen-5-ium triflate **1a** (36 mg, 0.10 mmol), 4-chlorostyrene (24 μL, 0.20 mmol) and 2-methoxyethan-1-ol (39 μL, 0.50 mmol) gave **5d** (14.4 mg, 59%) as an oil. R_f 0.14 [hexane-EtOAc (2:1)]; ^{1}H NMR (400 MHz, $CDCl_3$) δ 7.32 (2H, d, *J* = 8.7 Hz), 7.28 (2H, d, *J* = 8.7 Hz), 4.50 (1H, dd, *J* = 7.4, 4.3 Hz), 3.61 (1H, dd, *J* = 10.4, 7.4 Hz), 3.56–3.49 (4H, m), 3.42 (1H, dd, *J* = 10.4, 4.3 Hz), 3.36 (3H, s), 3.34 (3H, s); ^{13}C NMR (101 MHz, $CDCl_3$) δ 138.1, 133.8, 128.8, 128.5, 81.1, 77.0, 72.1, 68.6, 59.4, 59.1; IR (neat, cm^{-1}): 2981, 2876, 2824, 1596, 1489, 1453, 1408, 1374, 1339, 1296, 1242, 1197; HRMS (APCI): Found $(M+Na)^+$ 267.0757, $C_{12}H_{17}O_3ClNa$ requires 267.0758.

1-(1-(Benzyloxy)-2-methoxyethyl)-4-chlorobenzene (5e)

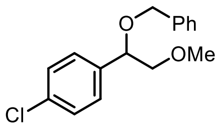

Following **GP6**, 5-methoxy-5*H*-dibenzo[*b*,*d*]thiophen-5-ium triflate **1a** (36 mg, 0.10 mmol), 4-chlorostyrene (24 μL, 0.20 mmol) and phenylmethanol (52 μL, 0.50 mmol) gave **5e** (16.5

mg, 60%) as an oil. R_f 0.23 [hexane-EtOAc (10:1)]; ^{1}H NMR (400 MHz, $CDCl_3$) δ 7.37–7.27 (9H, m), 4.55 (1H, dd, *J* = 7.3, 4.3 Hz), 4.53 (1H, d, *J* = 11.9 Hz), 4.37 (1H, d, *J* = 11.9 Hz), 3.64 (1H, dd, *J* = 10.4, 7.3 Hz), 3.46 (1H, dd, *J* = 10.4, 4.3 Hz), 3.36 (3H, s); ^{13}C NMR (101 MHz, $CDCl_3$) δ 138.1, 138.0, 133.9, 128.9, 128.7, 128.5, 127.9, 127.8, 79.7, 77.1, 70.9, 59.5; IR (neat, cm^{-1}): 3063, 3030, 2924, 2876, 1597, 1489, 1454, 1409, 1392, 1341, 1294, 1260, 1198, 1129; HRMS (APCI): Found $(M+Na)^+$ 299.0805, $C_{16}H_{17}O_2ClNa$ requires 299.0797.

Methyl (2*S*)-3-(1-(4-chlorophenyl)-2-methoxyethoxy)-2-(1,3-dioxoisoindolin-2-yl)propanoate (5f)

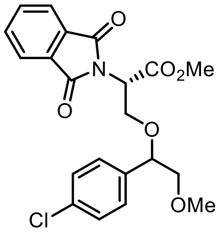

Following **GP6**, 5-methoxy-5*H*-dibenzo[*b*,*d*]thiophen-5-ium triflate **1a** (36 mg, 0.10 mmol), 4-chlorostyrene (24 μL, 0.20 mmol) and methyl (*S*)-2-(1,3-dioxoisoindolin-2-yl)-3-hydroxypropanoate (0.13 g, 0.50 mmol) gave **5f** (22.6 mg, 54%, d.r. = 1:1) as an oil. R_f 0.24 [hexane-EtOAc (2:1)]; ^{1}H NMR (400 MHz, $CDCl_3$) δ 7.89 (1H, dd, *J* = 5.5, 3.1 Hz), 7.82 (1H, dd, *J* = 5.5, 3.1 Hz), 7.78–7.72 (2H, m), 7.29 (1H, d, *J* = 8.5 Hz), 7.22 (1H, d, *J* = 8.5 Hz), 7.06 (2H, s), 5.19 (0.5H, dd, *J* = 10.1, 5.0 Hz), 5.14 (0.5H, dd, *J* = 9.5, 5.9 Hz), 4.44 (0.5H, dd, *J* = 7.4, 4.0 Hz), 4.37 (0.5H, dd, *J* = 7.6, 4.1 Hz), 4.21 (0.5H, dd, *J* = 10.9, 5.0 Hz), 4.17–4.07 (1.5H, m), 3.71 (1.5H, s), 3.71 (1.5H, s), 3.44 (0.5H, dd, *J* = 10.5, 7.4 Hz), 3.35 (0.5H, dd, *J* = 10.7, 7.6 Hz), 3.32 (0.5H, dd, *J* = 10.5, 4.0 Hz), 3.26 (0.5H, dd, *J* = 10.7, 4.1 Hz), 3.24 (1.5H, s), 3.01 (1.5H, s); ^{13}C NMR (101 MHz, $CDCl_3$) δ 167.9 (2 × C), 167.8, 167.5, 137.6, 137.4, 134.3, 134.2, 134.0, 133.9, 132.1, 132.0, 128.8, 128.6, 128.3 (2 × C), 123.6 (2 × C), 82.1, 81.2, 76.8, 76.7, 66.1 (2 × C), 59.4, 59.1, 52.9 (2 × C), 52.1, 51.6; IR (neat, cm^{-1}): 3019, 2926, 1778, 1747, 1716, 1490, 1469, 1437, 1391, 1289, 1263, 1215; HRMS (APCI): Found $(M+Na)^+$ 440.0869, $C_{21}H_{20}O_6NClNa$ requires 440.0871.

1-Chloro-4-(1-cyclobutoxy-2-methoxyethyl)benzene (5g)

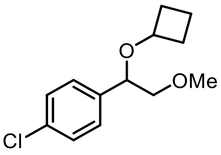

Following **GP6**, 5-methoxy-5*H*-dibenzo[*b*,*d*]thiophen-5-ium triflate **1a** (36 mg, 0.10 mmol), 4-chlorostyrene (24 μL, 0.20 mmol) and cyclobutanol (39 μL, 0.5 mmol) gave **5g** (11.8 mg,

49%) as an oil. R_f 0.14 [hexane-EtOAc (10:1)]; ^{1}H NMR (400 MHz, $CDCl_3$) δ 7.31 (2H, d, *J* = 8.6 Hz), 7.26 (2H, d, *J* = 8.6 Hz), 4.42 (1H, dd, *J* = 7.4, 4.3 Hz), 3.96–3.76 (1H, m), 3.57 (1H, dd, *J* = 10.3, 7.4 Hz), 3.39 (1H, dd, *J* = 10.3, 4.3 Hz), 3.36 (3H, s), 2.21–2.11 (1H, m), 2.02–1.89 (3H, m), 1.66–1.59 (1H, m), 1.43–1.31 (1H, m); ^{13}C NMR (101 MHz, $CDCl_3$) δ 138.7, 133.7, 128.6 (2 × C), 78.1, 77.0, 72.0, 59.4, 31.1, 30.7, 12.6; IR (neat, cm^{-1}): 2981, 2937, 2875, 2827, 1488, 1467, 1409, 1382, 1336, 1238, 1194, 1142, 1110; HRMS (APCI): Found $(M+Na)^+$ 263.0806, $C_{13}H_{17}O_2ClNa$ requires 263.0797.

1-(1-(*tert*-Butoxy)-2-methoxyethyl)-4-chlorobenzene (5h)

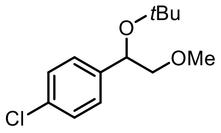

Following **GP6**, 5-methoxy-5*H*-dibenzo[*b*,*d*]thiophen-5-ium triflate **1a** (36 mg, 0.10 mmol), 4-chlorostyrene (24 μL, 0.20 mmol) and *tert*-butanol (47 μL, 0.50 mmol) gave **5h** (8.7 mg, 36%) as an oil. R_f 0.40 [hexane-EtOAc (10:1)]; ^{1}H NMR (400 MHz, $CDCl_3$) δ 7.31 (2H, d, *J* = 8.8 Hz), 7.28 (2H, d, *J* = 8.8 Hz), 4.61 (1H, dd, *J* = 7.1, 5.1 Hz), 3.45 (1H, dd, *J* = 10.2, 7.1 Hz), 3.33 (3H, s), 3.31 (1H, dd, *J* = 10.2, 5.1 Hz), 1.15 (9H, s); ^{13}C NMR (101 MHz, $CDCl_3$) δ 142.0, 132.9, 128.4, 128.0, 78.2, 74.9, 72.8, 59.5, 28.7; IR (neat, cm^{-1}): 2974, 2923, 2824, 1489, 1470, 1406, 1389, 1365, 1252, 1233, 1192, 1127, 1105; HRMS (APCI): Found $(M+Na)^+$ 265.0959, $C_{13}H_{19}O_2ClNa$ requires 265.0966.

Substrate scope of 1,2-alkoxy-amidation

***N*-(1-(4-Chlorophenyl)-2-methoxyethyl)acetamide (6a)**

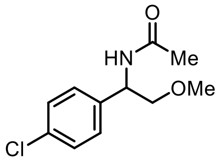

Following **GP7**, 5-methoxy-5*H*-dibenzo[*b*,*d*]thiophen-5-ium triflate **1a** (36 mg, 0.10 mmol), 4-chlorostyrene (24 μL, 0.20 mmol) and acetonitrile gave **6a** (14.3 mg, 63%) as a solid. ^{1}H NMR (500 MHz, $CDCl_3$) δ 7.29 (2H, d, *J* = 8.6 Hz), 7.26 (2H, d, *J* = 8.6 Hz), 6.22 (1H, br d, *J* = 7.6 Hz), 5.10 (1H, dt, *J* = 7.6, 4.6 Hz), 3.64 (1H, dd, *J* = 9.8, 4.4 Hz), 3.60 (1H, dd, *J* = 9.8, 4.7 Hz), 3.35 (3H, s), 2.03 (3H, s); ^{13}C NMR (126 MHz, $CDCl_3$) δ 169.7, 138.7, 133.4, 128.8, 128.3, 74.9, 59.3, 52.2, 23.5. Data in accordance with the literature.[15]

_N_-(1-(4-Chlorophenyl)-2-methoxyethyl)butyramide (6b)

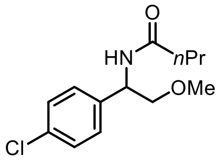

Following **GP7**, 5-methoxy-5*H*-dibenzo[*b*,*d*]thiophen-5-ium triflate **1a** (36 mg, 0.10 mmol), 4-chlorostyrene (24 µL, 0.20 mmol) and butyronitrile gave **6b** (14.1 mg, 55%) as an oil. R_f 0.34 [CH_2Cl_2–MeOH (20:1)]; ^{1}H NMR (400 MHz, $CDCl_3$) δ 7.29 (2H, d, *J* = 8.6 Hz), 7.25 (2H, d, *J* = 8.6 Hz), 6.18 (1H, br d, *J* = 7.6 Hz), 5.12 (1H, dt, *J* = 7.6, 4.5 Hz), 3.64 (1H, dd, *J* = 9.8, 4.4 Hz), 3.61 (1H, dd, *J* = 9.8, 4.6 Hz), 3.34 (3H, s), 2.24–2.17 (2H, m), 1.67 (2H, h, *J* = 7.4 Hz), 0.94 (3H, t, *J* = 7.4 Hz); ^{13}C NMR (101 MHz, $CDCl_3$) δ 172.7, 138.9, 133.3, 128.8, 128.3, 74.9, 59.2, 52.0, 38.8, 19.2, 13.9; HRMS (APCI): Found $(M+Na)^+$ 278.0923, $C_{13}H_{18}O_2NClNa$ requires 278.0918.

2-Chloro-_N_-(1-(4-chlorophenyl)-2-methoxyethyl)acetamide (6c)

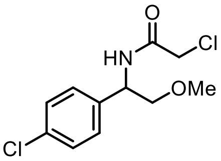

Following **GP7**, 5-methoxy-5*H*-dibenzo[*b*,*d*]thiophen-5-ium triflate **1a** (36 mg, 0.10 mmol), 4-chlorostyrene (24 µL, 0.20 mmol) and chloroacetonitrile gave **6c** (7.3 mg, 28%) as an solid. R_f 0.32 [CH_2Cl_2–MeOH (20:1)]; ^{1}H NMR (400 MHz, $CDCl_3$) δ 7.33 (1H, br d, *J* = 7.9 Hz), 7.30 (2H, d, *J* = 8.6 Hz), 7.26 (2H, d, *J* = 8.6 Hz), 5.09 (1H, dt, *J* = 7.9, 4.6 Hz), 4.08 (1H, d, *J* = 15.2 Hz), 4.03 (1H, d, *J* = 15.2 Hz), 3.66 (1H, dd, *J* = 9.8, 4.4 Hz), 3.63 (1H, dd, *J* = 9.8, 4.8 Hz), 3.36 (3H, s); ^{13}C NMR (101 MHz, $CDCl_3$) δ 165.7, 137.8, 133.6, 128.8, 128.3, 74.6, 59.3, 52.6, 42.7; HRMS (APCI): Found $(M+H)^+$ 262.0397, $C_{11}H_{14}O_2NCl_2$ requires 262.0396.

_N_-(1-(4-Chlorophenyl)-2-methoxyethyl)-2-phenylacetamide (6d)

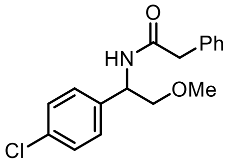

Following **GP7**, 5-methoxy-5*H*-dibenzo[*b*,*d*]thiophen-5-ium triflate **1a** (36 mg, 0.10 mmol), 4-chlorostyrene (24 µL, 0.20 mmol) and phenylacetonitrile gave **6d** (7.6 mg, 25%) as a solid. R_f 0.32 [CH_2Cl_2–MeOH (20:1)]; ^{1}H NMR (400 MHz, $CDCl_3$) δ 7.41–7.27 (5H, m), 7.25 (2H, d, *J* = 8.4 Hz), 7.12 (2H, d, *J* = 8.4 Hz), 6.12 (1H, br d, *J* = 7.7 Hz), 5.09 (1H, dt, *J* = 8.3, 4.6

Hz), 3.61 (2H, s), 3.54 (2H, d, *J* = 4.6 Hz), 3.25 (3H, s); ^{13}C NMR (101 MHz, $CDCl_3$) δ 170.7, 138.5, 134.9, 129.4, 129.1, 128.7, 128.2, 127.5, 122.1, 74.8, 59.2, 52.2, 43.9; HRMS (APCI): Found $(M+H)^+$ 304.1096, $C_{17}H_{19}O_2NCl$ requires 304.1099.

***N*-(1-(4-Chlorophenyl)-2-methoxyethyl)isobutyramide (6e)**

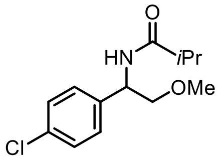

Following **GP7**, 5-methoxy-5*H*-dibenzo[*b*,*d*]thiophen-5-ium triflate **1a** (36 mg, 0.10 mmol), 4-chlorostyrene (24 µL, 0.20 mmol) and isobutyronitrile gave **6e** (10.7 mg, 42%) as a solid. R_f 0.26 [CH_2Cl_2–MeOH (20:1)]; ^{1}H NMR (400 MHz, $CDCl_3$) δ 7.29 (2H, d, *J* = 8.8 Hz), 7.24 (2H, d, *J* = 8.8 Hz), 6.20 (1H, br d, *J* = 7.5 Hz), 5.09 (1H, dt, *J* = 7.5, 4.5 Hz), 3.64 (1H, dd, *J* = 9.8, 4.5 Hz), 3.61 (1H, dd, *J* = 9.8, 4.6 Hz), 3.35 (3H, s), 2.42 (1H, hept, *J* = 7.0 Hz), 1.17 (3H, d, *J* = 7.0 Hz), 1.16 (3H, d, *J* = 7.0 Hz); ^{13}C NMR (101 MHz, $CDCl_3$) δ 176.6, 138.9, 133.3, 128.8, 128.3, 74.9, 59.3, 51.9, 35.7, 19.8, 19.6; HRMS (APCI): Found $(M+Na)^+$ 278.0921, $C_{13}H_{18}O_2NClNa$ requires 278.0918.

***N*-(1-(4-Chlorophenyl)-2-methoxyethyl)cyclopropanecarboxamide (6f)**

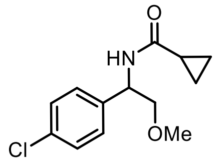

Following **GP7**, 5-methoxy-5*H*-dibenzo[*b*,*d*]thiophen-5-ium triflate **1a** (36 mg, 0.10 mmol), 4-chlorostyrene (24 µL, 0.20 mmol) and cyclopropanecarbonitrile gave **6f** (14.0 mg, 55%) as a solid. R_f 0.26 [CH_2Cl_2–MeOH (20:1)]; ^{1}H NMR (400 MHz, $CDCl_3$) δ 7.29 (2H, d, *J* = 8.8 Hz), 7.27 (2H, d, *J* = 8.8 Hz), 6.39 (1H, br d, *J* = 7.5 Hz), 5.11 (1H, dt, *J* = 7.5, 4.6 Hz), 3.65 (1H, dd, *J* = 9.8, 4.5 Hz), 3.62 (1H, dd, *J* = 9.8, 4.7 Hz), 3.36 (3H, s), 1.42 (1H, tt, *J* = 7.9, 4.6 Hz), 1.03–0.90 (2H, m), 0.79–0.69 (2H, m); ^{13}C NMR (101 MHz, $CDCl_3$) δ 173.3, 138.9, 133.3, 128.8, 128.3, 75.0, 59.3, 52.3, 14.9, 7.5 (2 × C); HRMS (APCI): Found $(M+Na)^+$ 276.0765, $C_{13}H_{16}O_2NClNa$ requires 276.0762.

***N*-(1-(4-Chlorophenyl)-2-methoxyethyl)pivalamide (6g)**

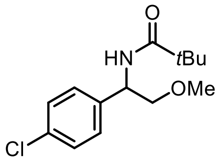

Following **GP7** but reaction run at 0 °C, 5-methoxy-5*H*-dibenzo[*b*,*d*]thiophen-5-ium triflate **1a** (36 mg, 0.10 mmol), 4-chlorostyrene (24 μL, 0.20 mmol) and trimethylacetonitrile gave **6g** (7.6 mg, 28%) as a solid. R_f 0.34 [CH_2Cl_2–MeOH (20:1)]; ^1H NMR (400 MHz, $CDCl_3$) δ 7.29 (2H, d, J = 8.6 Hz), 7.23 (2H, d, J = 8.6 Hz), 6.39 (1H, br d, J = 7.2 Hz), 5.06 (1H, dt, J = 7.2, 4.5 Hz), 3.63 (1H, dd, J = 9.8, 4.4 Hz), 3.61 (1H, dd, J = 9.8, 4.8 Hz), 3.35 (3H, s), 1.22 (9H, s); ^{13}C NMR (101 MHz, $CDCl_3$) δ 178.1, 139.1, 133.2, 128.8, 128.2, 74.9, 59.3, 52.0, 38.9, 27.7; HRMS (APCI): Found $(M+Na)^+$ 292.1079, $C_{14}H_{20}O_2NClNa$ requires 292.1075.

***N*-(1-(4-Chlorophenyl)-2-methoxyethyl)benzamide (6h)**

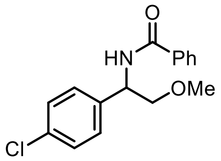

Following **GP7**, 5-methoxy-5*H*-dibenzo[*b*,*d*]thiophen-5-ium triflate **1a** (36 mg, 0.10 mmol), 4-chlorostyrene (24 μL, 0.20 mmol) and benzonitrile gave **6h** (13.0 mg, 45%) as a solid. R_f 0.34 [CH_2Cl_2–MeOH (20:1)]; ^1H NMR (400 MHz, $CDCl_3$) δ 7.85–7.78 (2H, m), 7.55–7.49 (1H, m), 7.47–7.41 (2H, m), 7.34 (2H, d, J = 8.7 Hz), 7.30 (2H, d, J = 8.7 Hz), 6.95 (1H, br d, J = 7.5 Hz), 5.30 (1H, dt, J = 7.5, 4.5 Hz), 3.75 (1H, dd, J = 9.8, 4.4 Hz), 3.72 (1H, dd, J = 9.8, 4.7 Hz), 3.39 (3H, s); ^{13}C NMR (101 MHz, $CDCl_3$) δ 167.0, 138.7, 134.3, 133.4, 131.8, 128.8, 128.7, 128.4, 127.2, 74.9, 59.3, 52.7; HRMS (APCI): Found $(M+Na)^+$ 312.0767, $C_{16}H_{16}O_2NClNa$ requires 312.0762.

Ethyl 3-benzamido-2-methoxy-3-phenylpropanoate (6i)

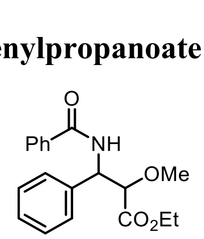

Following **GP7** but reaction run at 0 °C, 5-methoxy-5*H*-dibenzo[*b*,*d*]thiophen-5-ium triflate **1a** (36 mg, 0.10 mmol), ethyl cinnamate (24 μL, 0.20 mmol) and benzonitrile gave **6i** (12.4 mg, 38%, d.r. = 5:1) as an inseparable mixture. R_f 0.34 [CH_2Cl_2–MeOH (20:1)]; ^1H NMR (400 MHz, $CDCl_3$) δ 7.84–7.76 (m, 2H), 7.53–7.29 (m, 8H), 7.15 (d, J = 8.8 Hz, 1H), 5.72–5.61 (m, 1H), 4.27–4.20 (m, 1.66H), 4.15 (d, J = 2.2 Hz, 0.83H), 4.10–4.03 (m, 0.51H), 3.51 (s, 0.51H),

3.40 (s, 2.49H), 1.27 (t, *J* = 7.2 Hz, 2.49H), 1.10 (t, *J* = 7.2 Hz, 0.51H); ^{13}C NMR (101 MHz, $CDCl_3$) δ 170.4, 170.1, 167.0, 166.7, 139.2, 137.3, 134.4, 134.0, 131.8 (2 × C), 128.8, 128.7 (3 × C), 127.8, 127.7, 127.3, 127.2, 126.9, 126.8, 82.7, 82.4, 61.7, 61.3, 59.2 (2 × C), 54.9, 54.7, 14.3, 14.1; HRMS (APCI): Found $(M+H)^+$ 328.1538, $C_{19}H_{22}O_4N$ requires 328.1543.

5 Picture of Reaction Set-up

Pictures of set-up for 0.1 mmol scale reactions.

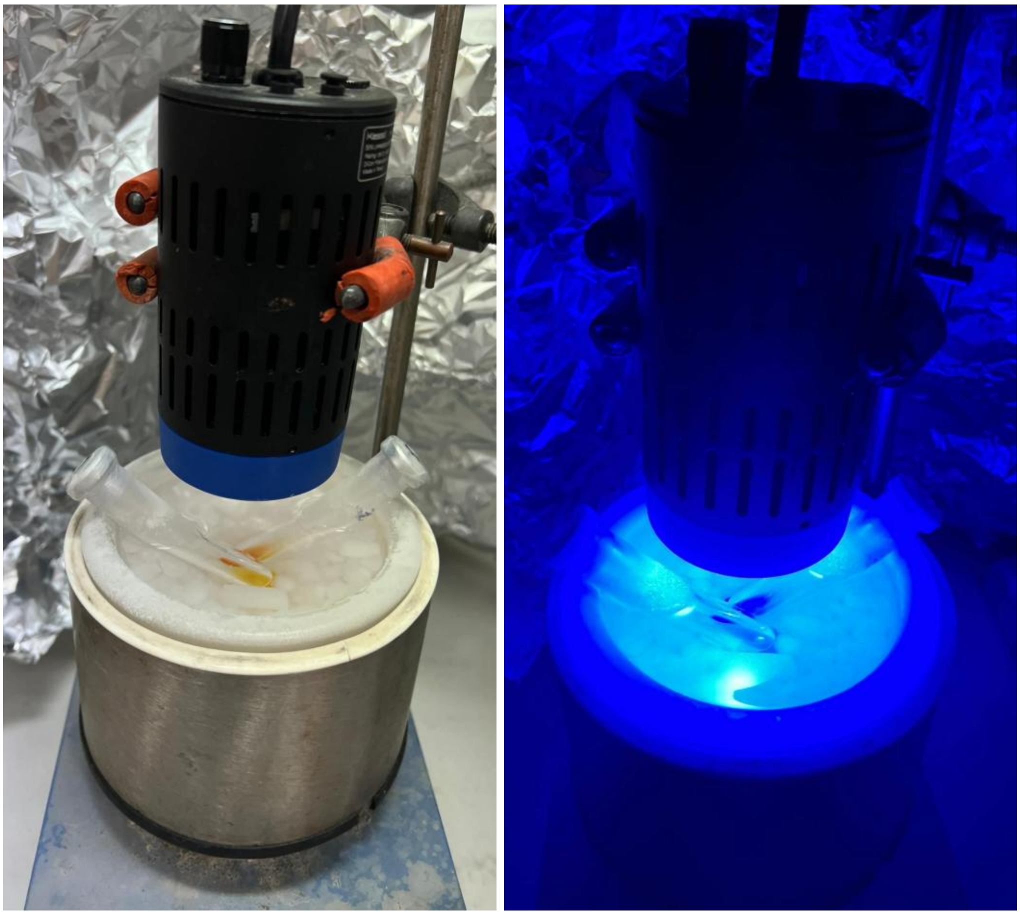

Figure S1. Set-up for 0.1 mmol scale reactions.

6 Scale-up Experiment

Scale-up experiment conducted at Phamaron UK

6.1 Synthesis of Alkoxy Sulfonium Salt 1a

Step 1:

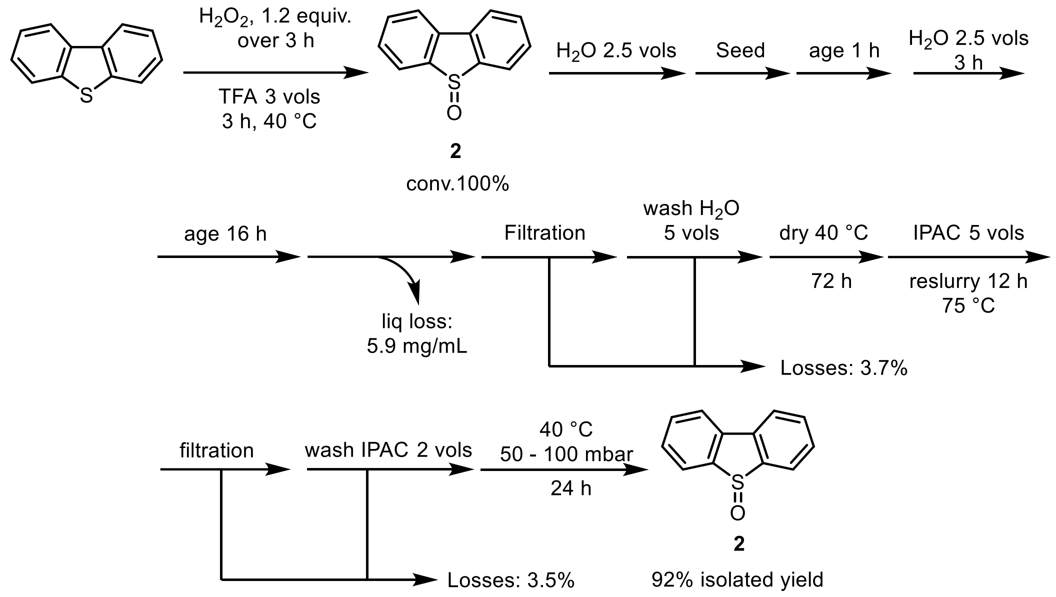

Scheme S2. Synthesis of **2**, Oxidation Step.

Dibenzothiophene (DBT) (490.00 g, 2.6 mol, 1.0 equiv.) was charged into a 5 L Radley reactor followed by TFA (1.47 L; 3 volumes, see **Figure S2**, picture A). The temperature of the jacket was regulated to keep the contents temperature at 40 °C. In the meantime, a peristaltic pump was primed with H_2O_2 30% vols. and connected to the reactor by PTFE tubing. The pump was set to dispense H_2O_2 (319.4 mL, 3.1 mol, 1.2 equiv.) over a period of 3 h while the jacket temperature was adjusted to keep the contents temperature between 40-45 °C (**Figure S2**, pictures B to E).[a] Reaction conversion was monitored by HPLC following Method A (see section 6.5), until complete disappearance of the dibenzothiophene peak. The reaction mixture was then cooled down to 20 °C and water (1.225 L, 2.5 volumes) was added. During this addition, the jacket temperature was regulated to keep the content between 20-25 °C. The reaction mixture was then seeded with 250 mg of **2** dispersed in 2 mL of water at 20 °C. The seed bed was left aging for 1 h (**Figure S2**, picture F).[b]

---

[a] **Please note:** the reaction is highly exothermic and could lead to uncontrolled TFA boil out if H_2O_2 is not carefully dispensed. The reaction is run at 40 °C to prevent any accumulation of hydrogen peroxide.

[b] **Please note:** it has been noticed that the only seed material that allows seed bed growth in the reaction mixture at this stage is a **2** batch that has not gone through IPAC reslurry. To generate this seed batch, perform a reaction on a smaller scale, adding 5 volumes of H_2O at the end of the reaction and wait for self-seeding. Reducing the reaction temperature to 10 °C should help. IPAC: isopropyl acetate.

Water (1.225 L, 2.5 vols.) was added to the reaction mixture over a period of 3 h, keeping the contents temperature at 20 °C, then the slurry was left aging for 16 h at 20 °C (**Figure S2**, picture G). A small sample was taken and filtered to measure the liquor loss at this stage (5.9 mg/mL) and the reaction was filtered using a large HDPE (High Density Polyethylene) Buckner funnel and a 30 μm filter cloth (**Figure S2**, picture H). The wet cake was washed with water (2500 mL, 5 vols). The mother liquors and the washes were collected and analysed to determine the percentage of compound **2** lost in the liquid. It was determined that 3.7% of **2** was lost in the workup. The wet cake was then transferred into glass trays and placed in a vacuum oven (50-100 mbar) for 72 h at 40 °C. 498.18 g (95% yield) of **2** was obtained as a fine white powder (**Figure S2**, picture J).

This reaction was repeated to obtain a total of 999.85 g of **2** (96% yield).

The material from the two reactions was combined and charged together in a 5 L Radley reactor followed by IPAC (isopropyl acetate, 4.2 L, 4.2 vols). The slurry was heated up to 75 °C and left aging at this temperature for 12 h. At this point the slurry was slowly cooled down to 20 °C over a period of 4 h. A sample of the slurry was taken and filtered to determine the liquor loss (1.6 mg/mL). The mixture was filtered using a large HDPE Buckner funnel and a 30 μm filter cloth. The cake was washed with IPAC (2.0 L, 2.0 vols) The mother liquors and the washes were collected and analysed to determine the percentage of **2** lost in the liquid. It was determined that 3.5% of **2** was lost in the workup. The wet cake was then transferred into glass trays and placed in a vacuum oven (50-100 mbar) for 24 h at 40 °C, to obtain **2** (955.52 g, 92% yield) as a fine white powder.

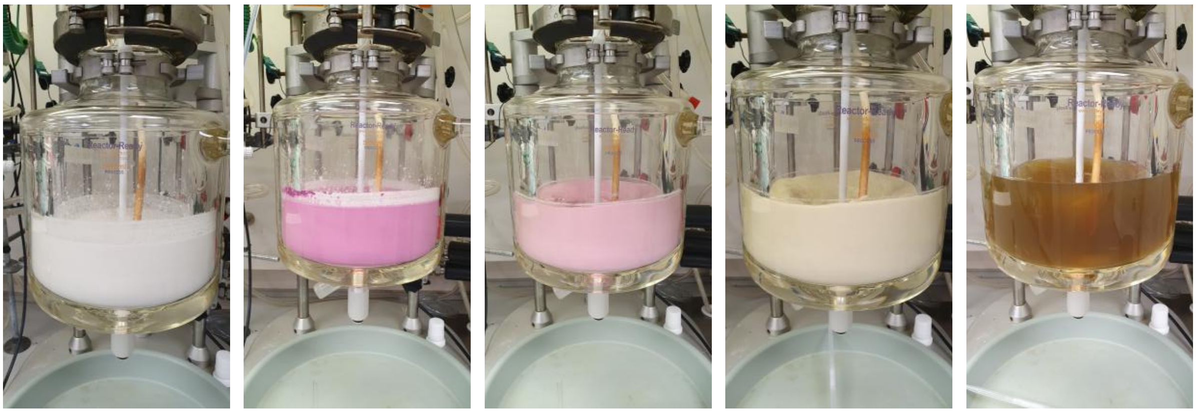

A: TFA + DBT | **B**: First drop of H_2O_2 | **C**: 30% H_2O_2 | **D**: 60% H_2O_2 | **E**: 100% H_2O_2

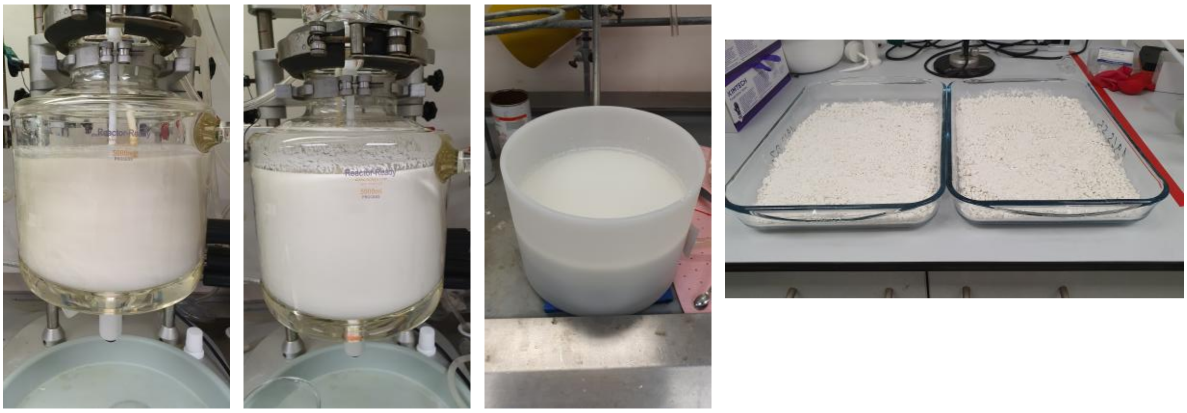

F: Seed bed | **G**: End of crystallisation | **H**: Filtration | **J**: Dry **2**

Figure S2. Large scale synthesis of **1a**, Oxidation step, photographic monitoring.

Step 2:

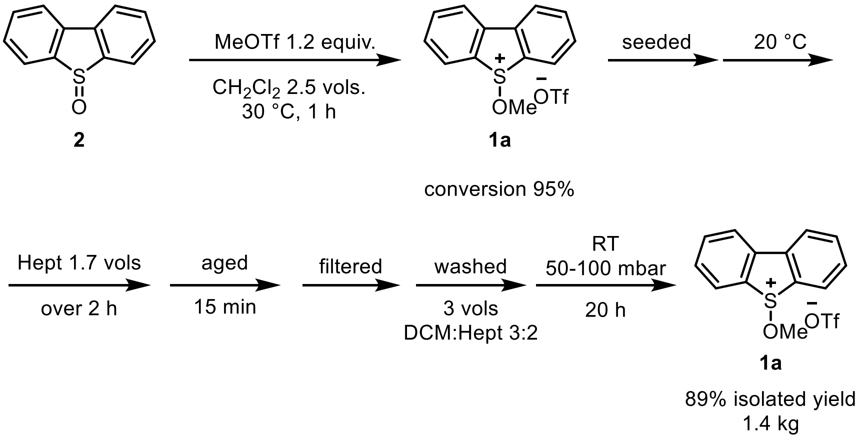

Scheme S3. Synthesis of **1a**, Alkylation Step.

2 (860 g, 4.3 mol, 1.0 equiv.) was charged in a 5 L Radley reactor followed by CH_2Cl_2 (2.15 L, 2.5 vols, see **Figure S3**, picture K). The temperature of the jacket was regulated to keep the

contents temperature at 30 °C. In the meantime, a peristaltic pump was primed with MeOTf and connected to the reactor by PTFE tubing. The pump was set to dispense MeOTf (845.7 g, 5.2 mol, 1.2 equiv.) over a period of 1 h while the jacket temperature was adjusted to keep the contents temperature between 30-35 °C (**Figure S3**, picture L). Reaction conversion was monitored by ^{1}H-NMR, until conversion >95% was achieved. The reaction mixture was then seeded with 50 mg of **1a** at 30 °C. The seed bed was left aging for 15 min before cooling down the content to 20 °C over a period of 30 min (**Figure S3**, picture M).

Heptane (1.462 L, 1.7 vols) was slowly added over a period of 2 h while keeping the content temperature at 20 °C, then the slurry was left aging for 15 minutes at 20 °C (**Figure S3**, picture N). The reaction was filtered using a large HDPE Buckner funnel and a 30 μm filter cloth inside a bag with a constant nitrogen flow (**Figure S3**, picture P). The wet cake was washed with 3.5 L (4 vols) of a 3:2 CH_2Cl_2:Heptane solution. The wet cake was then transferred into glass trays and placed in a vacuum oven (50-100 mbar) for 20 h at room temperature to give **1a** (1404 g, 90% yield) as a yellow powder (**Figure S3**, picture Q).

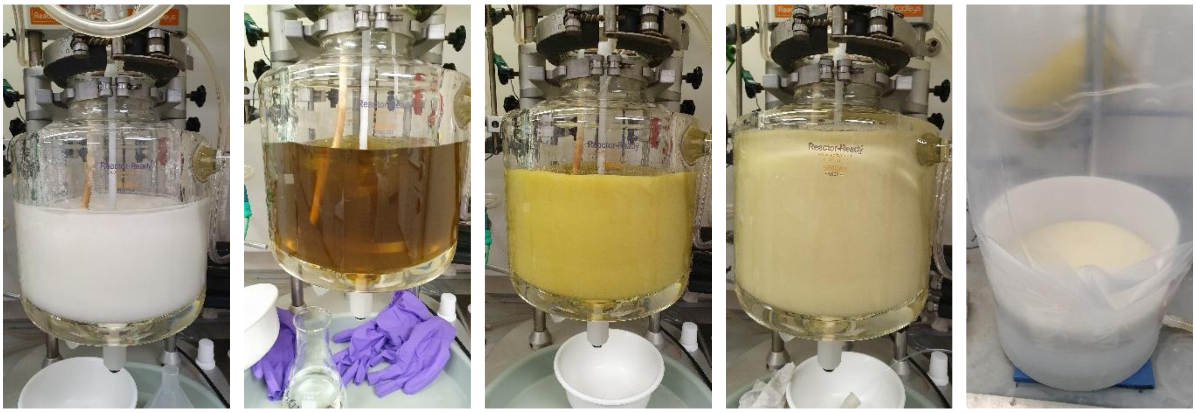

K: **1a** in CH_2Cl_2 | **L**: End of reaction | **M**: Seed bed | **N**: End of crystallisation | **P**: Filtration

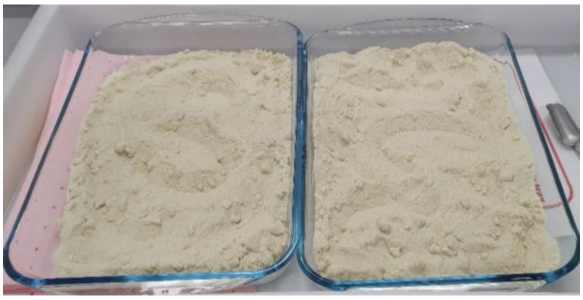

Q: Dry **1a**

Figure S3. Large scale synthesis of **1a**, Alkylation step, photographic monitoring.

6.2 Stability Studies on 1a under the Reaction Conditions

The reaction stream was aged without irradiation to monitor the stability of **1a** over time in pre-reaction conditions.

1a (0.20 g, 0.55 mmol, 1.0 equiv.), tris(2,2'-bipyridine)ruthenium(II) hexafluorophosphate (0.5 mg, 0.0006 mmol, 0.1 mol%) and internal standard dimethyl sulfone (51.7 mg, 0.55 mmol, 1.0 equiv.) were dissolved in 4 mL of acetone, followed by 4-chlorostyrene (132 μL, 1.10 mmol, 2.0 equiv.), 2-chloropyridine (31.2 mg, 0.27 mmol, 0.5 equiv.) and H_2O (19.8 μL, 1.10 mmol, 2.0 equiv.). The mixture was stirred at room temperature for 24 h. 25 μL of solution was collected at different time points and diluted in an NMR tube with 0.6 mL of acetone-d_6. Quantitative ^{1}H-NMR analysis was performed on these samples to determine the degradation of **1a** by following the ratio between **1a** signal (at 8.61 ppm) and the signal of the internal standard dimethyl sulfone (at 3.00 ppm). The results are reported in the table below (**Table S4**).

Table S4: 1a recovery upon aging the reaction stream without irradiation.

**Entry**	**Time** (h)	**1a recovery** (%)
1	0	100
2	2	89
3	8	71
4	24	48

As seen above, **1a** degraded slowly over time in the reaction mixture when aged in solution with all the required reagents. Given the long reaction time required for a kg scale flow experiment, having a single reacting solution is not a viable option.

To assess whether the stability of **1a** can be improved if separated from the other reagents, the following stability test was carried out: **1a** (0.20 g, 0.55 mmol, 1.0 equiv.), and dimethyl sulfone (51.7 mg, 0.55 mmol, 1.0 equiv.) were dissolved into 2 mL of acetone. The mixture was stirred at room temperature for 24 h. 25 μL of solution was collected at different time points and diluted in an NMR tube with 0.6 mL of acetone-d_6. Quantitative ^{1}H-NMR analysis was performed on these samples to determine the degradation of **1a** by following the ratio between MeODBT signal (at 8.61 ppm) and the signal of the internal standard dimethyl sulfone

(at 3.00 ppm). The results are reported in the table below (**Table S5**). As seen, there is a greater degree of recovery of **1a** when aged in solution separated from all the other reagents.

Table S5: 1a recovery upon aging in acetone without irradiation.

**Entry**	**Time** (h)	**1a recovery** (%)
1	0	100
2	1	99
3	3	97
4	6	96
5	23	88

As can be seen from these stability studies, although the starting material mixture was stable for lab-scale synthesis within a few hours of preparation, it was not stable for extended periods of time under the conditions for bulk material preparation. Therefore, in the continuous flow setup, a single feed setup was used for conditions exploration and a two-feed setup was applied on the kilo-scale process.

6.3 General Procedures for Photoflow Reactions

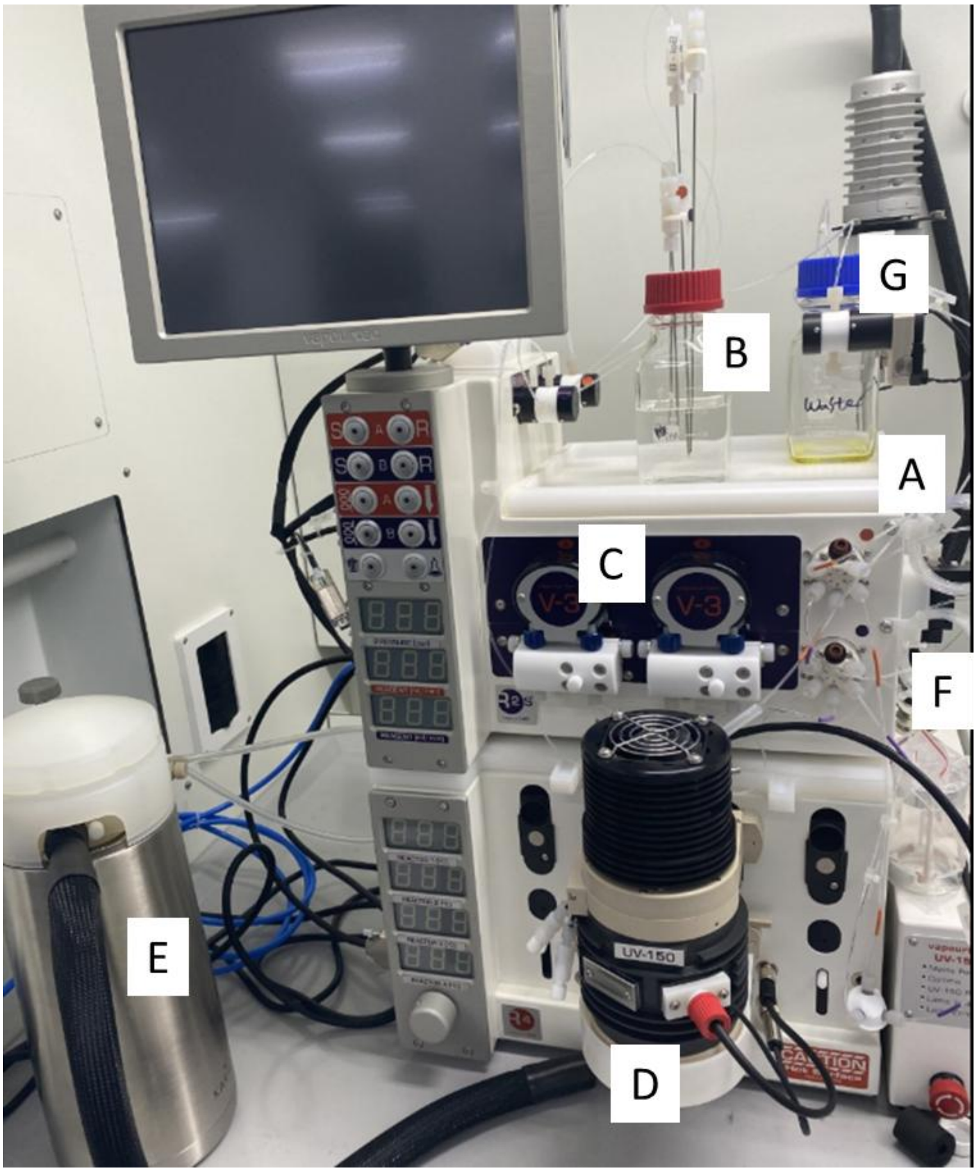

Figure S4. Photo of the flow system (Vapourtec® R2/R4 series connected to a UV-150 photoreactor). **A**: 2 mL loading coil; **B**: Inlet for reagent and solvent feed bottles; **C**: peristaltic pump; **D**: UV-150 photoreactor; **E**: cooling tank; **F**: backpressure regulator; **G**: collection port.

General Procedure A for lab-scale continuous flow setup (single feed)

1a (0.20 g, 0.55 mmol, 1.0 equiv.) and tris(2,2'-bipyridine)ruthenium(II) hexafluorophosphate (0.5 mg, 0.0006 mmol, 0.1 mol%) was charged to a vial and inerted with nitrogen vacuum cycle, three times. 4 mL acetone (20 vol.), previously degassed by 15 minutes nitrogen sparging, was charged followed by 4-chlorostyrene (152.2 mg, 1.098 mmol, 2.0 equiv.), 2-chloropyridine (31.2 mg, 0.274 mmol, 0.5 equiv.) and H_2O (19.8 mg, 1.098 mmol, 2.0 equiv.). The mixture was stirred to full dissolution. Vapourtec R2/R4 was configured with a UV-150

photoreactor and a 450 nm 16 W LED lamp. The system was primed with acetone and cooled to 5 °C. The resulting yellow solution was injected at the desired flow rate and all fractions were collected. To the combined fractions was added 1,3,5-trimethoxybenzene as internal standard for qNMR analysis.

General Procedure B for lab-scale continuous flow setup (two feeds)

1a (0.20 g 0.55 mmol, 1.0 equiv.) was charged to a vial and inerted with nitrogen vacuum cycle, three times before dissolved in 2 mL acetone (10 vol.). tris(2,2'-bipyridine)ruthenium(II) hexafluorophosphate (0.5 mg, 0.0006 mmol, 0.1 mol%) was charged to a vial and inerted with nitrogen vacuum cycle, three times. 2 mL acetone (20 vol.) was charged followed by 4-chlorostyrene (152.2 mg, 1.098 mmol, 2.0 equiv.), 2-chloropyridine (31.2 mg, 0.274 mmol, 0.5 equiv.) and H_2O (19.8 mg, 1.098 mmol, 2.0 equiv.). Both vials were stirred to full dissolution and degassed with nitrogen sparging for at least 15 min. Vapourtec R2/R4 was configured with a UV-150 photoreactor and a 450 nm 16 W LED lamp. The system was primed with acetone and cooled to the target temperature. The front of the two feeds were marked by an air bubble and the flow rates were adjusted so that the two feeds reached the mixing tee-piece at the same time. The resulting solutions were then injected at the desired flow rate respectively. All fractions were collected. To the combined fractions was added 1,3,5-trimethoxybenzene as internal standard for qNMR analysis.

6.4 Optimization of Batch Conditions to Continuous Flow Setup

Several continuous flow conditions were examined following the optimized batch conditions. Lower temperatures and inert atmosphere both contributed to higher yield.

Table S6: Temperature optimisation for photoflow.

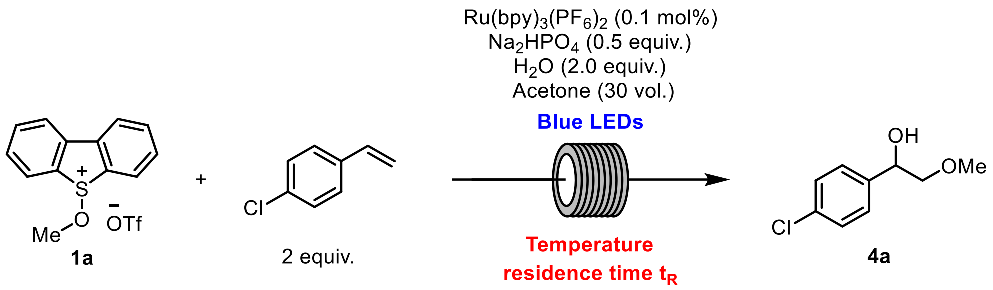

Entry	Temp (°C)	Flow rate (mL/min)	Residence time (min)	qNMR yield (%)
1	-4 to -15	0.167	60	42
2	-20 to -37	0.667	15	71
3	-30	2.00	5	70
4	-20	2.00	5	64
5	+5	5.00	2	59

Both temperature and flow rate had an impact on yield. The temperature proved difficult to stabilize at slow flow rates (**Table S6**, entries 1-2). Faster flow rates (entries 2-5) allowed for better control while maintaining good yields. The best results were obtained at -30 °C (entry 3), though the reaction still gave good yields at higher temperatures (entries 4-5).

Table S7: Base optimisation for photoflow.

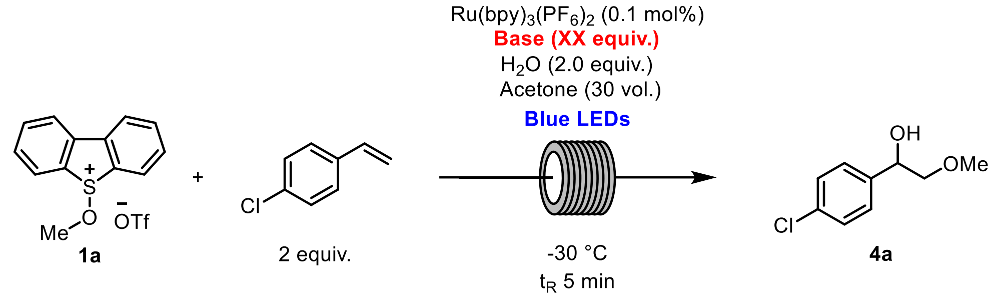

Entry	Base	Equiv.	qNMR Yield (%)
1	Na_2HPO_4	0.5	80
2	NaH_2PO_4 $2H_2O$	0.25	79
3	$Bu_4NH_2PO_4$	0.5	49
4	2-Cl-Py	0.5	81
5	DBU	0.5	52
6	DABCO	0.5	19

While Na_2HPO_4 gave high yields (**Table S7**, entries 1-2), concerns about the reaction solution's homogeneity at low temperatures led to a brief screening of more solvent soluble bases (entries 3-6), with 2-chloropyridine giving similar results to those previously obtained with Na_2HPO_4.

Table S8. Concentration and set up optimisation for photoflow.

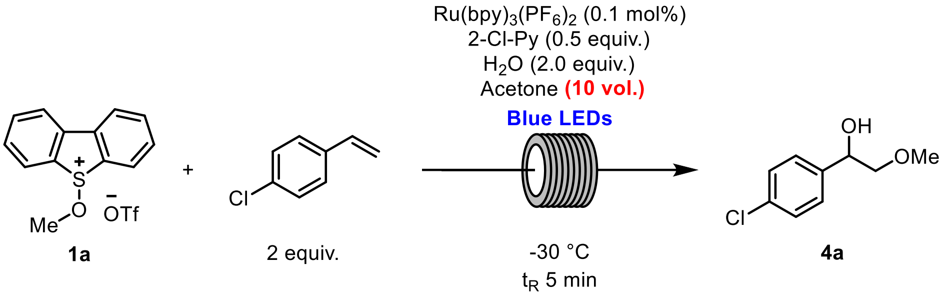

Entry	Concentration (g/mL)	Feed	1a flow rate (mL/min)	3a flow rate(mL/min)	qNMR yield (%)
1	0.1	1	-	-	81-85
2	0.2	1	-	-	68
3[a]	0.2	1	-	-	60
4	0.1	2	1.0	1.0	82
5	0.1	2	1.2	1.0	81
6	0.1	2	1.0	1.2	76

[a] **1a** aged in acetone 16 h before running the reaction.

Doubling the concentration (**Table S8**, entries 2-3) led to lower yields. Due to the instability of **1a** in the reaction conditions over time pre-irradiation, the reaction solution was split in to two streams, one containing **1a**, the other containing the rest of the reaction components (entries 4-6). This allowed high yields (entry 4) without risking the degradation of the **1a** in the reaction conditions before irradiation. Modifying the flow speed of either stream (entries 5-6) by 20% showed significant flexibility in the flow rate without compromising the yield.

Table S9. Final settings optimization for photoflow.

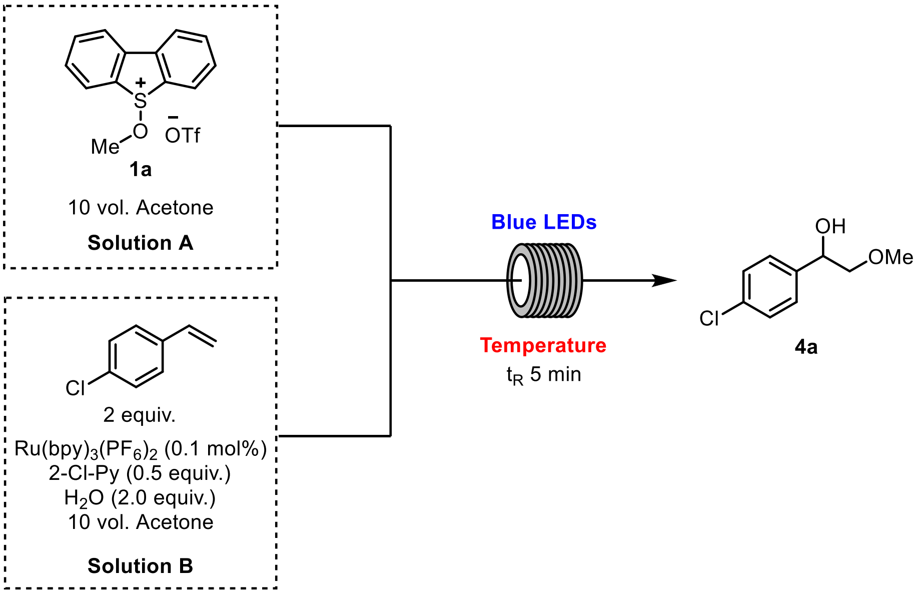

Entry	Temp (°C)	Flow rate (mL/min)	Residence time (min)	qNMR yield (%)
1	-30	2.00	5	82
2	+5	2.00	5	69
3[a]	+5	2.00	5	59
4[a,b]	+5	2.00	5	35

[a]Non-degassed reaction; [b]Concentration increased to 0.2 g/mL.

Due to the limited temperature range of the kg-scale photoflow reactor, the optimized reaction conditions were tried out at 5 °C (**Table S9**, entry 2). While a small drop in yield was observed, the yield was deemed high enough to pursue the large-scale reaction, with the aim of keeping the temperature between 0 °C and 5 °C. Degassing the solvent proved essential to avoid reaction deactivation (entries 3-4), and increasing the concentration once again led to lower yields (entry 4).

6.5 Analysis Methods and Process Safety Assessment

qNMR analysis

The alkylation step to prepare **1a** and the photoflow reaction were analyzed using quantitative ^{1}H NMR.

To the combined fractions was added a measured amount of 1,3,5-trimethoxybenzene (m_{int}) as internal standard and the standard fully dissolved. A sample of the mixture was then dissolved in $CDCl_3$ and processed for ^{1}H NMR. NMR spectra were recorded using a Bruker 400 Ultrashield Spectrometer at 25 °C. Chemical shifts are reported in parts per million (ppm) and referenced against the residual solvent signal of $CDCl_3$ (7.26 ppm for ^{1}H NMR). qNMR parameters were set to 8 scans and 40 s d_1 delay. The featured peaks of both internal standard (I_{int}, typically 5.8 ppm) and sample (I_{sample}, typically 4.6 ppm) and their corresponding numbers of nuclei (N_{int} and N_{sample}) were measured and the product qNMR assay (m_{sample}) was calculated using the equation below. Finally, the qNMR assay yield was calculated against the theoretical output of product:

$$m_{sample} = \frac{I_{sample}}{I_{int}} \times \frac{N_{int}}{N_{sample}} \times \frac{M_{sample}}{M_{int}} \times m_{int} \times P_{int}$$

$$Assay\ Yield = \frac{m_{sample}}{m_{theory}} \times 100\%$$

The following example was performed on a 200 mg MeODBT reaction:

Target product peak integration $I_{sample} = 2.26$

Internal standard peak integration $I_{int} = 3.00$

Internal standard peak nuclei $N_{int} = 3$

Target product peak nuclei $N_{sample} = 1$

Product molecular weight $M_{sample} = 186.36$ g/mol

Internal standard molecular weight $M_{int} = 168.19$ g/mol

Internal standard weight $m_{int} = 34.7$ mg

Internal standard purity $P_{int} = 99.9\%$

Theoretical output $m_{theory} = 102$ mg

$$m_{sample} = \frac{2.26}{3.00} \times \frac{3}{1} \times \frac{186.36}{168.19} \times 34.7 \times 99.9\% = 86.9 \text{ mg}$$

$$Assay\ Yield = \frac{86.9}{102} \times 100\% = 85\%$$

HPLC analysis

Table S10. The oxidation step to prepare **2** was monitored by HPLC, method A.

Method Parameter	Condition		
**Column**	Ascentis Express C18 4.6 x 100 mm, 2.7 µm		
**Flowrate**	1.8 mL/min		
**Wavelength detection**	254 nm		
**Column Temperature**	40 °C		
**Injection volume**	2 µL		
**Sample temperature**	room temperature		
**Mobile Phase A**	0.1% (v/v) H_3PO_4 in water		
**Mobile Phase B**	Acetonitrile		
**Diluent**	Acetonitrile		
**Gradient**	**Time (min)**	**A%**	**B%**
	0.0	90	10
	6.0	5	95
	8.0	5	95
	8.01	90	10
	10.0	90	10

Compound	***RT / min***
DBT	5.80
**2**	3.00

Process Safety Assessment

Thermal stability information was obtained for all starting materials as well as both the starting mixture and end-of-reaction mixture. Differential scanning calorimetry (DSC) showed a large (301.5 J/g) exotherm in the starting material mixture prior to irradiation (Figure 5). But isothermal age at 50 °C for 16 h showed similar thermal activity, indicating good thermal stability of the reaction mixture. DSC of individual starting materials and end-of-reaction

mixture found no obvious thermal hazard. ARC (accelerated rate calorimetry) also showed no obvious thermal hazard.

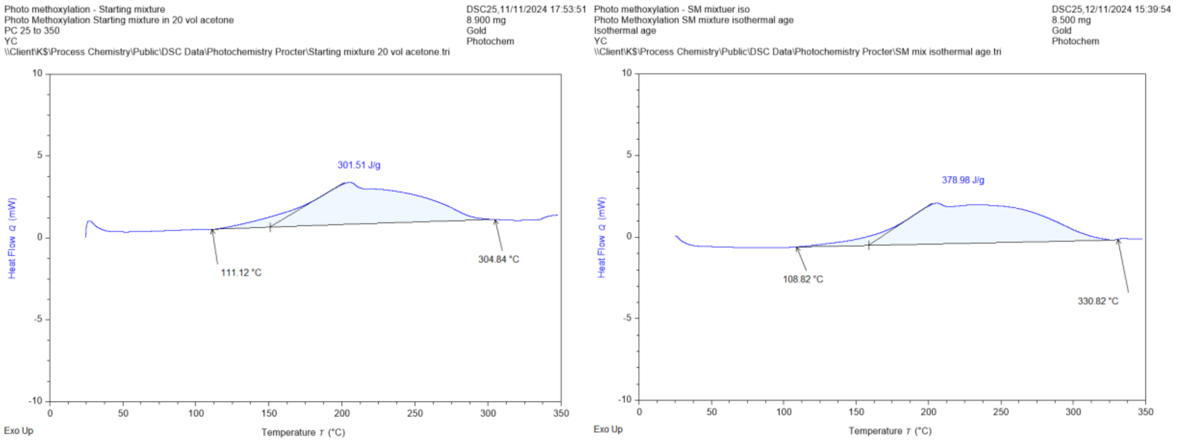

Figure S5. DSC traces for the starting mixture with and without isothermal age.

All reagent charging and handling, as well as the photoreactor itself, were handled in a fume hood. Chemists were present at all times to constantly monitor the system pressure and temperature during the operation and slow/stop the addition, and/or switch LED, if the reactor started to pressurise or leak. Drums feeding into both pumps, the mixing vessel itself, and the collection drums were secondary contained.

6.6 Large Scale Photoflow Development

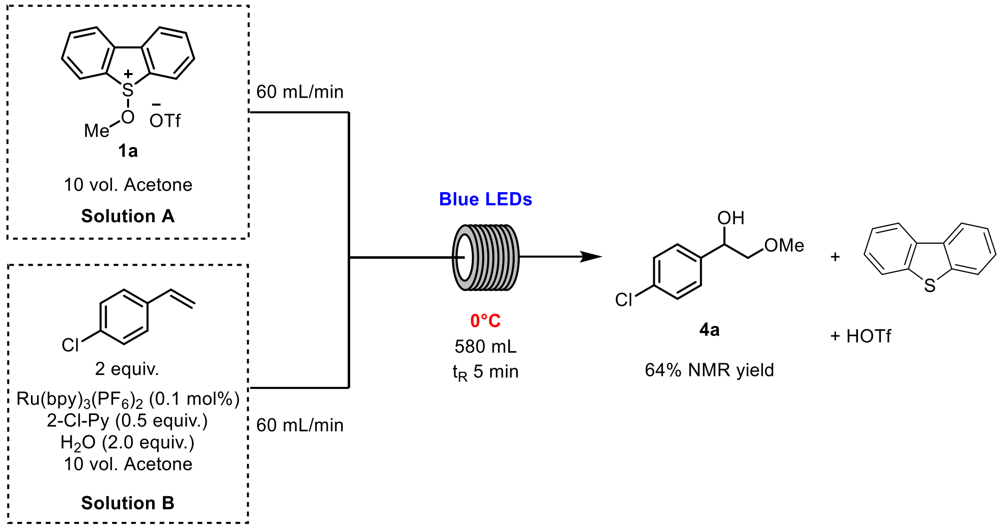

Plug flow photoreactor (PFP) design

The PFP is comprised of FEP tubing (Polyflon technology Ltd, 6.35 mm OD, 4.76 mm ID, total irradiated volume ~ 580 mL) coiled around an aluminium central frame holder and submerged into a IPA/water bath. The bath is connected to a heat exchanger and recirculated at -15 °C during the reaction. Both sides of the bath are flanked with two LED panels, each consisting of 18 units of 100W COB-LEDs (HP COB LED by Ivy Bridge Technology Co. Ltd, 450–455 nm) with a total input power of 3.6 kW. The LED panels are continuously cooled by water flowing through the copper pipe embedded into the LED panels. The temperature at the inlet and outlet of the photoreactor, as well as the water bath are monitored by digital thermometers equipped with Type K thermocouple (Comark C-48 type). The flow rates of reaction mixtures are controlled by diaphragm metering pumps (EXTRONIC ExBb by ProMinent). The inlet of combined stream is controlled by a Masterflex gear pump (Ismatec® Reglo digital gear pump drive with B-mount pump head). The inlet flow rate is also monitored by a mass flow meter (Model M14 by Bronkhorst UK) positioned before entry into the irradiation volume of the reactor. The product mixture is collected into a Polastic coated Duran Schott bottle. The PFP set up in a standard fume hood is shown in **Figure S6** and the emission spectrum of the LED chips is shown in **Figure S7**.

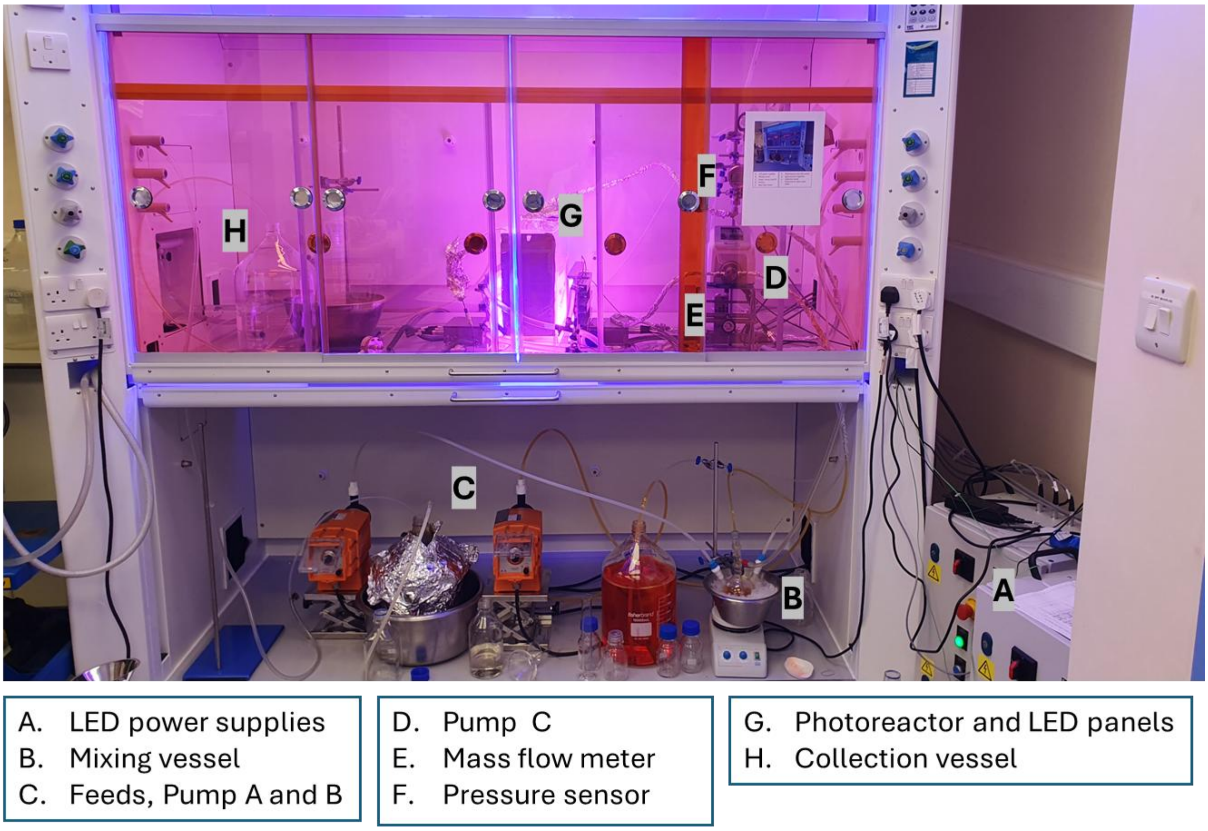

Figure S6. FPF setup in a 200 x 220 x 70 cm fume hood.

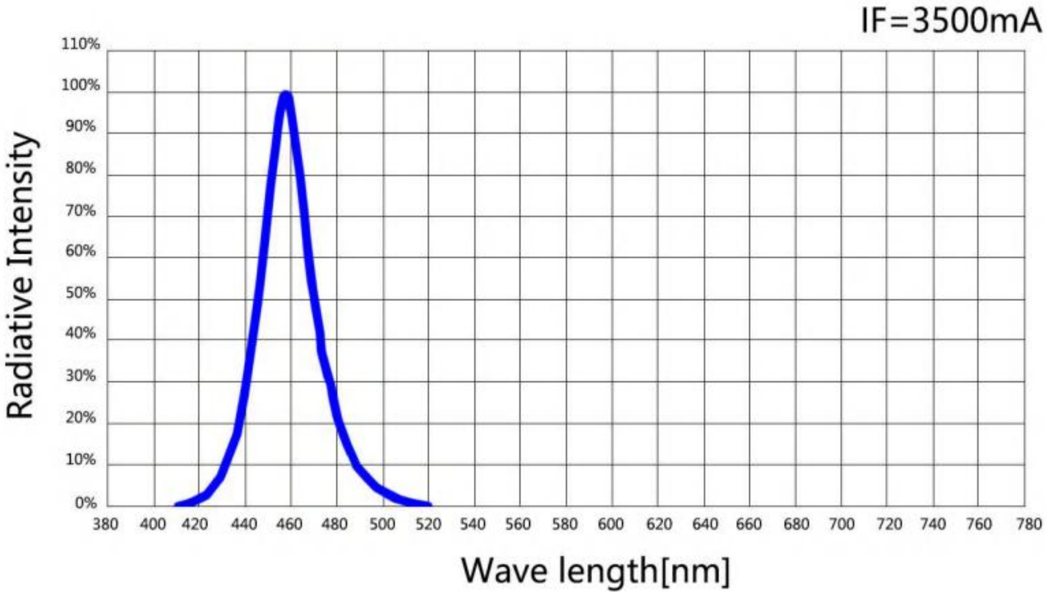

Figure S7. Emission spectrum of LED chips provided by Ivy Bridge Technology Co. Ltd.

Procedure for the 1.0 kg scale operation.

1a was synthesized according to procedure previously described. All other reagents and solvents were used as received from the respective suppliers: 4-chlorostyrene, 2-chloropyridine and tris(2,2'-bipyridine)ruthenium(II) hexafluorophosphate were purchased from Sigma Aldrich, and acetone was purchased from VWR.

A 10 L plastic coated Duran Schott bottle was charged with **1a** (1.0 kg, 2.74 mol, 1.0 equiv.) and acetone (7.8 kg, 10 L). A separate 10 L plastic coated Duran Schott bottle was charged with 4-chlorostyrene (0.76 kg, 5.49 mol, 2.0 equiv.), 2-chloropyridine (0.13 kg, 1.37 mol, 0.5 equiv.), tris(2,2'-bipyridine)ruthenium(II) hexafluorophosphate (2.0 g, 0.003 mol, 0.1 mol%), water (0.1 kg, 5.49 mol, 2.0 equiv.) and acetone (7.8 kg, 10 L). The contents in both drums were stirred for full dissolution at 22 °C and kept under inert environment through constant nitrogen sparging. Both feeds were pumped into a 500 mL mixing flask at 60 mL/min flow rate using two diaphragm pumps. The combined solution in the mixing flask was pre-cooled in ice batch and continuously stirred using a magnetic stirrer and allowed to reach ~450 mL mark prior to being pumped into the PFP at 94 g/min (120 mL/min) flow rate. The photoreactor was pre-cooled to 0 °C prior to reaction and kept constant throughout. The product mixture was collected using a plastic-lined Duran Schott bottle. The solution level in the mixing vessel was monitored throughout the operation. Additional acetone was added to the mixing vessel near the end of the run to chase the remaining reaction mixture in the system.

All product-containing stream was combined and well mixed. A sample was removed from bulk for qNMR analysis (**Figure S8**). The assay yield of kilo-scale operation was quantified and summarized in **Table S11**. Overall, 64% assay yield was achieved for the 1.0 kg-scale process over 3.5 h.

Table S11. Assay yield calculation of 1.0 kg scale run.

**1.0 kg scale run – qNMR assay**	
End-of-reactoin solution size (kg)	16.668
Sample size (g)	9.998
Target product peak integration I_{sample}	1.16
Internal standard peak integration I_{int}	3.00
Internal standard peak nuclei N_{int}	3

Target product peak nuclei N_{sample}	1
Product molecular weight M_{sample} (g/mol)	186.36
Internal standard molecular weight M_{int} (g/mol)	168.19
Internal standard TMB weight m_{int} (mg)	152.8
Internal standard purity P_{int} (%)	99.9
Theoretical output m_{theory} (g)	512.2
Product weight in sample m_{sample} (mg)	196.2
Product weight total m_{total} (g)	327.1
**Assay yield (%)**	**64**

The calculation for 1.0 kg scale operation:

Product weight in sample:

$$m_{sample} = \frac{I_{sample}}{I_{int}} \times \frac{N_{int}}{N_{sample}} \times \frac{M_{sample}}{M_{int}} \times m_{int} \times P_{int}$$

$$= \frac{1.16}{3.00} \times \frac{3}{1} \times \frac{186.36}{168.19} \times 152.8 \times 99.9\% = 196.2 \text{ mg}$$

Product weight in end-of-reaction:

$$m_{total} = m_{sample} \times \frac{EoR\ solution}{sample\ size} = \frac{196.2}{10^3} \times \frac{16.668 \times 10^3}{9.998} = 327.1 \text{ g}$$

Assay yield of 1.0 kg scale operation:

$$Assay\ Yield = \frac{m_{total}}{m_{theory}} \times 100\% = \frac{327.1}{512.2} \times 100\% = 64\%$$

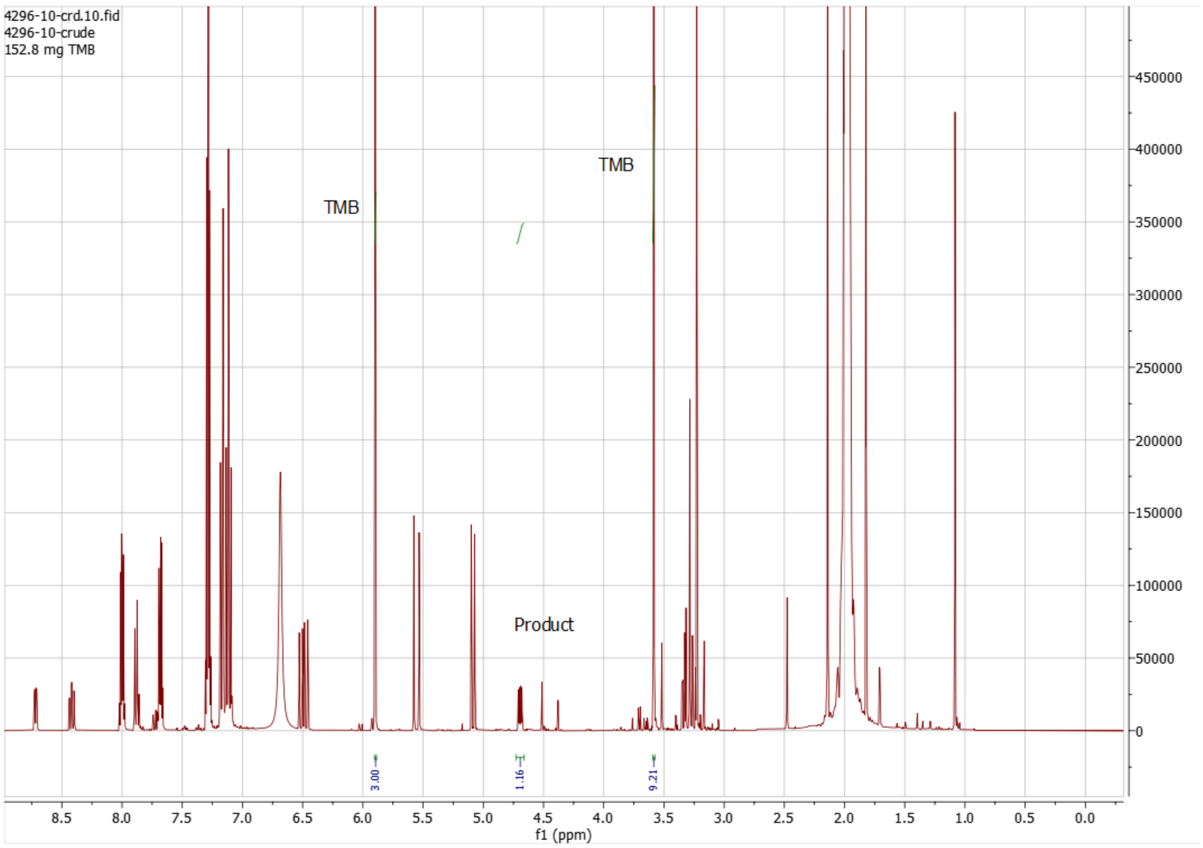

Figure S8. qNMR spectrum of the product mixture from the 1.0 kg-scale process.

7 Mechanistic Considerations

7.1 Detection of Carbon Radical Intermediate Experiment

Detection of TEMPO adduct

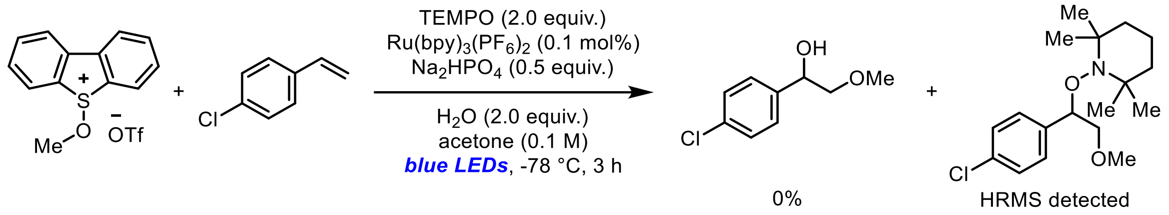

Following **GP5** but with the addition of TEMPO (31 mg, 0.2 mmol, 2.0 equiv.). After reaction, a small aliquot of the product mixture was taken directly and analyzed by ESI HR-MS. The TEMPO adduct could be detected: HRMS (APCI): Found $(M+H)^+$ 326.1881, $C_{18}H_{29}O_2NCl$ requires 326.1881.

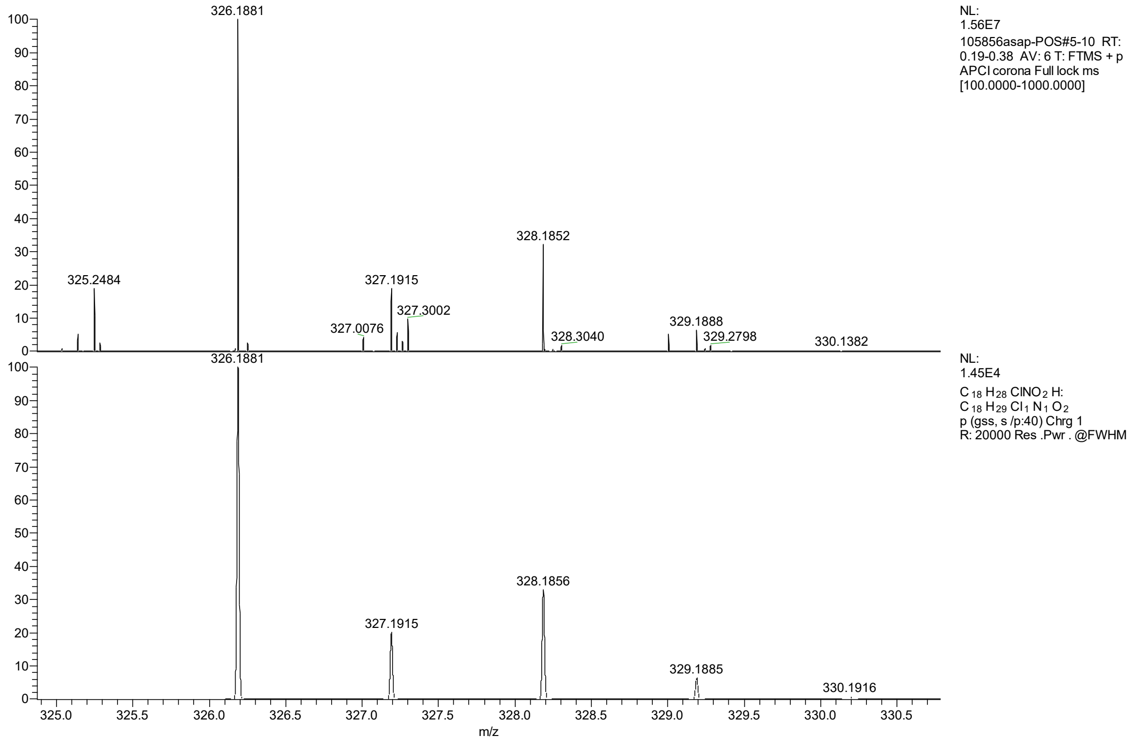

Figure S9. HRMS detection of the TEMPO adduct.

Detection of sulfonium salt intermediate

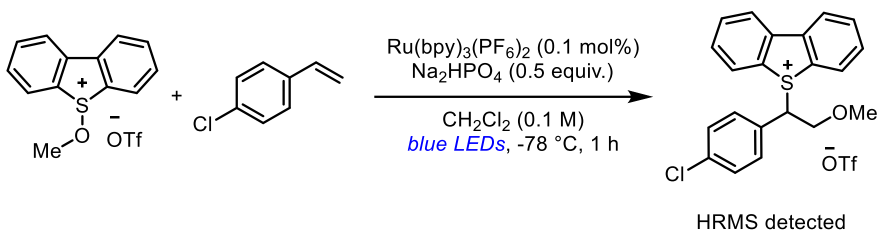

Following **GP6** but without the presence of a nucleophile. After 1 h reaction, a small aliquot of the product mixture was taken directly and analyzed by ESI HR-MS. The proposed intermediate could be detected: HRMS (ESI): Found $(M{-}OTf)^{+}$ 353.0761, $C_{21}H_{18}OClS$ requires 353.0756.

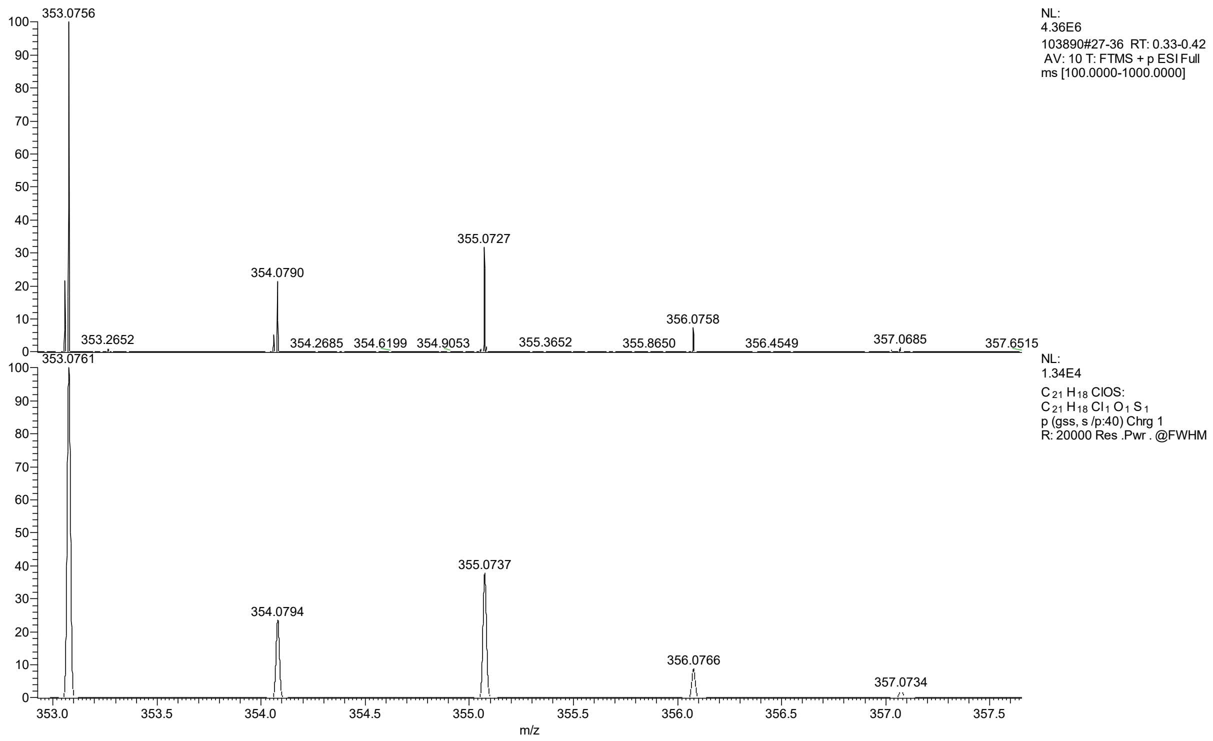

Figure S10. HRMS detection of sulfonium salt intermediate.

7.2 O^{18} Isotope Labeling Studies

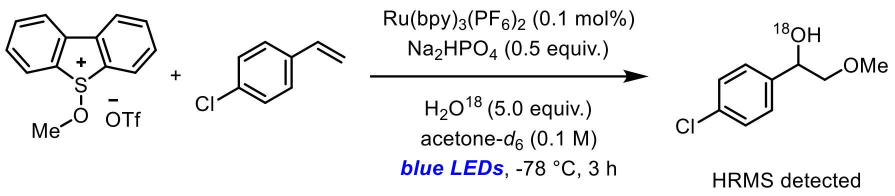

Following **GP6** but using H_2O^{18} (9 µL, 5.0 equiv.) in acetone-d_6. After reaction, a small aliquot of the product mixture was taken directly and analyzed by ESI HR-MS. The O^{18} isotope labled product could be detected:

HRMS (APCI): Found (M-H$^+$) 187.0413, $C_9H_{10}OO^{18}Cl$ requires 187.0417.

HRMS (APCI): Found (M-H$^+$) 185.0371, $C_9H_{10}O_2Cl$ requires 185.0375.

Relative amount of O^{18} compound = 3.81% when compared to the O^{16} compound.

Table S12. Relative amount of O^{18} compound and the O^{16} compound.

T: FTMS - p APCI corona Full lock ms [5(
m/z= 175.8636-195.5920

m/z	Intensity	Relative
179.0709	1323705.8	1.77
180.0788	1069990.9	1.43
181.0502	4452641.0	5.96
182.0581	1085699.5	1.45
182.9851	5106700.0	6.84
183.0215	12372492.0	16.57
183.0659	1957875.9	2.62
184.9822	1538714.5	2.06
185.0008	6693506.5	8.96
185.0186	3574078.3	4.79
185.0371	74681448.0	100.00
186.0405	6714761.0	8.99
186.9978	1815846.1	2.43
187.0220	2935478.8	3.93
187.0342	23491086.0	31.46
187.0413	2844371.5	3.81
188.0375	1955166.6	2.62
189.0384	992157.3	1.33
191.0168	1266482.6	1.70

7.3 Evidence for the Fragmentation of Alkoxy Radicals

Alkoxy Radicals are known to undergo *β*-scission processes a C−C bond at the *β*-position is homolytically cleaved to form an alkyl radical and a carbonyl compound.[21]

Following **GP5**, but with lepidine **8** (26 µL, 0.20 mmol) added as a radical acceptor, 5-((3-methylbutan-2-yl)oxy)-5*H*-dibenzo[*b*,*d*]thiophen-5-ium triflate **1aj** (42 mg, 0.10 mmol) gave 2-isopropyl-4-methylquinoline **9** (5% NMR yield) along with acetaldehyde **10a** (24% NMR yield), and isobutyraldehyde **10b** (4% NMR yield). The NMR yields were determined by adding mesitylene (7 µL, 0.05 mmol, 0.5 equiv.) as an NMR internal standard.

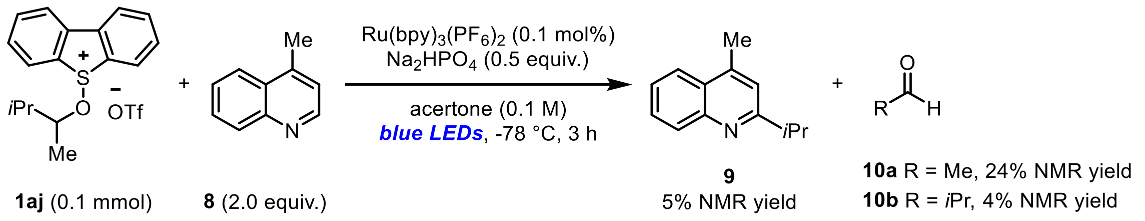

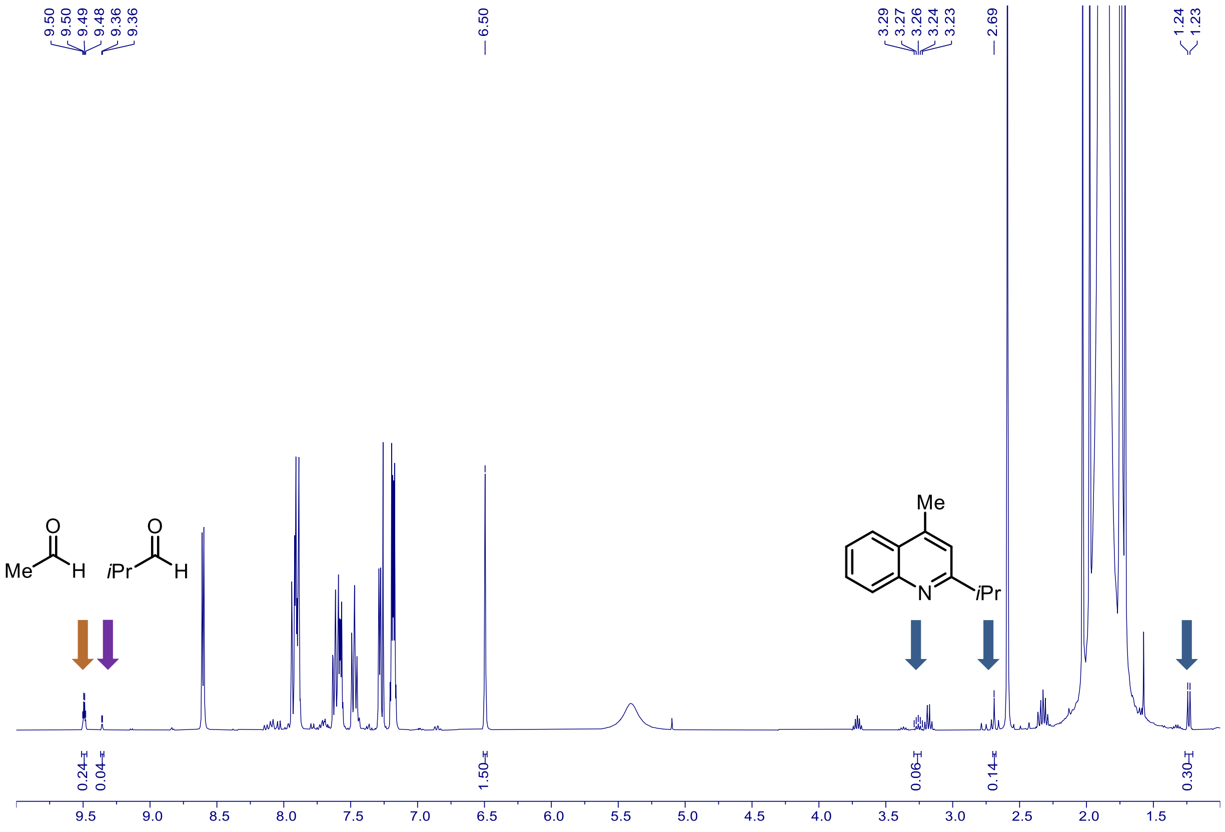

Figure S11. ^{1}H NMR spectrum of the product mixture.

Following **GP5**, but with lepidine **8** (26 μL, 0.20 mmol) added as a radical acceptor and using HFIP as solvent, 5-((3-methylbutan-2-yl)oxy)-5*H*-dibenzo[*b*,*d*]thiophen-5-ium triflate **1aj** (42 mg, 0.10 mmol) gave 2-isopropyl-4-methylquinoline **9** (36% NMR yield) along with acetaldehyde **10a** (72% NMR yield). The NMR yields were determined by adding mesitylene (7 μL, 0.05 mmol, 0.5 equiv.) as an NMR internal standard.

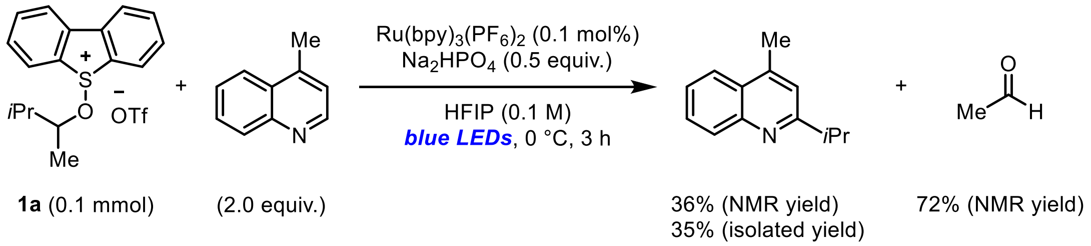

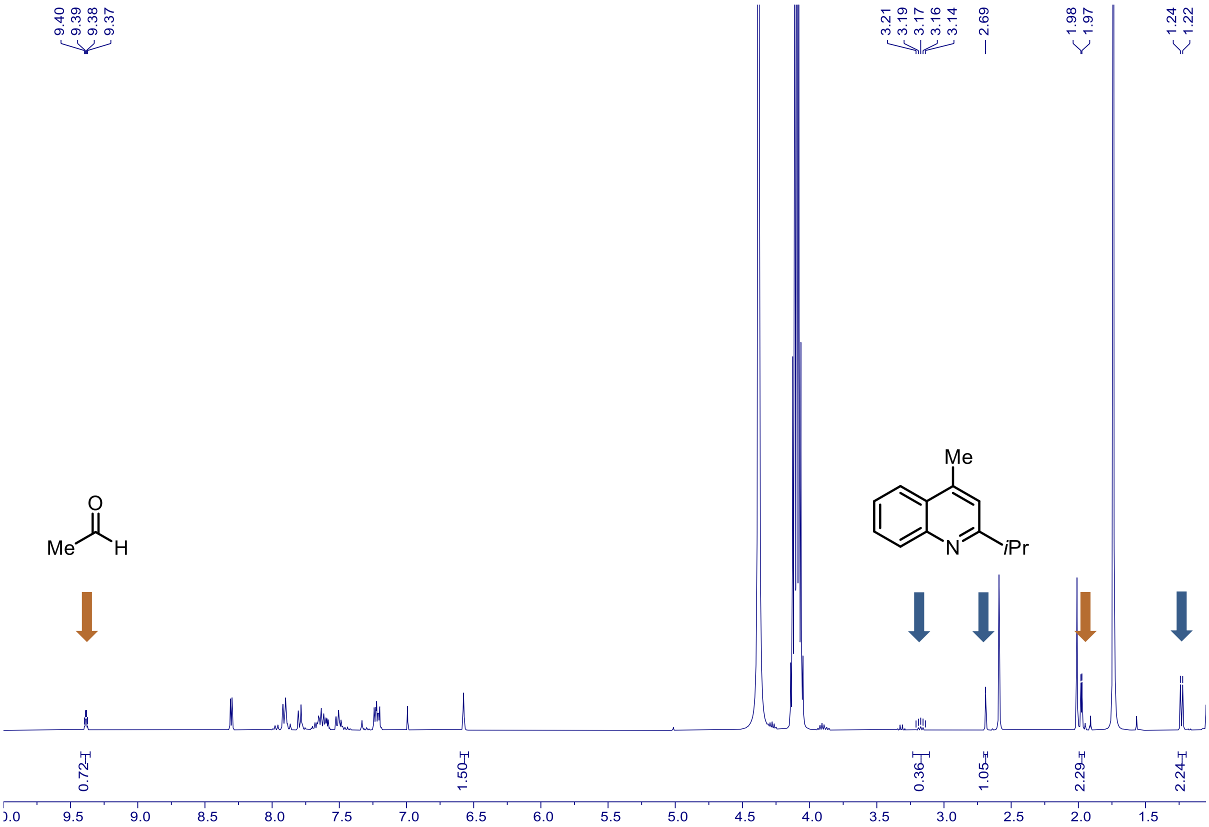

Figure S12. ^{1}H NMR spectrum of the product mixture.

Data for 2-isopropyl-4-methylquinoline **9** (6.5 mg, 35%, isolated): ^{1}H NMR (400 MHz, $CDCl_3$) δ 8.06 (1H, d, *J* = 8.4 Hz), 7.95 (1H, dd, *J* = 8.4, 1.4 Hz), 7.67 (1H, ddd, *J* = 8.4, 6.8, 1.4 Hz), 7.50 (1H, ddd, *J* = 8.2, 6.9, 1.3 Hz), 7.18 (1H, s), 3.23 (1H, hept, *J* = 6.9 Hz), 2.69 (3H, s), 1.39

(6H, d, J = 6.9 Hz); ^{13}C NMR (101 MHz, $CDCl_3$) δ 167.5, 129.6, 129.1, 125.6, 123.7, 119.9, 37.3, 22.7, 19.0. Data in accordance with the literature.[22]

Data for acetaldehyde **10a**: ^{1}H NMR (400 MHz, $CDCl_3$) δ 9.39 (1H, q, J = 3.0 Hz), 1.98 (3H, d, J = 3.0 Hz).

7.4 Cyclic Voltammetry Studies

Cyclic Voltammetry General Experimental Details

Cyclic voltammetry was conducted on an MultiTrace (PalmSens) potentiostat using a 3-electrode cell configuration. A glassy carbon working electrode was employed alongside a platinum wire counter electrode and a Ag/AgCl reference electrode. All the solutions were degassed by bubbling N_2 prior to measurements. 5 mM solutions of the desired compounds were freshly prepared in dry solvent along with 0.1 M of tetrabutylammonium hexafluorophosphate as supporting electrolyte and were examined at a scan rate of 0.1 V s^{-1}. Ferrocene ($E_{1/2}$ = +0.42 V vs SCE)[23] was added at the end of the measurements as an internal standard to determine the precise potential scale. Potential values are given versus the saturated calomel electrode (SCE). Irreversible reduction waves were obtained in all cases; therefore, the potentials were obtained from the maximum current, $E_p$max.

Table S13. Redox properties.

Redox properties (V vs SCE)				
	$^*E_{red}$	$^*E_{ox}$	E_{red}	E_{ox}
**$Ru(bpy)_3(PF_6)_2$**	+0.77	–0.81	–1.33	+1.29
**1a**	NA	NA	–0.14	NA

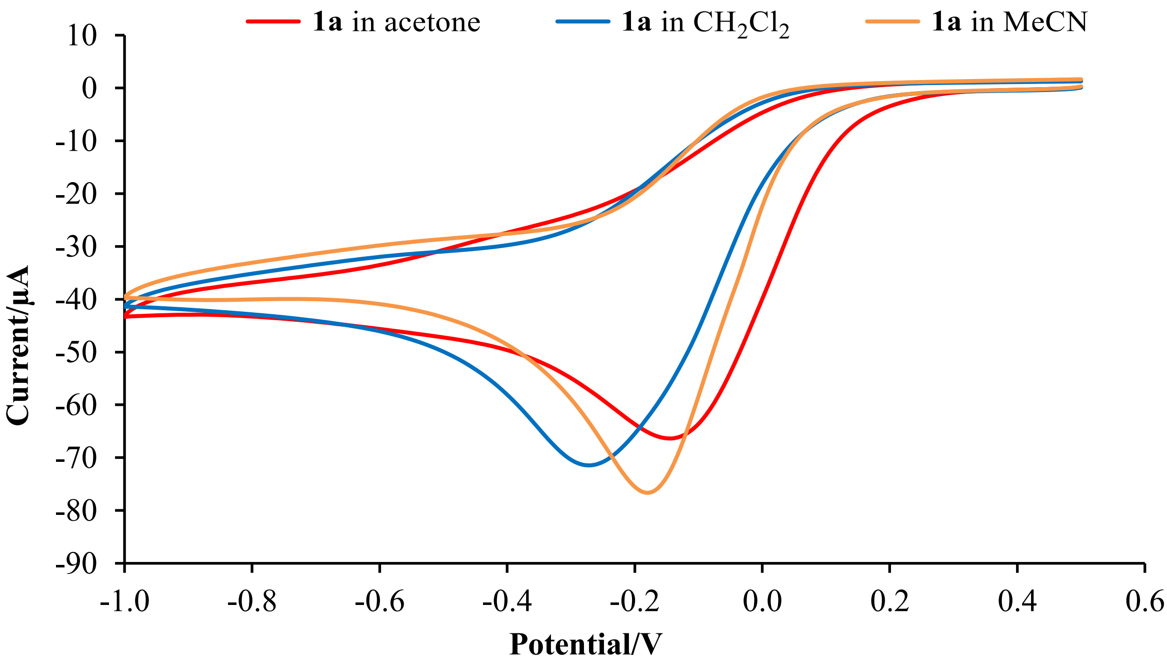

Figure S13. Cyclic voltammetry studies of the reaction component MeODBT·OTf **1a** (5.0 mM) in different solvents.

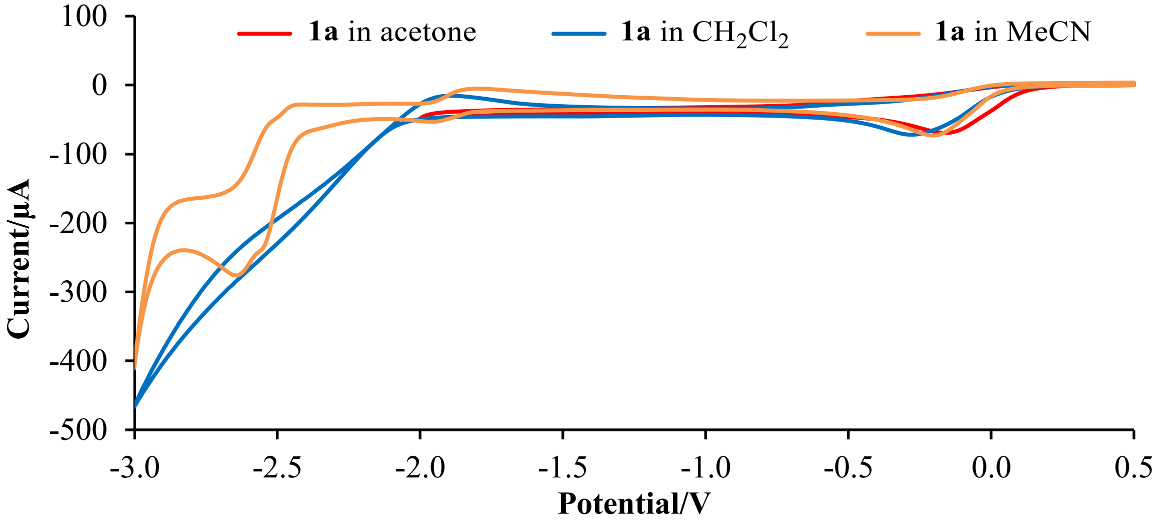

Figure S14. Cyclic voltammetry studies of the reaction component MeODBT·OTf **1a** (5.0 mM) in different solvents.

7.5 Emission Quenching Studies

General Experimental Details

Luminescence spectra were recorded on an Edinburgh Instruments FLS1000 Photoluminescence Spectrometer (with double 325 mm focal length excitation and emission monochromators in Czerny Turner configuration) in 1 cm path length quartz cuvettes. Samples were excited using a 450 W Xe lamp with a long-pass filter on the detection arm and emission captured by a PMT-900 (visible) detector. All data were corrected for excitation lamp and emission detector responses using the in build software in Floracle and plotted in Excel.

Fluorescence Quenching Experiments

Stern-Volmer quenching experiments were carried out monitoring the emission intensity of argon-degassed solutions of $Ru(bpy)_3(PF_6)_2$ (5×10^{-5} M) containing variable amounts of the quencher in dry acetone.

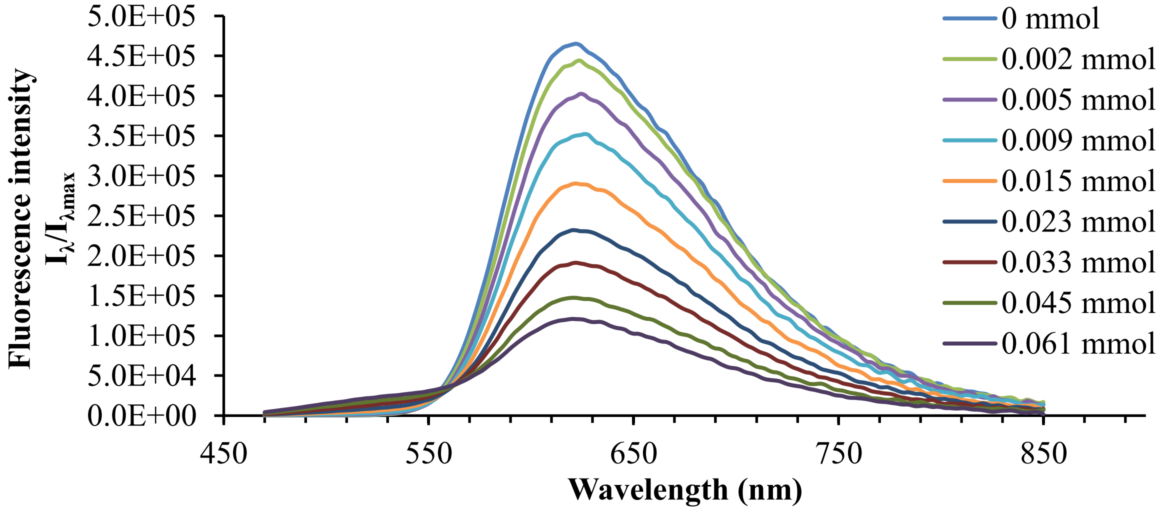

Figure S15. Emission spectra of $Ru(bpy)_3(PF_6)_2$ in dry acetone (5×10^{-5} M) with varying concentrations of MeODBTOTf **1a**.

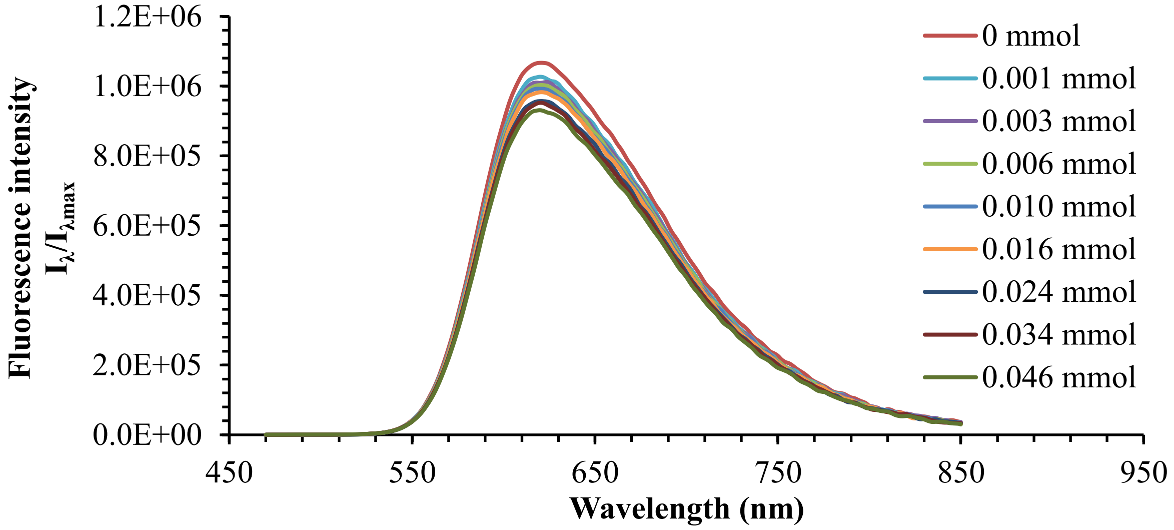

Figure S16. Emission spectra of $Ru(bpy)_3(PF_6)_2$ in dry acetone (5×10^{-5} M) with varying concentrations of 4-Cl-styrene **3a**.

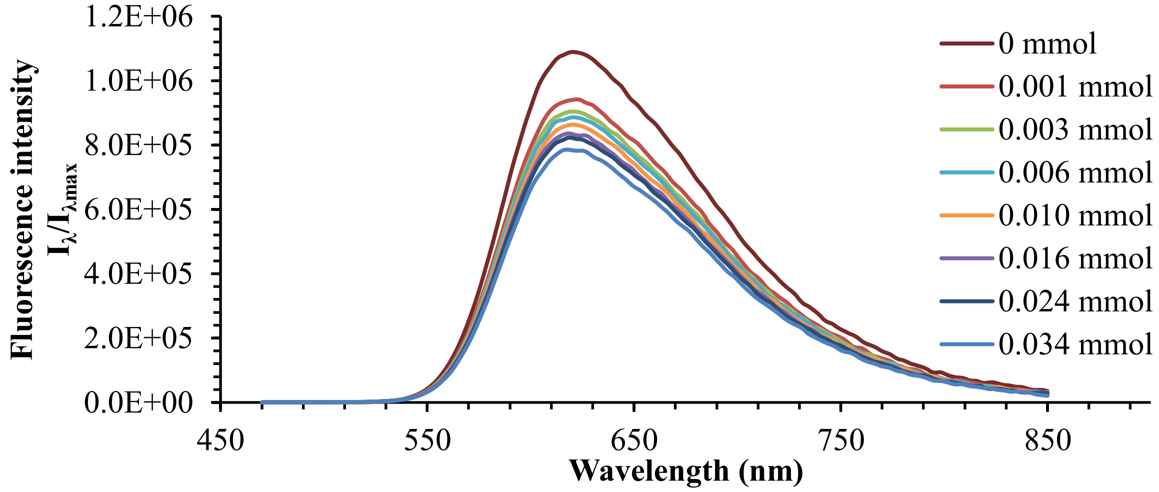

Figure S17. Emission spectra of $Ru(bpy)_3(PF_6)_2$ in dry acetone (5×10^{-5} M) with varying concentrations of H_2O.

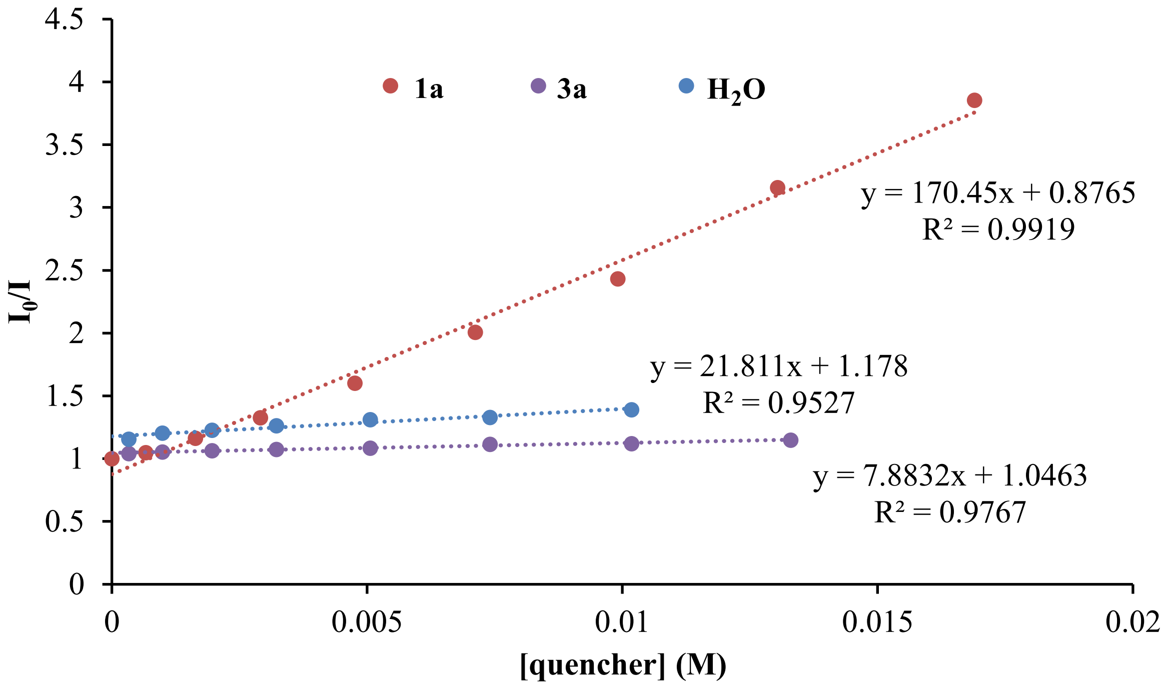

Figure S18. Stern-Volmer plot for of Ru(bpy)$_3$(PF$_6$)$_2$ in dry acetone (5×10^{-5} M) with varying concentrations of MeODBTOTf **1a**, 4-Cl-styrene **3a** and H_2O.

7.6 Quantum Yield (Φ) Determination

General Experimental Details

Samples were irradiated using Kessil blue LEDs (λ_{max} = 456 nm) set to 25% intensity, with the reaction tube placed exactly 2 cm from the bulb. The quantum yield was calculated following procedures previously reported.[24] The ferrioxalate actinometer solution decomposes from ferric to ferrous ions upon irradiation, the ferrous ions are then complexed with 1,10-phenanthroline and the UV/Vis absorbance of the complex is monitored at 510 nm. The moles of complex formed are related to the moles of photons absorbed.

Preparation of Solutions

Ferrioxalate solution (A)

In a dark room, potassium ferrioxalate trihydrate (1.84 g, 3.75 mmol, 0.15 M) was added to a 25 mL volumetric flask. H_2SO_4 (0.05 M in H_2O) was then added until the graduation mark was reached, and the solution allowed to equilibrate for 30 min. The solution was wrapped in aluminium foil and stored in the dark.

Phenanthroline solution (B)

Phenanthroline (50 mg, 0.28 mmol) was added to a 25 mL volumetric flask and H_2O was added until the solution reached the graduation mark. The solution was allowed to equilibrate for 30 min.

Buffer Solution (C)

NaOAc (1.24 g, 12.5 mmol) was added to a 25 mL volumetric flask. Water (HPLC grade, 20 mL) was added followed by H_2SO_4 (95% w/w, 250 μL), water was then added until the graduation mark was reached, and the solution allowed to equilibrate for 30 min.

Measurements

Photon Flux Measurement (456 nm)

In a dark room, a microwave vial was charged with **solution A** (1.0 mL) and irradiated for 5 s.

After irradiation, 0.1 mL of the solution was immediately transferred to a 5 mL volumetric flask containing **solution B** (0.5 mL), **solution C** (2 mL) and water (HPLC grade, 1.5 mL). This was repeated 2 more times, irradiating for 10 s and 15 s, respectively. A control sample was also made, where **solution A** (0.1 mL) was added directly to a 5 mL volumetric flask

containing **solution B** (0.5 mL) and **solution C** (2 mL) and water (HPLC grade, 1.5 mL). The UV/Vis spectra of the samples were then taken (blank sample = solution C (2 mL) in H_2O (2 mL)) and the absorption measured at 510 nm.

The conversion was calculated using equation 1:

$$mol\ Fe^{2+} = \frac{V_1\, V_3 \Delta A}{V_2\, l\, \varepsilon} \qquad (1)$$

V_1 = irradiated volume (0.001 L).

V_2 = aliquot of irradiated solution added to quencher (0.0001 L).

V_3 = volume after complexation (0.0041 L).

ΔA = difference in absorbance between the irradiated and non-irradiated solutions.

l = path length (1 cm).

ε = molar absorptivity at 510 nm (11100 L mol^{-1} cm^{-1}).

Photon Flux was calculated using equation 2:

$$photon\ flux = \frac{mol\ Fe^{2+}}{\Phi\ t\ f} \qquad (2)$$

Φ = quantum yield for the ferrioxalate actinometer (1.14 at 436 nm).[24]

t = time.

f = fraction of light absorbed by ferrioxalate at 436 nm (0.9995, calculation shown below).

The moles of Fe^{2+} were plotted as a function of time, allowing the slope of the graph to be used to represent $\frac{mol\ Fe^{2+}}{t}$ which was determined to be 5.93×10^{-7} mol s^{-1}. The photon flux was then calculated to be 5.20×10^{-7} einstein s^{-1}.

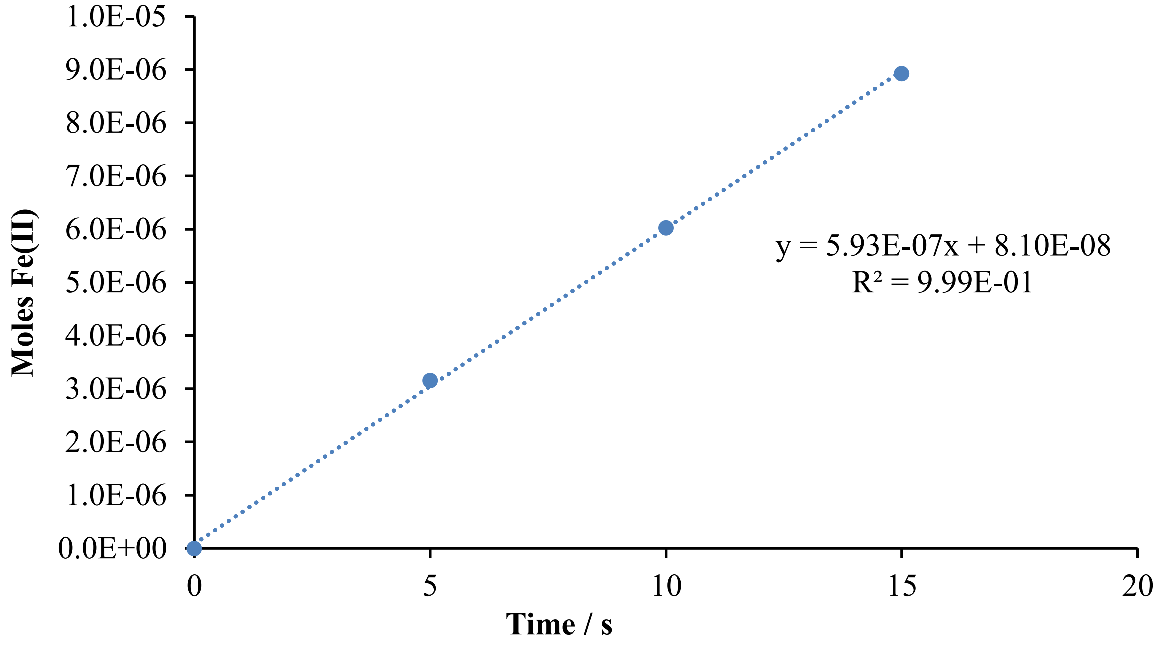

Figure S19. Moles of actinometer converted to Fe(II) as a function of time.

Fraction of Light Absorbed (f) by Ferrioxalate Measurement

In a dark room, a quartz cuvette was charged with solution A directly. The UV/Vis spectrum of the sample was taken and the absorbance at 390 nm measured. The fraction of light absorbed was calculated using eq. 3:

$$fraction\ of\ light\ absorbed\ (f) = 1 - 10^{-A} \quad (3)$$

A = absorbance of the actinometer at 436 nm (3.3112).

Quantum Yield Measurement

A dry tube equipped with a stirring bar was charged with alkoxysulfonium salt **1a** (36.4 mg, 0.10 mmol, 1.0 equiv.) and Na_2HPO_4 (7 mg, 0.05 mmol, 0.5 equiv.). The tube was capped with a Supelco aluminium crimp seal with septum (PTFE/butyl), then evacuated under high vacuum and backfilled with N_2 (3 times). Degassed $Ru(bpy)_3(PF_6)_2$ in acetone stocking solution (1.0 mL, 0.1 M) and 4-chlorostyrene (24 µL, 0.20 mmol), and H_2O (4 µL, 0.20 mmol, 2.0 equiv.) were sequentially added. The vial was purged with a stream of N_2 and the lid sealed with parafilm and placed 2 cm from Kessil blue LEDs (λ_{max} = 456 nm) set to 25% intensity. After irradiation, mesitylene (7 µL, 0.05 mmol, 0.5 equiv.) was added as an internal standard directly to the reaction solution. The moles of product **4a** formed was quantified by ^{1}H NMR spectroscopy; this was used to calculate the quantum yield using a modified eq 2.

$$\Phi = \frac{mol\ product}{flux\ t\ f} \qquad (2)$$

t = reaction time.

photon flux = 5.20 x 10^{-7} einstein s^{-1}.

f = fraction of light absorbed by reaction mixture at 456 nm (0.947 based on an absorbance of 1.2764).

Reactions performed for two different time periods (30 s and 60 s) were carried out, each averaged over two runs.

The moles of product **4a** formed were plotted as a function of time, allowing the slope of the graph to be used to represent $\frac{mol\ product}{t}$ which was calculated to be 5.00 × 10^{-7} mol s^{-1}, and hence the quantum yield Φ was calculated to be 1.02.

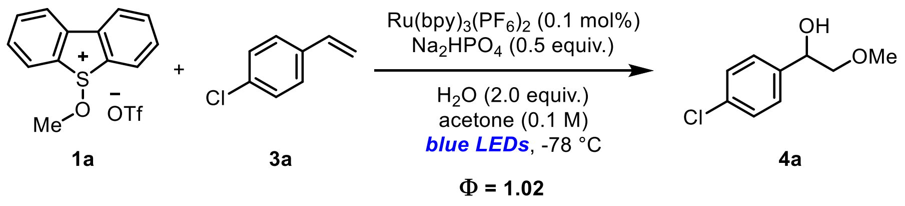

A quantum yield (Φ) of 1.02 for the coupling of **1a** and **3a** to give **4a**, suggests the involvement – at least partially – of a radical-chain mechanism.

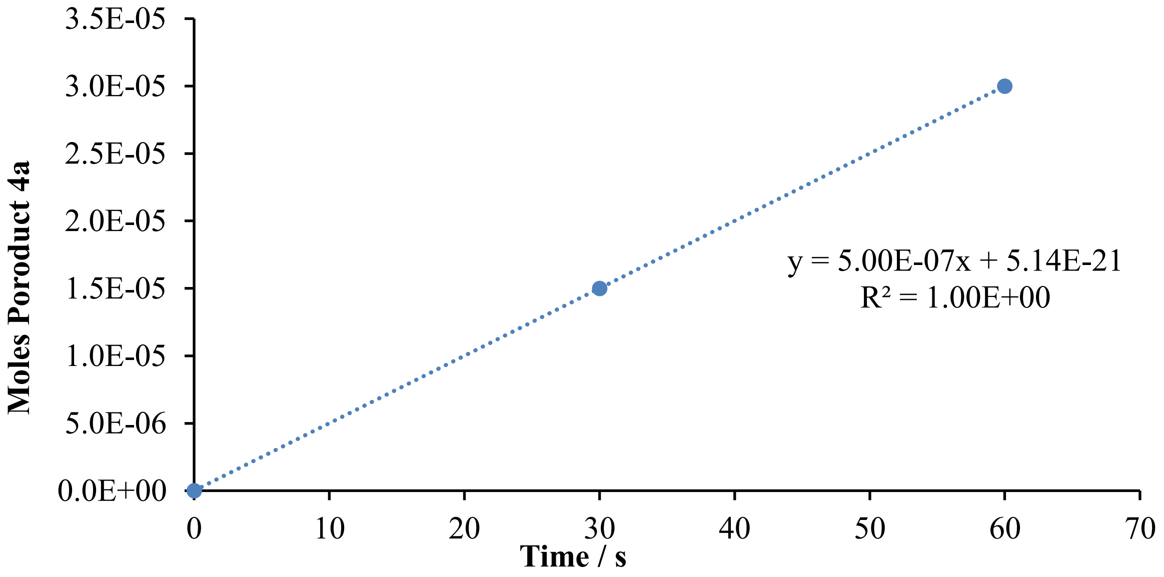

Figure S20. The moles of product **4a** formed were plotted as a function of time.

7.7 X-Ray Structures

***tert*-Butyl 7-chloro-5-hydroxy-5-(methoxymethyl)-2,3,4,5-tetrahydro-1*H*-benzo[*b*]azepine-1-carboxylate (4be)**

CCDC: 2432415.

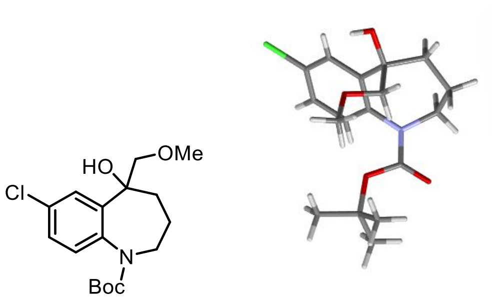

Crystallized from benzene by slow evaporation at room temperature.

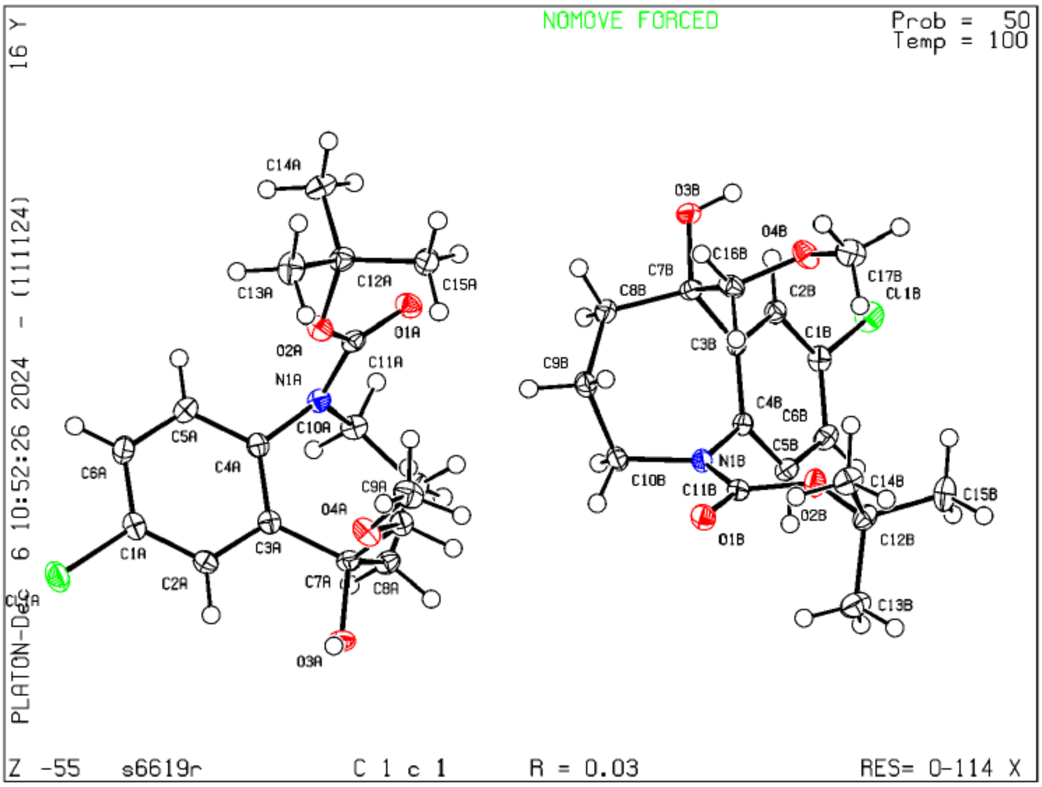

Datablock: s6619r

Bond precision:	C-C = 0.0030 A		Wavelength=1.54184
Cell:	a=10.9541(1)	b=11.3761(1)	c=28.0222(3)
	alpha=90	beta=93.322(1)	gamma=90
Temperature: 100 K			

	Calculated	Reported
Volume	3486.12(6)	3486.12(6)
Space group	C c	C 1 c 1
Hall group	C -2yc	C -2yc
Moiety formula	C17 H24 Cl N O4	C17 H24 Cl N O4
Sum formula	C17 H24 Cl N O4	C17 H24 Cl N O4
Mr	341.82	341.82
Dx,g cm-3	1.303	1.303
Z	8	8
Mu (mm-1)	2.105	2.105
F000	1456.0	1456.0
F000'	1463.04	
h,k,lmax	13,14,35	13,14,35
Nref	7551[3779]	7287
Tmin,Tmax	0.747,0.863	0.217,1.000
Tmin'	0.419	

Correction method= # Reported T Limits: Tmin=0.217 Tmax=1.000 AbsCorr = GAUSSIAN

Data completeness= 1.93/0.97 Theta(max)= 79.766

R(reflections)= 0.0281(7237) wR2(reflections)= 0.0731(7287)

S = 1.061 Npar= 426

(4*S*)-4-(2-(4-Chlorophenyl)-2-hydroxyethoxy)dihydrofuran-2(3*H*)-one (4cf)

CCDC: 2432693.

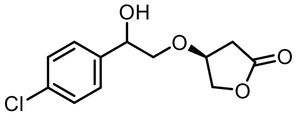

d.r. = 1:1

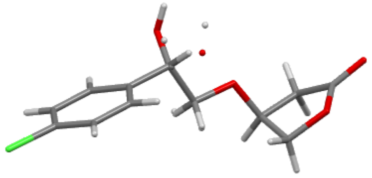

Crystallized from benzene by slow evaporation at room temperature.

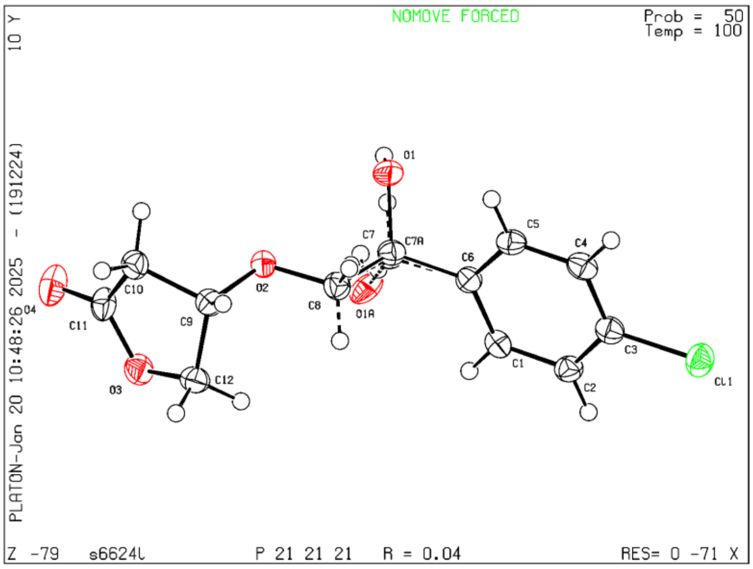

Datablock: s6624l

```
Bond precision:    C-C = 0.0050 A          Wavelength=1.54184

Cell:              a=5.42852(13)      b=7.5950(2)      c=28.9338(7)
                   alpha=90           beta=90          gamma=90
Temperature:       100 K
```

	Calculated	Reported
Volume	1192.93(5)	1192.94(5)
Space group	P 21 21 21	P 21 21 21
Hall group	P 2ac 2ab	P 2ac 2ab
Moiety formula	C12 H13 Cl O4	C12 H13 Cl O4
Sum formula	C12 H13 Cl O4	C12 H13 Cl O4
Mr	256.67	256.67
Dx,g cm-3	1.429	1.429
Z	4	4
Mu (mm-1)	2.865	2.865
F000	536.0	536.0
F000'	539.08	
h,k,lmax	6,9,36	6,9,36
Nref	2488[1490]	2460
Tmin,Tmax	0.765,0.934	0.481,1.000
Tmin'	0.207	

```
Correction method= # Reported T Limits: Tmin=0.481 Tmax=1.000
AbsCorr = MULTI-SCAN

Data completeness= 1.65/0.99       Theta(max)= 75.884

R(reflections)= 0.0425( 2260)                      wR2(reflections)=
                                                   0.1133( 2460)
S = 1.058                  Npar= 175
```

8 NMR Spectra

1a – ^{1}H NMR (400 MHz, acetone-d_6)

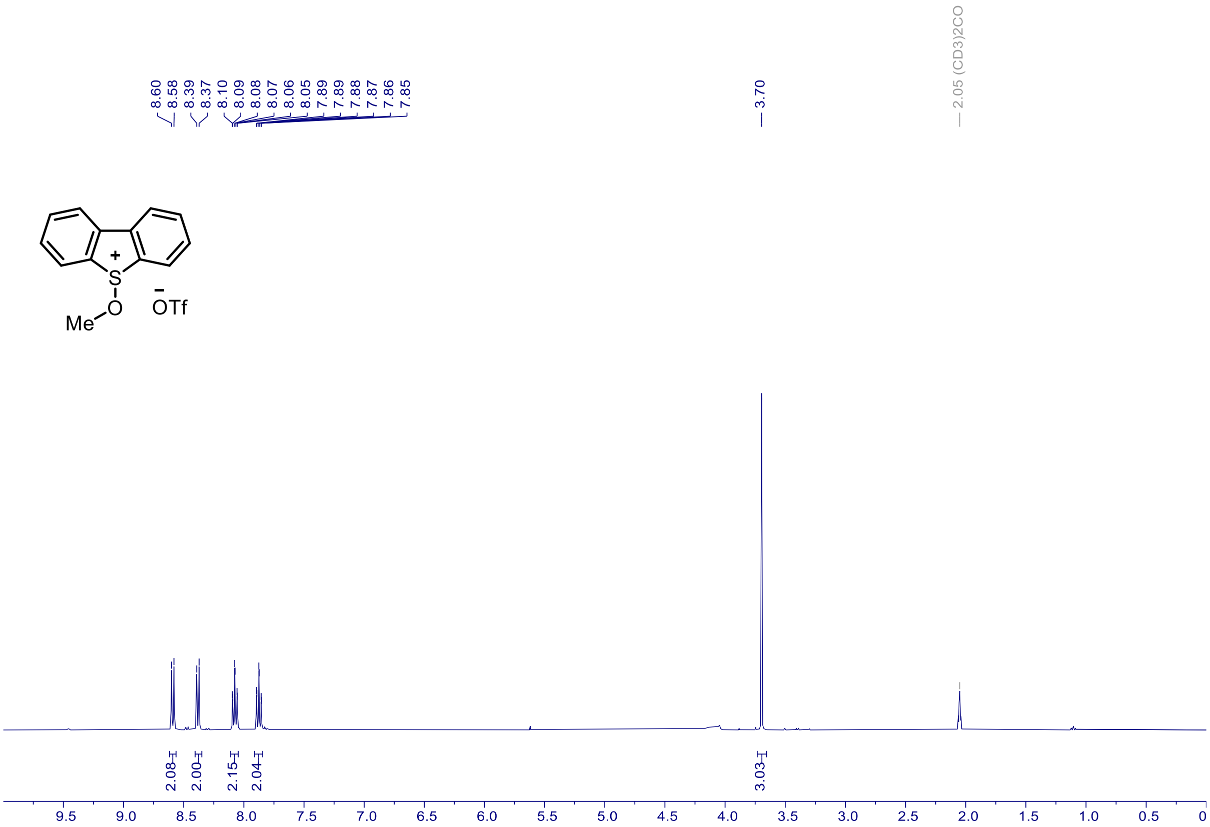

1a – ^{13}C NMR (101 MHz, acetone-d_6)

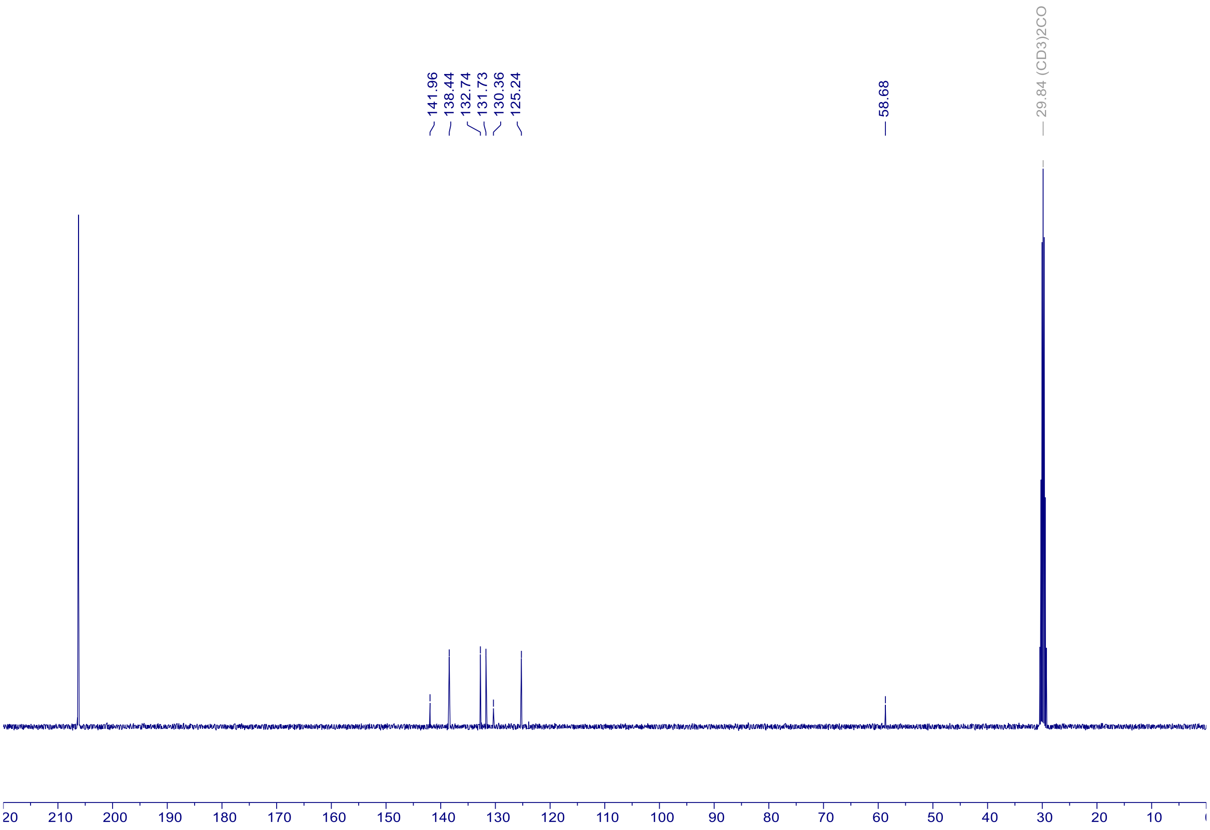

1a – ^{19}F NMR (376 MHz, acetone-d_6)

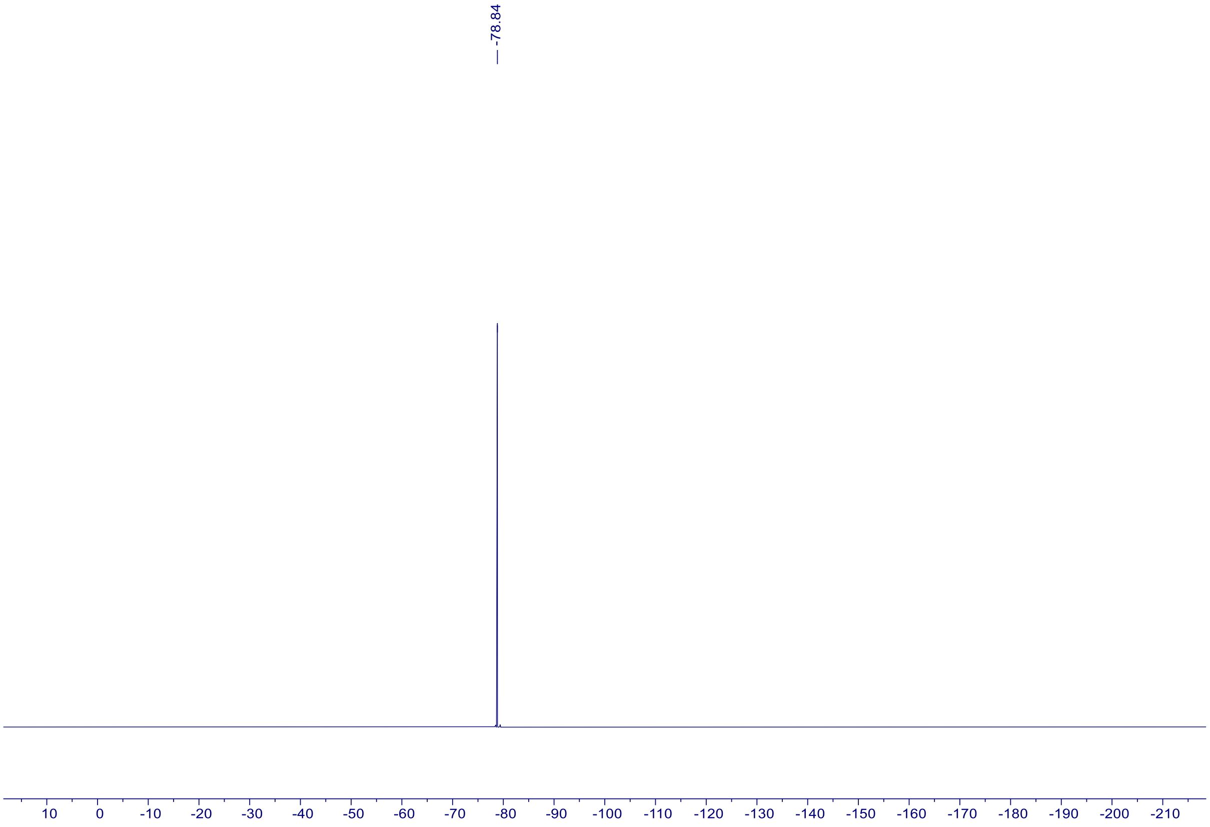

1a$_1$ – ^{1}H NMR (500 MHz, acetone-d_6)

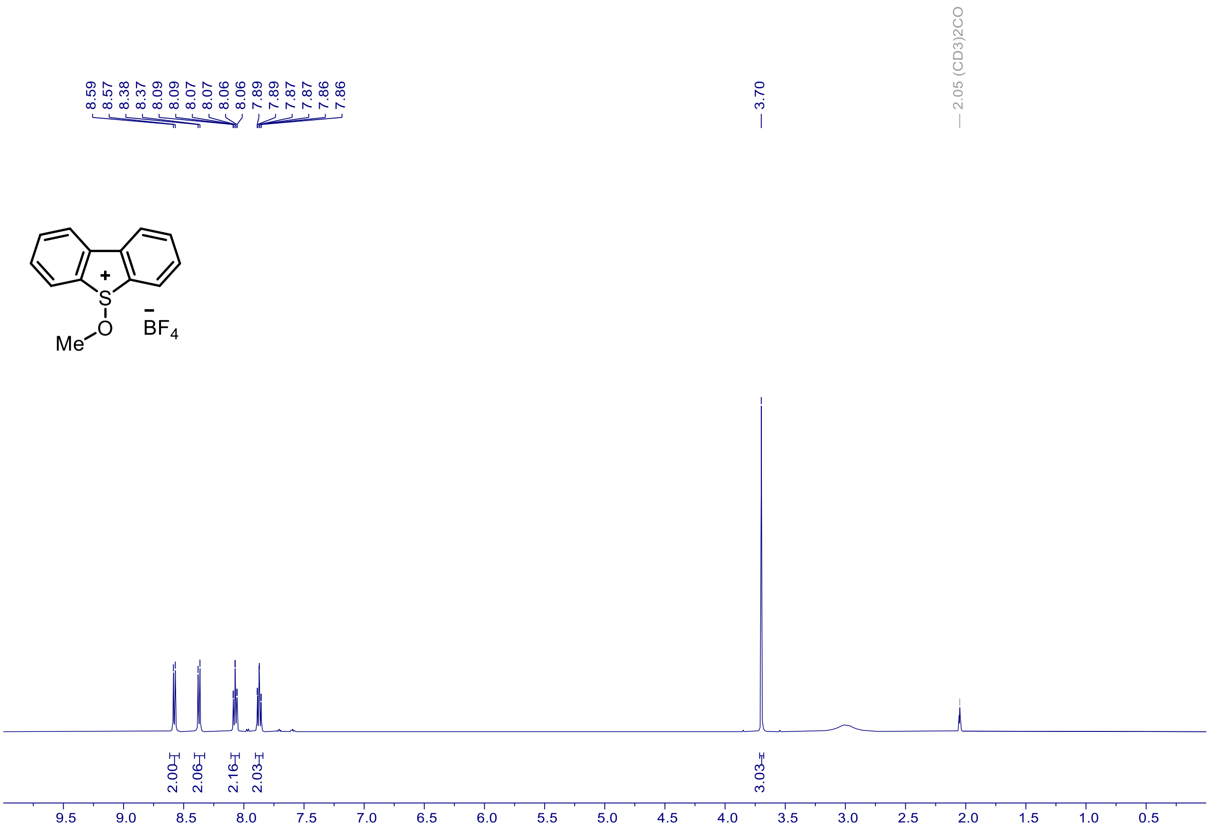

1a₁ – ^{13}C NMR (126 MHz, acetone-d_6)

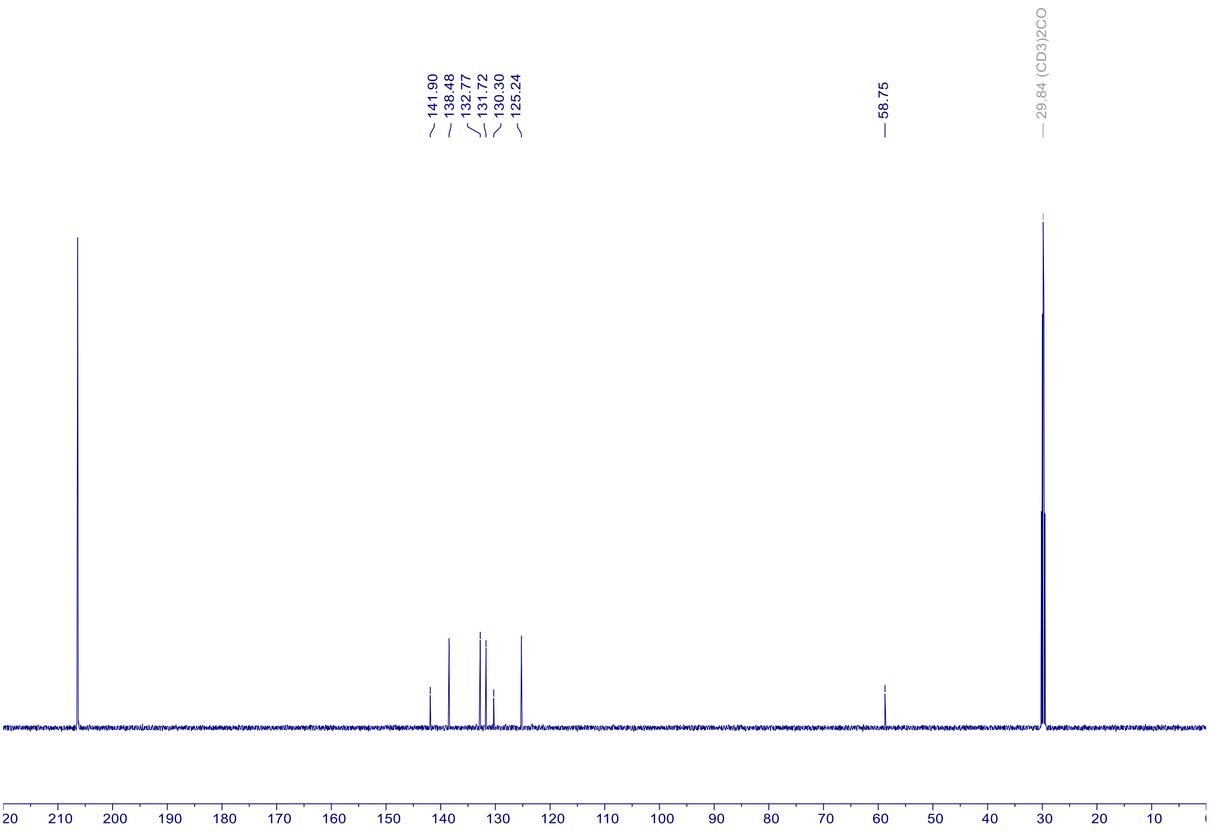

1a₁ – ^{19}F NMR (471 MHz, acetone-d_6)

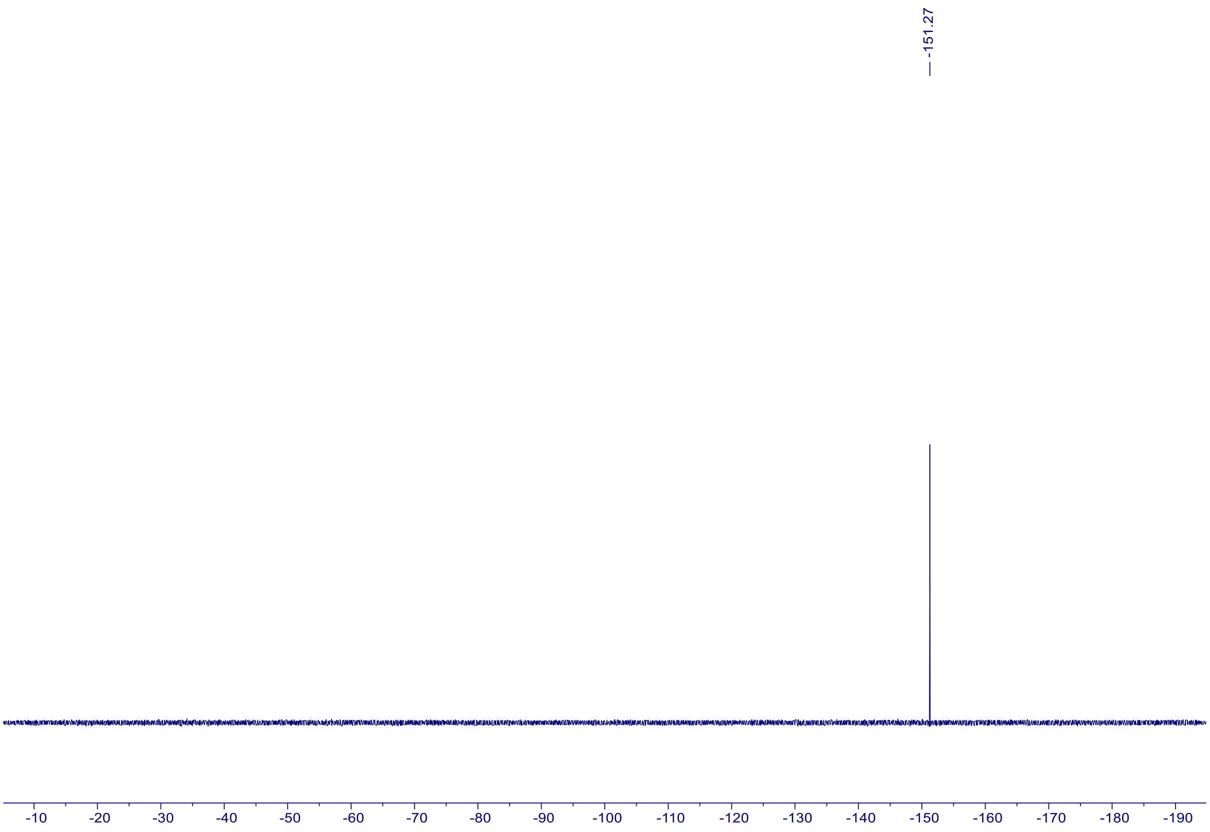

1a₂ – ^{1}H NMR (500 MHz, acetone-d_6)

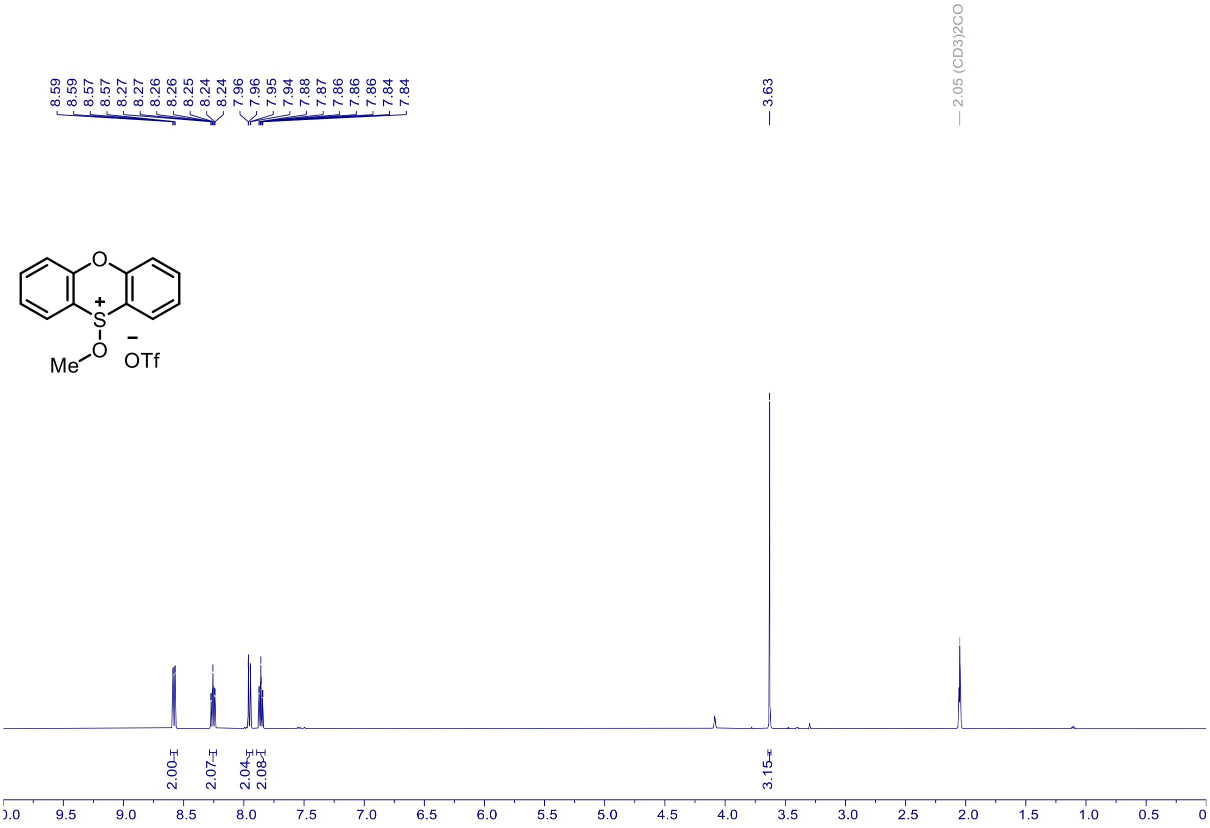

1a₂ – ^{13}C NMR (126 MHz, acetone-d_6)

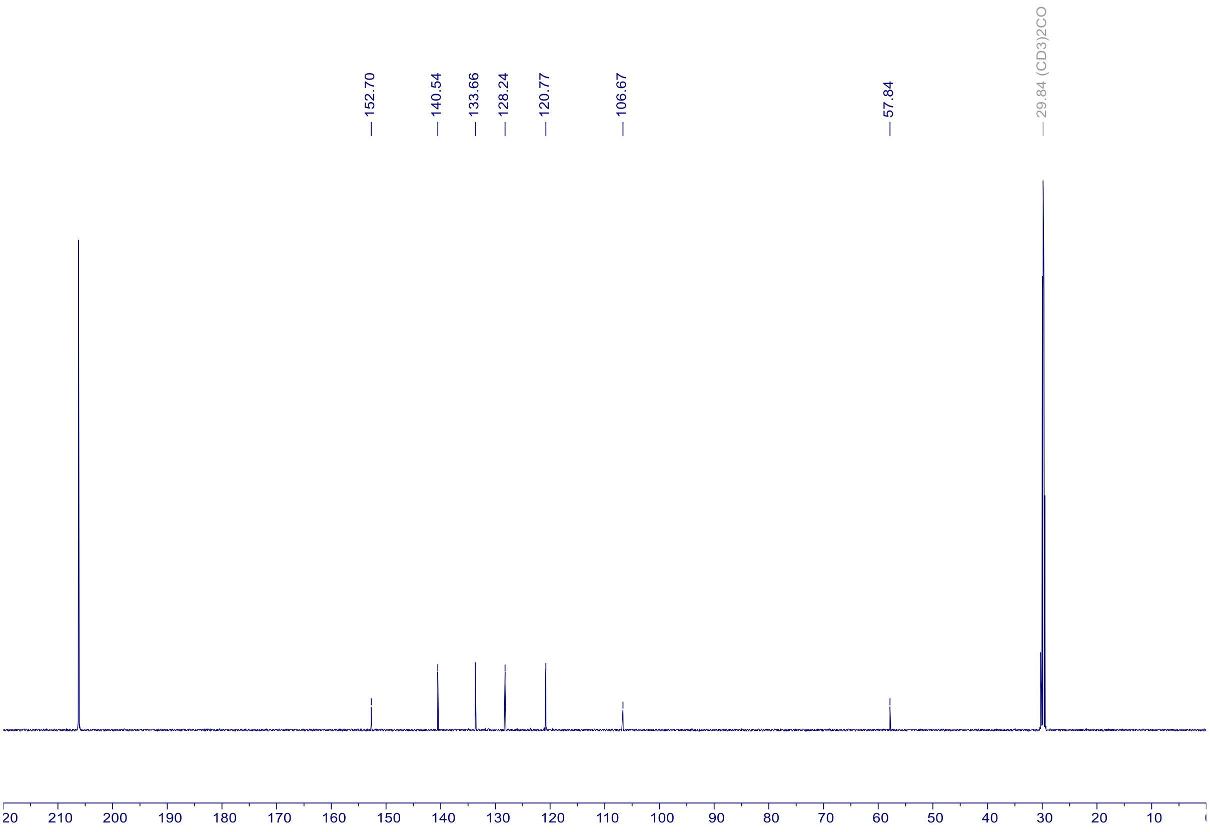

1a2 – ^{19}F NMR (471 MHz, acetone-d_6)

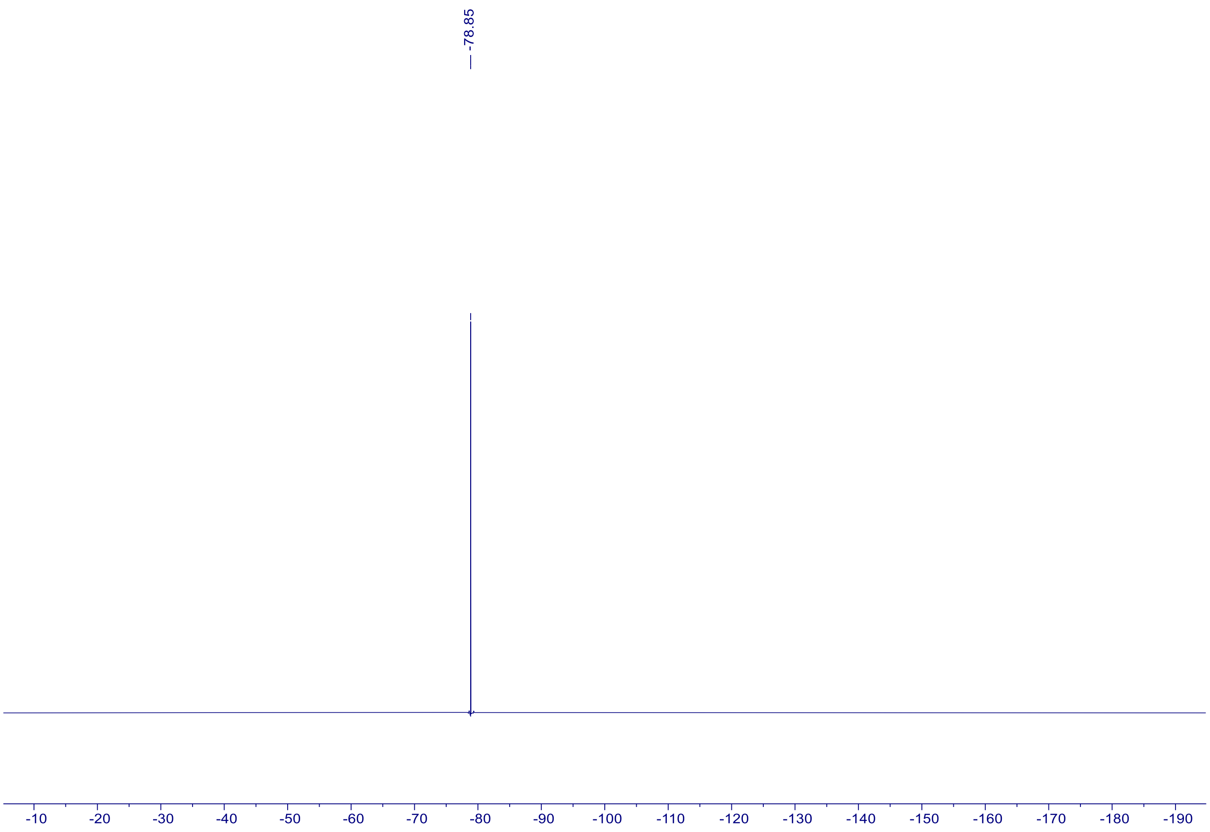

1a3 – ^{1}H NMR (500 MHz, acetone-d_6)

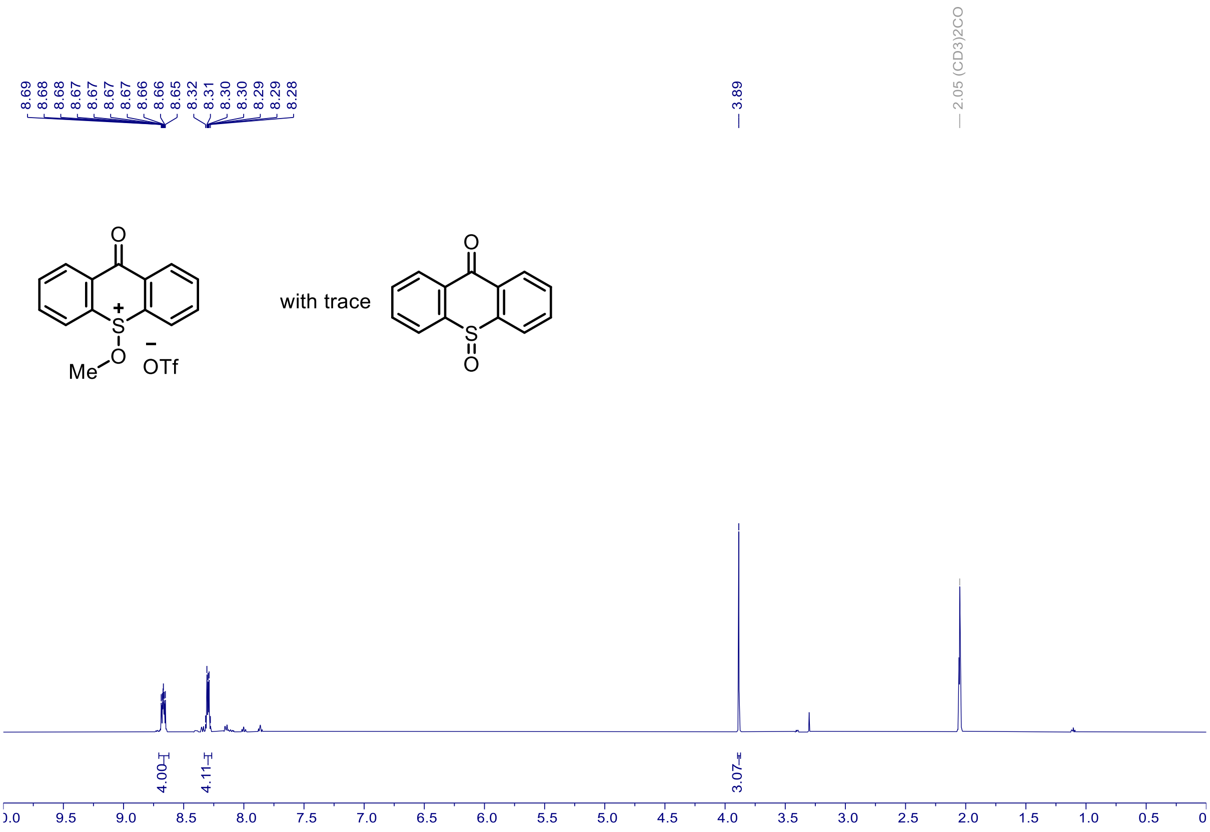

1a3 – ^{13}C NMR (126 MHz, acetone-d_6)

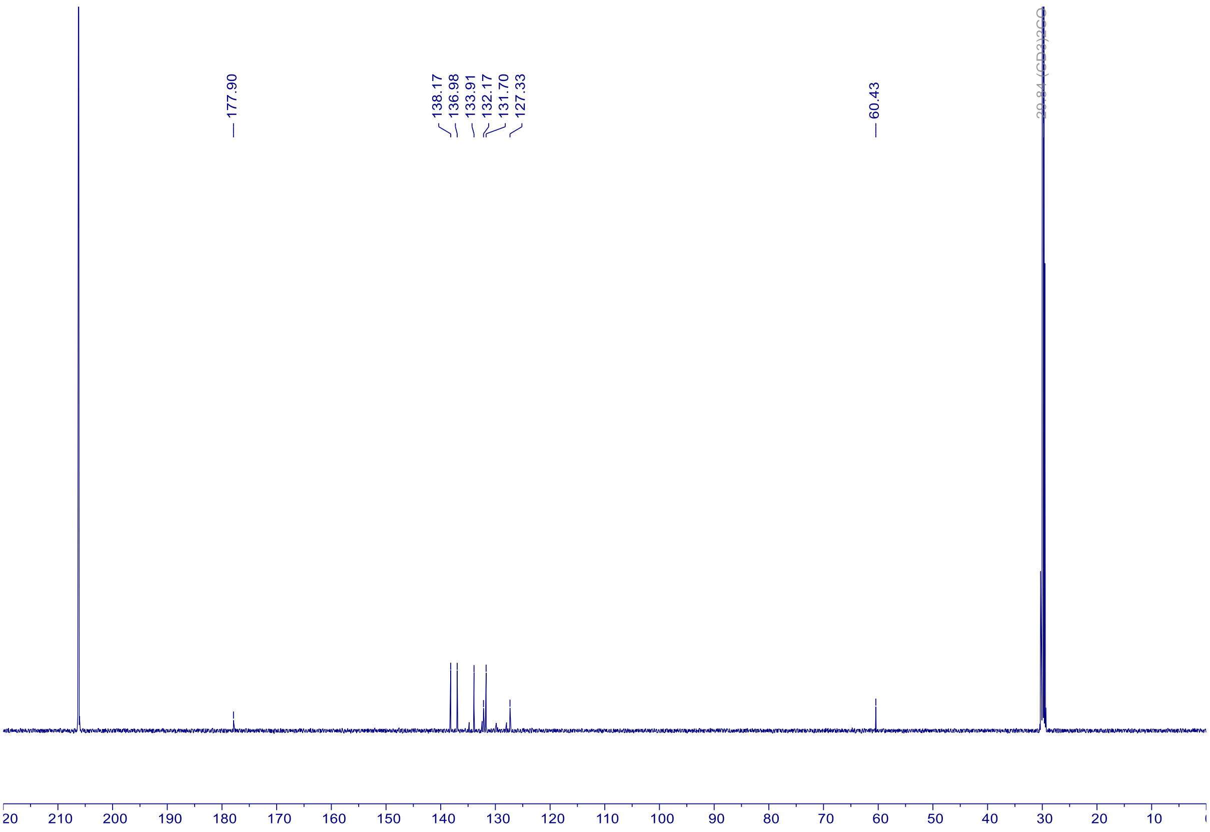

1a3 – ^{19}F NMR (471 MHz, acetone-d_6)

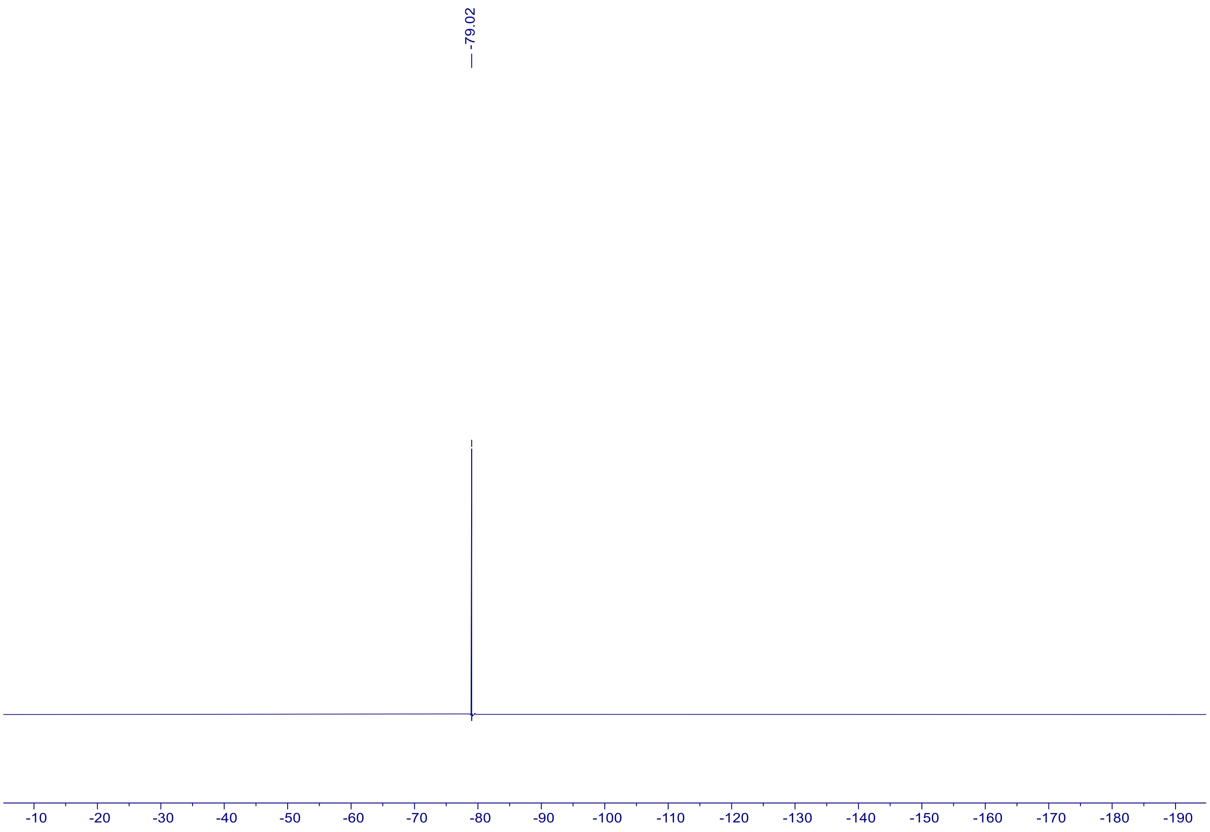

1a4 – ^{1}H NMR (400 MHz, acetone-d_6)

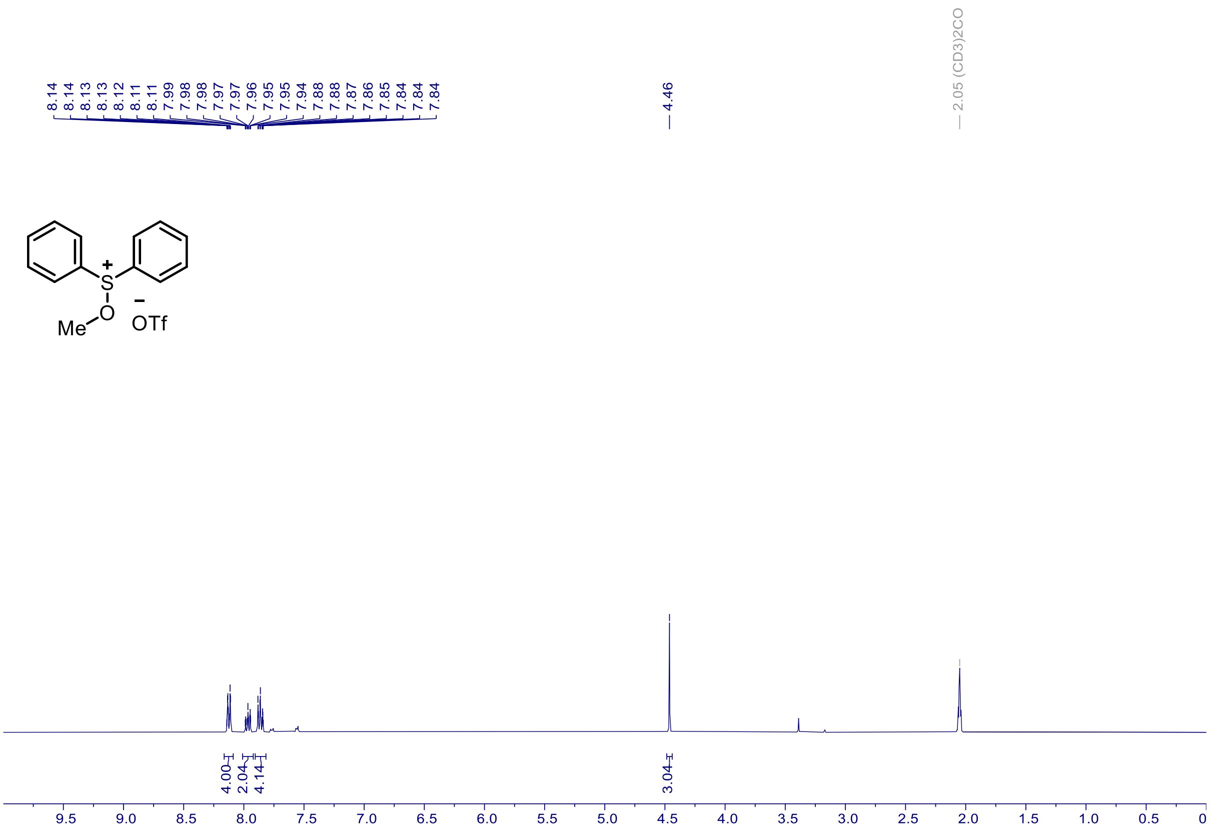

1a4 – ^{13}C NMR (101 MHz, acetone-d_6)

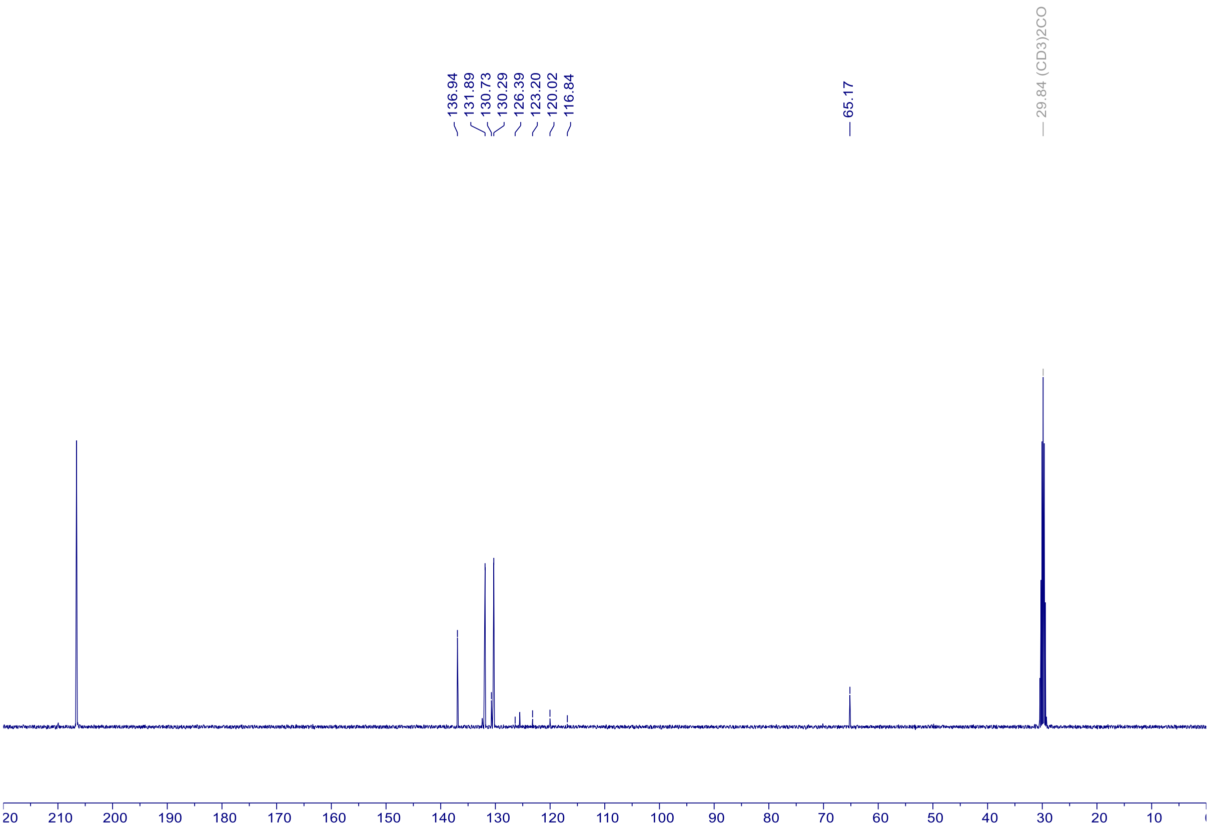

1a₄ – ^{19}F NMR (376 MHz, acetone-d_6)

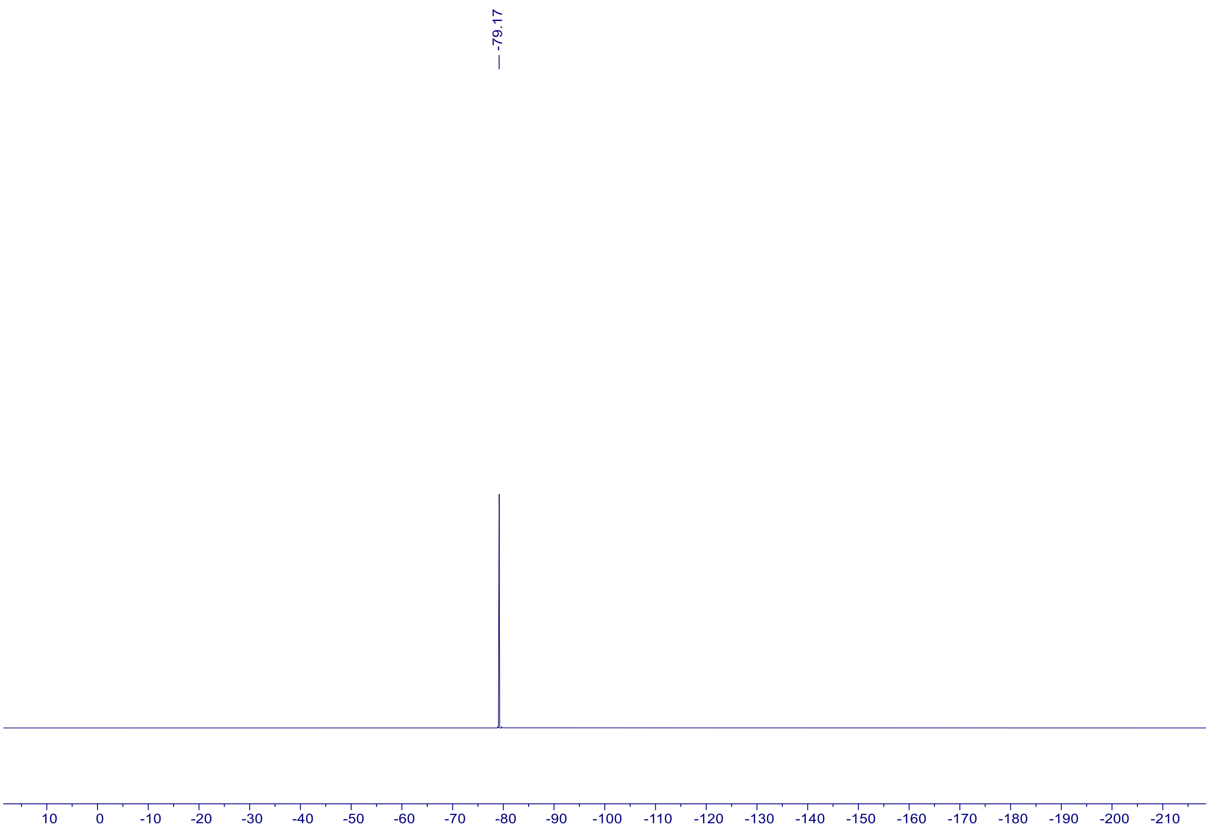

1b – ^{1}H NMR (500 MHz, acetone-d_6)

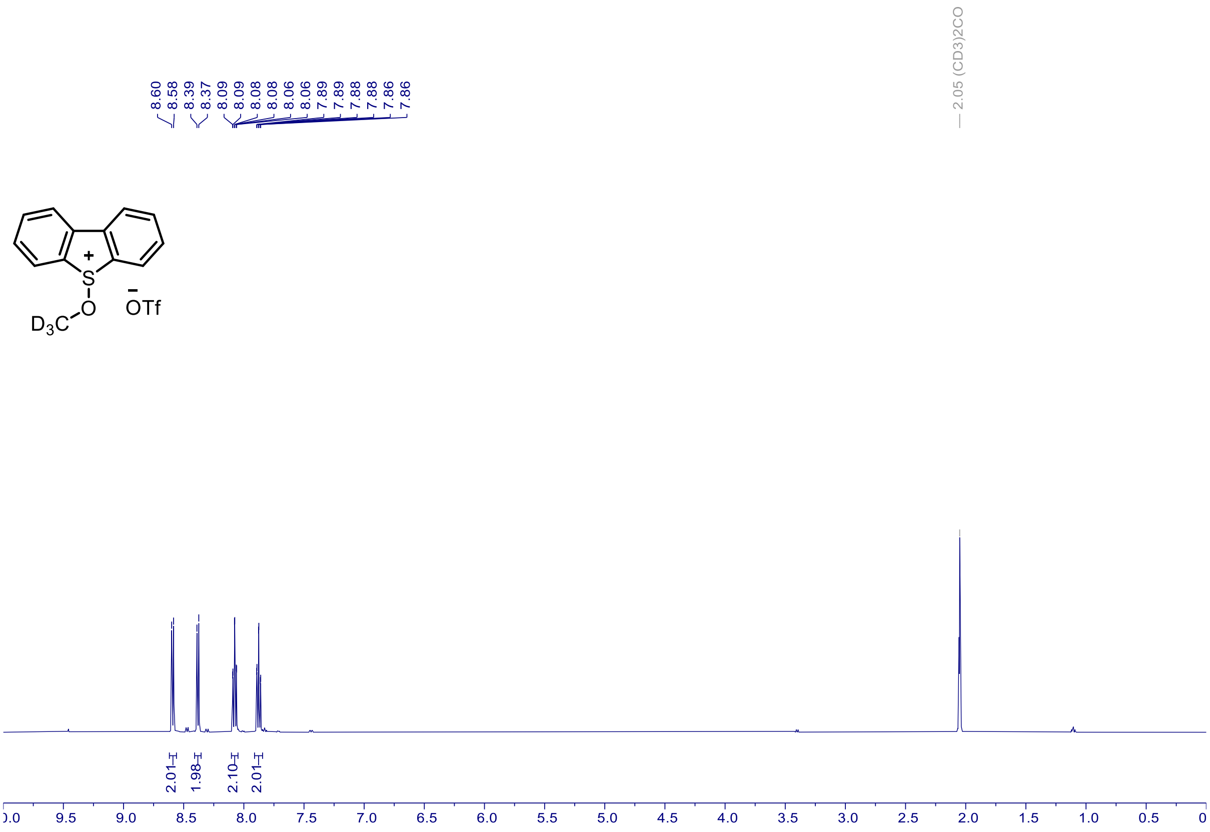

1b – ^{13}C NMR (126 MHz, acetone-d_6)

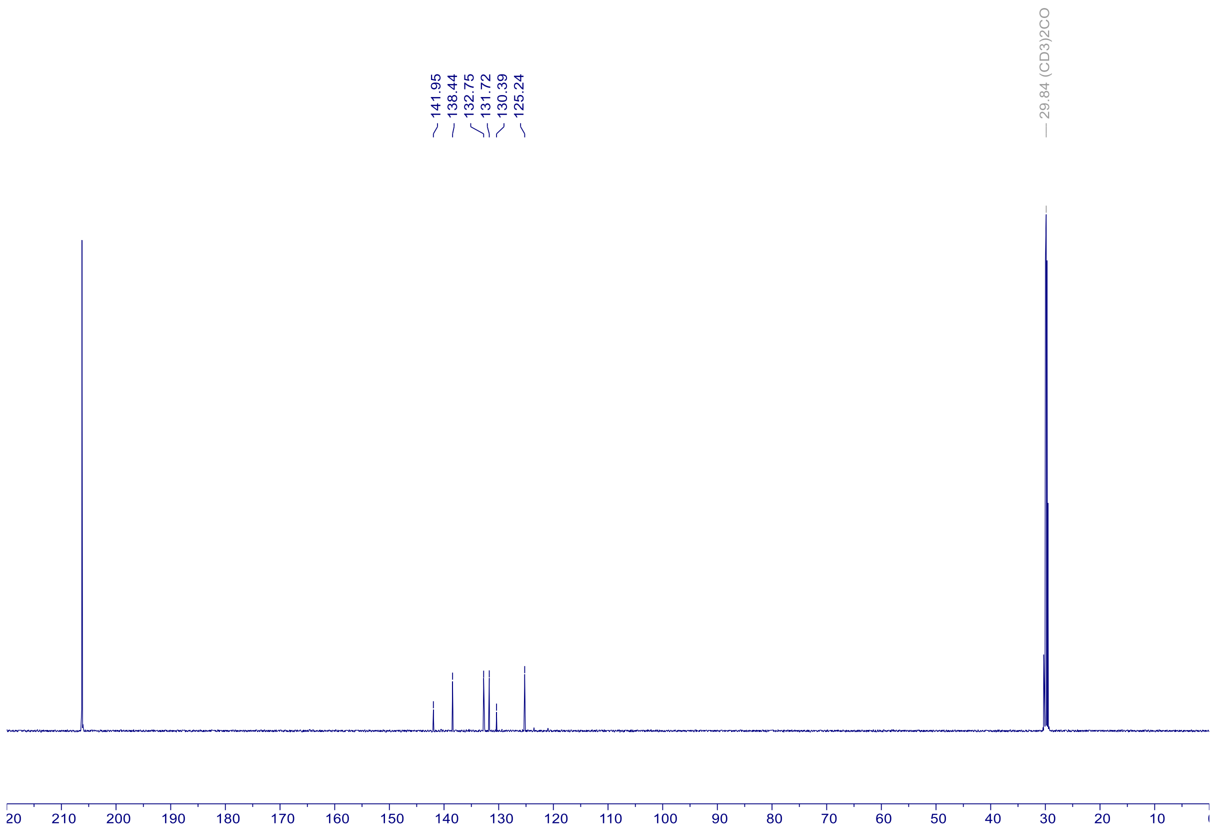

1b – ^{19}F NMR (471 MHz, acetone-d_6)

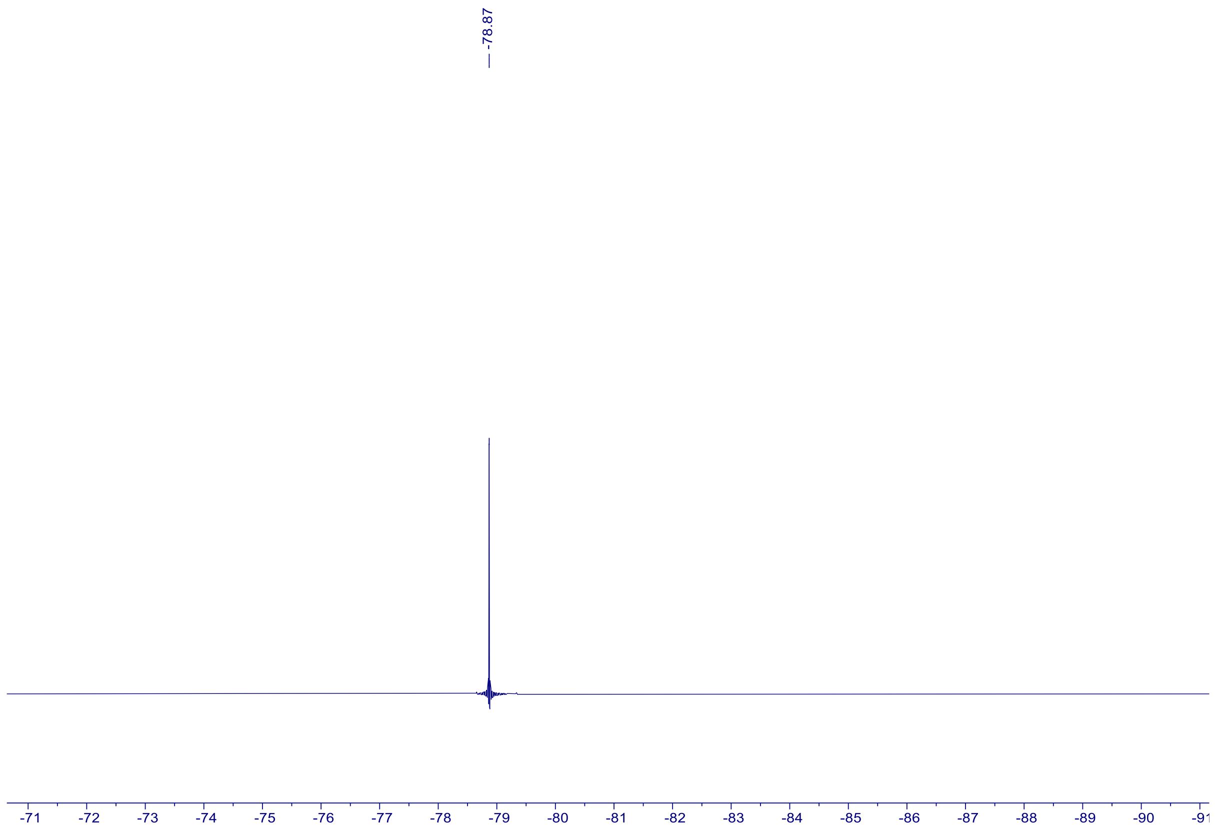

1b – ^{2}H NMR (61 MHz, acetone-d_6)

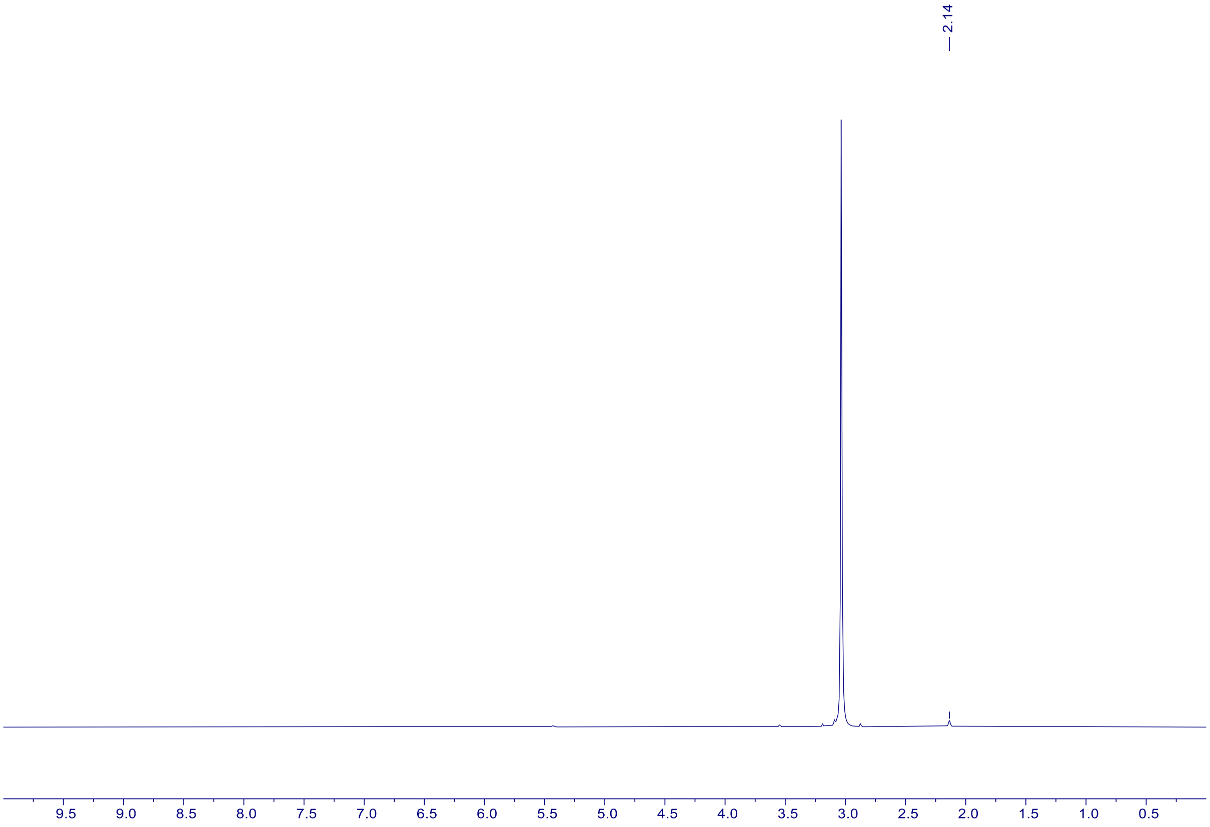

1c – ^{1}H NMR (400 MHz, acetone-d_6)

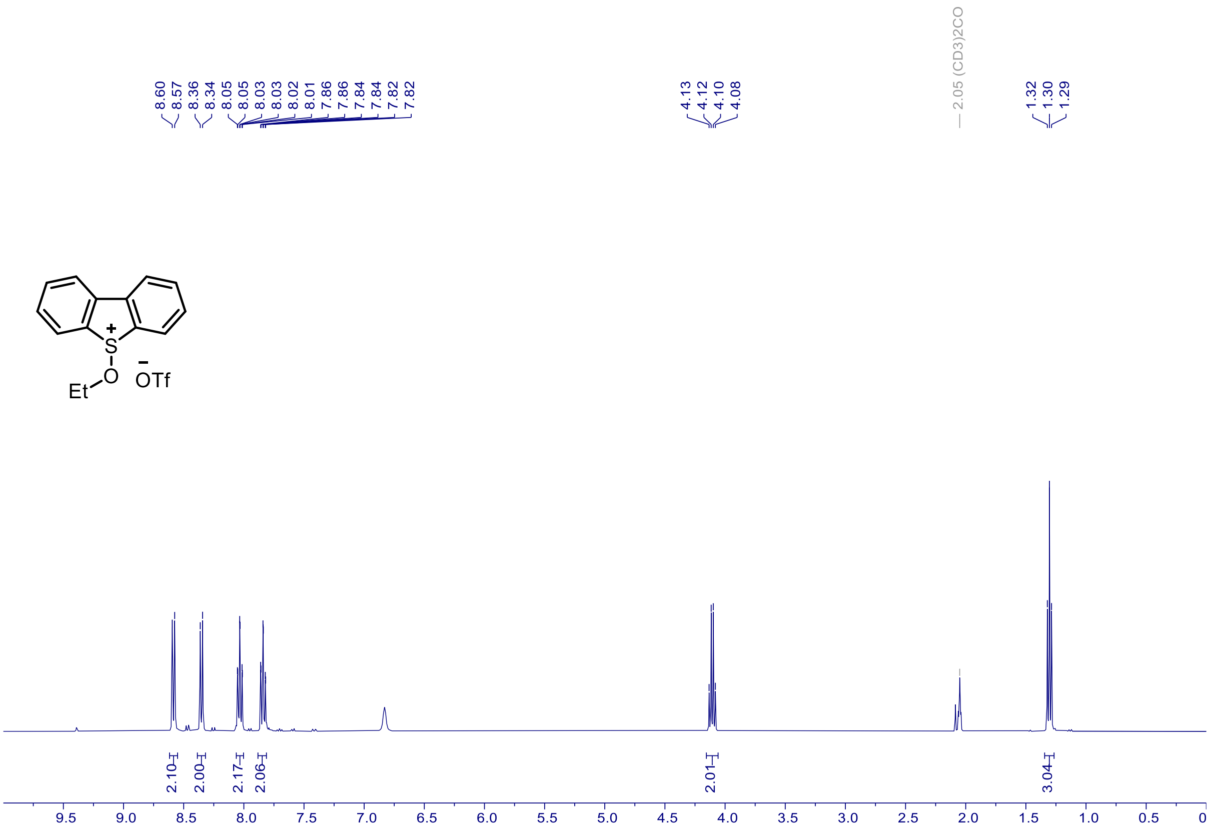

1c – ^{13}C NMR (101 MHz, acetone-d_6)

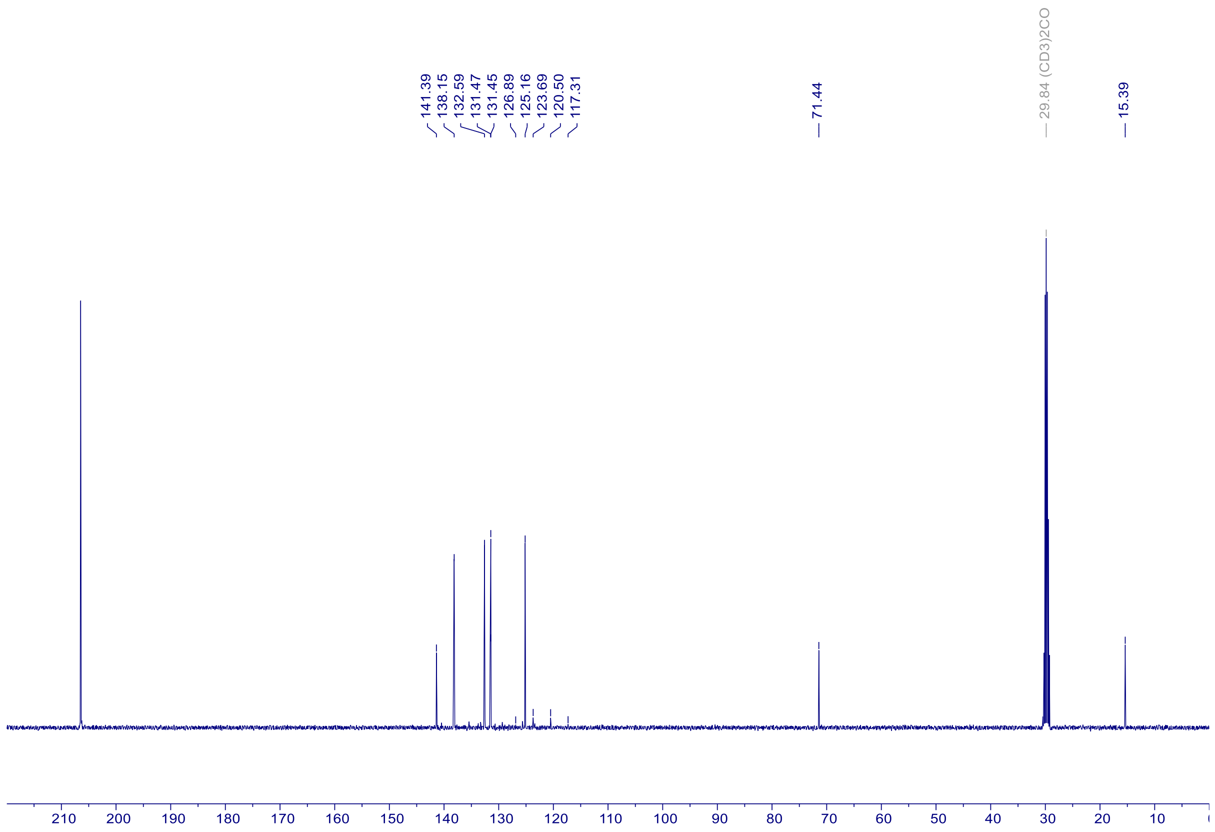

1c – ^{19}F NMR (376 MHz, acetone-d_6)

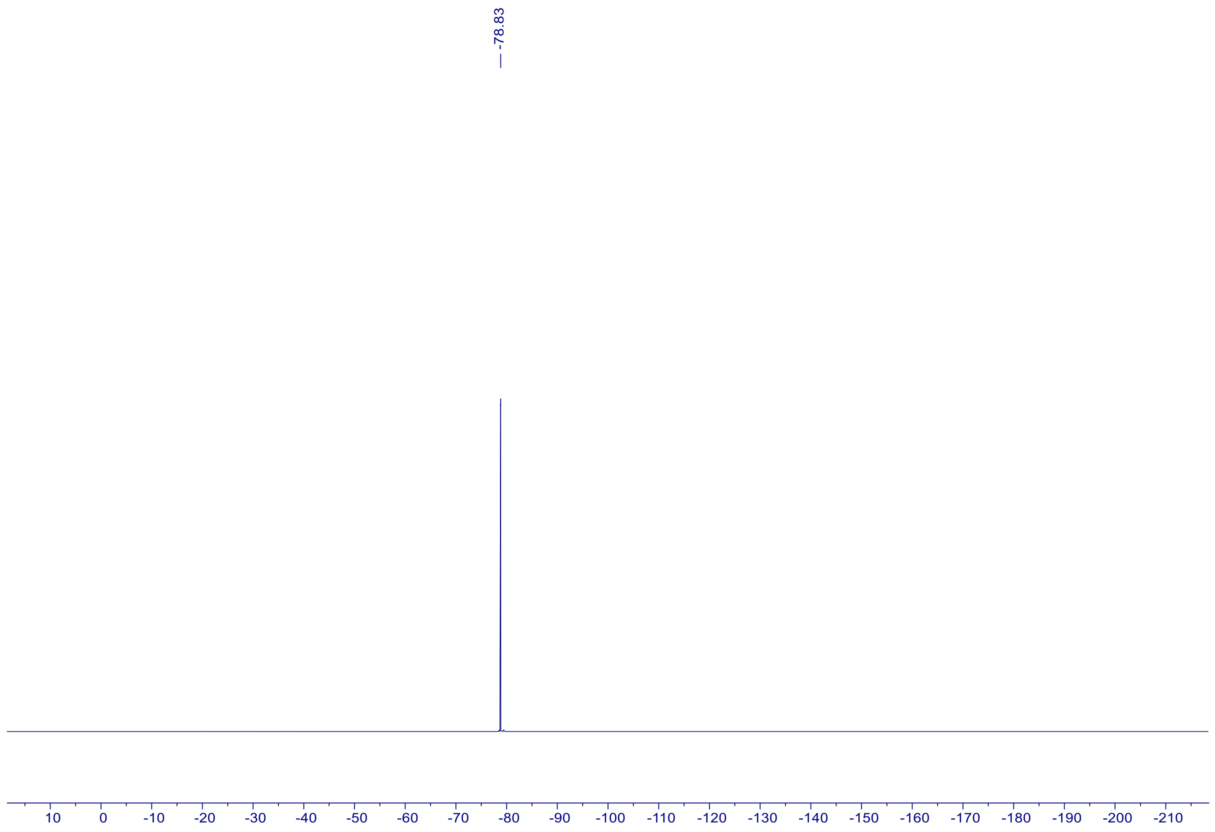

1d – ^{1}H NMR (500 MHz, acetone-d_6)

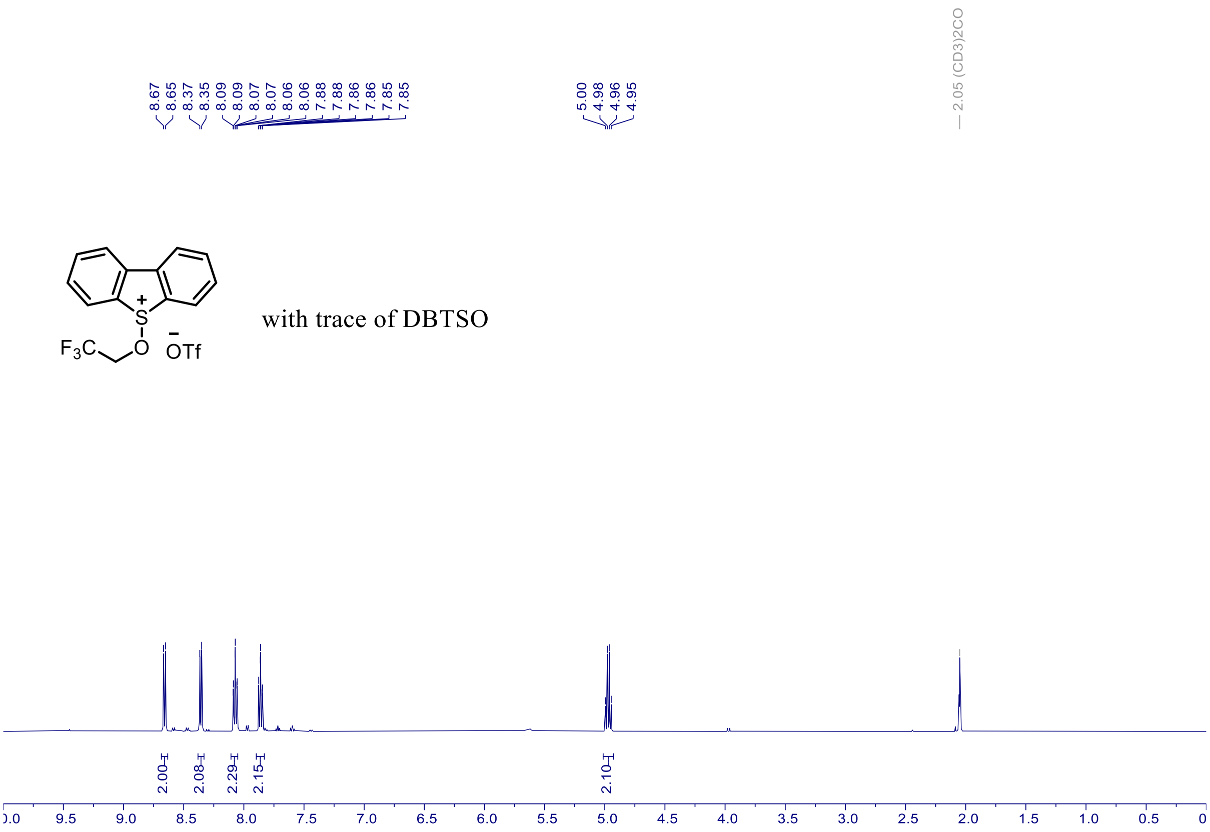

1d – ^{13}C NMR (126 MHz, acetone-d_6)

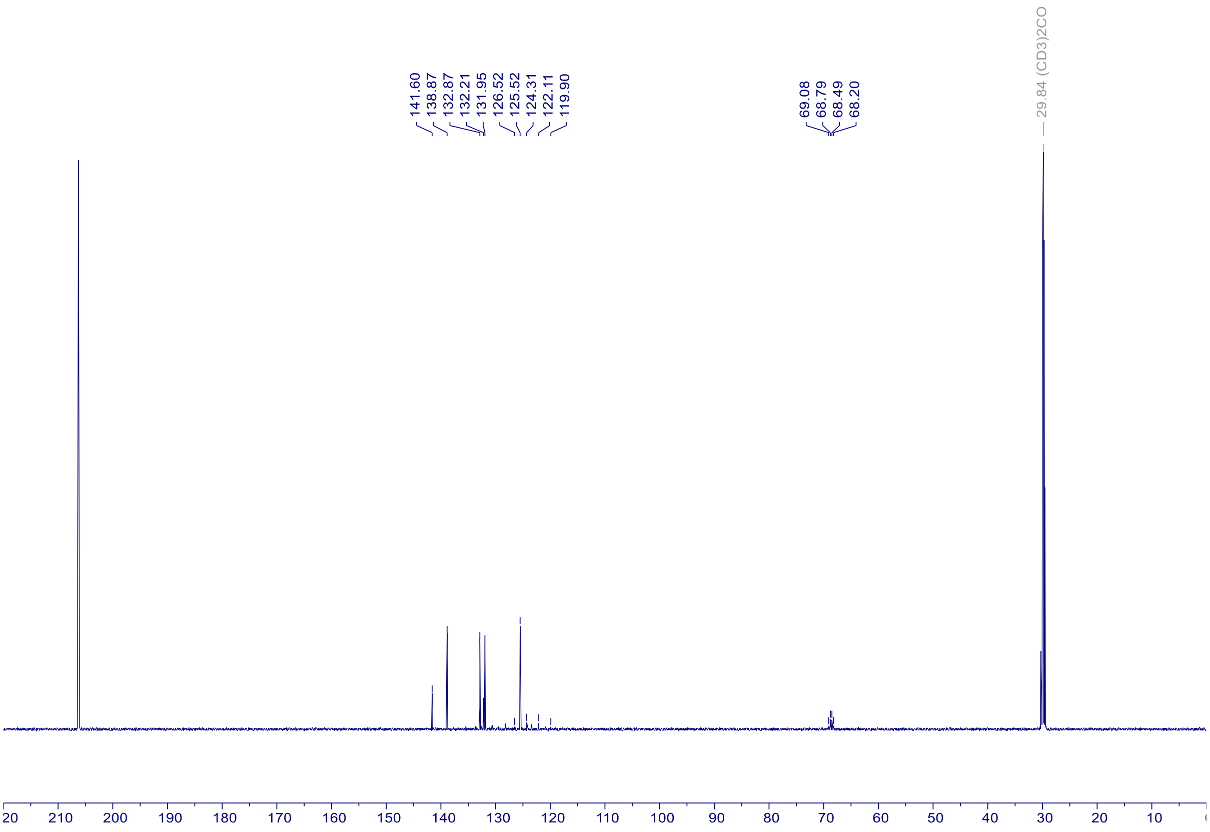

1d – ^{19}F NMR (471 MHz, acetone-d_6)

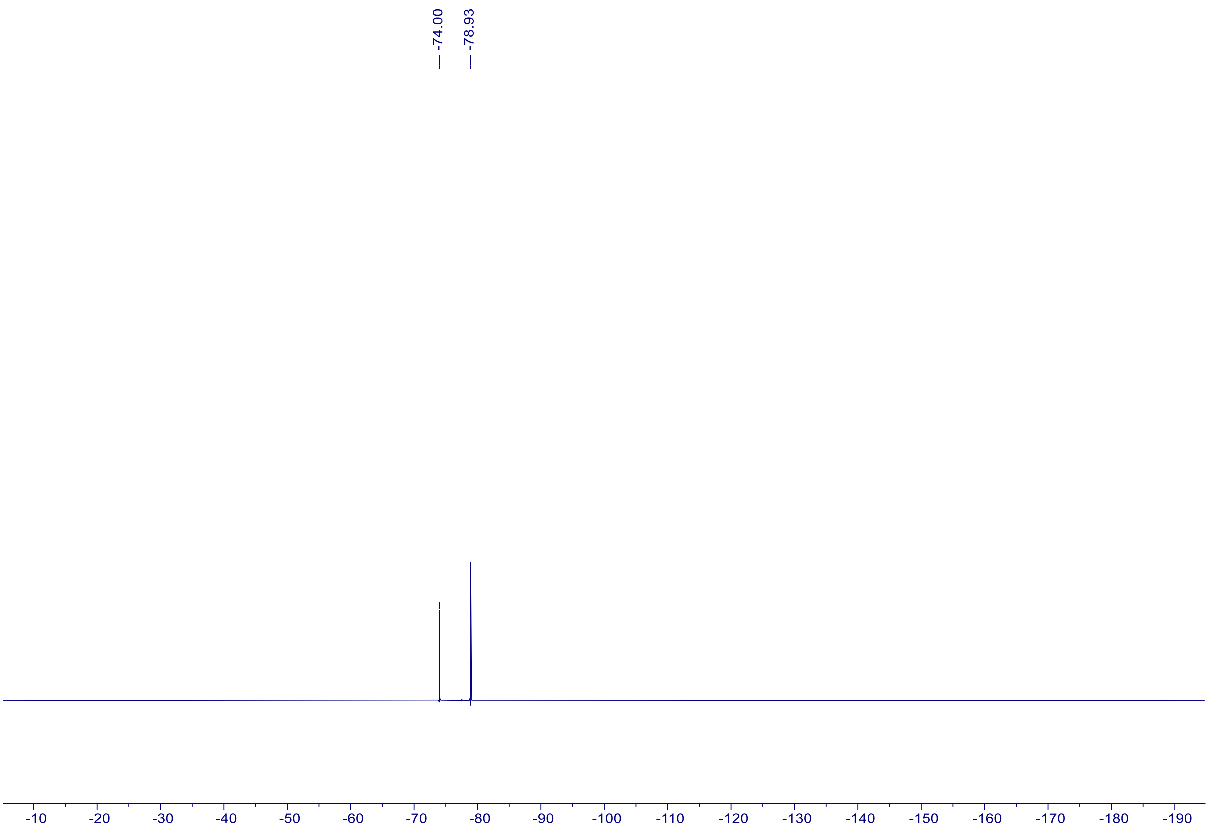

1e – ^{1}H NMR (400 MHz, acetone-d_6)

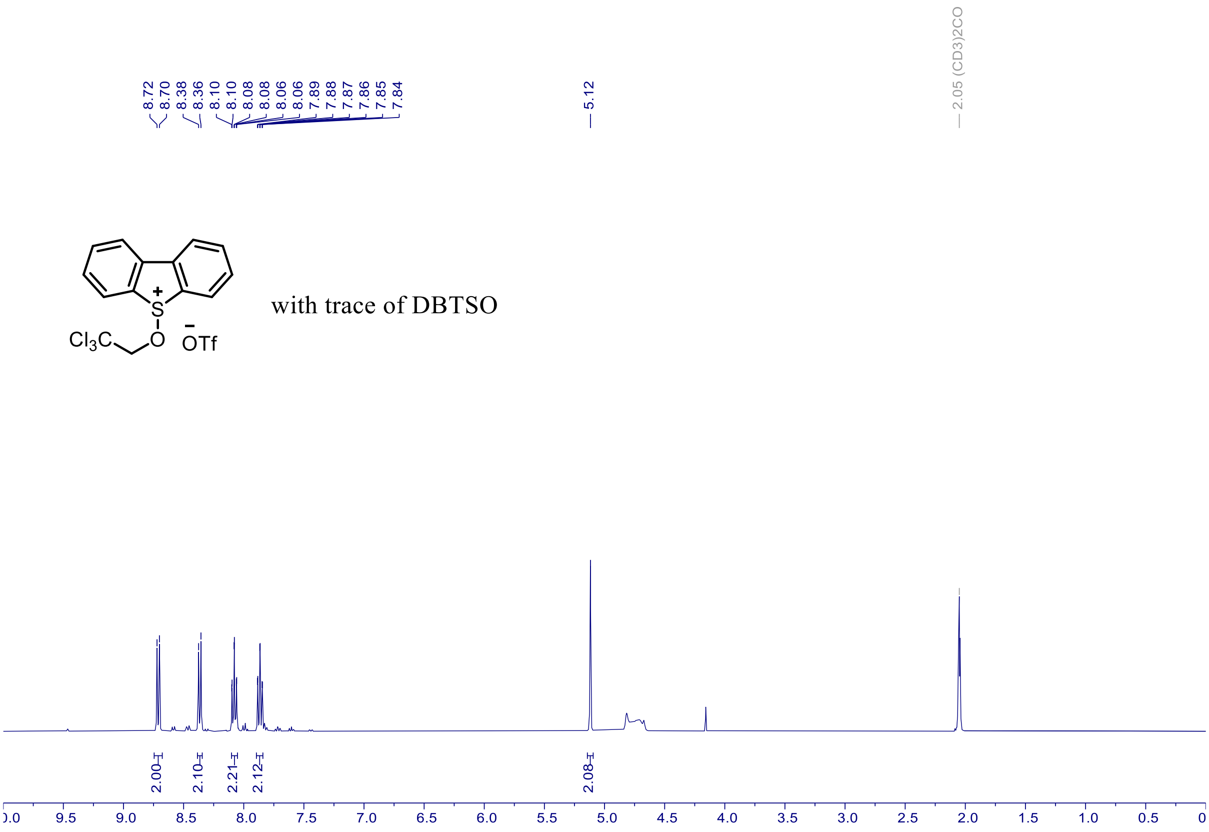

1e – ^{13}C NMR (101 MHz, acetone-d_6)

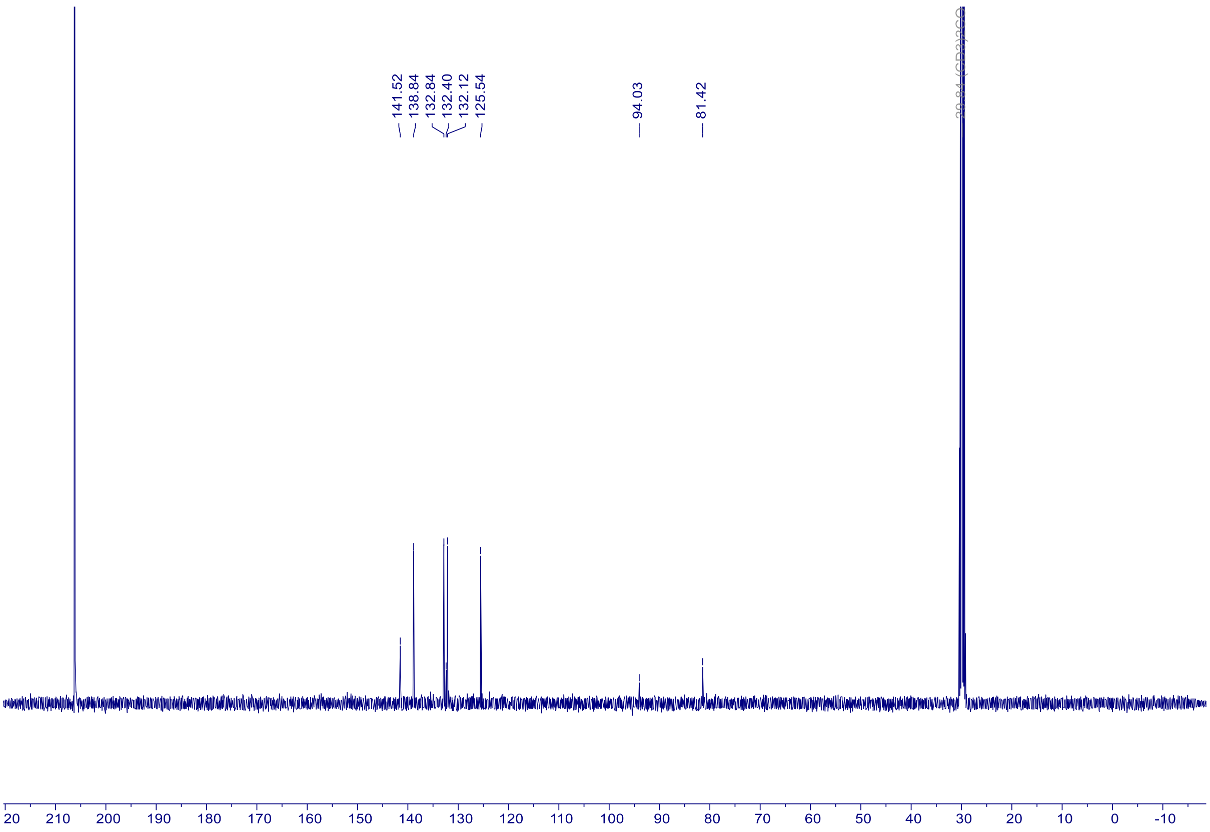

1e – ^{19}F NMR (376 MHz, acetone-d_6)

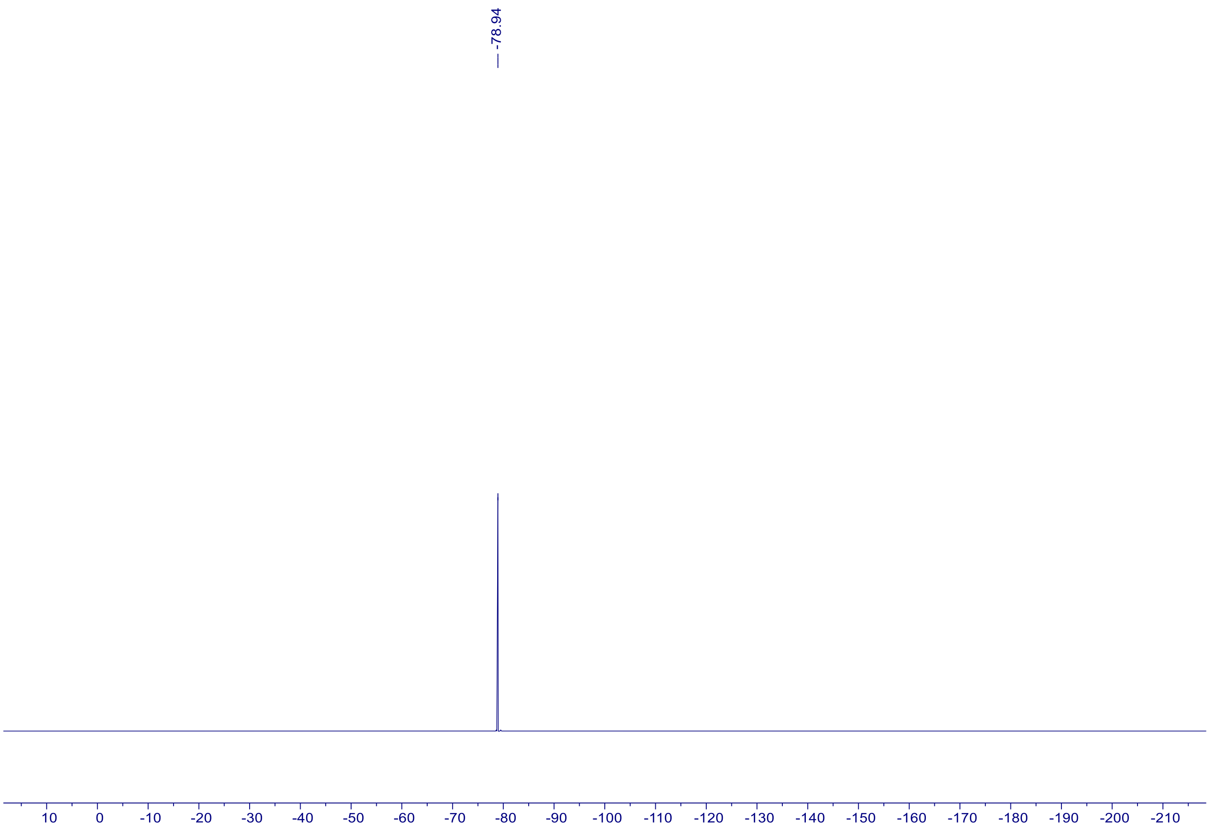

1f – ^{1}H NMR (500 MHz, acetone-d_6)

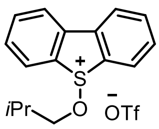

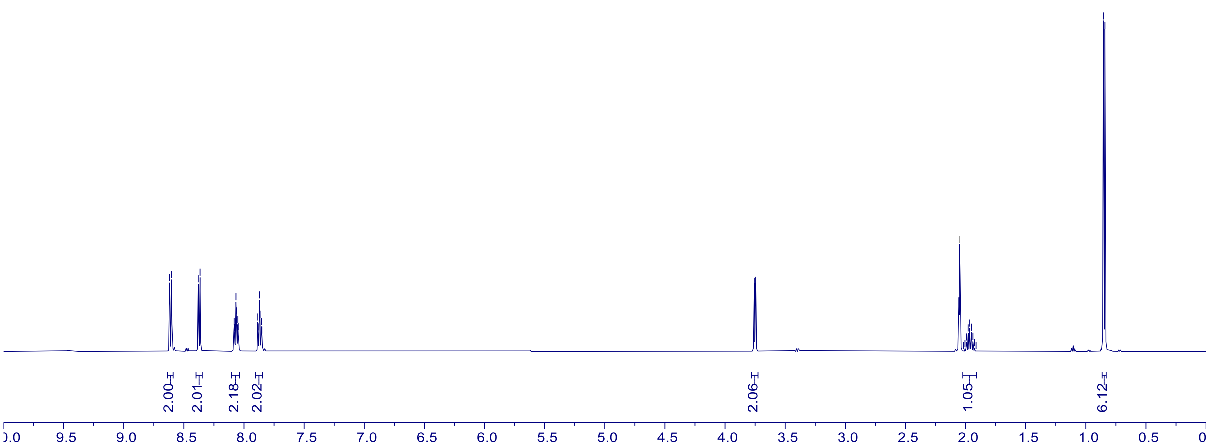

1f – ^{13}C NMR (126 MHz, acetone-d_6)

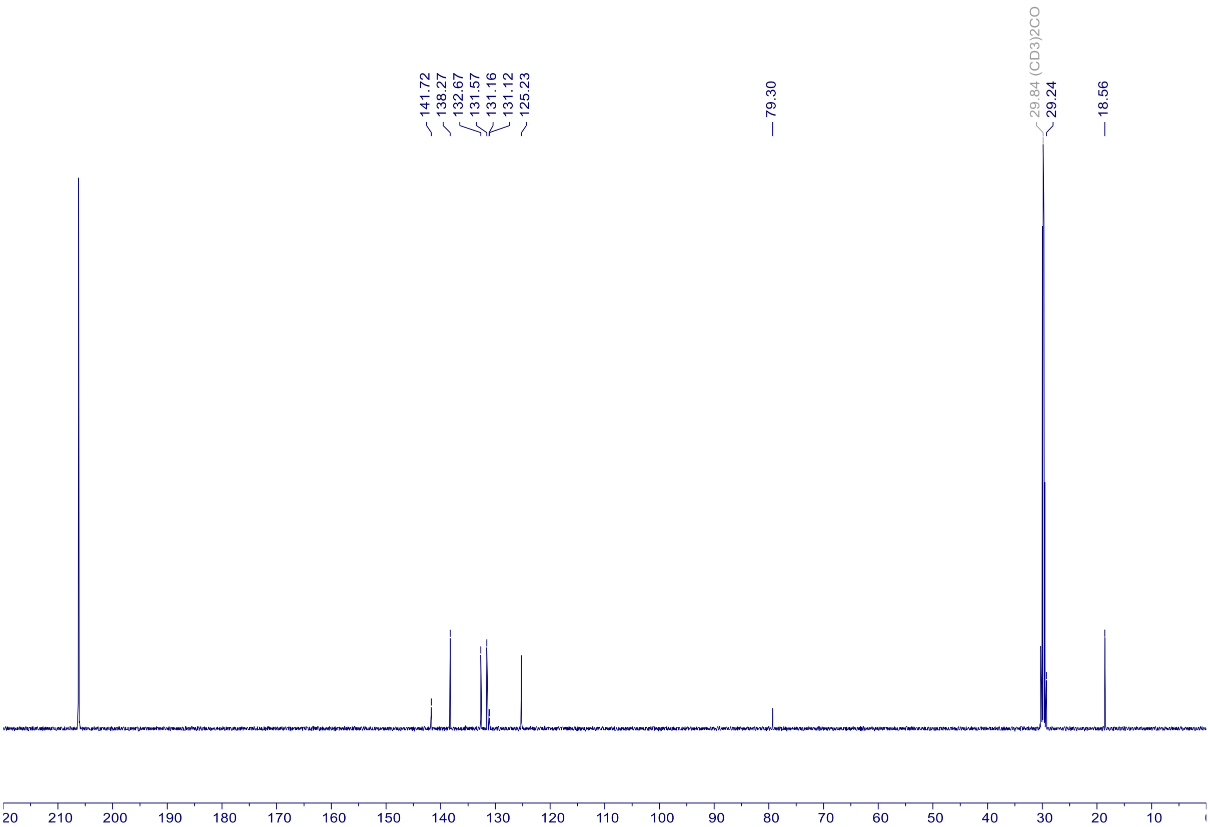

1f – ^{19}F NMR (471 MHz, acetone-d_6)

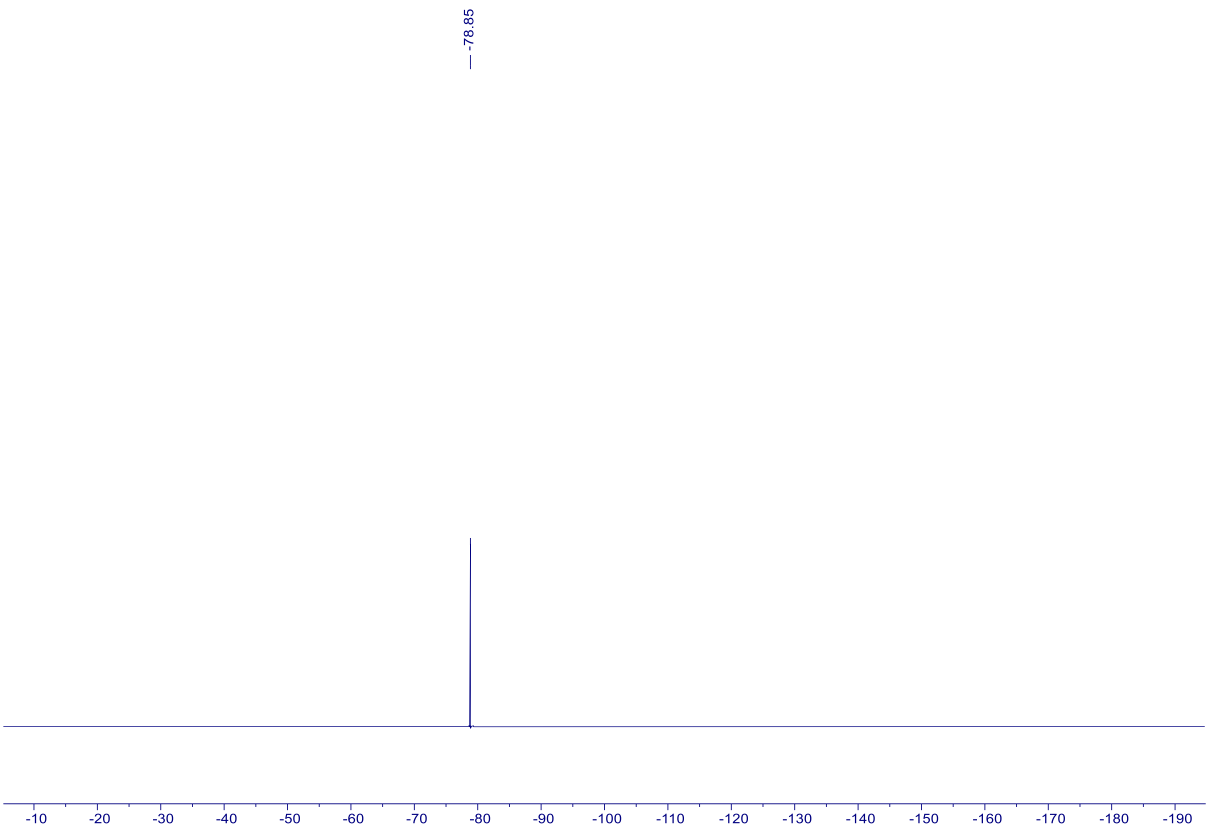

1g – ^{1}H NMR (400 MHz, acetone-d_6)

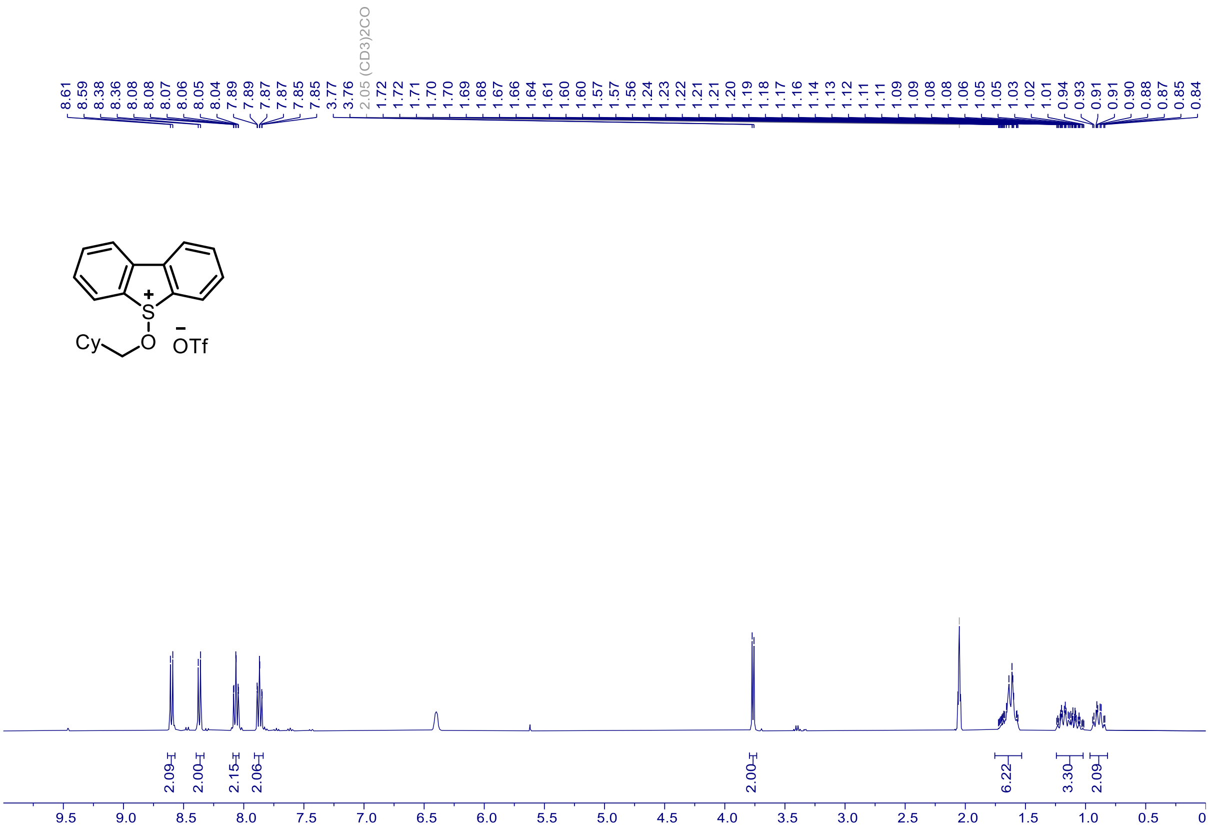

1g – ^{13}C NMR (101 MHz, acetone-d_6)

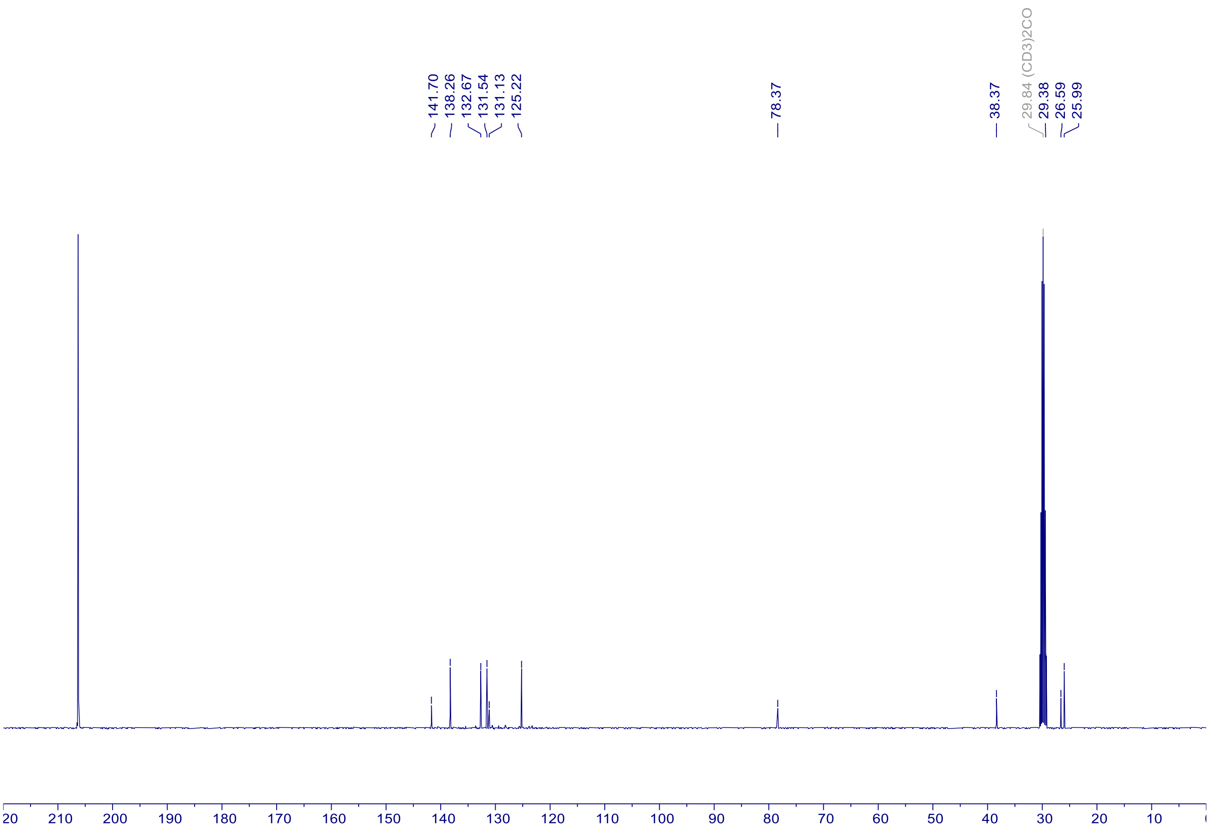

1g – ^{19}F NMR (376 MHz, acetone-d_6)

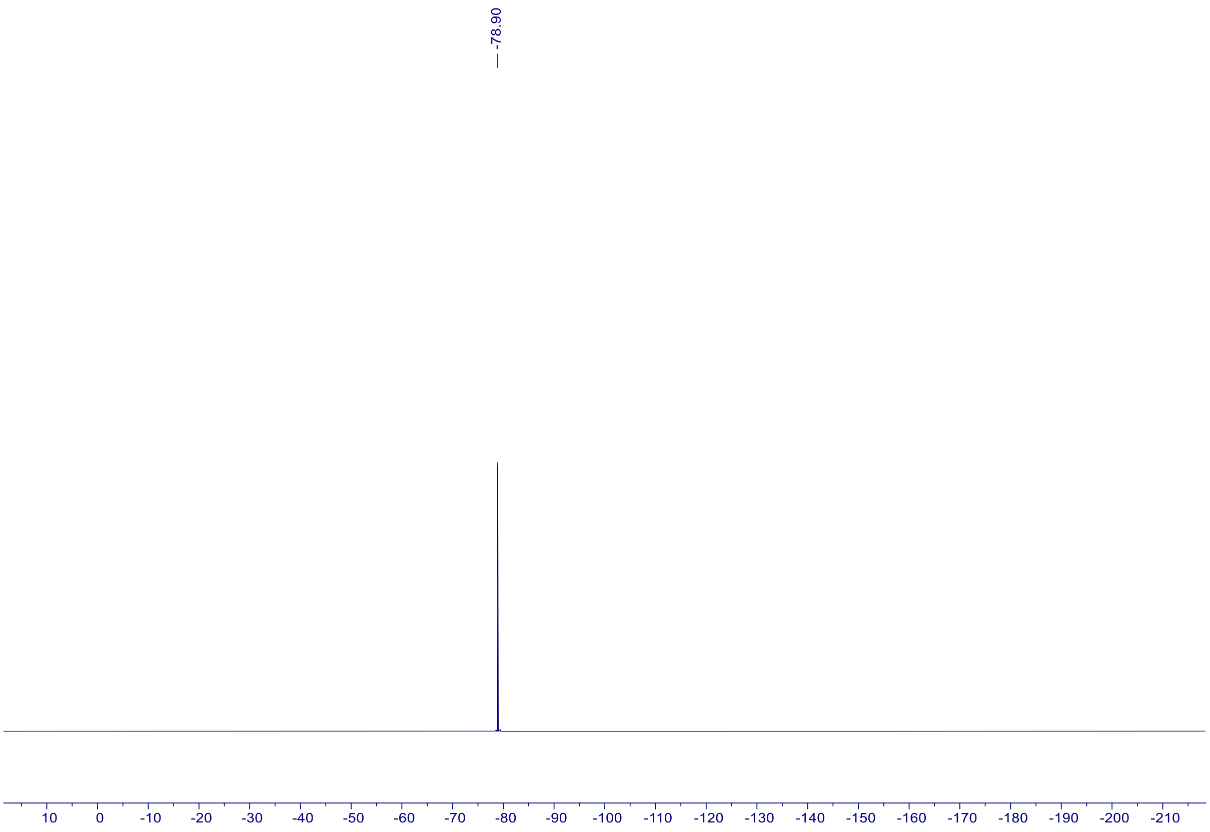

1h – ^{1}H NMR (400 MHz, acetone-d_6)

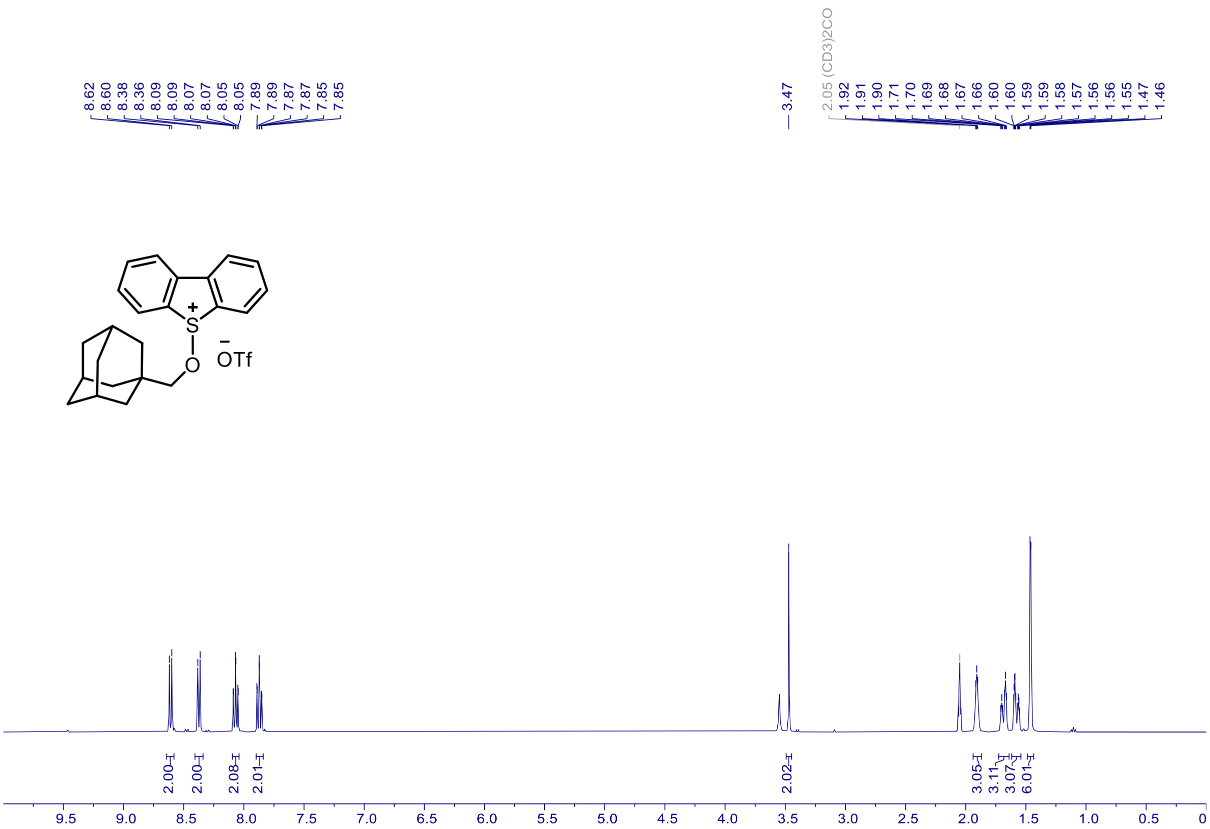

1h – ^{13}C NMR (101 MHz, acetone-d_6)

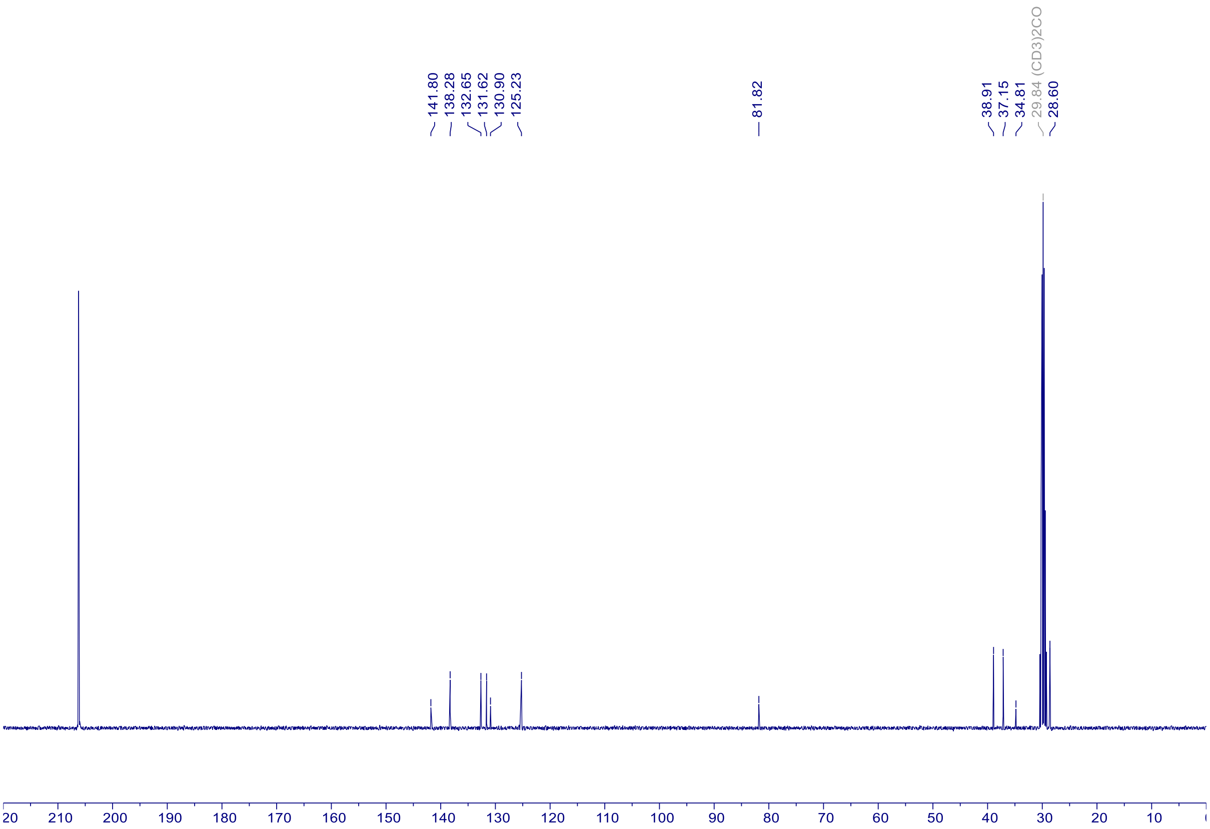

1h – ^{19}F NMR (376 MHz, acetone-d_6)

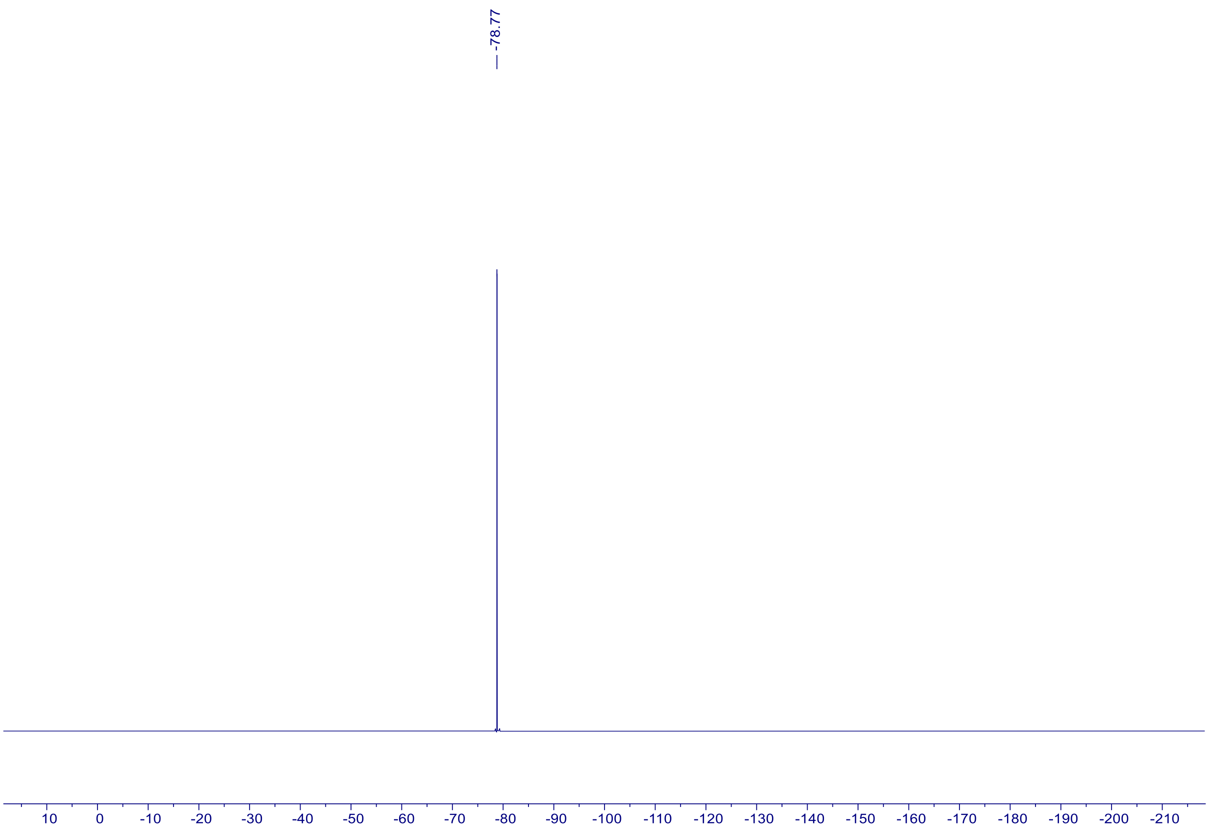

1i – ^{1}H NMR (400 MHz, acetone-d_6)

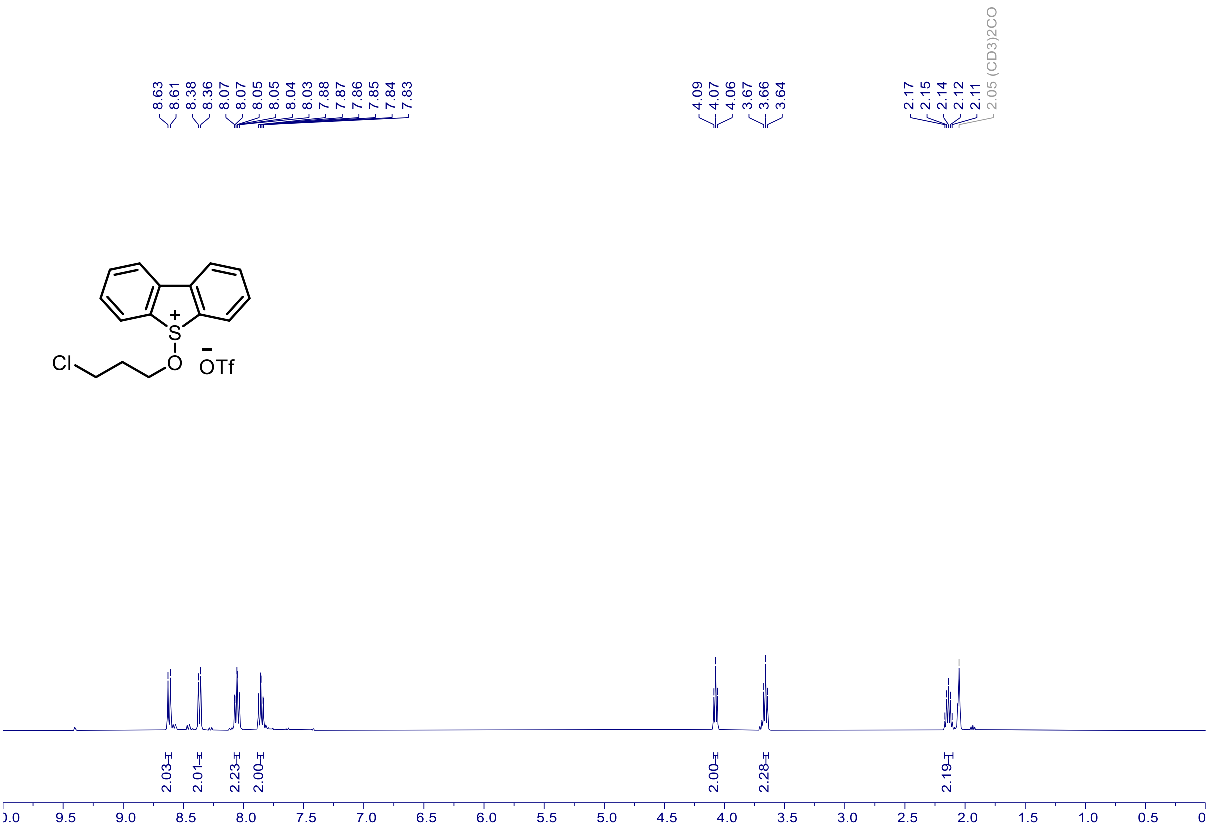

1i – ^{13}C NMR (101 MHz, acetone-d_6)

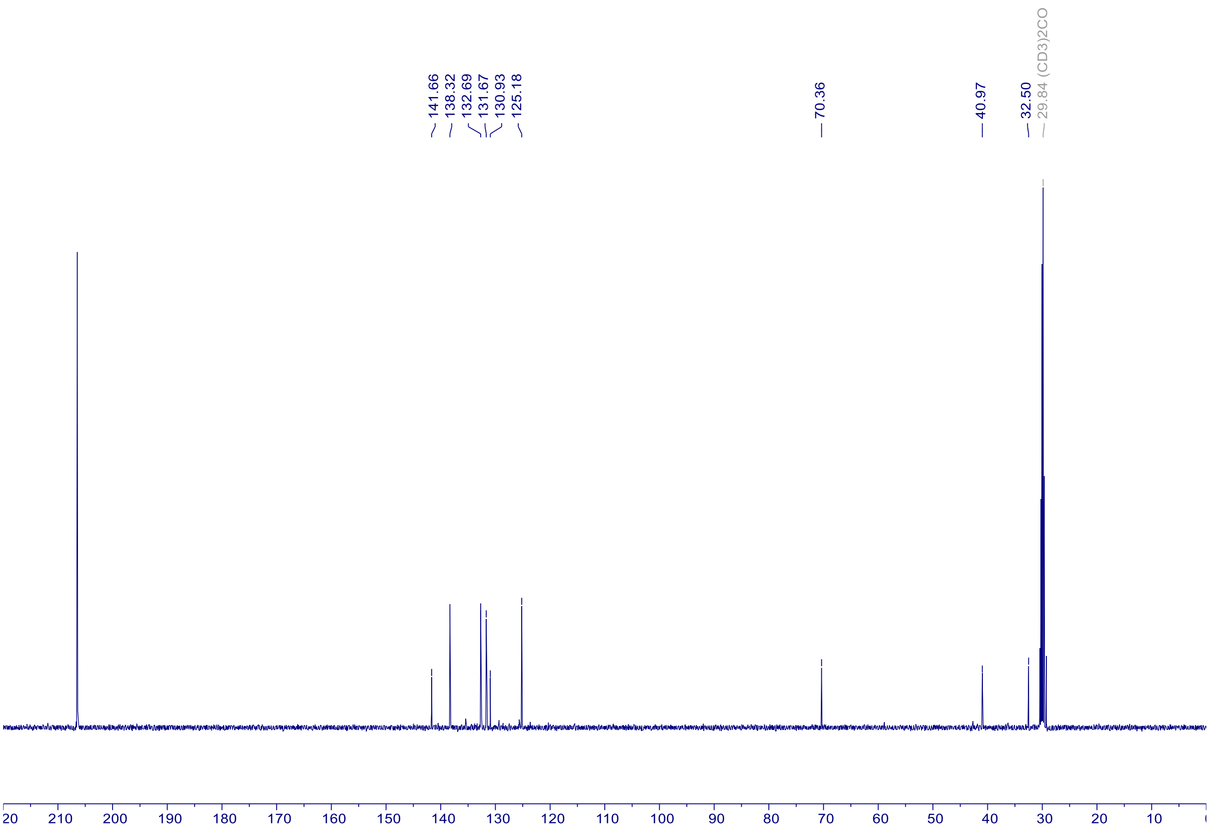

1i – ^{19}F NMR (376 MHz, acetone-d_6)

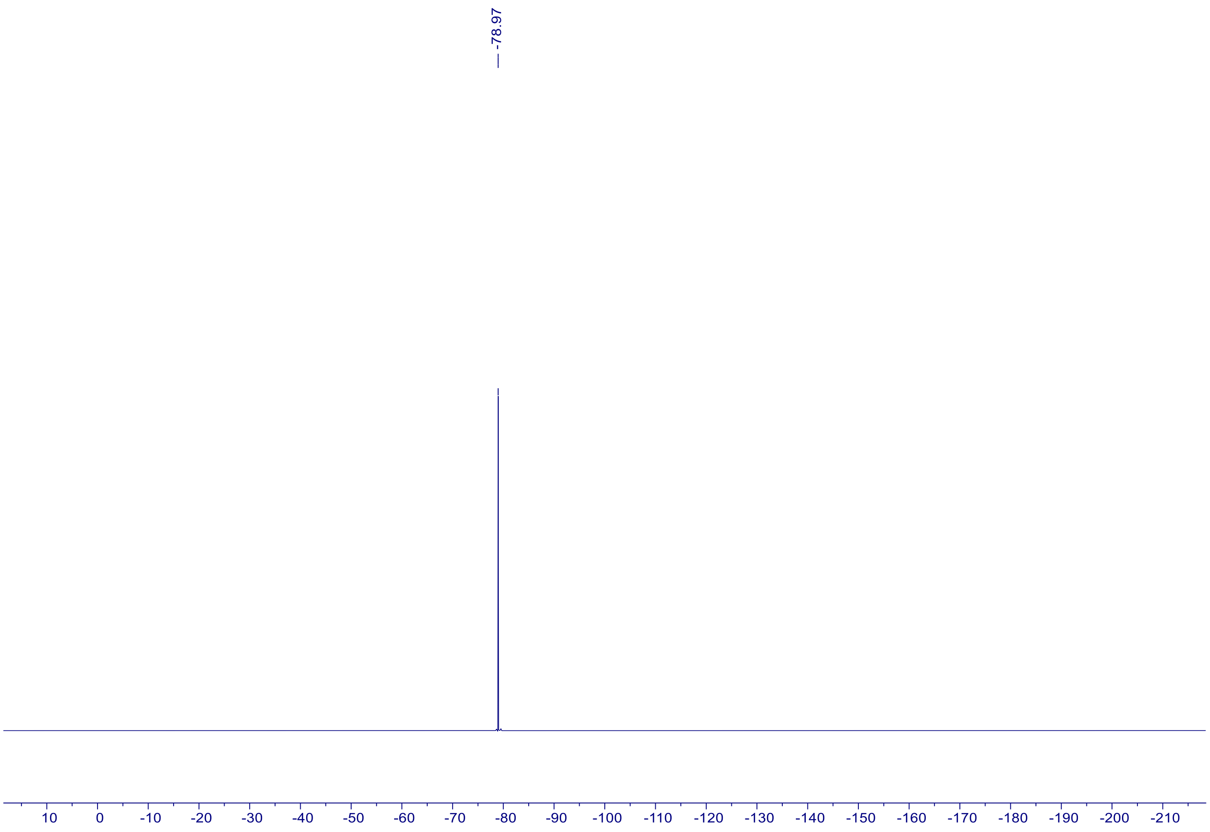

1j – ^{1}H NMR (500 MHz, acetone-d_6)

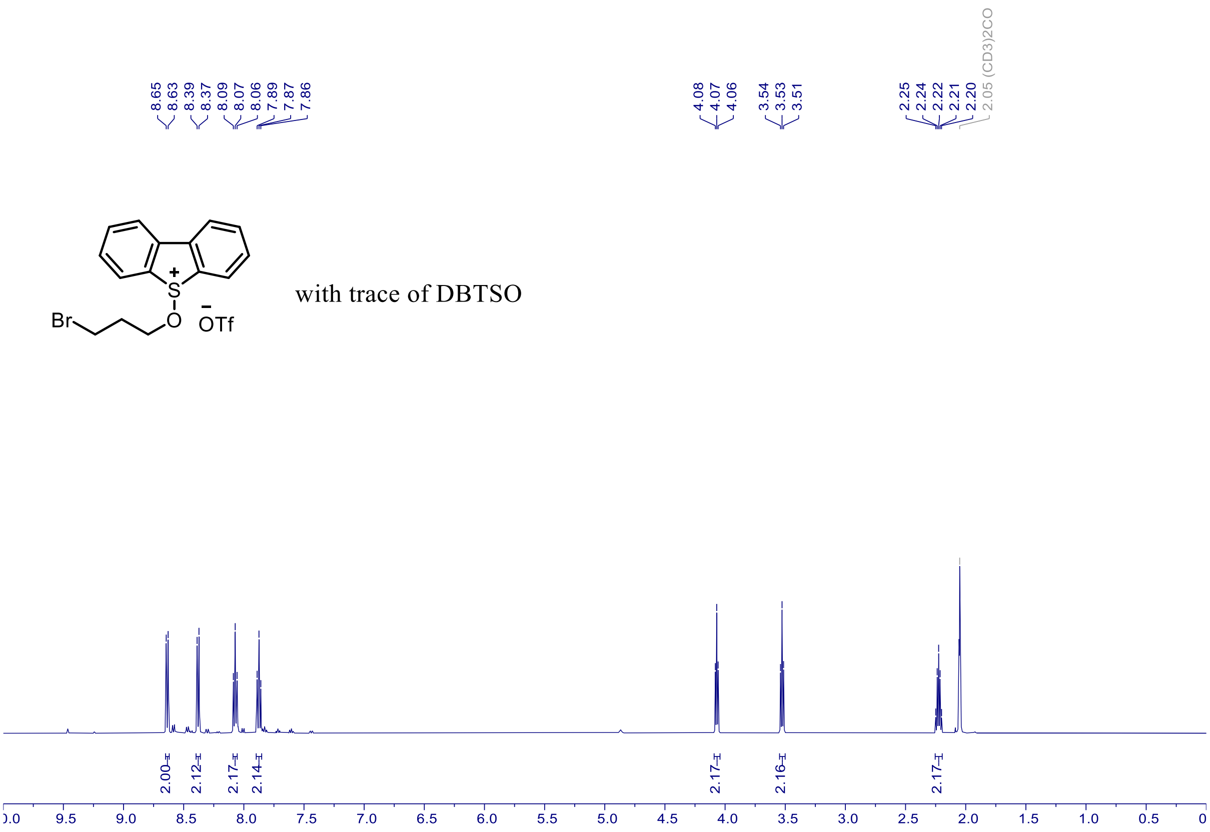

1j – ^{13}C NMR (126 MHz, acetone-d_6)

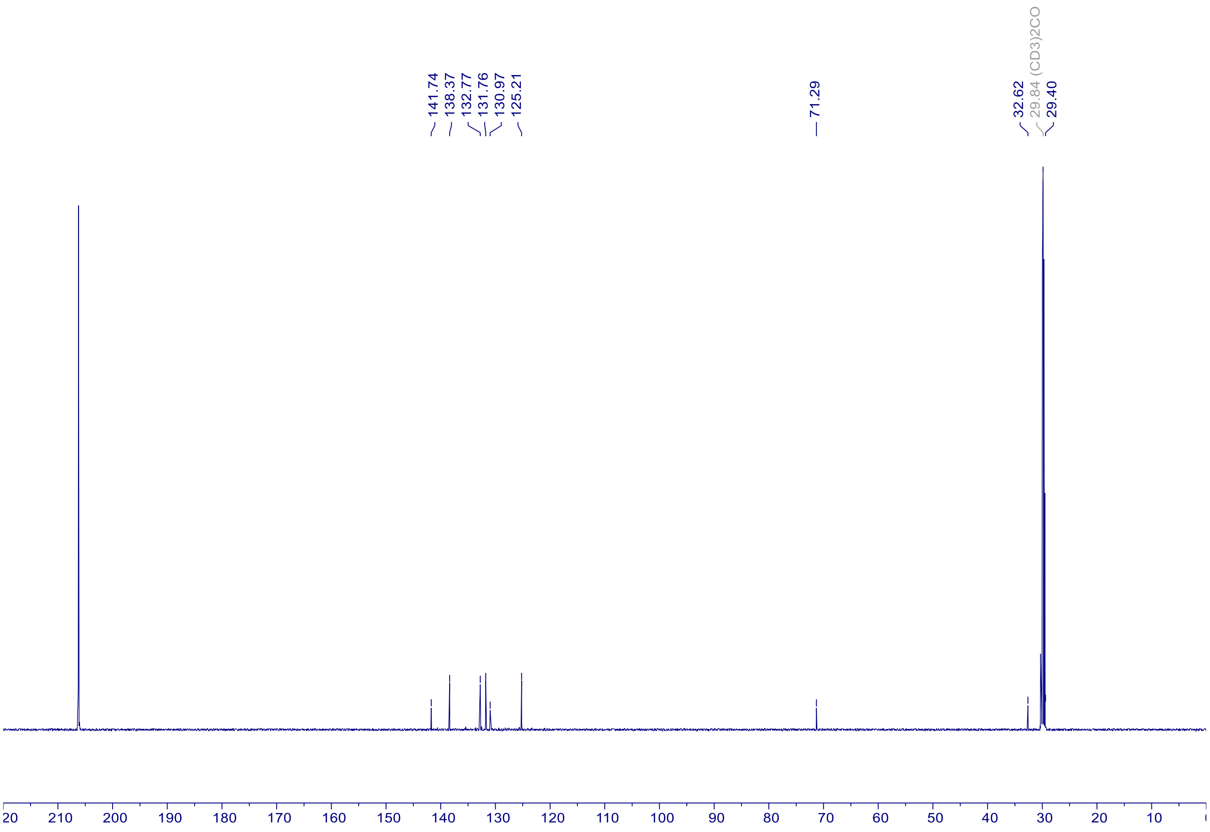

1j – ^{19}F NMR (471 MHz, acetone-d_6)

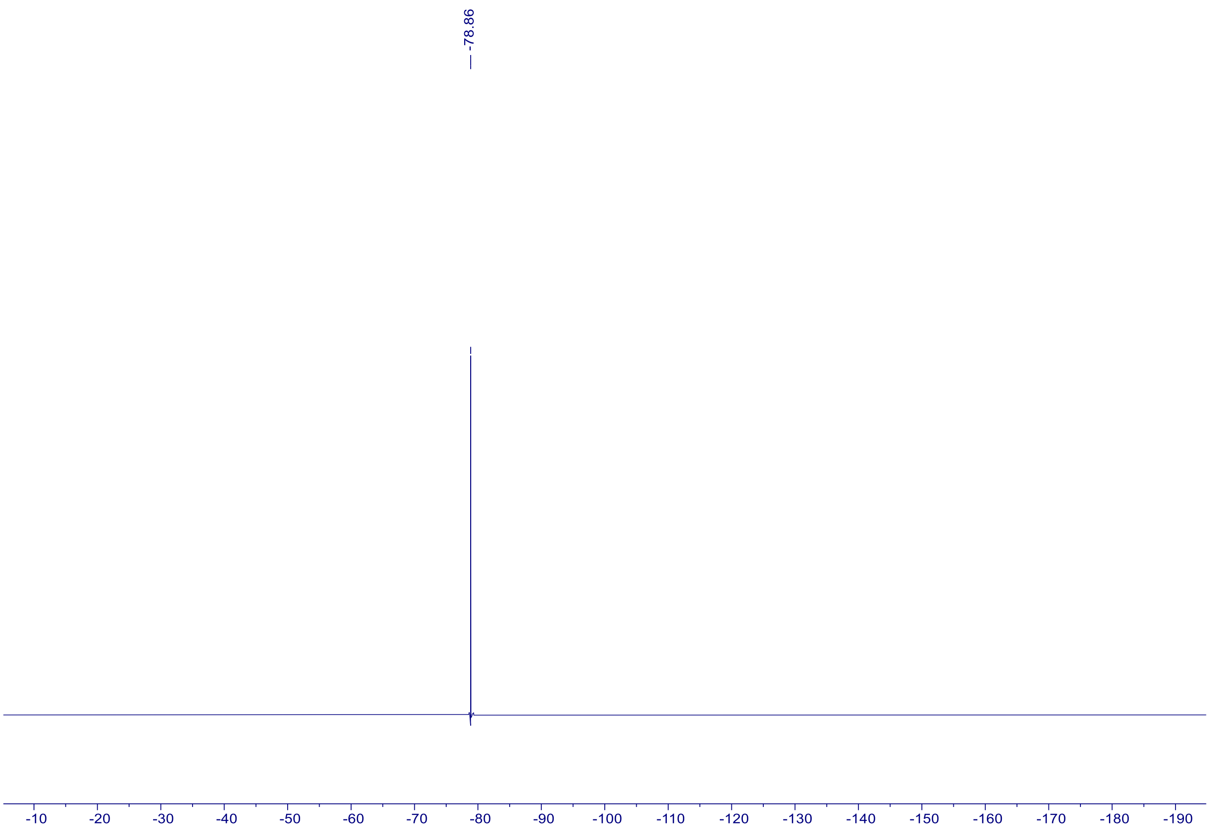

1k – ^{1}H NMR (400 MHz, acetone-d_6)

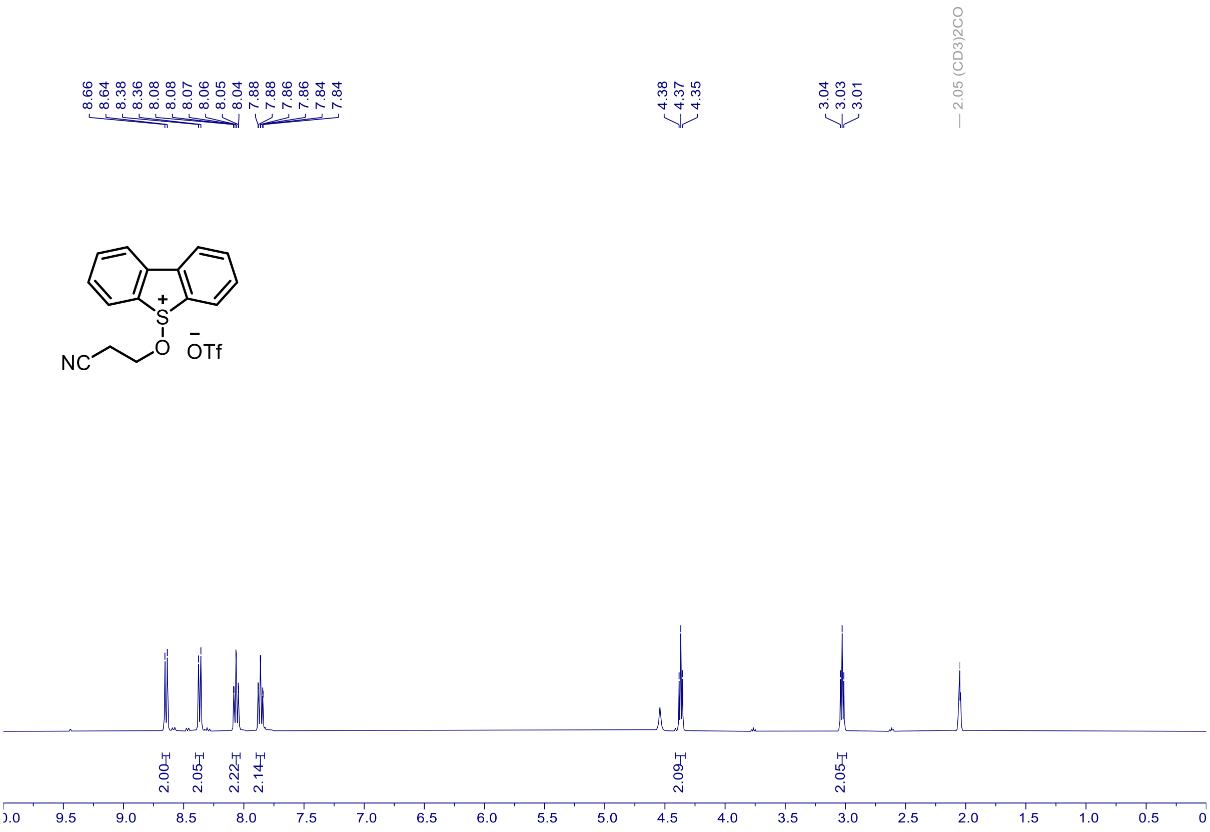

1k – ^{13}C NMR (101 MHz, acetone-d_6)

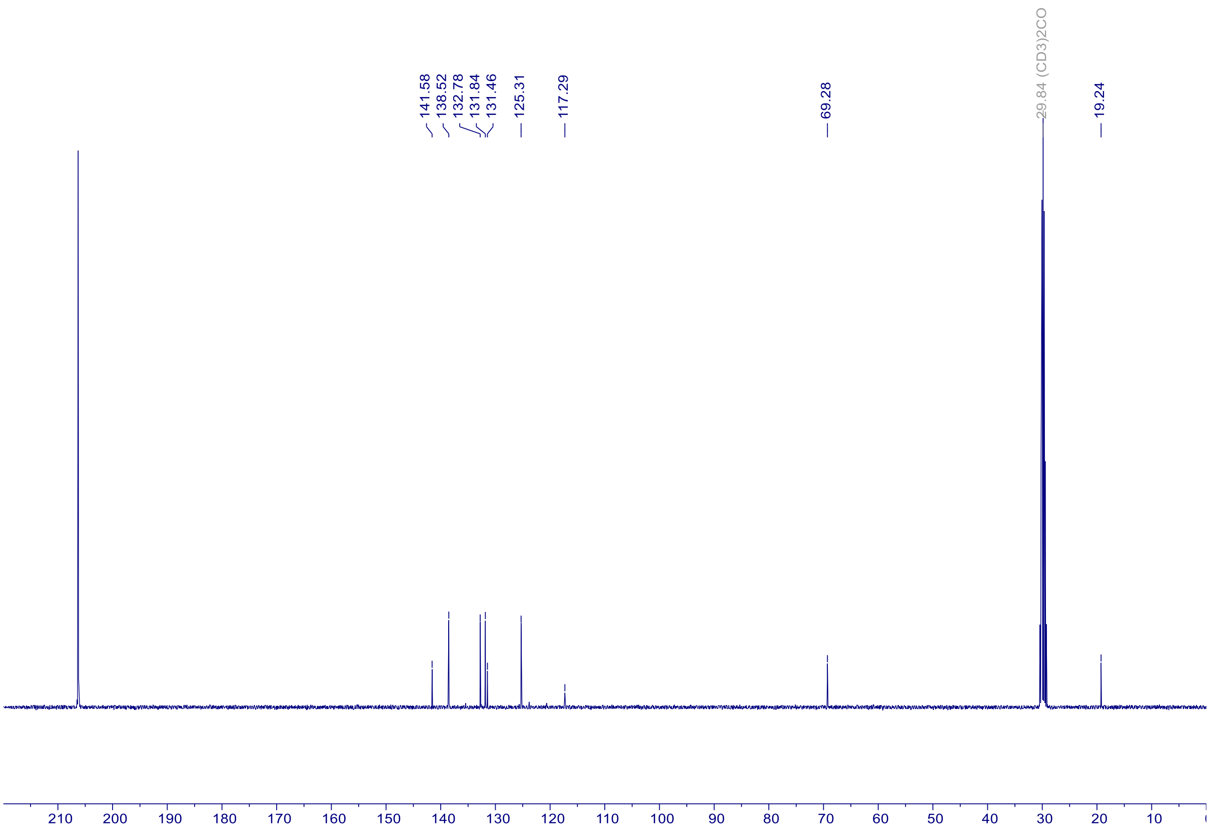

1k – ^{19}F NMR (376 MHz, acetone-d_6)

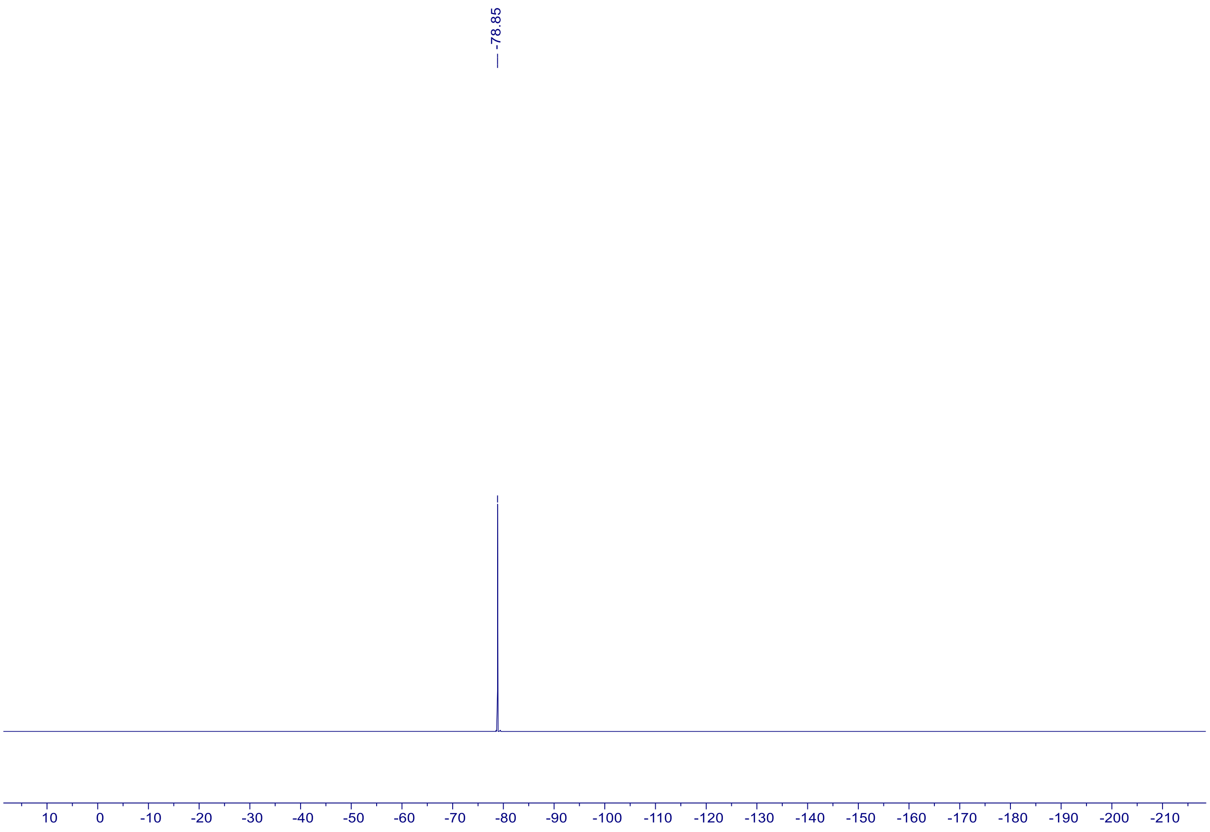

1l – ^{1}H NMR (400 MHz, acetone-d_6)

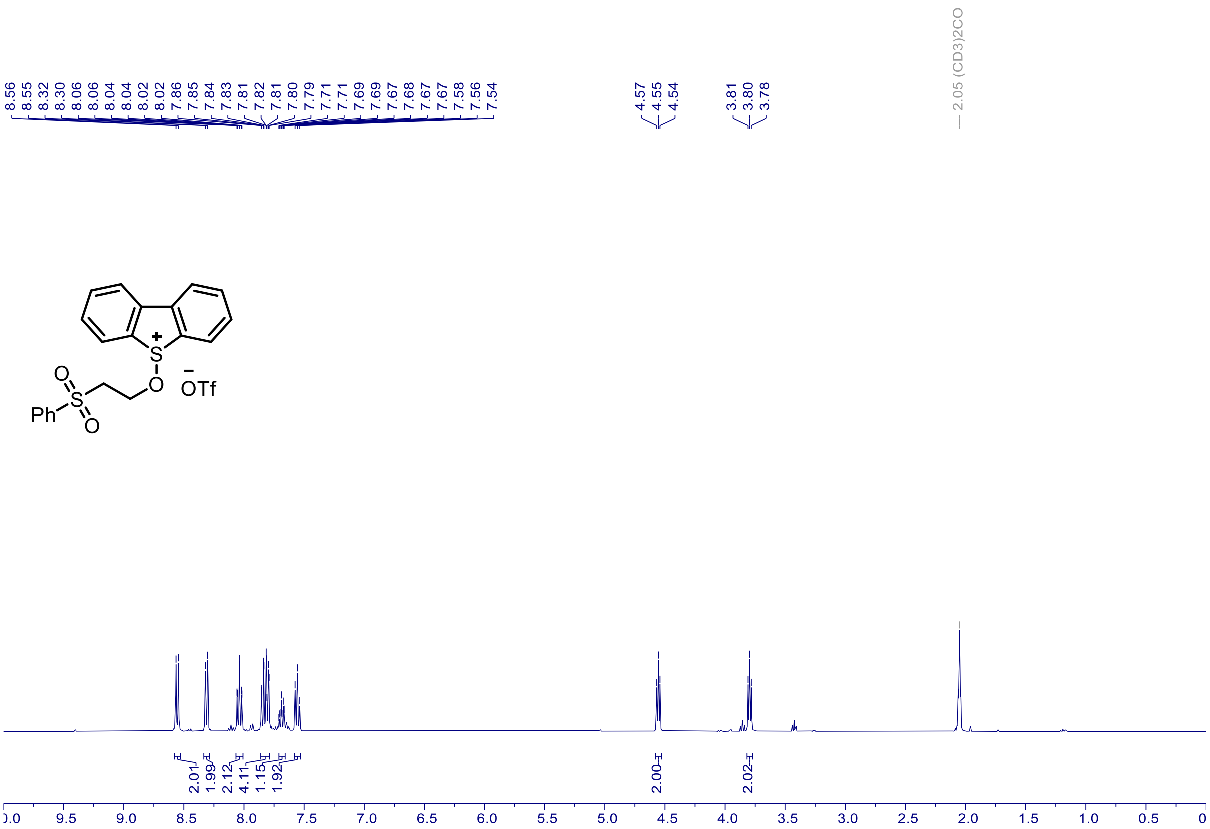

1l – ^{13}C NMR (101 MHz, acetone-d_6)

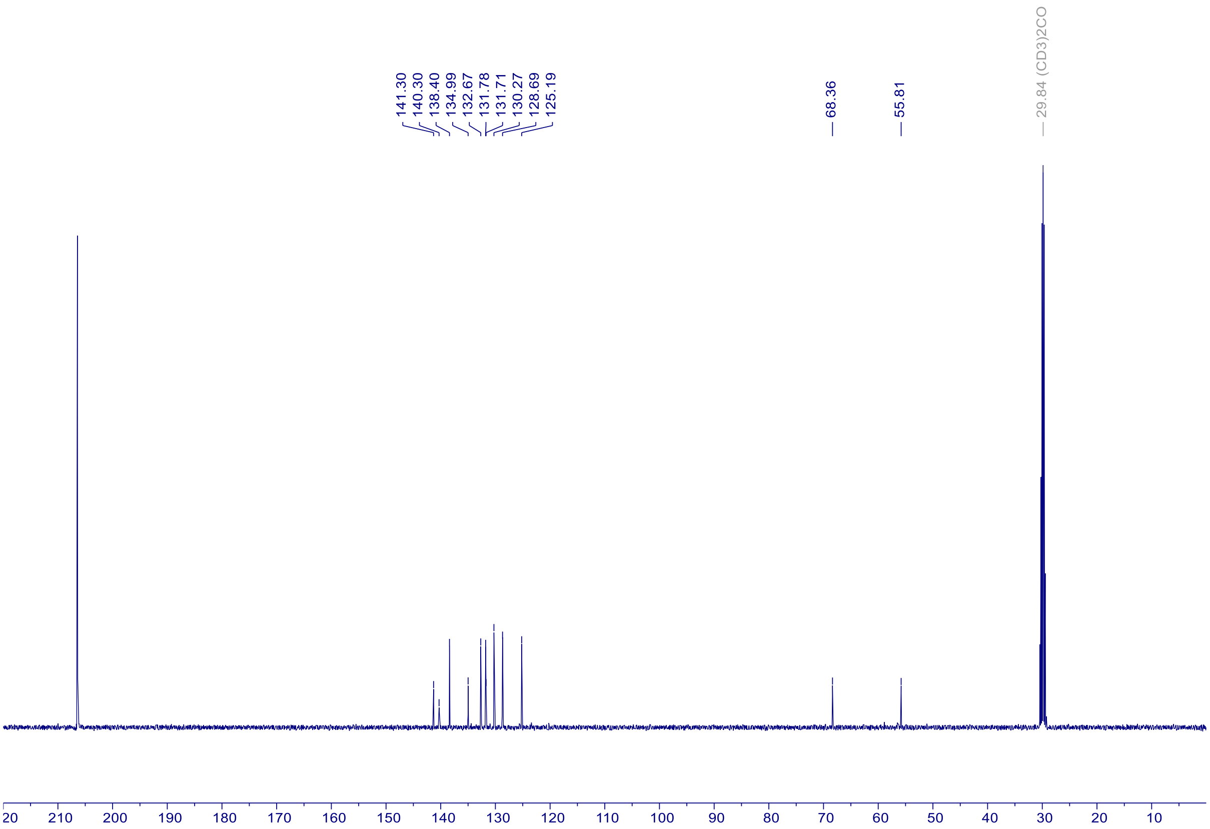

1l – ^{19}F NMR (376 MHz, acetone-d_6)

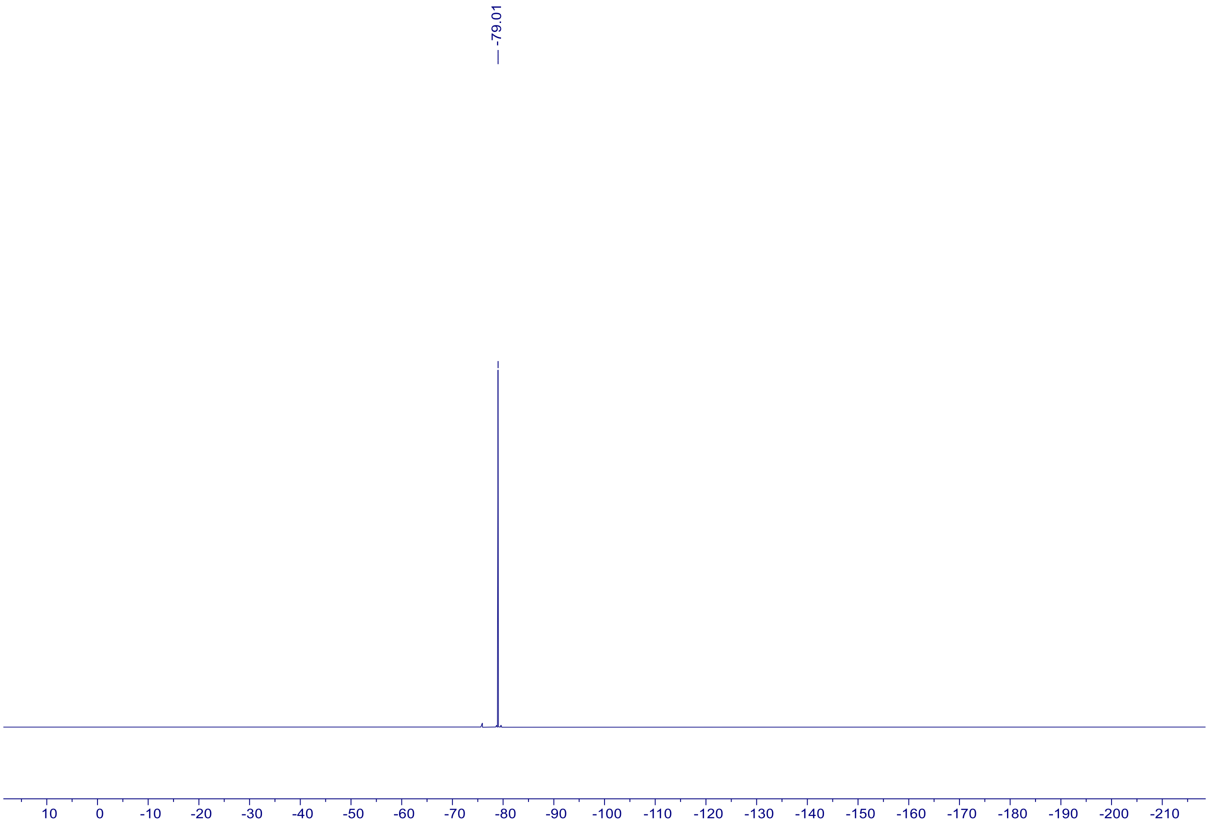

1m – ^{1}H NMR (400 MHz, acetone-d_6)

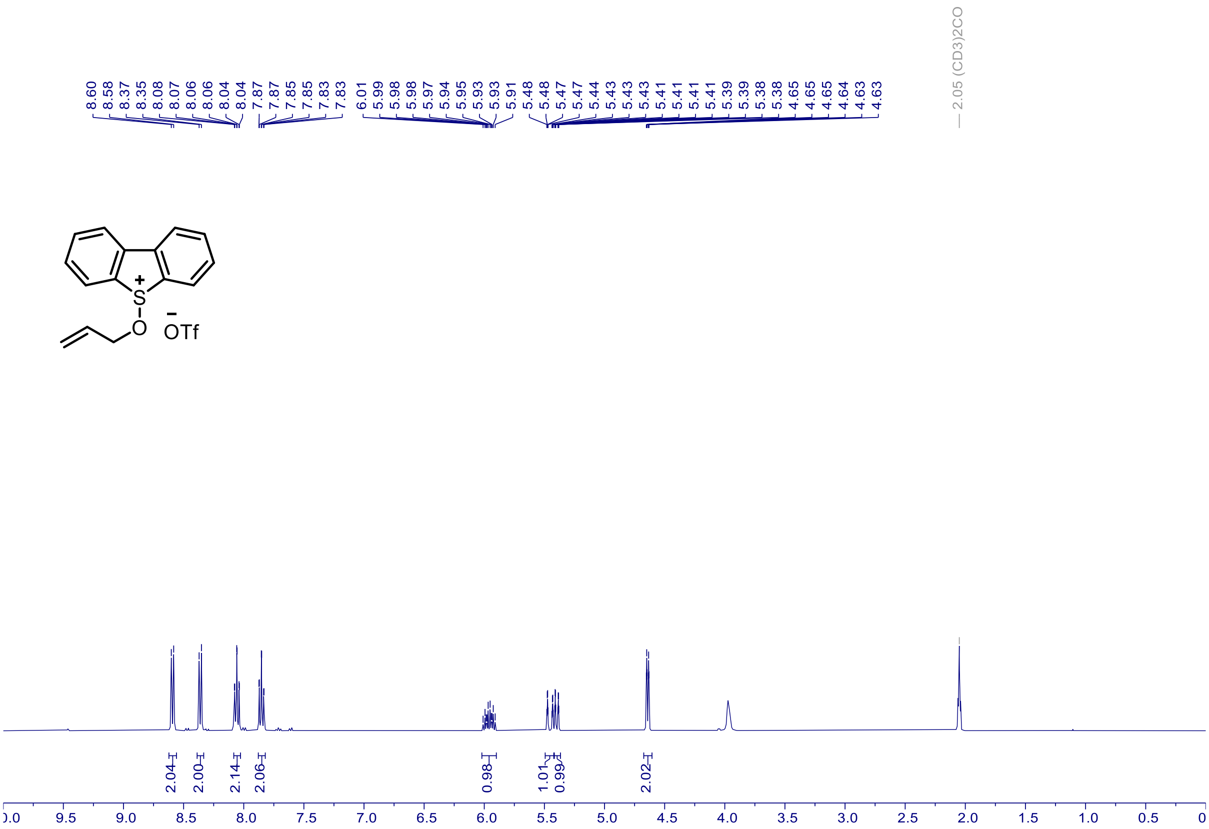

1m – ^{13}C NMR (101 MHz, acetone-d_6)

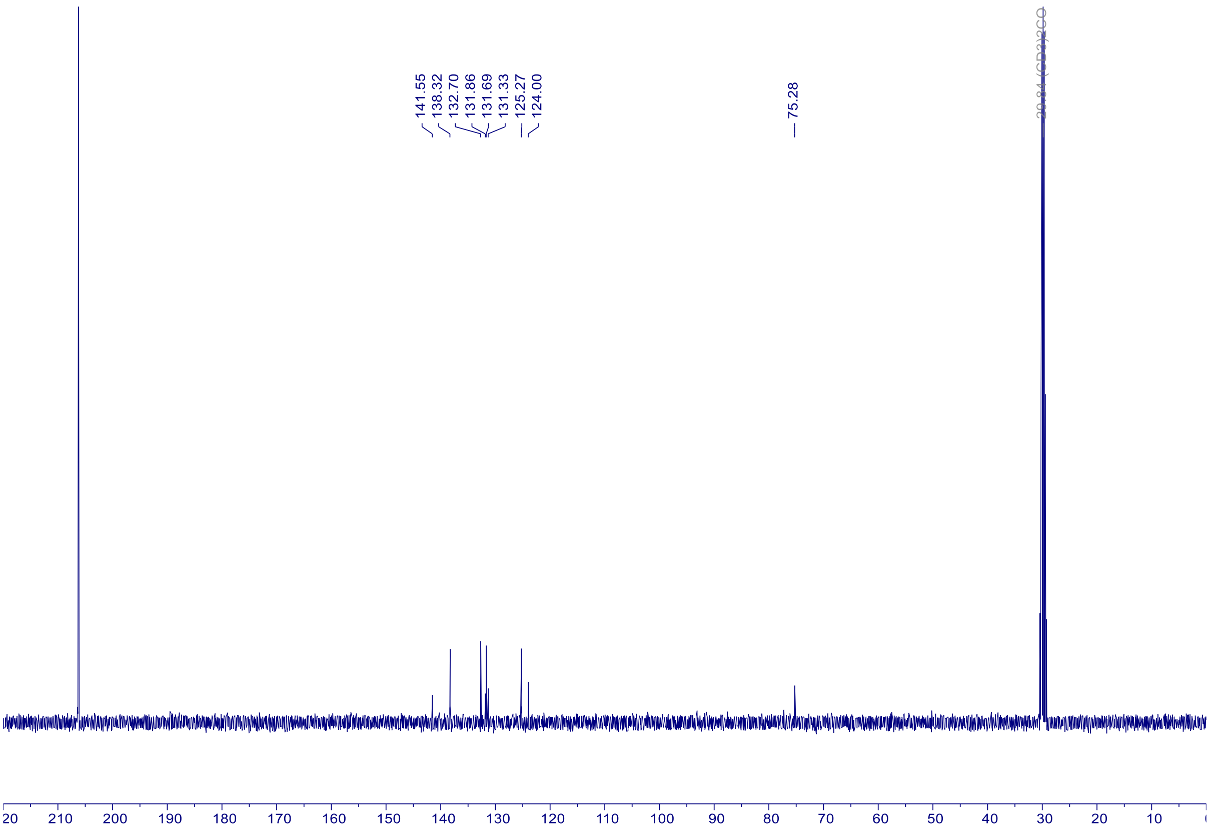

1m – ^{19}F NMR (376 MHz, acetone-d_6)

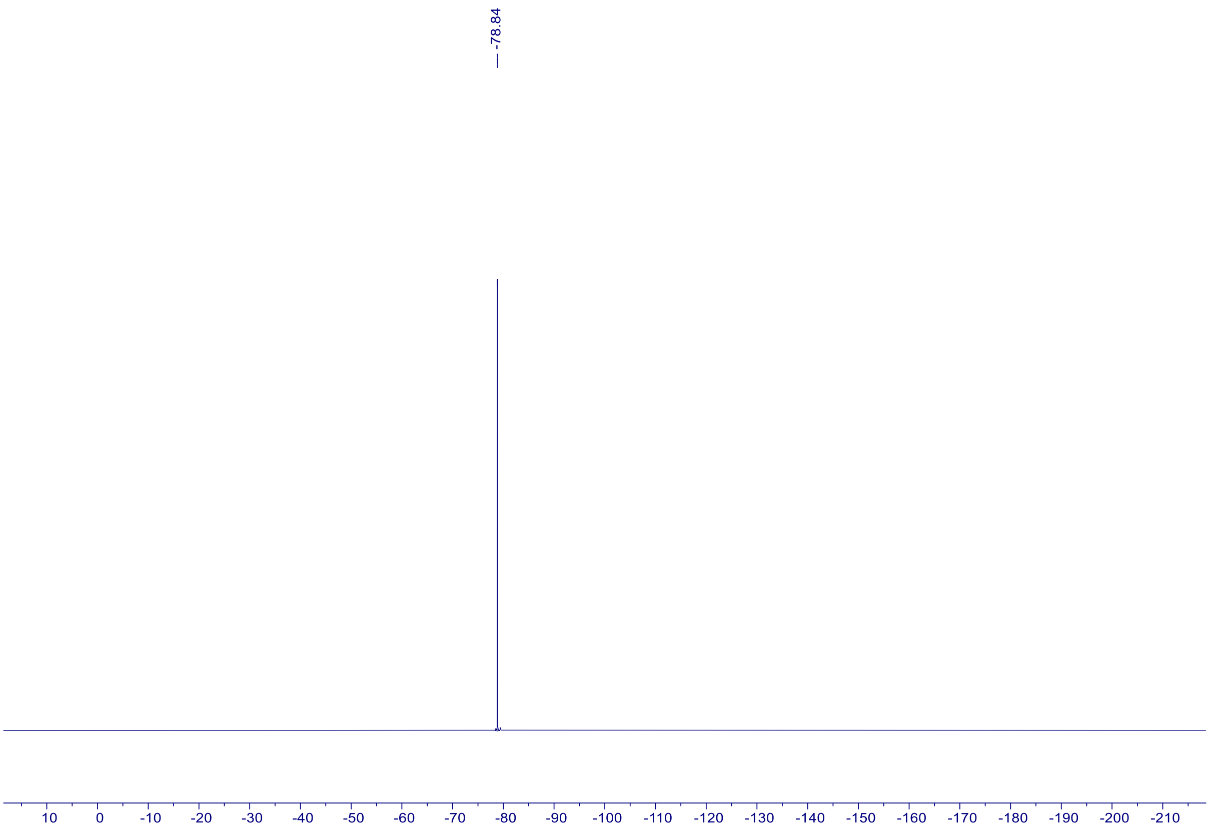

1n – ^{1}H NMR (500 MHz, acetone-d_6)

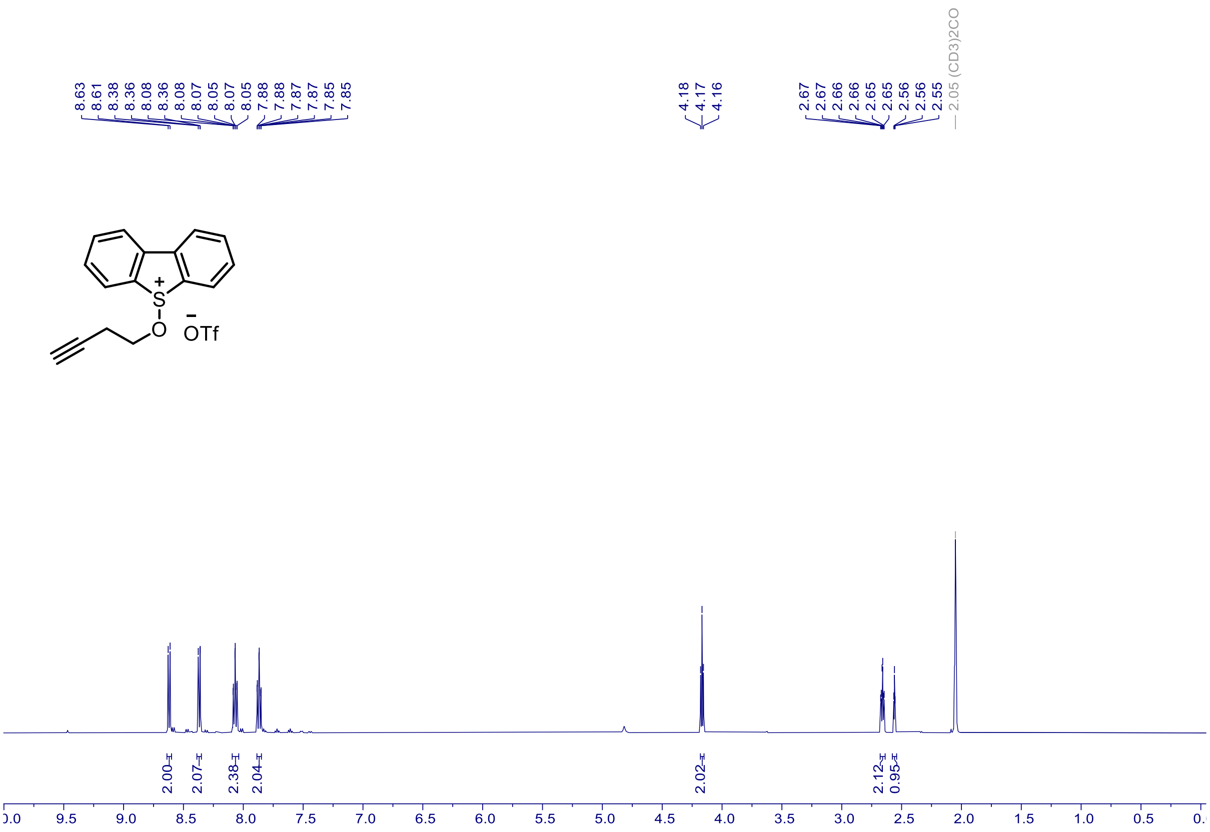

1n – ^{13}C NMR (126 MHz, acetone-d_6)

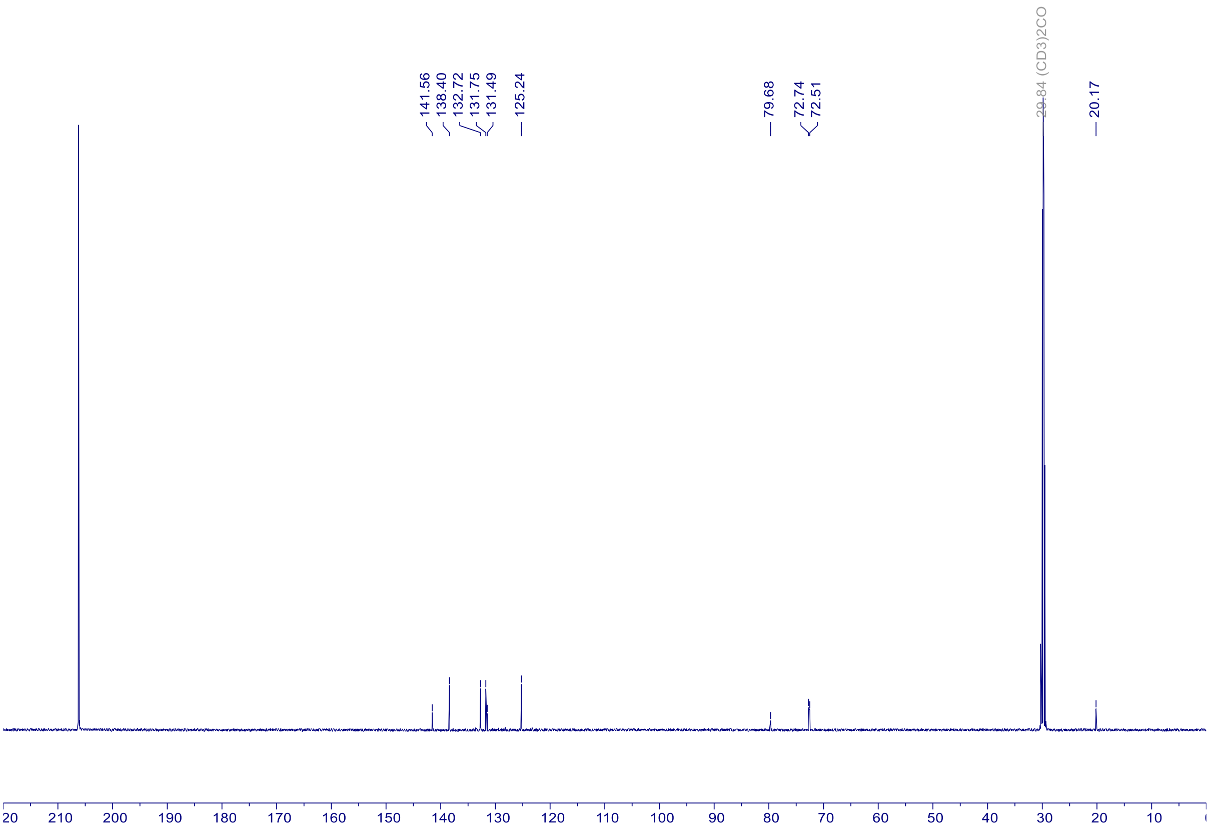

1n – ^{19}F NMR (471 MHz, acetone-d_6)

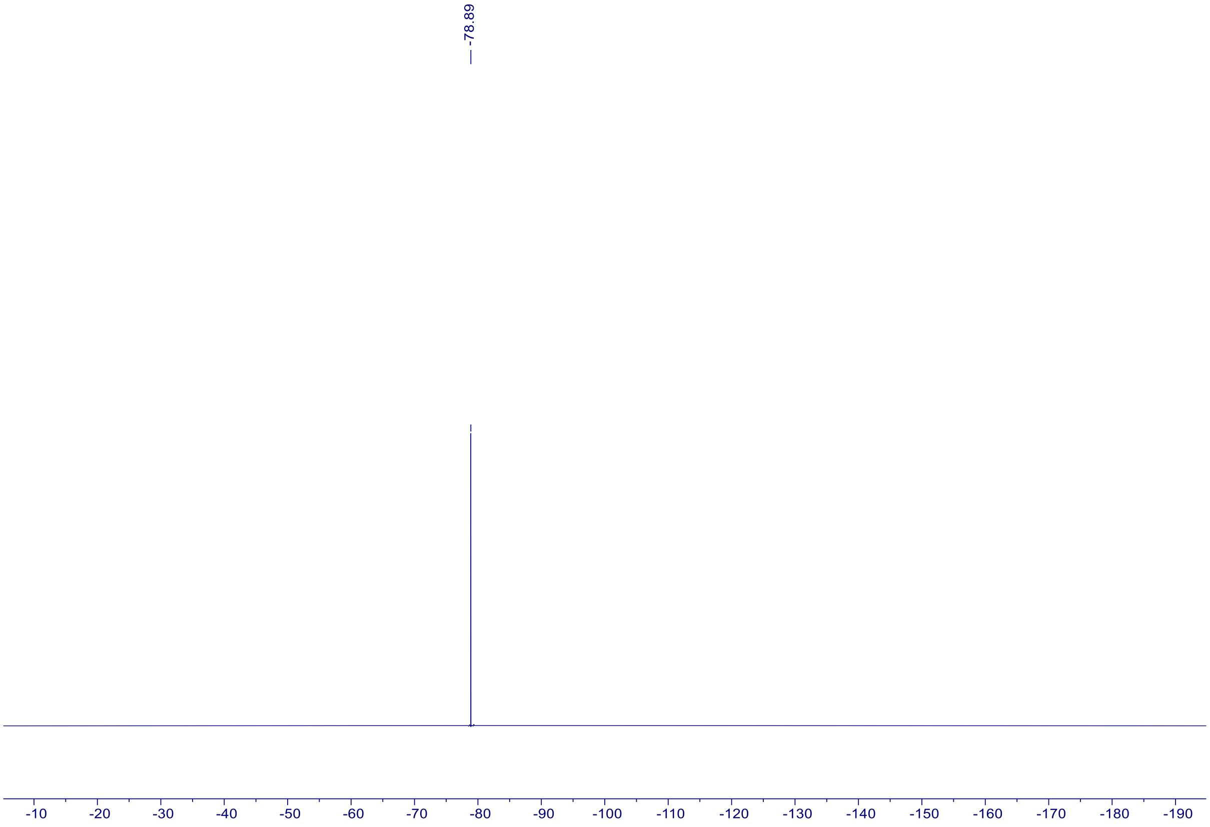

1o – ^{1}H NMR (400 MHz, acetone-d_6)

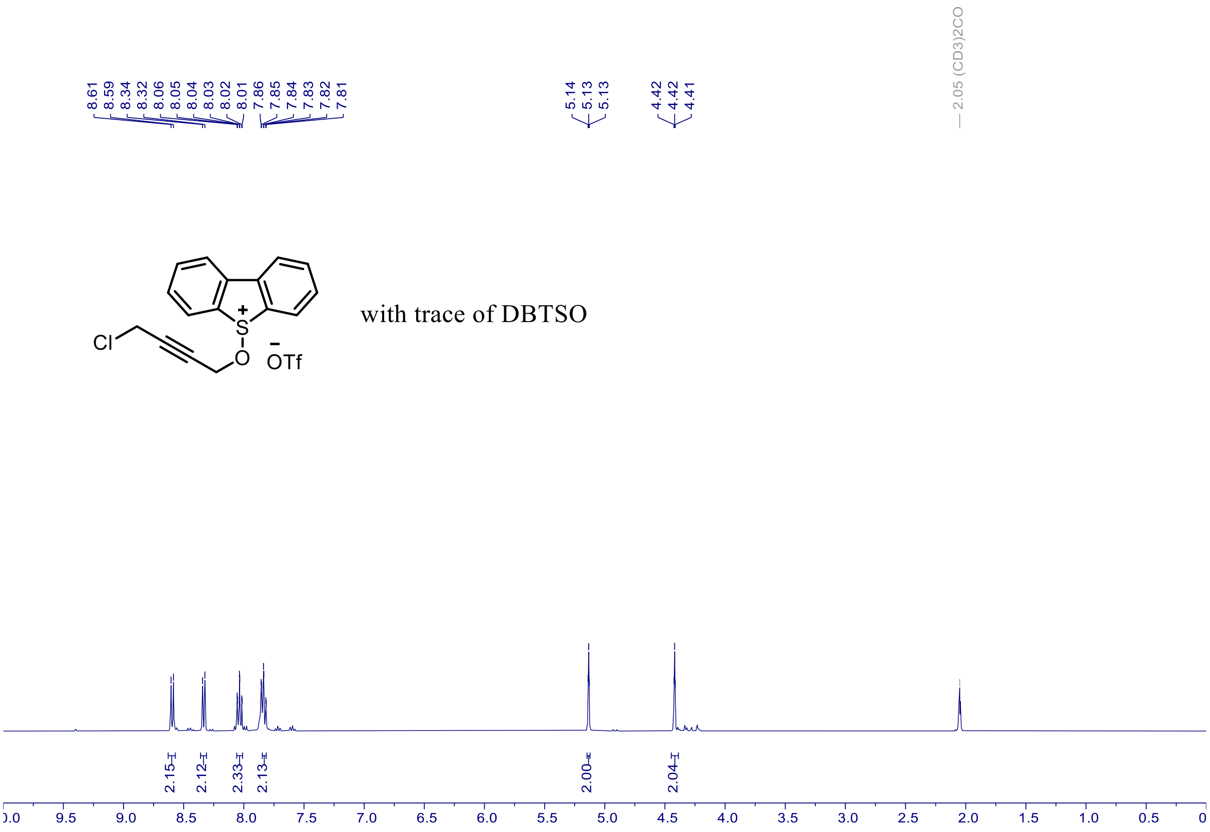

1o – ^{13}C NMR (101 MHz, acetone-d_6)

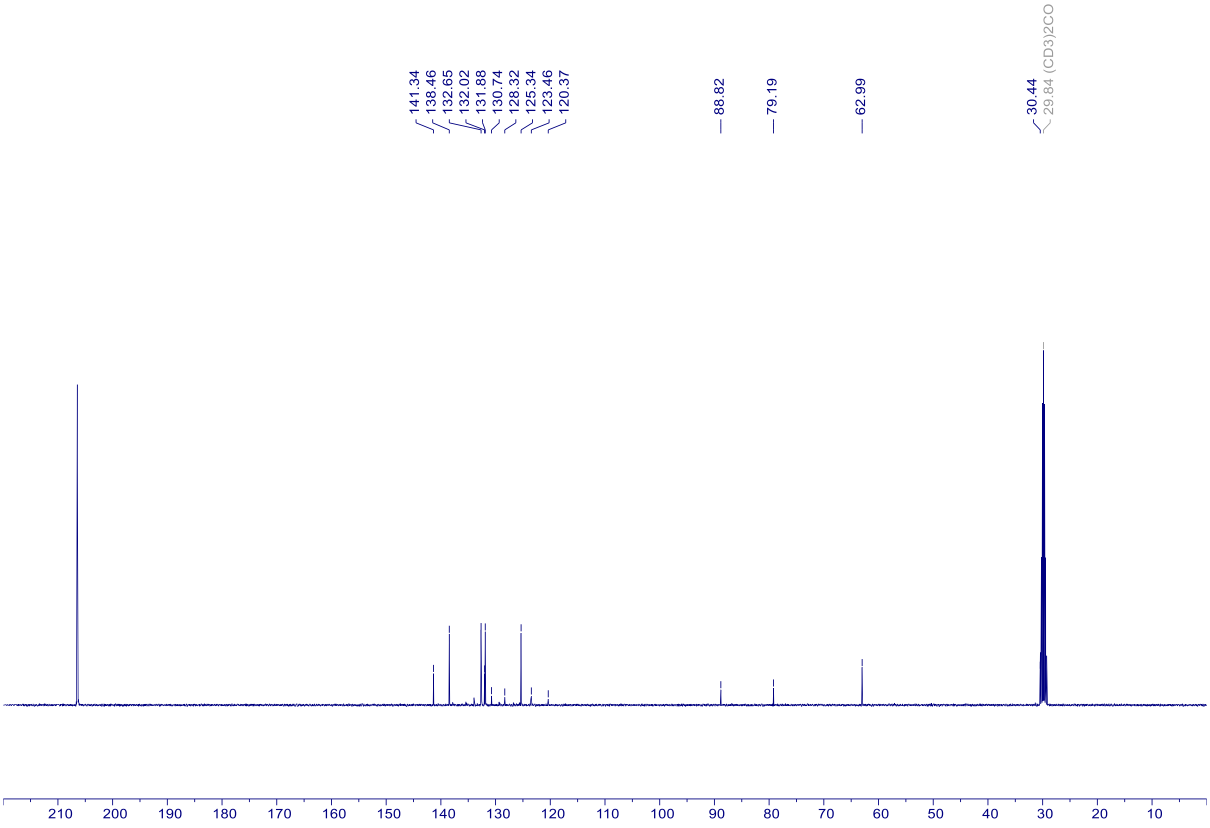

1o – ^{19}F NMR (376 MHz, acetone-d_6)

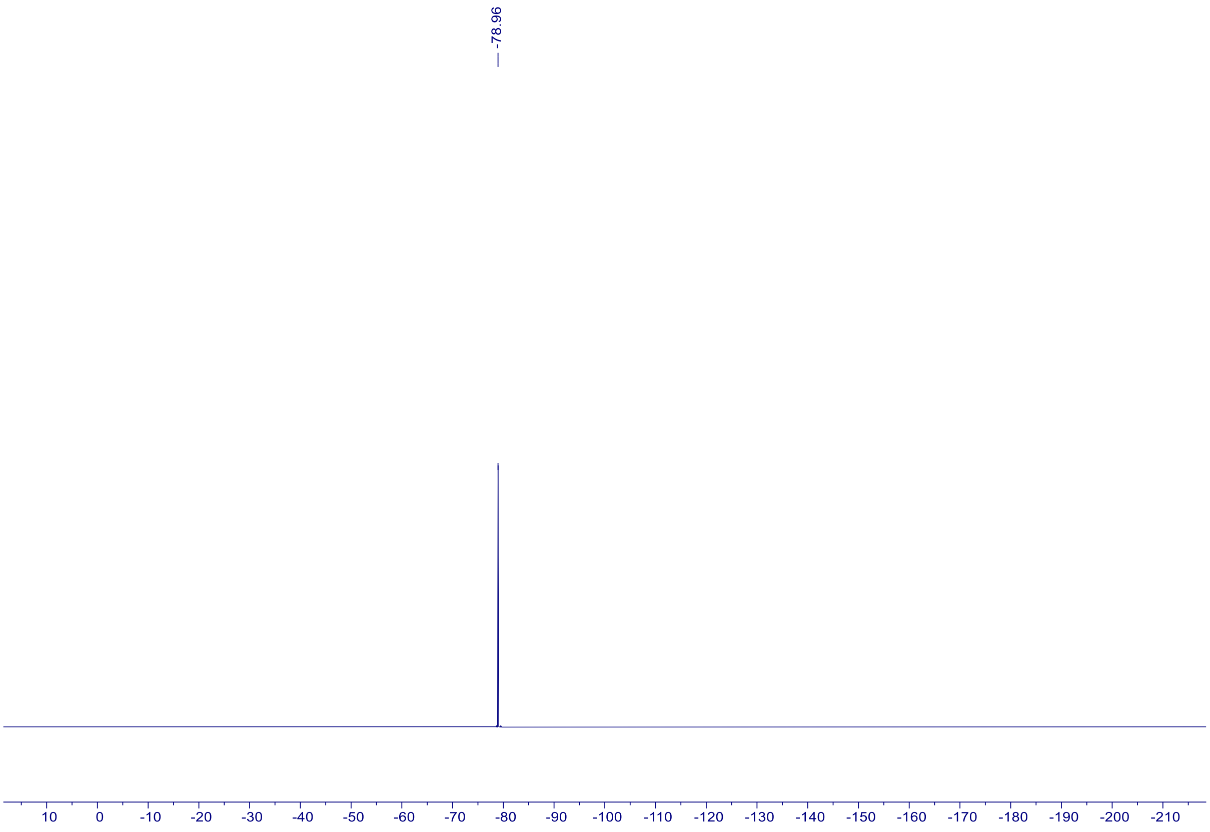

1p – ^{1}H NMR (400 MHz, acetone-d_6)

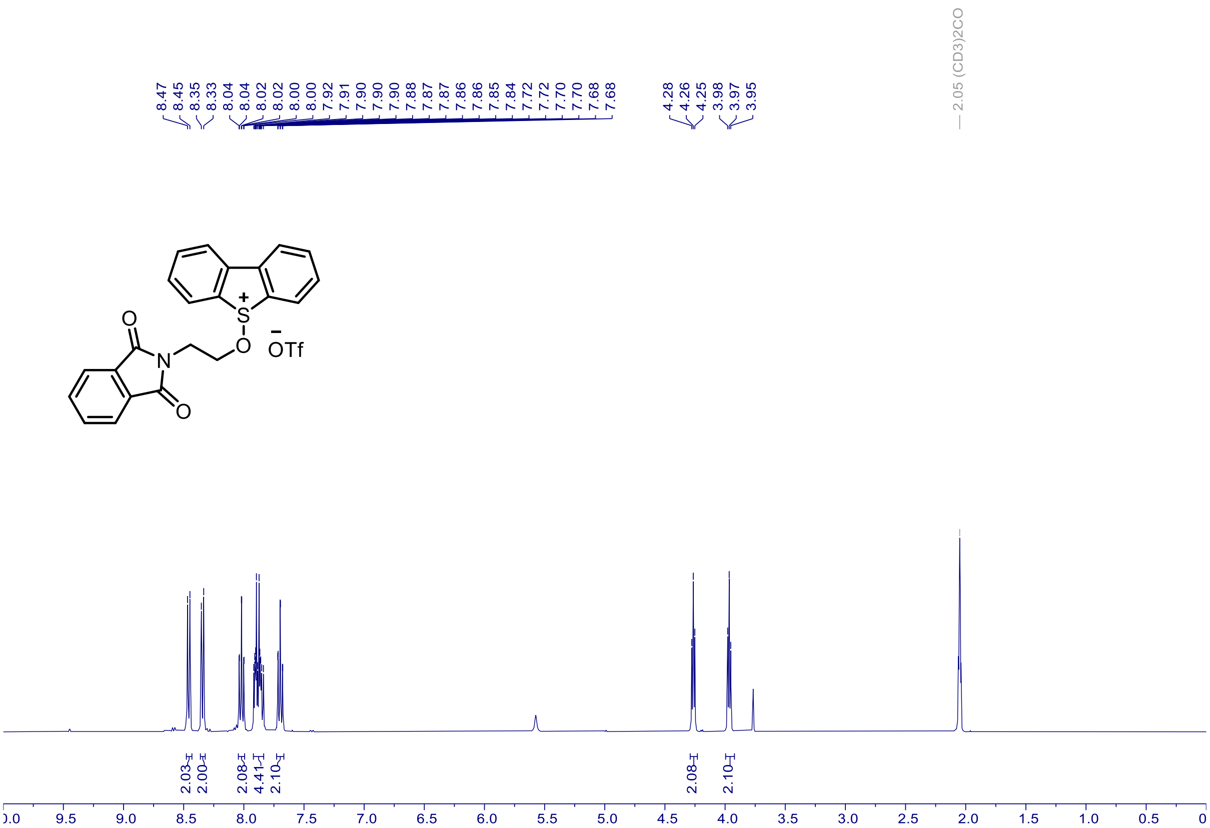

1p – ^{13}C NMR (101 MHz, acetone-d_6)

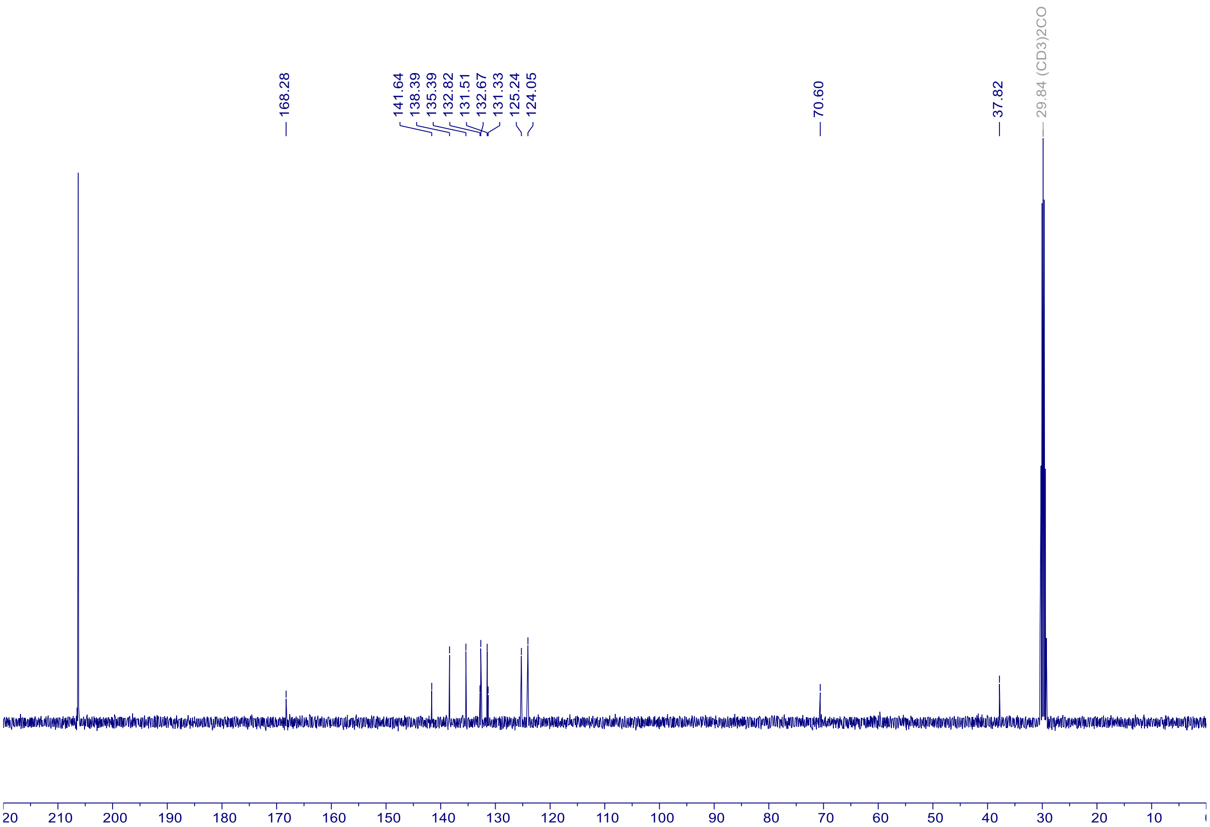

1p – ^{19}F NMR (376 MHz, acetone-d_6)

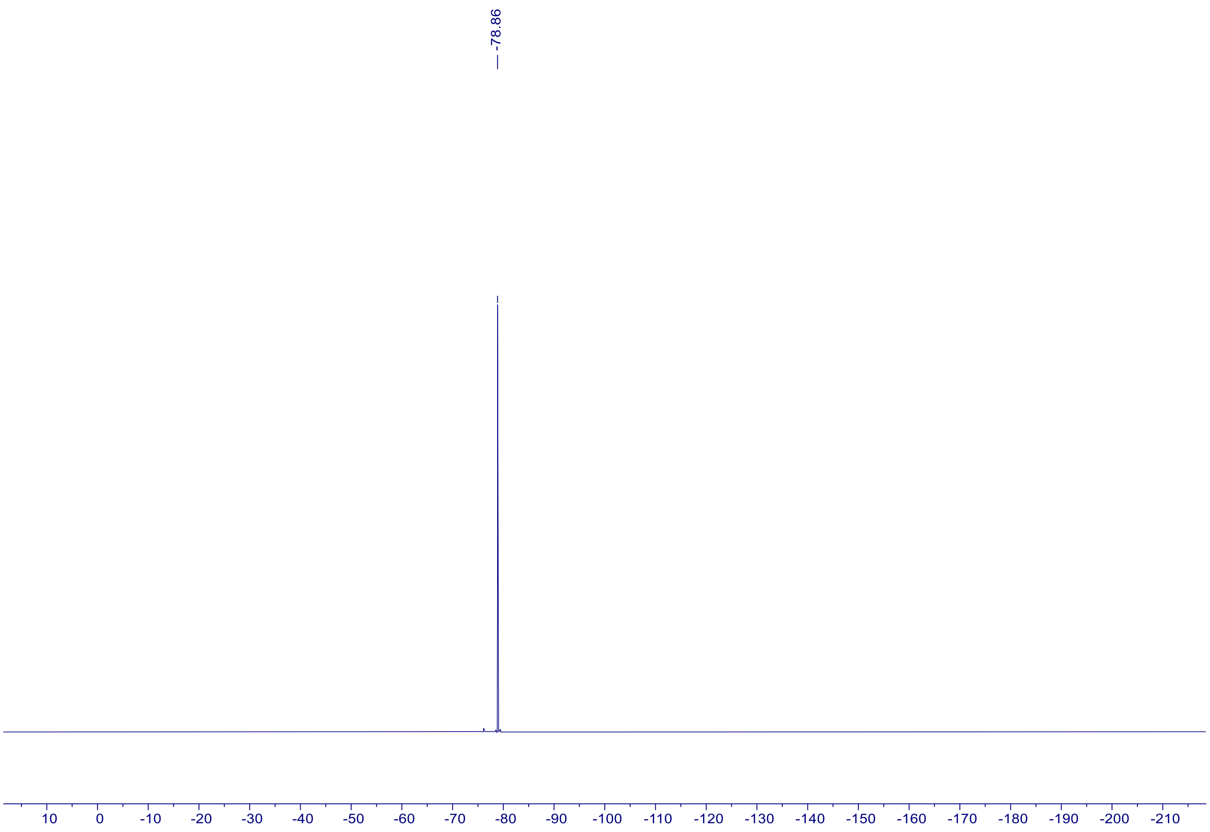

1q – ^{1}H NMR (400 MHz, acetone-d_6)

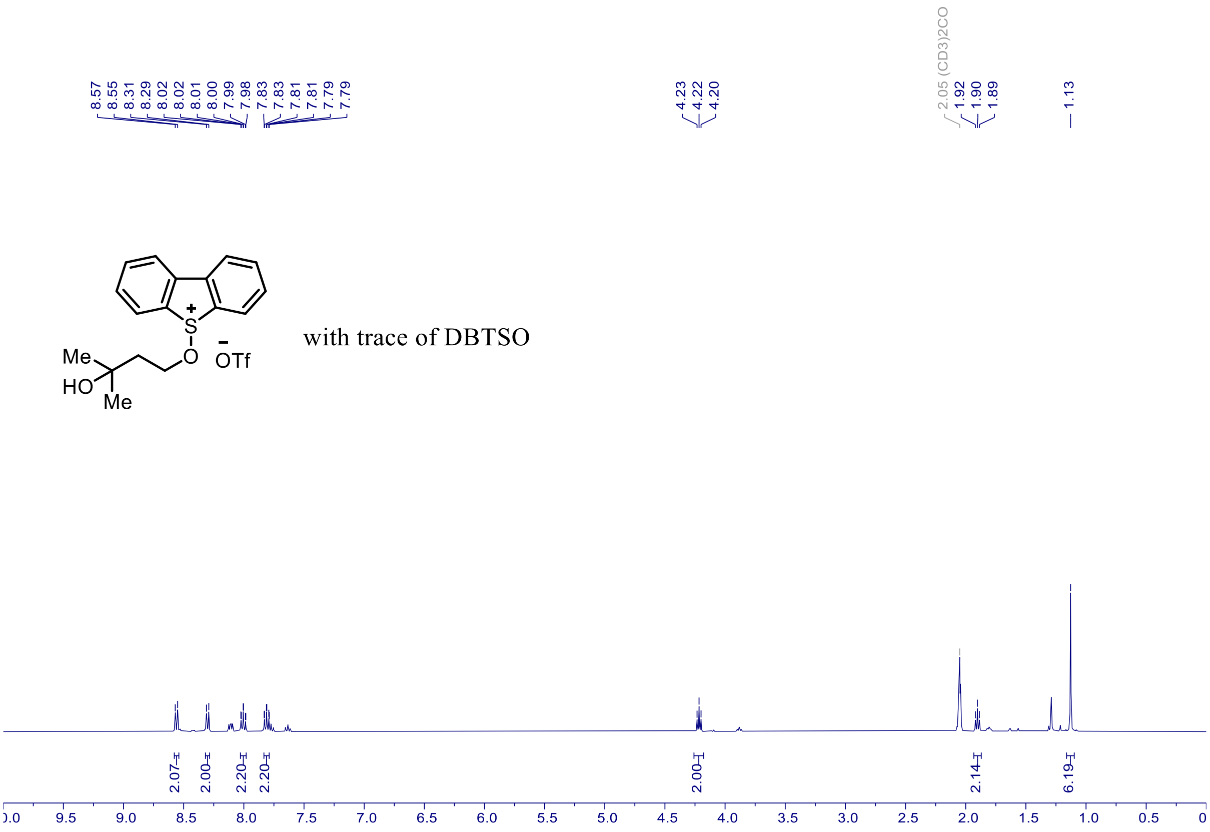

1q – ^{13}C NMR (101 MHz, acetone-d_6)

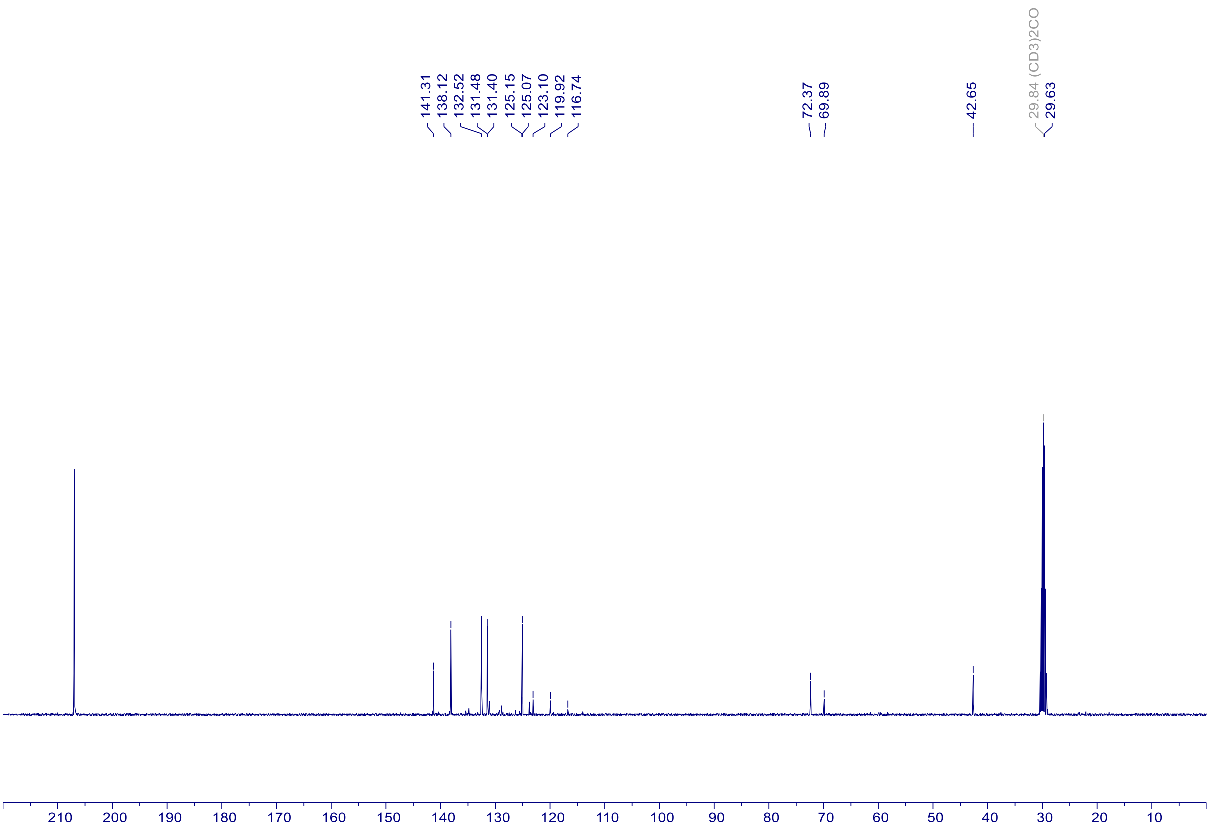

1q – ^{19}F NMR (376 MHz, acetone-d_6)

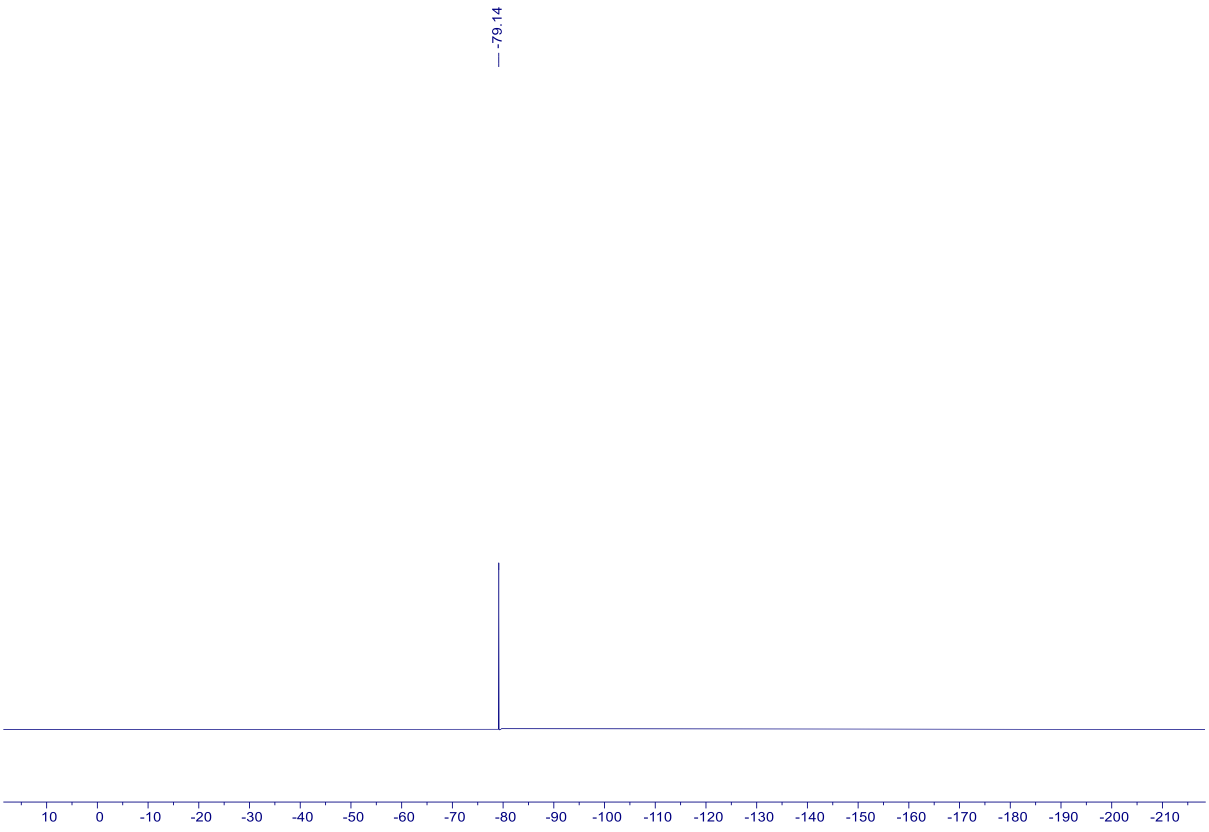

1r – ^{1}H NMR (400 MHz, acetone-d_6)

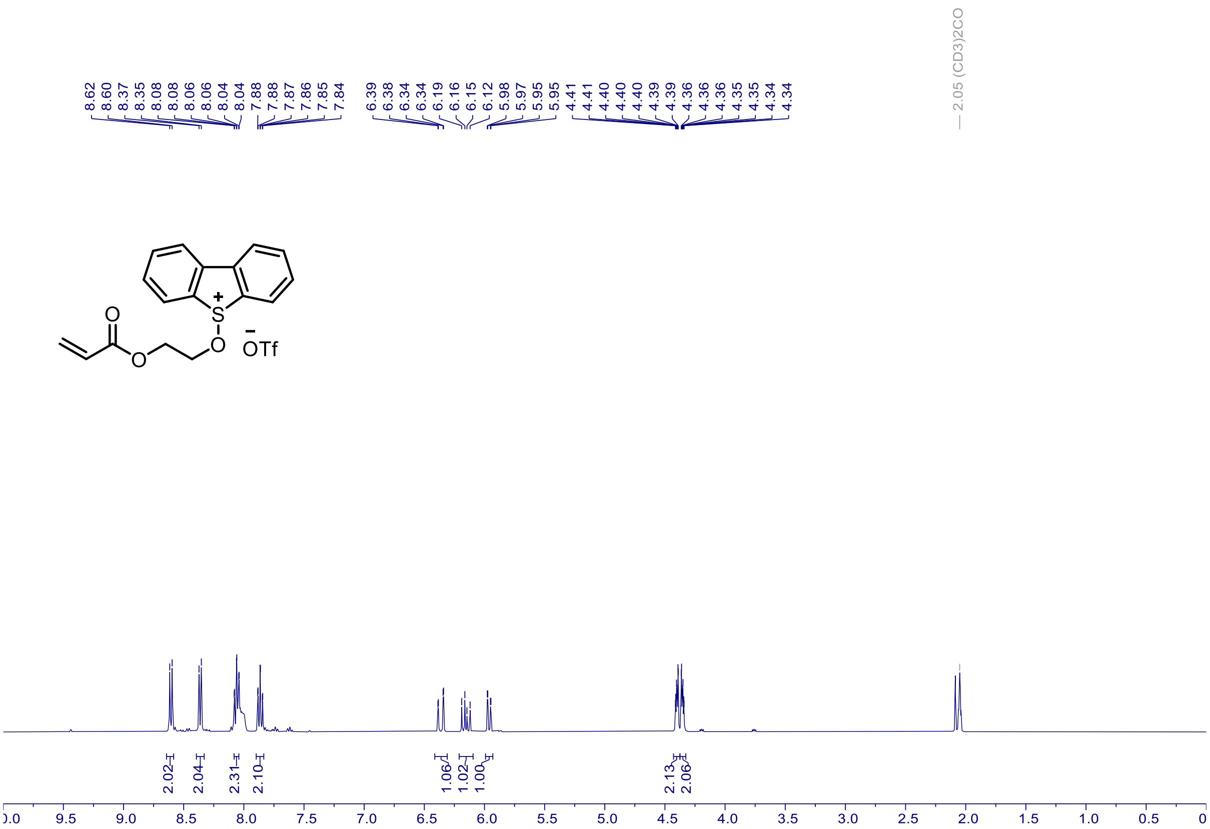

1r – ^{13}C NMR (101 MHz, acetone-d_6)

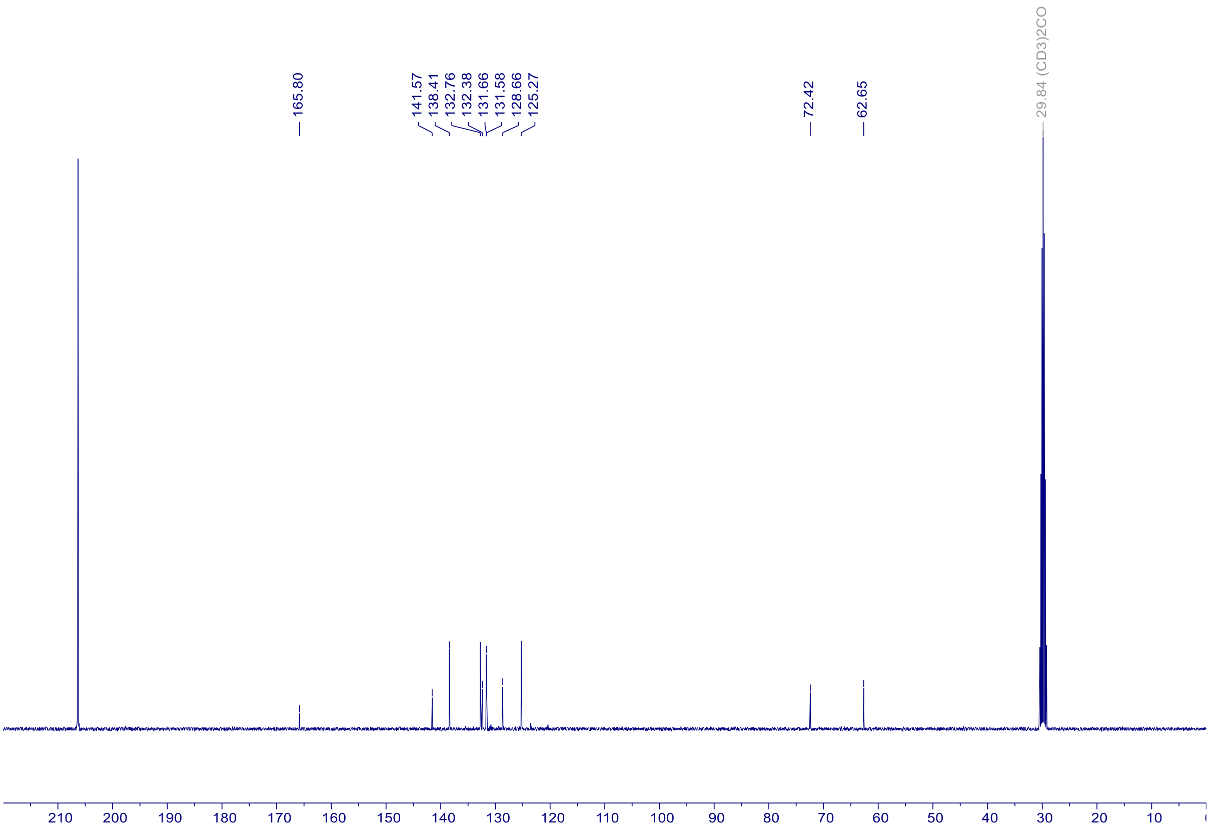

1r – ^{19}F NMR (376 MHz, acetone-d_6)

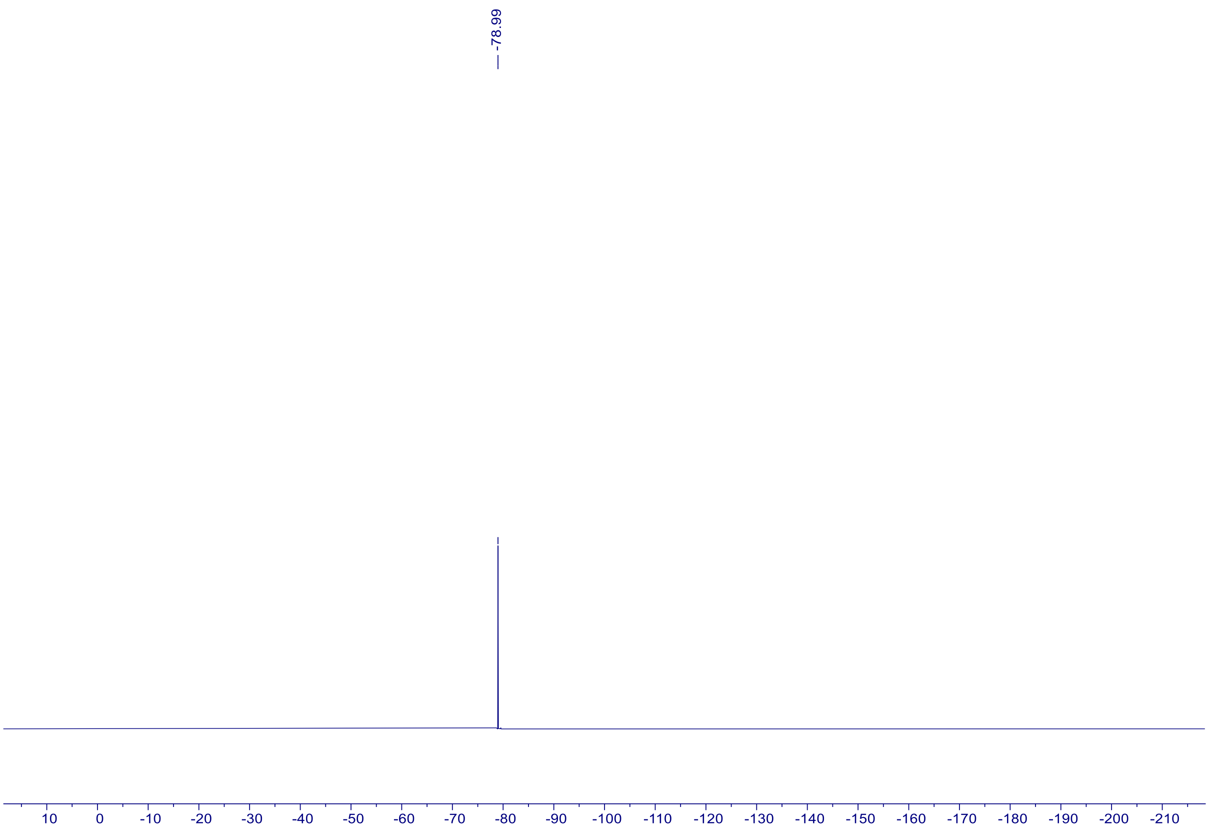

1s – ^{1}H NMR (400 MHz, acetone-d_6)

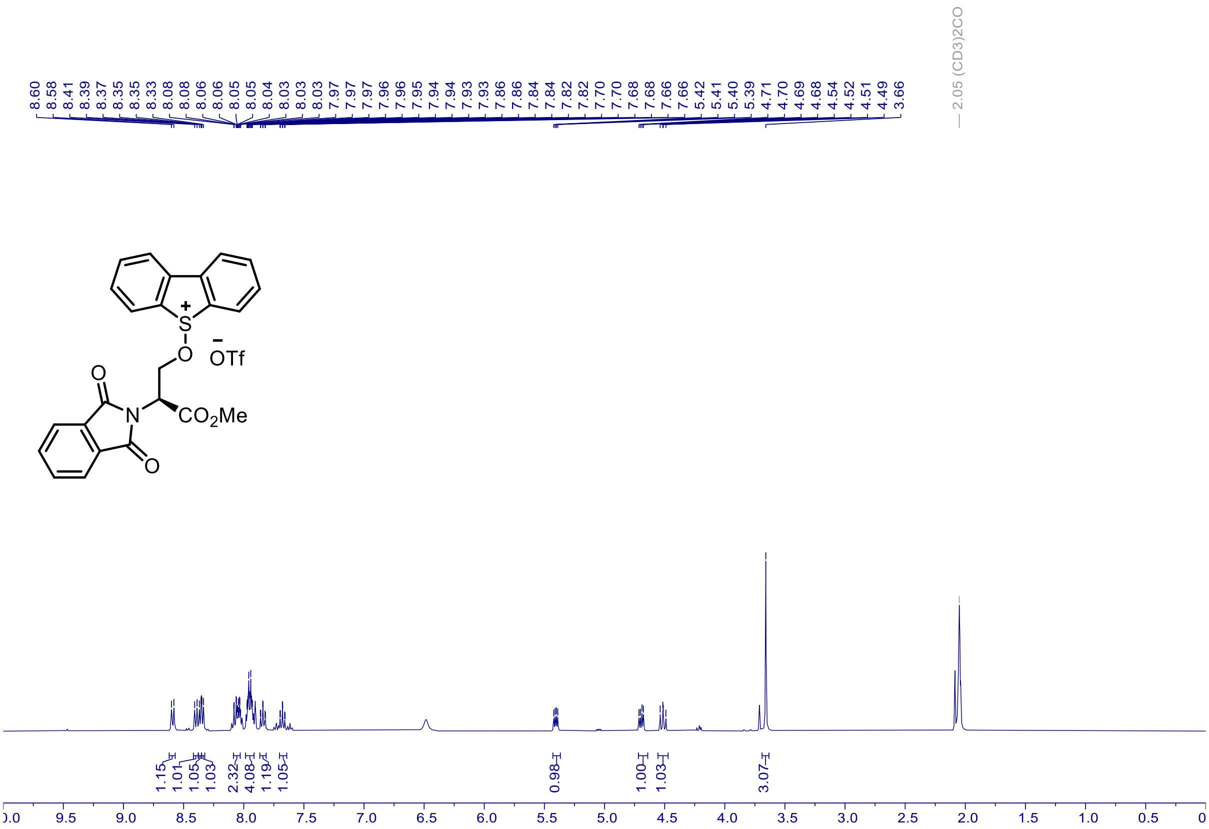

1s – ^{13}C NMR (101 MHz, acetone-d_6)

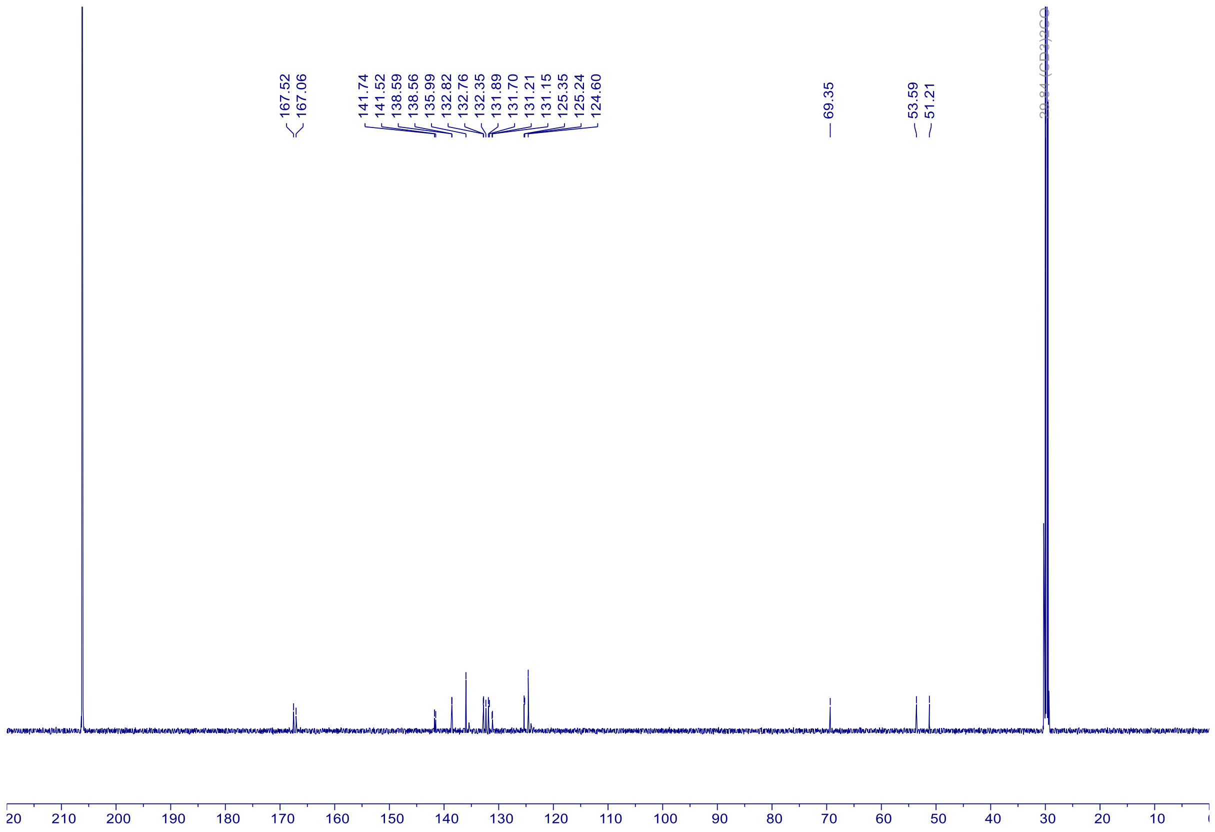

1s – ^{19}F NMR (376 MHz, acetone-d_6)

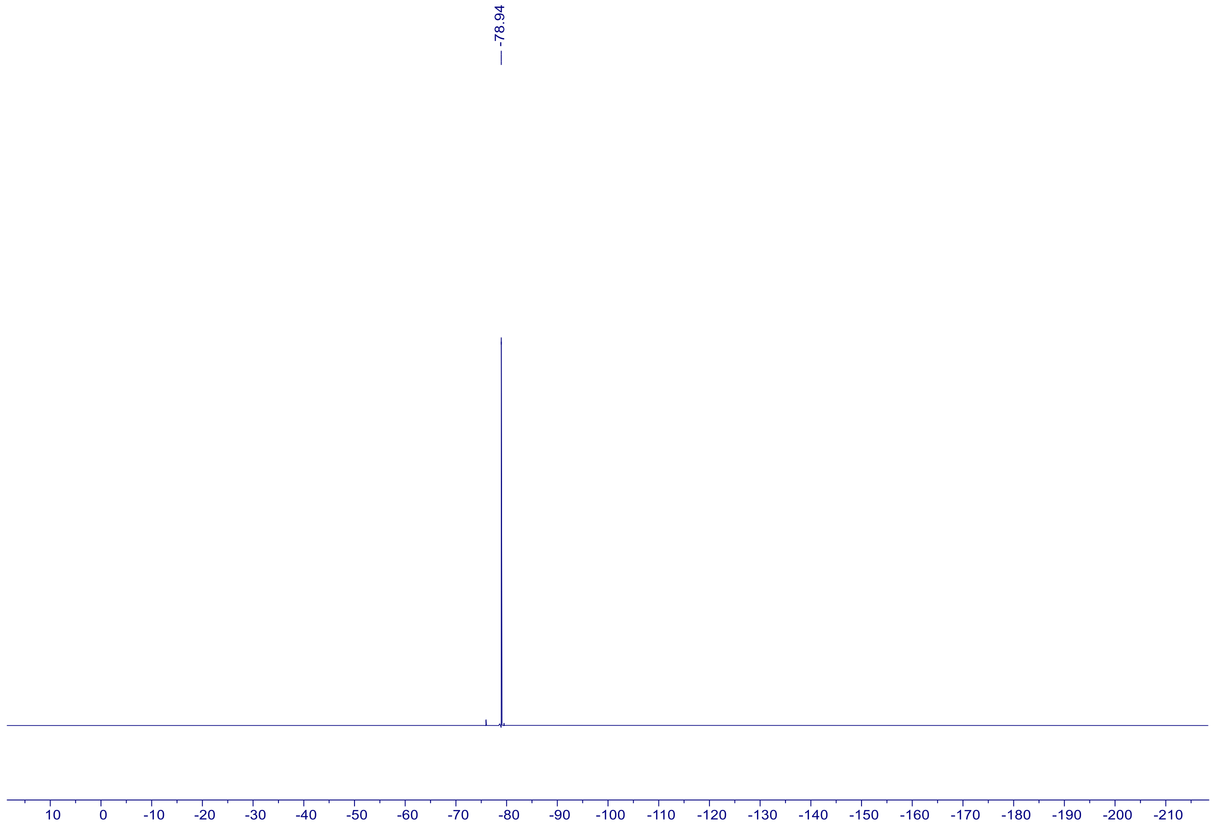

1s' – ^{1}H NMR (500 MHz, acetone-d_6)

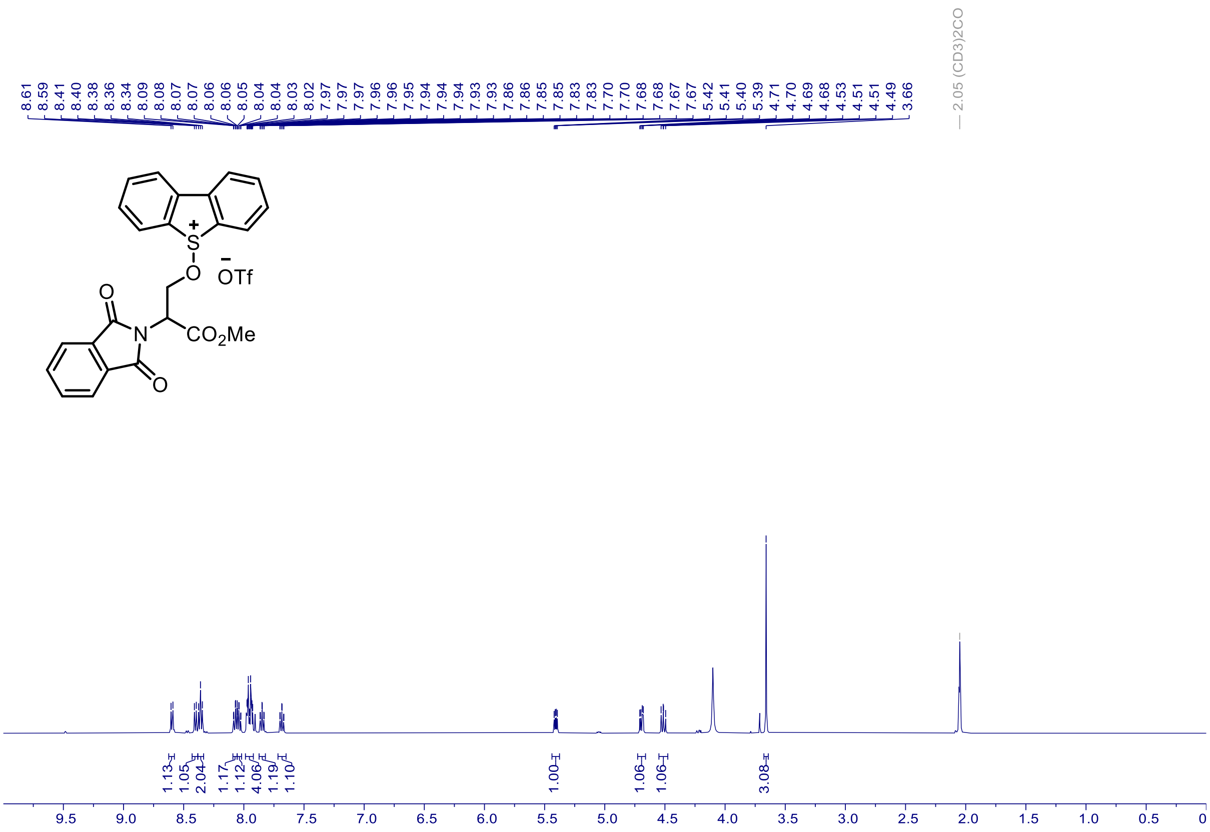

1s' – ^{13}C NMR (126 MHz, acetone-d_6)

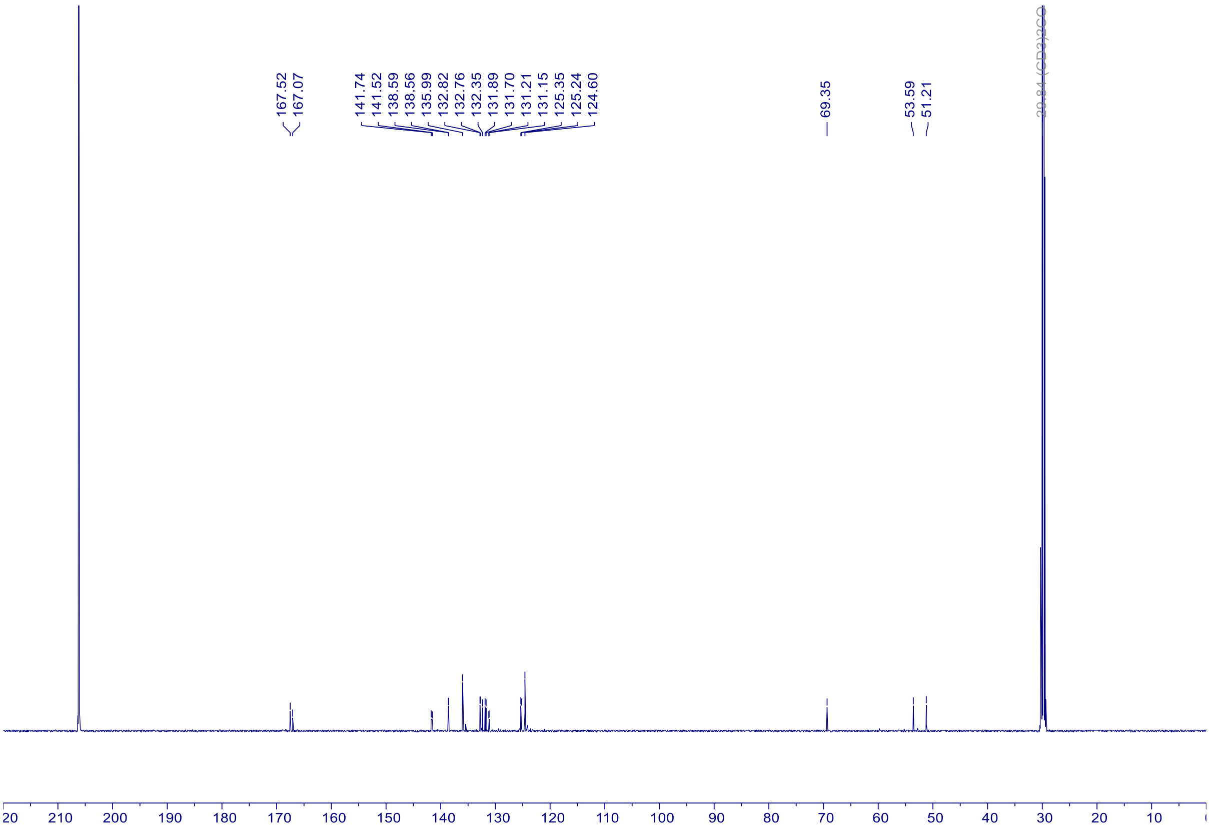

1s' – ^{19}F NMR (471 MHz, acetone-d_6)

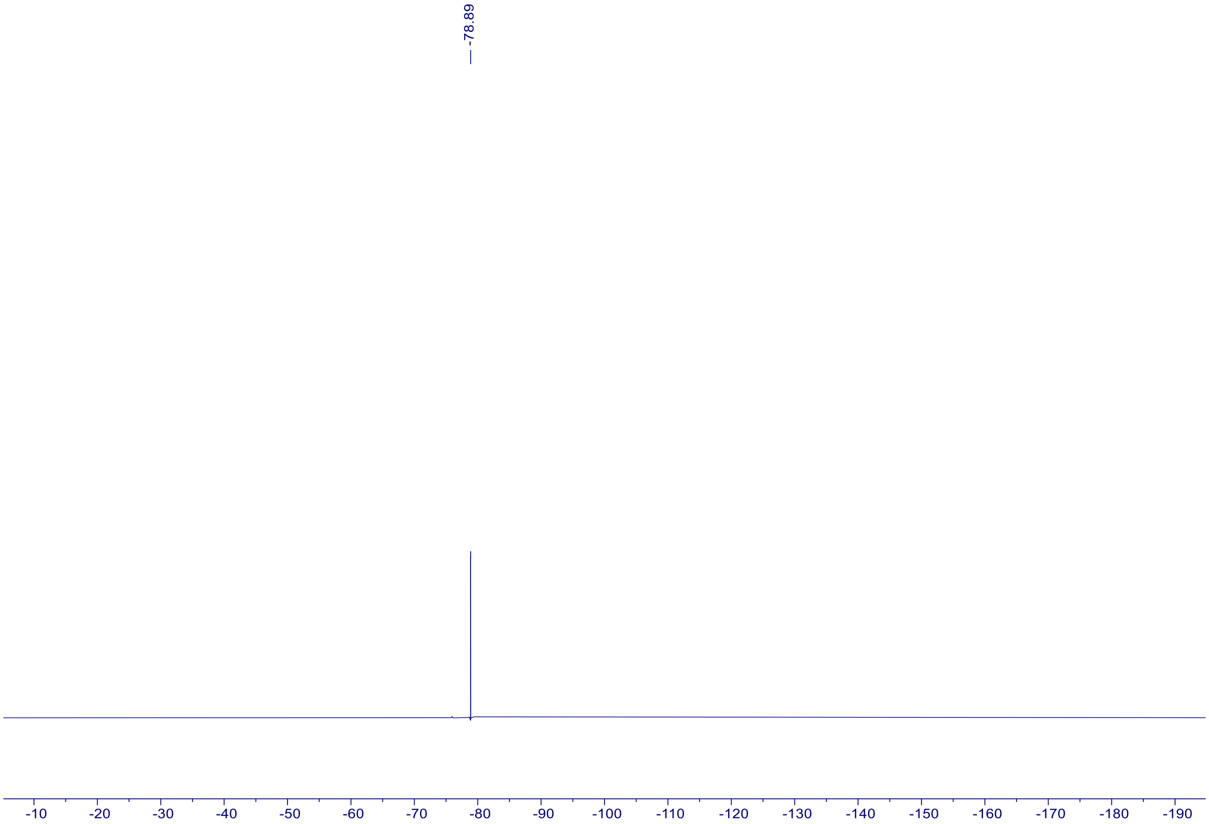

1t – ^{1}H NMR (400 MHz, acetone-d_6)

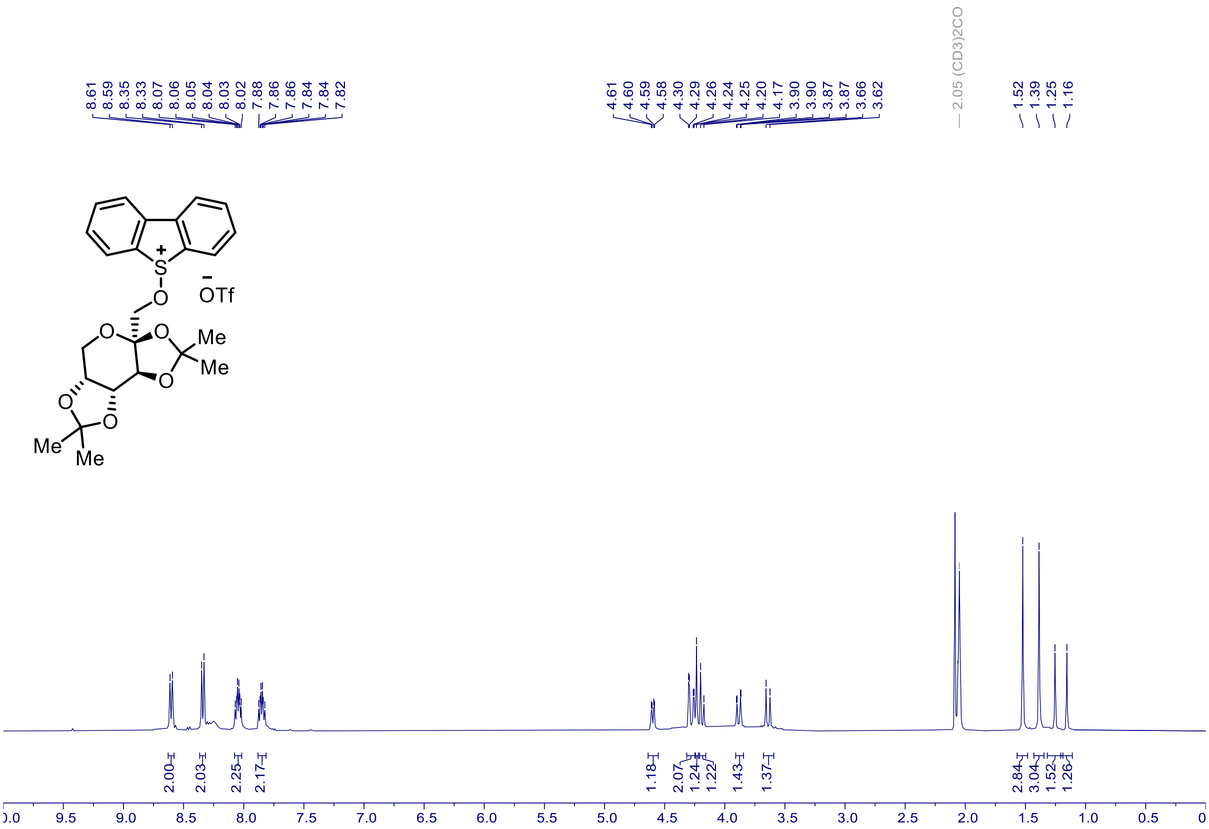

1t – ^{13}C NMR (101 MHz, acetone-d_6)

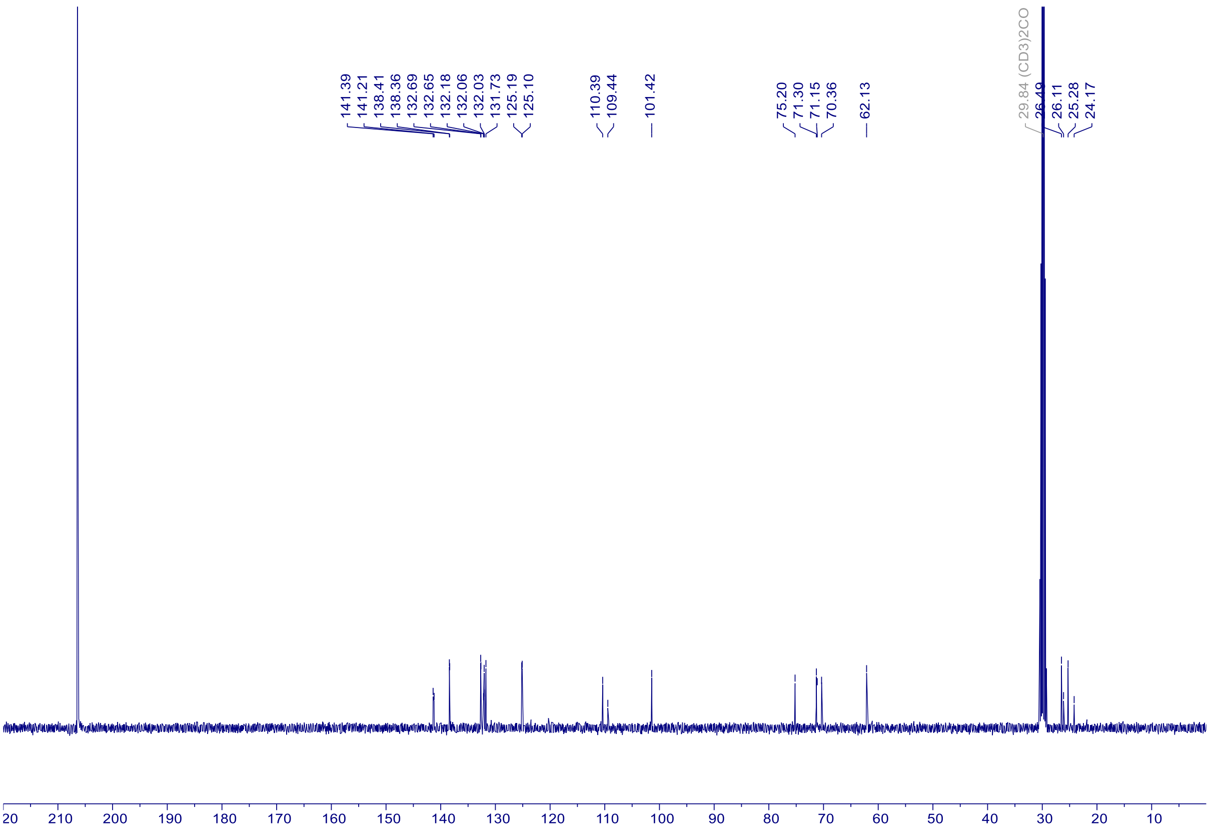

1t – ^{19}F NMR (376 MHz, acetone-d_6)

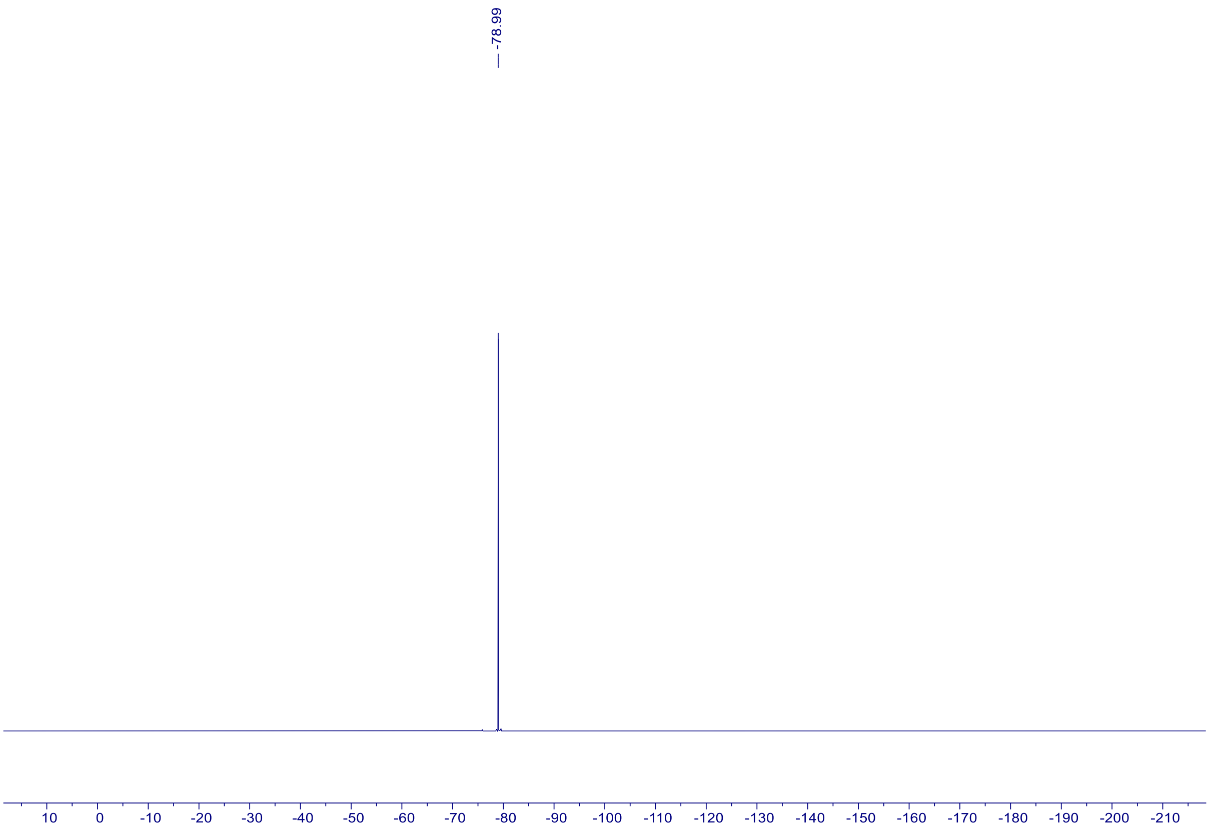

1u – ^{1}H NMR (400 MHz, acetone-d_6)

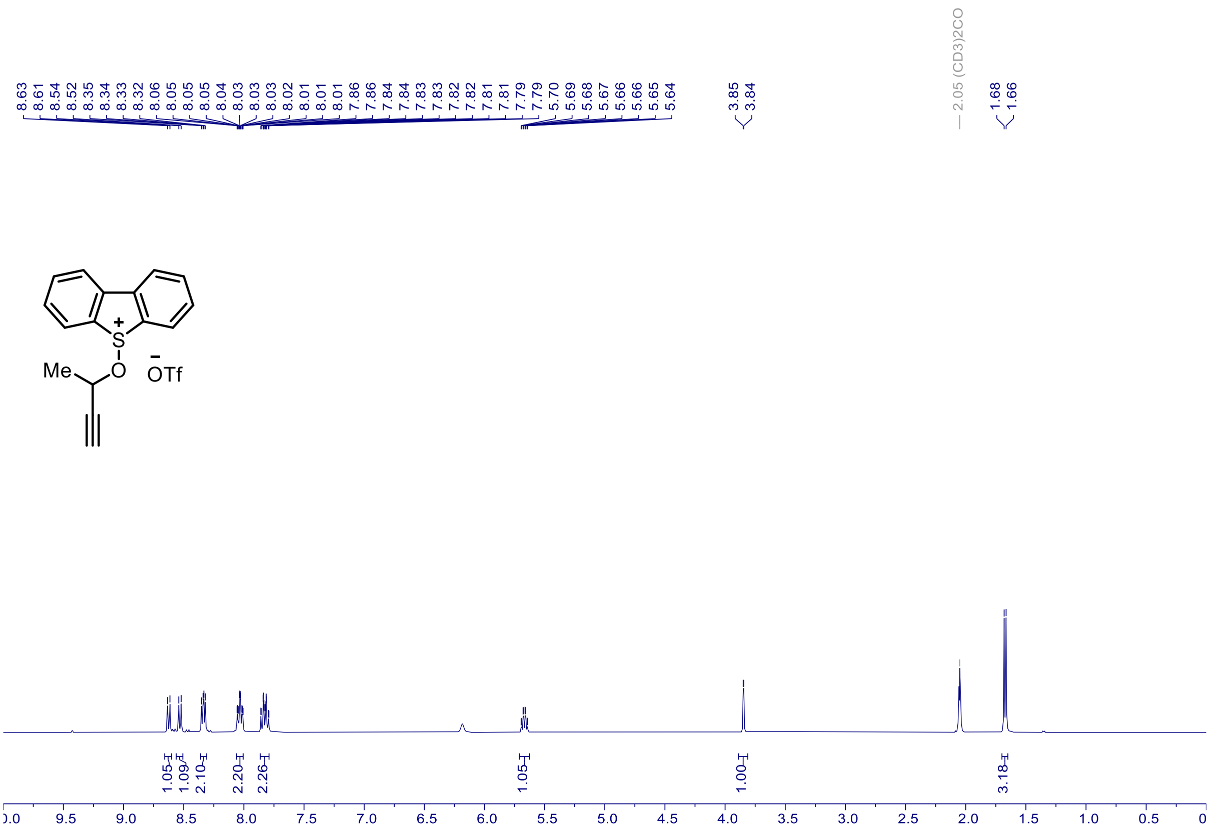

1u – ^{13}C NMR (101 MHz, acetone-d_6)

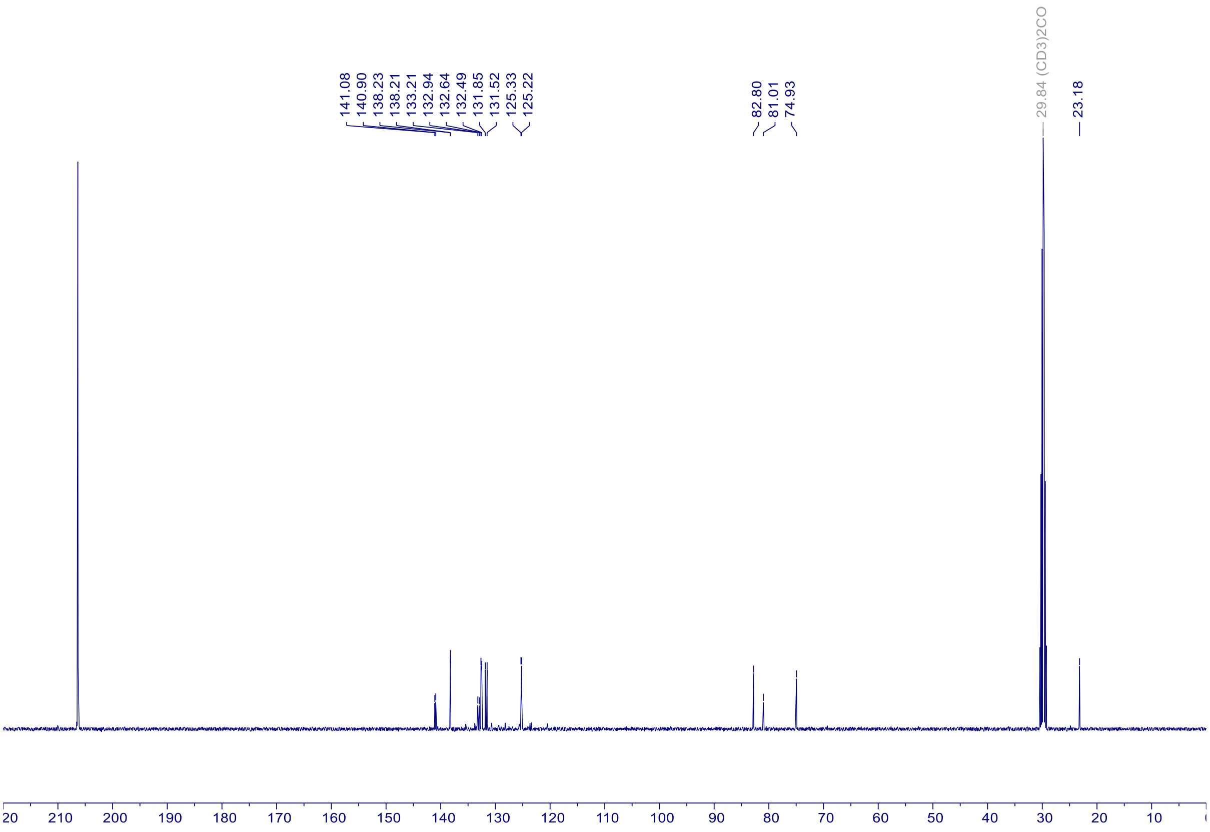

1u – ^{19}F NMR (376 MHz, acetone-d_6)

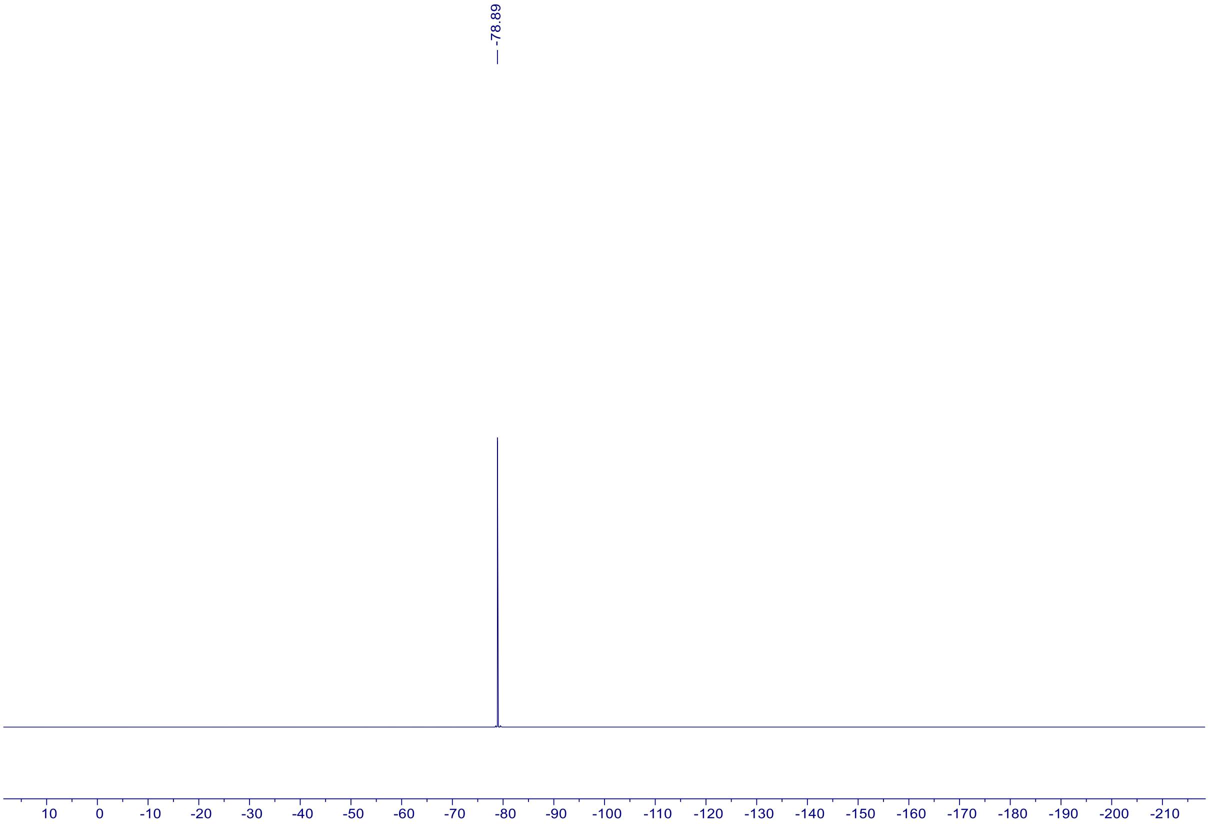

1v – ^{1}H NMR (400 MHz, acetone-d_6)

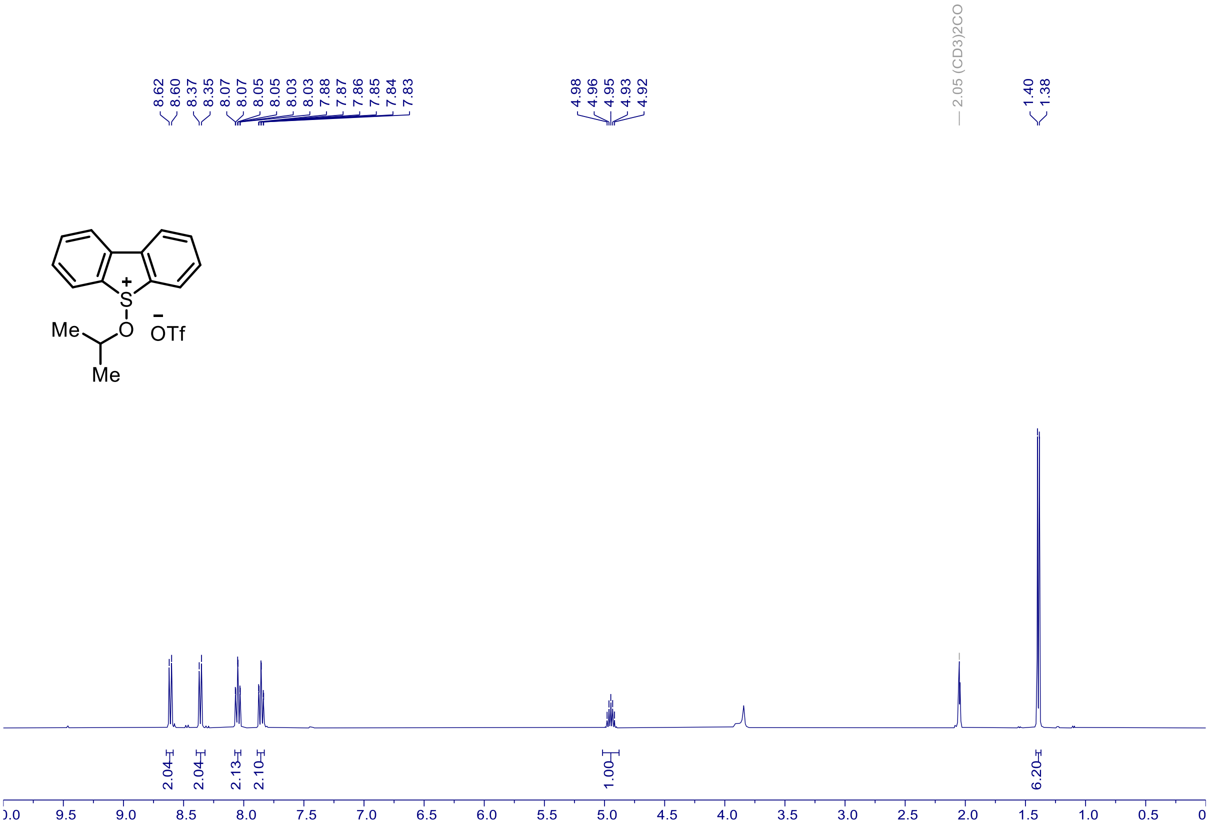

1v – ^{13}C NMR (101 MHz, acetone-d_6)

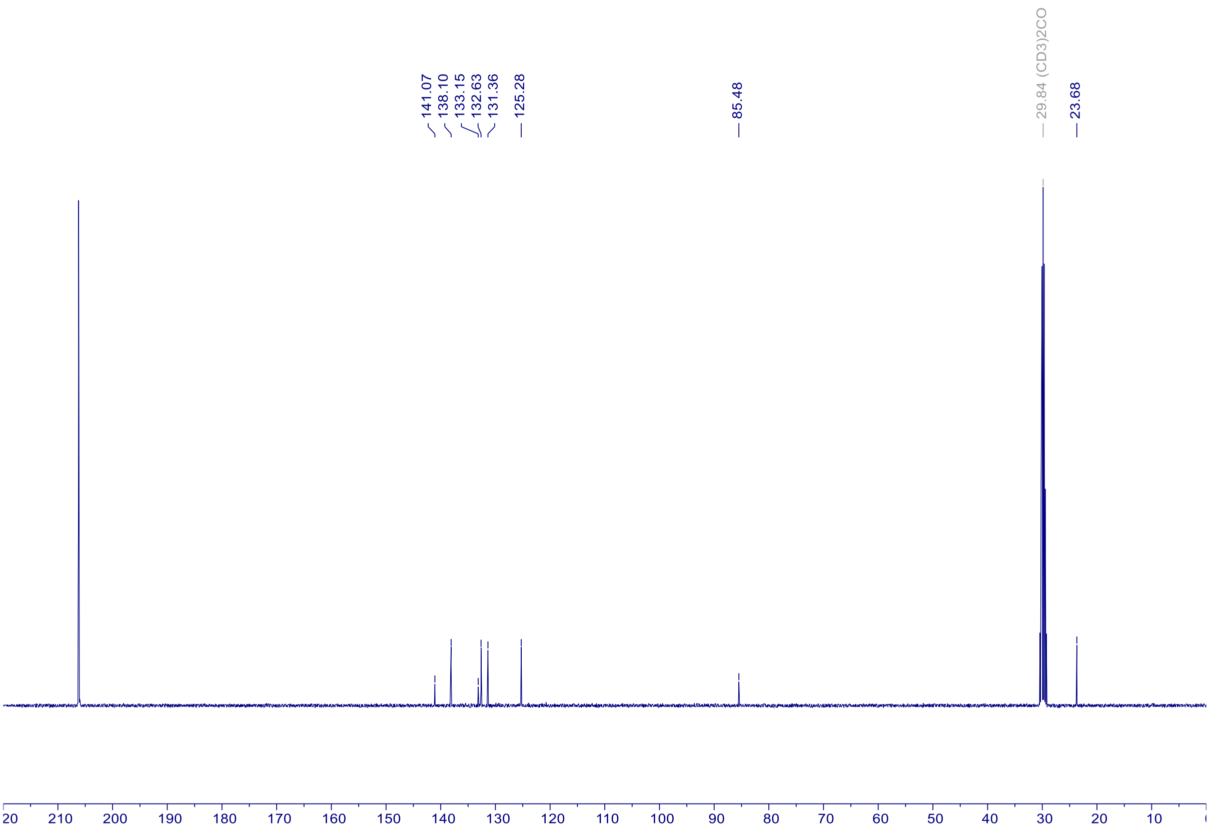

1v – ^{19}F NMR (376 MHz, acetone-d_6)

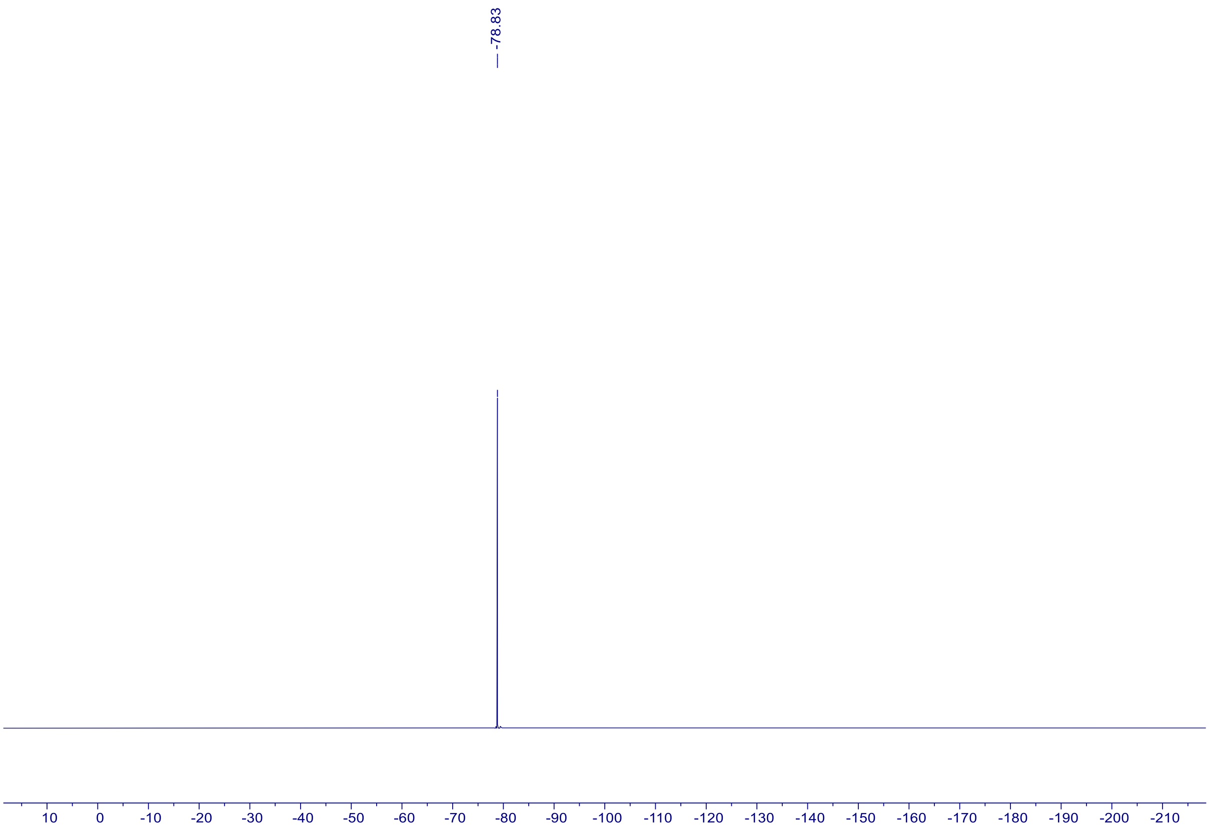

1w – ^{1}H NMR (400 MHz, acetone-d_6)

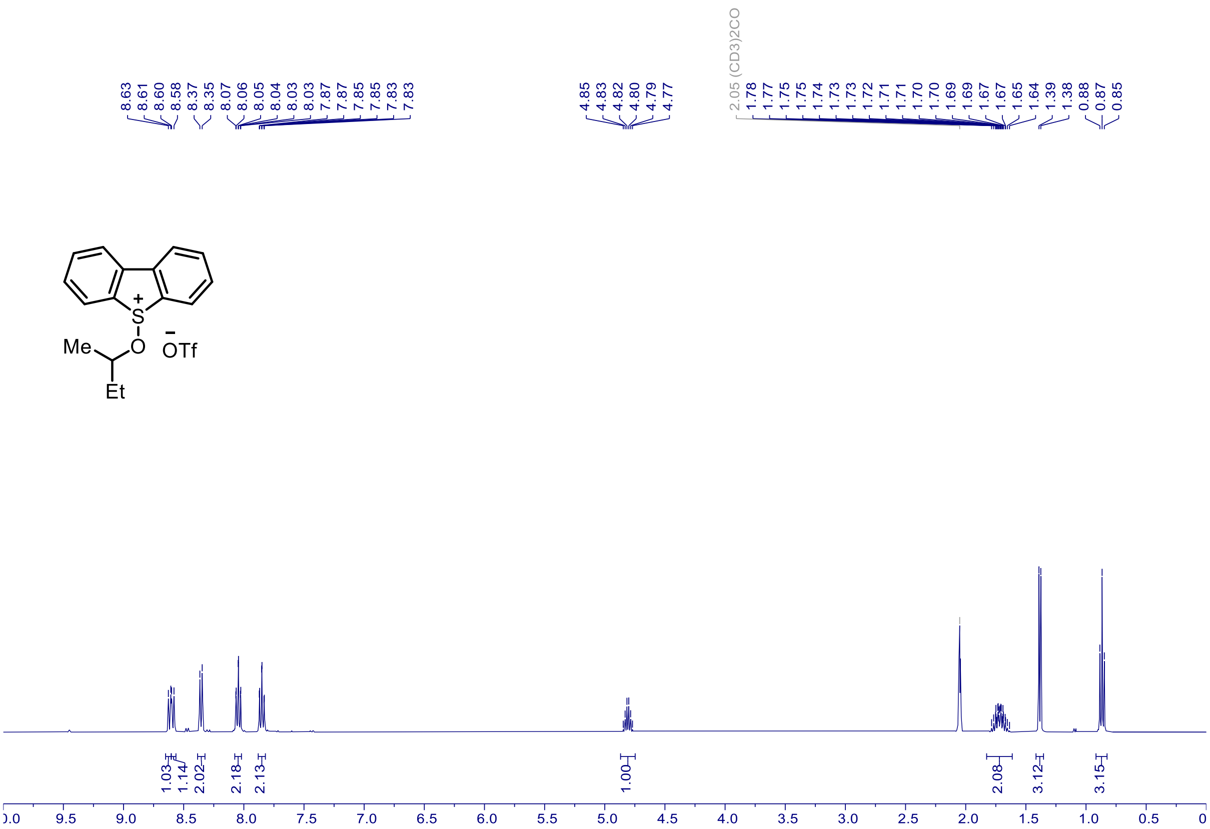

1w – ^{13}C NMR (101 MHz, acetone-d_6)

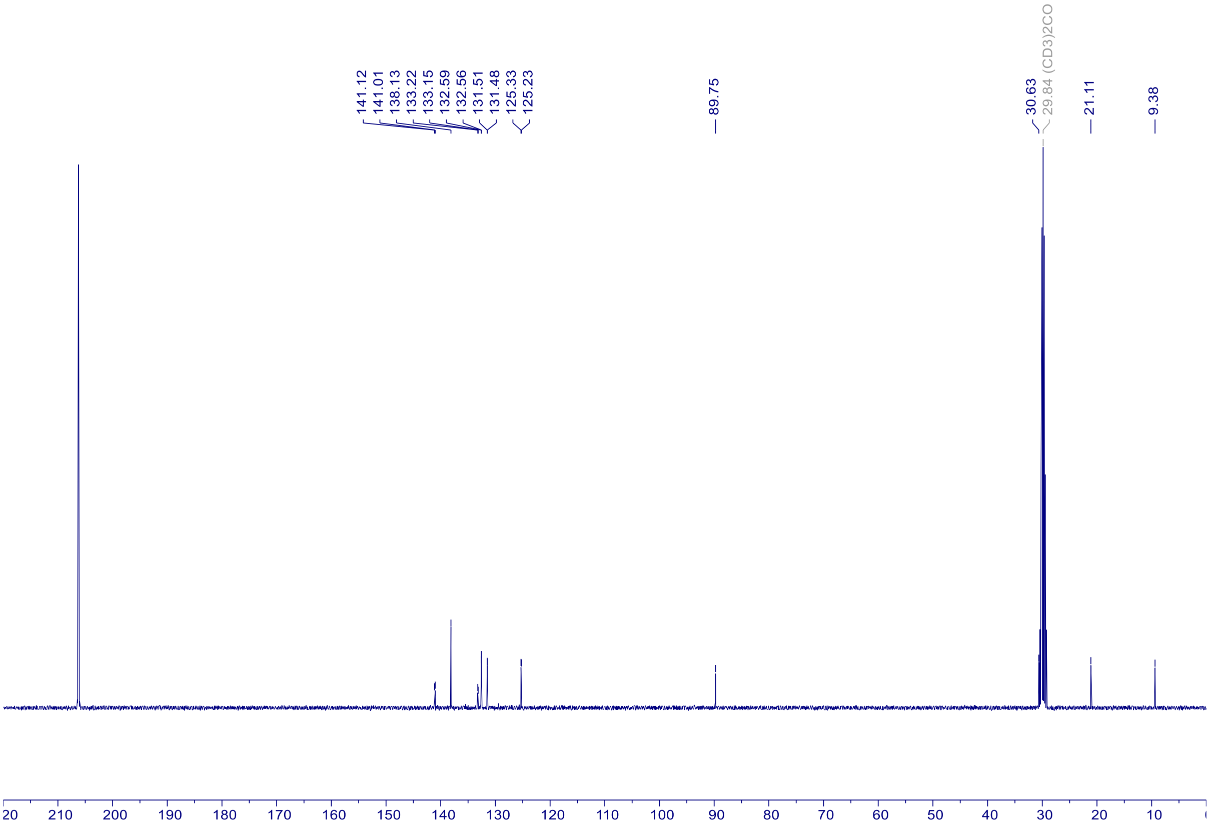

1w – ^{19}F NMR (376 MHz, acetone-d_6)

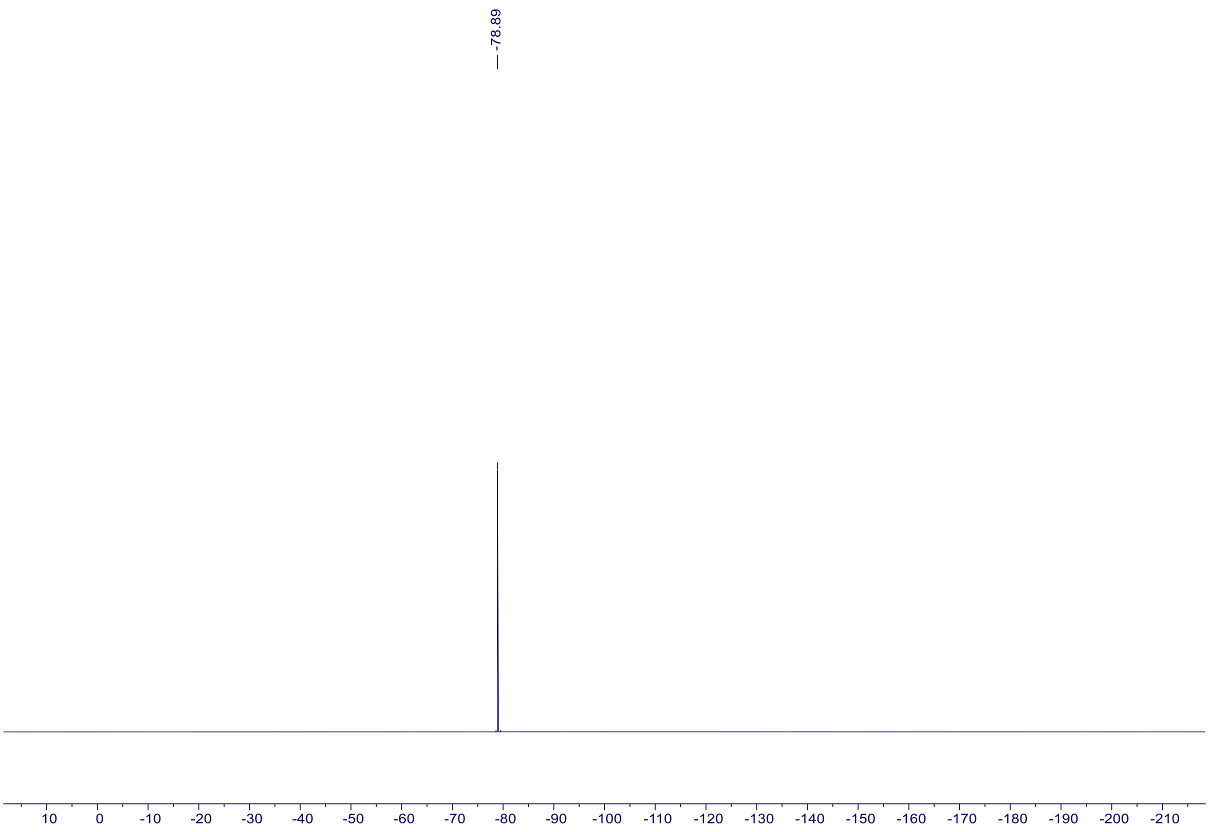

1x – ^{1}H NMR (500 MHz, acetone-d_6)

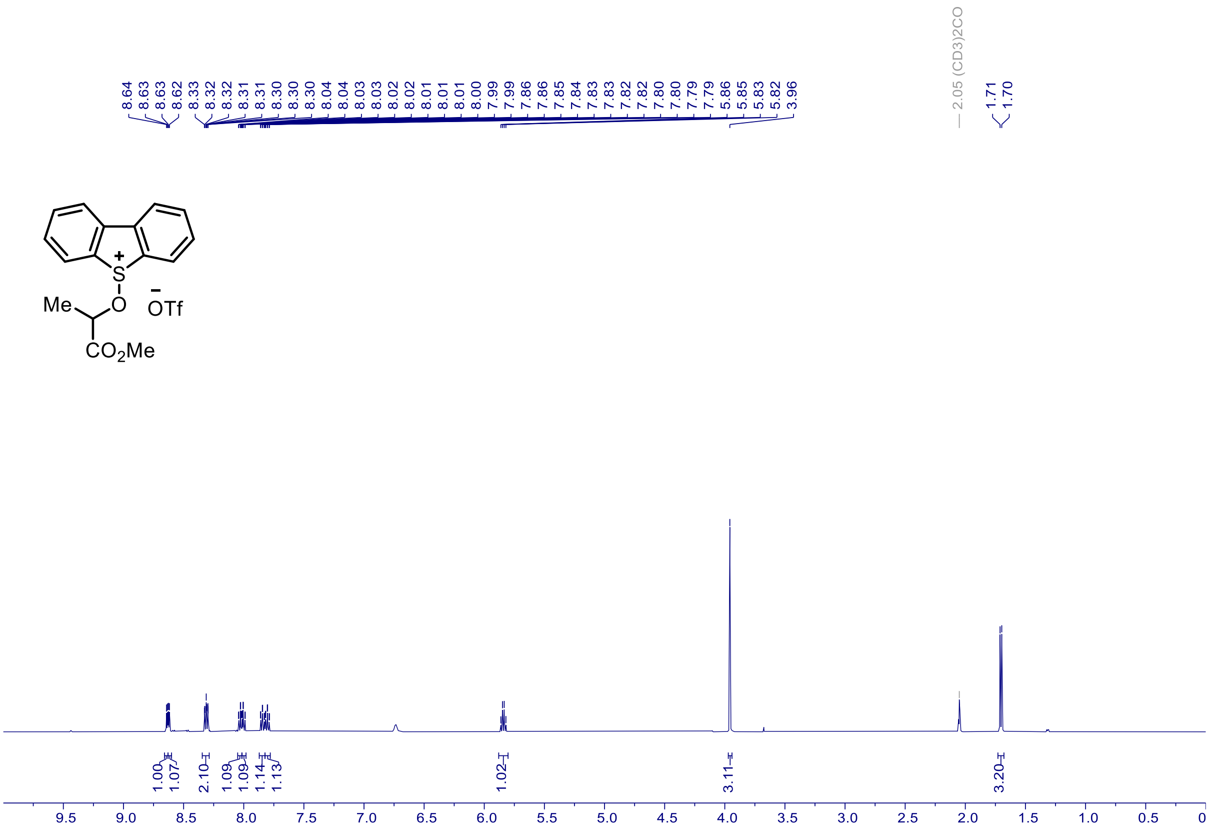

1x – ^{13}C NMR (126 MHz, acetone-d_6)

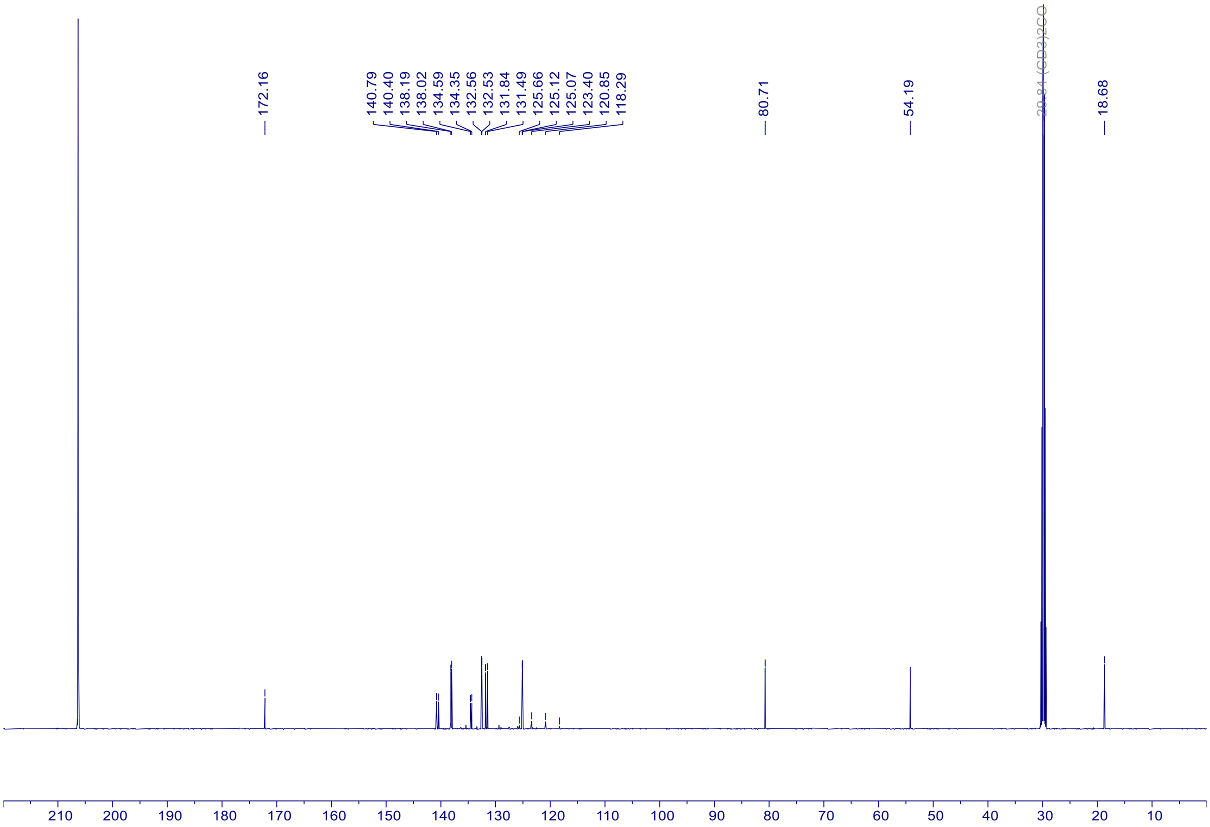

1x – ^{19}F NMR (471 MHz, acetone-d_6)

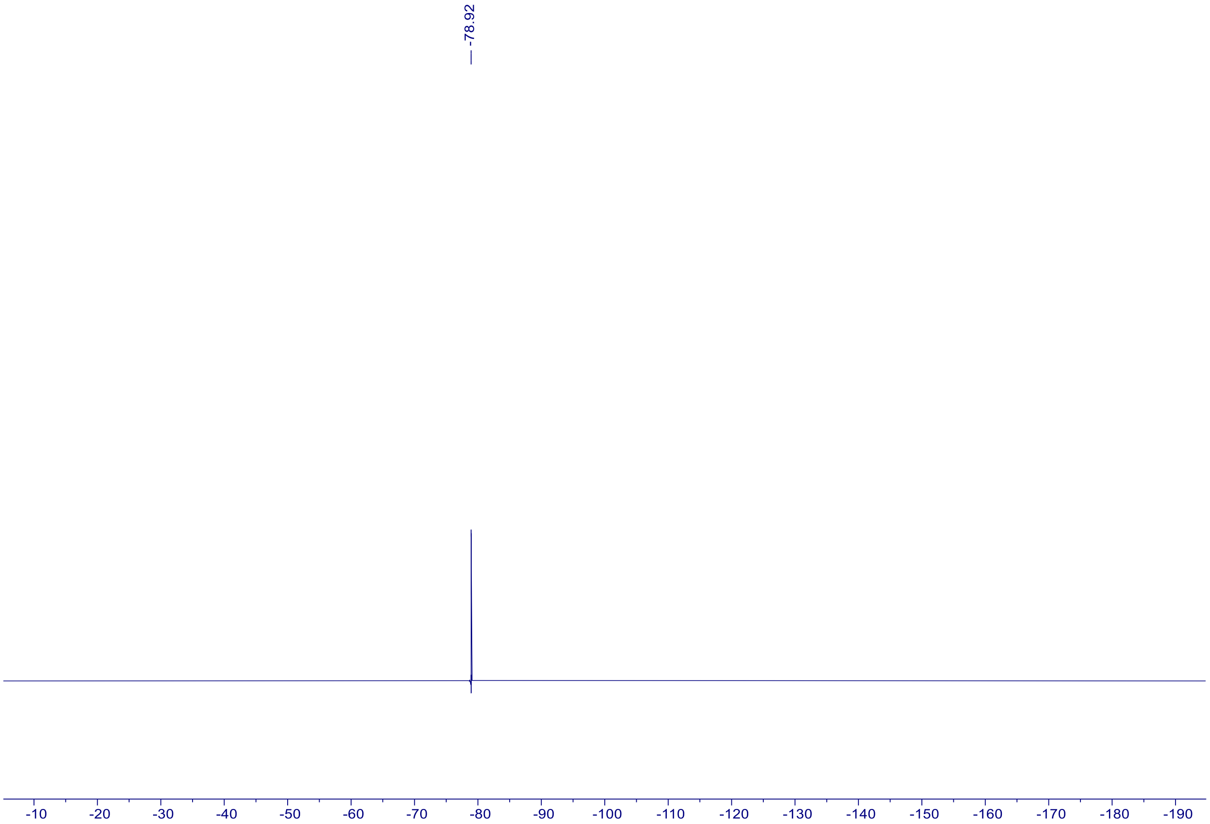

1y – ^{1}H NMR (400 MHz, acetone-d_6)

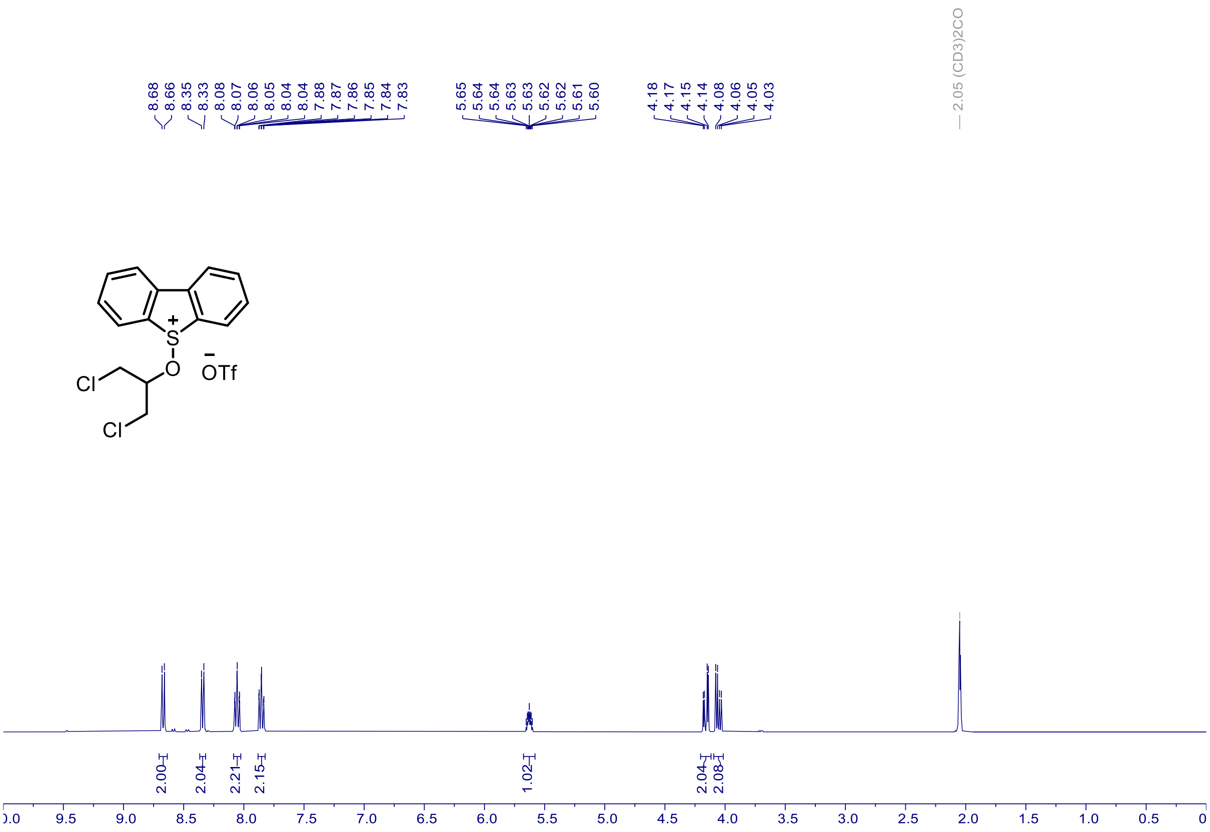

1y – ^{13}C NMR (101 MHz, acetone-d_6)

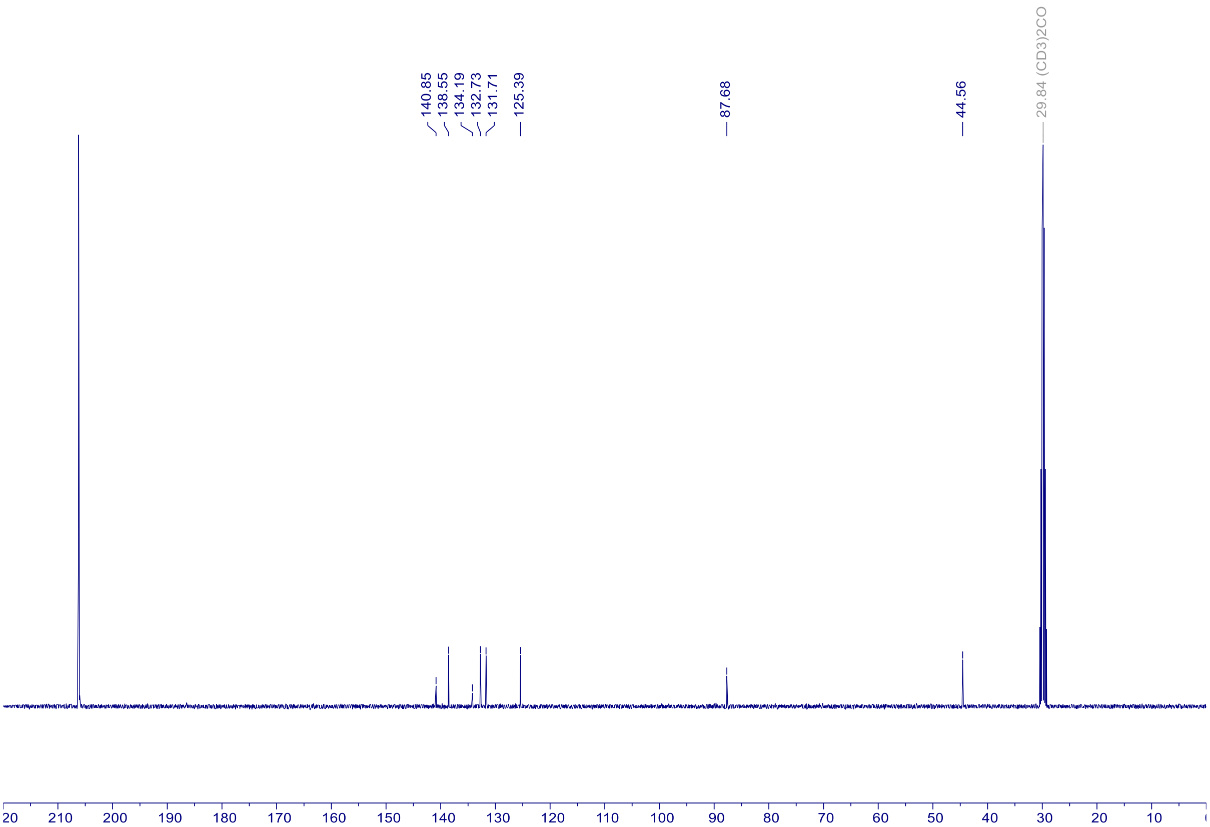

1y – ^{19}F NMR (376 MHz, acetone-d_6)

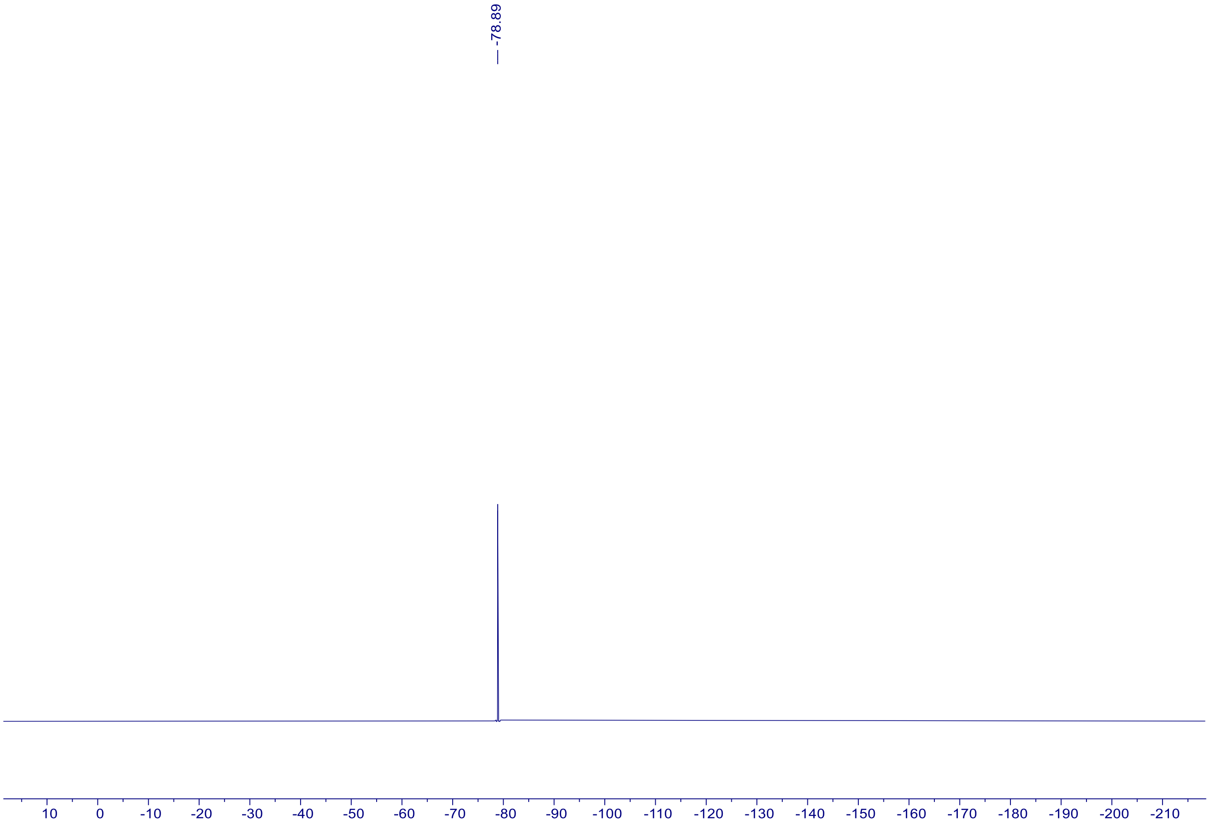

1z – ^{1}H NMR (400 MHz, acetone-d_6)

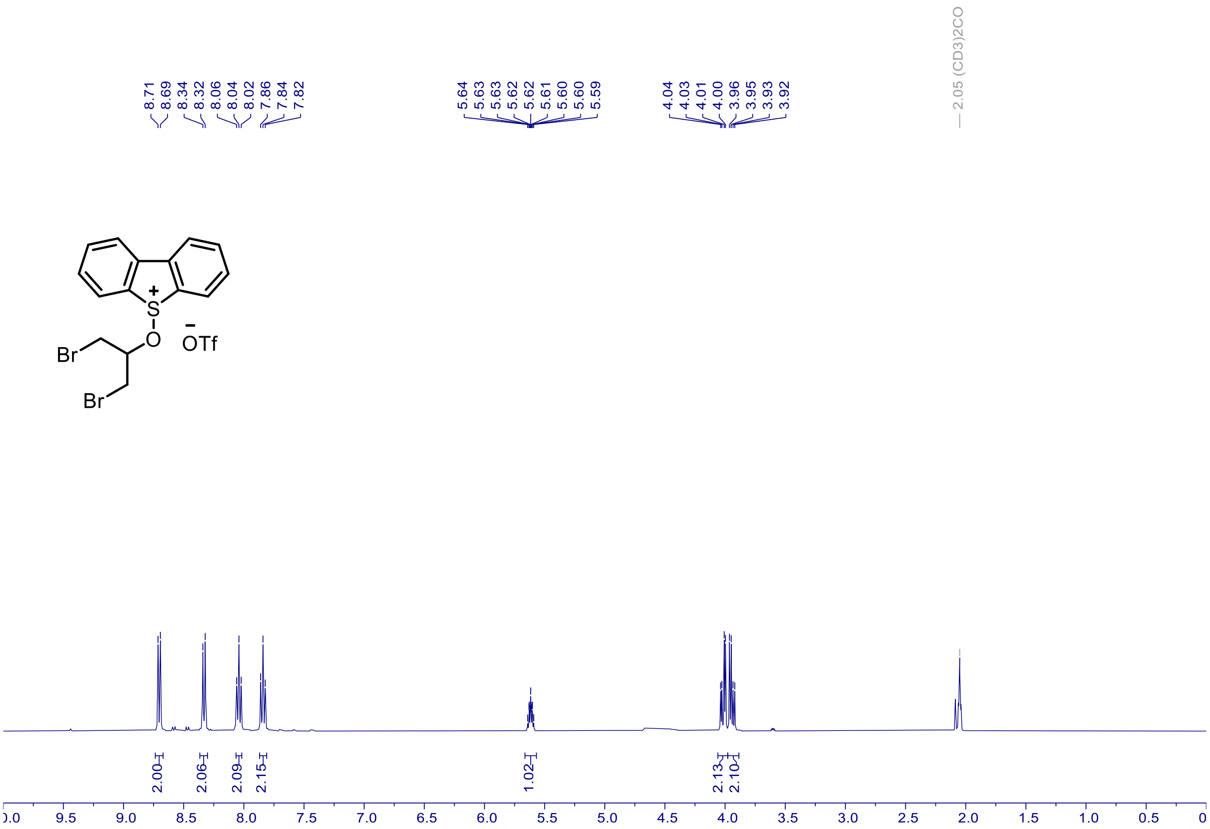

1z – ^{13}C NMR (101 MHz, acetone-d_6)

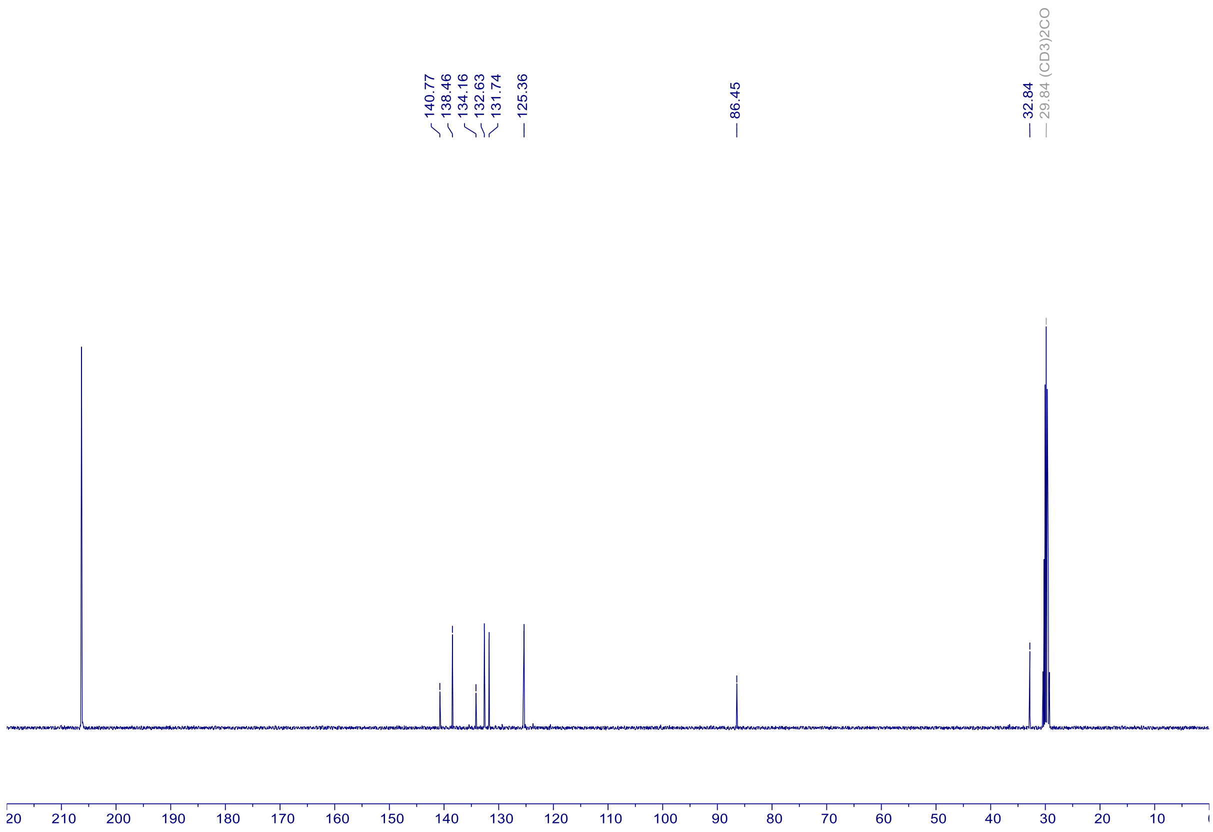

1z – ^{19}F NMR (376 MHz, acetone-d_6)

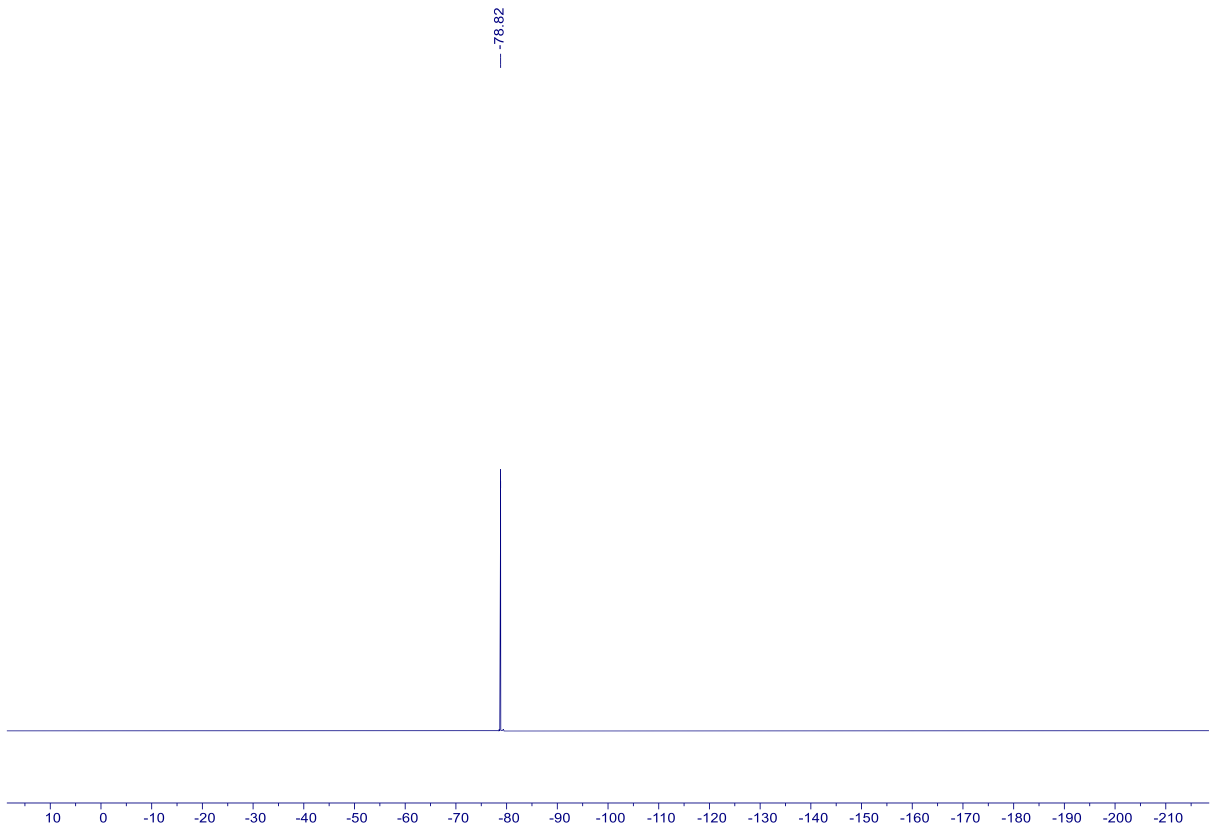

1aa – ^{1}H NMR (400 MHz, acetone-d_6)

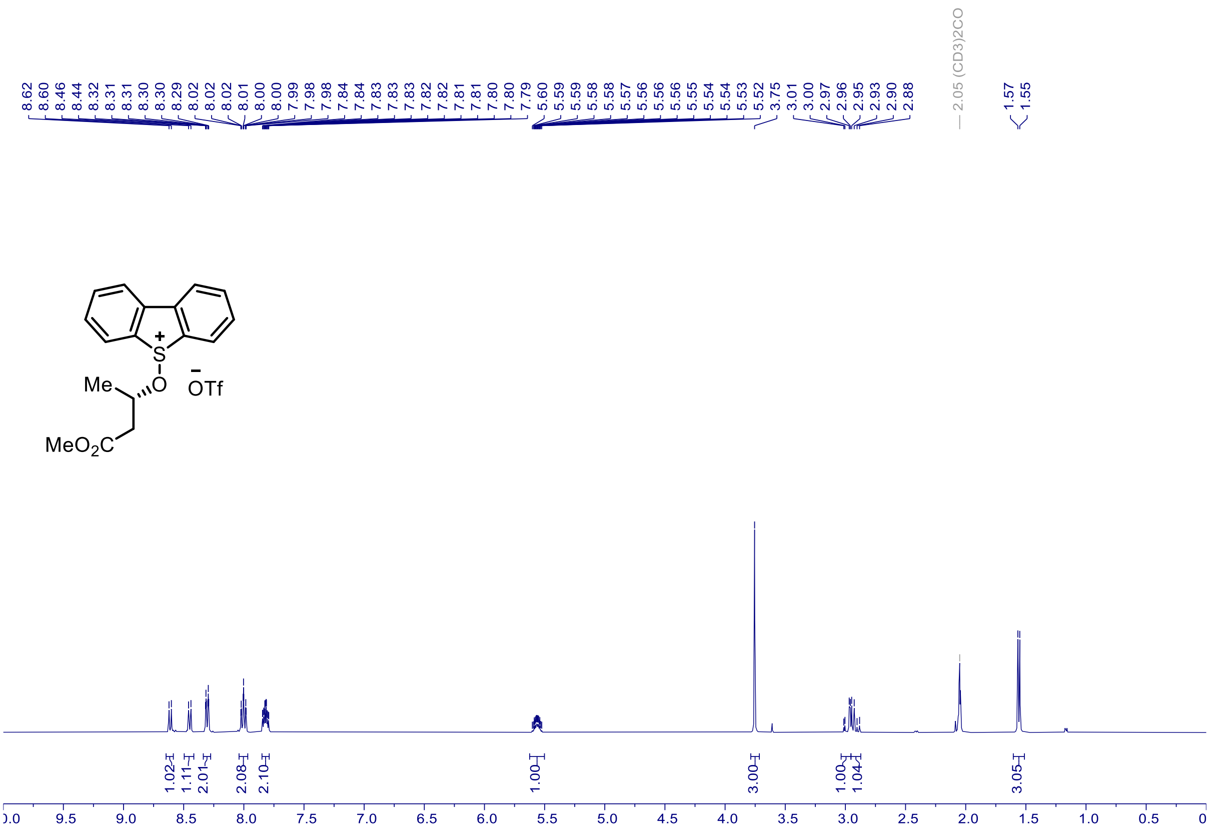

1aa – ^{13}C NMR (101 MHz, acetone-d_6)

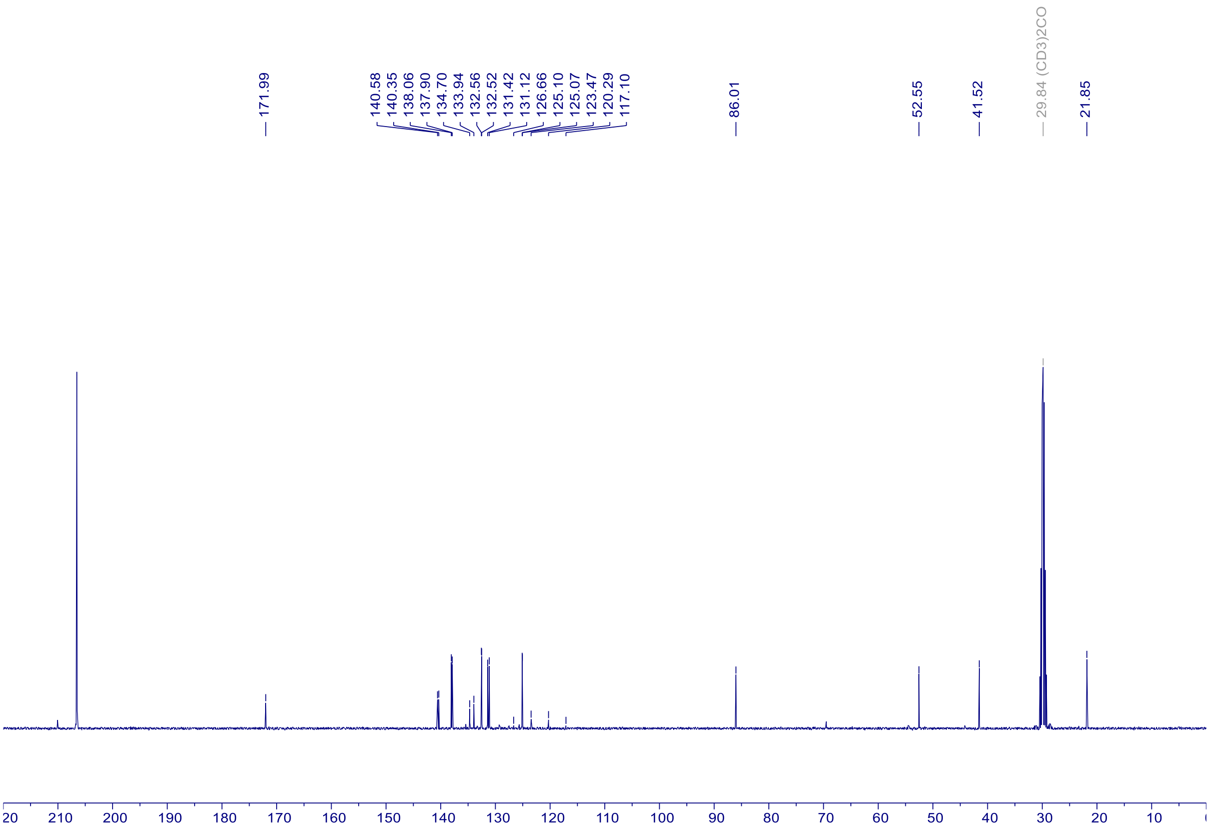

1aa – ^{19}F NMR (376 MHz, acetone-d_6)

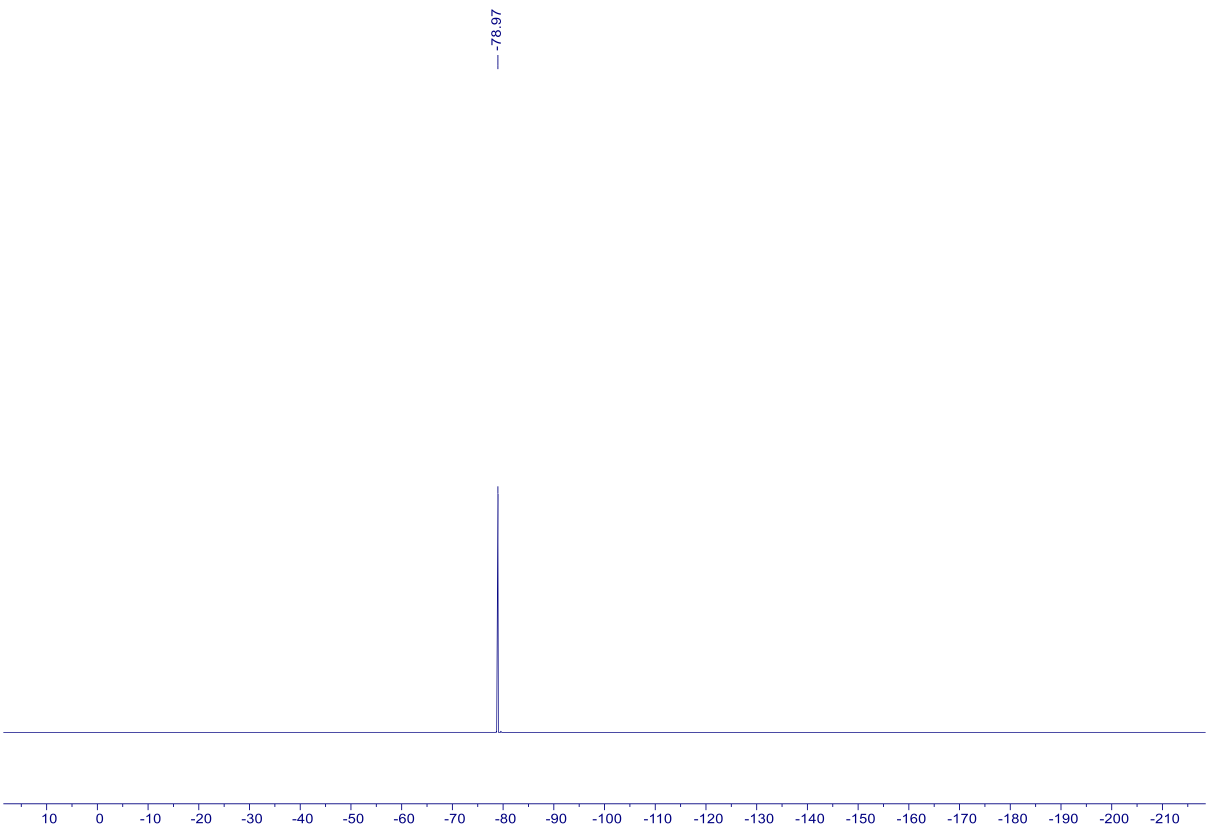

1aa' – ^{1}H NMR (400 MHz, acetone-d_6)

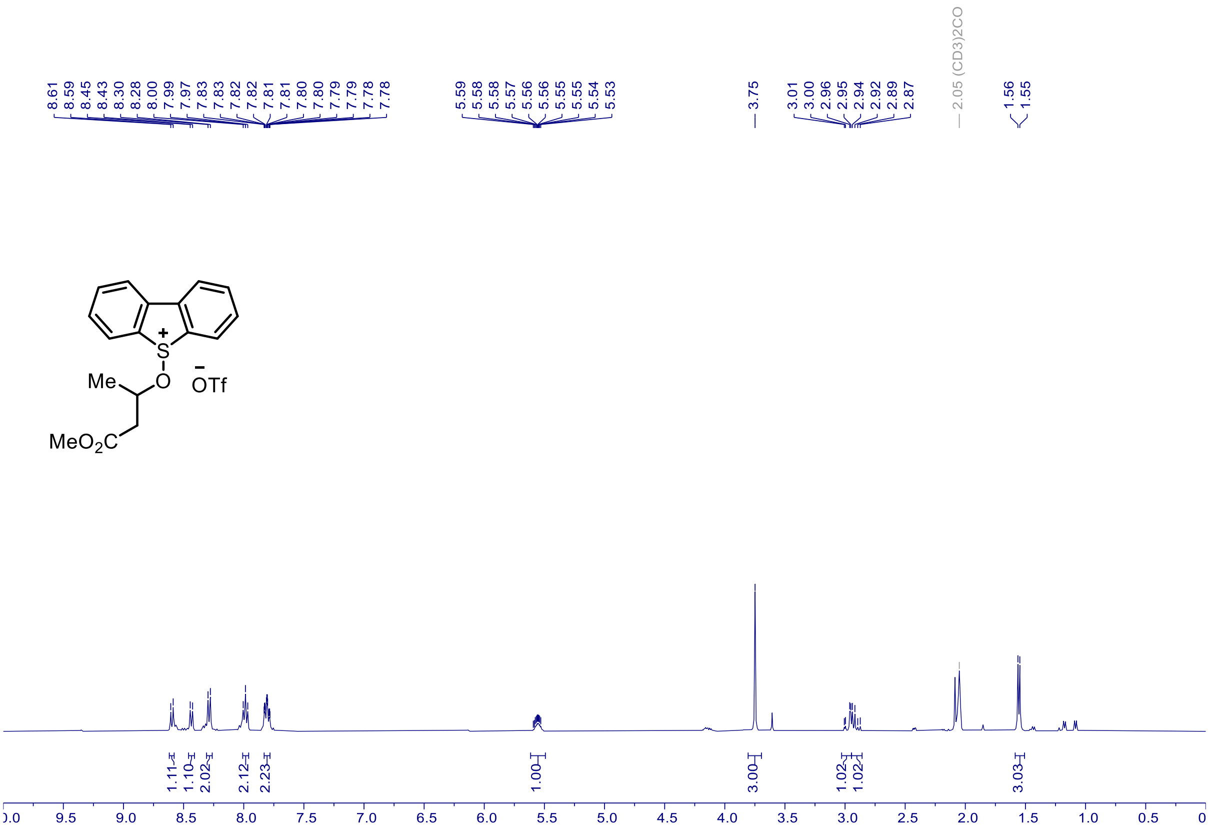

1aa' – ^{13}C NMR (101 MHz, acetone-d_6)

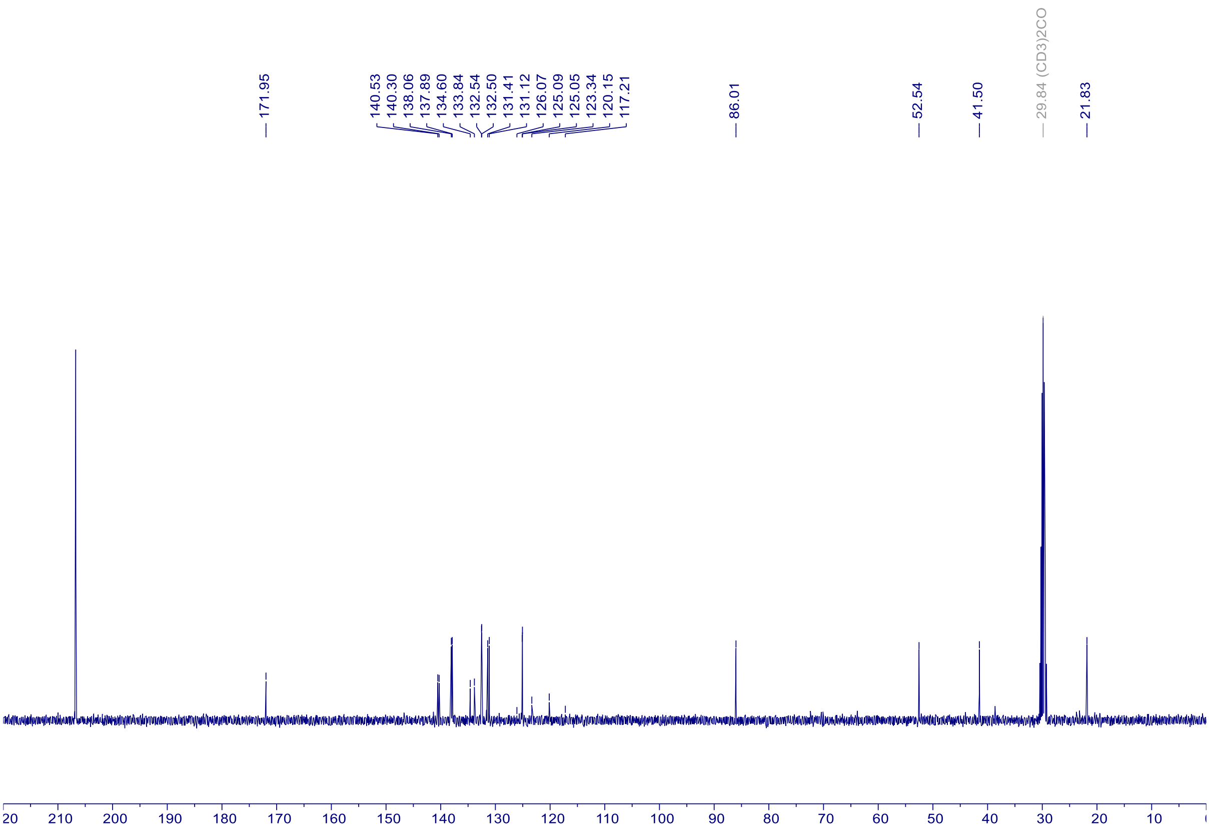

1aa' – ^{19}F NMR (376 MHz, acetone-d_6)

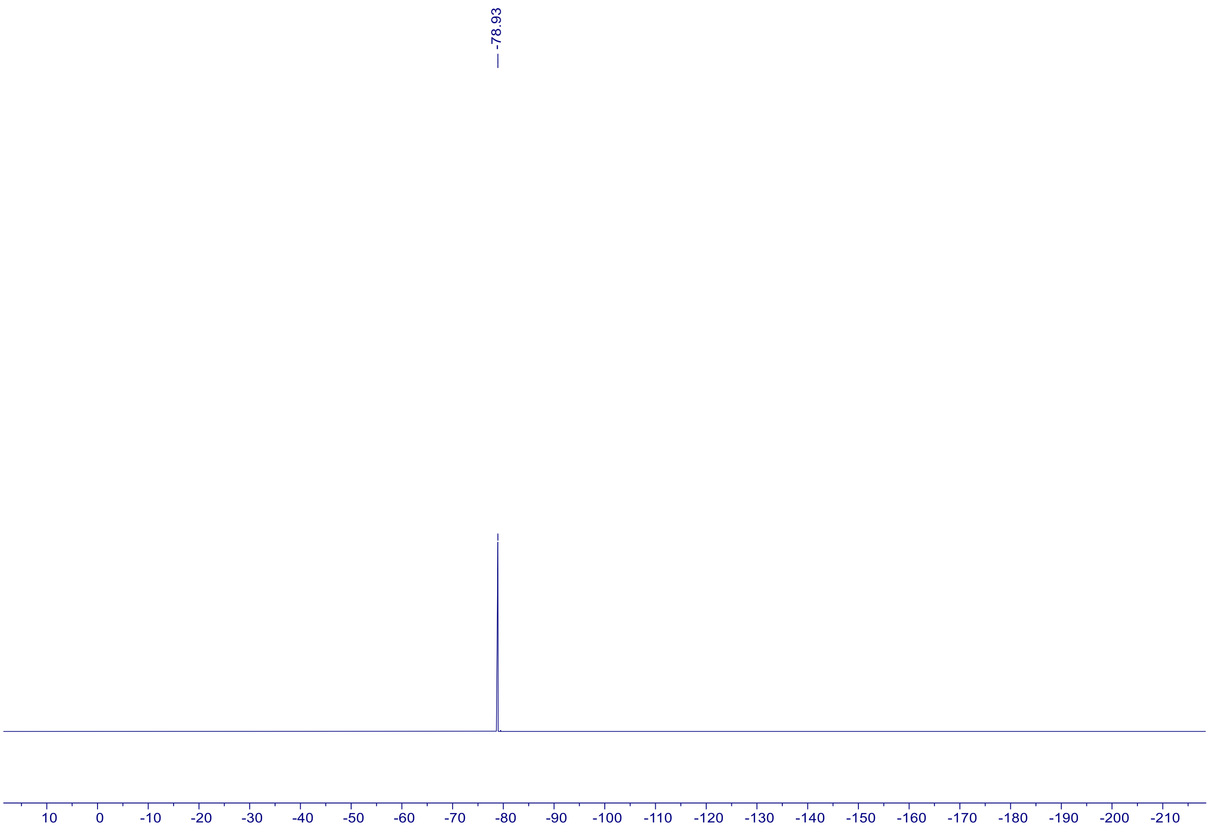

1ab – ^{1}H NMR (500 MHz, acetone-d_6)

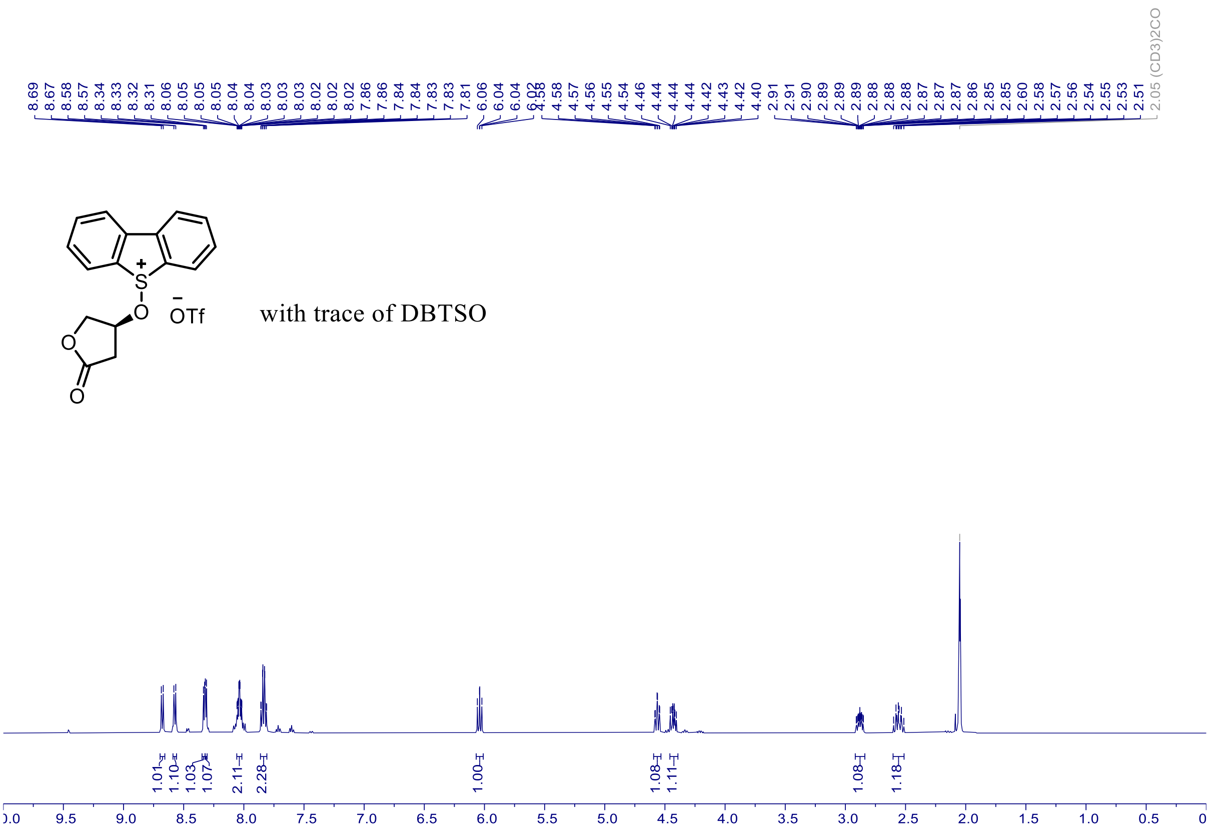

with trace of DBTSO

1ab – ^{13}C NMR (126 MHz, acetone-d_6)

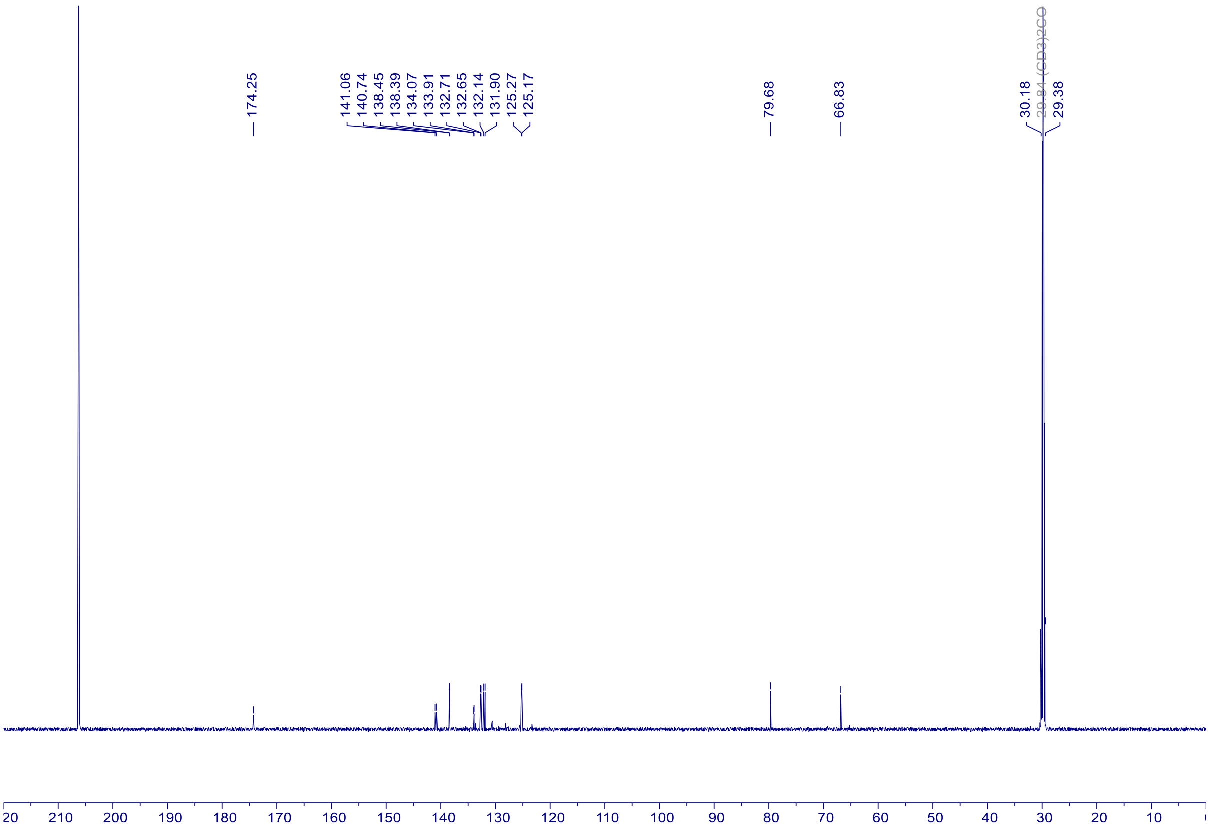

1ab – ^{19}F NMR (471 MHz, acetone-d_6)

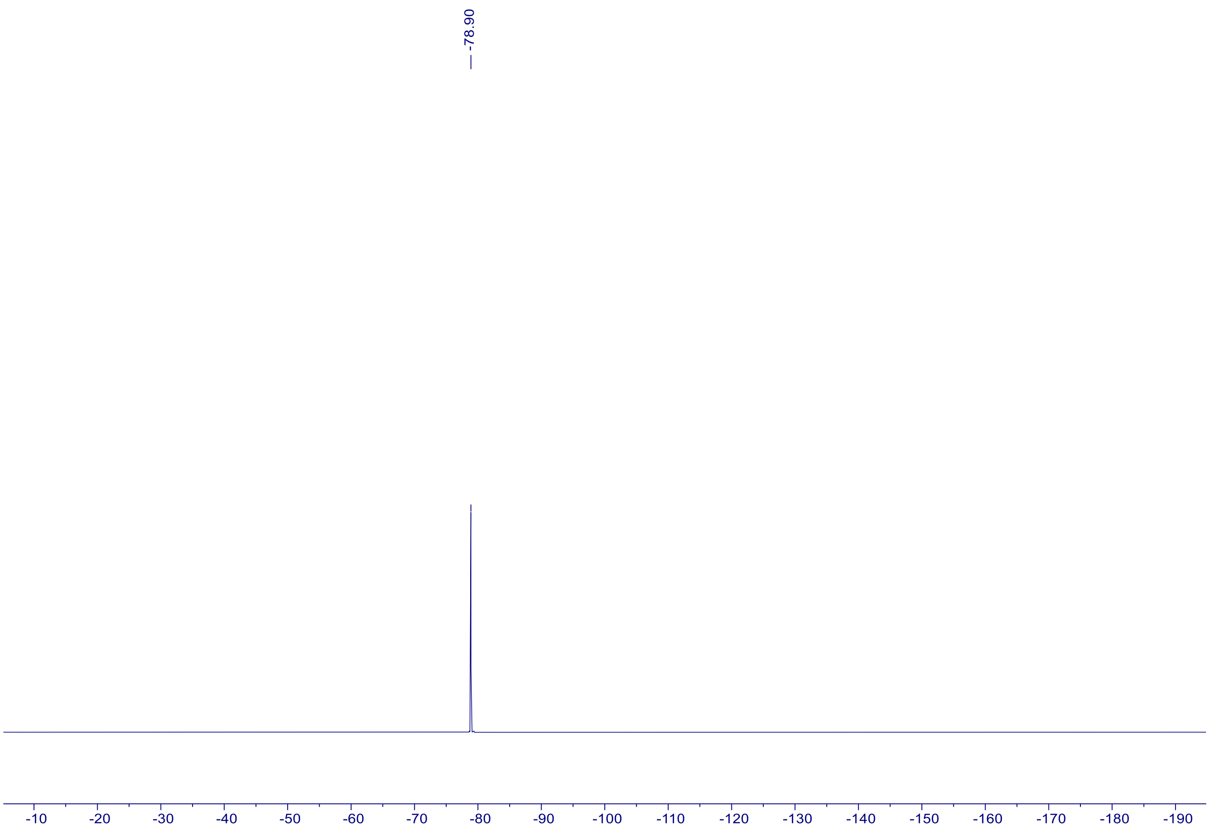

1ac – ^{1}H NMR (500 MHz, CDCl$_3$)

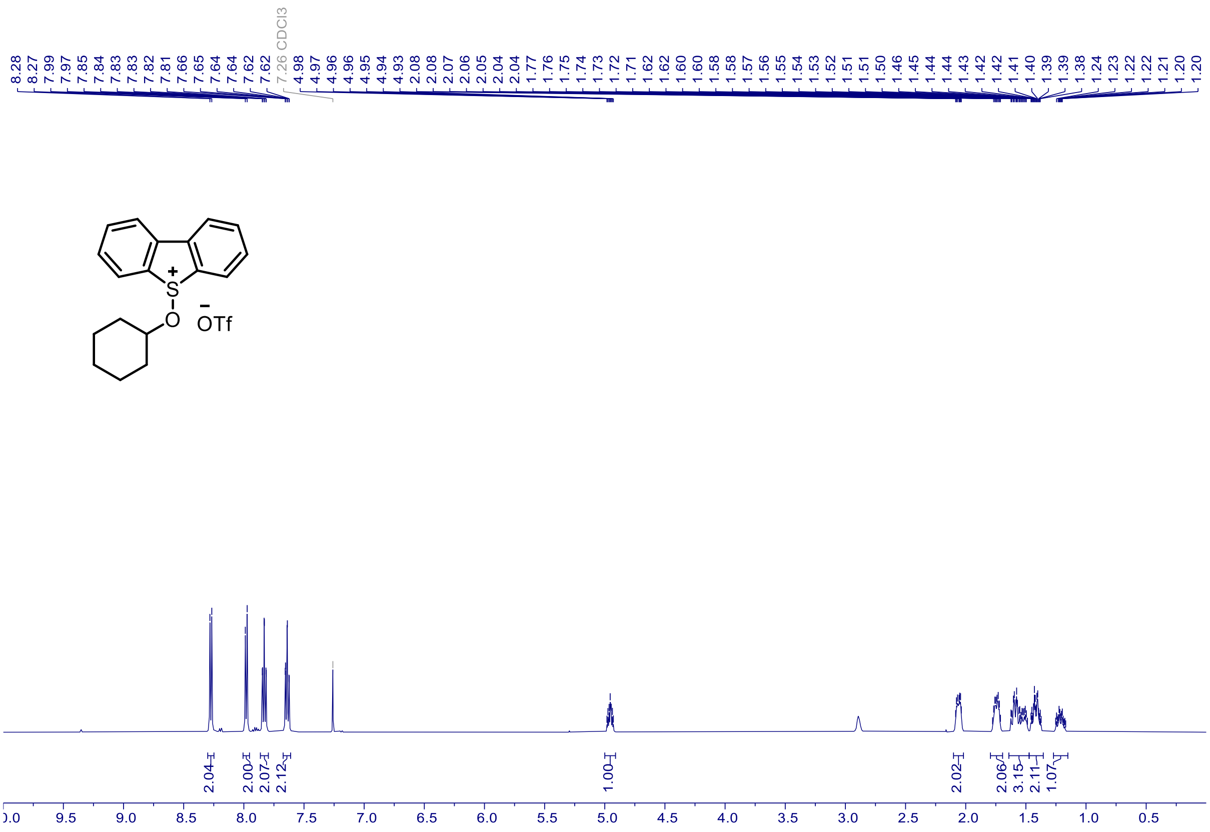

1ac – ^{13}C NMR (126 MHz, $CDCl_3$)

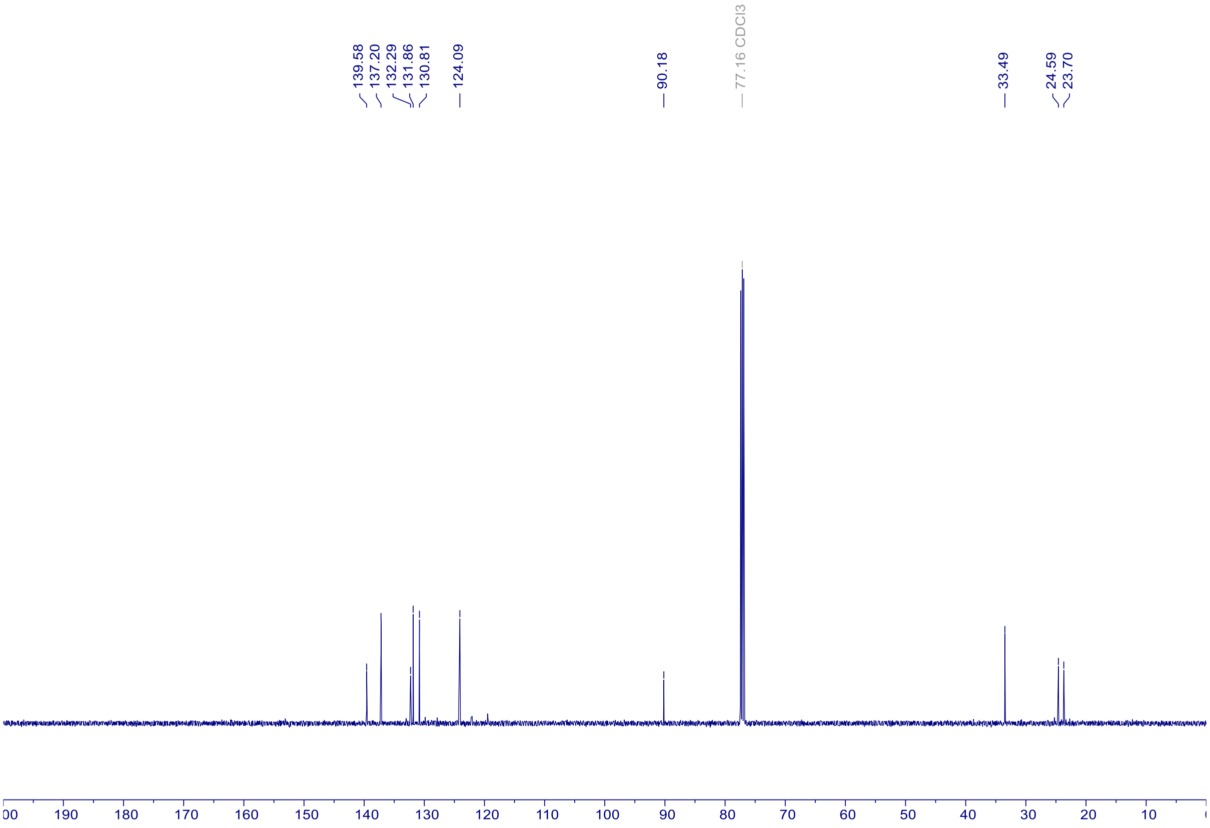

1ac – ^{19}F NMR (471 MHz, $CDCl_3$)

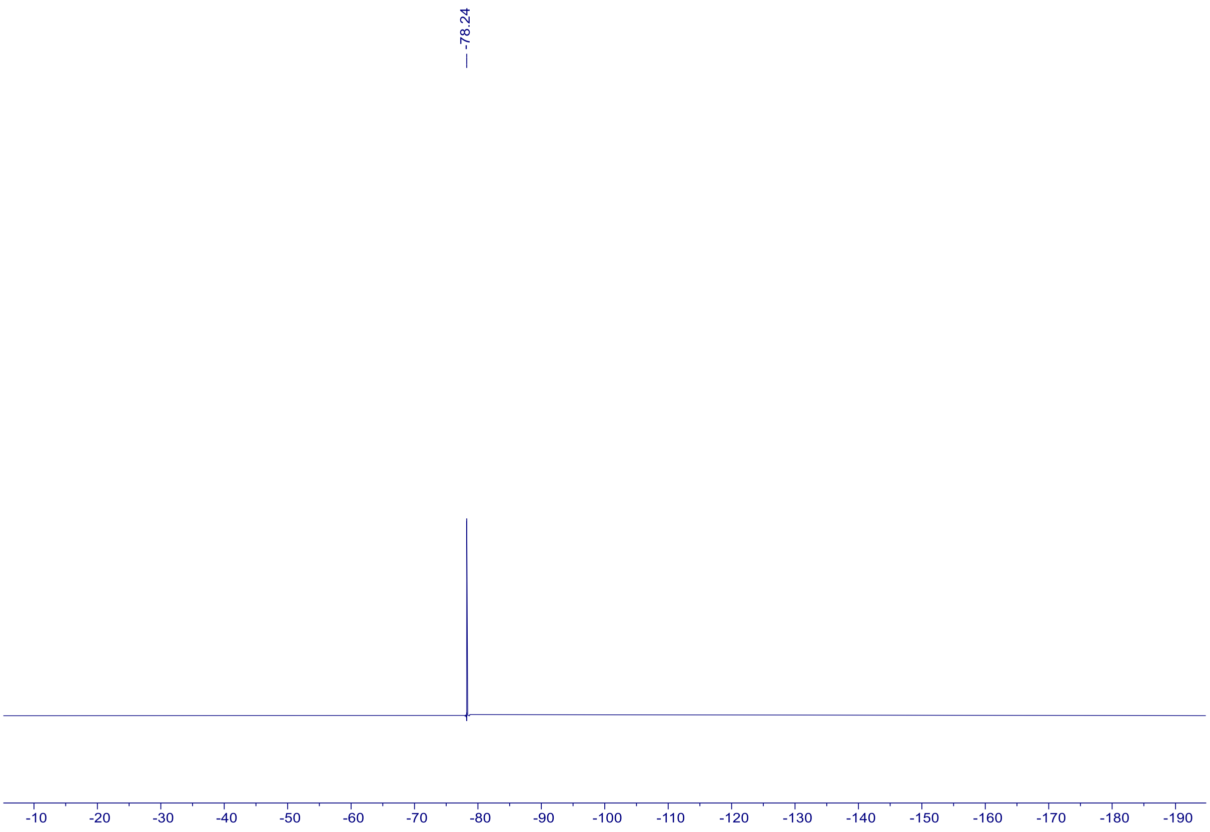

1ad – ^{1}H NMR (500 MHz, acetone-d_6)

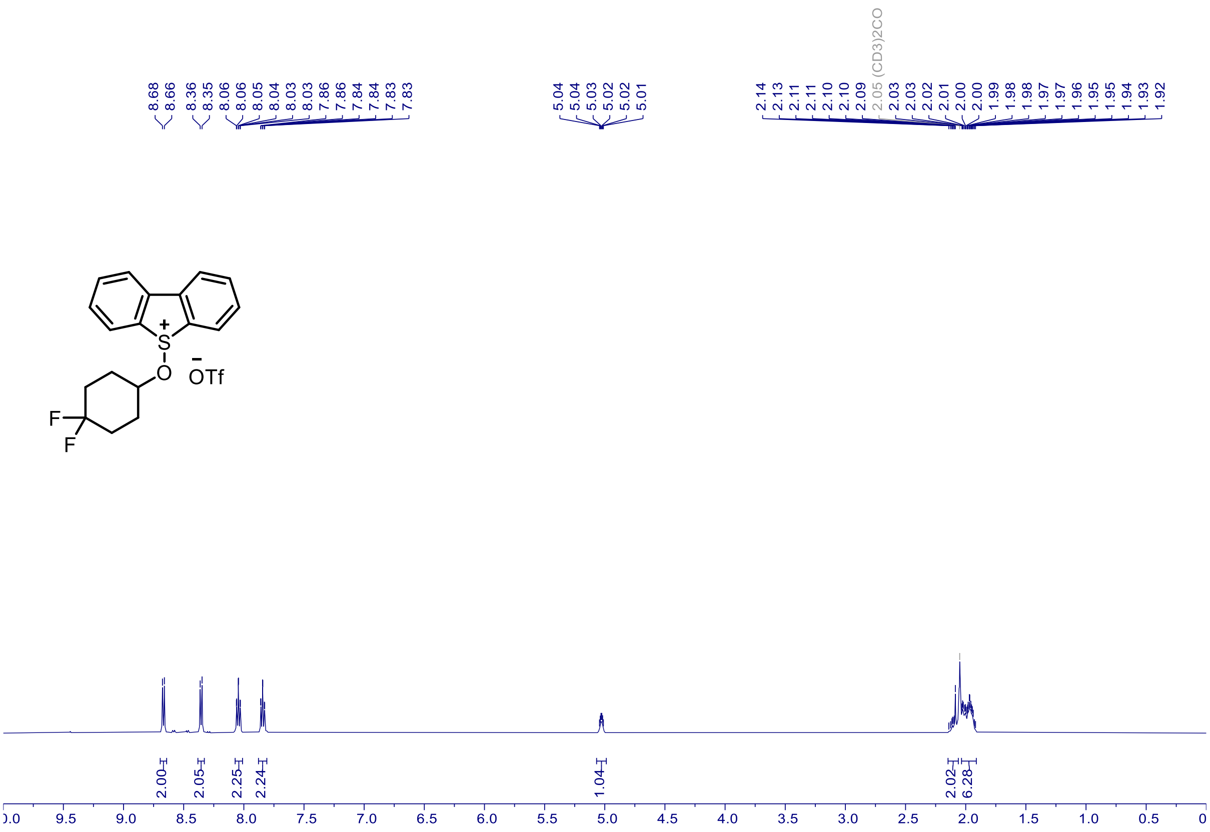

1ad – ^{13}C NMR (126 MHz, acetone-d_6)

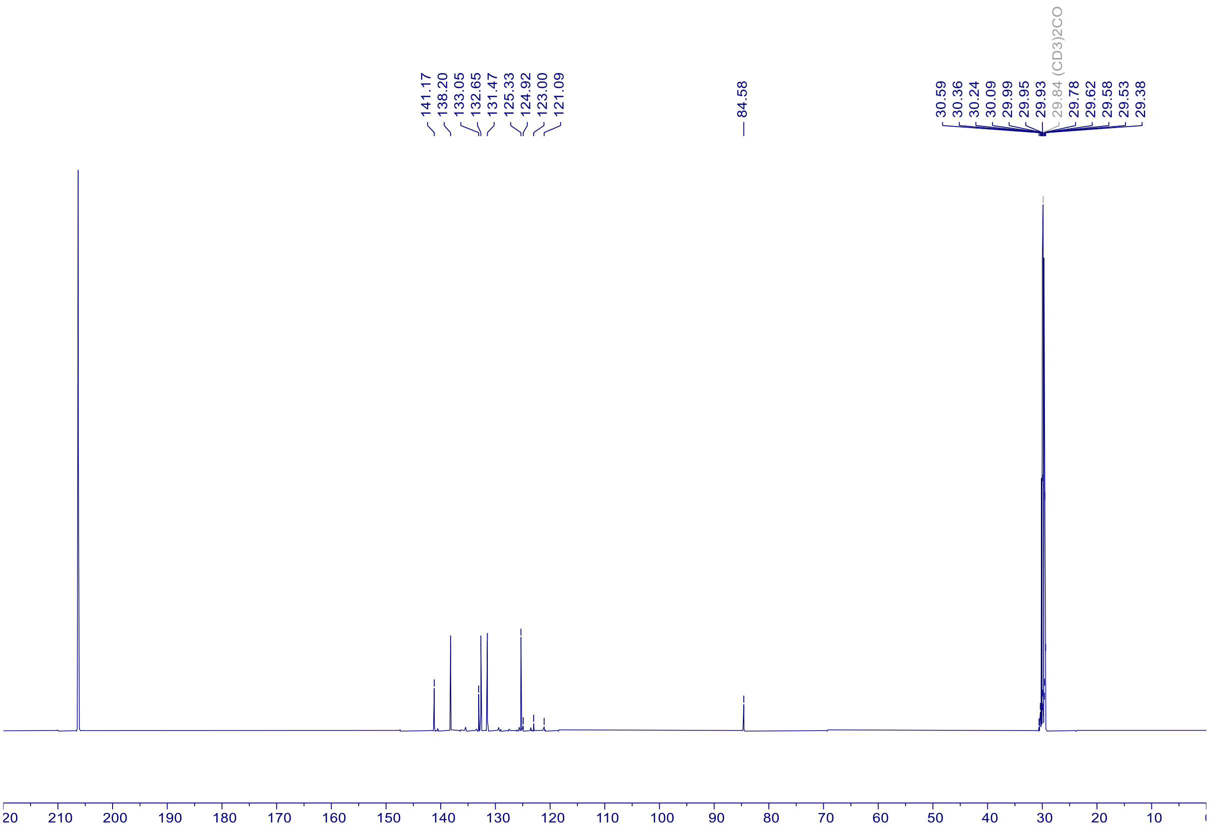

1ad – ^{19}F NMR (471 MHz, acetone-d_6)

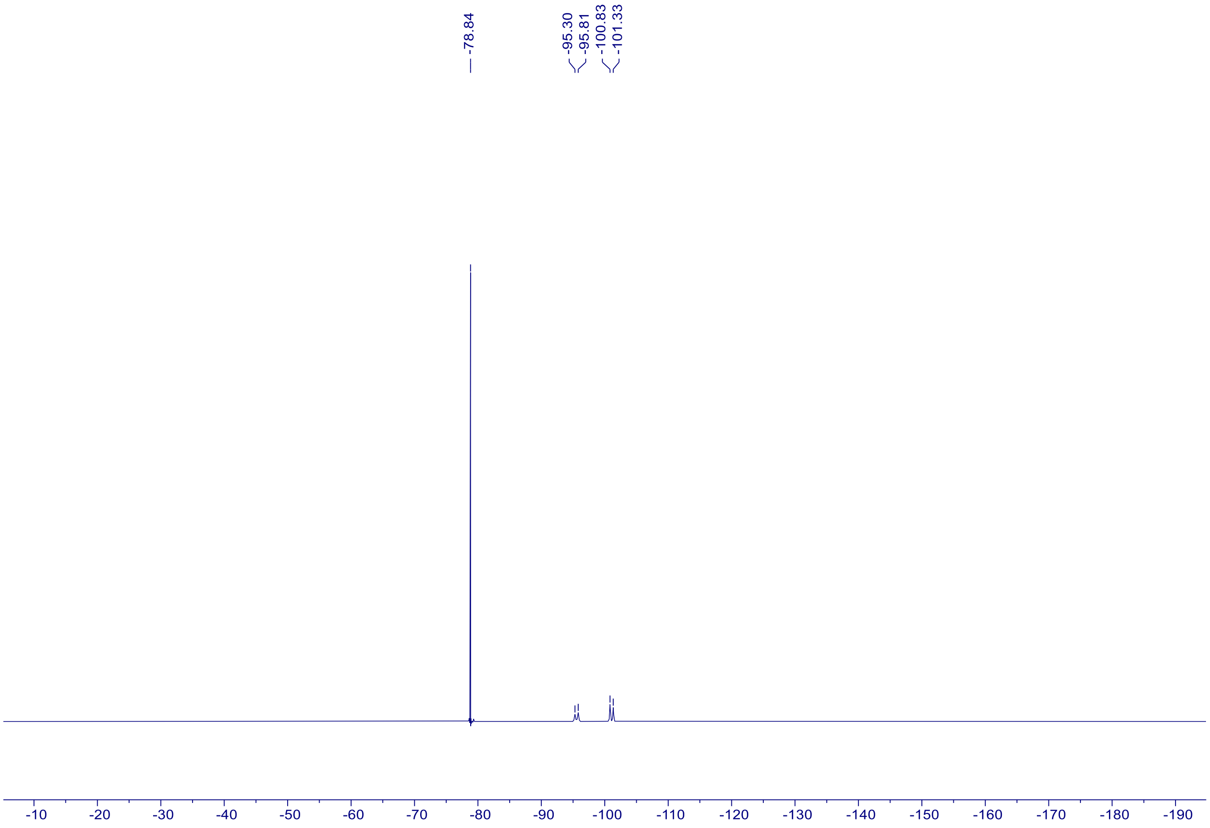

1ae – ^{1}H NMR (400 MHz, acetone-d_6)

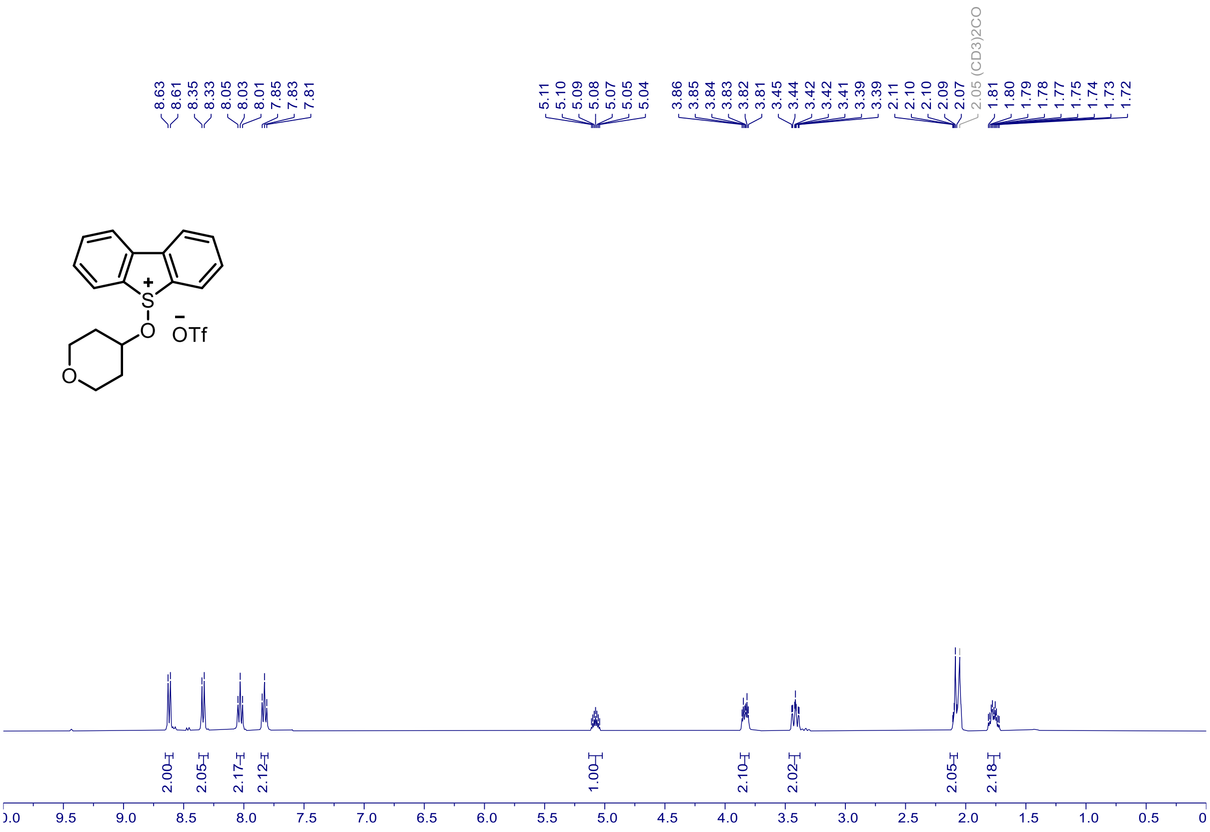

1ae – ^{13}C NMR (101 MHz, acetone-d_6)

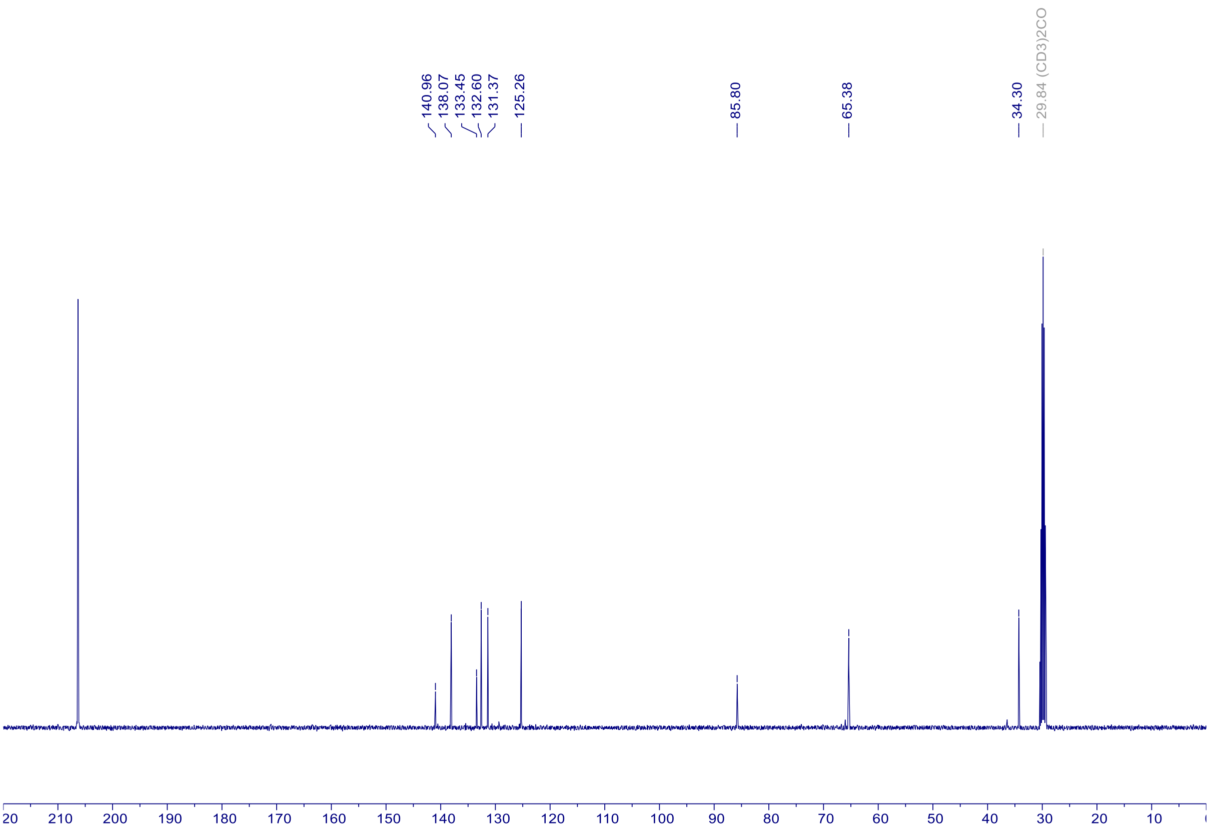

1ae – ^{19}F NMR (376 MHz, acetone-d_6)

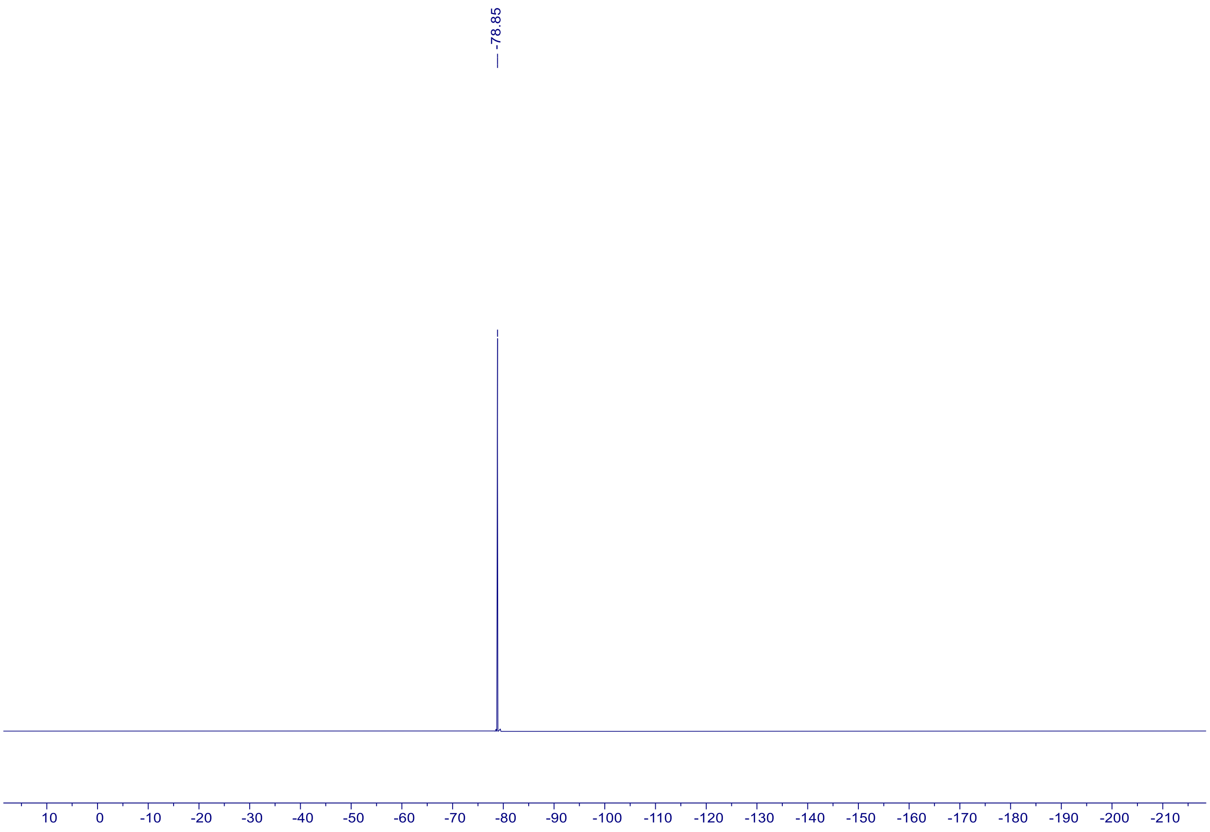

1af – ^{1}H NMR (400 MHz, acetone-d_6)

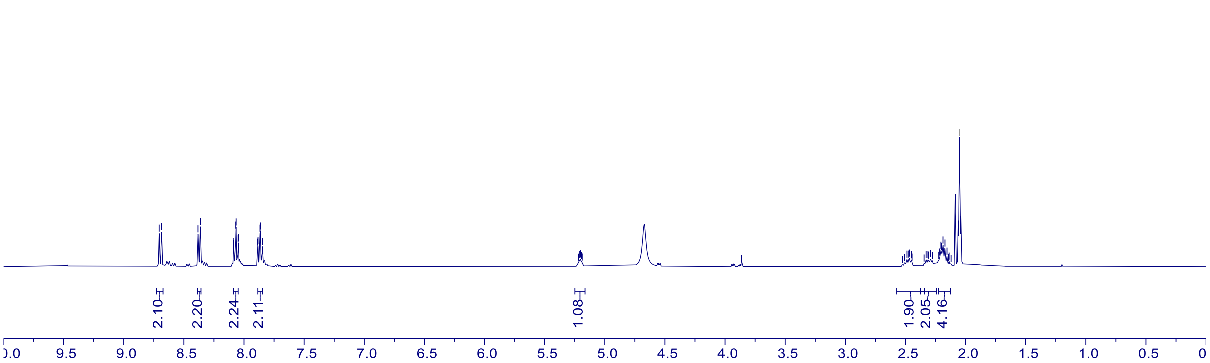

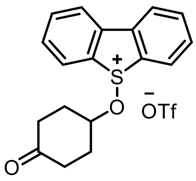

with trace of DBTSO

1af – ^{13}C NMR (101 MHz, acetone-d_6)

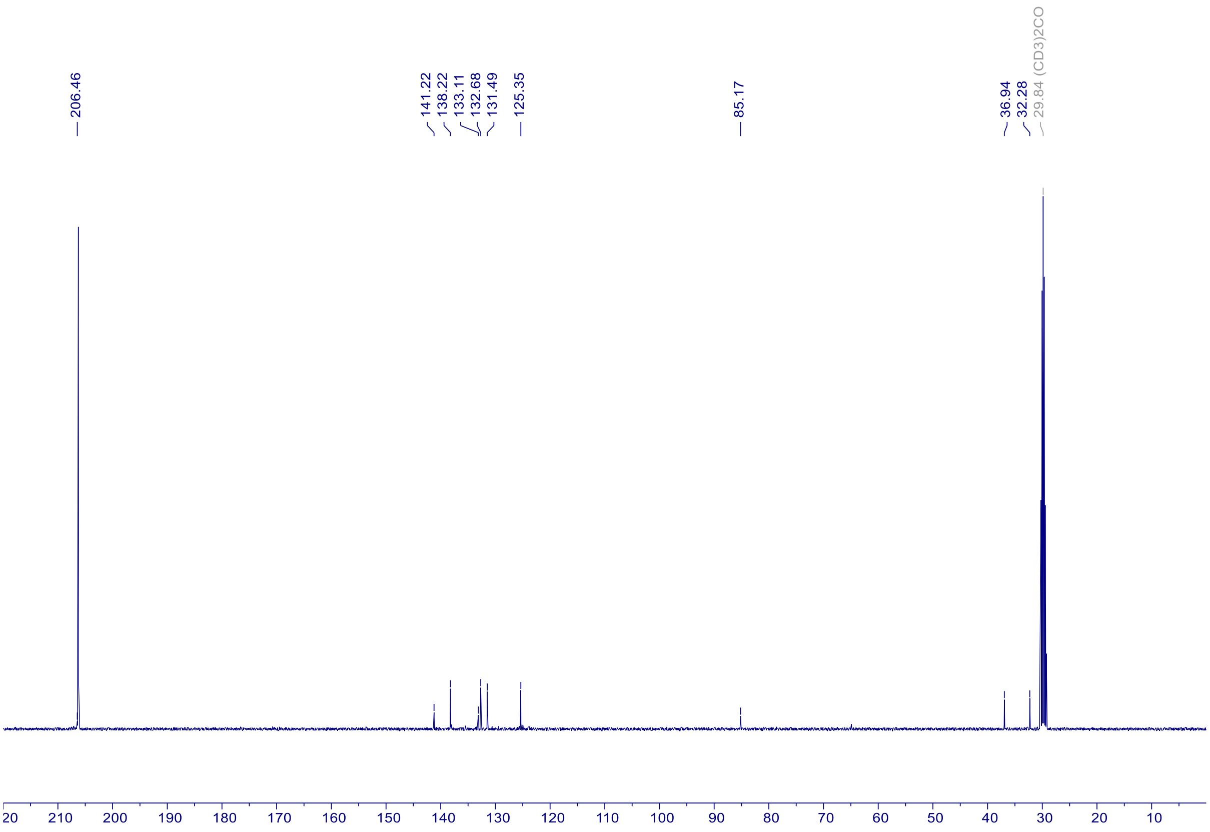

1af – ^{19}F NMR (376 MHz, acetone-*d*$_6$)

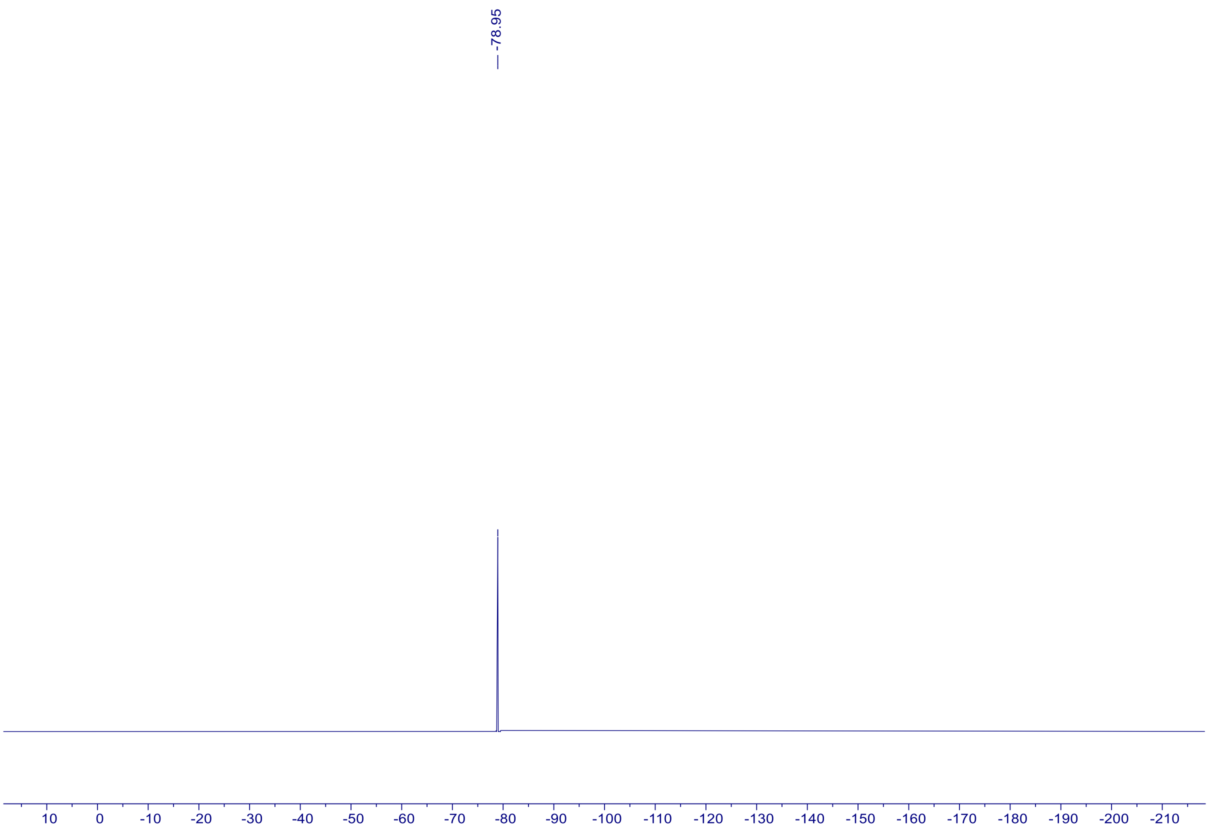

1ag – ^{1}H NMR (400 MHz, acetone-*d*$_6$)

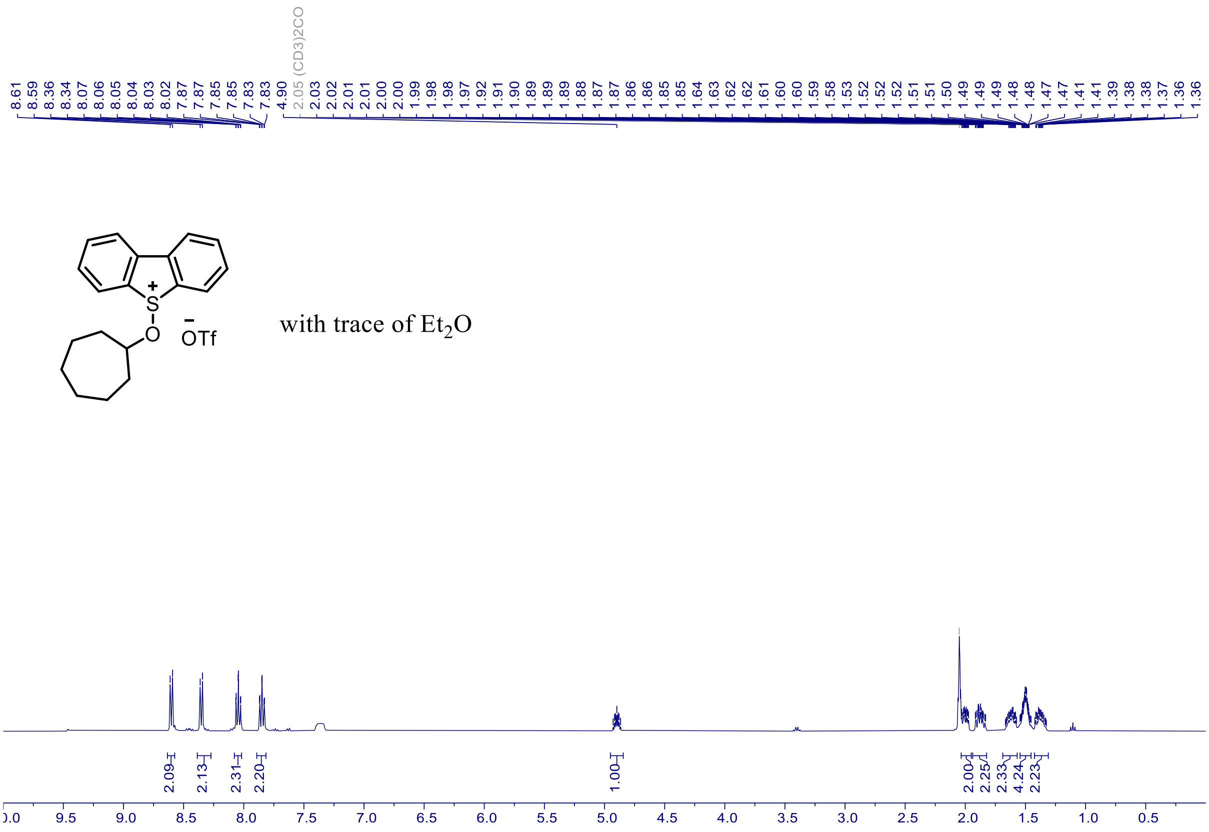

with trace of Et_2O

1ag – ^{13}C NMR (101 MHz, acetone-d_6)

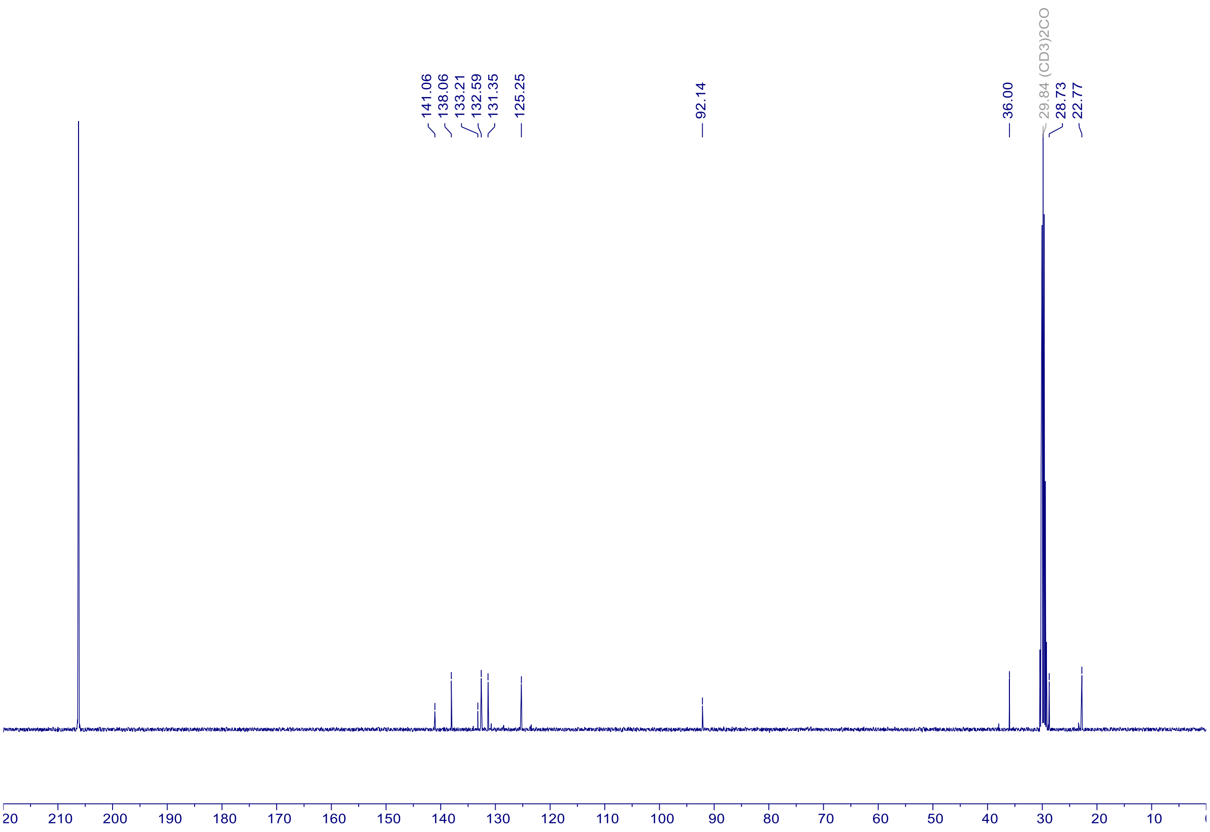

1ag – ^{19}F NMR (376 MHz, acetone-d_6)

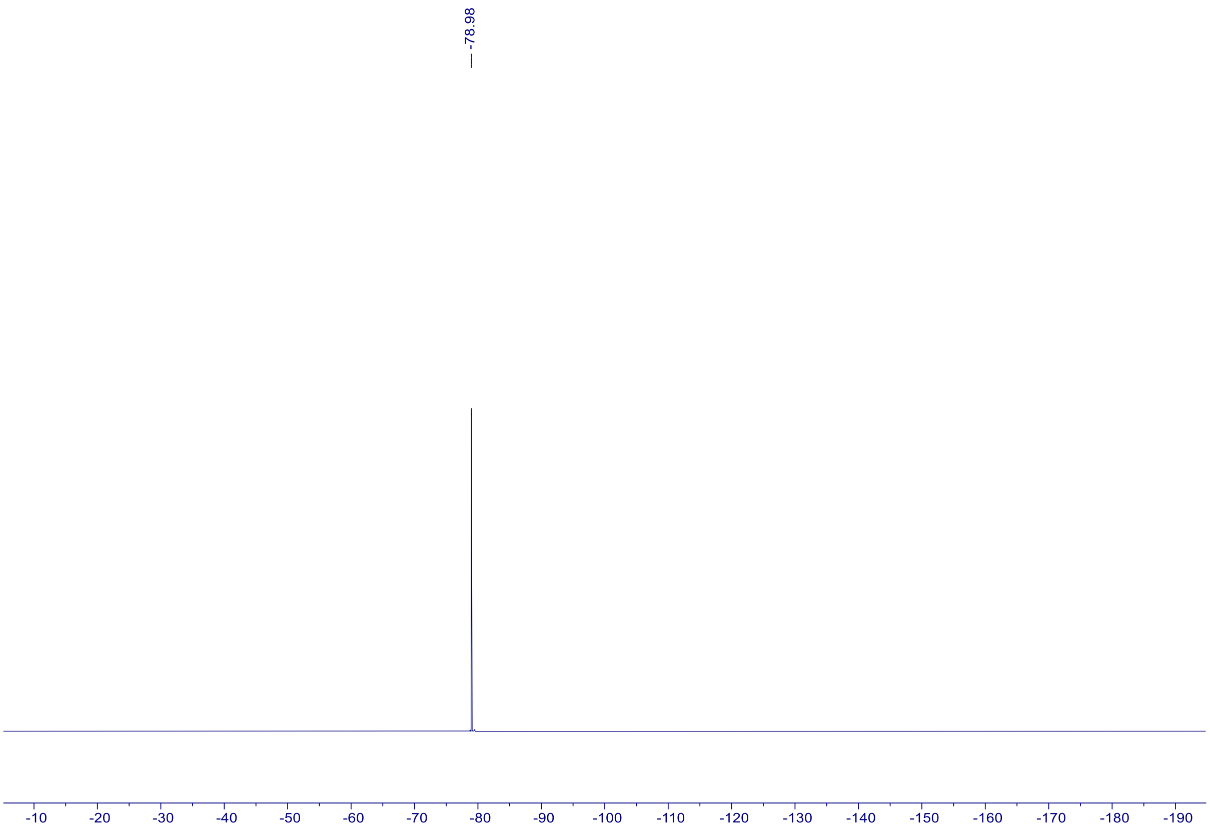

1ah – ^{1}H NMR (500 MHz, acetone-d_6)

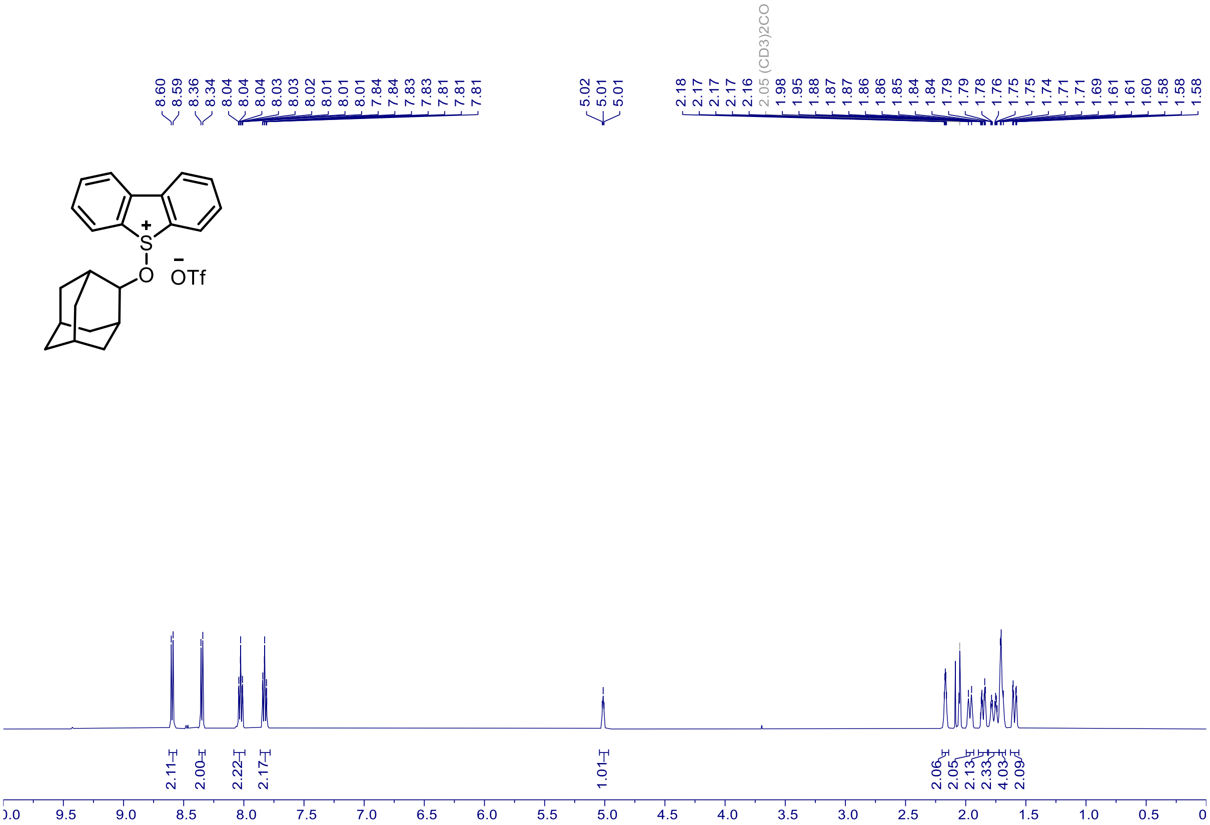

1ah – ^{13}C NMR (126 MHz, acetone-d_6)

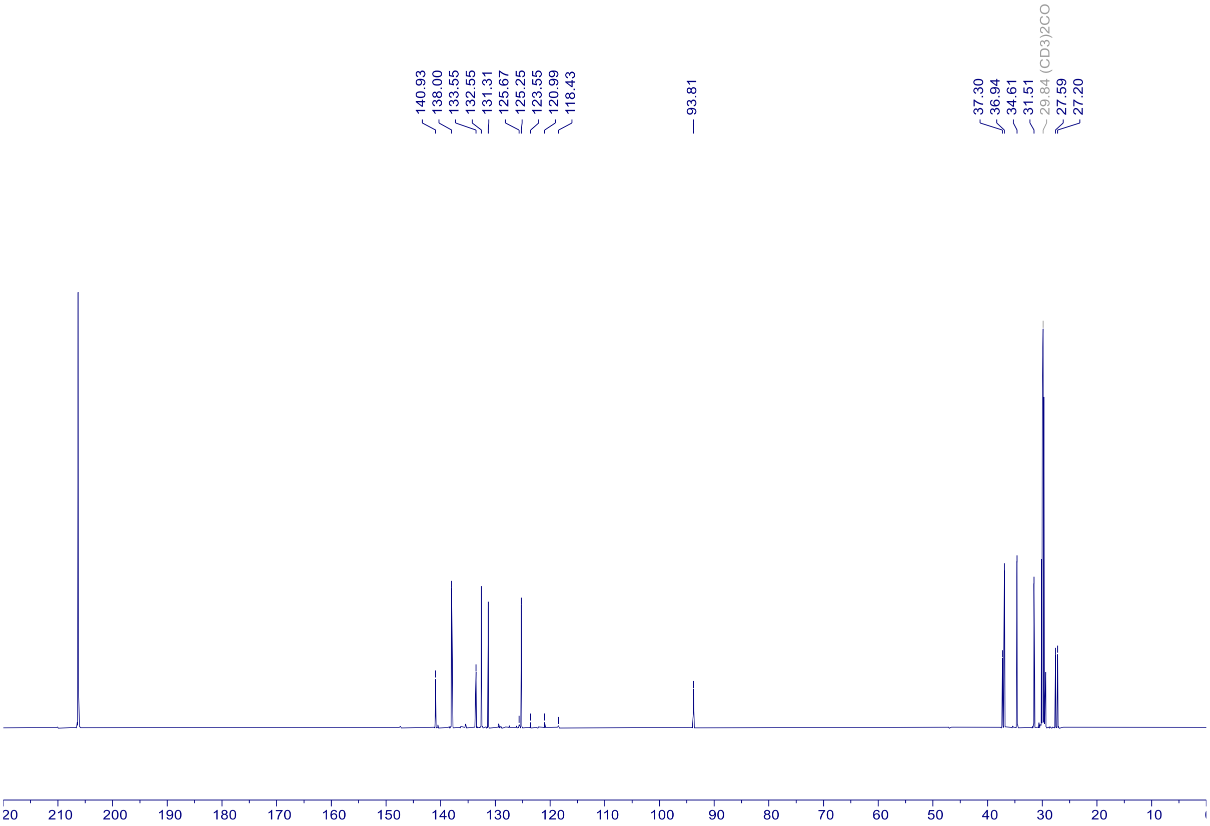

1ah – ^{19}F NMR (471 MHz, acetone-d_6)

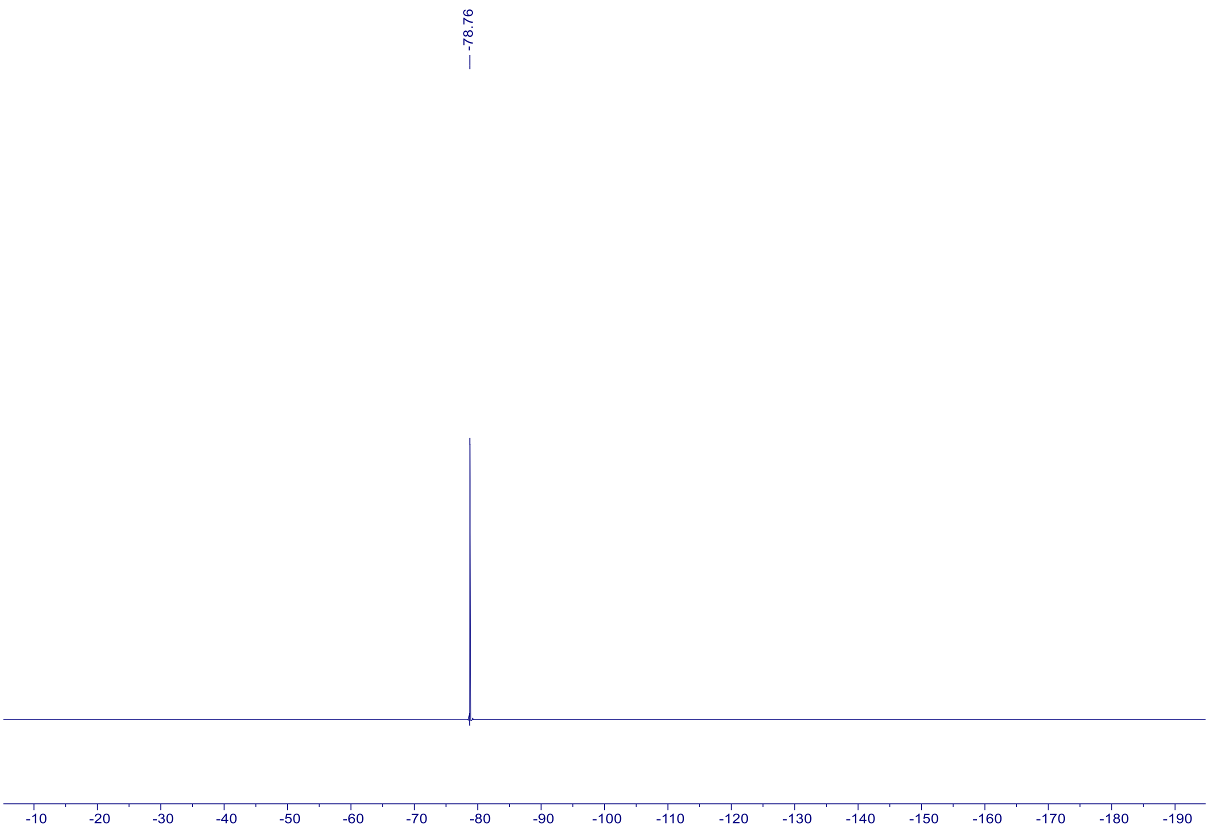

1ai – ^{1}H NMR (400 MHz, acetone-d_6)

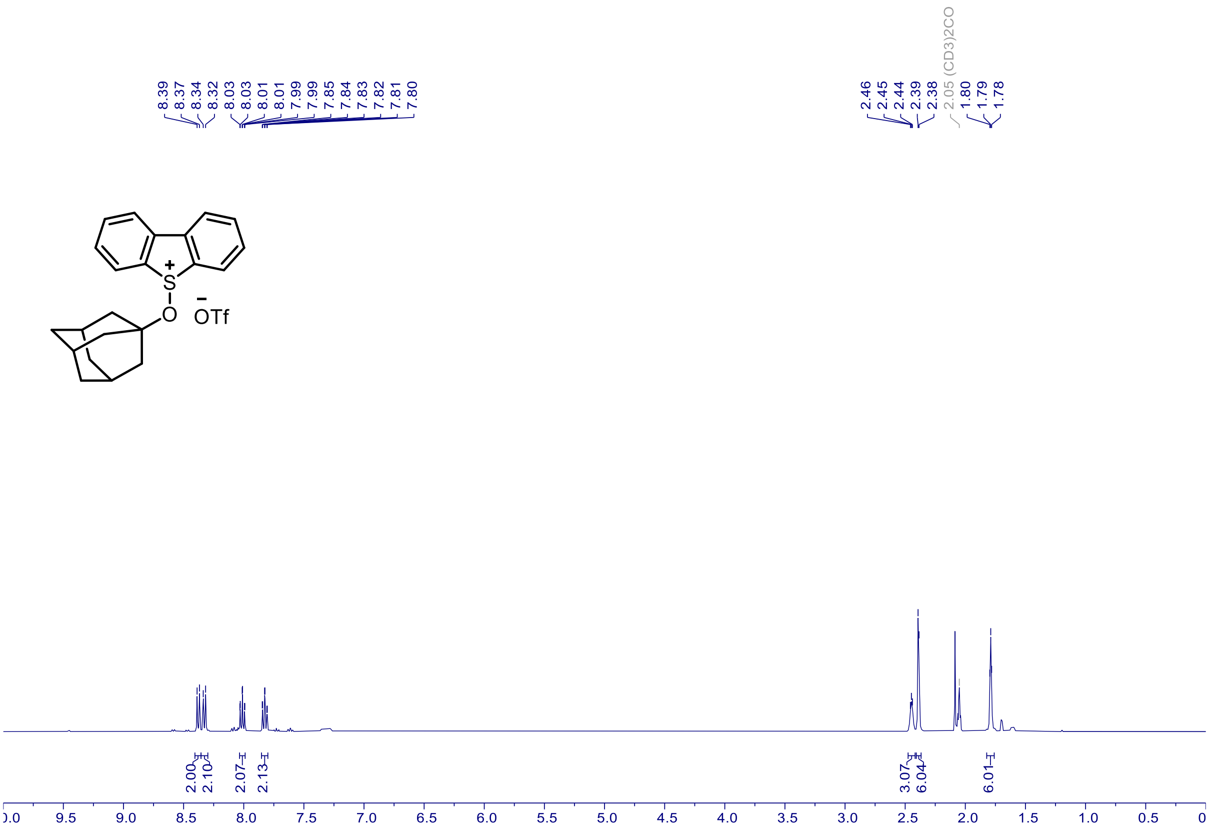

1ai – ^{13}C NMR (126 MHz, acetone-d_6)

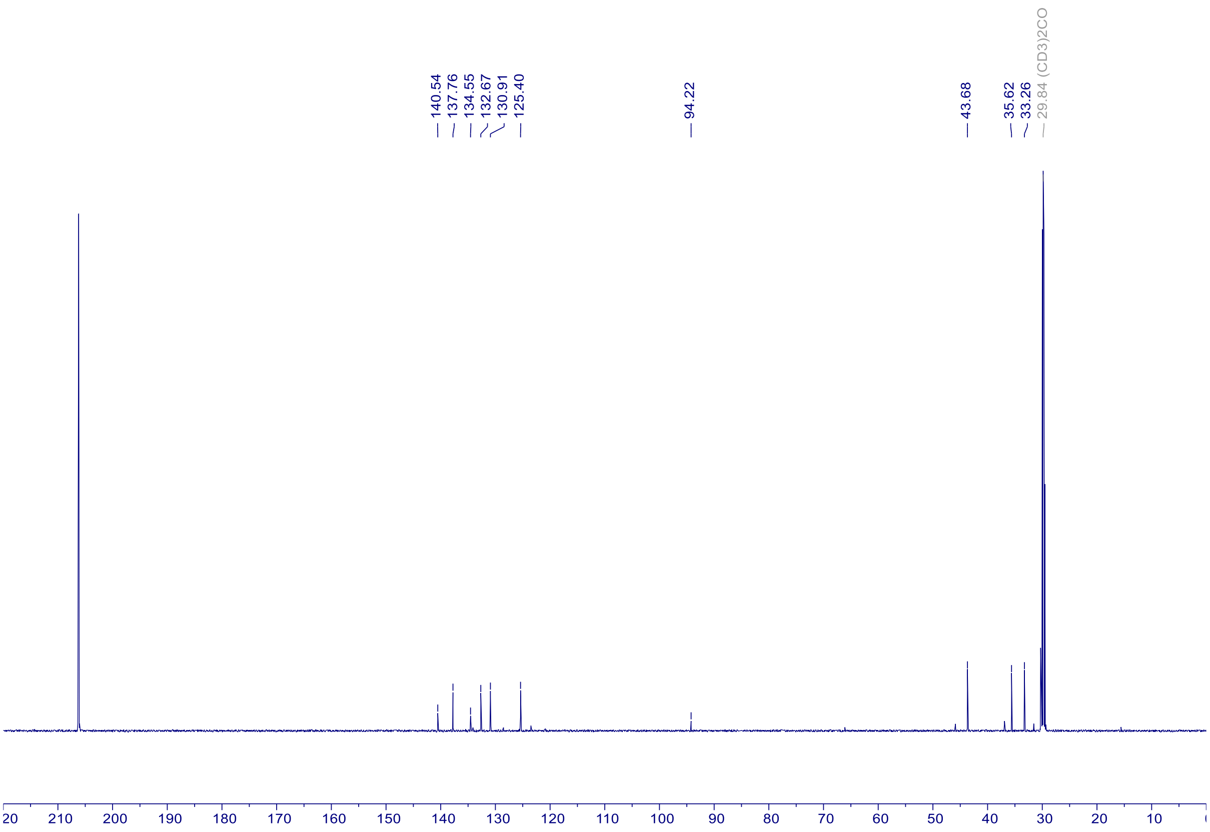

1ai – ^{19}F NMR (471 MHz, acetone-d_6)

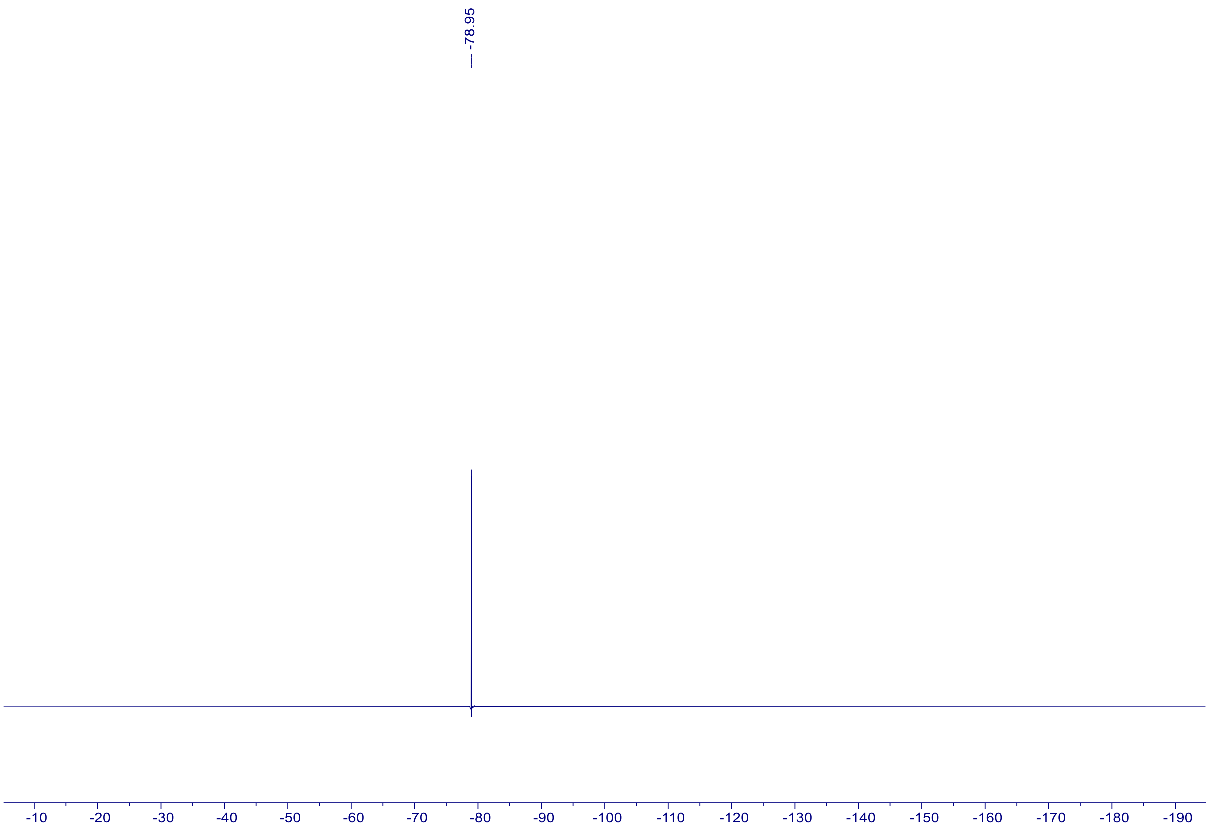

1aj – ^{1}H NMR (500 MHz, acetone-d_6)

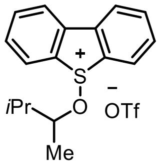

with trace of DBTSO

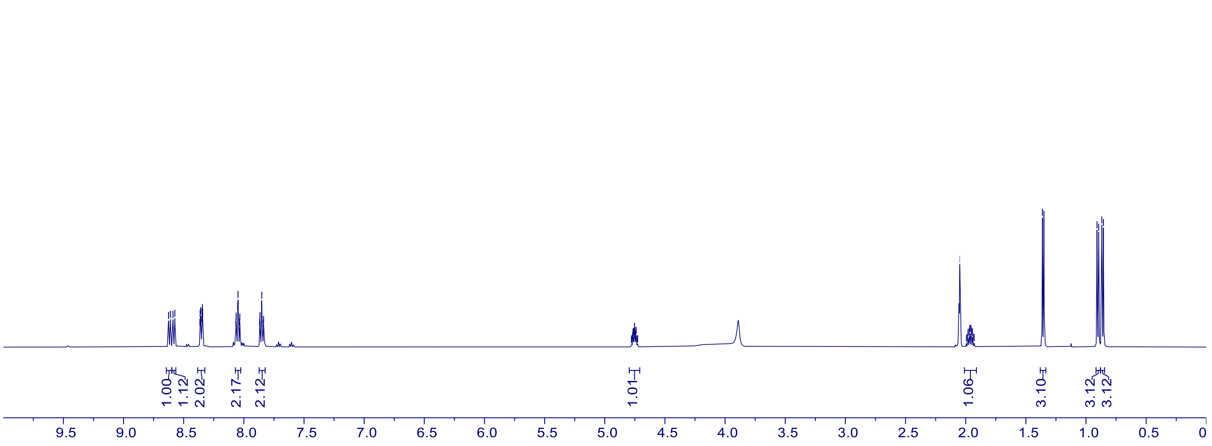

1aj – ^{13}C NMR (126 MHz, acetone-d_6)

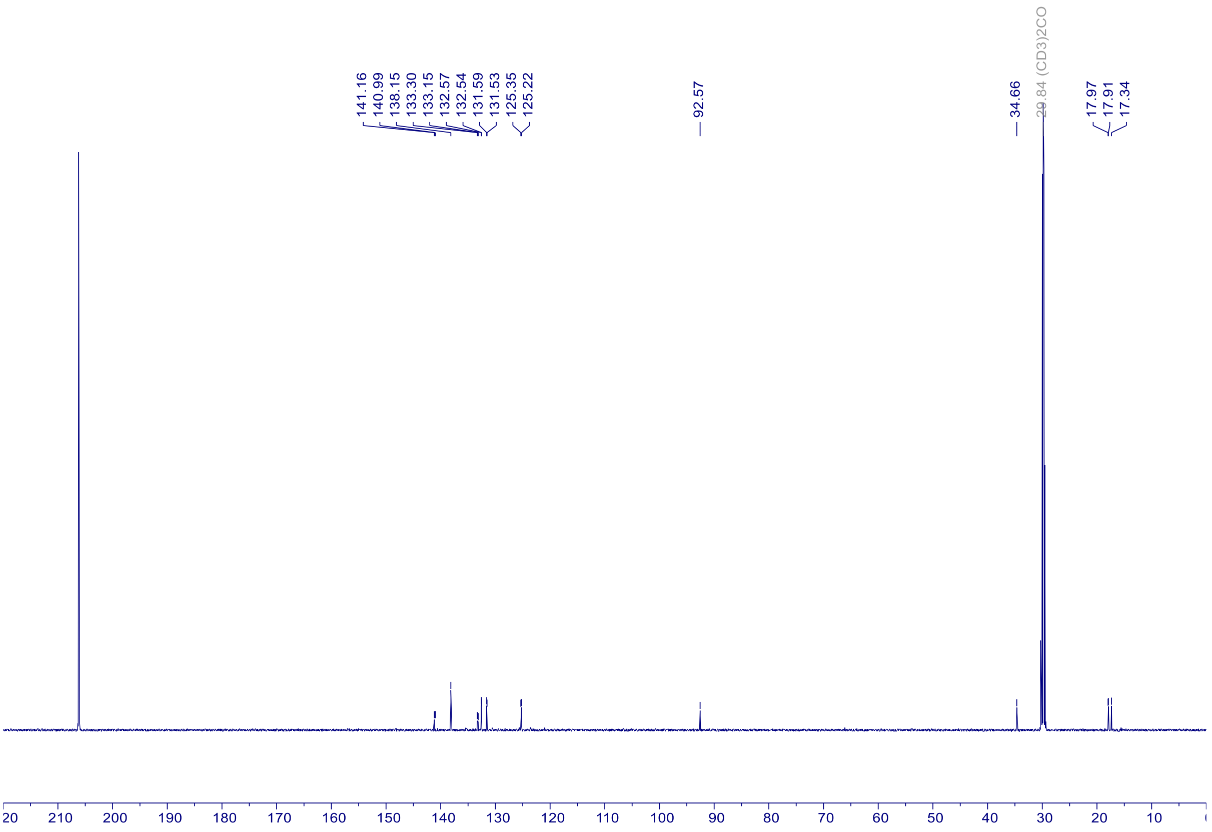

1aj – ^{19}F NMR (471 MHz, acetone-d_6)

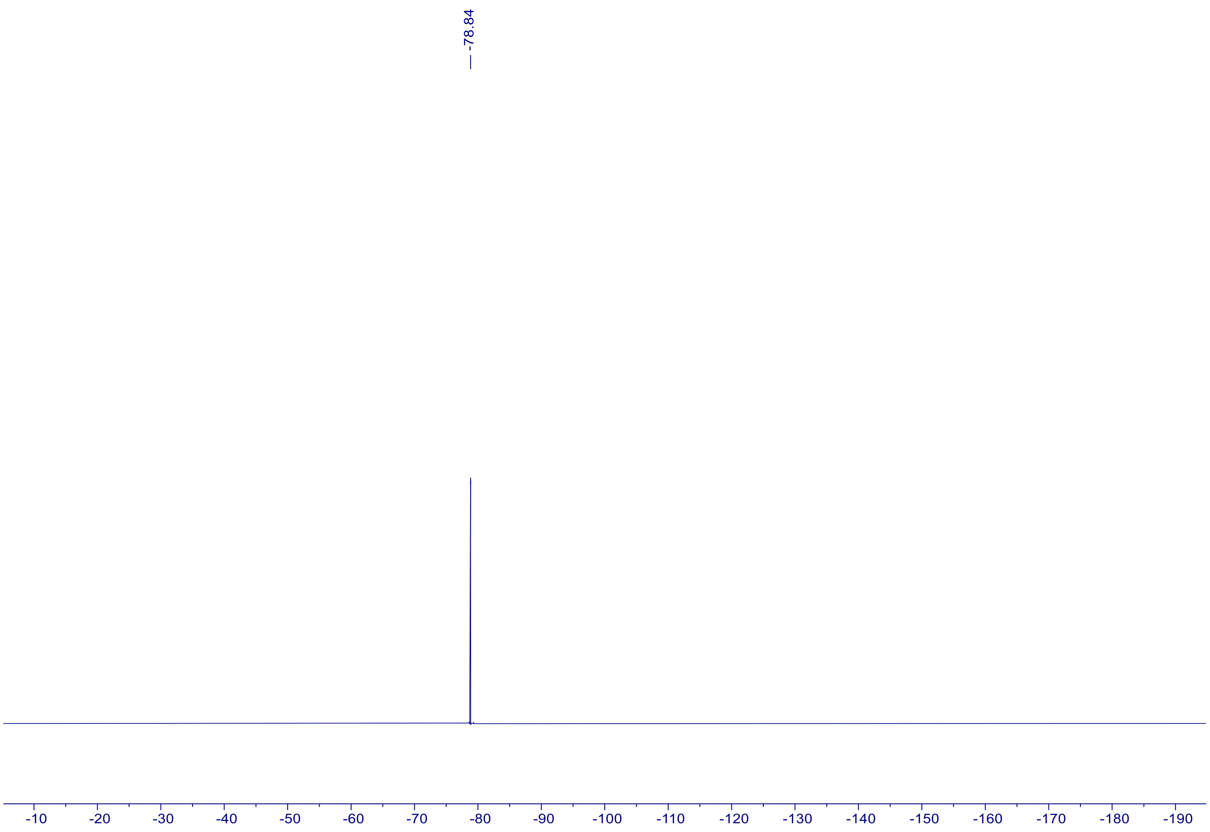

3b – ^{1}H NMR (500 MHz, $CDCl_3$)

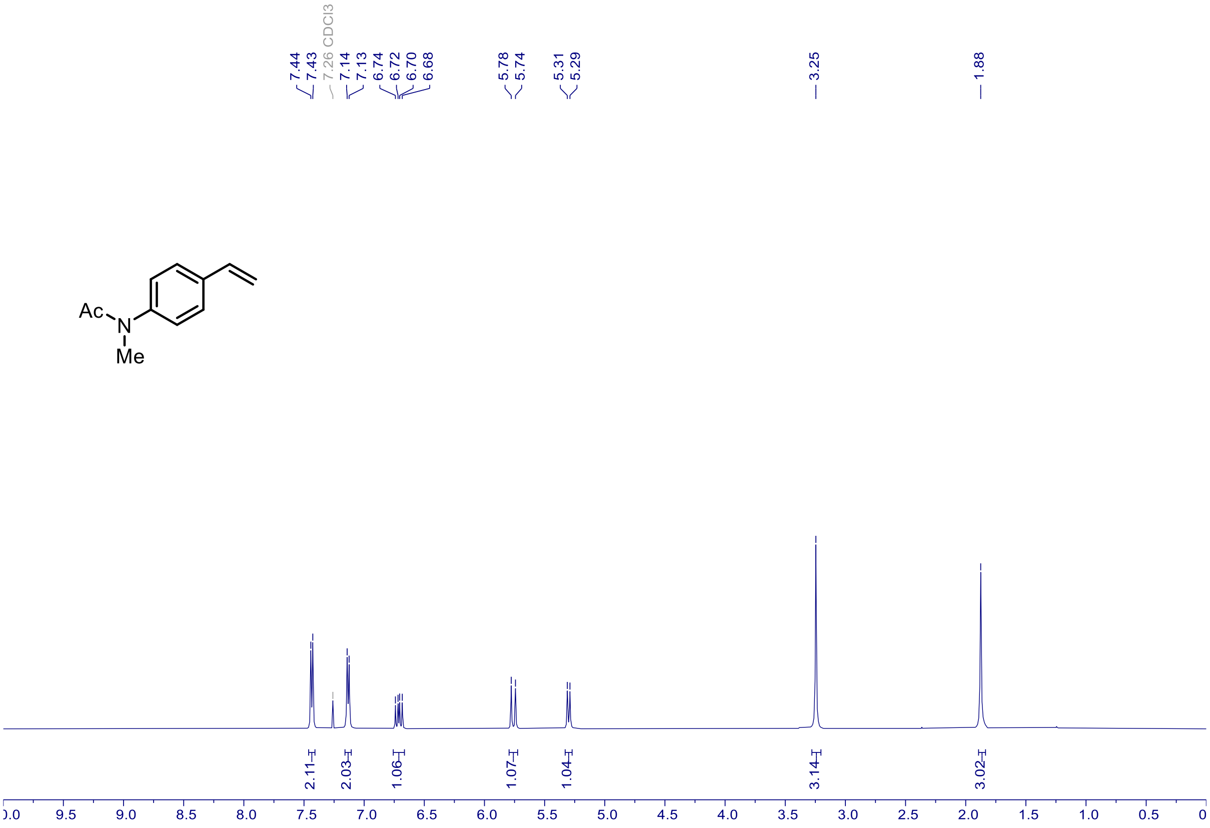

3b – ^{13}C NMR (126 MHz, $CDCl_3$)

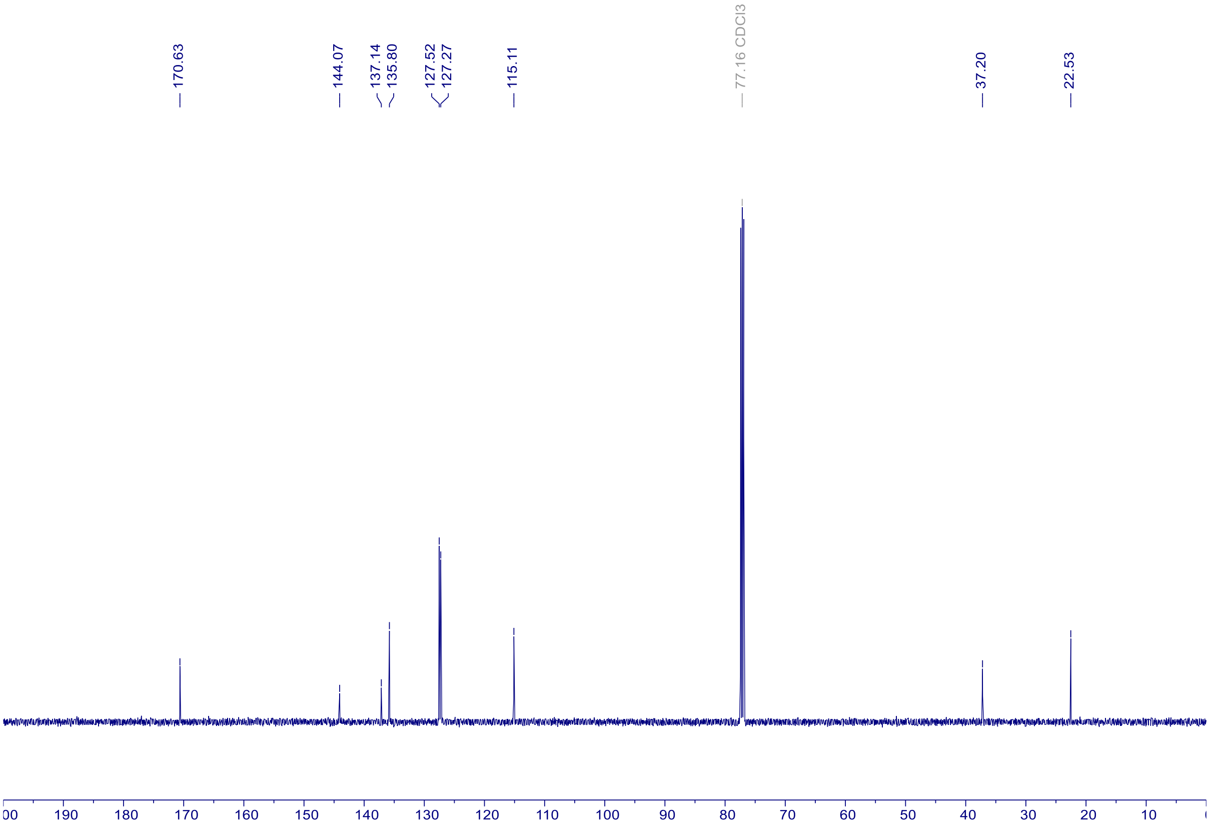

3c – ^{1}H NMR (400 MHz, $CDCl_3$)

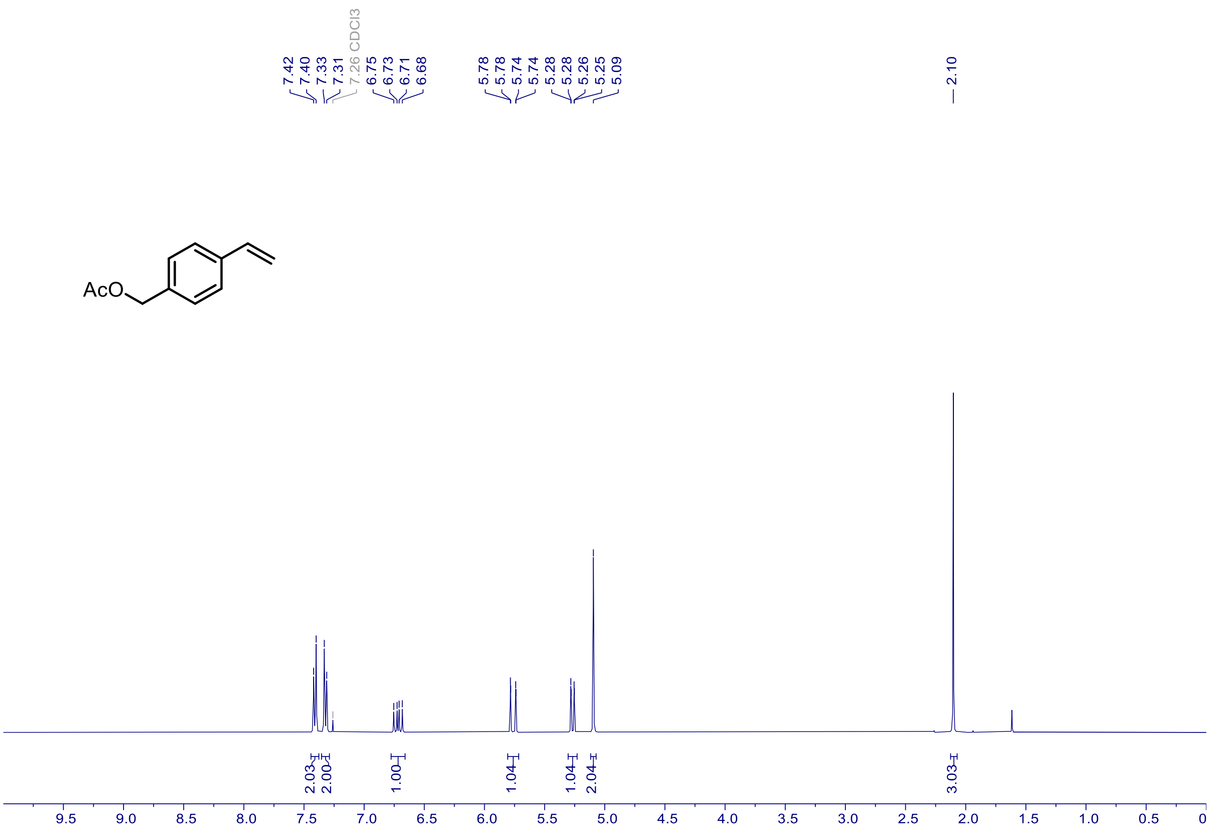

3c – ^{13}C NMR (101 MHz, $CDCl_3$)

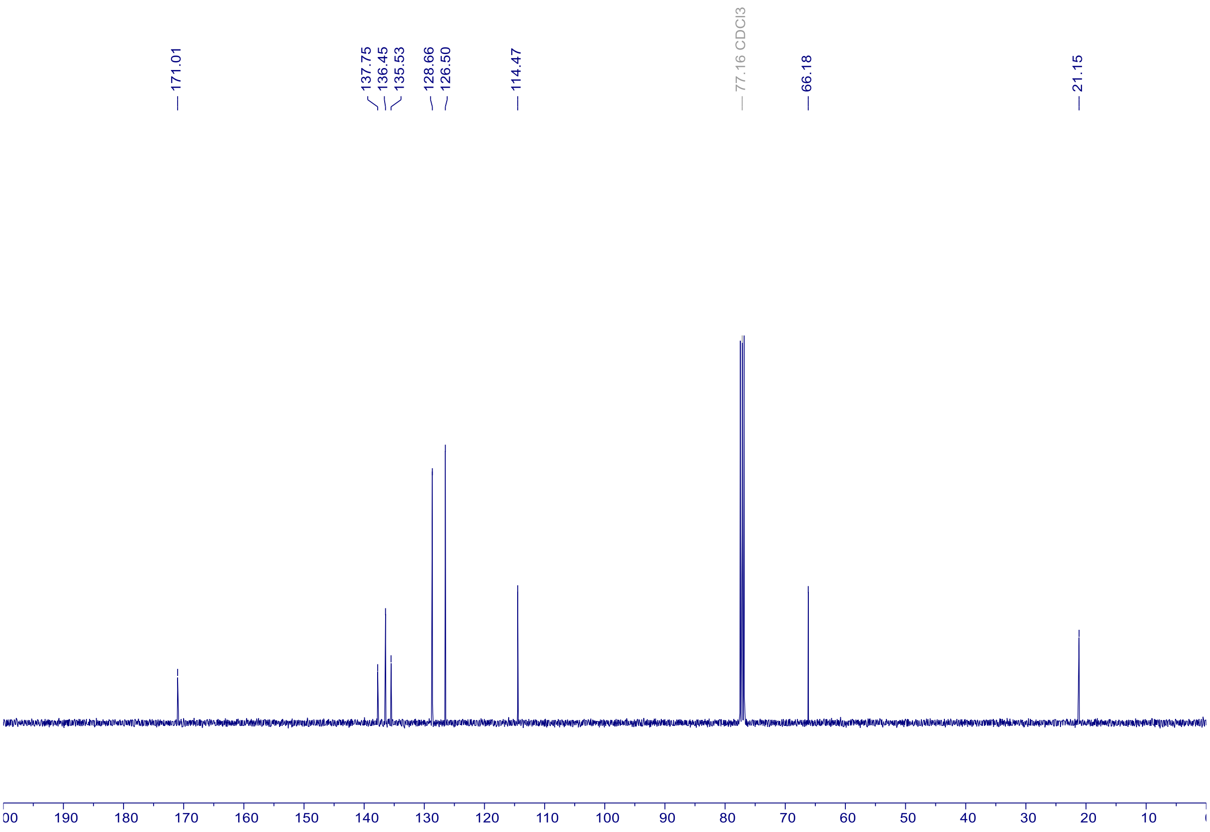

3d – ^{1}H NMR (500 MHz, CDCl$_3$)

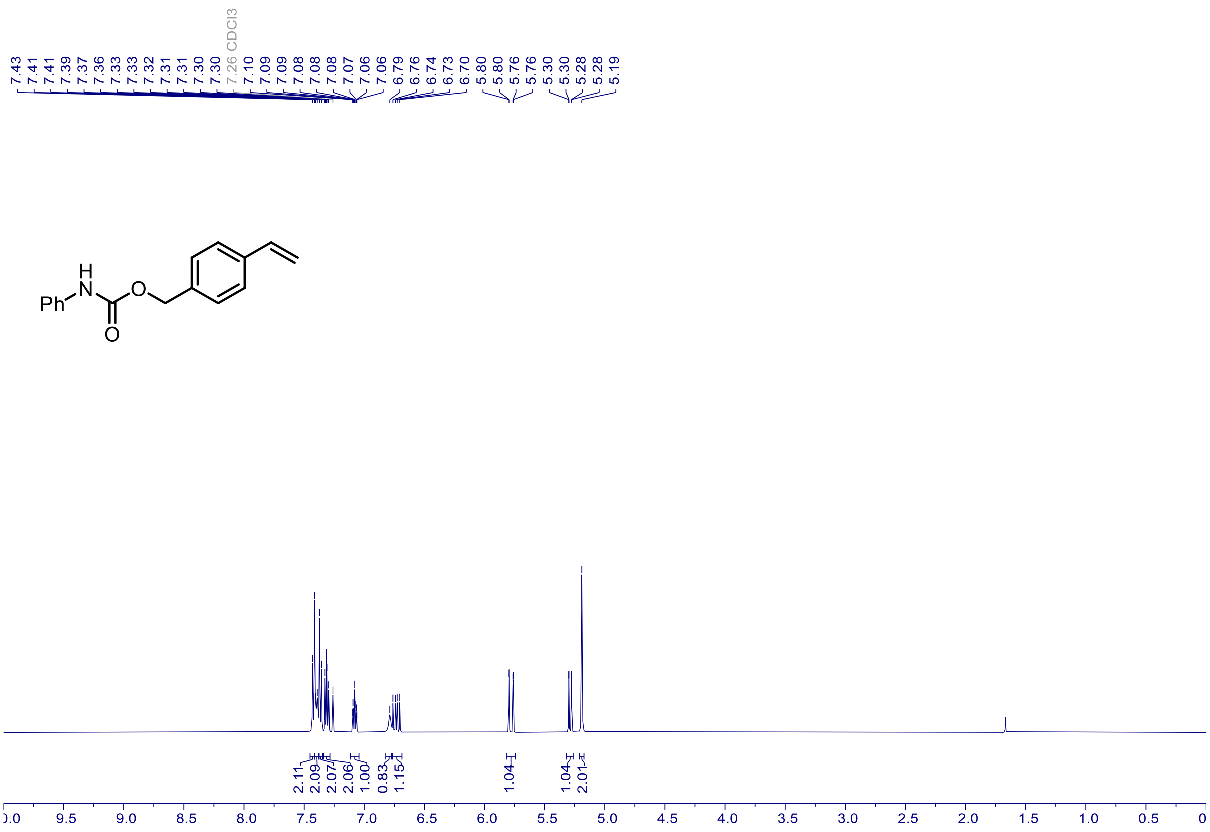

3d – ^{13}C NMR (126 MHz, CDCl$_3$)

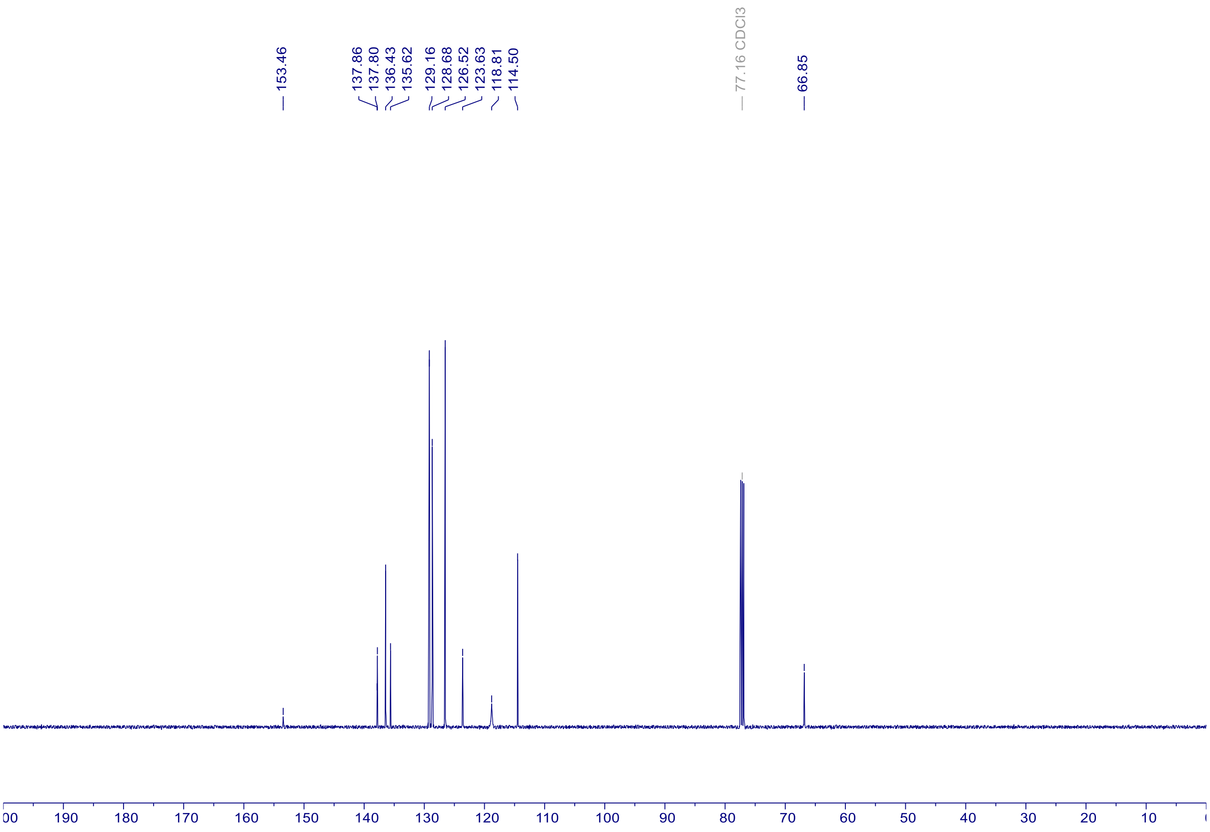

3f – ^{1}H NMR (400 MHz, CDCl$_3$)

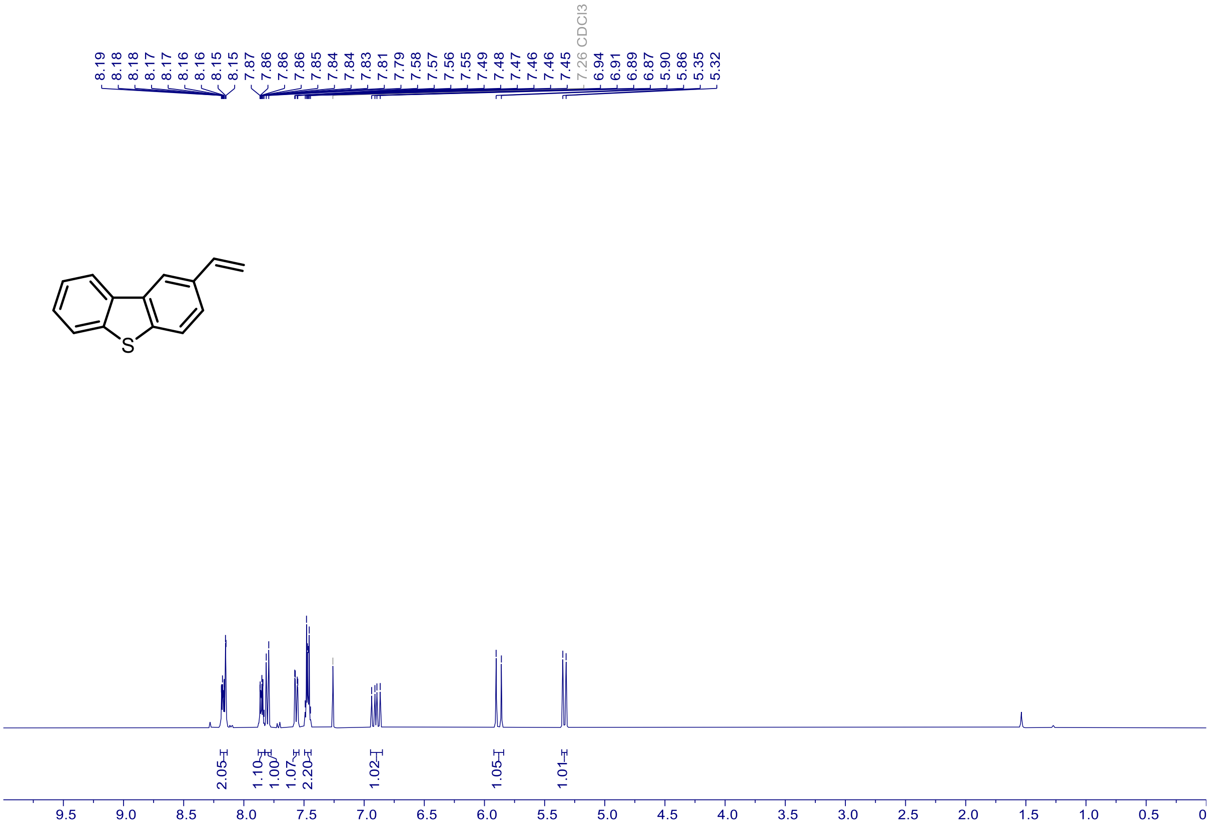

3f – ^{13}C NMR (101 MHz, CDCl$_3$)

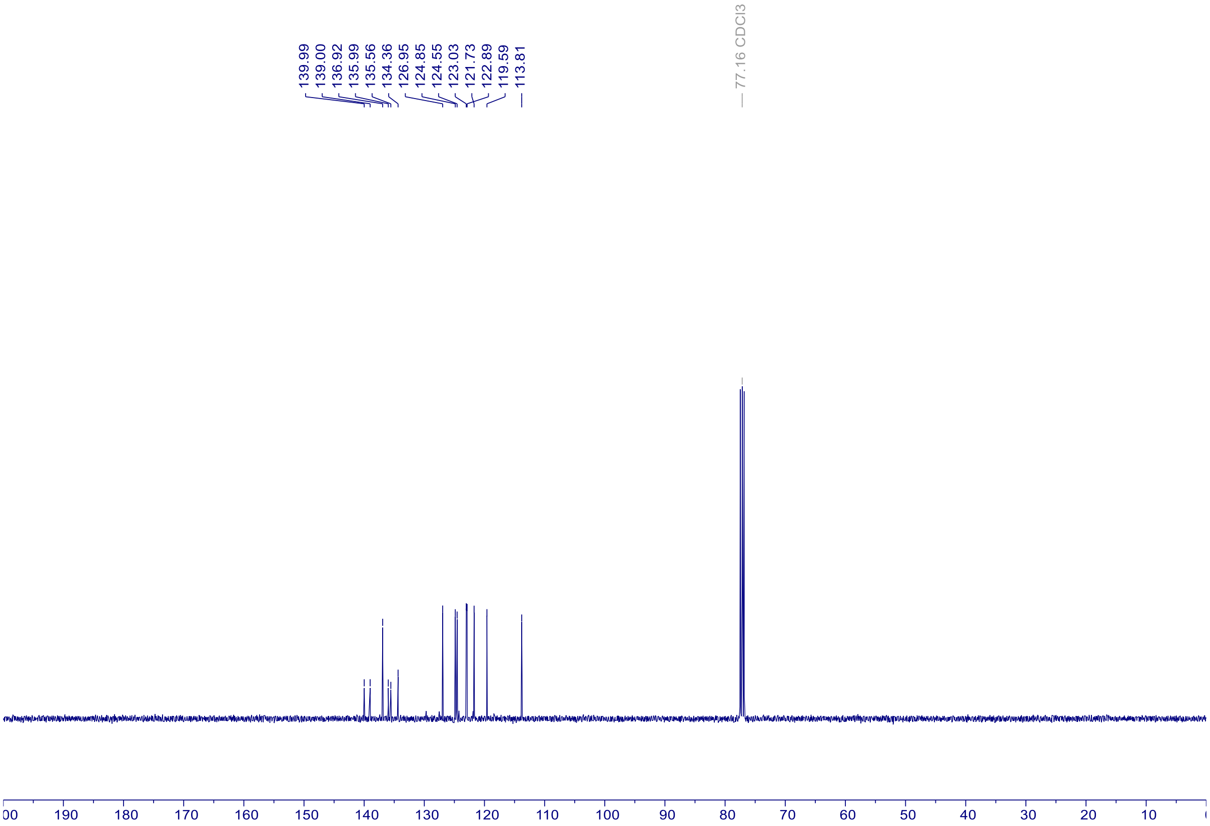

3g – ^{1}H NMR (400 MHz, $CDCl_3$)

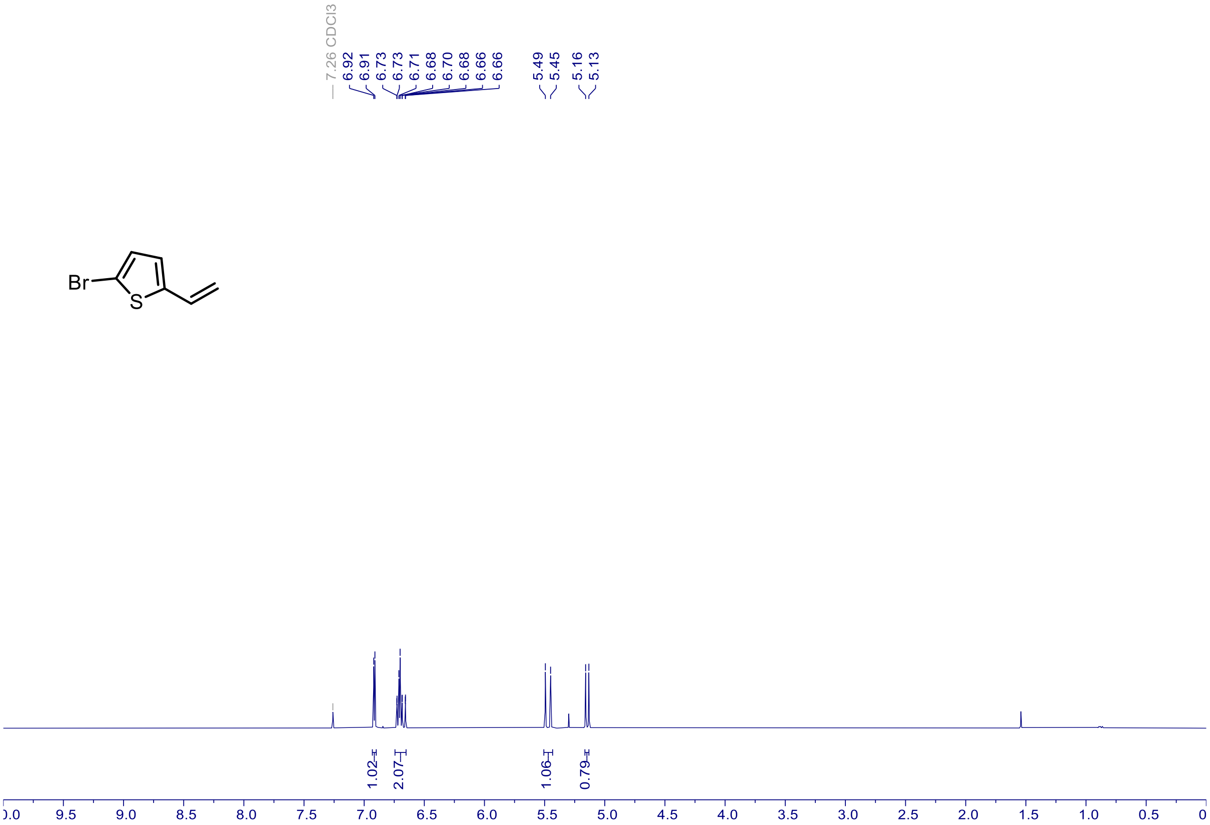

3g – ^{13}C NMR (101 MHz, $CDCl_3$)

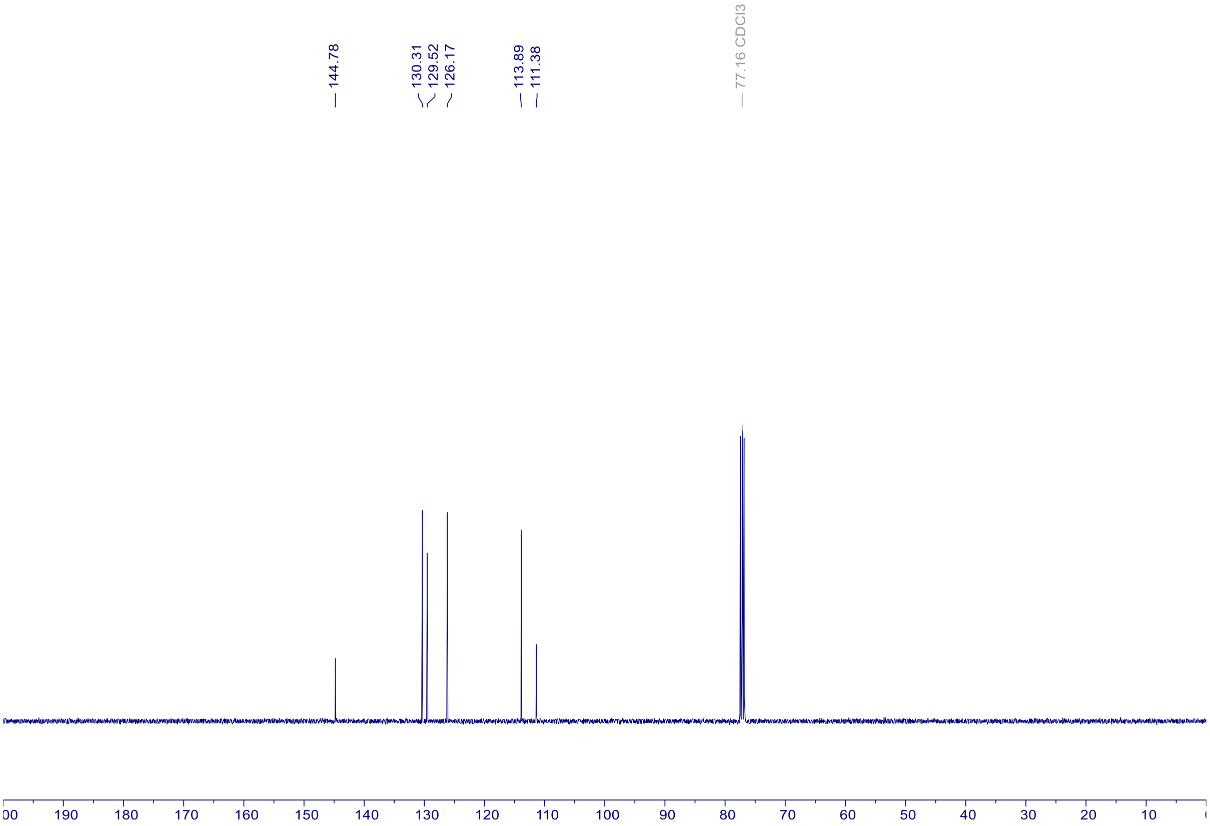

3h – ^{1}H NMR (400 MHz, $CDCl_3$)

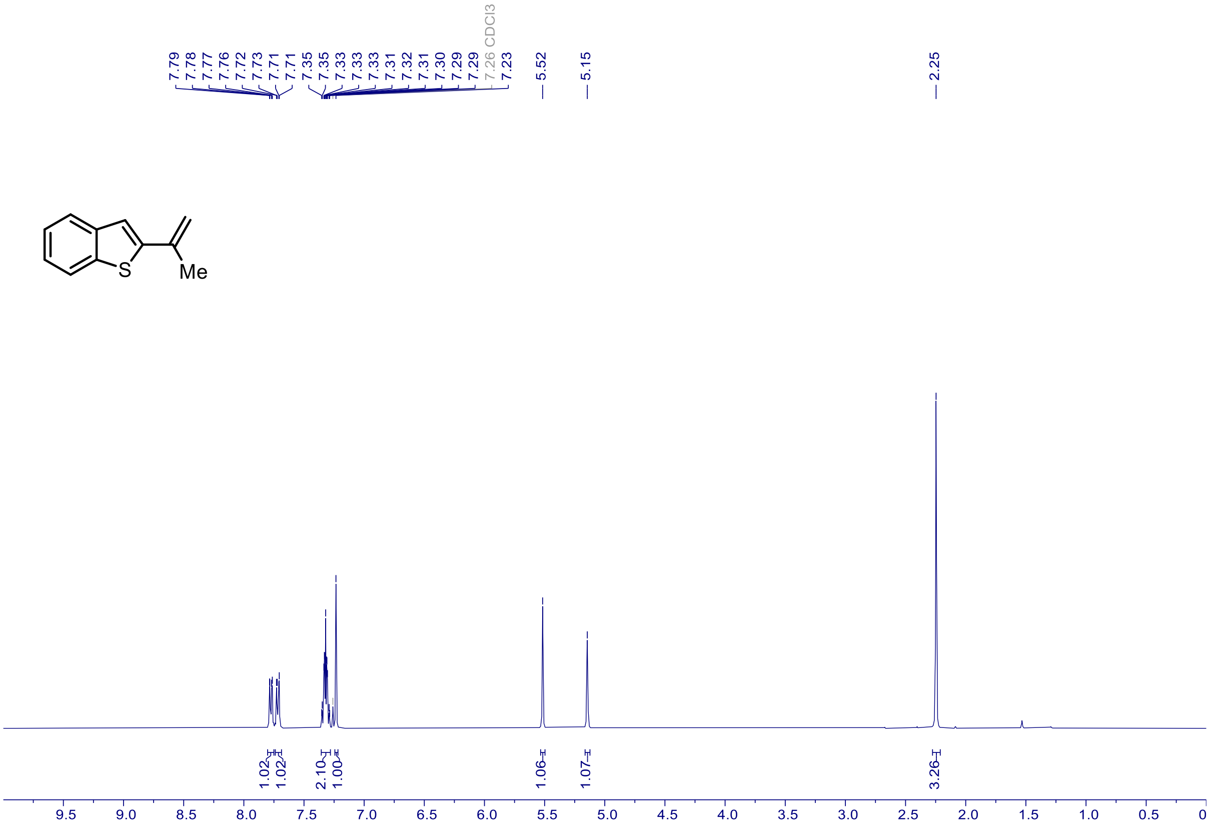

3h – ^{13}C NMR (101 MHz, $CDCl_3$)

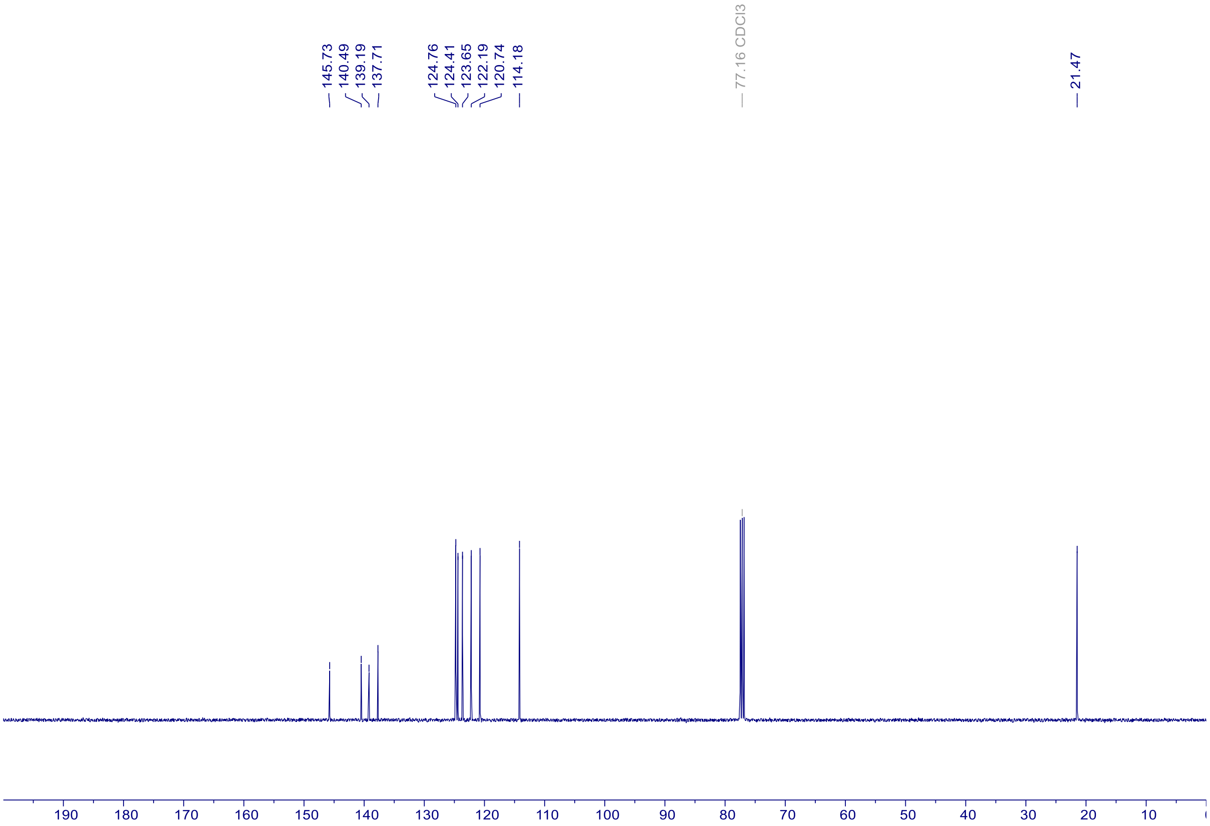

3i – ^{1}H NMR (500 MHz, $CDCl_3$)

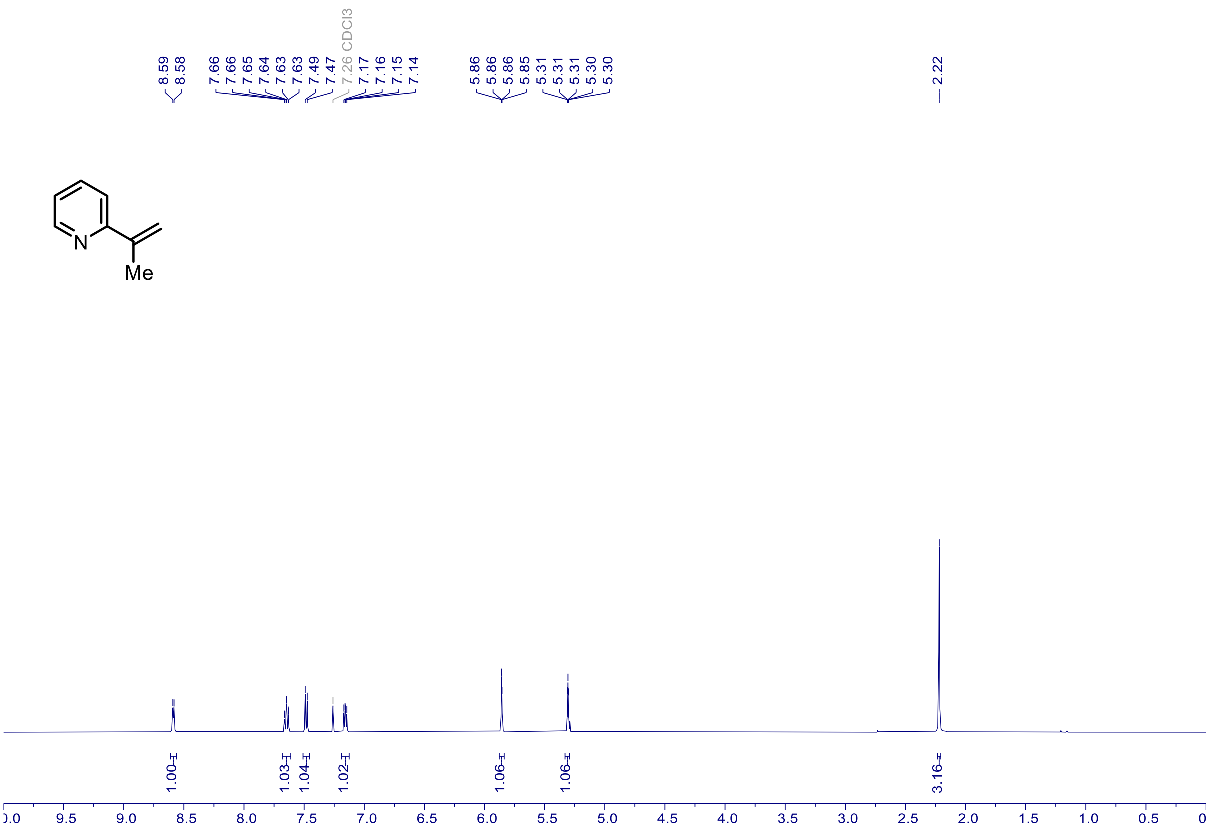

3i – ^{13}C NMR (126 MHz, $CDCl_3$)

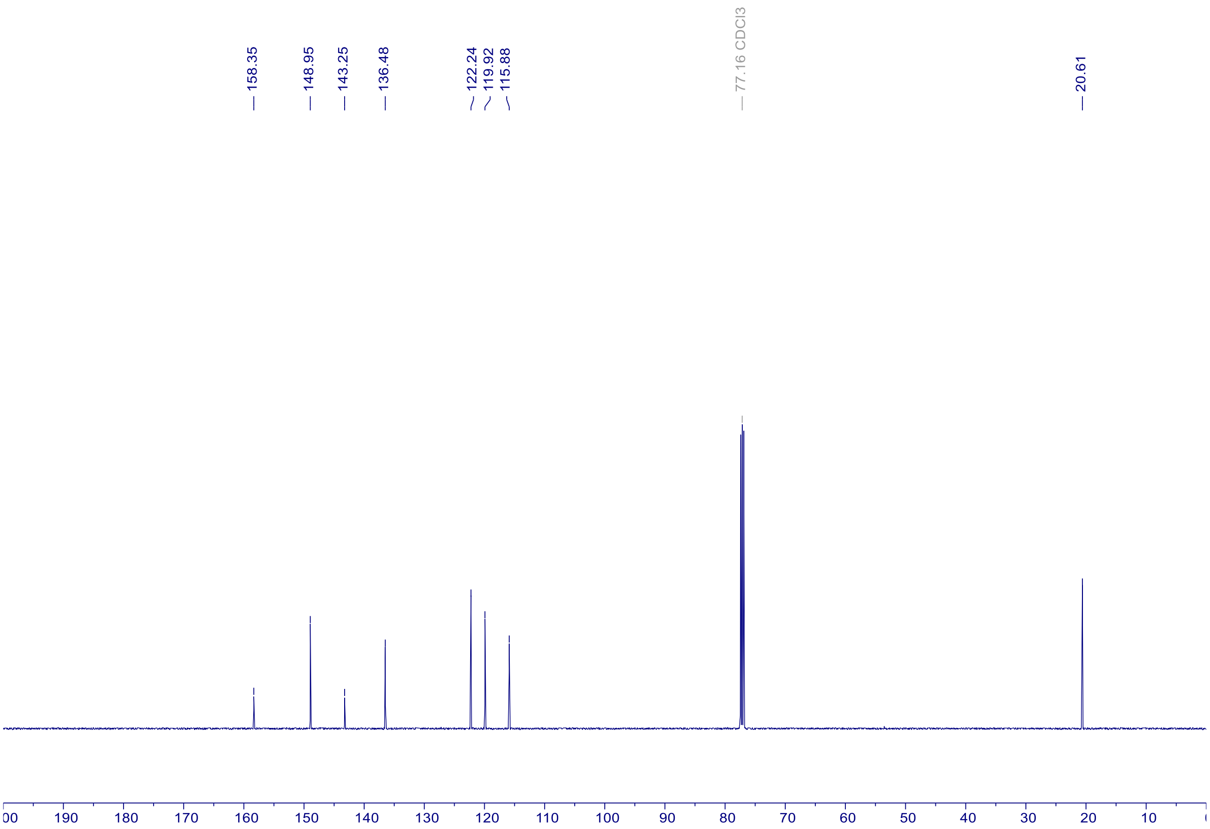

3j – ¹H NMR (400 MHz, $CDCl_3$)

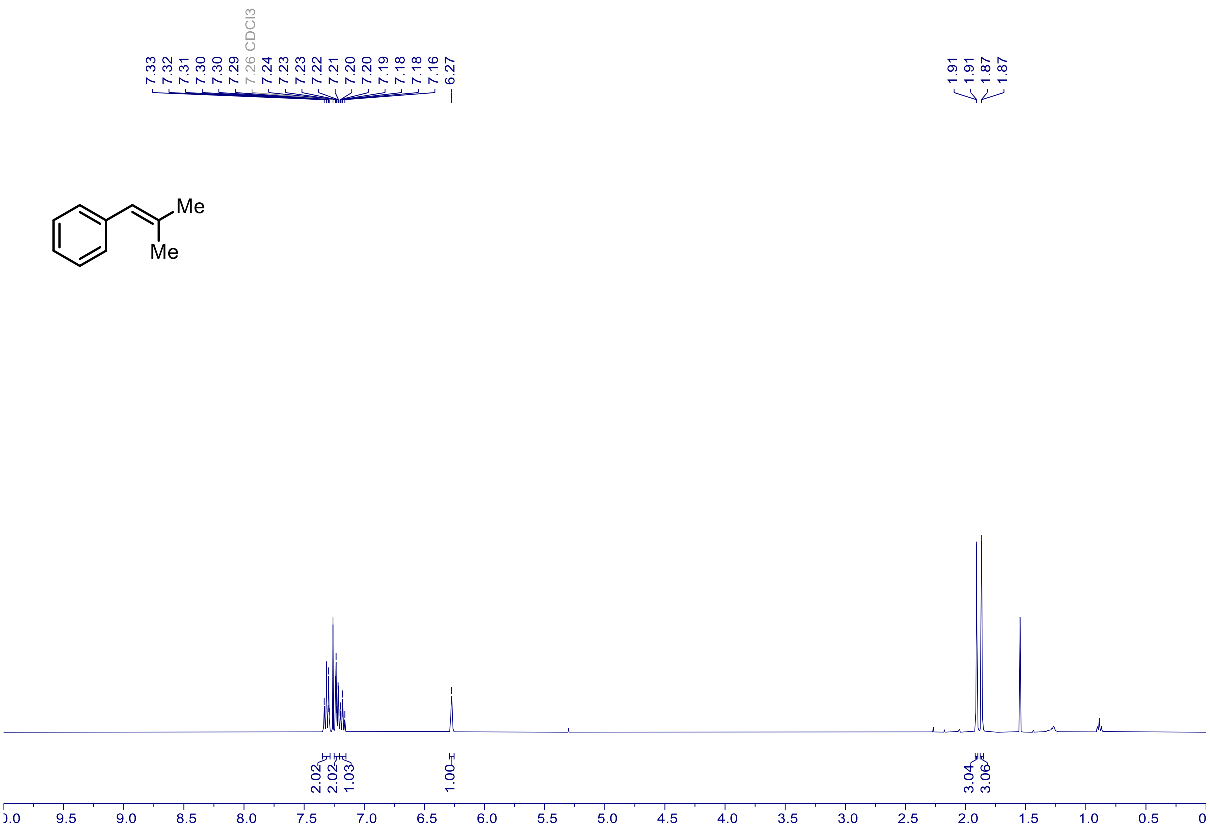

3j – ¹³C NMR (101 MHz, $CDCl_3$)

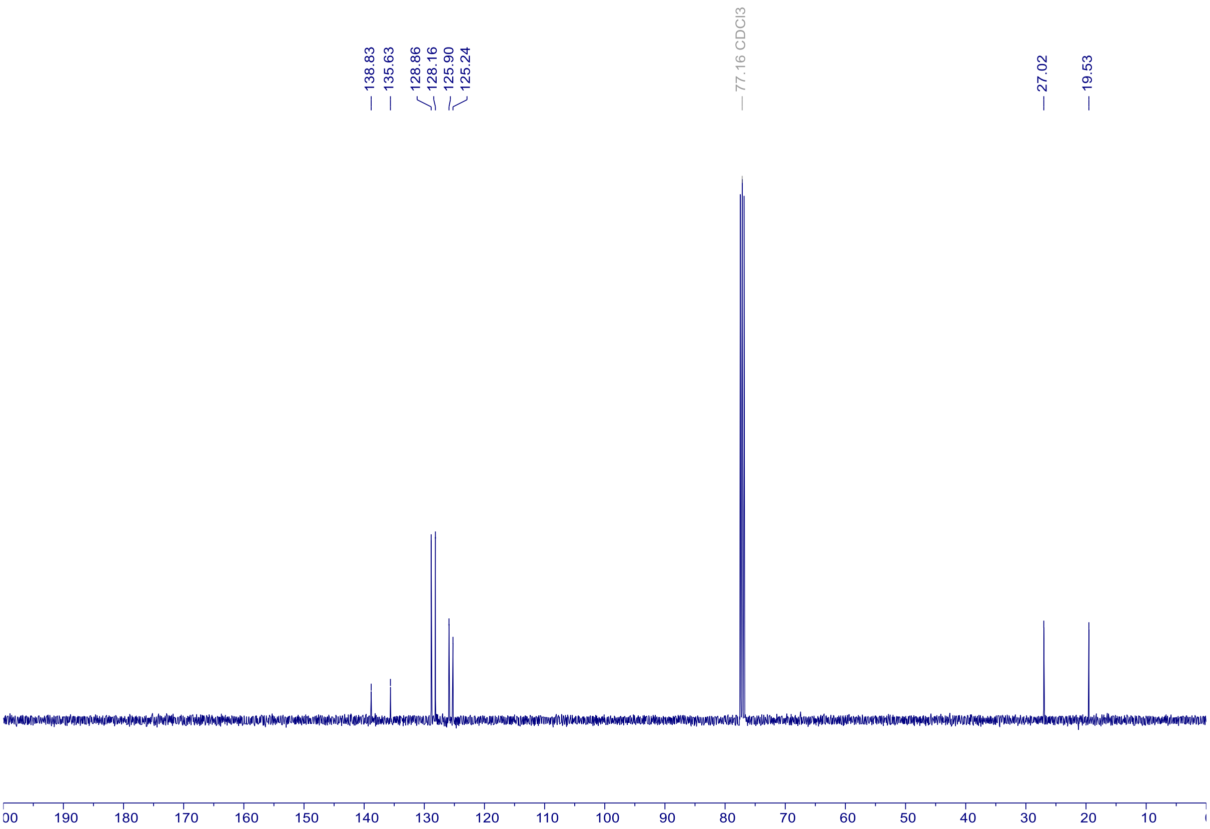

3k – ^{1}H NMR (400 MHz, $CDCl_3$)

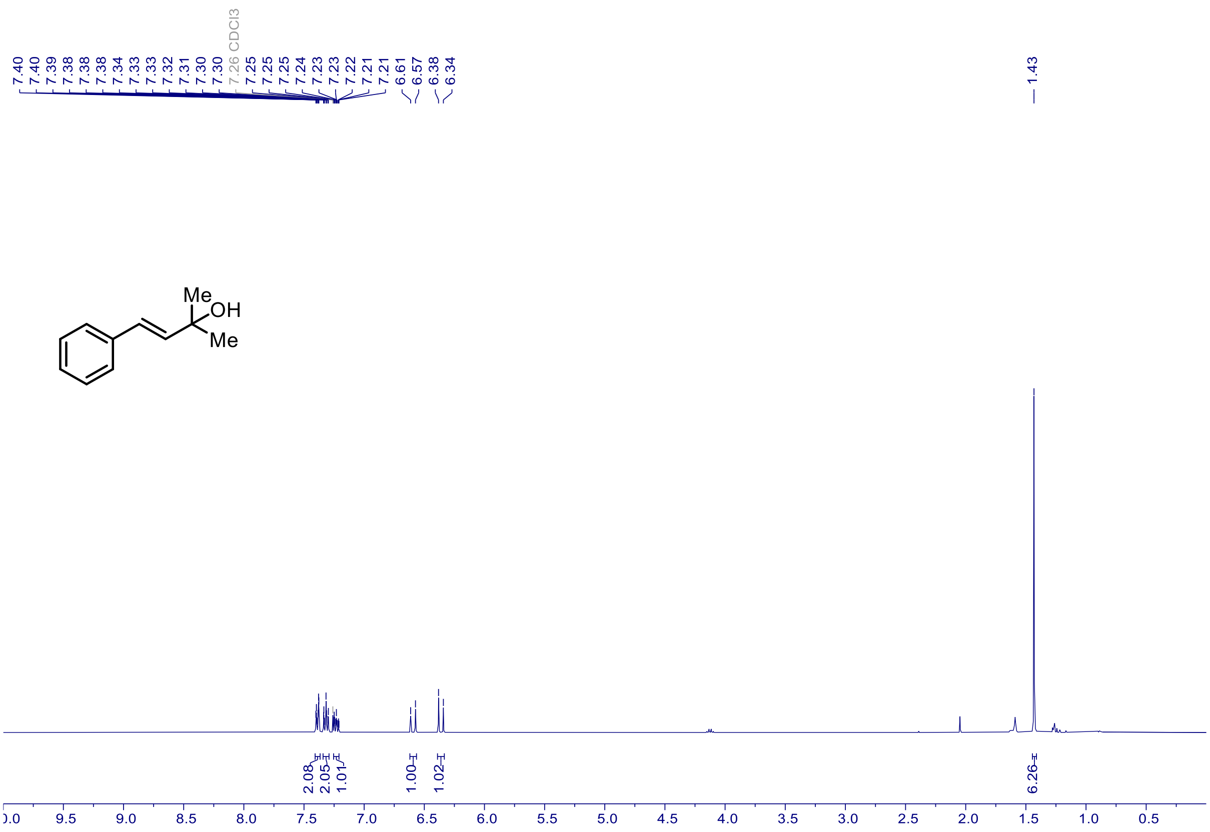

3k – ^{13}C NMR (101 MHz, $CDCl_3$)

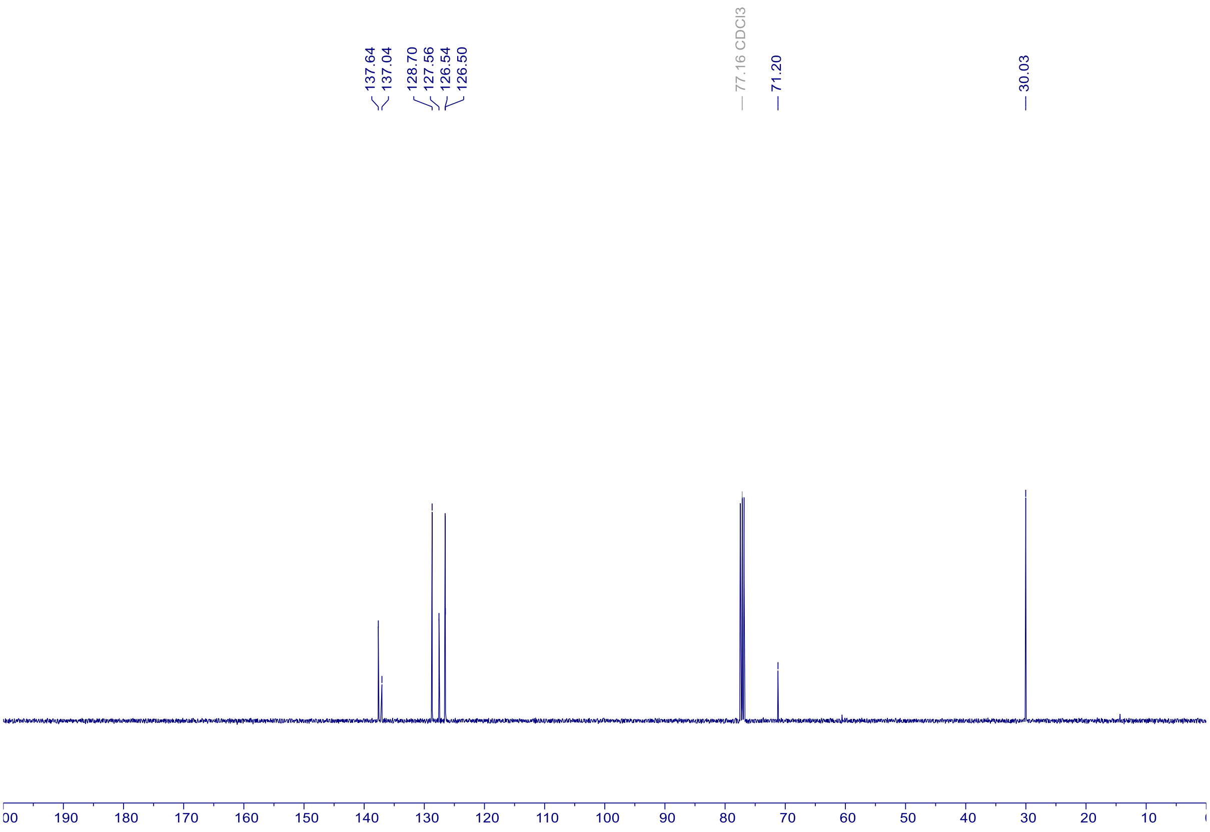

3l – ^{1}H NMR (500 MHz, $CDCl_3$)

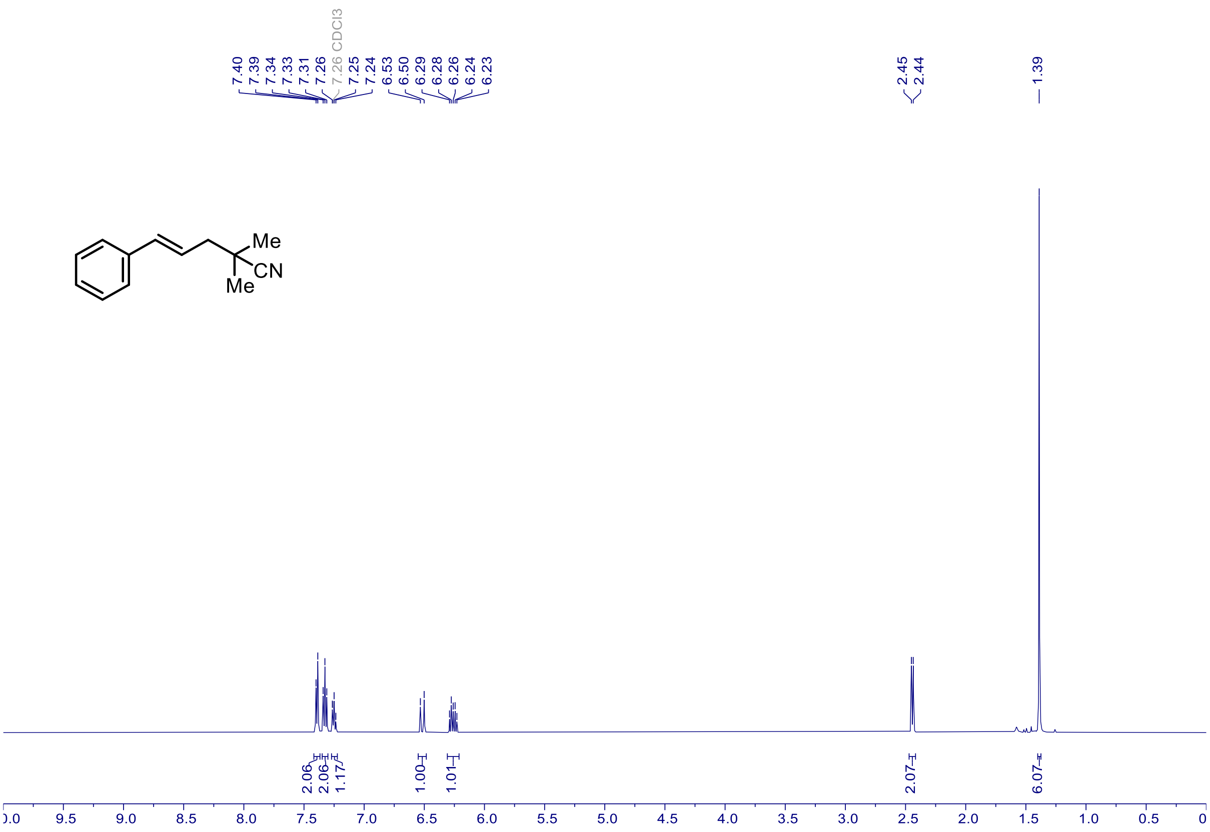

3l – ^{13}C NMR (126 MHz, $CDCl_3$)

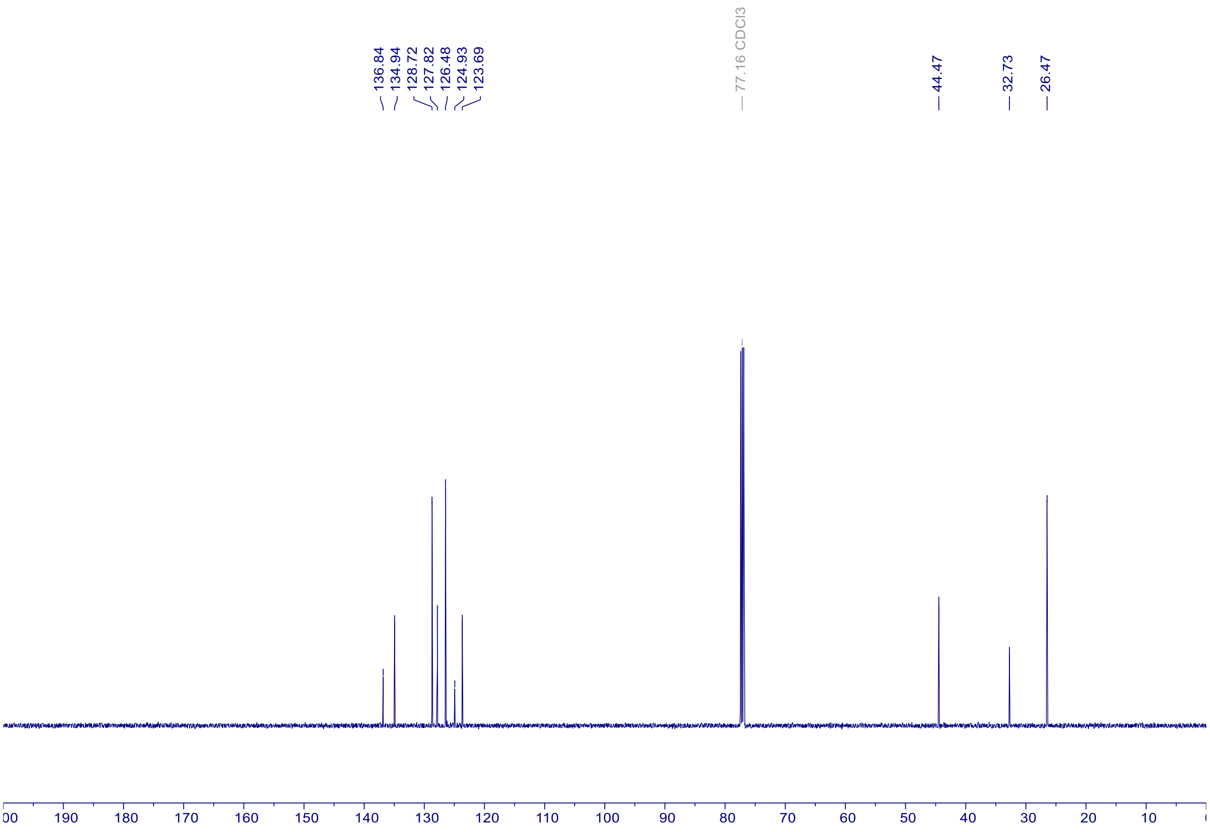

3m – ^{1}H NMR (500 MHz, CDCl$_3$)

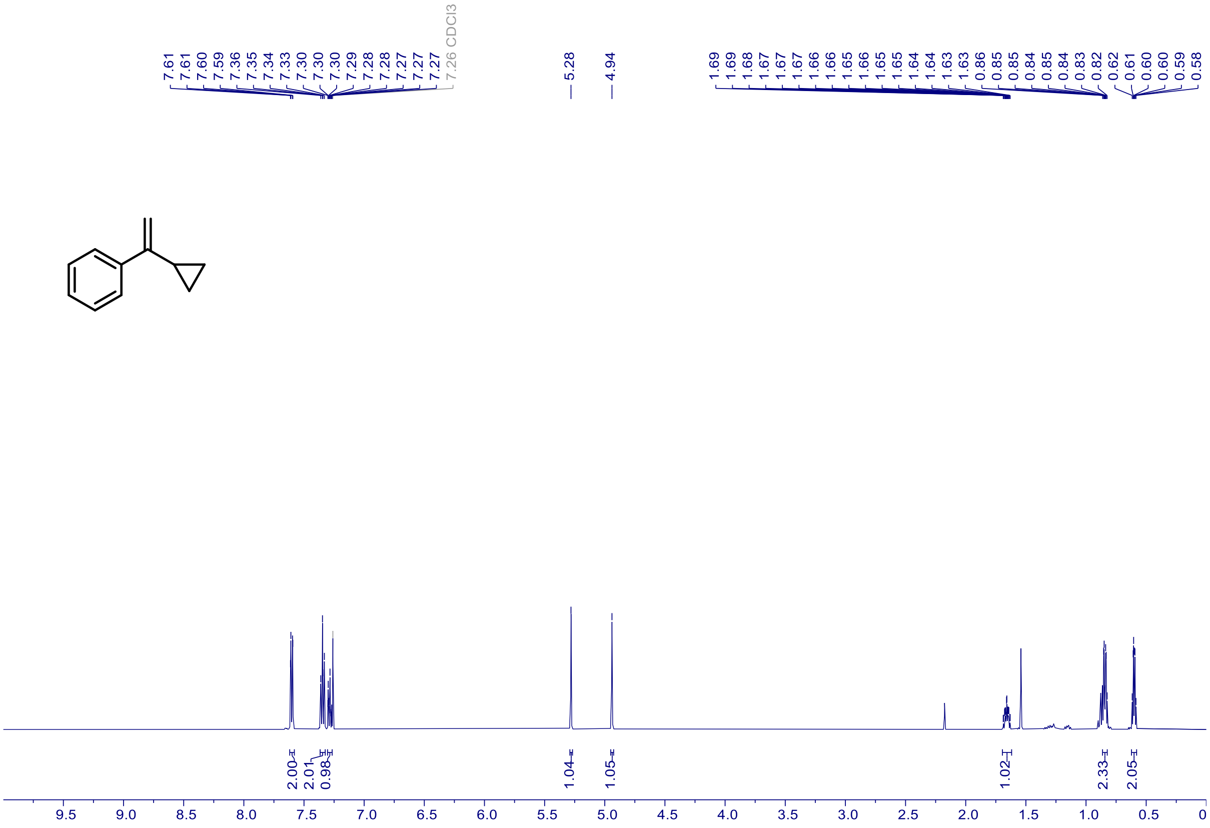

3m – ^{13}C NMR (126 MHz, CDCl$_3$)

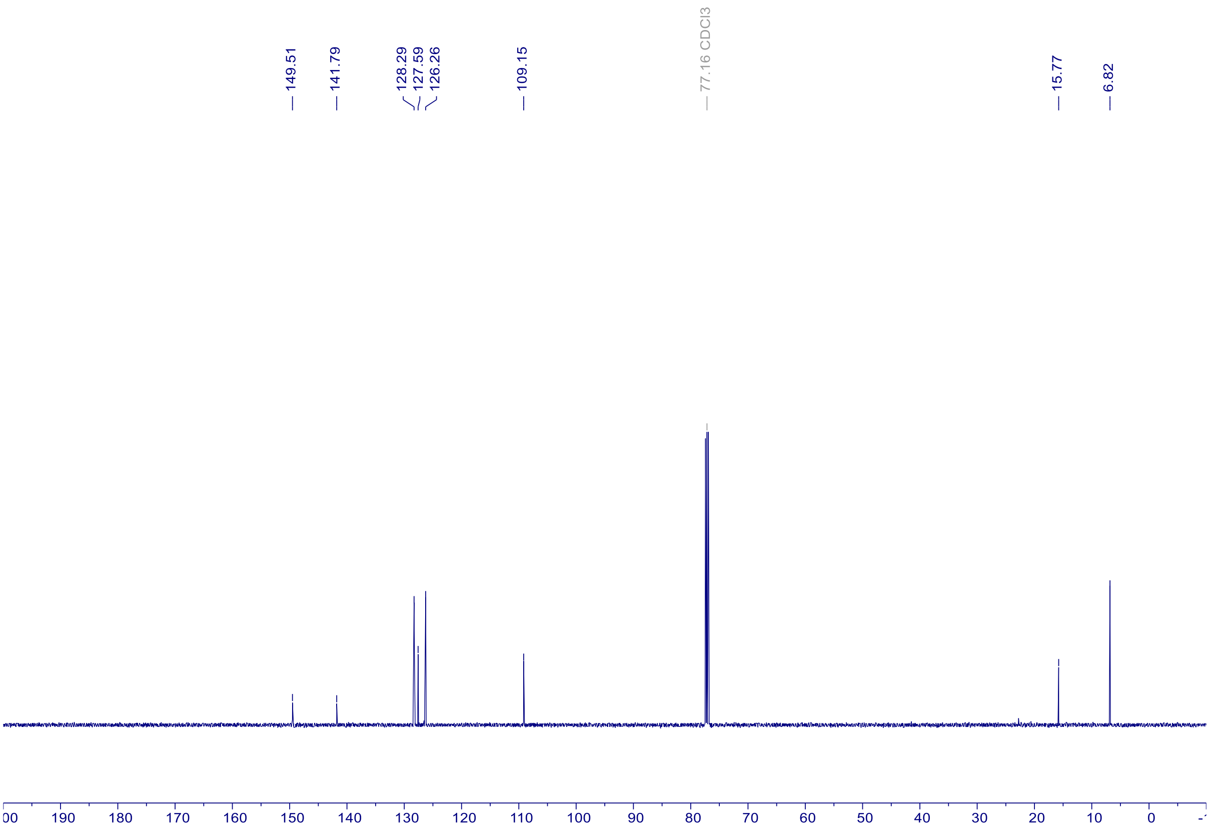

3n – ^{1}H NMR (400 MHz, DMSO-d_6)

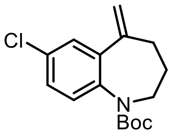

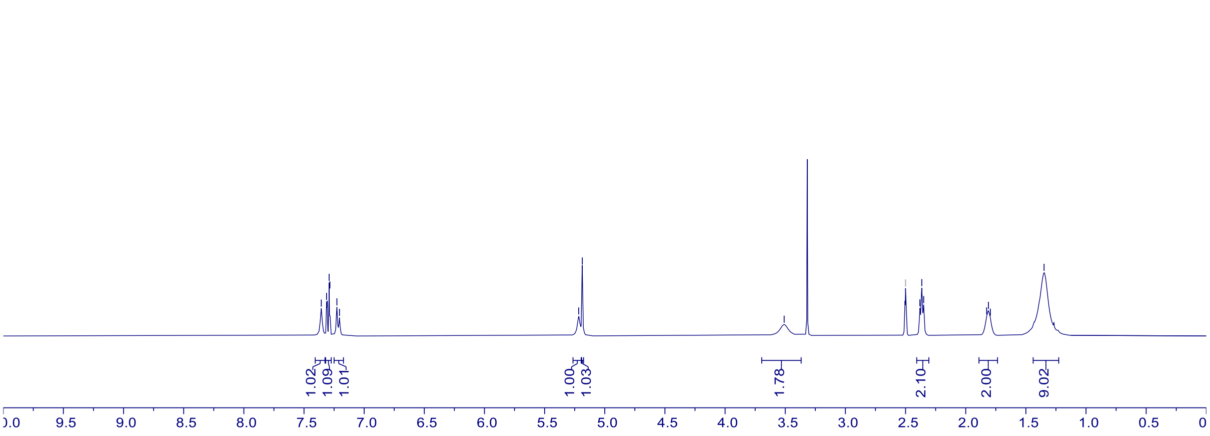

3n – ^{13}C NMR (101 MHz, DMSO-d_6)

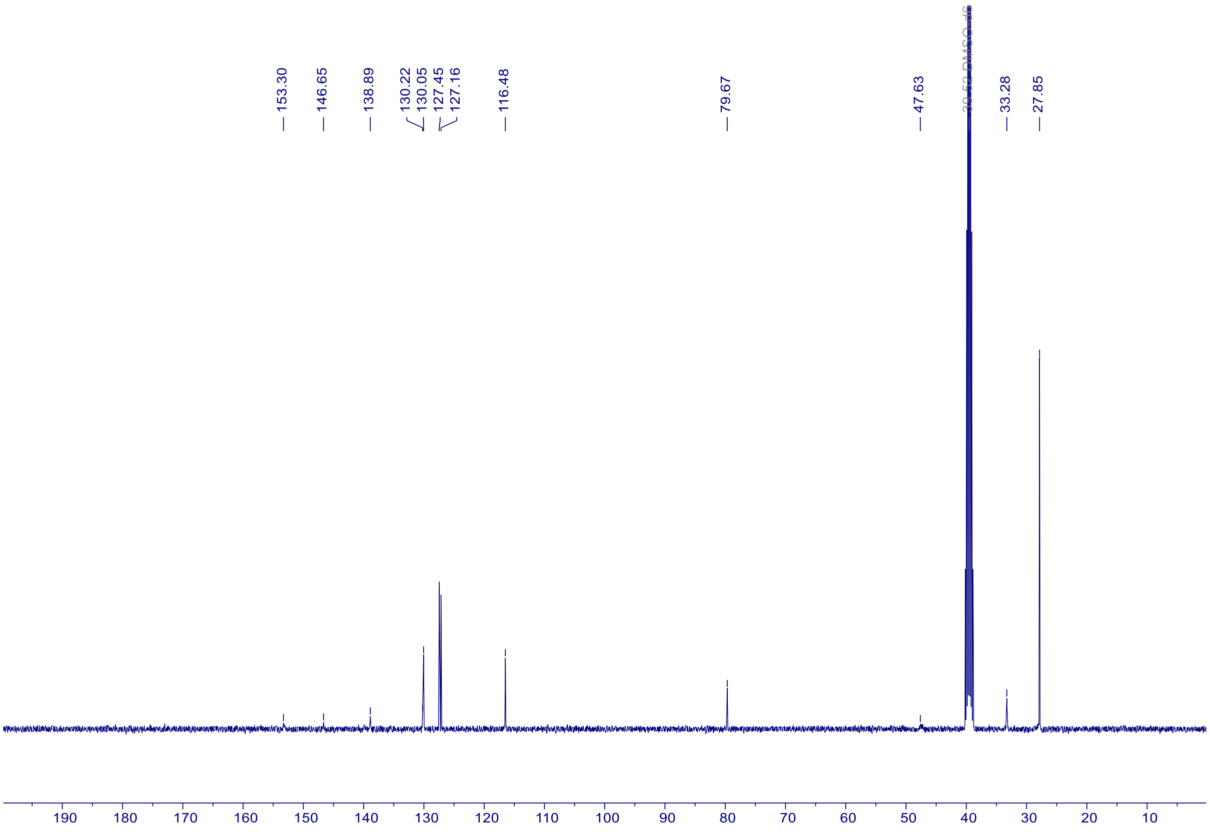

3o – ^{1}H NMR (400 MHz, $CDCl_3$)

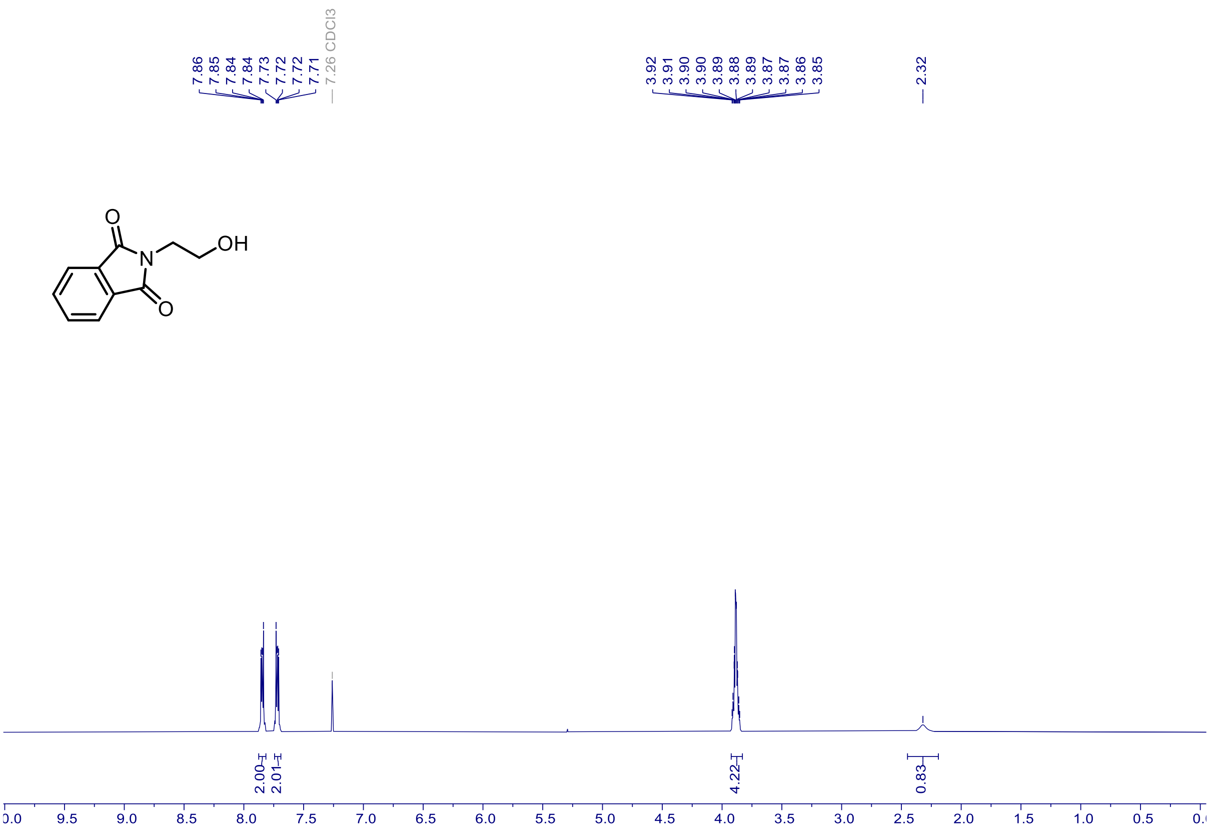

3o – ^{13}C NMR (101 MHz, $CDCl_3$)

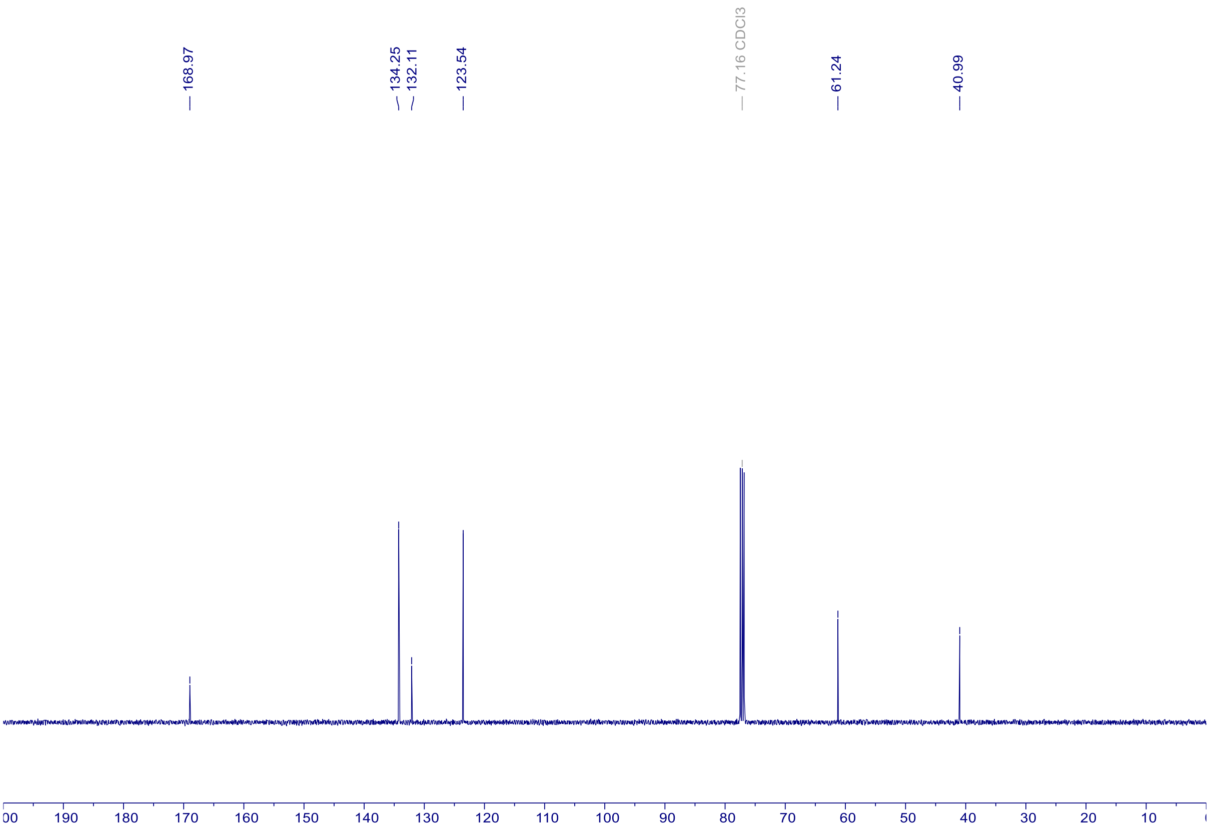

3p – ^{1}H NMR (500 MHz, $CDCl_3$)

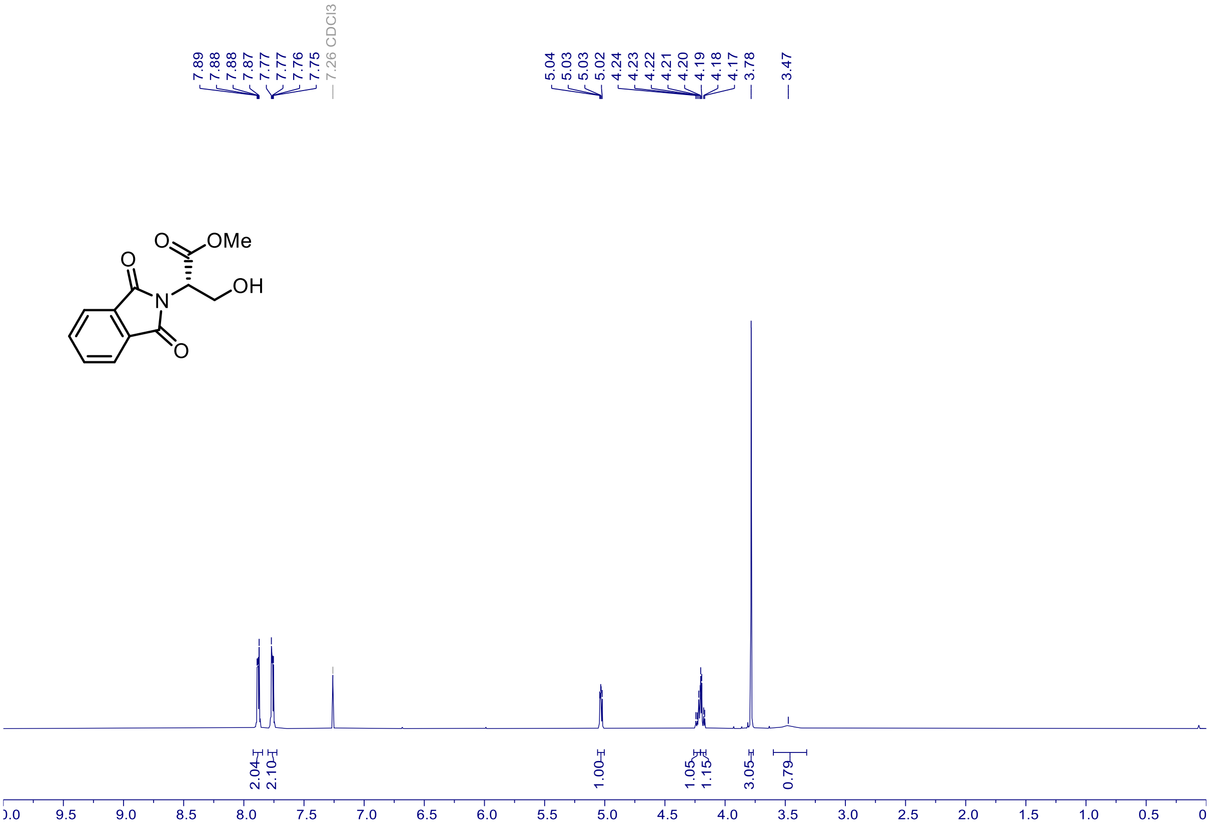

3p – ^{13}C NMR (126 MHz, $CDCl_3$)

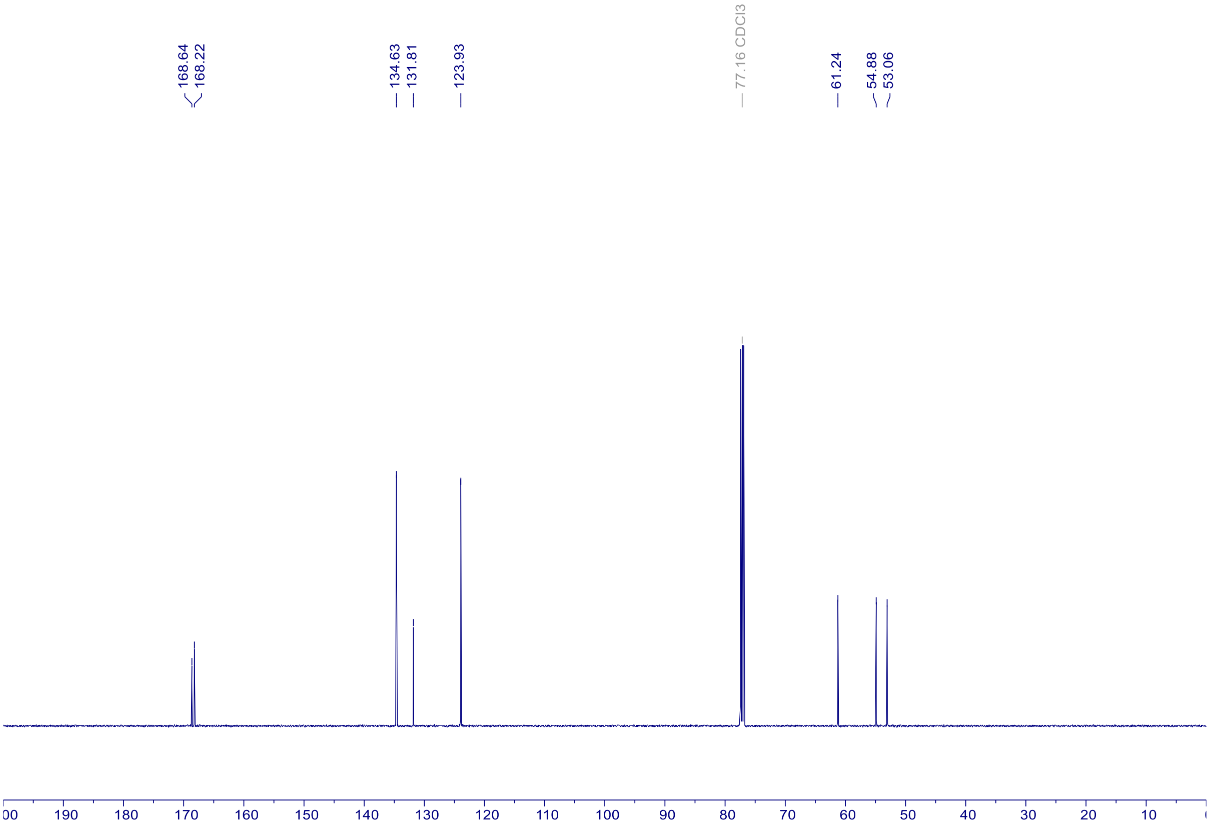

3p' – ^{1}H NMR (500 MHz, $CDCl_3$)

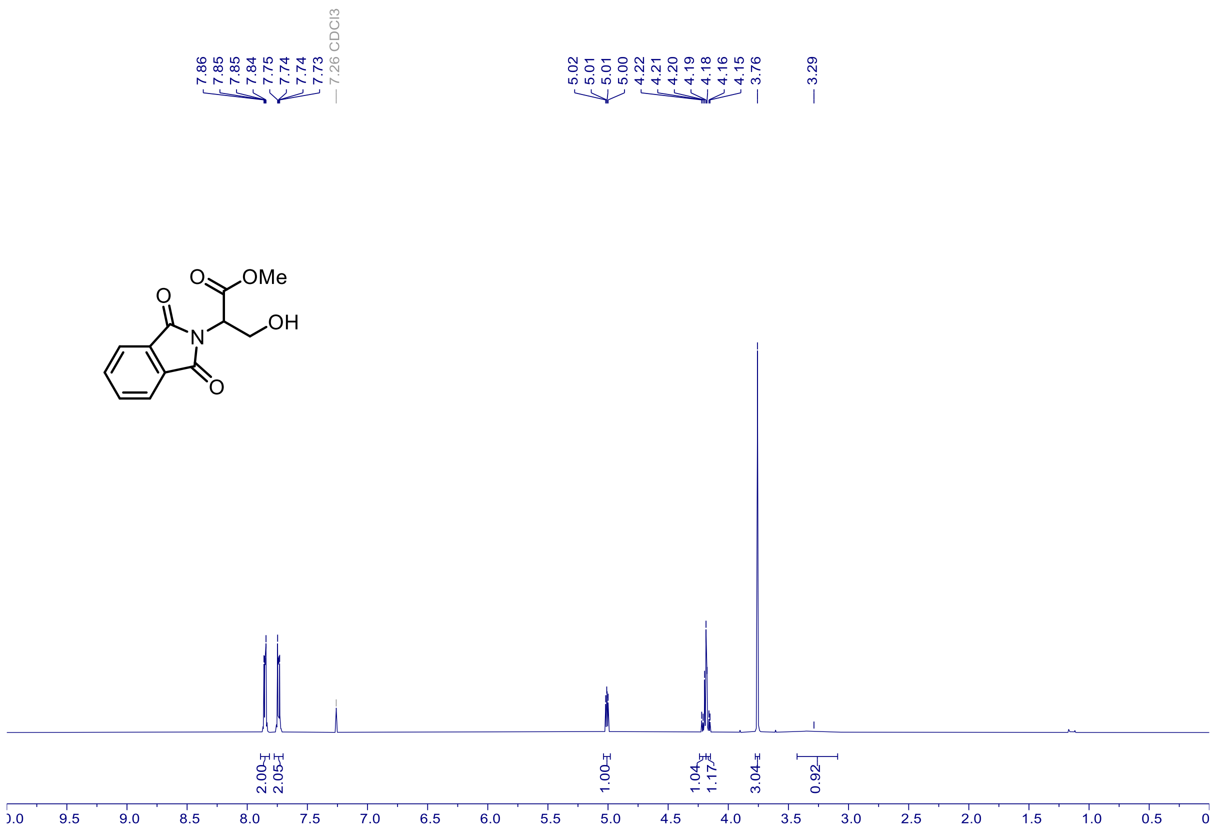

3p' – ^{13}C NMR (126 MHz, $CDCl_3$)

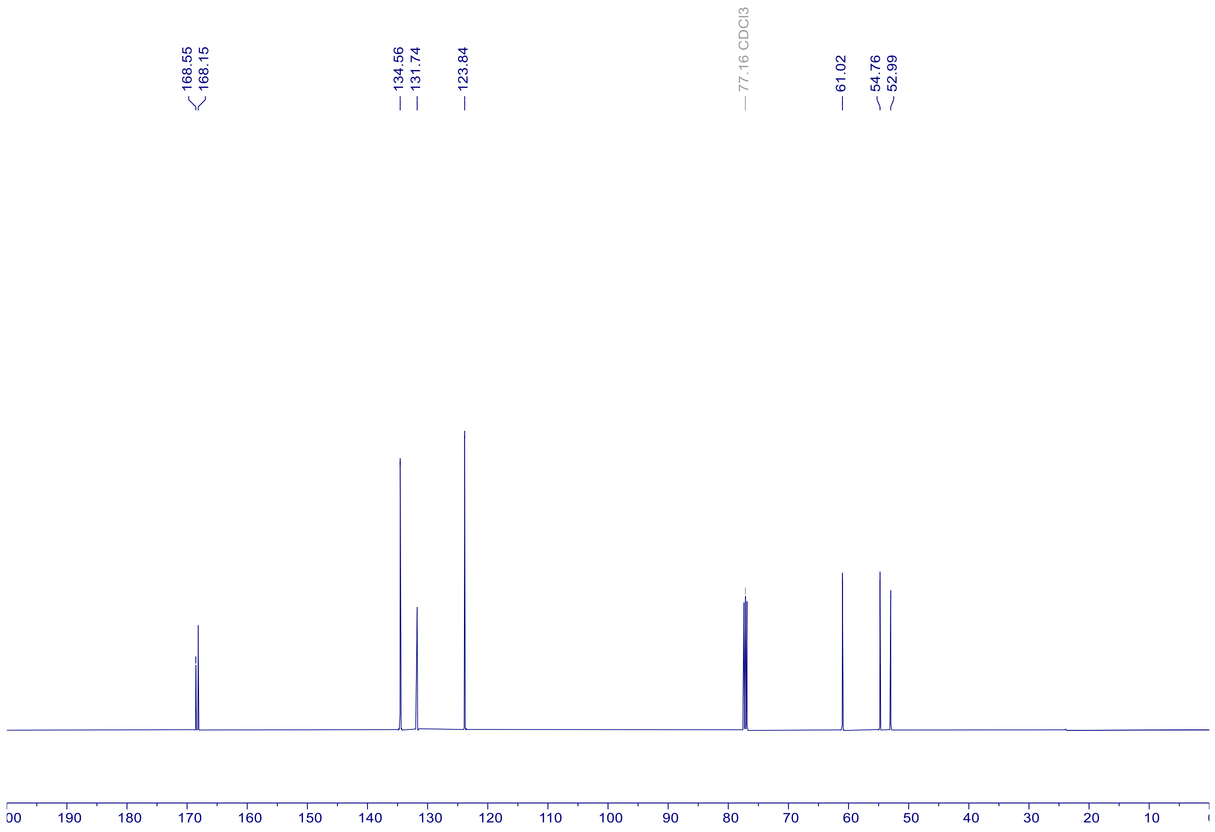

4a – ^{1}H NMR (500 MHz, $CDCl_3$)

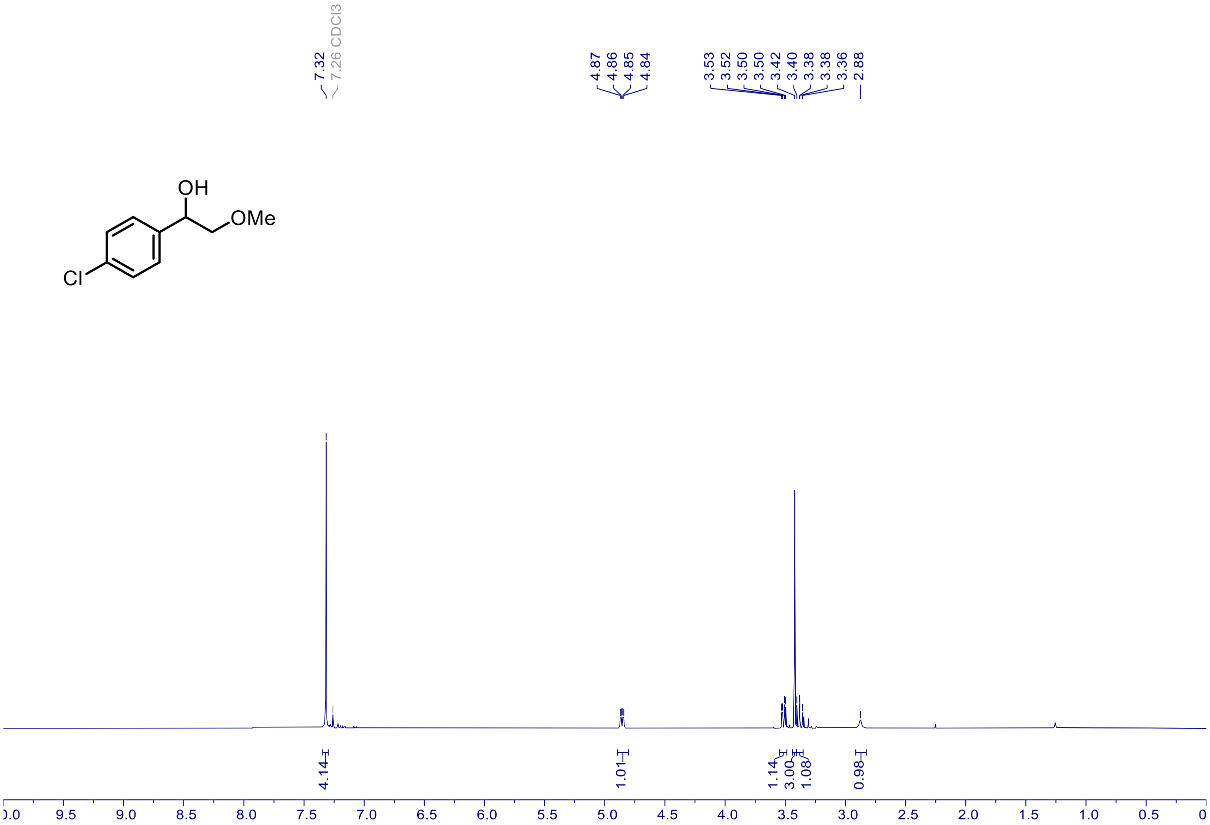

4a – ^{13}C NMR (126 MHz, $CDCl_3$)

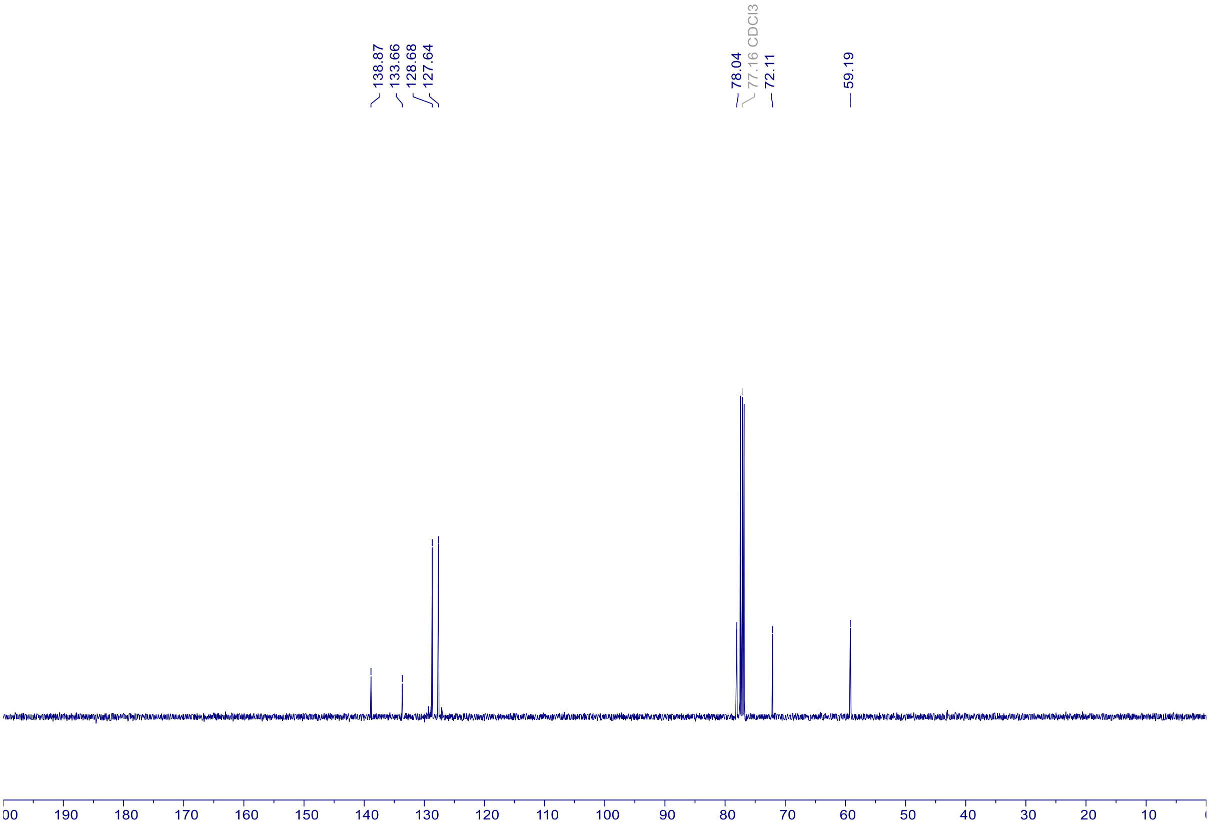

4b – ^{1}H NMR (400 MHz, $CDCl_3$)

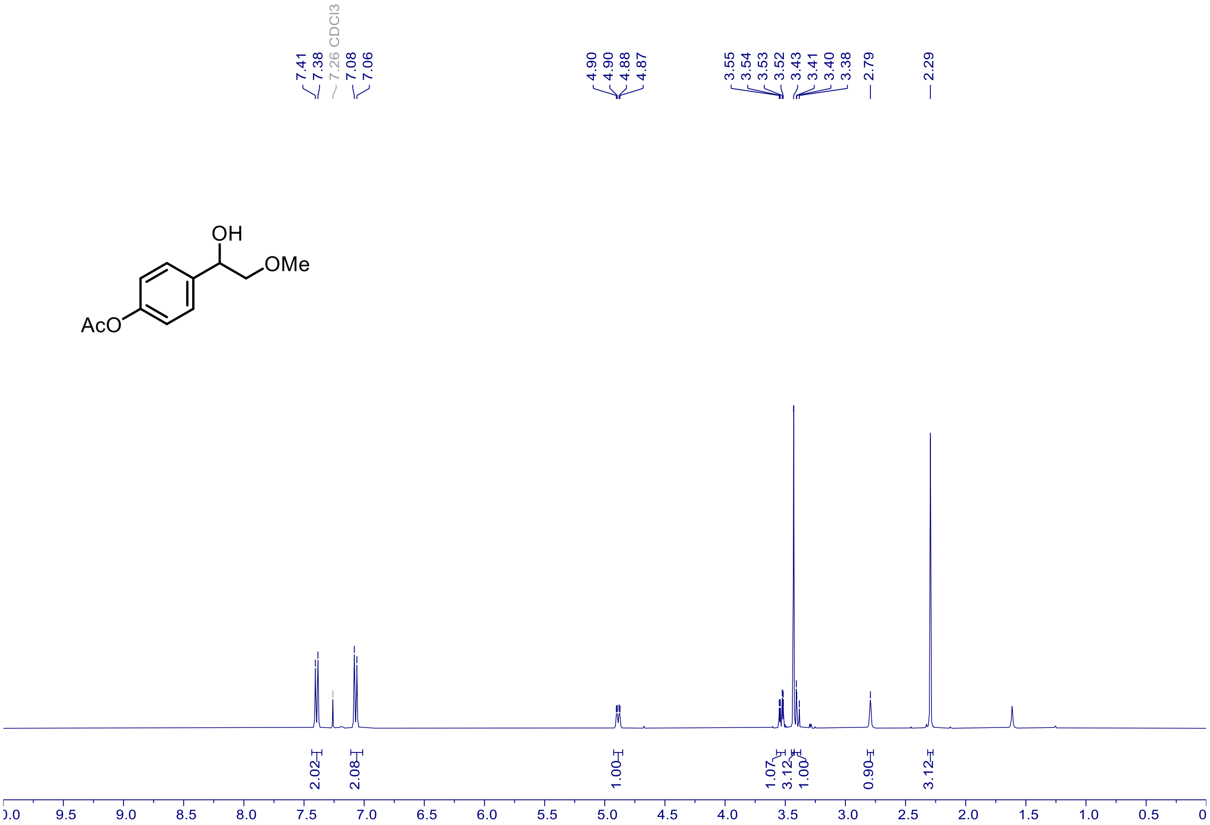

4b – ^{13}C NMR (101 MHz, $CDCl_3$)

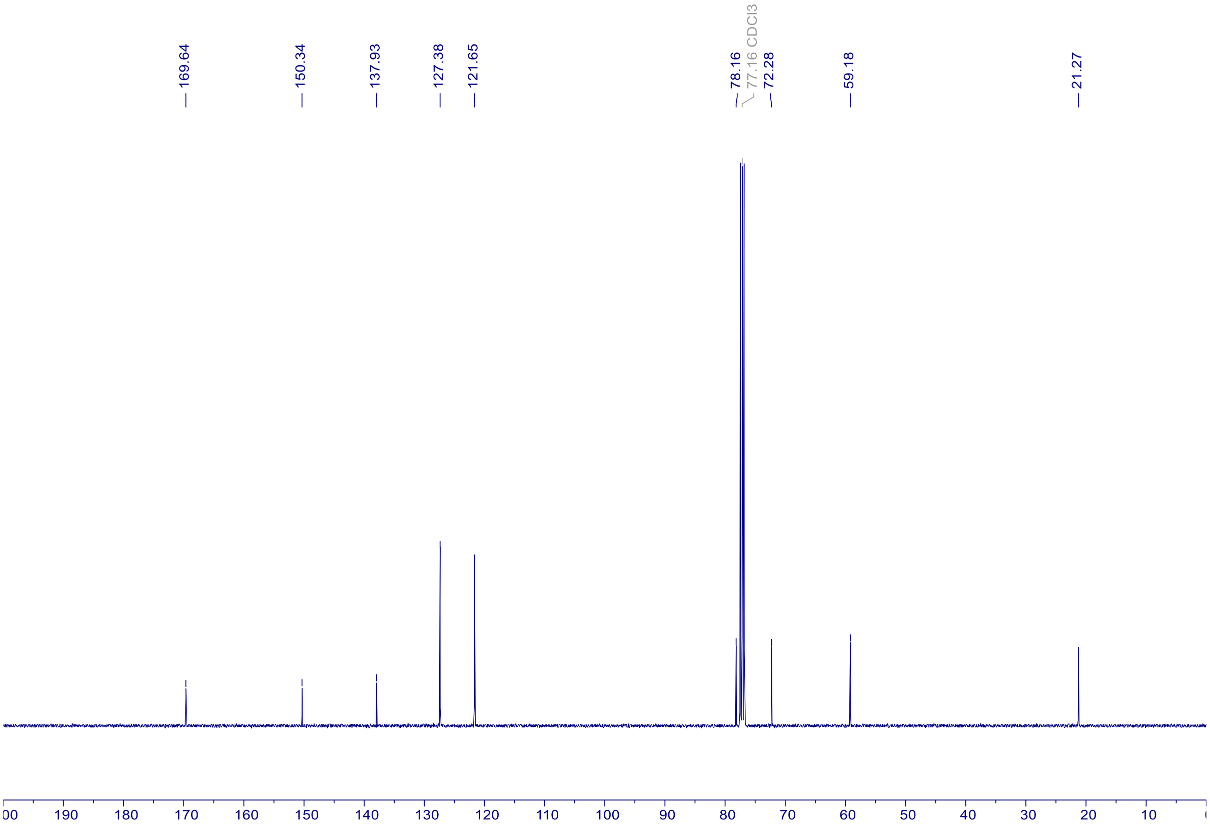

4c – ^{1}H NMR (400 MHz, CDCl$_3$)

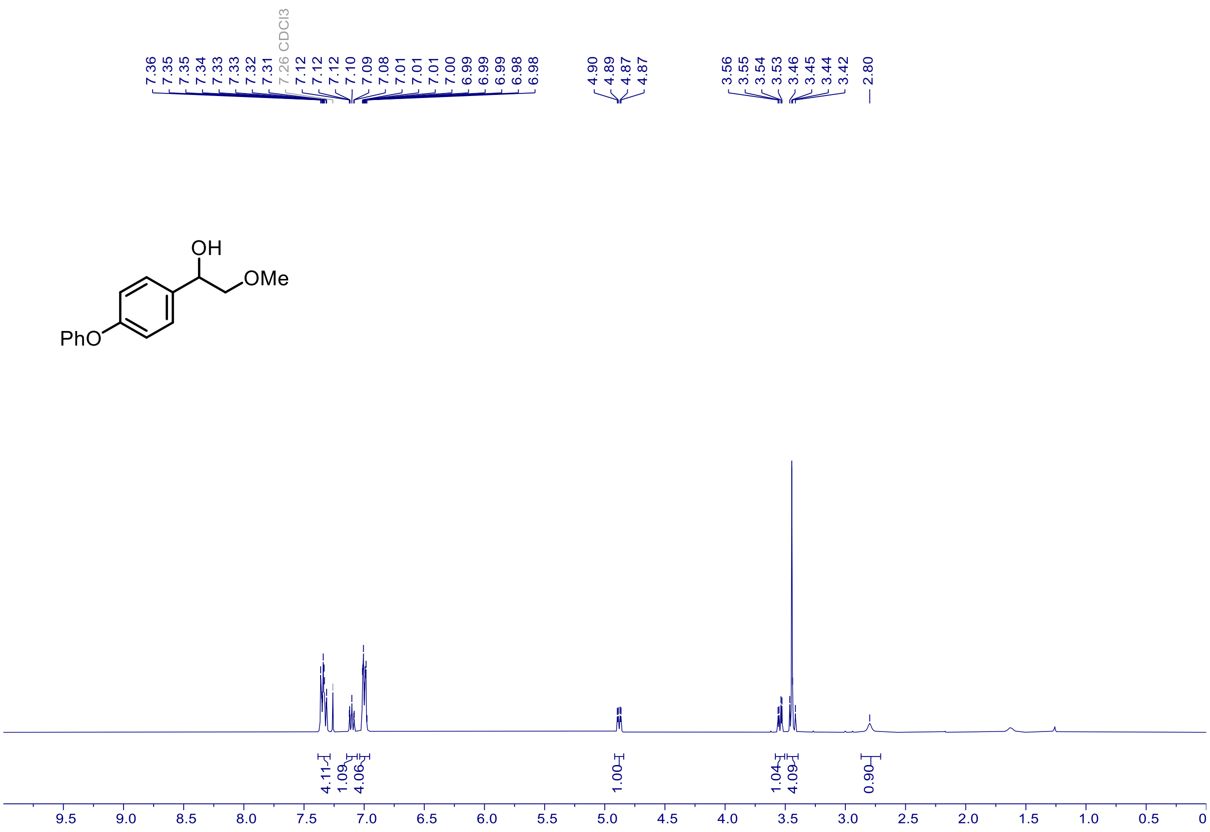

4c – ^{13}C NMR (101 MHz, CDCl$_3$)

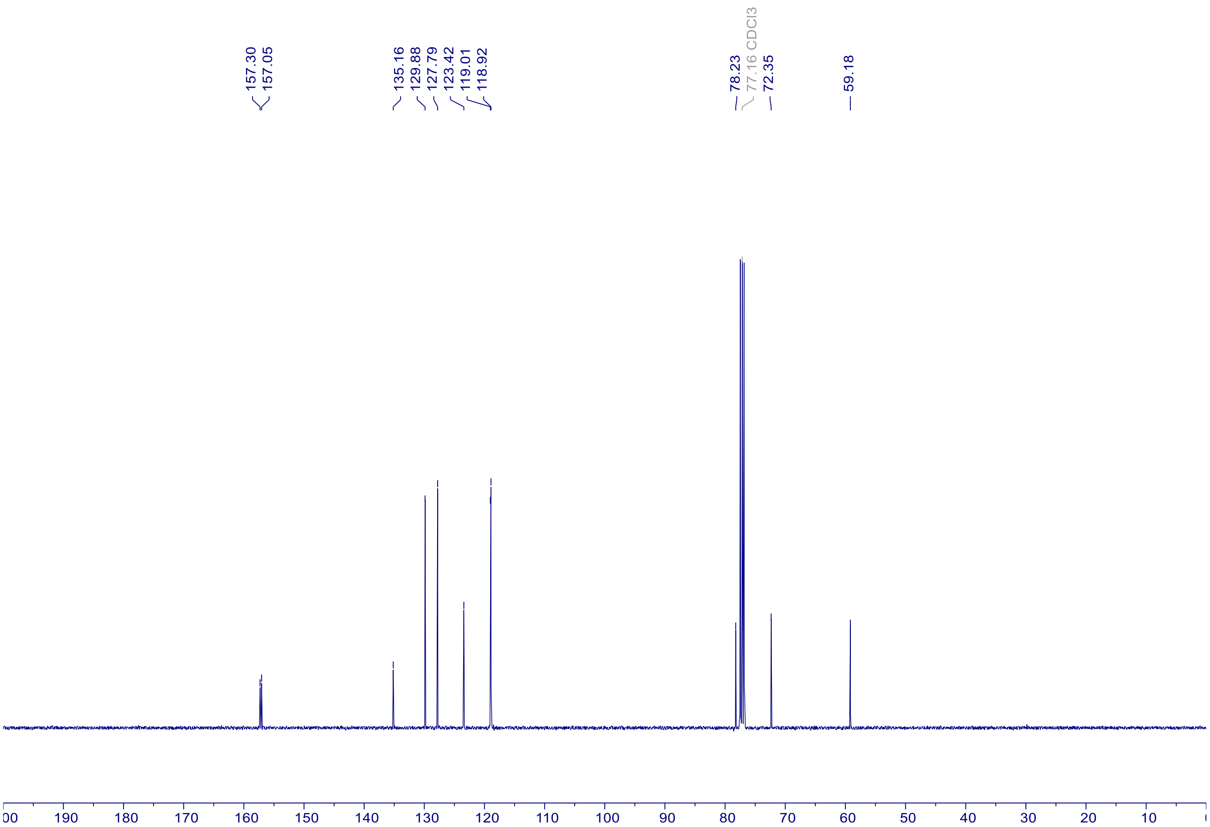

4e – ^{1}H NMR (400 MHz, CDCl$_3$)

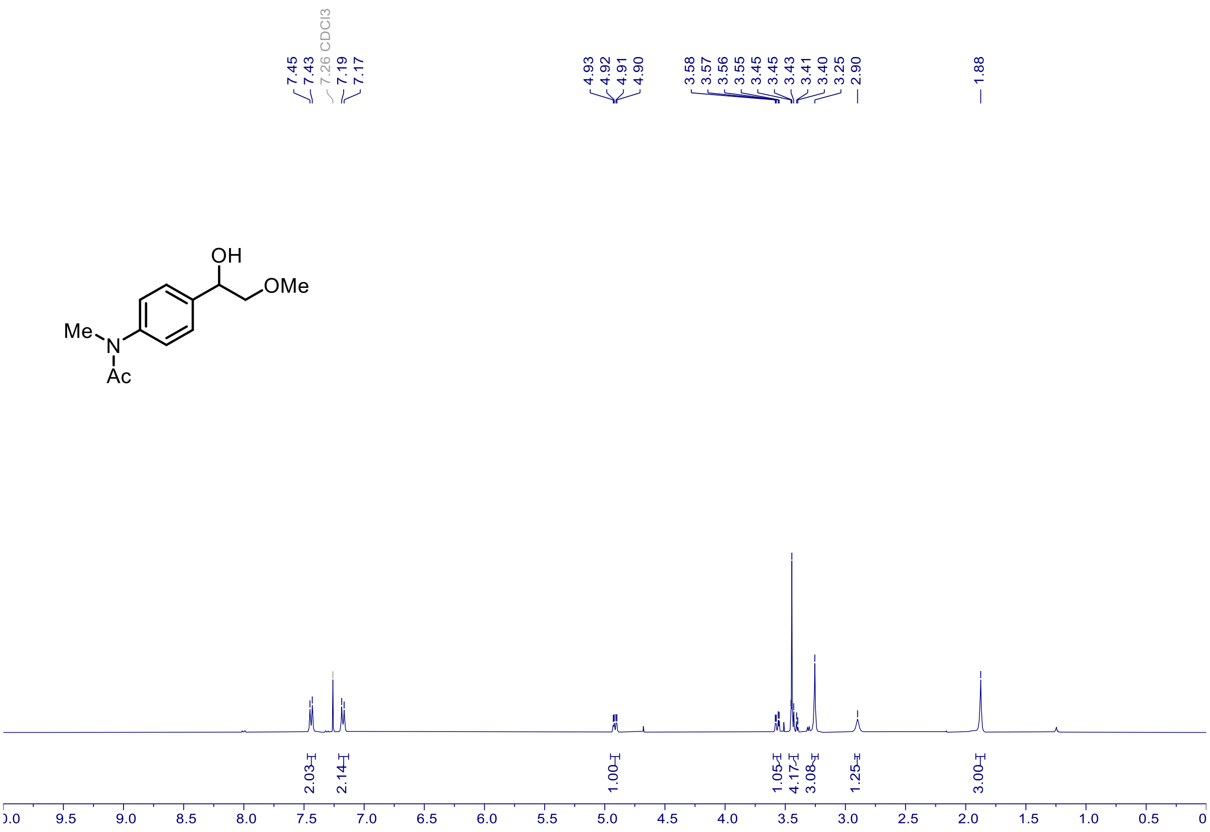

4e – ^{13}C NMR (101 MHz, CDCl$_3$)

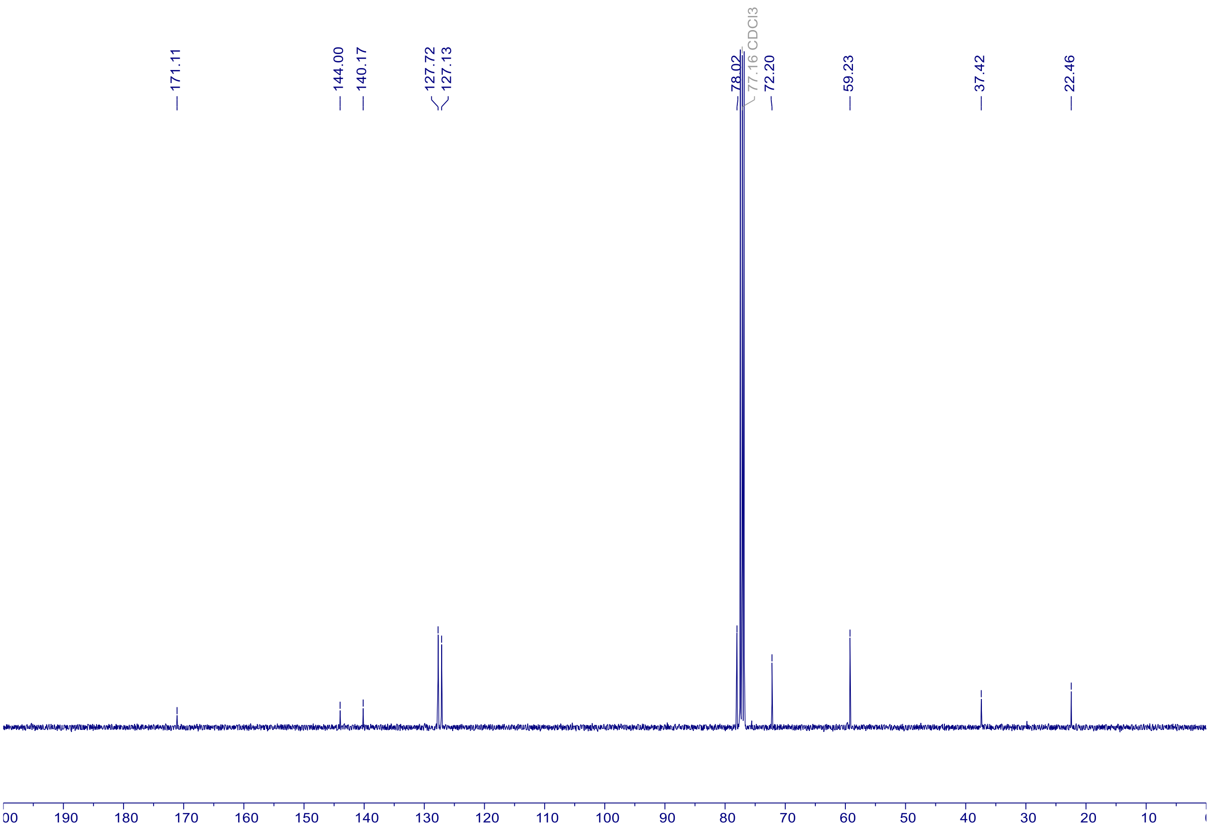

4f – ^{1}H NMR (400 MHz, $CDCl_3$)

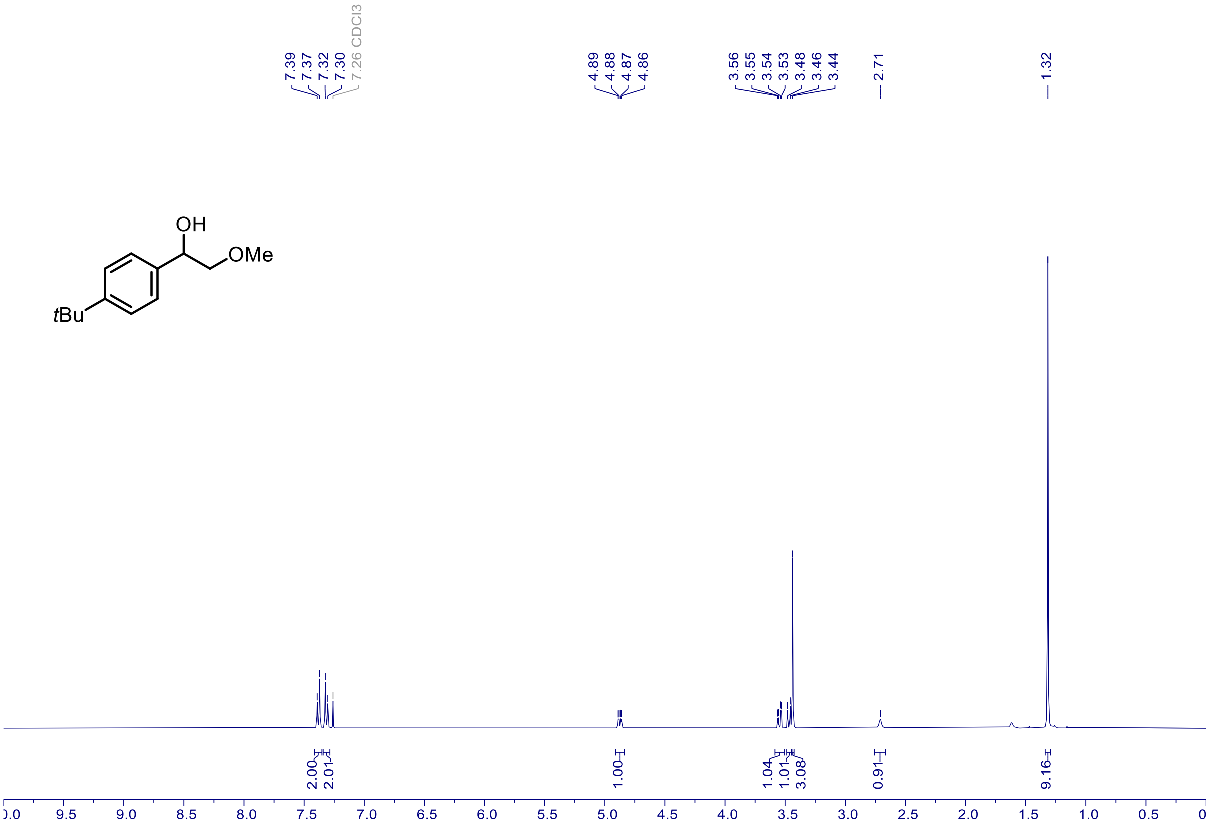

4f – ^{13}C NMR (101 MHz, $CDCl_3$)

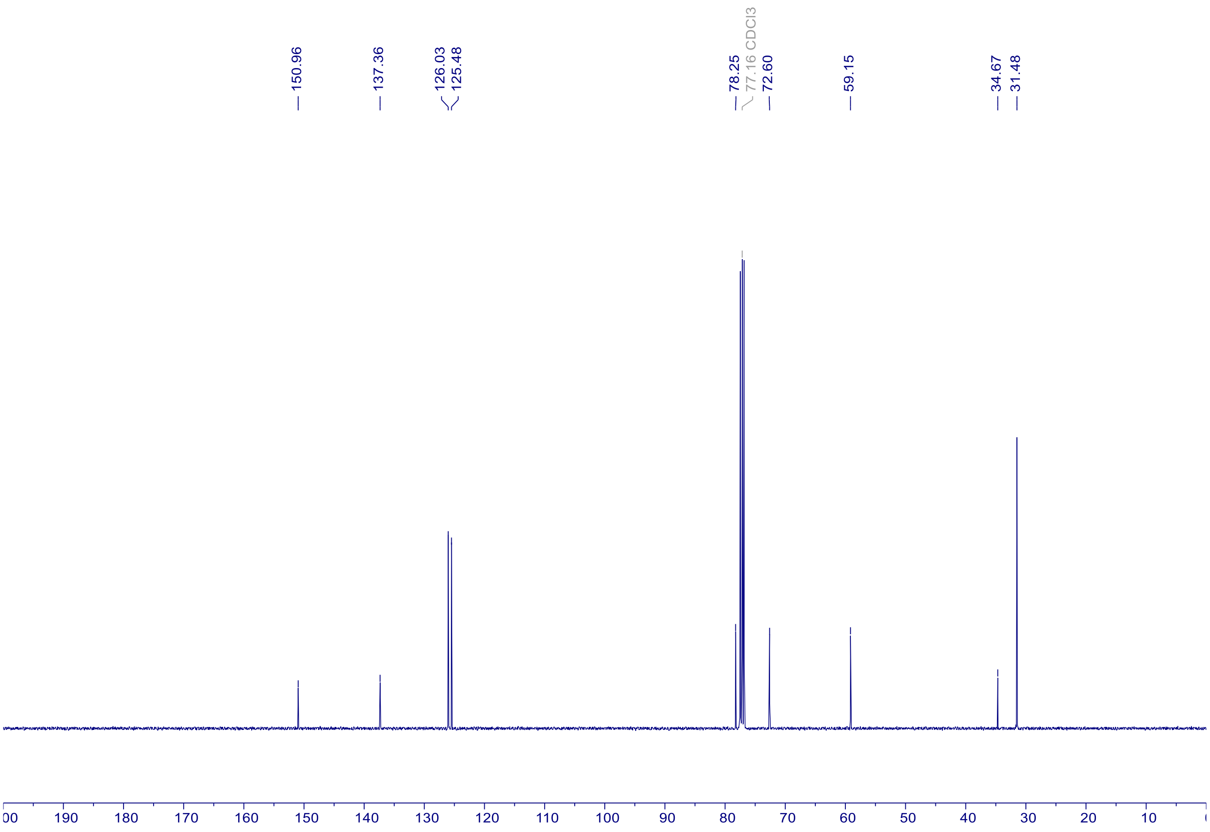

4g – ^{1}H NMR (400 MHz, $CDCl_3$)

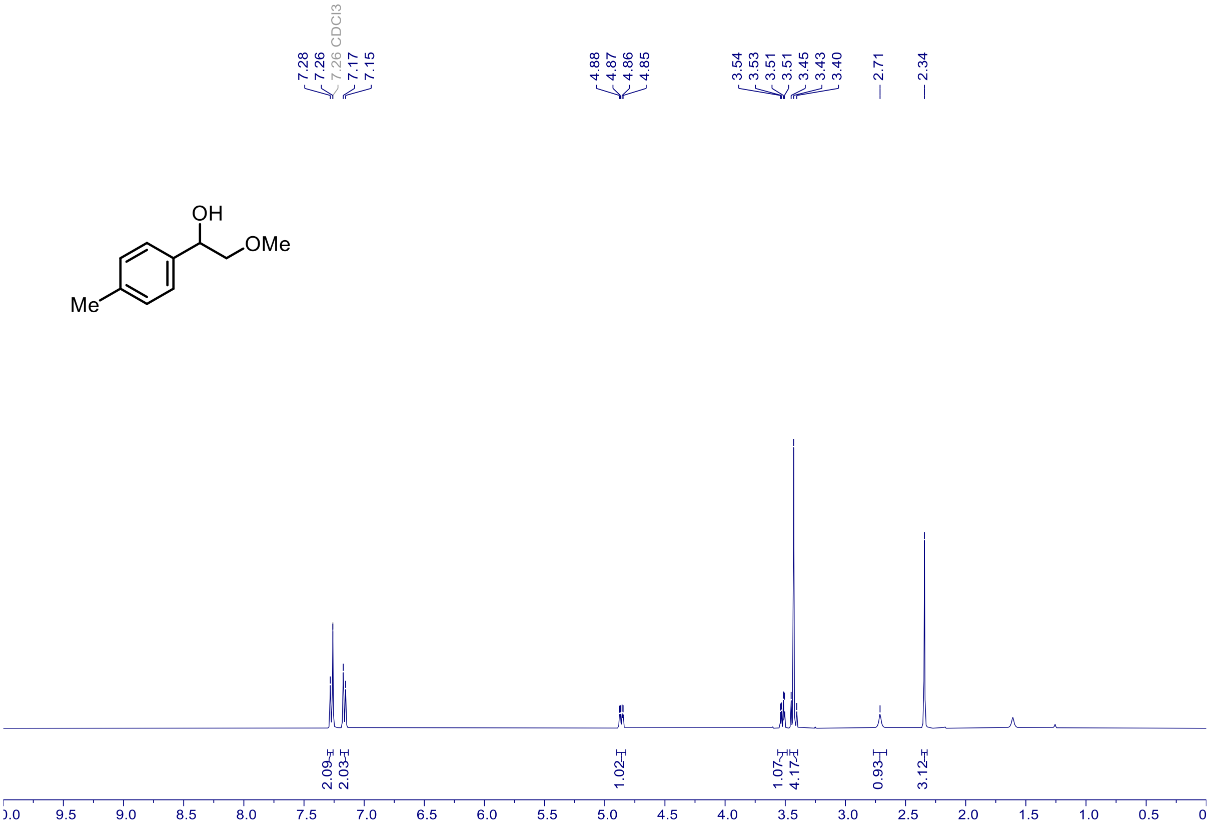

4g – ^{13}C NMR (101 MHz, $CDCl_3$)

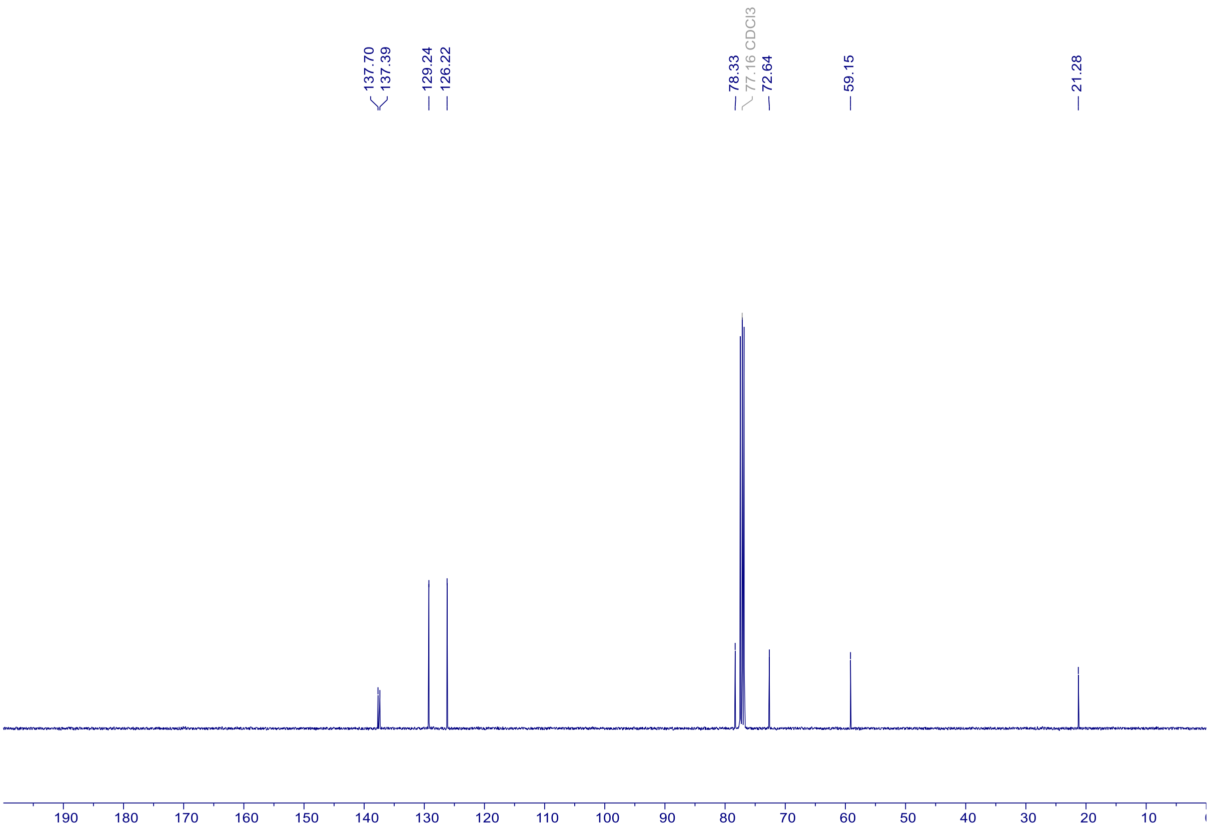

4h – ^{1}H NMR (400 MHz, $CDCl_3$)

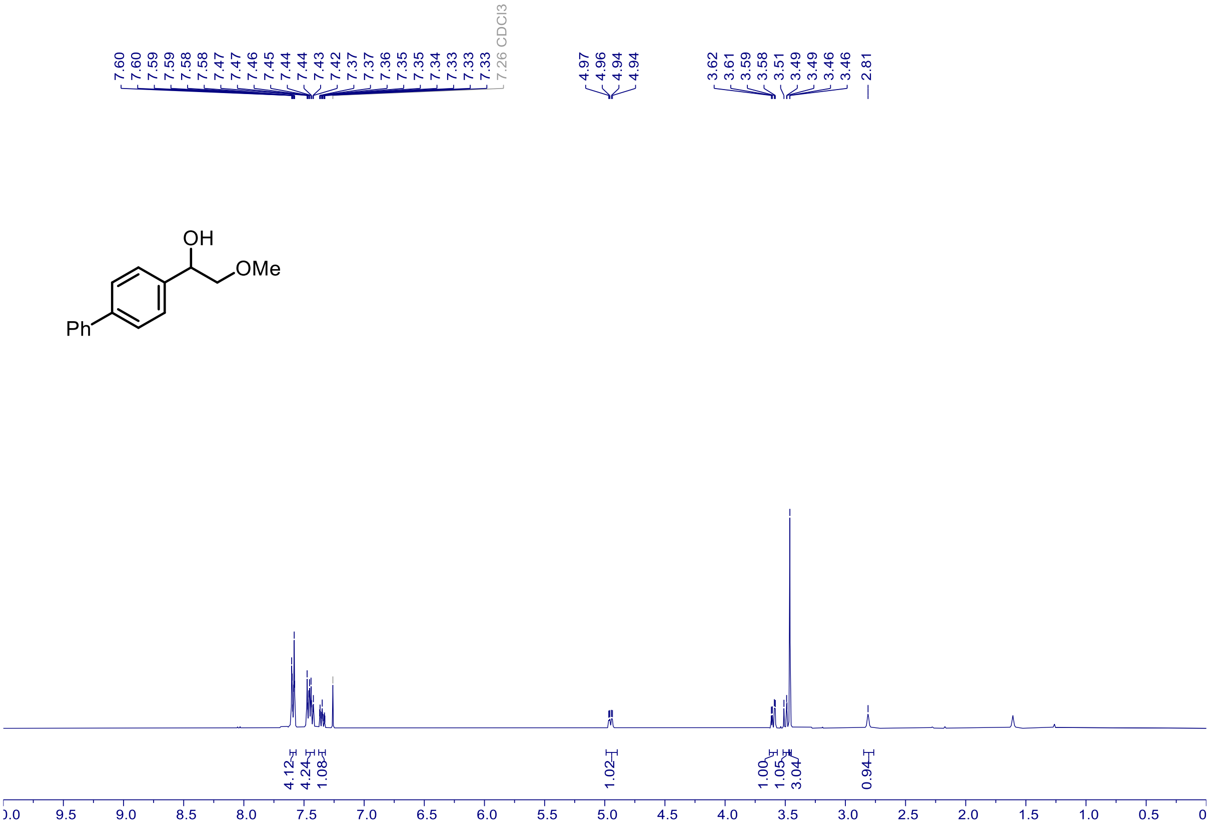

4h – ^{13}C NMR (101 MHz, $CDCl_3$)

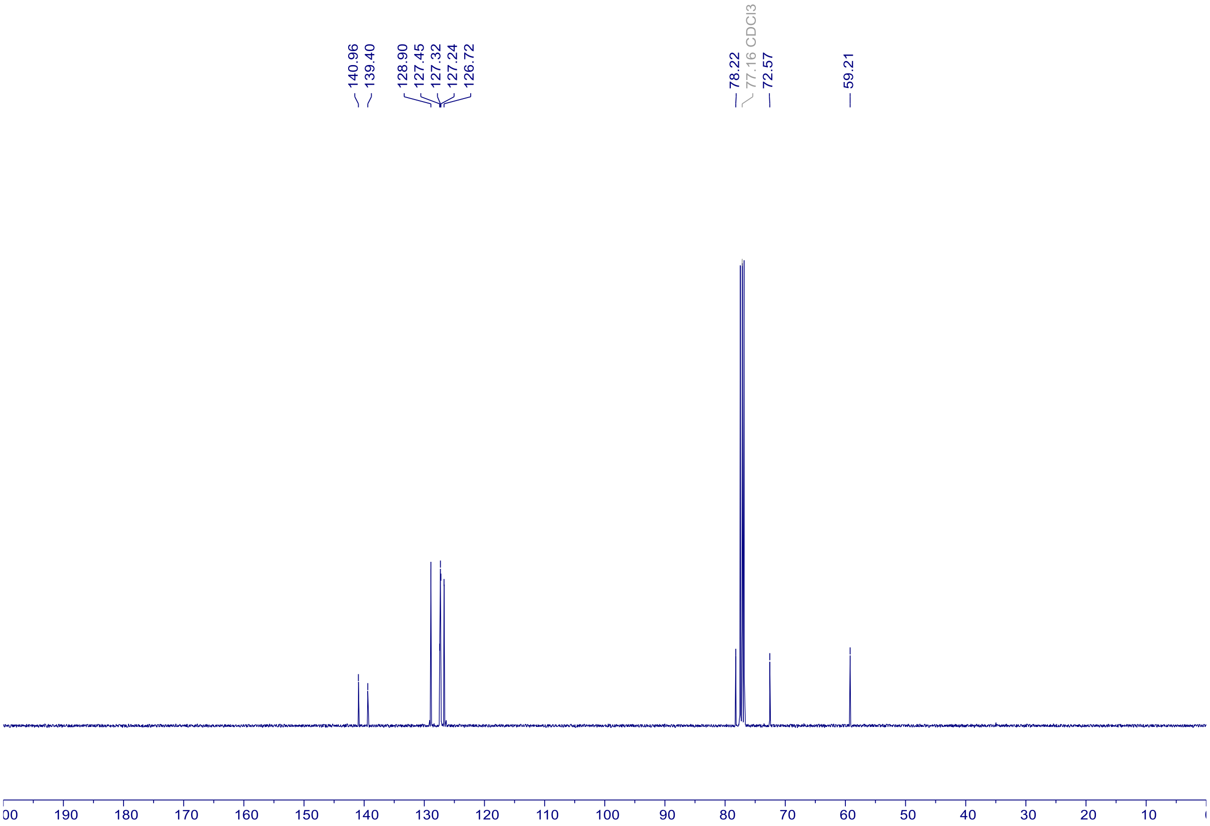

4i – ^{1}H NMR (400 MHz, $CDCl_3$)

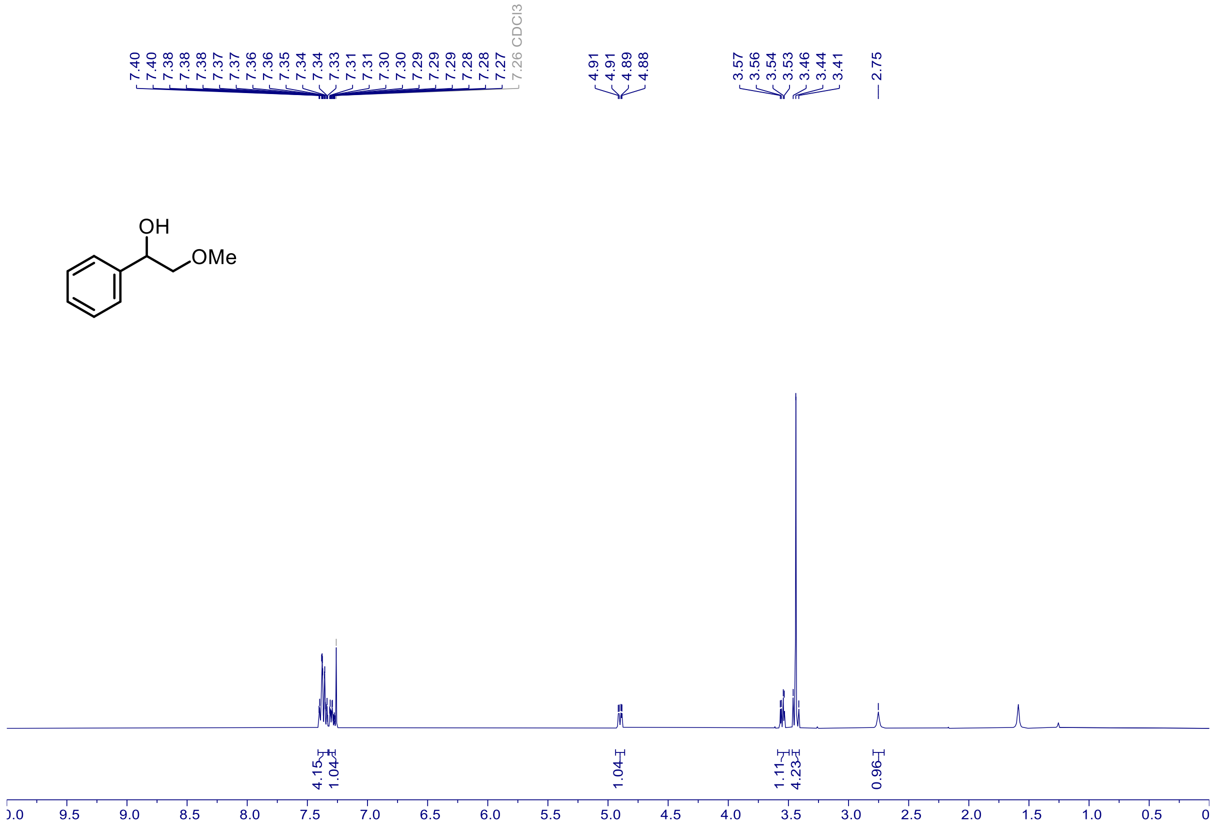

4i – ^{13}C NMR (101 MHz, $CDCl_3$)

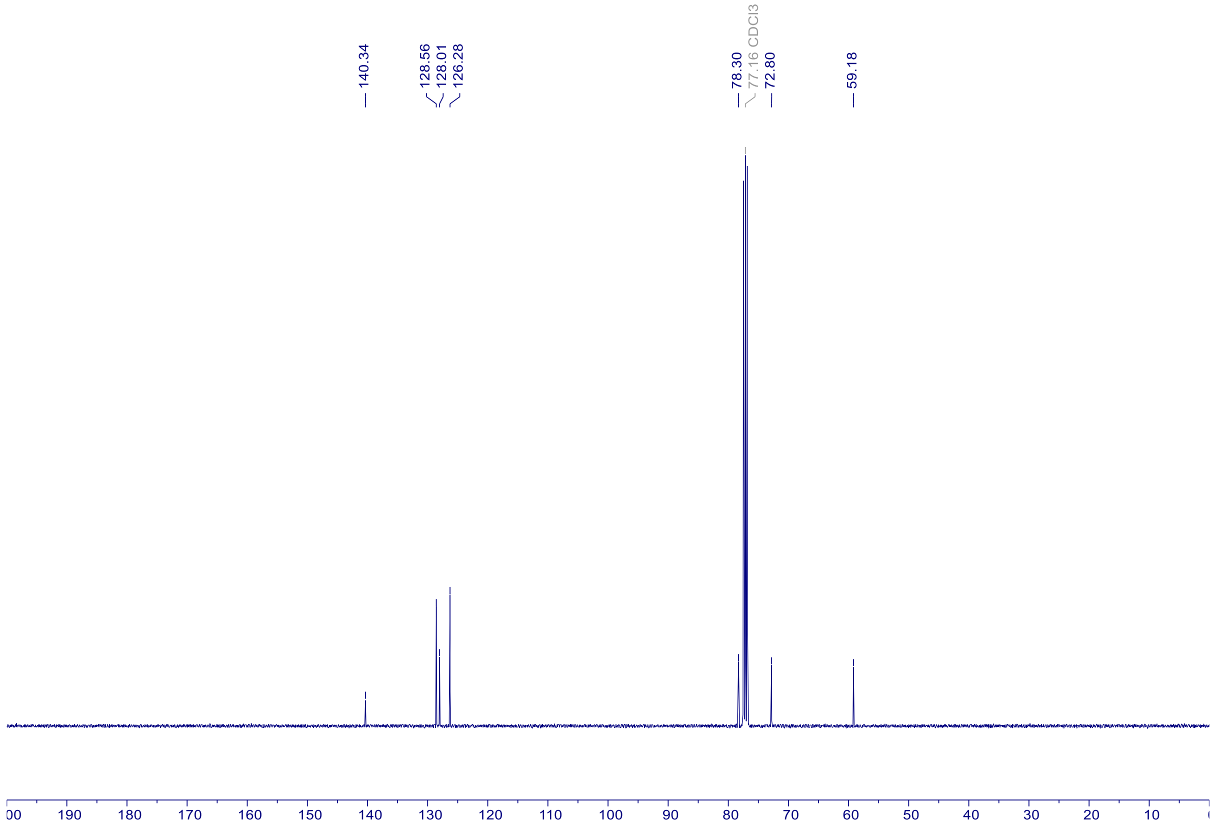

4j – ^{1}H NMR (400 MHz, $CDCl_3$)

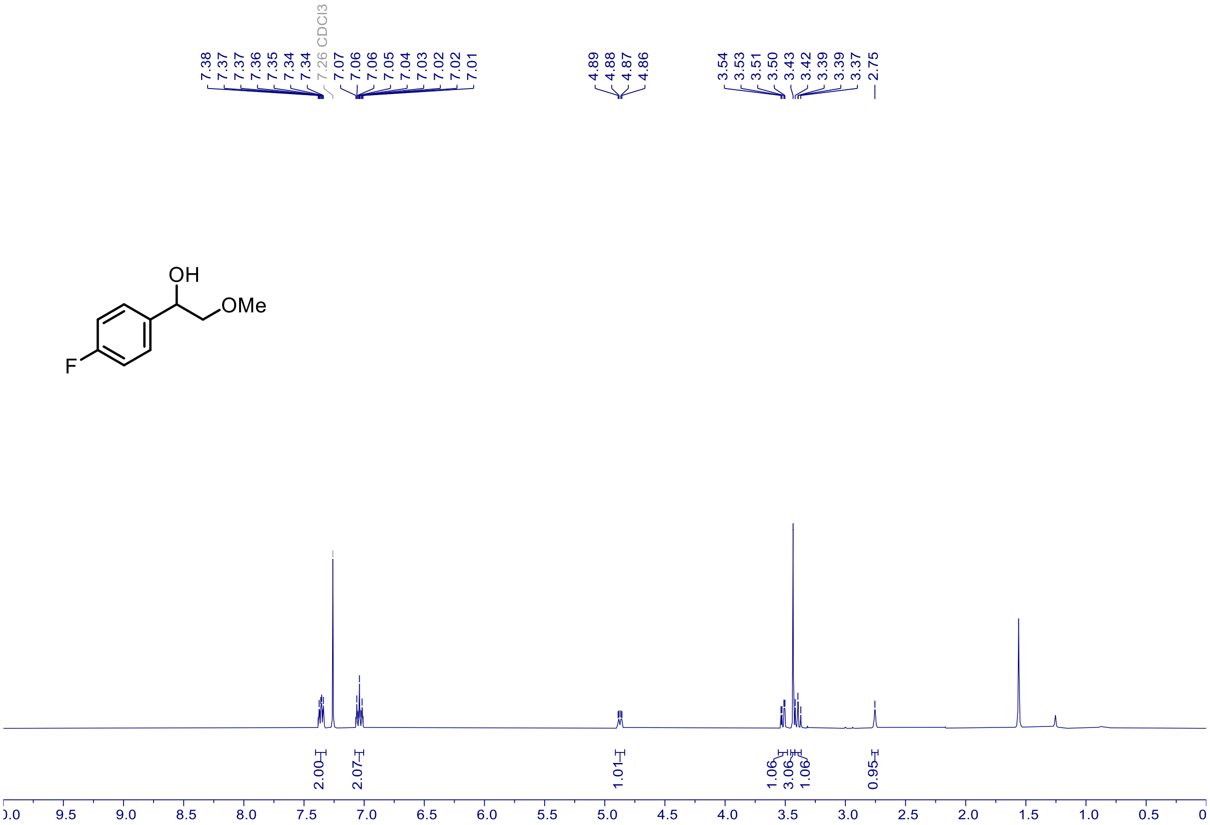

4j – ^{13}C NMR (101 MHz, $CDCl_3$)

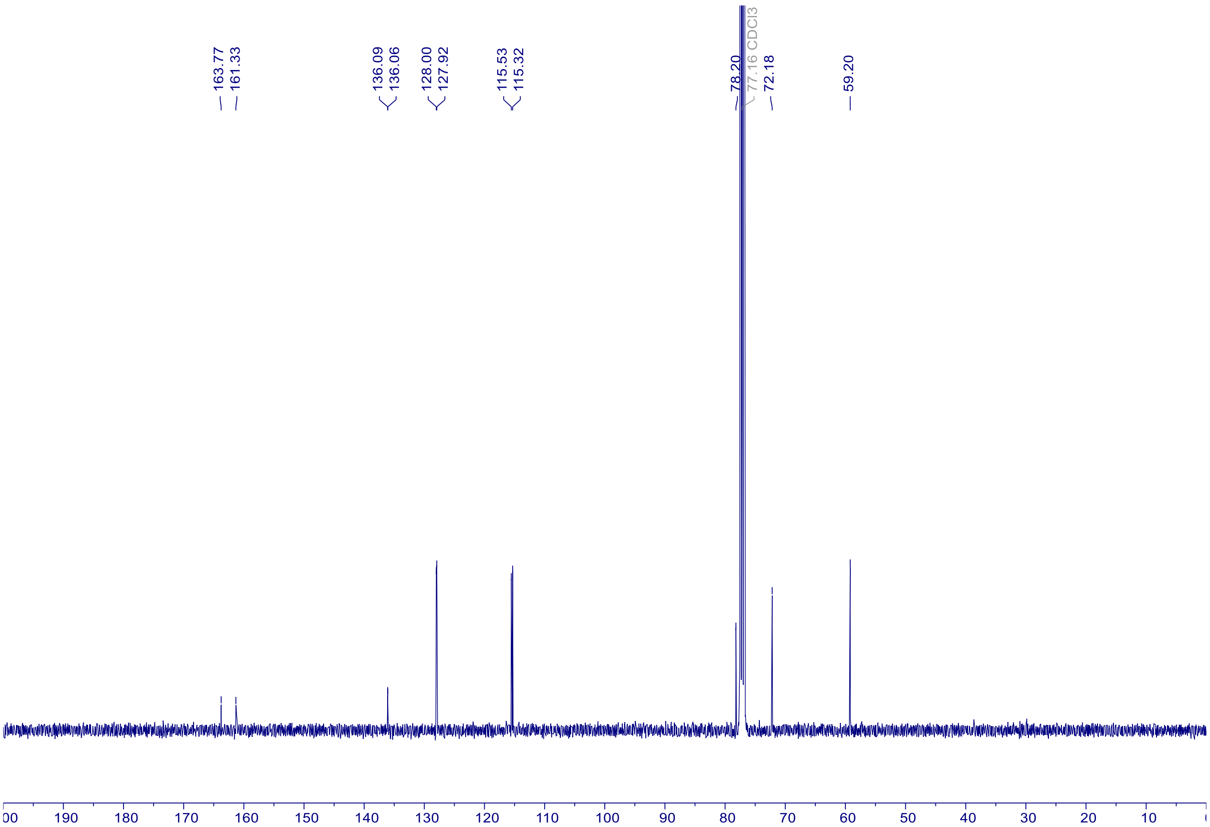

4j – ^{19}F NMR (376 MHz, $CDCl_3$)

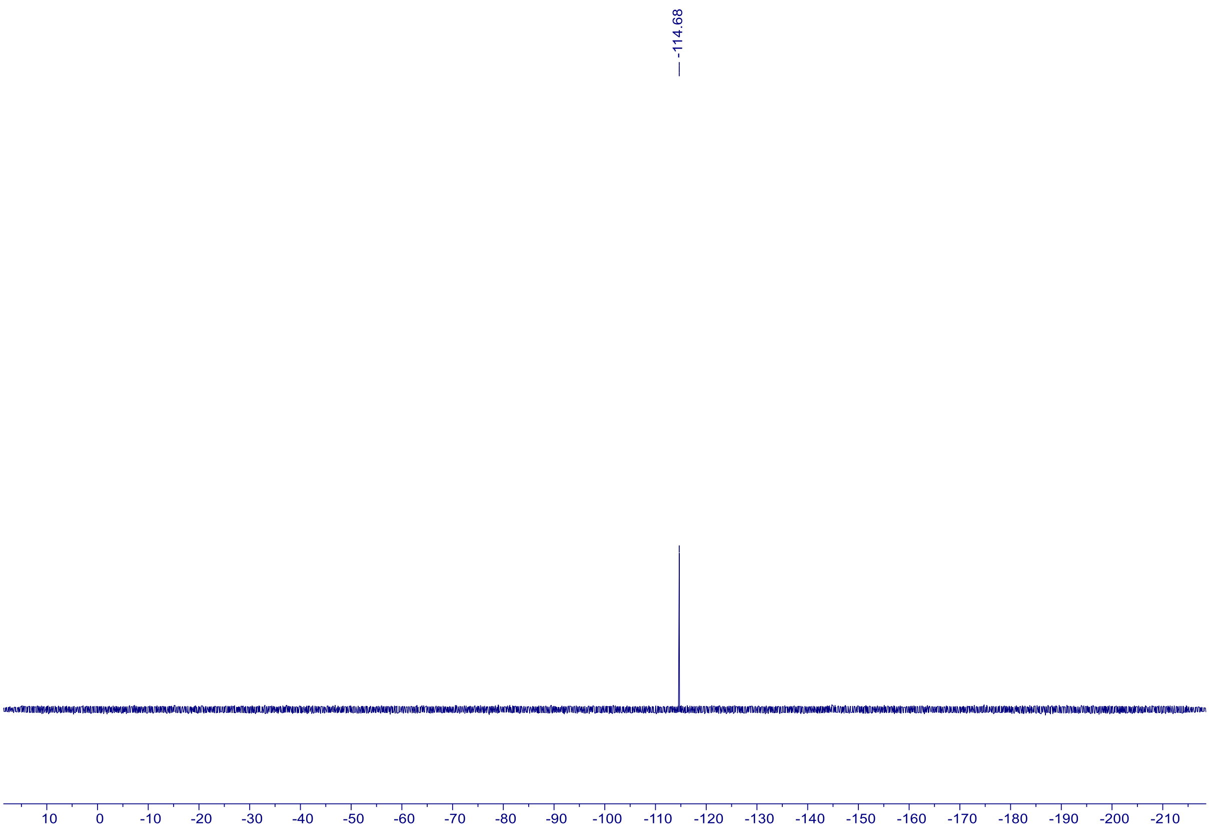

4k – ^{1}H NMR (500 MHz, $CDCl_3$)

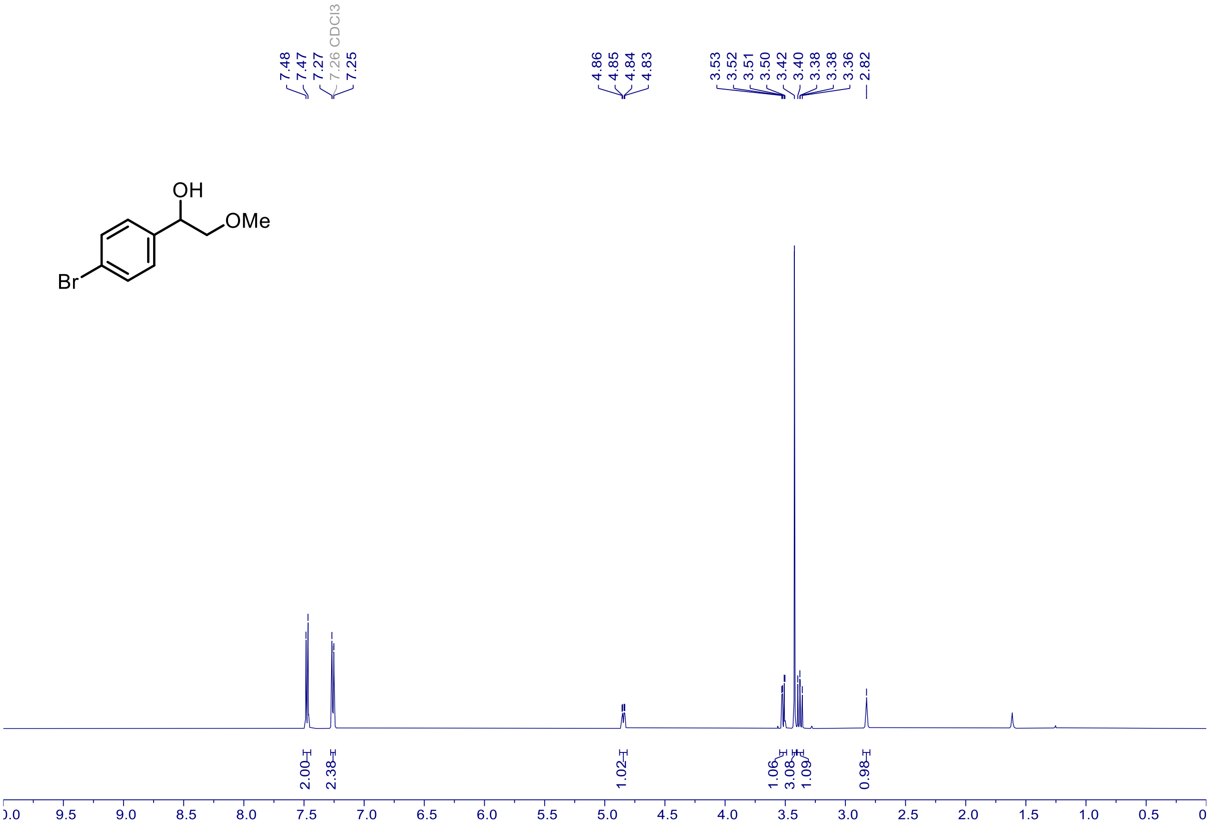

4k – ^{13}C NMR (126 MHz, $CDCl_3$)

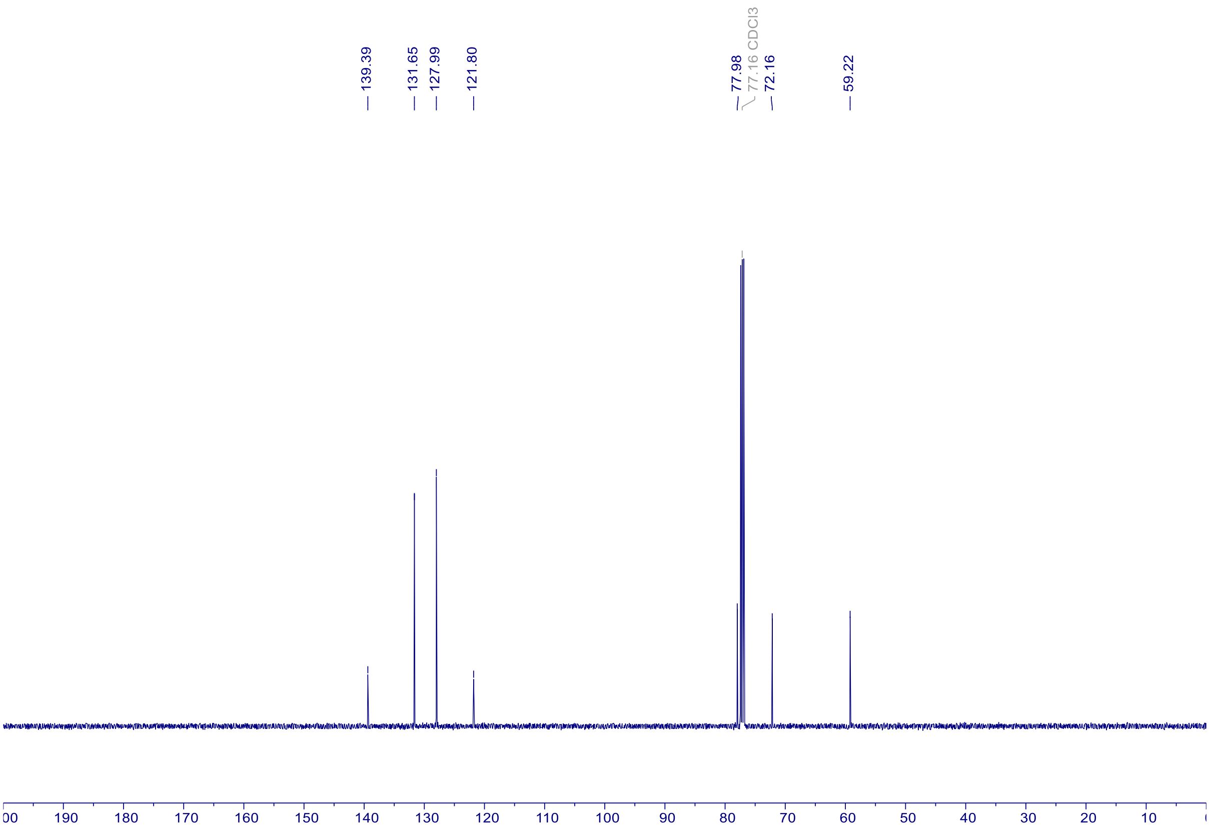

4l – ^{1}H NMR (400 MHz, $CDCl_3$)

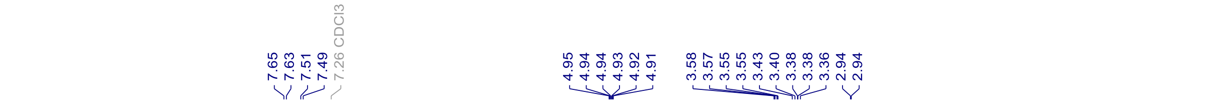

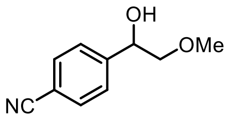

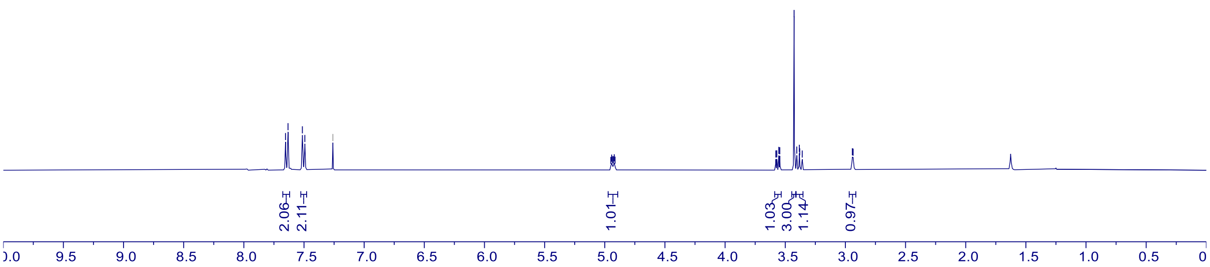

4l – ^{13}C NMR (101 MHz, $CDCl_3$)

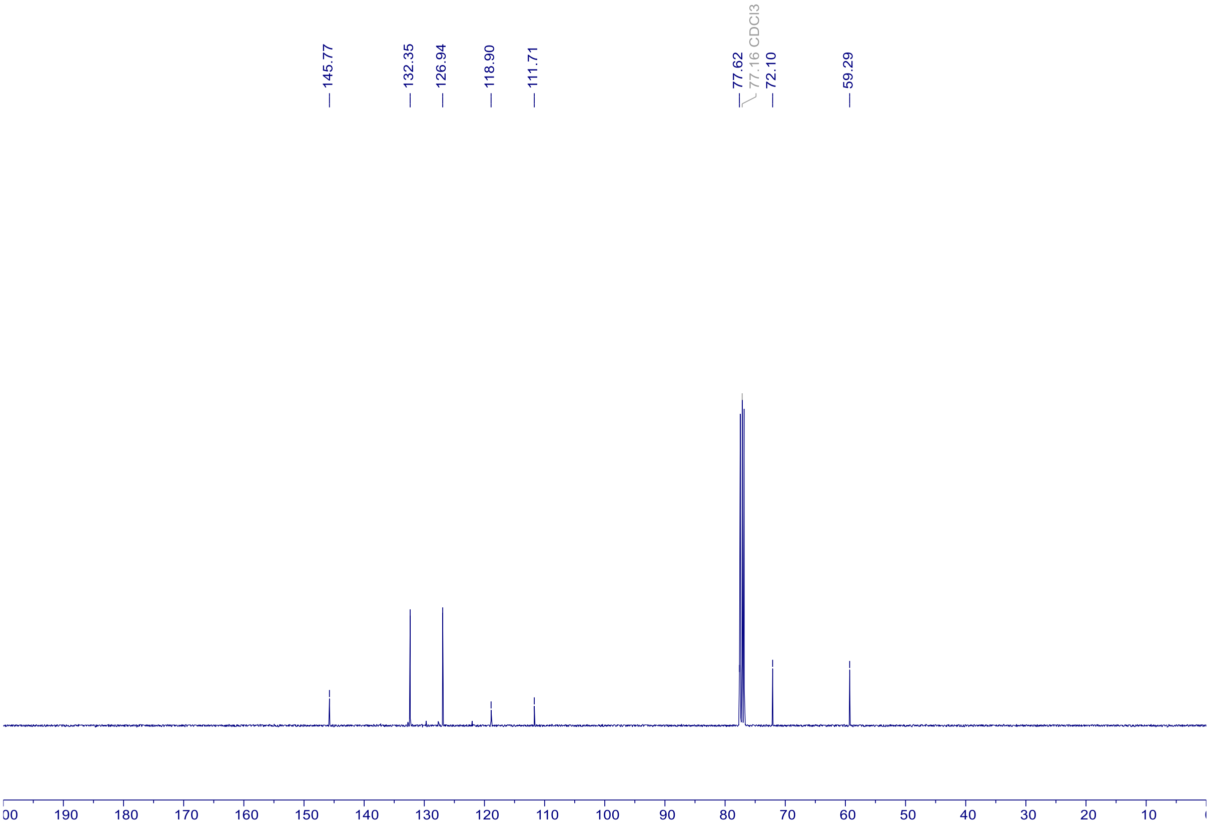

4m – ^{1}H NMR (500 MHz, CDCl$_3$)

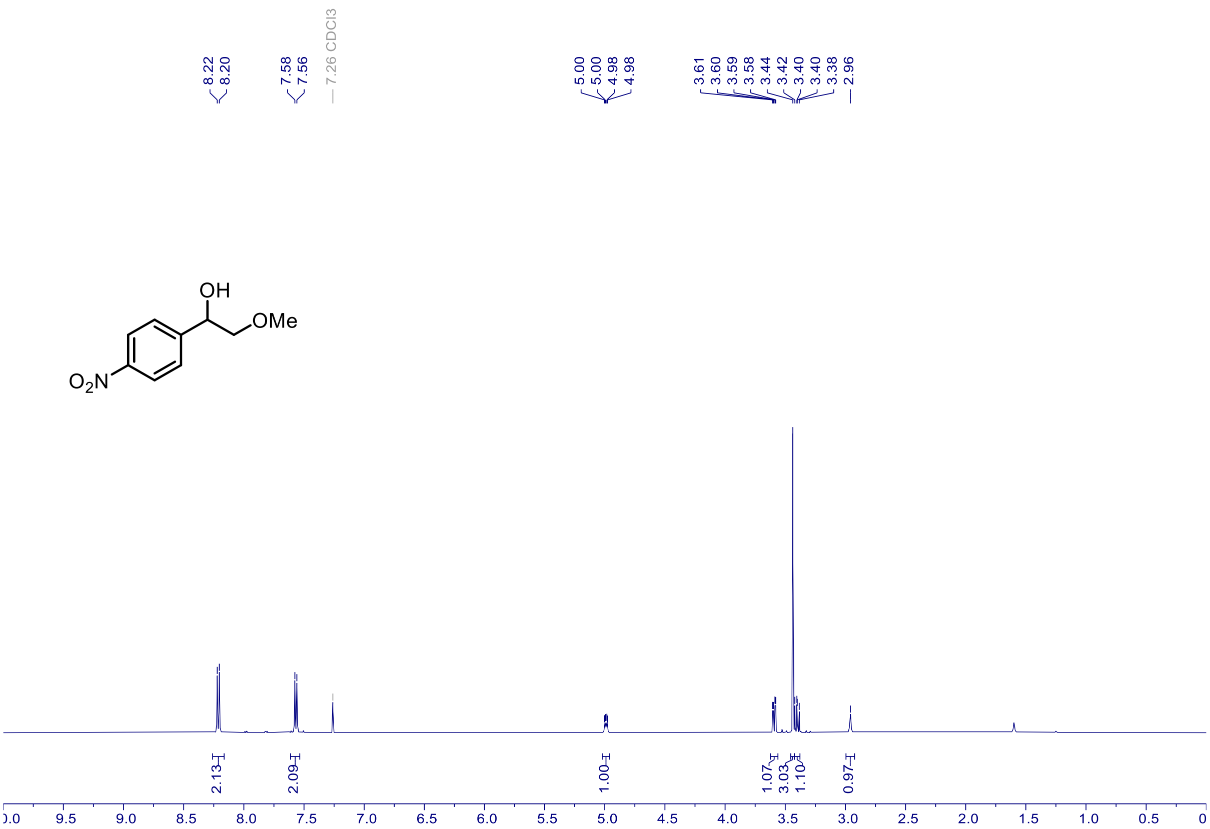

4m – ^{13}C NMR (126 MHz, CDCl$_3$)

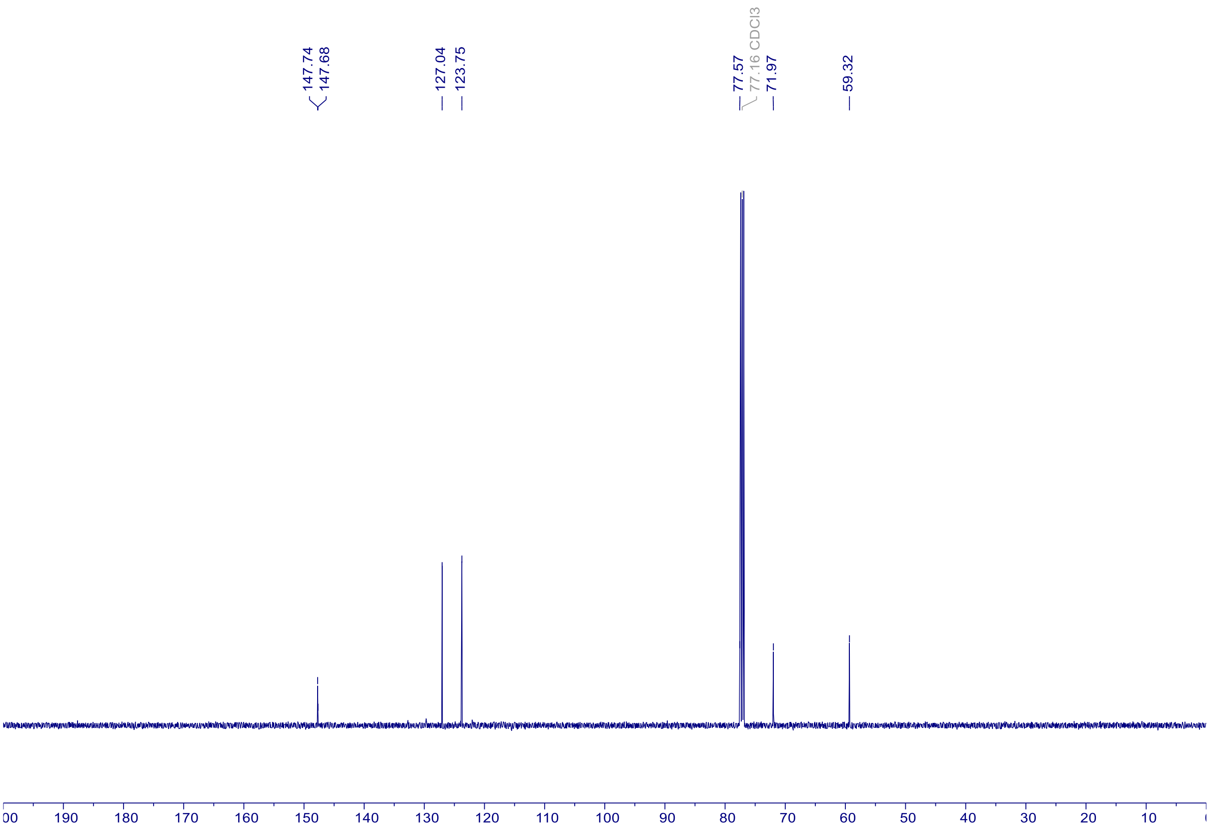

4n – ^{1}H NMR (400 MHz, $CDCl_3$)

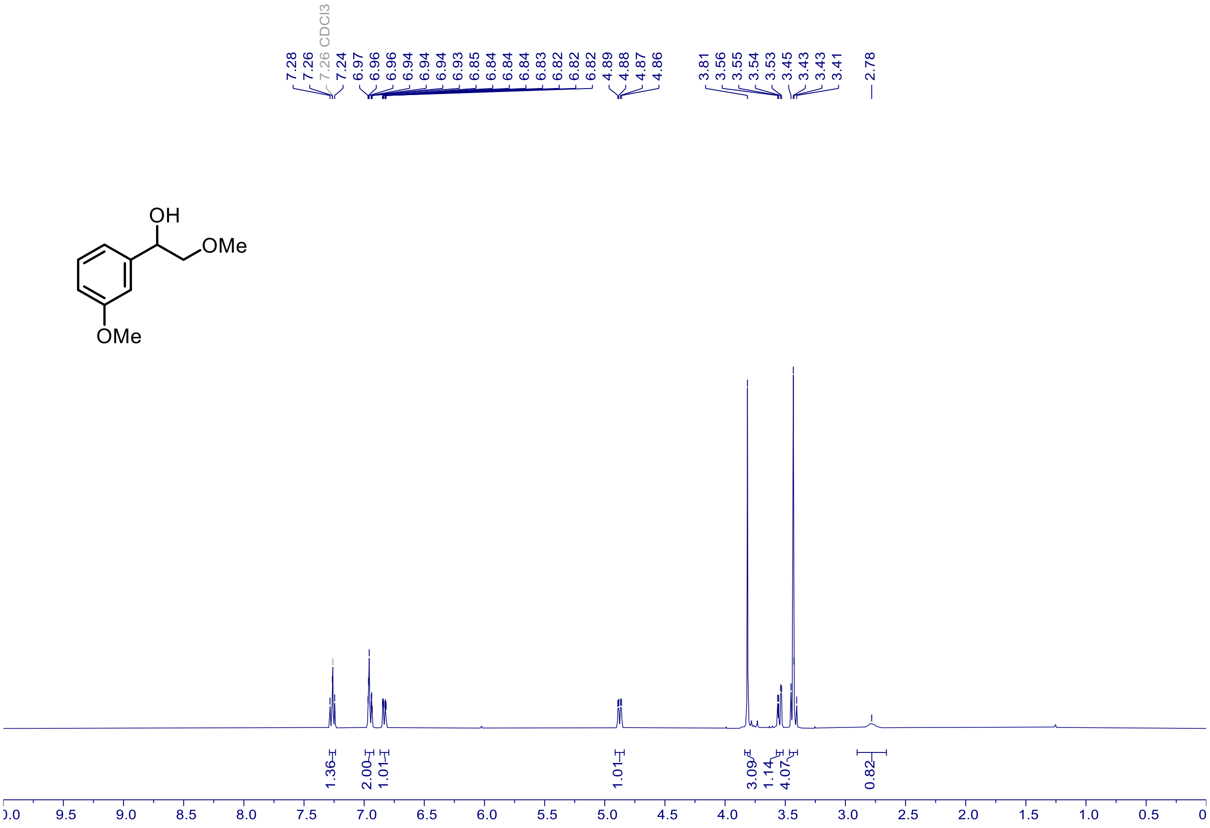

4n – ^{13}C NMR (101 MHz, $CDCl_3$)

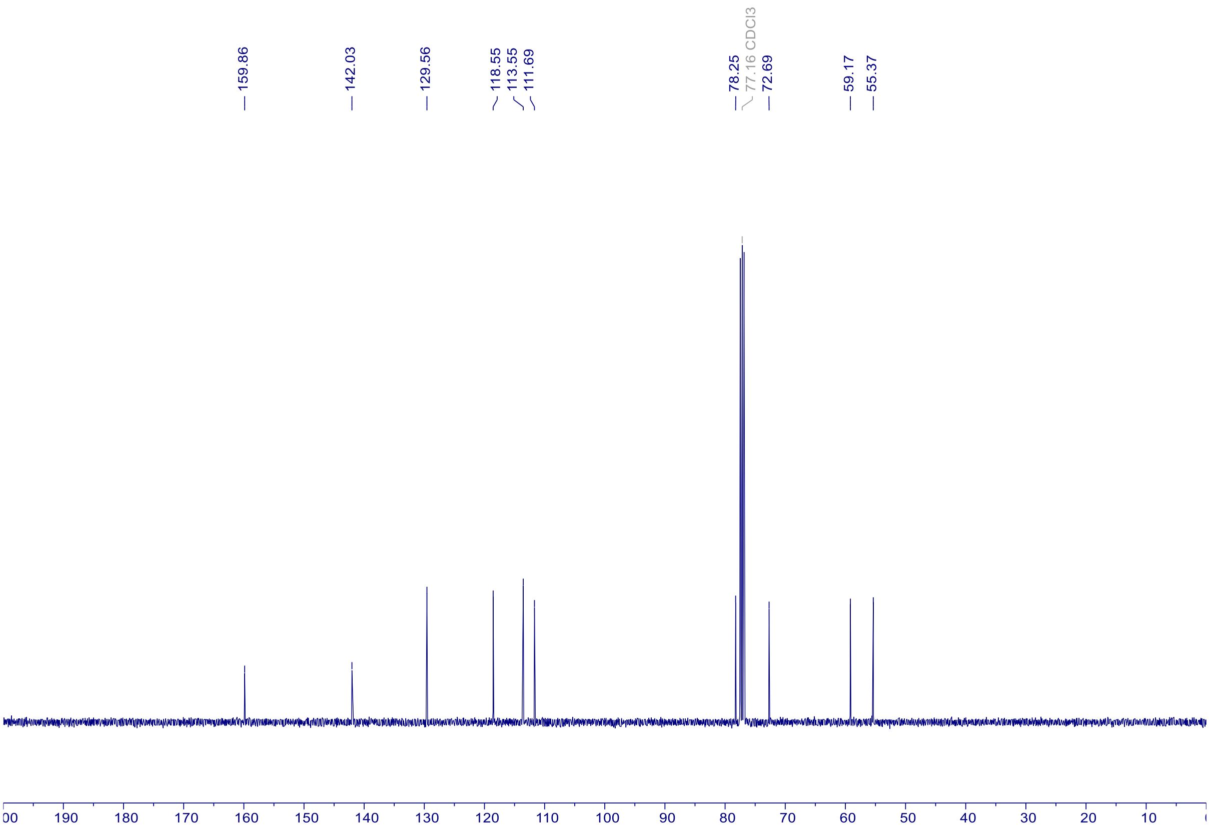

4o – ^{1}H NMR (400 MHz, $CDCl_3$)

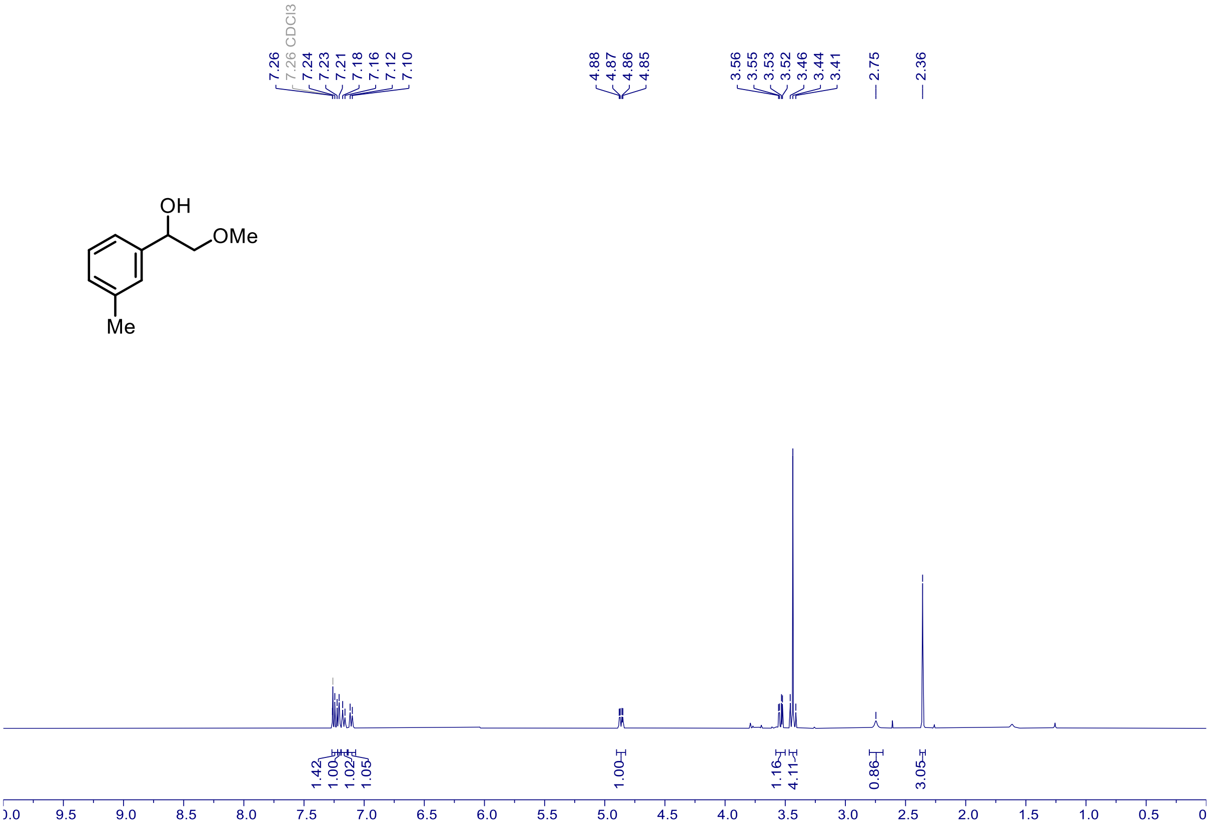

4o – ^{13}C NMR (101 MHz, $CDCl_3$)

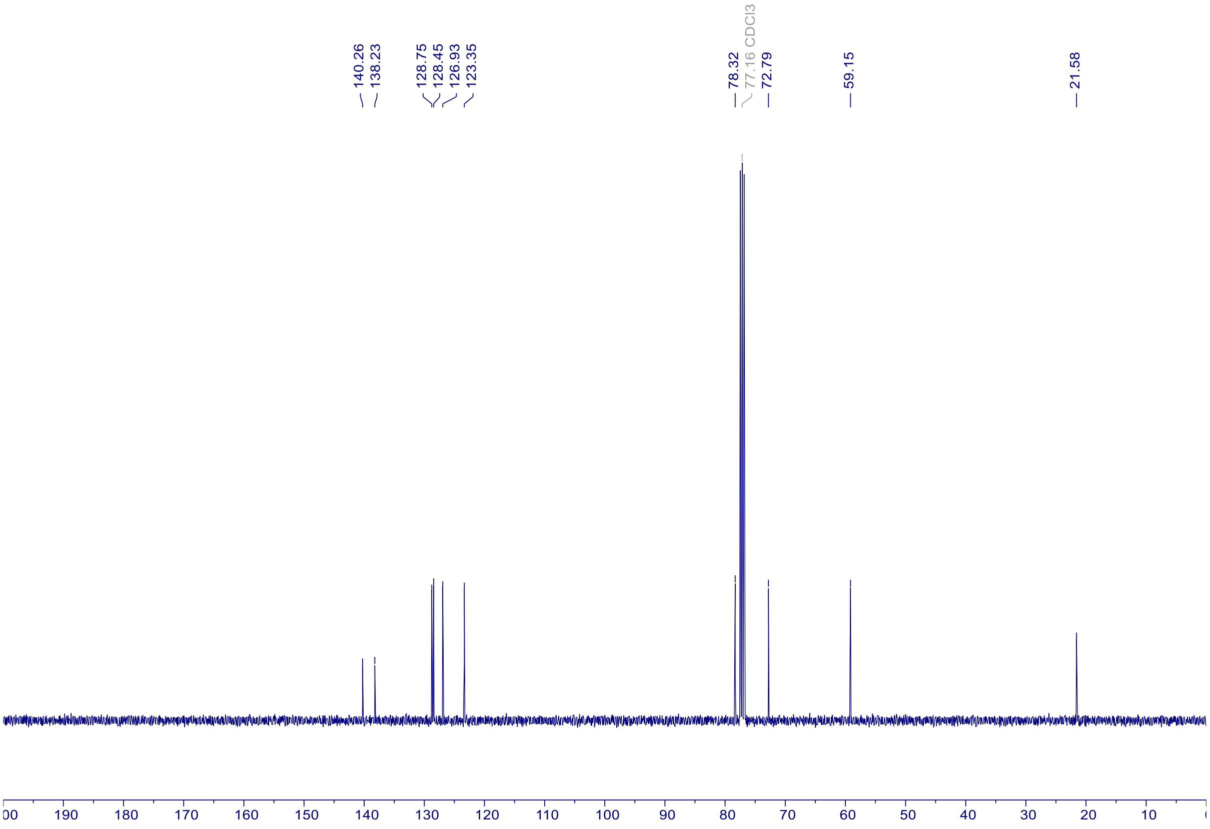

4p – ^{1}H NMR (400 MHz, $CDCl_3$)

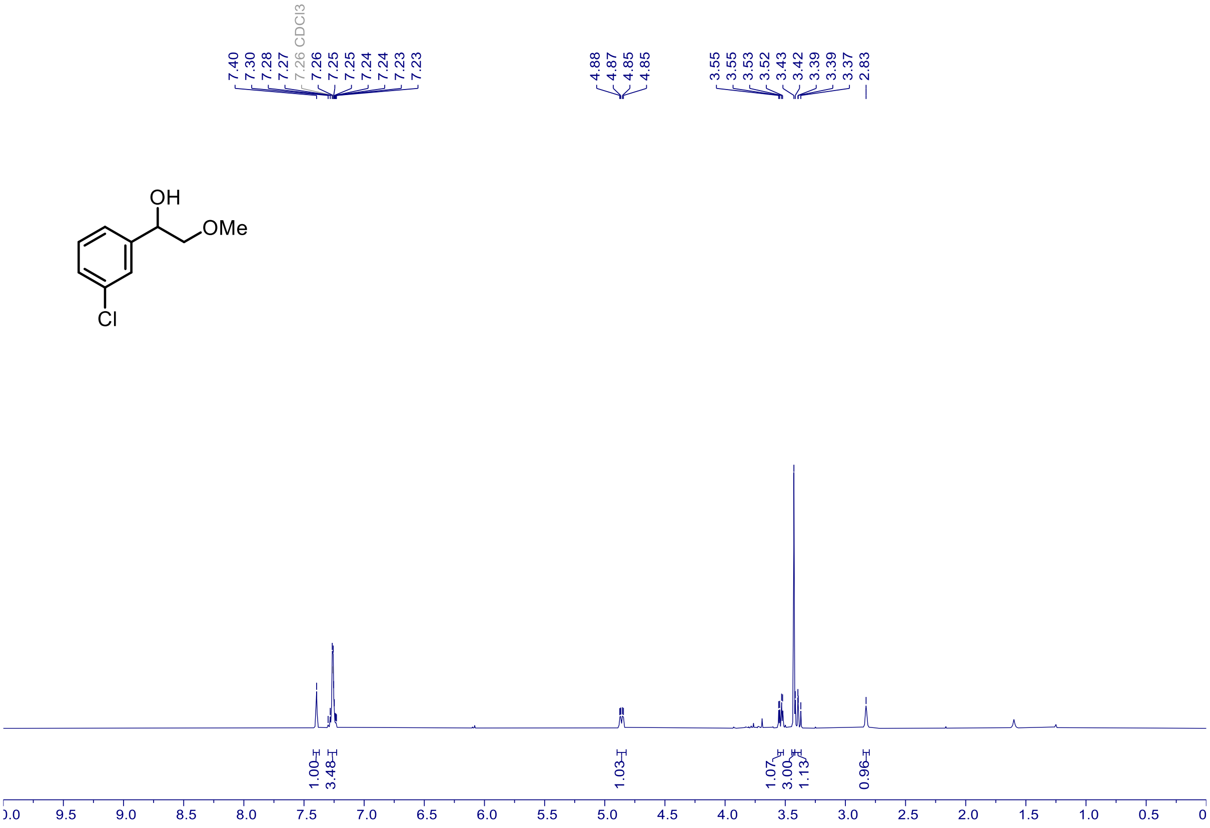

4p – ^{13}C NMR (101 MHz, $CDCl_3$)

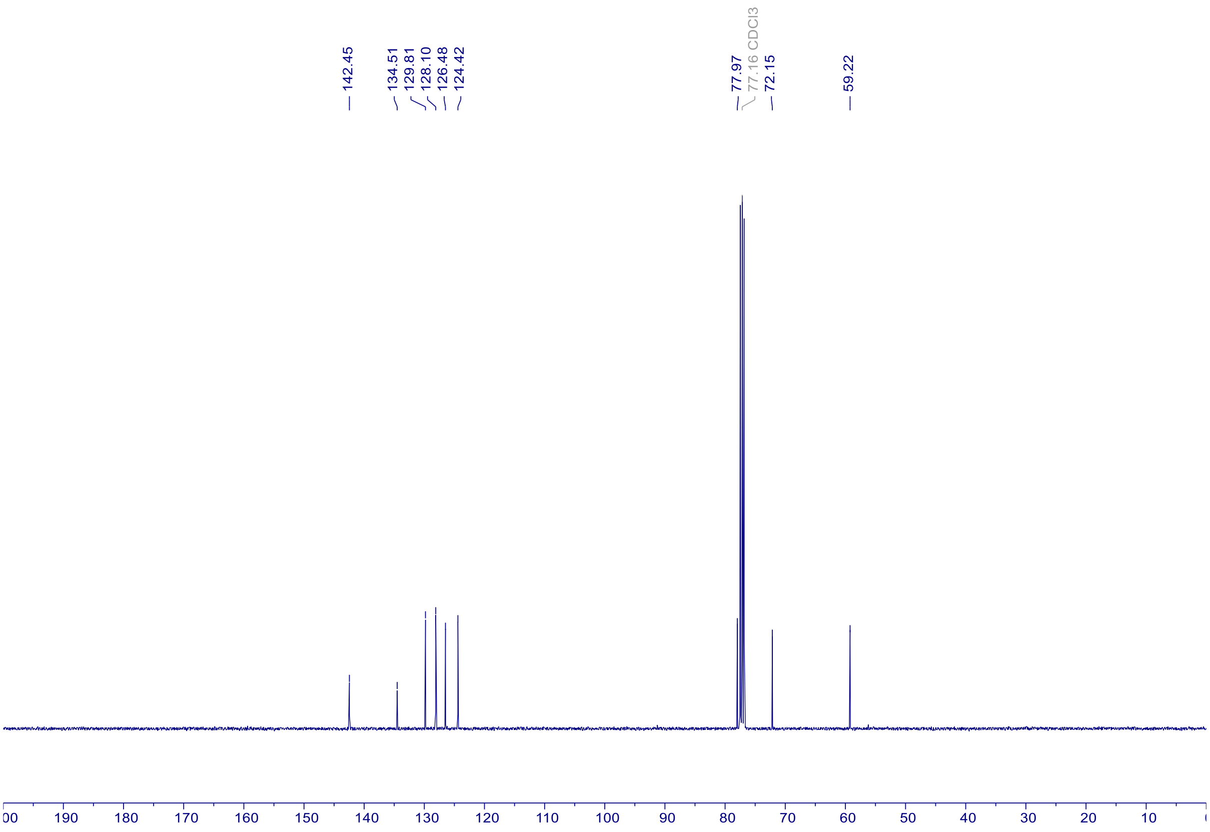

4q – ^{1}H NMR (400 MHz, $CDCl_3$)

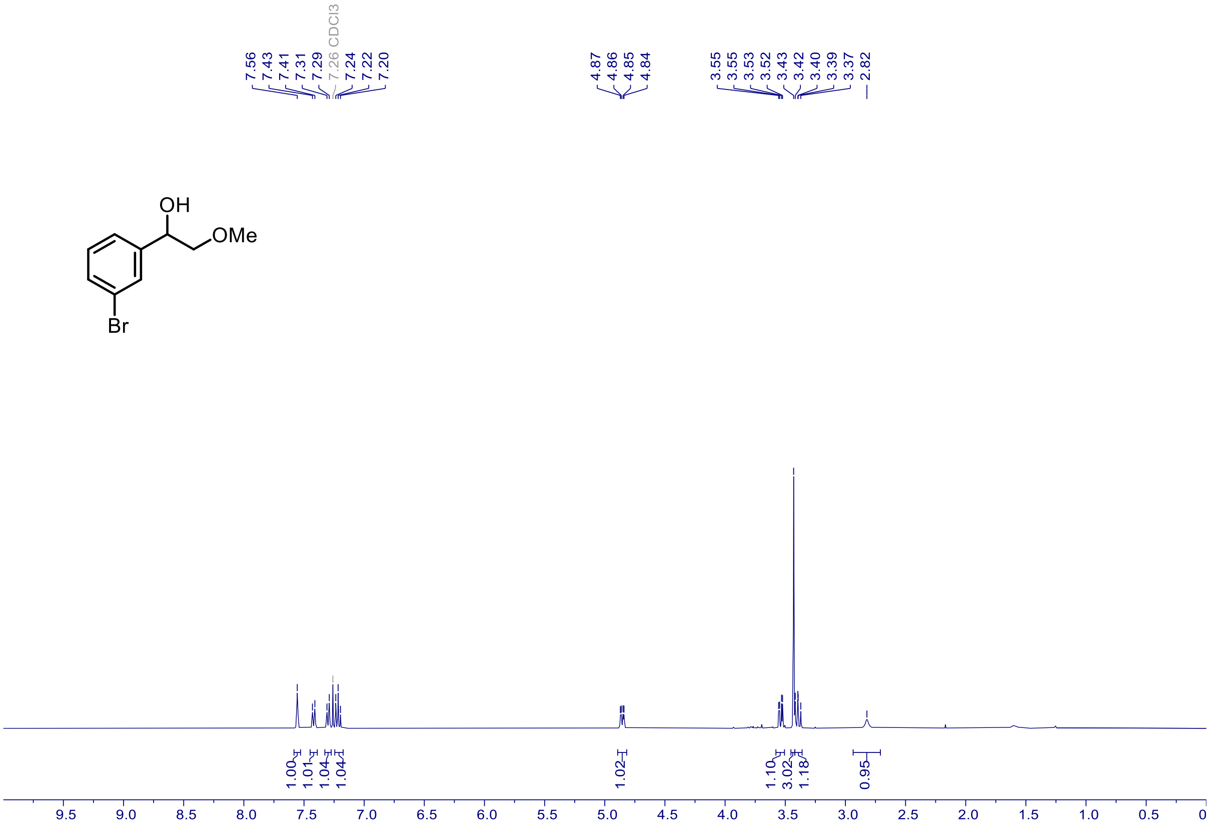

4q – ^{13}C NMR (101 MHz, $CDCl_3$)

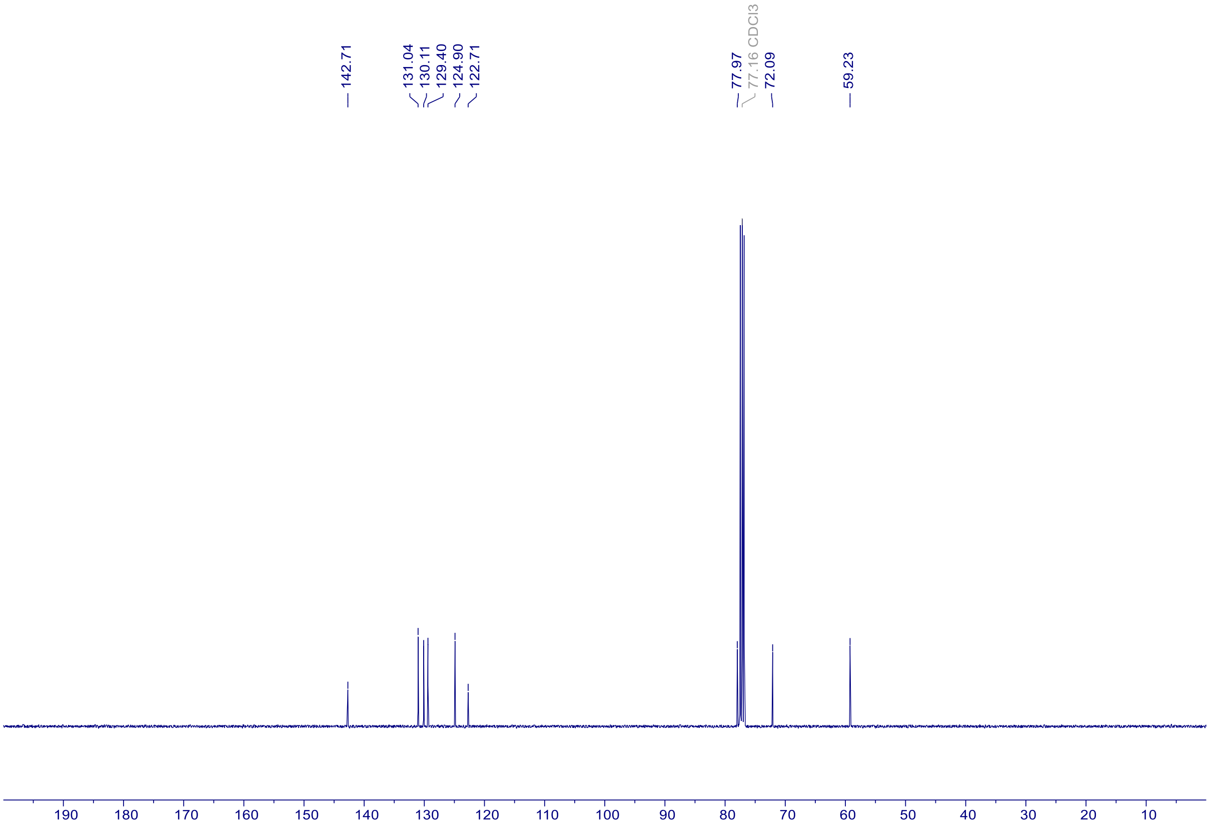

4r – ^{1}H NMR (400 MHz, $CDCl_3$)

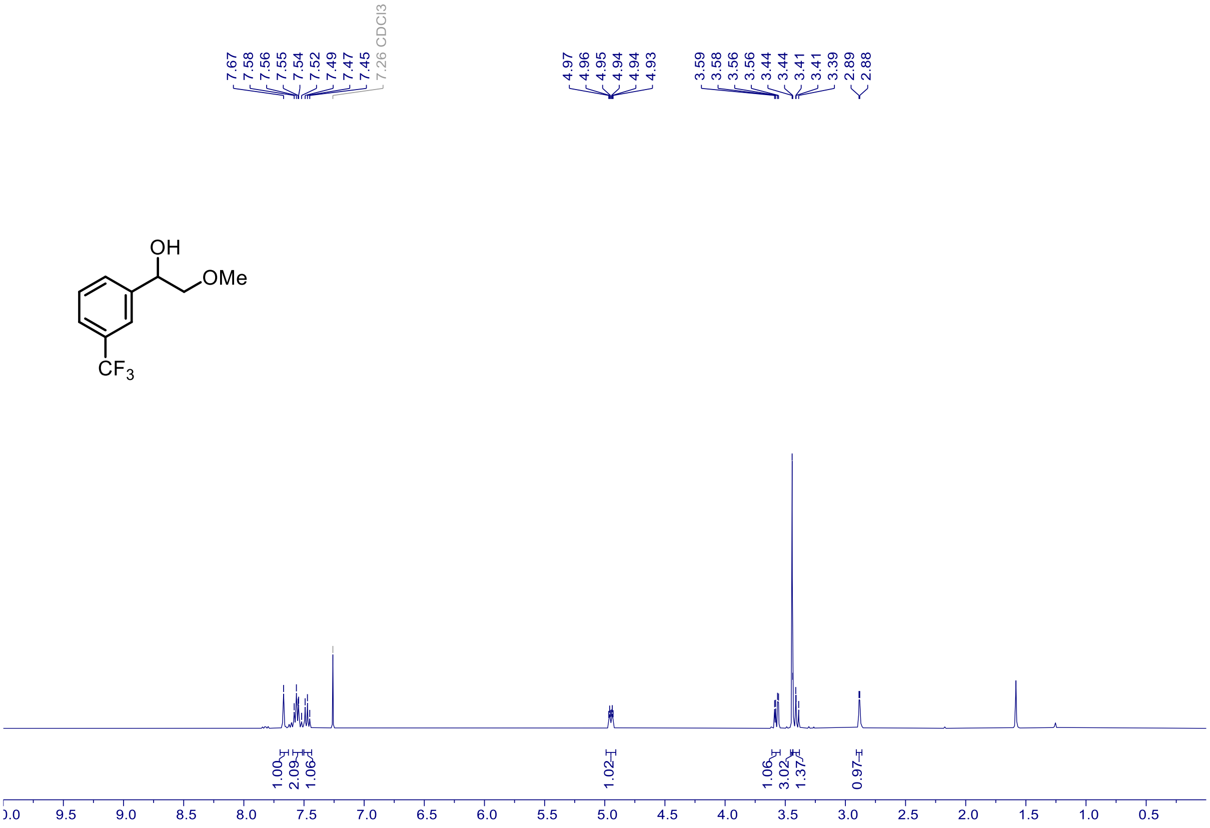

4r – ^{13}C NMR (101 MHz, $CDCl_3$)

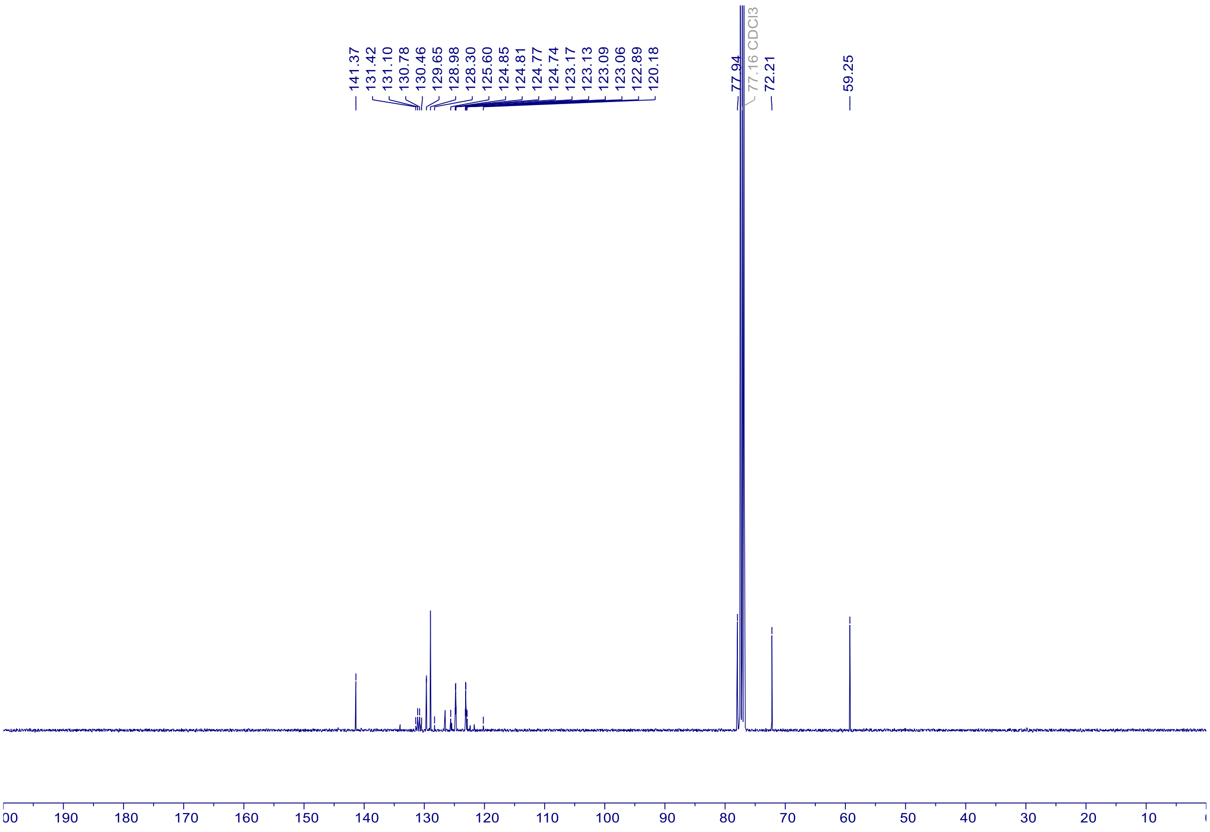

4r – ^{19}F NMR (376 MHz, $CDCl_3$)

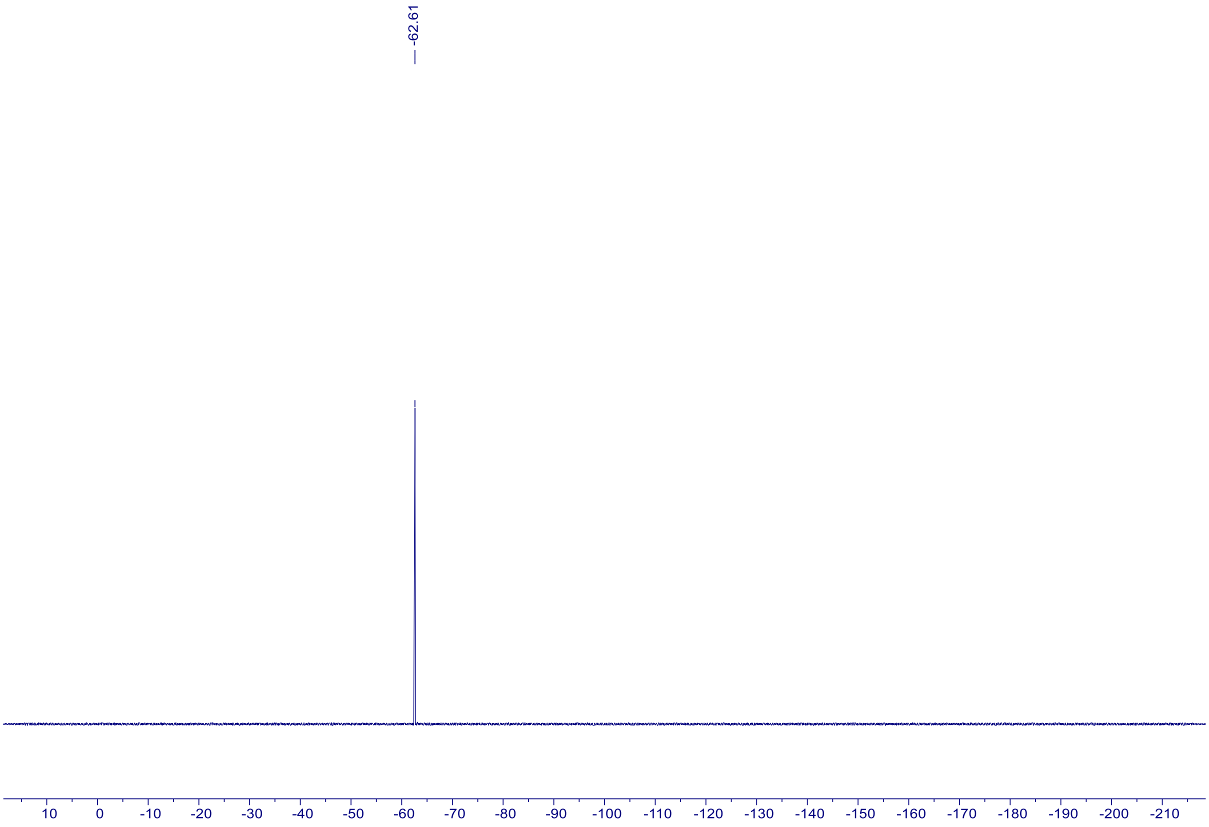

4s – ^{1}H NMR (400 MHz, $CDCl_3$)

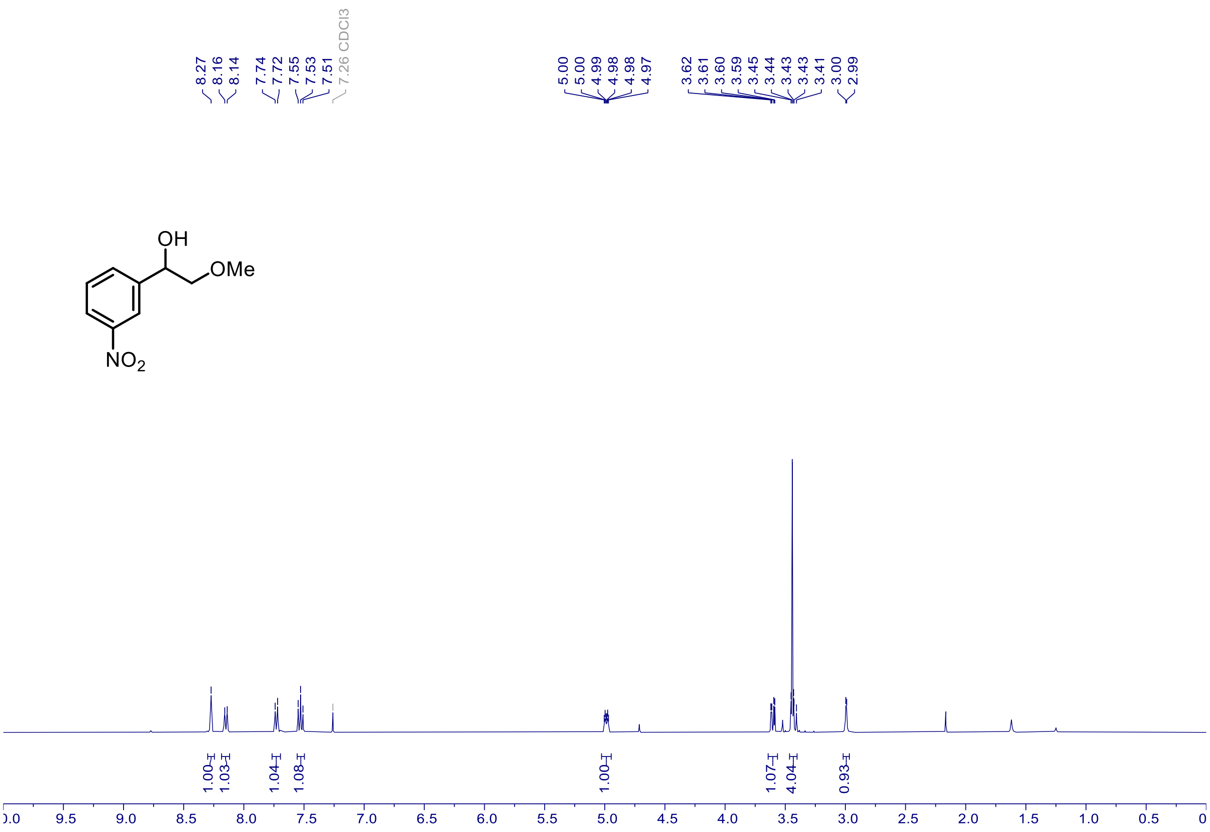

4s – ^{13}C NMR (101 MHz, $CDCl_3$)

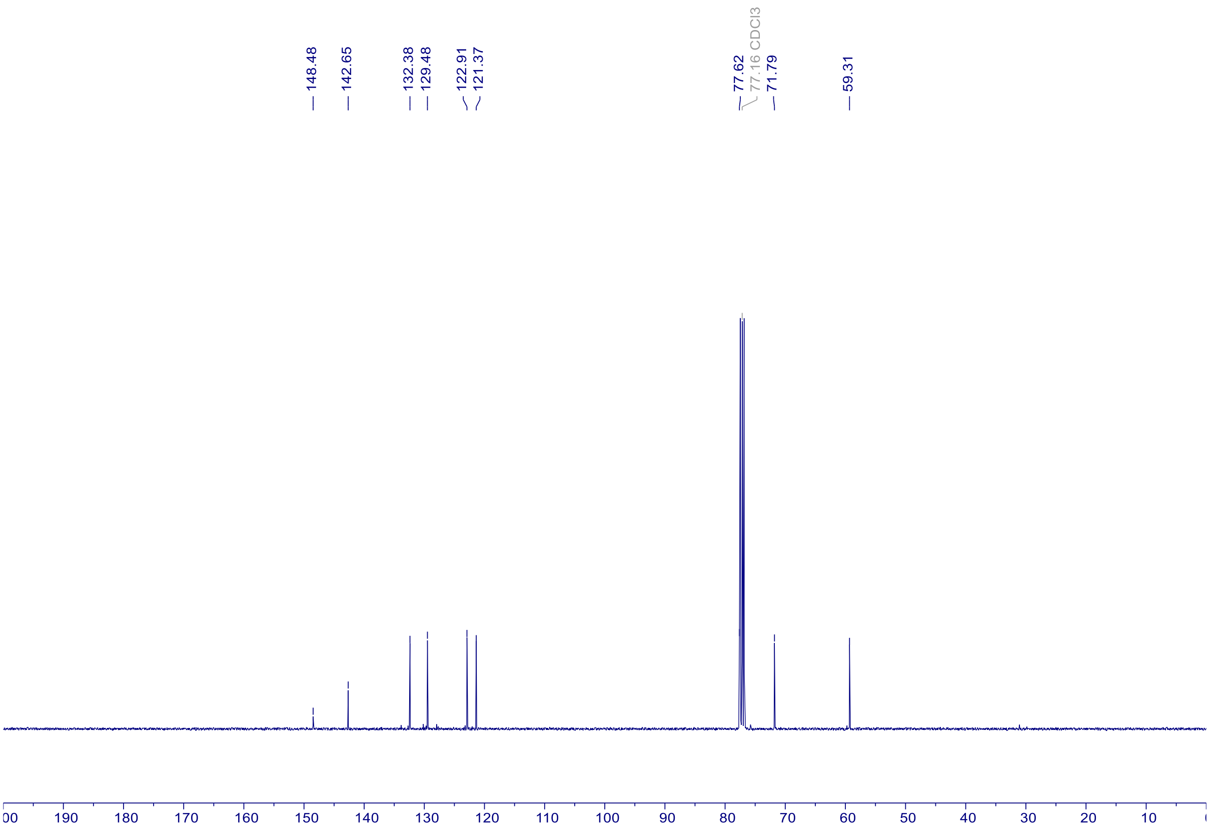

4t – ^{1}H NMR (400 MHz, $CDCl_3$)

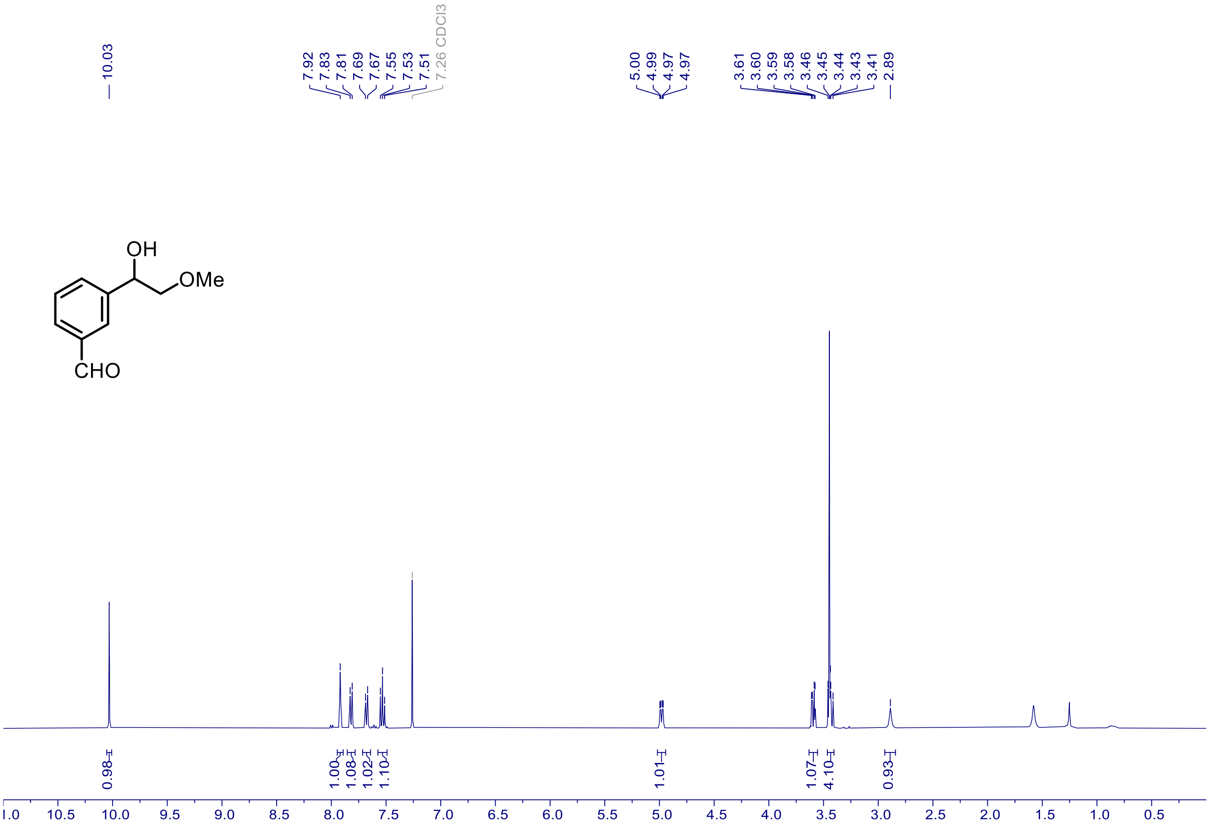

4t – ^{13}C NMR (101 MHz, $CDCl_3$)

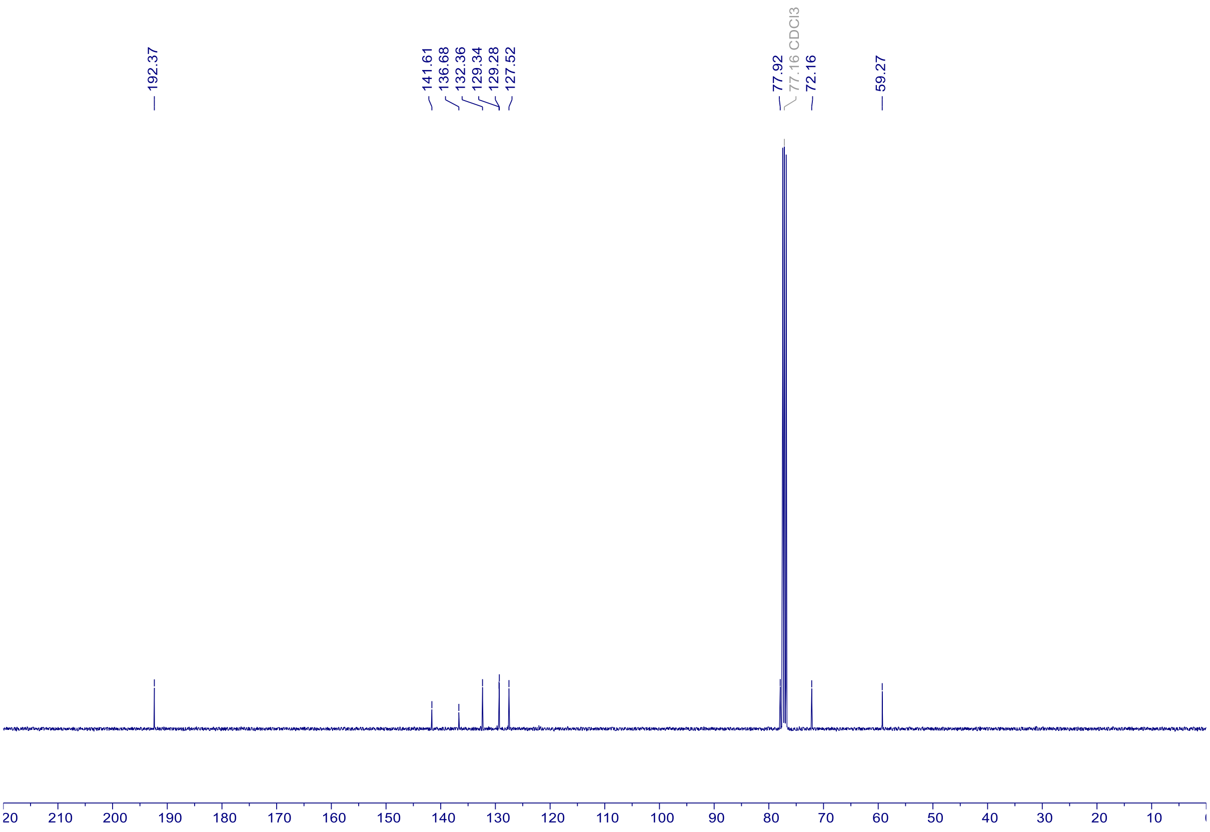

4u – ^{1}H NMR (400 MHz, $CDCl_3$)

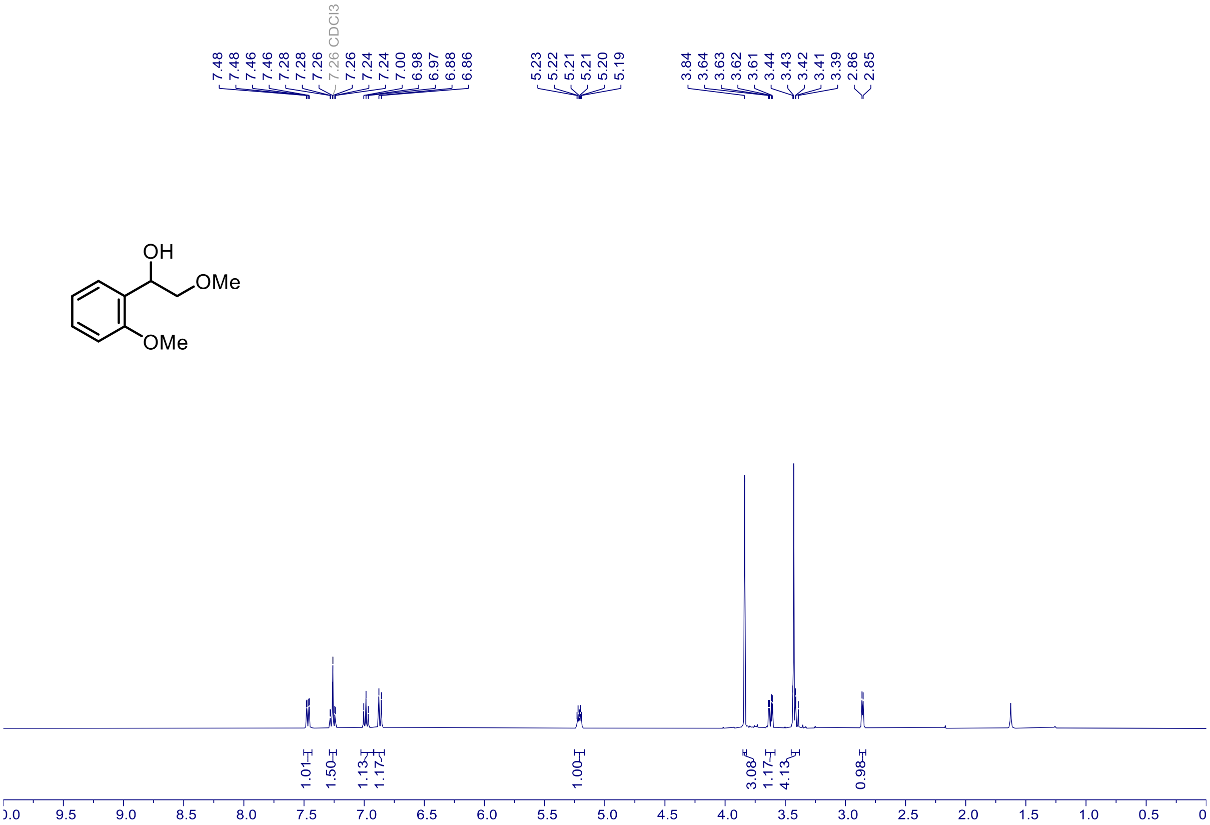

4u – ^{13}C NMR (101 MHz, $CDCl_3$)

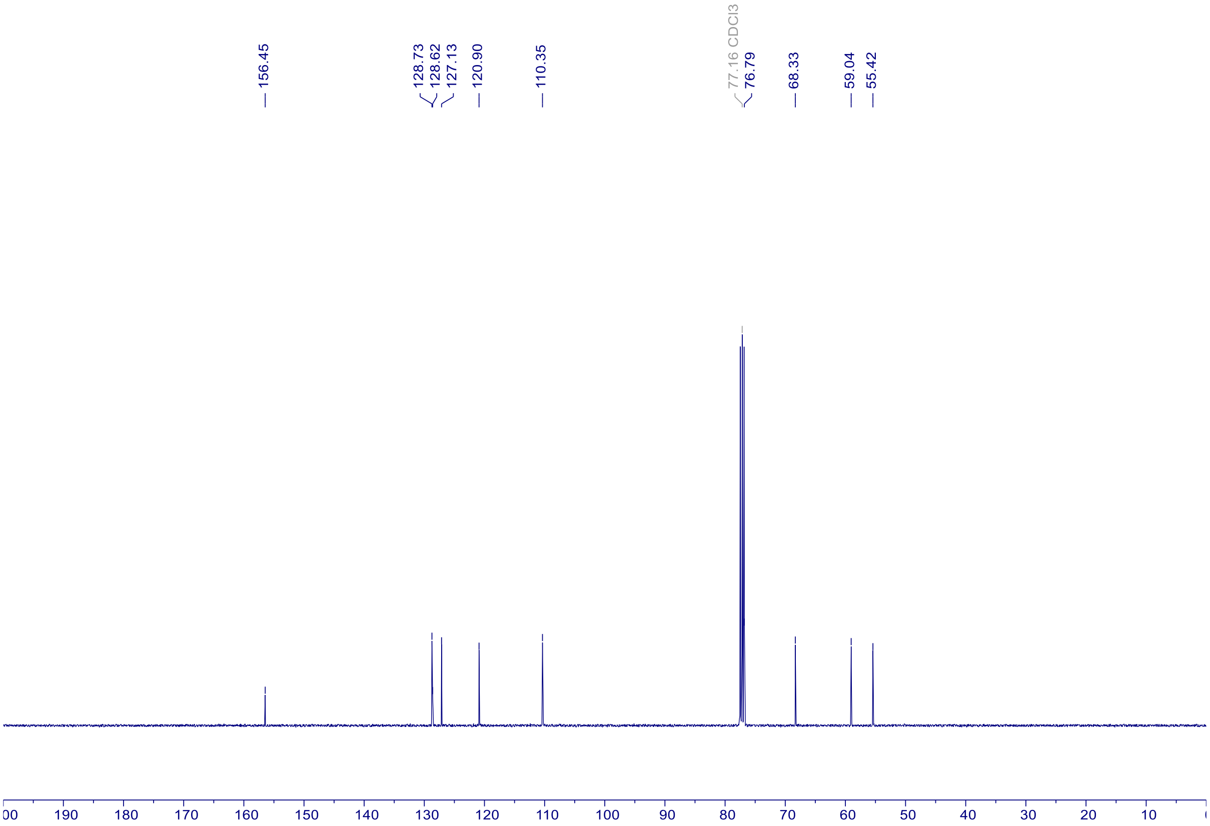

4v – ^{1}H NMR (400 MHz, $CDCl_3$)

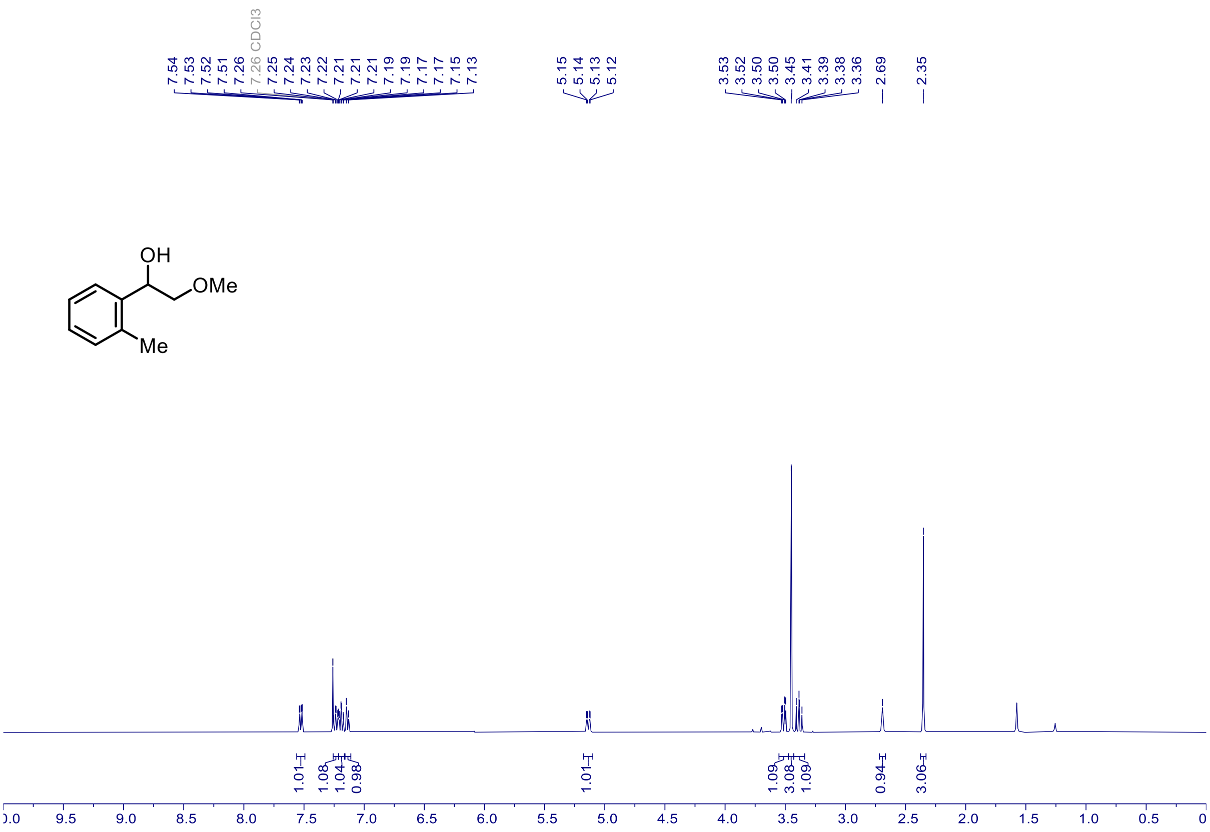

4v – ^{13}C NMR (101 MHz, $CDCl_3$)

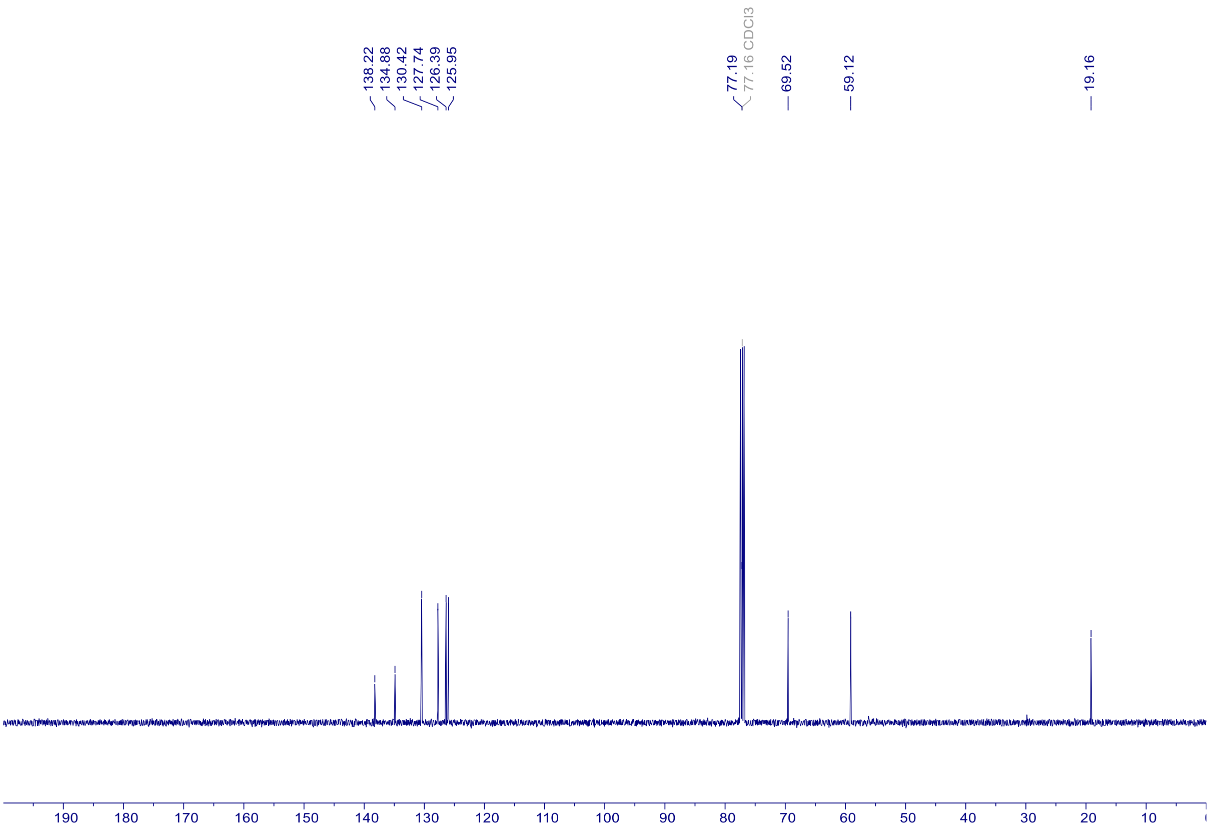

4w – ^{1}H NMR (400 MHz, $CDCl_3$)

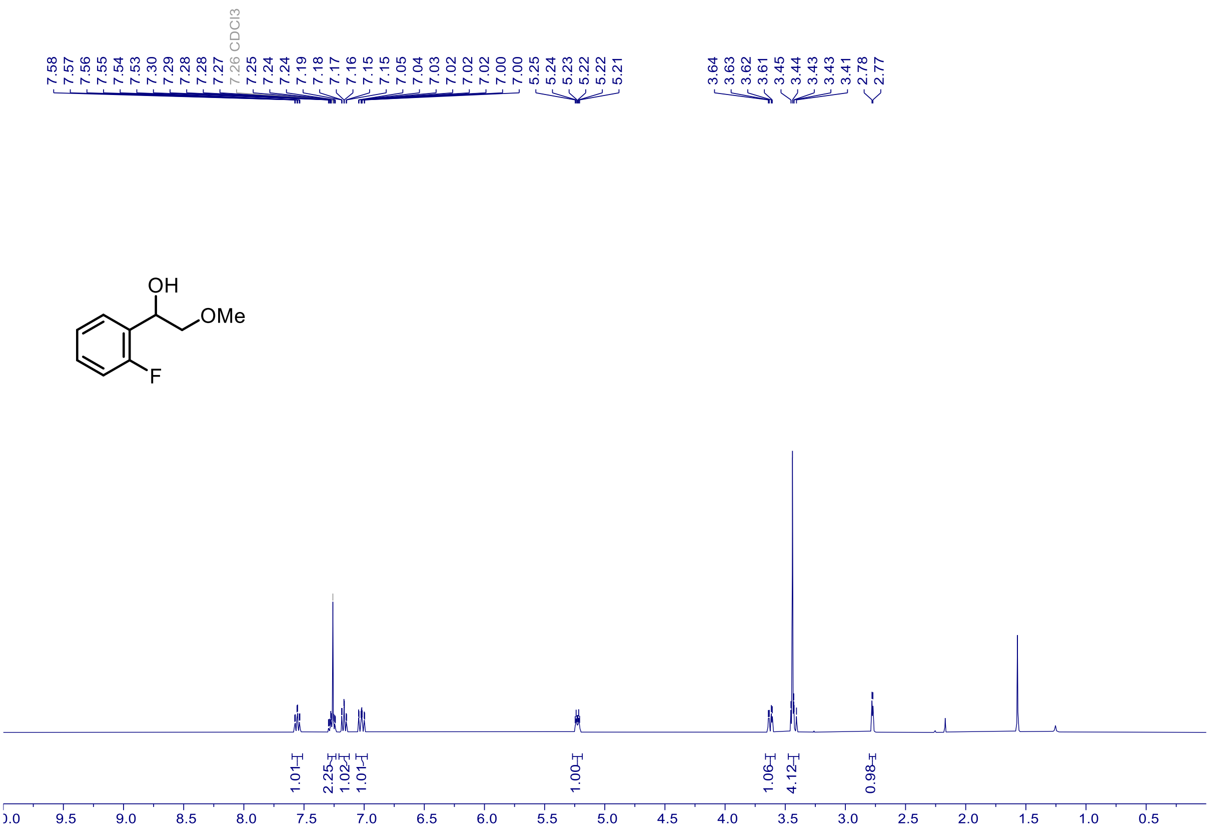

4w – ^{13}C NMR (101 MHz, $CDCl_3$)

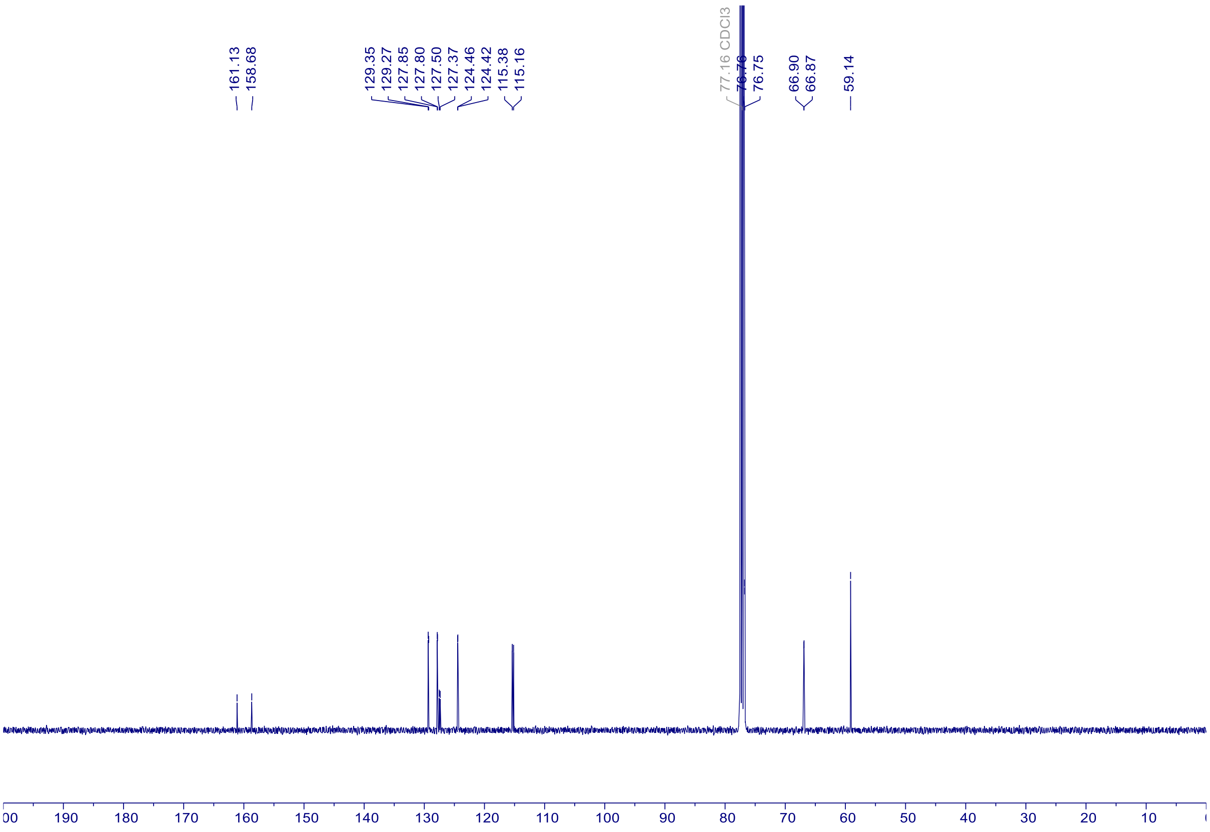

4w – ^{19}F NMR (376 MHz, $CDCl_3$)

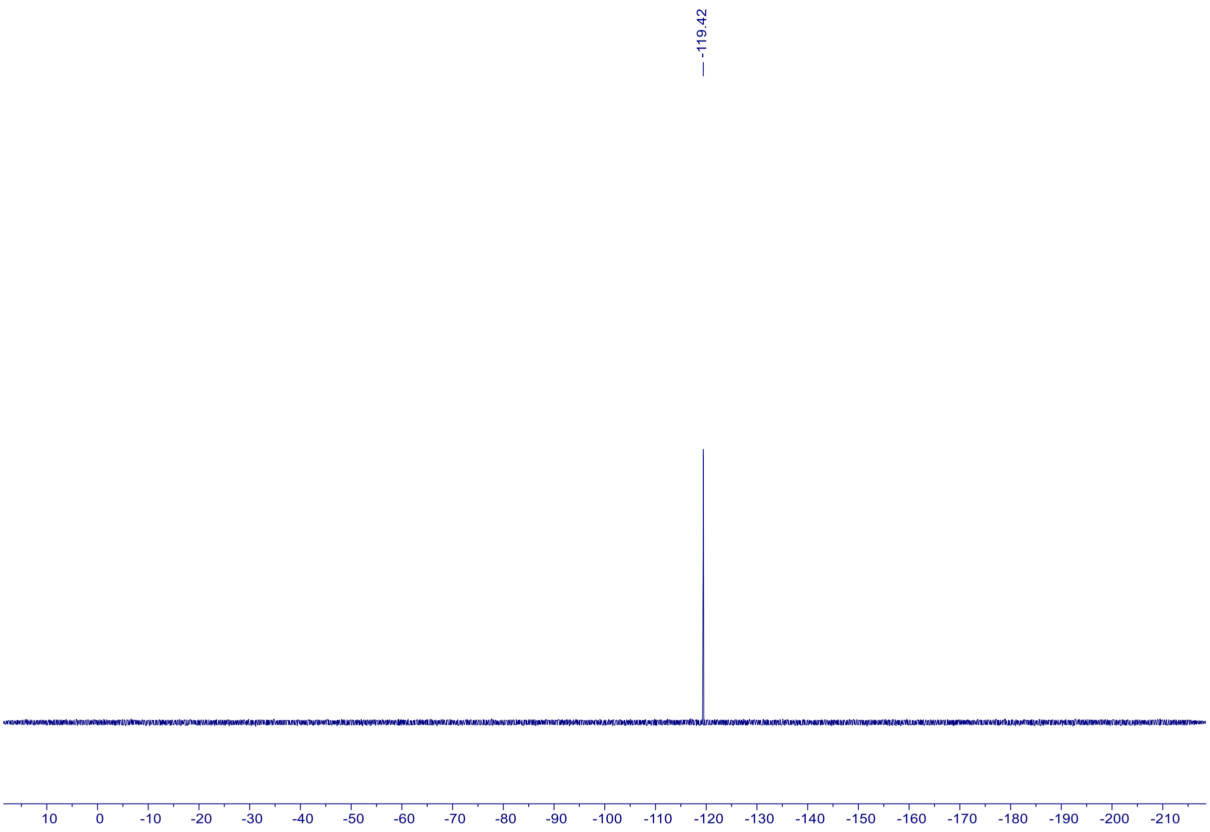

4x – ^{1}H NMR (500 MHz, $CDCl_3$)

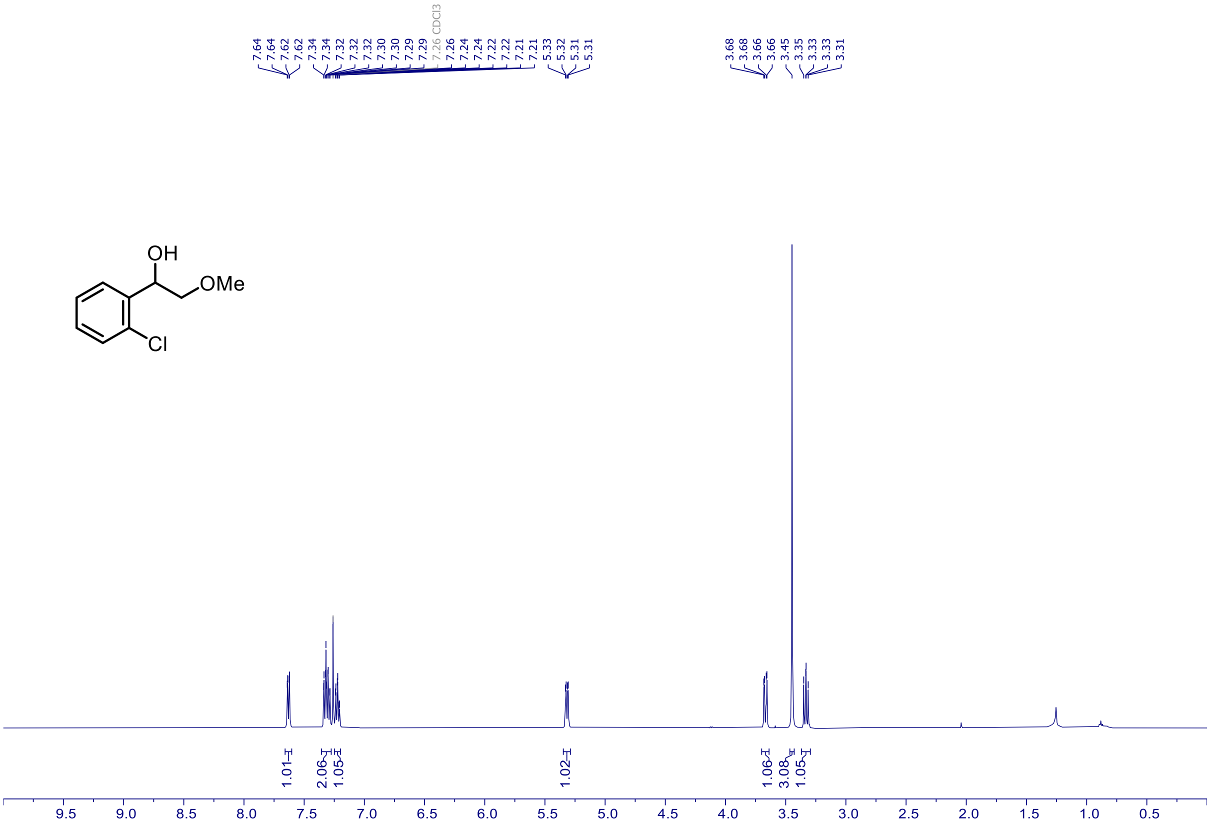

4x – ^{13}C NMR (126 MHz, $CDCl_3$)

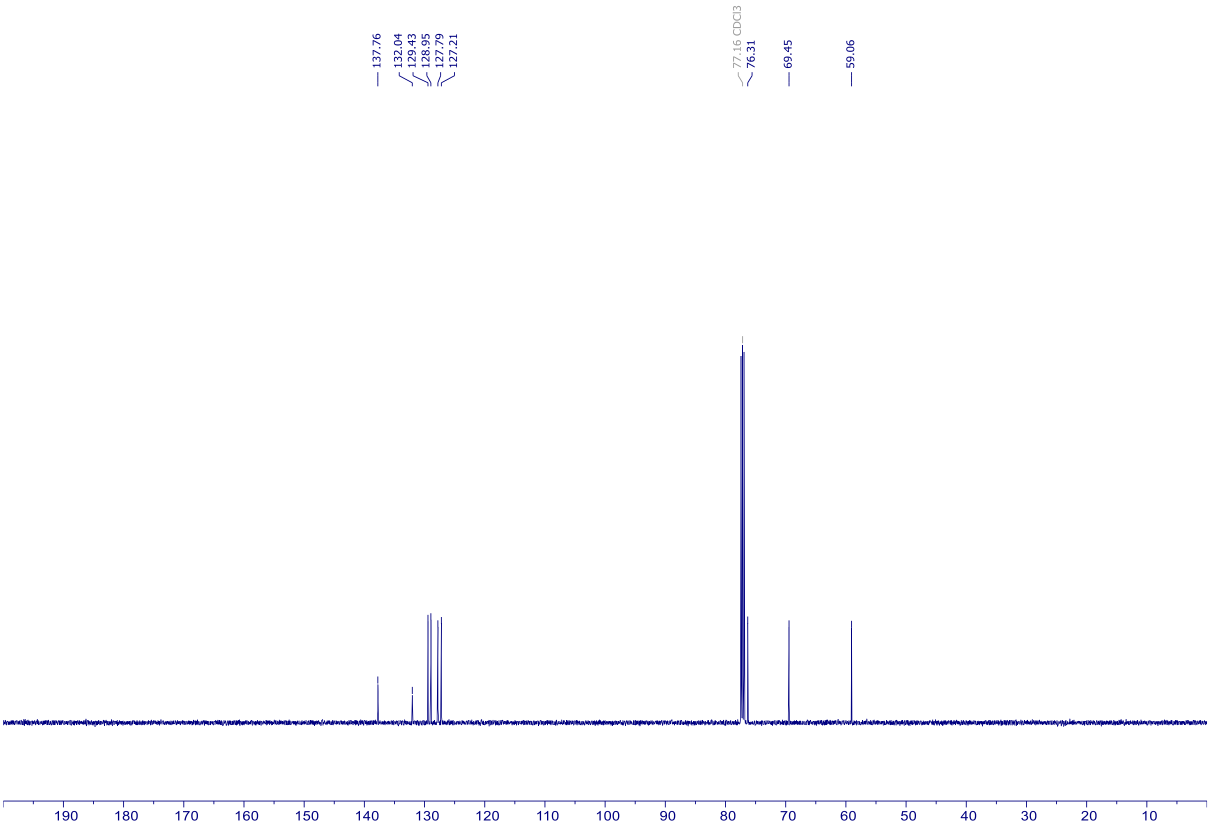

4y – ^{1}H NMR (400 MHz, CDCl$_3$)

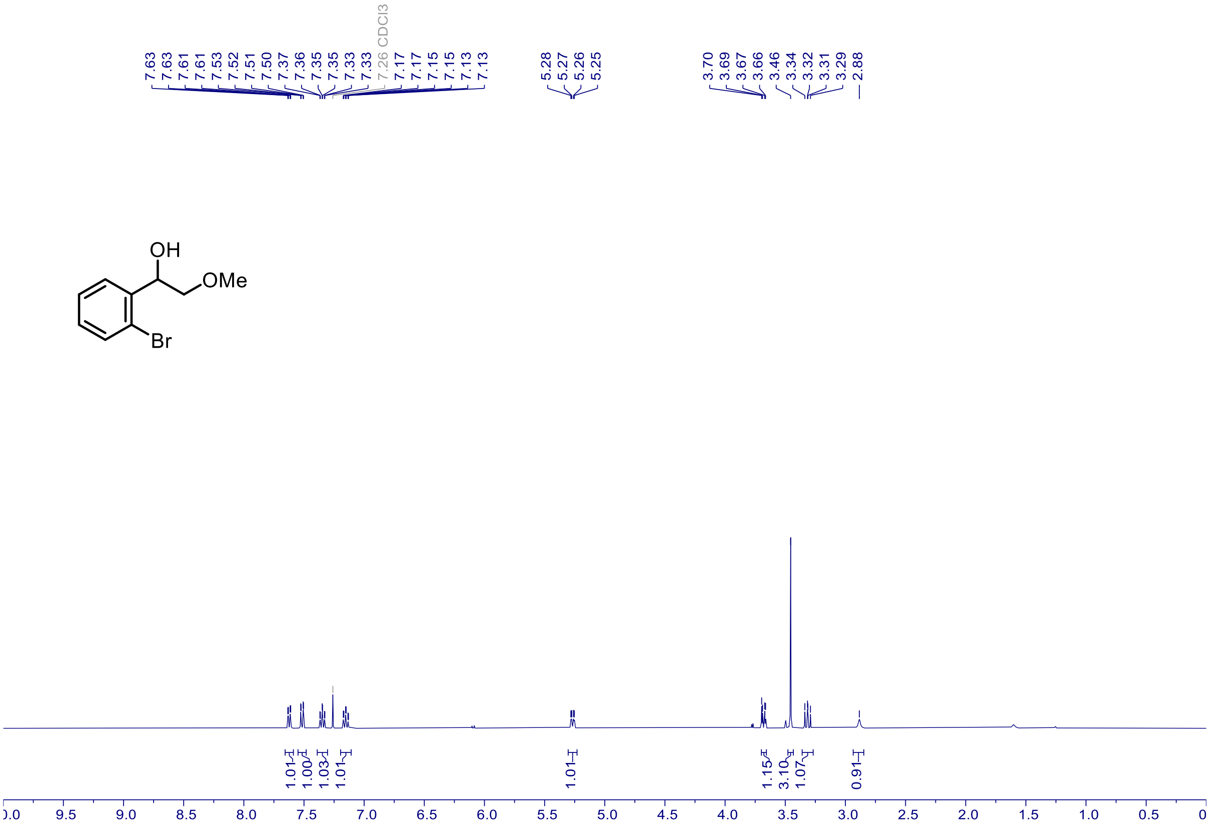

4y – ^{13}C NMR (101 MHz, CDCl$_3$)

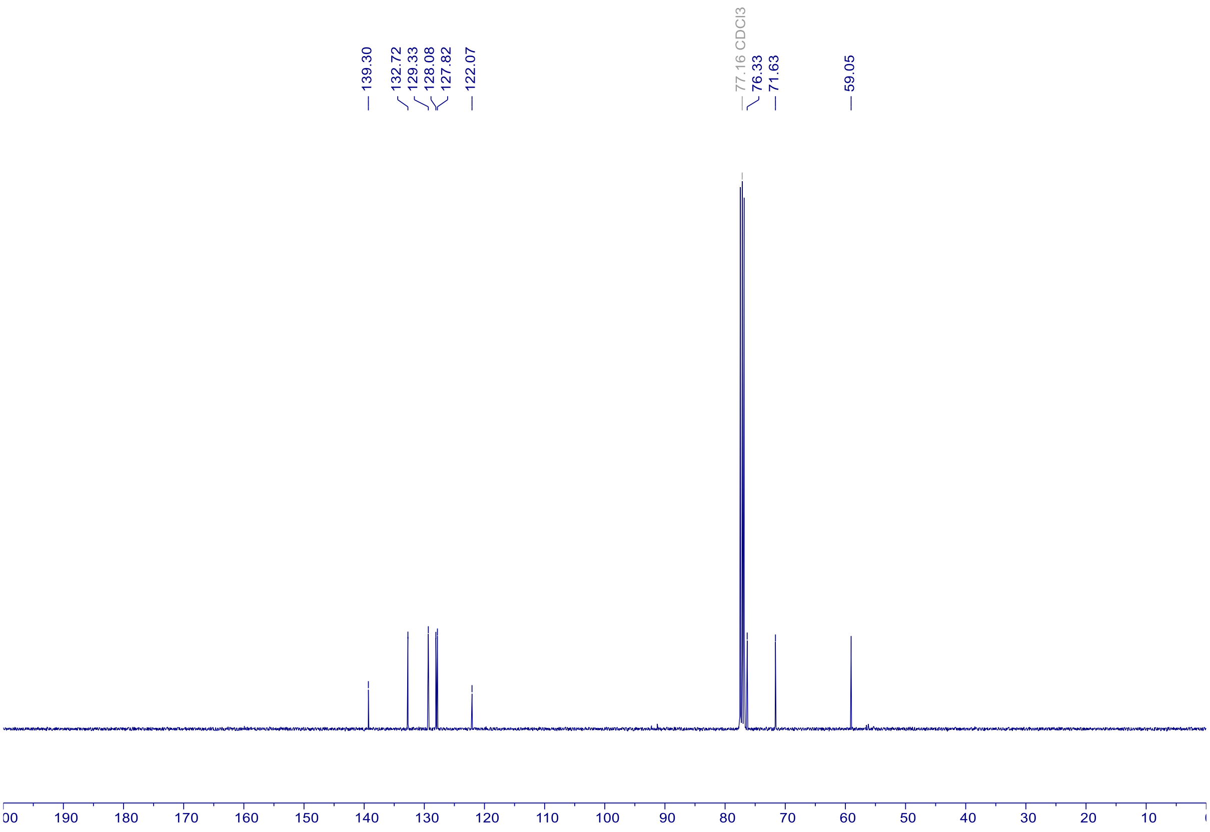

4z – ^{1}H NMR (500 MHz, CDCl$_3$)

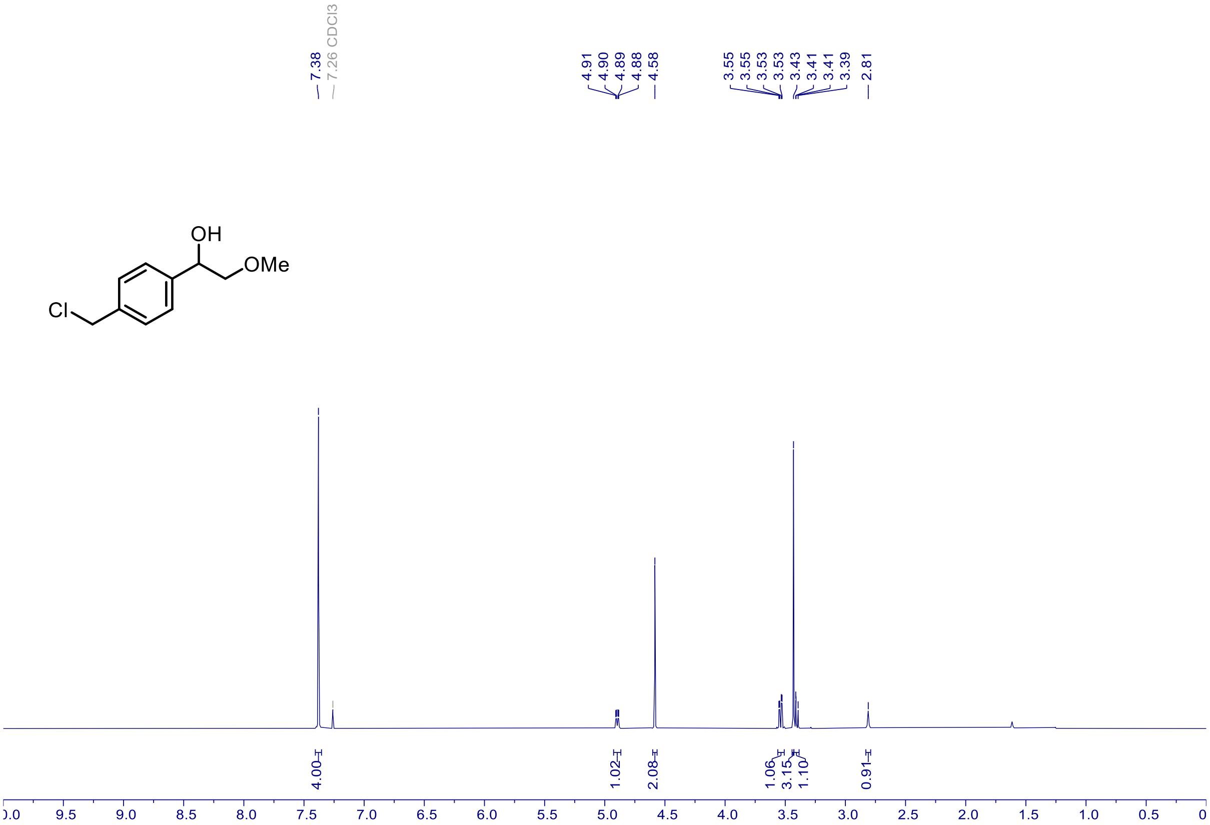

4z – ^{13}C NMR (126 MHz, CDCl$_3$)

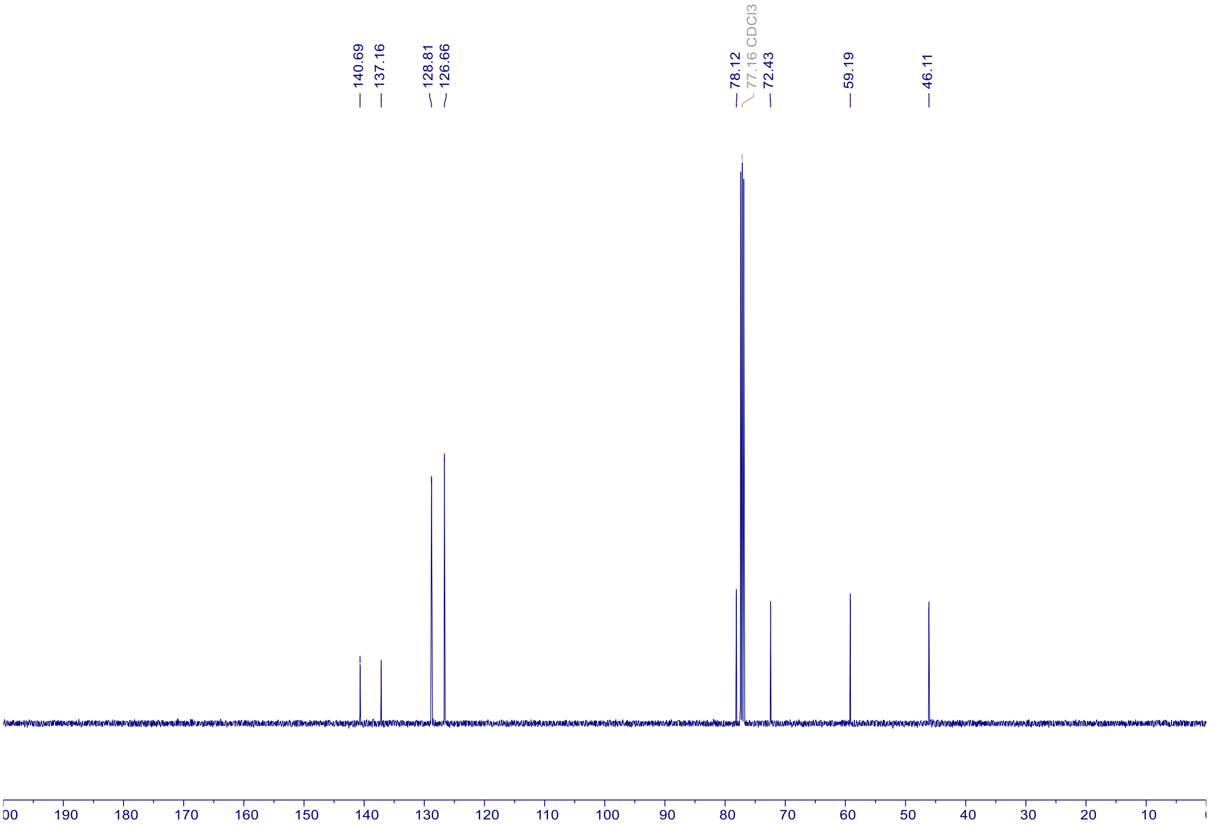

4aa – ^{1}H NMR (400 MHz, $CDCl_3$)

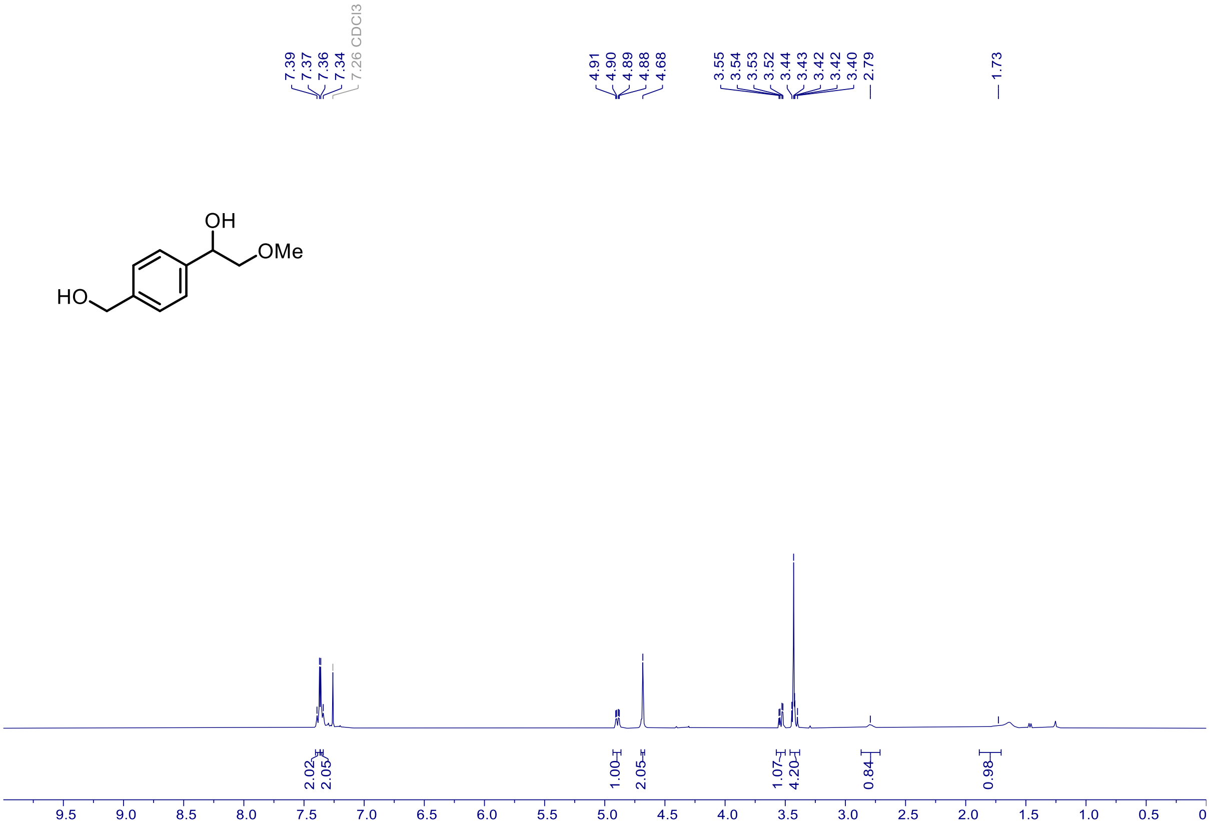

4aa – ^{13}C NMR (101 MHz, $CDCl_3$)

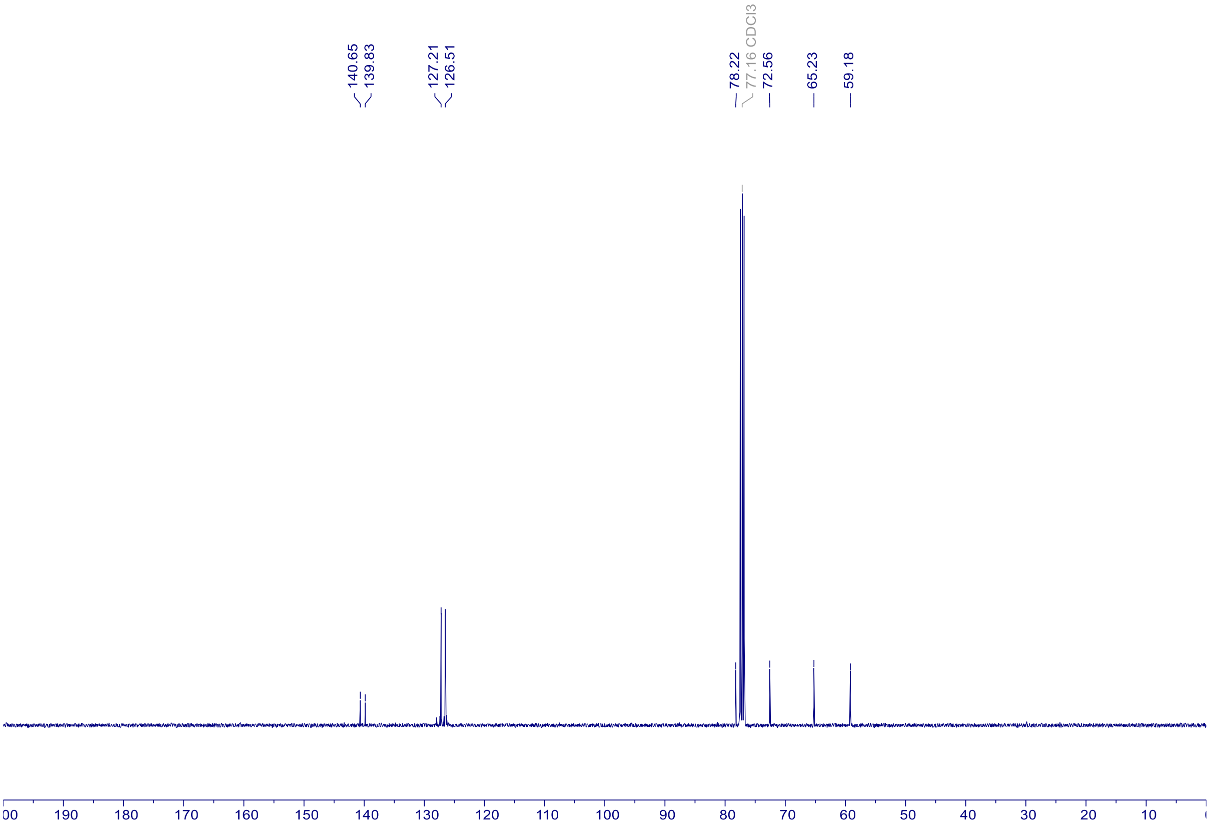

4ab – ^{1}H NMR (400 MHz, $CDCl_3$)

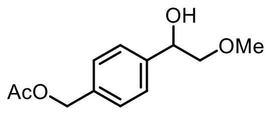

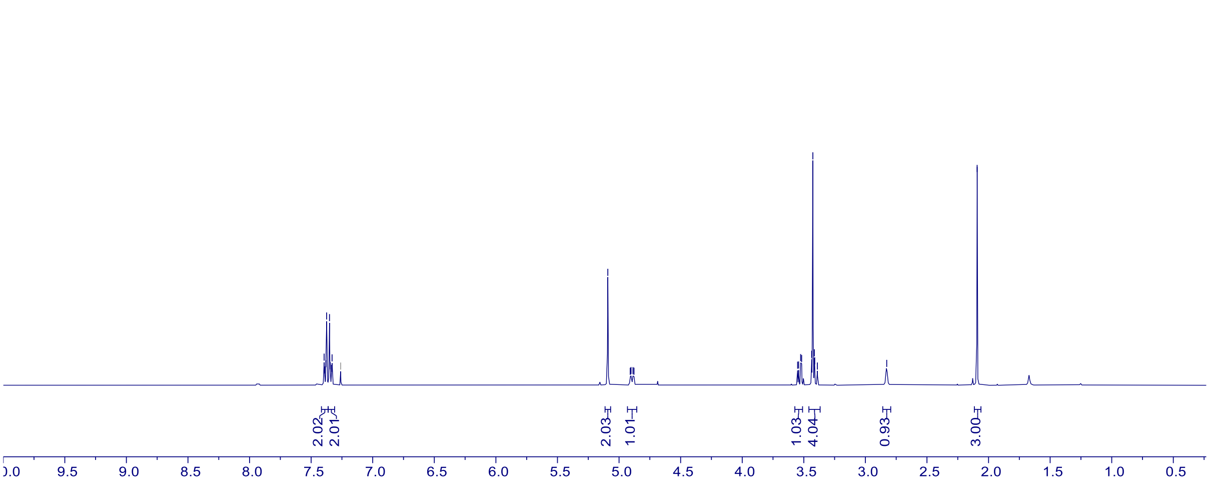

4ab – ^{13}C NMR (101 MHz, $CDCl_3$)

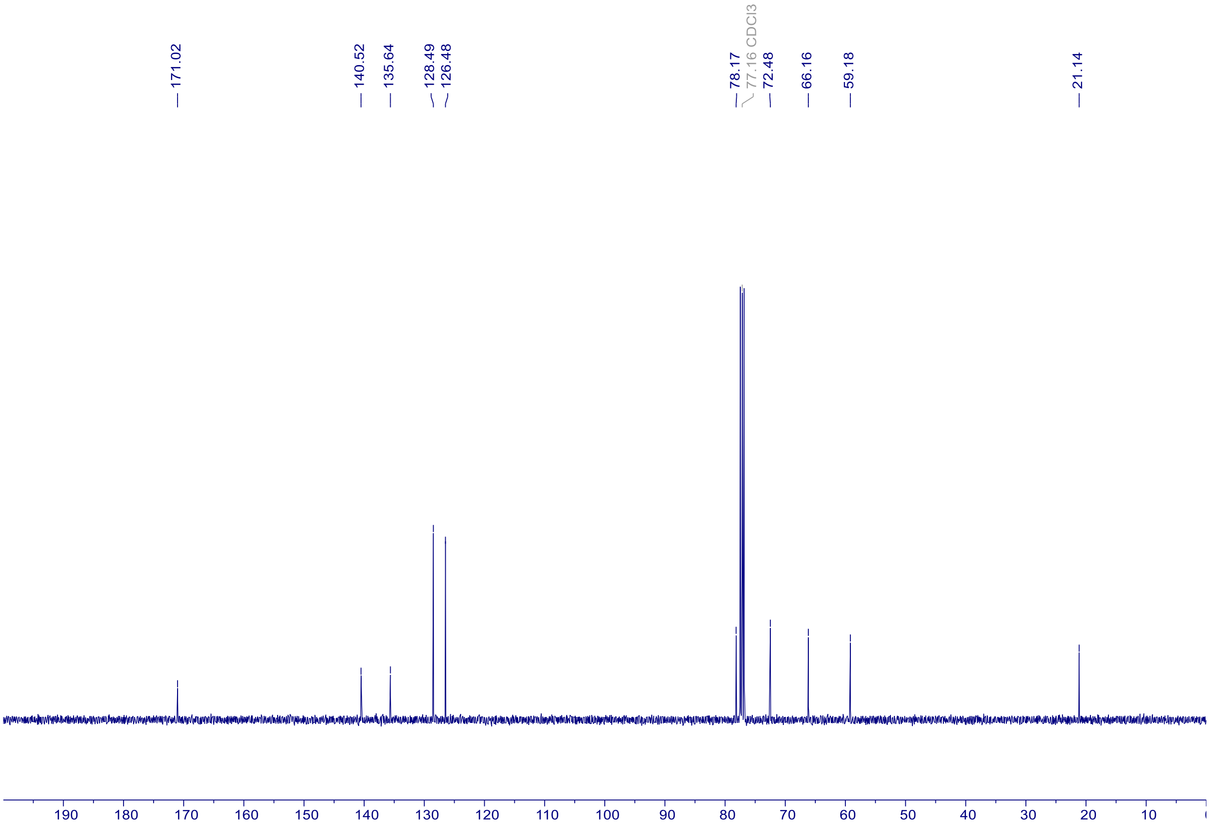

4ac – ^{1}H NMR (500 MHz, $CDCl_3$)

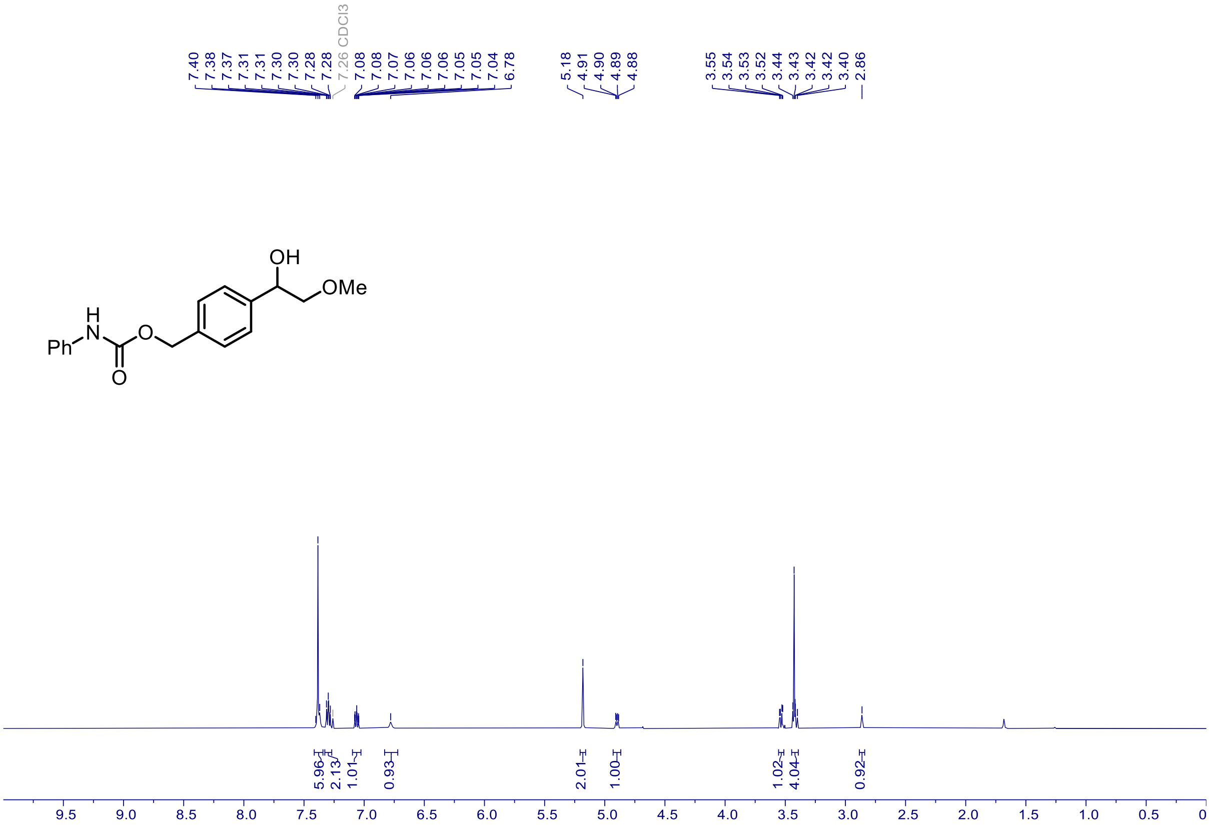

4ac – ^{13}C NMR (126 MHz, $CDCl_3$)

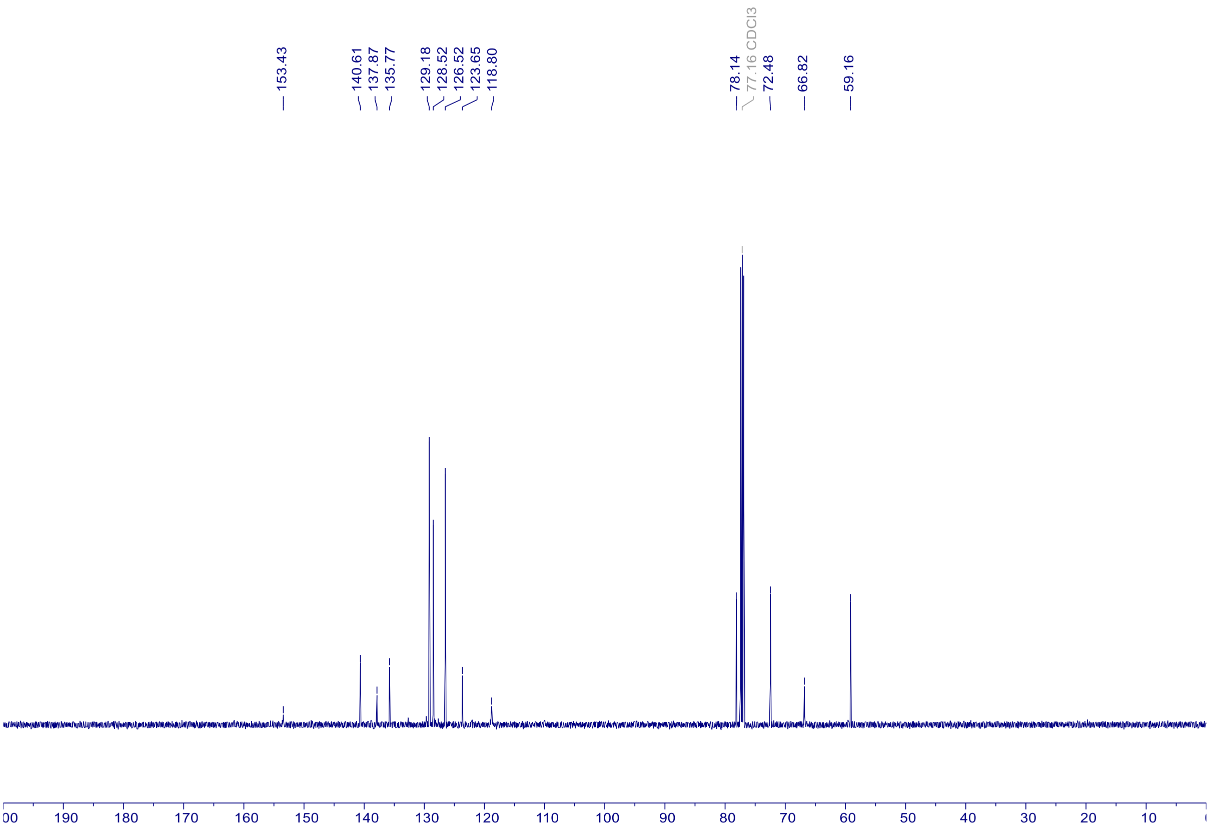

4ad – ^{1}H NMR (400 MHz, $CDCl_3$)

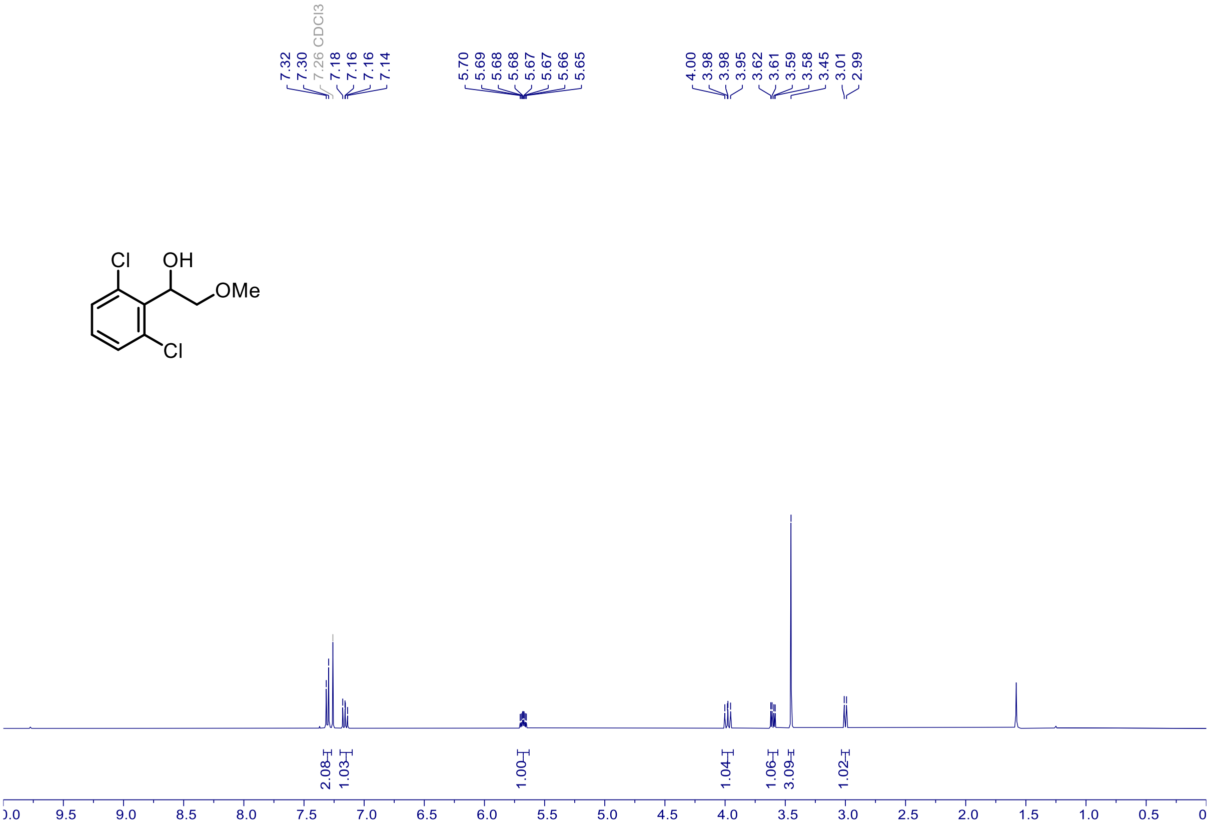

4ad – ^{13}C NMR (101 MHz, $CDCl_3$)

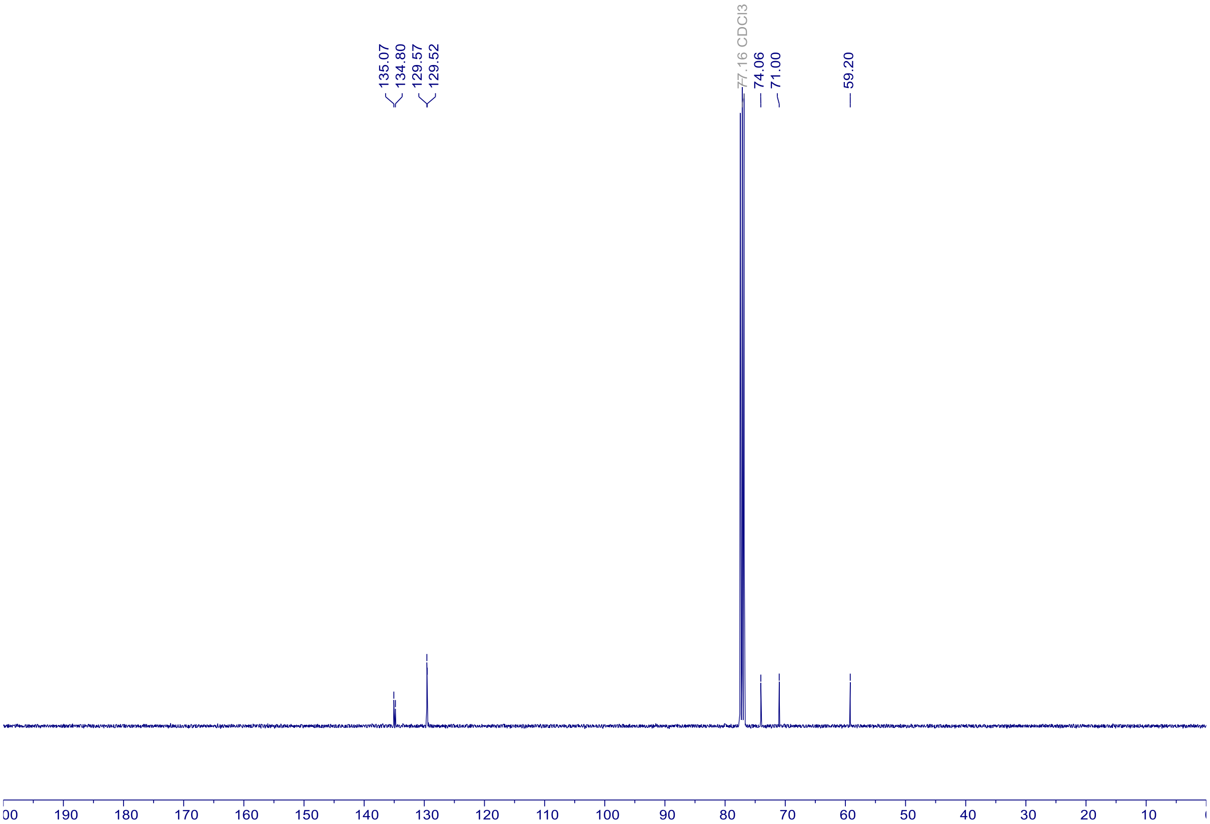

4ae – ^{1}H NMR (400 MHz, $CDCl_3$)

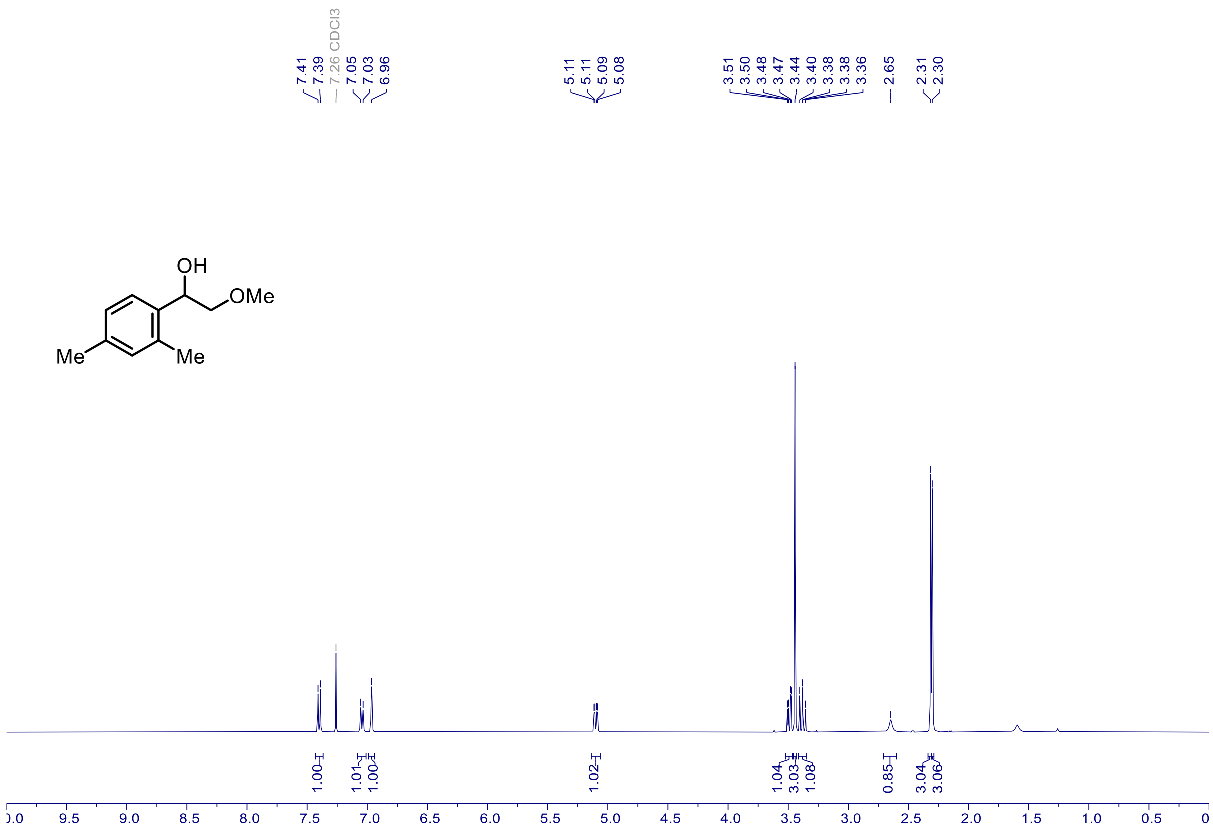

4ae – ^{13}C NMR (101 MHz, $CDCl_3$)

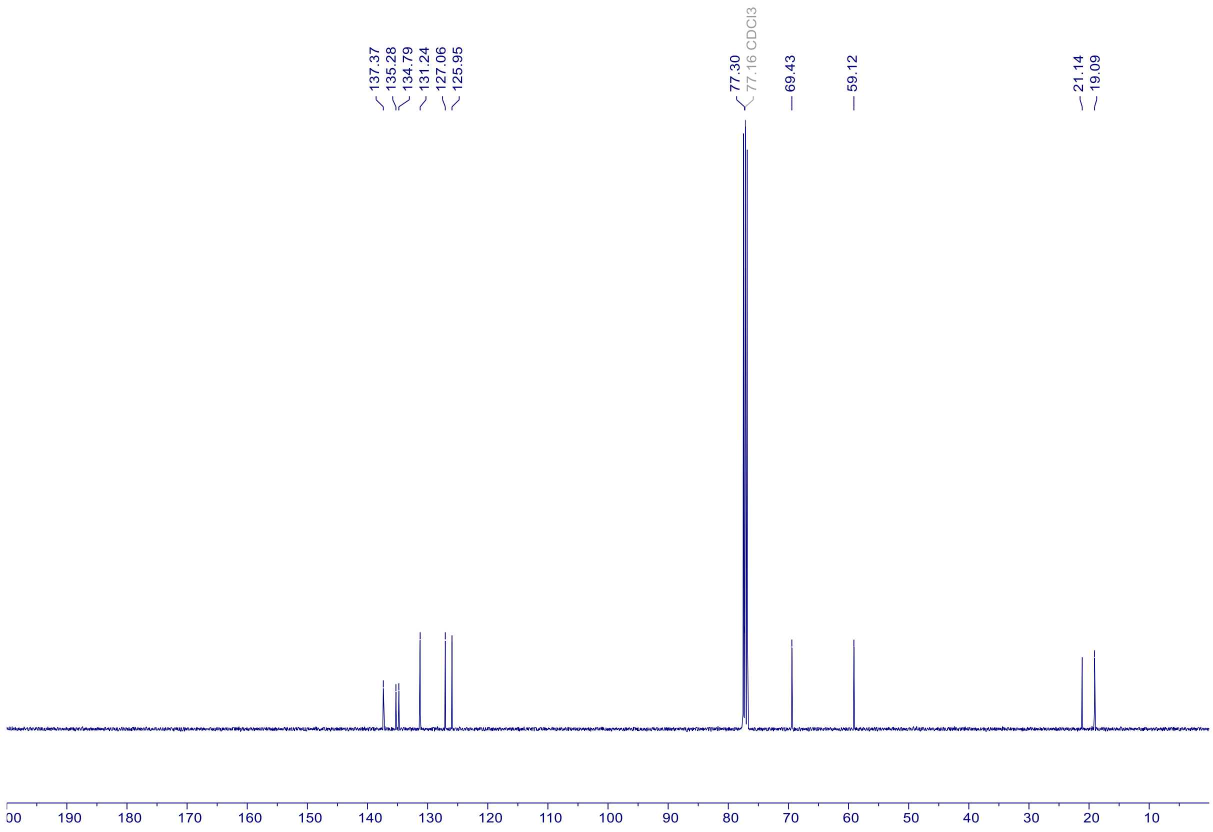

4af – ^{1}H NMR (400 MHz, $CDCl_3$)

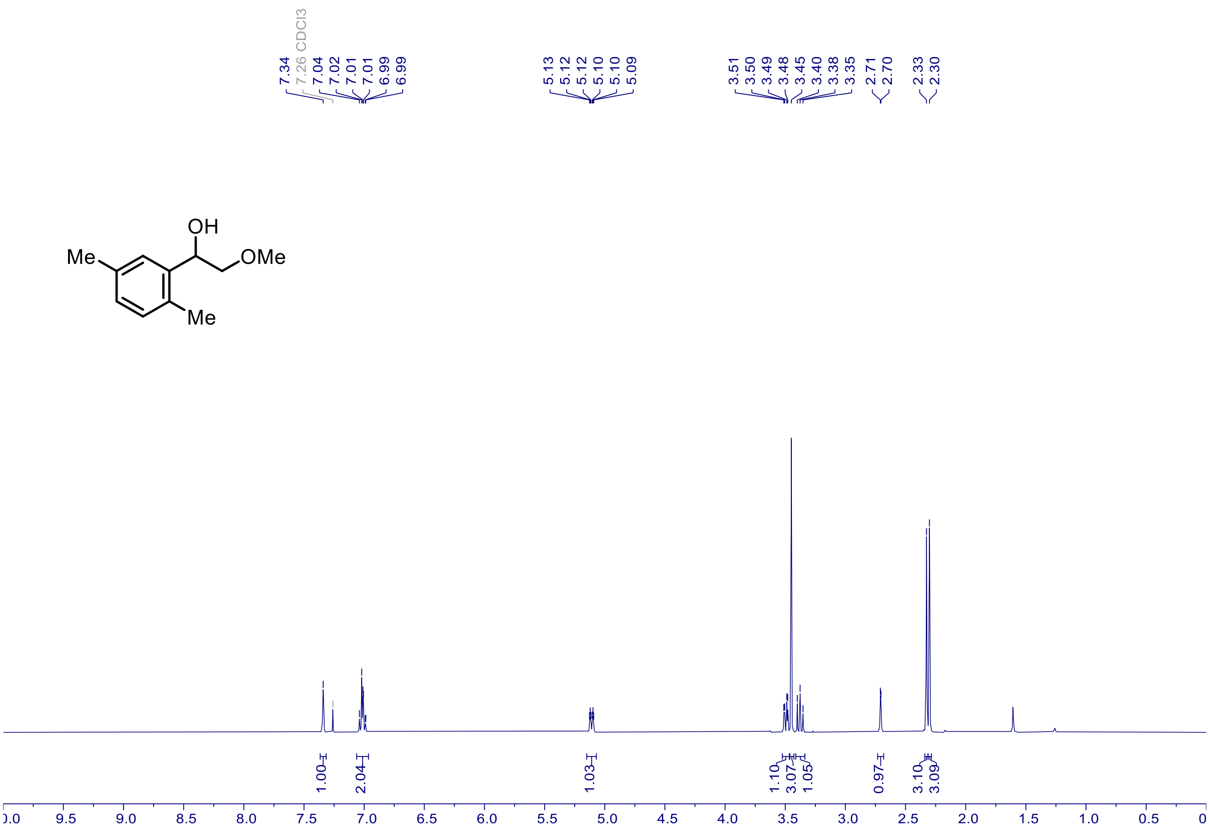

4af – ^{13}C NMR (101 MHz, $CDCl_3$)

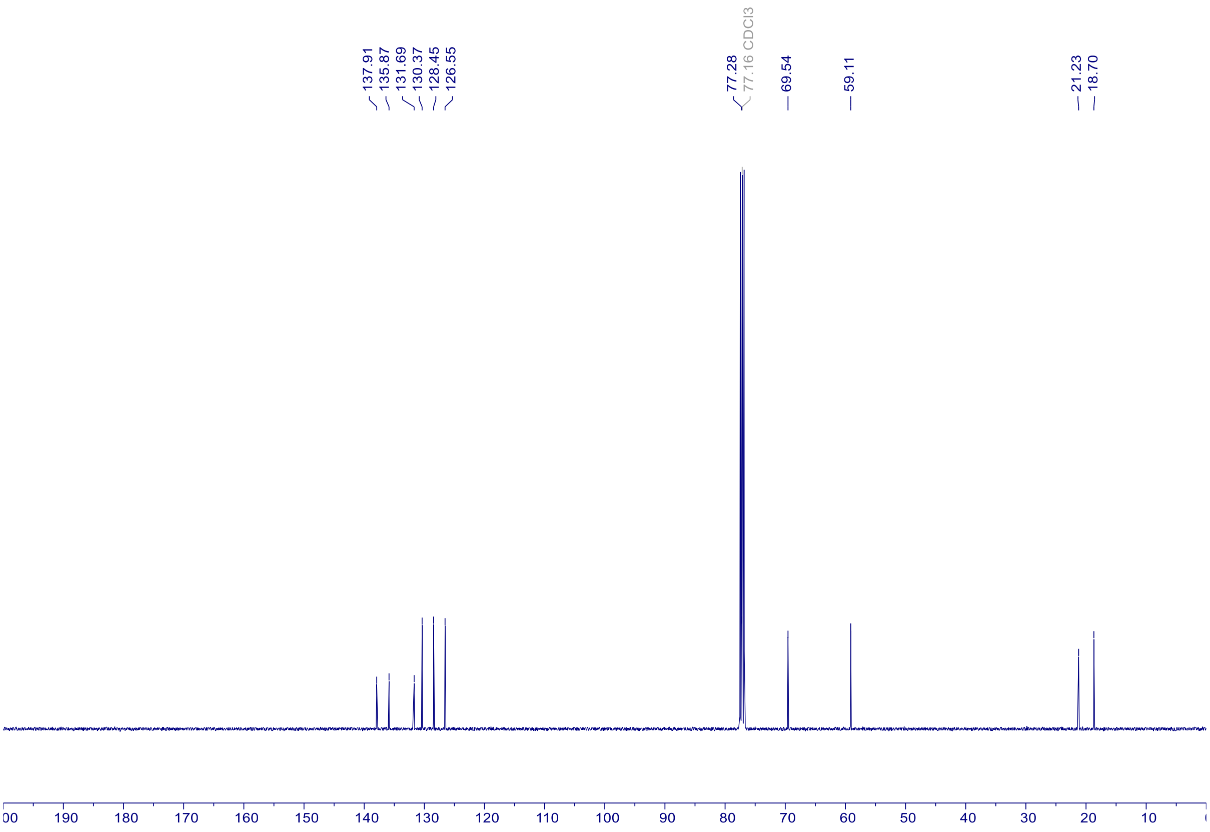

4ag – ^{1}H NMR (400 MHz, $CDCl_3$)

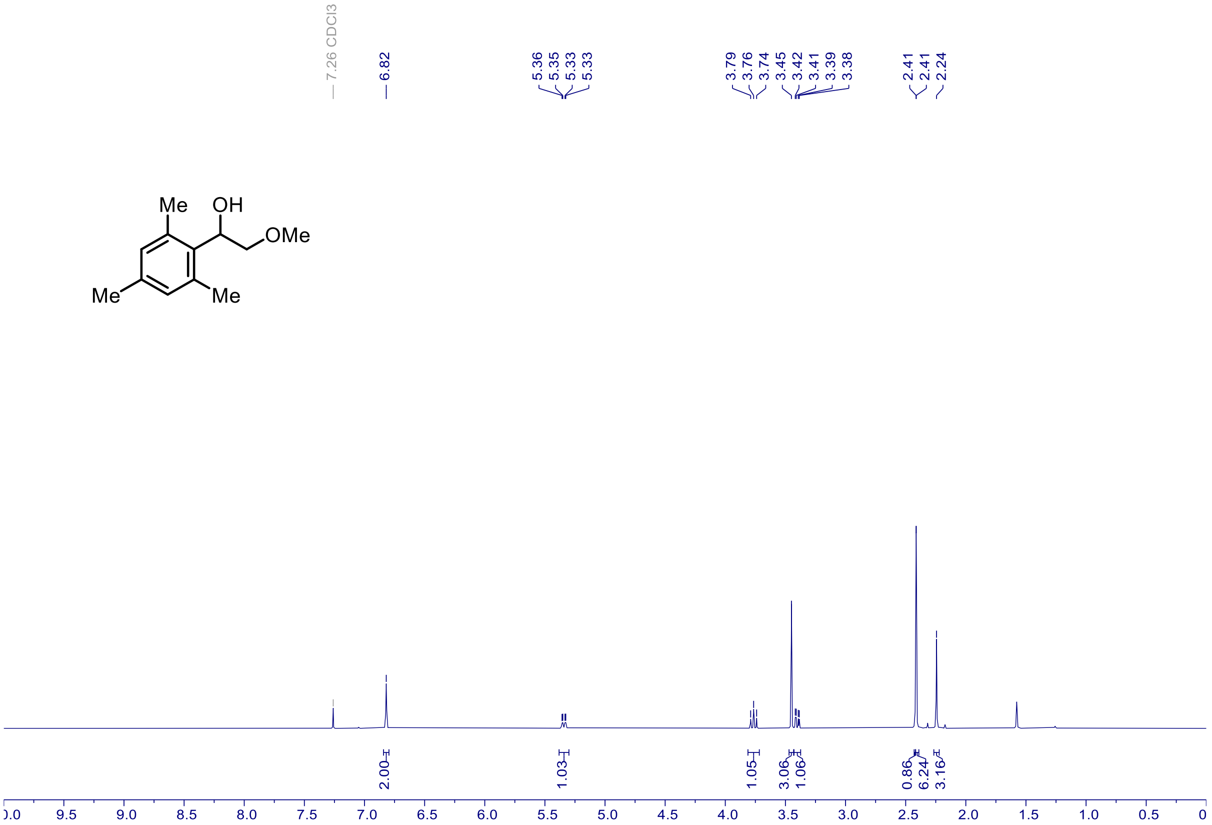

4ag – ^{13}C NMR (101 MHz, $CDCl_3$)

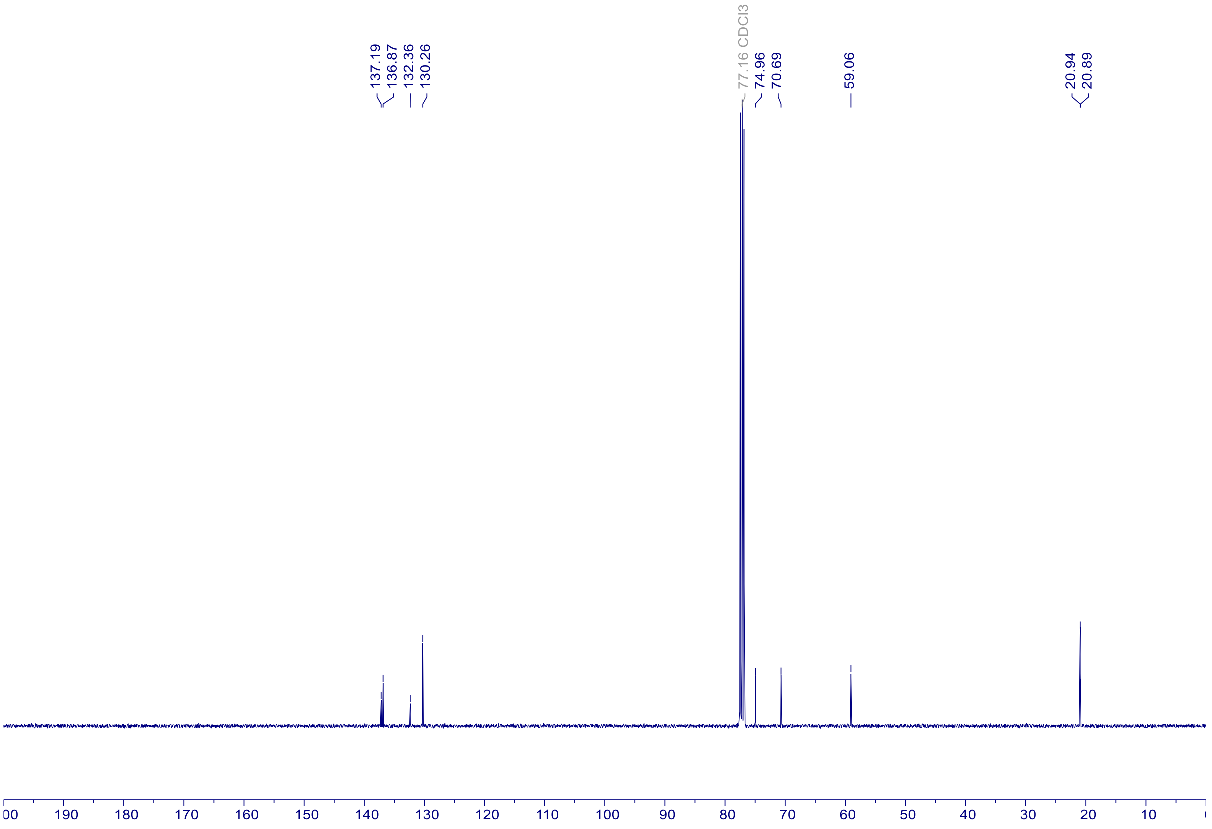

4ah – ^{1}H NMR (400 MHz, $CDCl_3$)

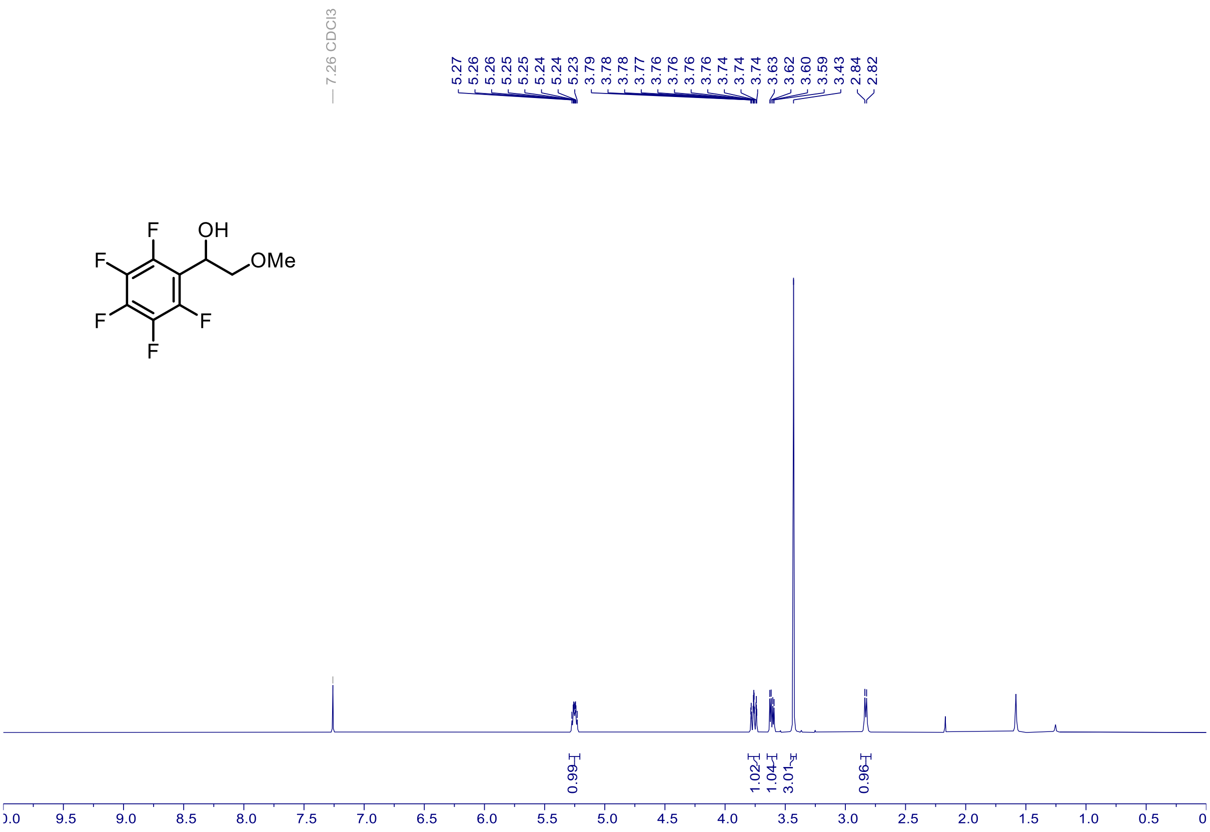

4ah – ^{13}C NMR (101 MHz, $CDCl_3$)

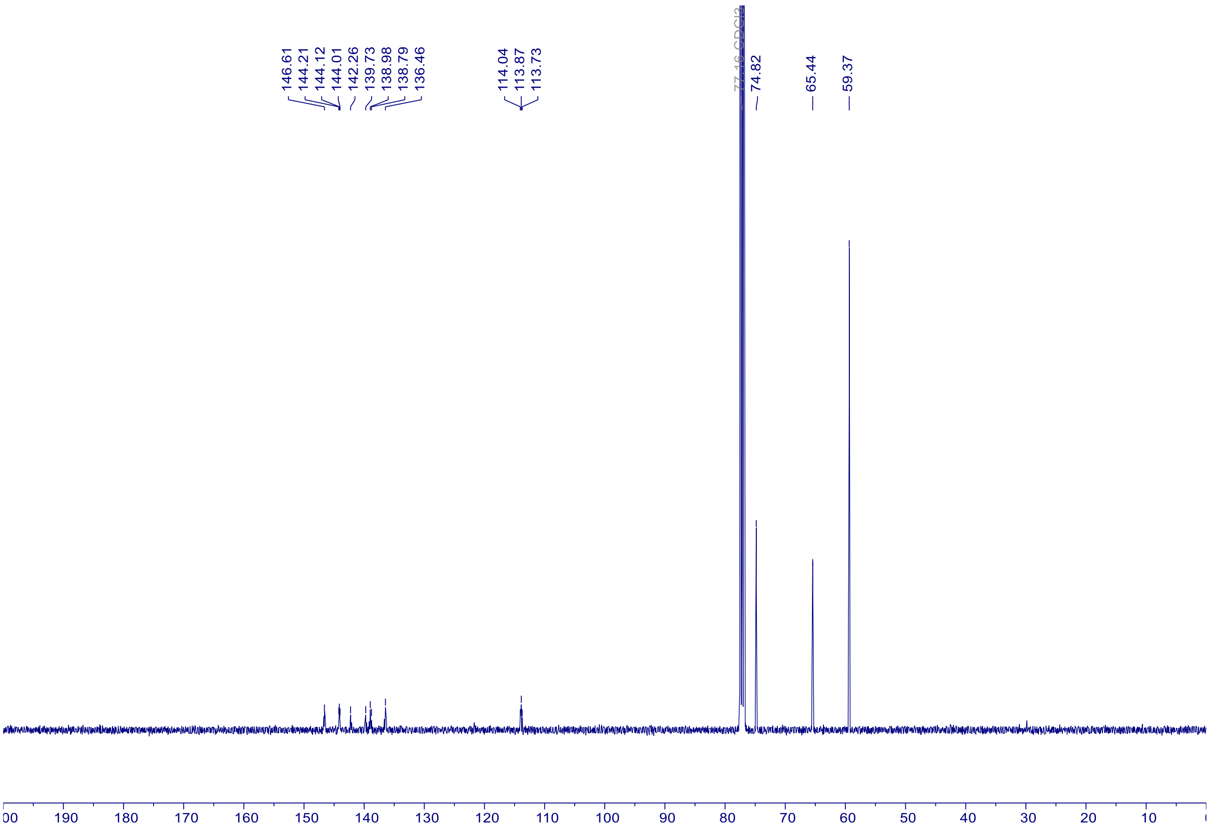

4ah – ^{19}F NMR (376 MHz, $CDCl_3$)

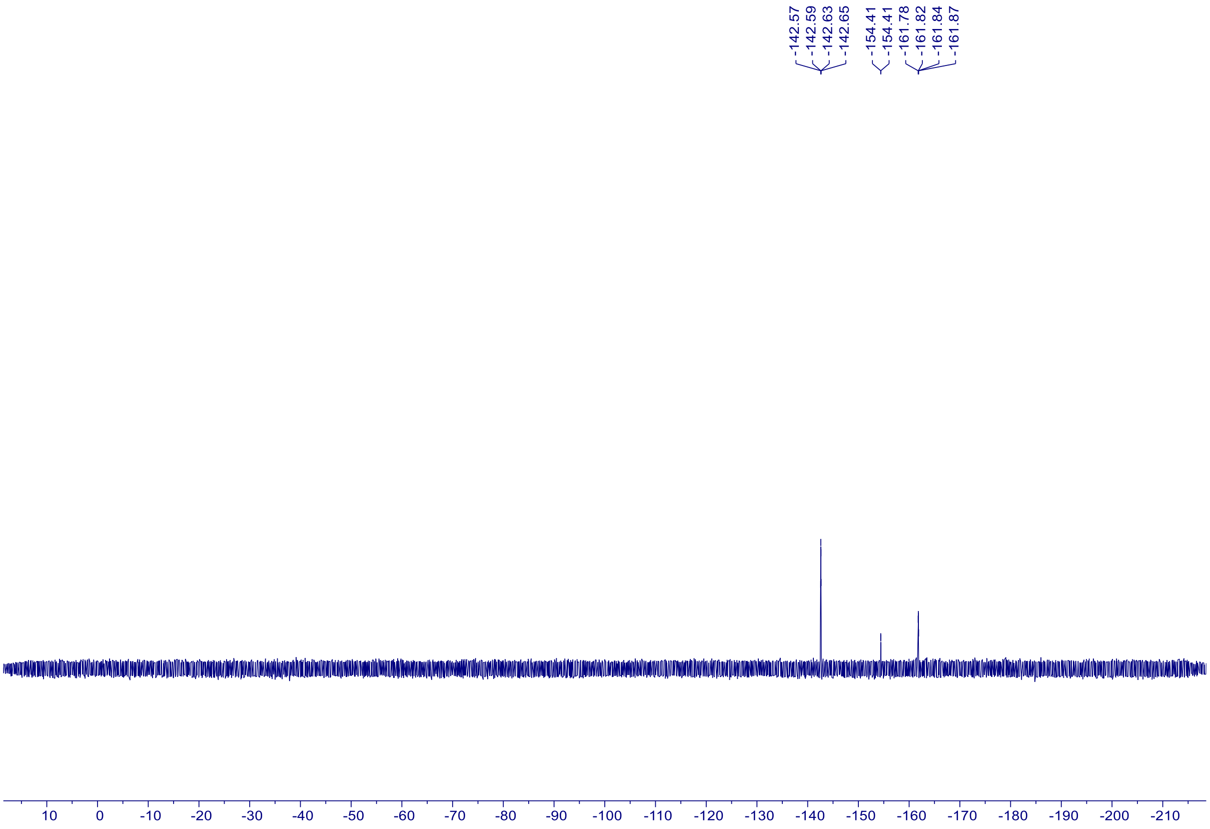

4ai – ^{1}H NMR (400 MHz, $CDCl_3$)

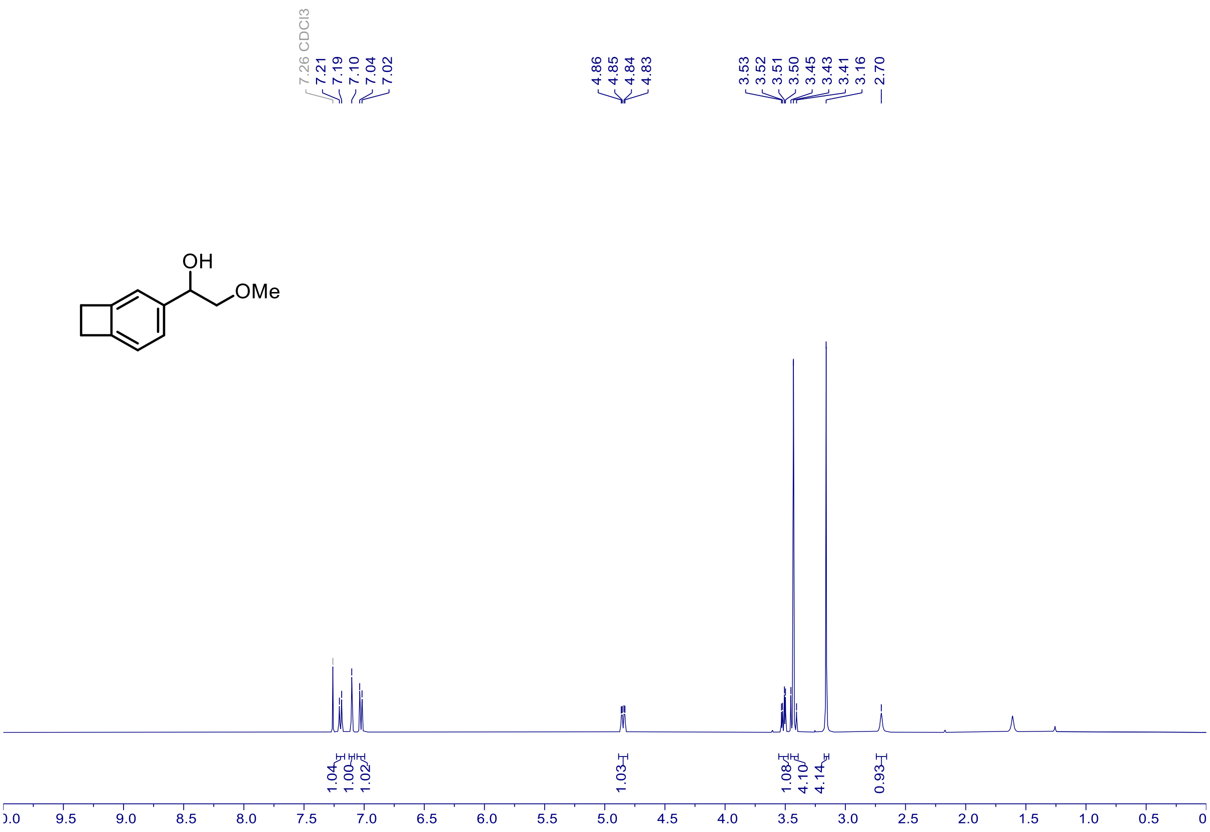

4ai – ^{13}C NMR (101 MHz, $CDCl_3$)

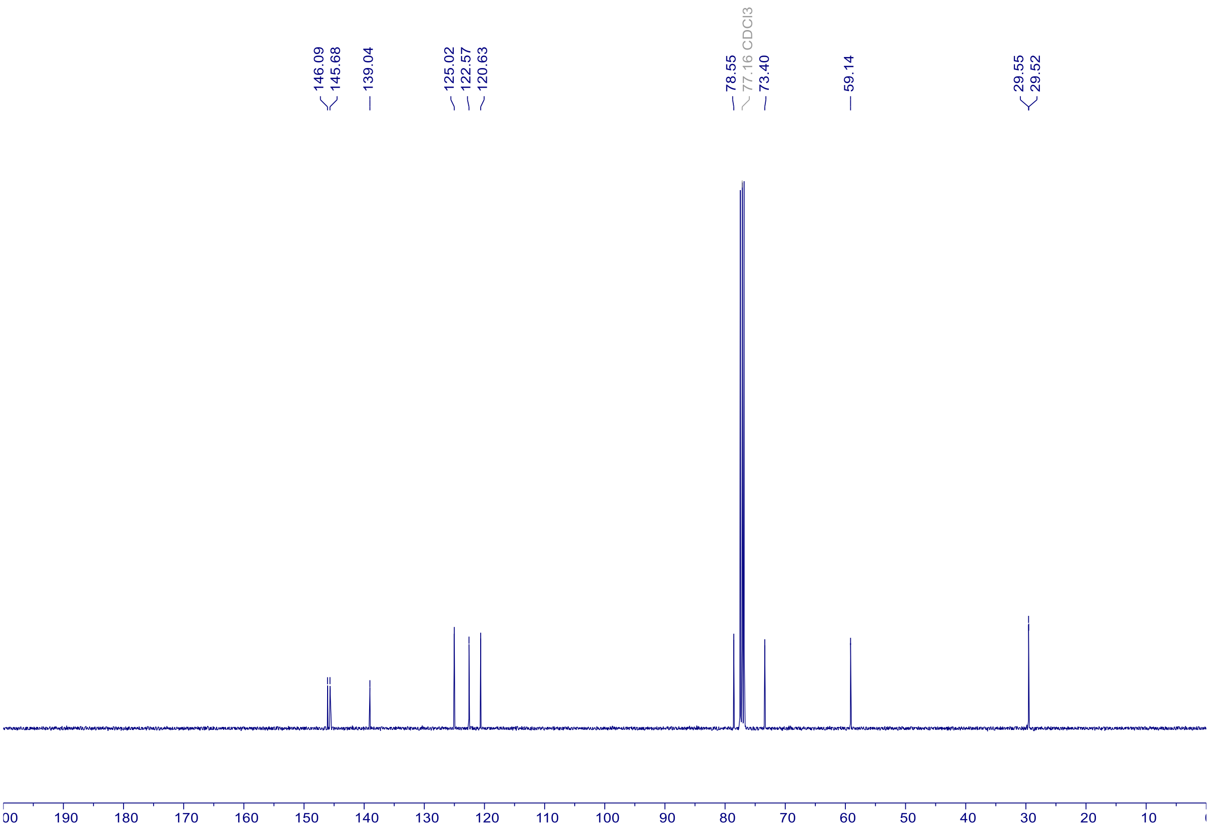

4aj – ^{1}H NMR (400 MHz, $CDCl_3$)

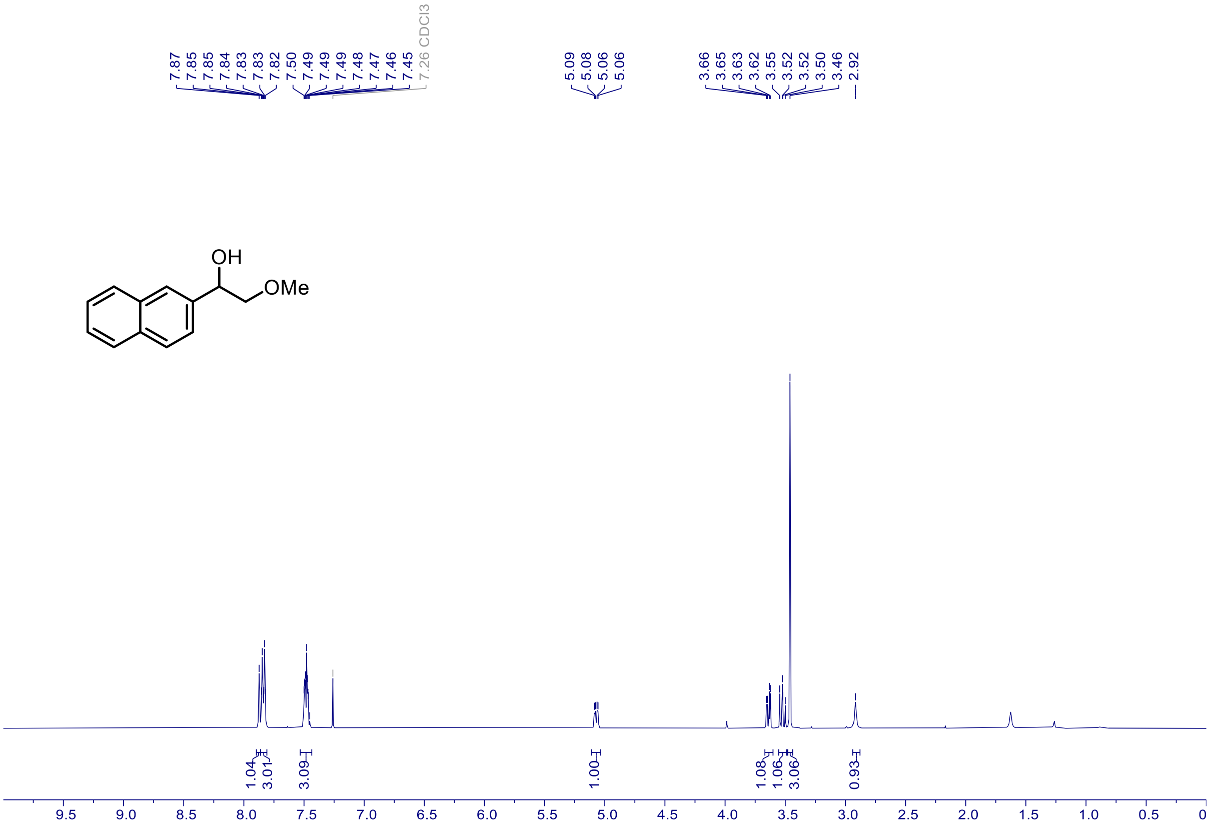

4aj – ^{13}C NMR (101 MHz, $CDCl_3$)

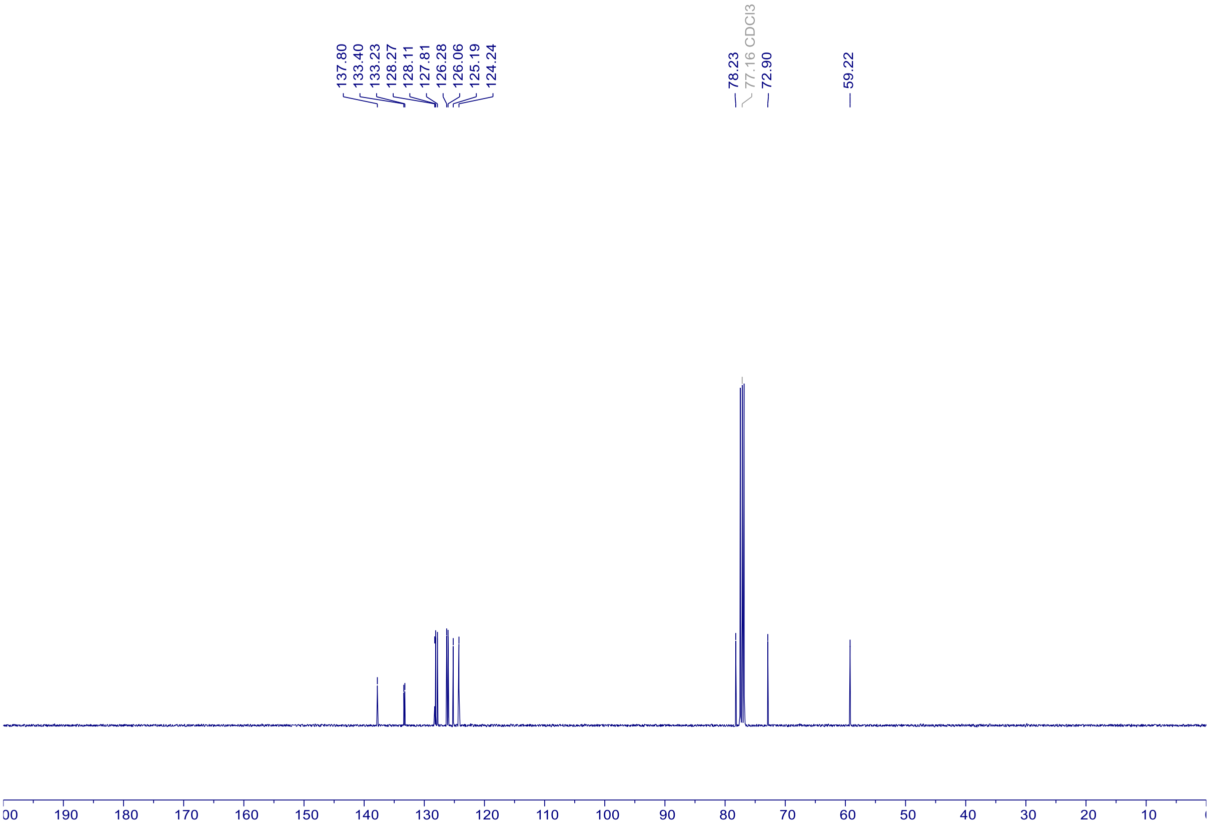

4ak – ^{1}H NMR (400 MHz, $CDCl_3$)

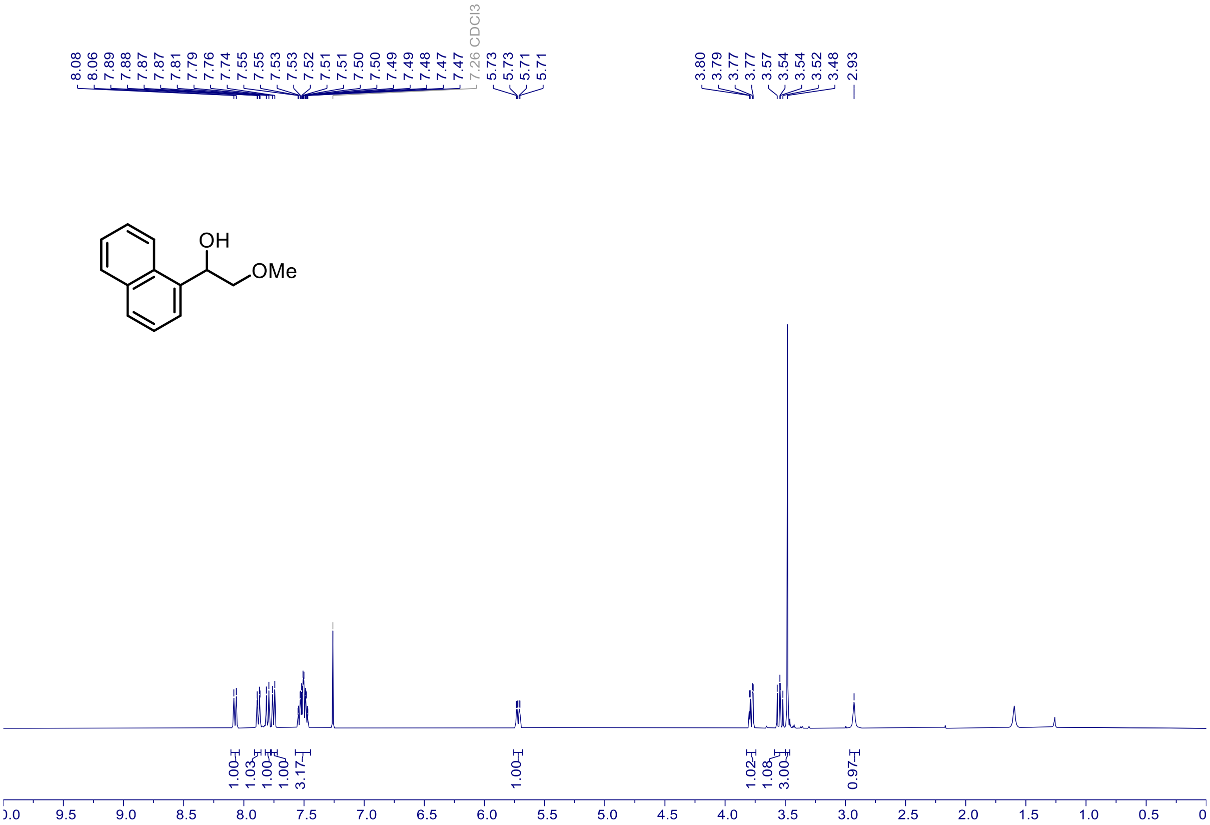

4ak – ^{13}C NMR (101 MHz, $CDCl_3$)

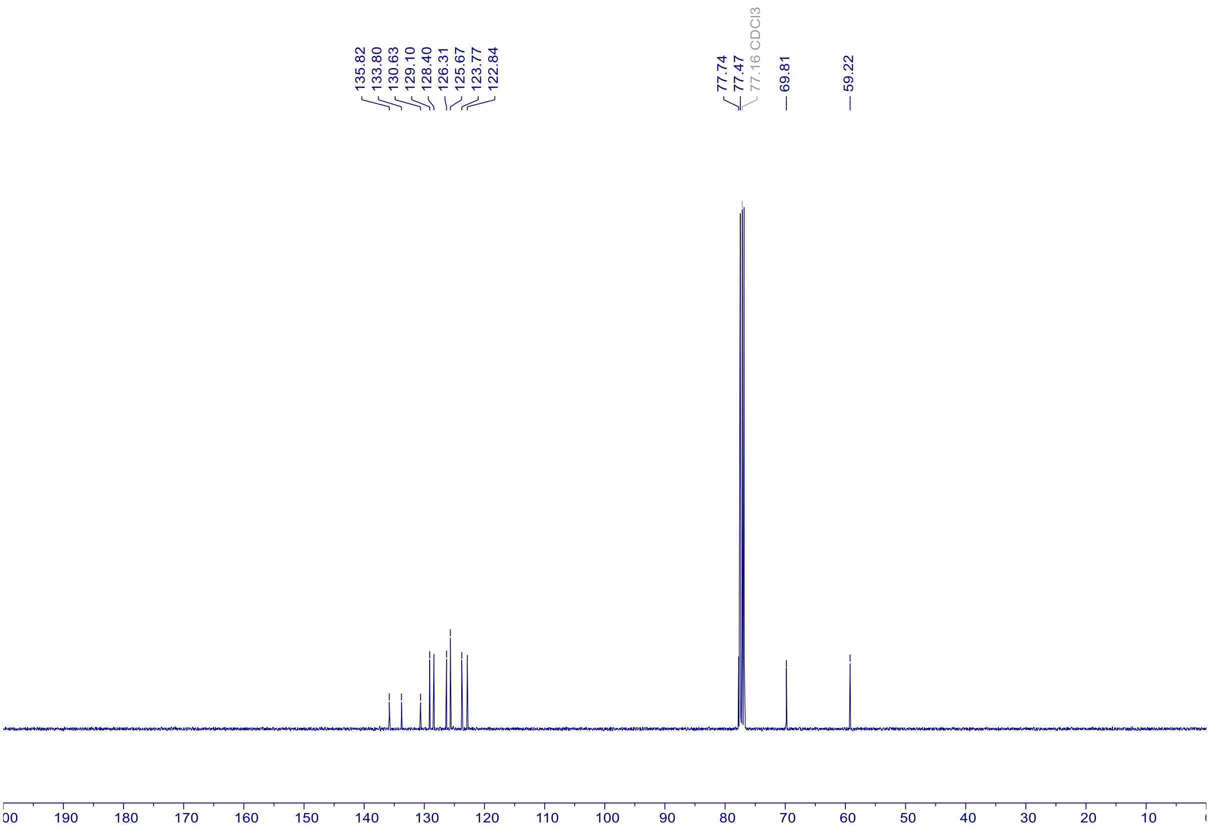

4al – ^{1}H NMR (400 MHz, $CDCl_3$)

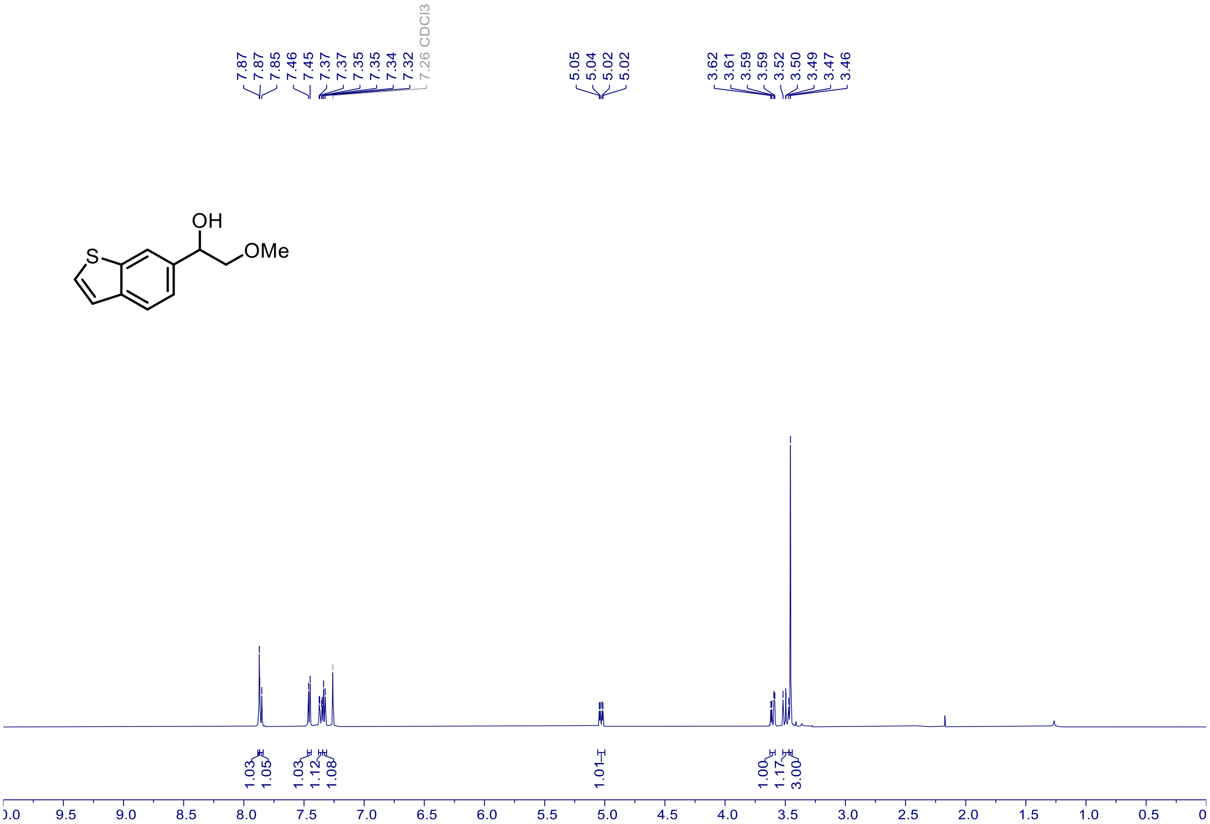

4al – ^{13}C NMR (101 MHz, $CDCl_3$)

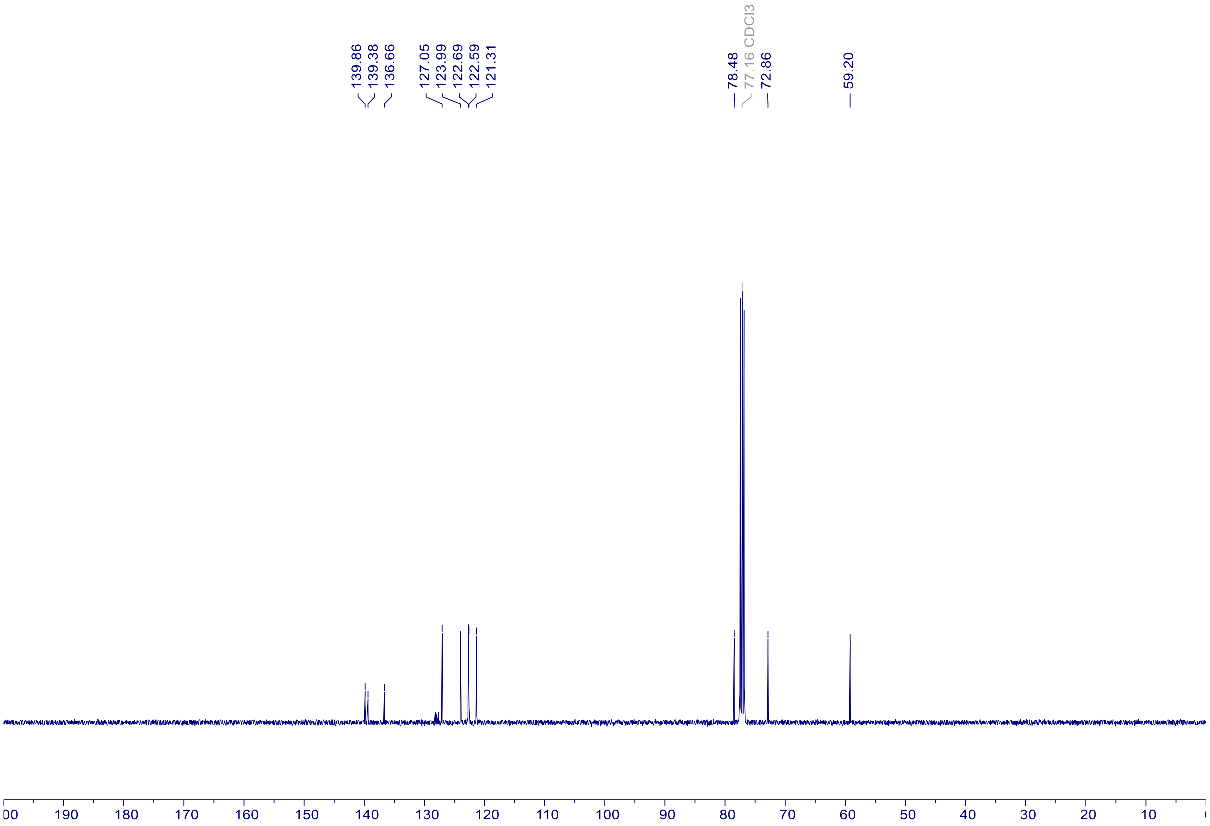

4am – ^{1}H NMR (400 MHz, $CDCl_3$)

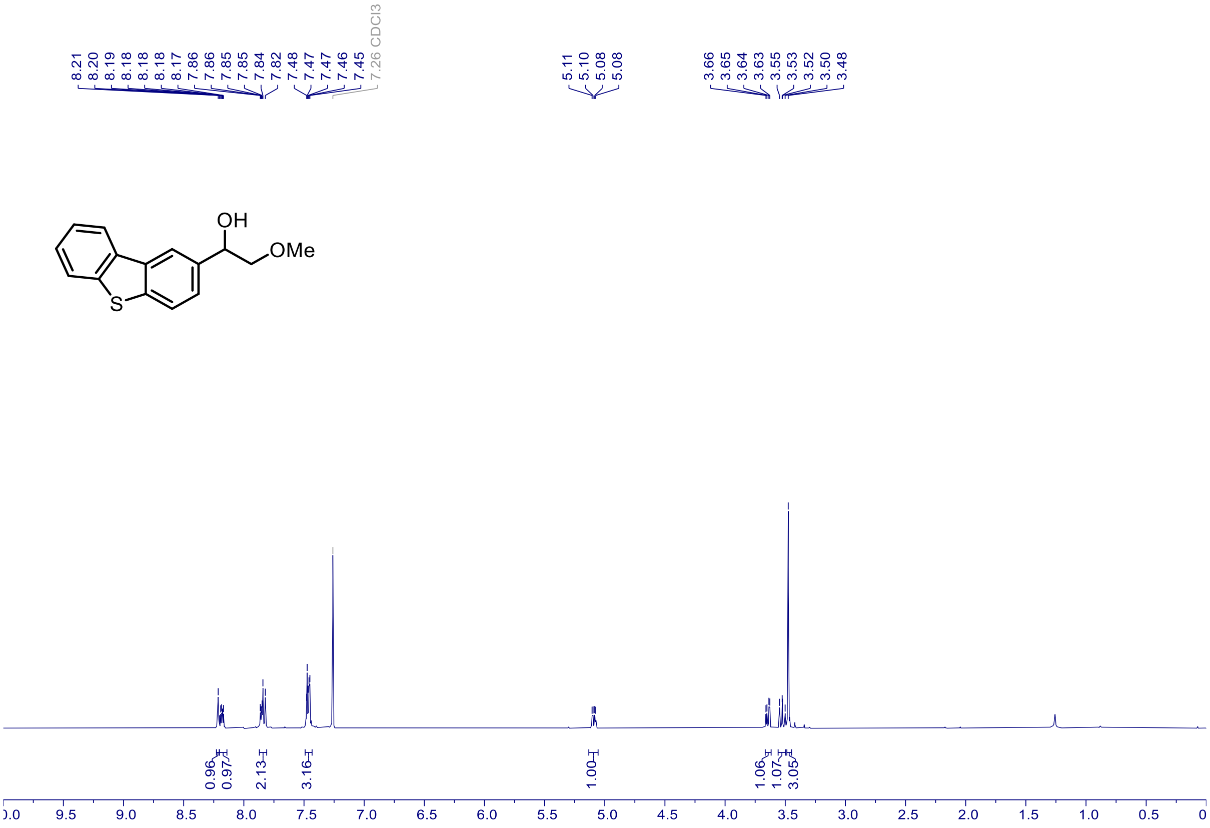

4am – ^{13}C NMR (101 MHz, $CDCl_3$)

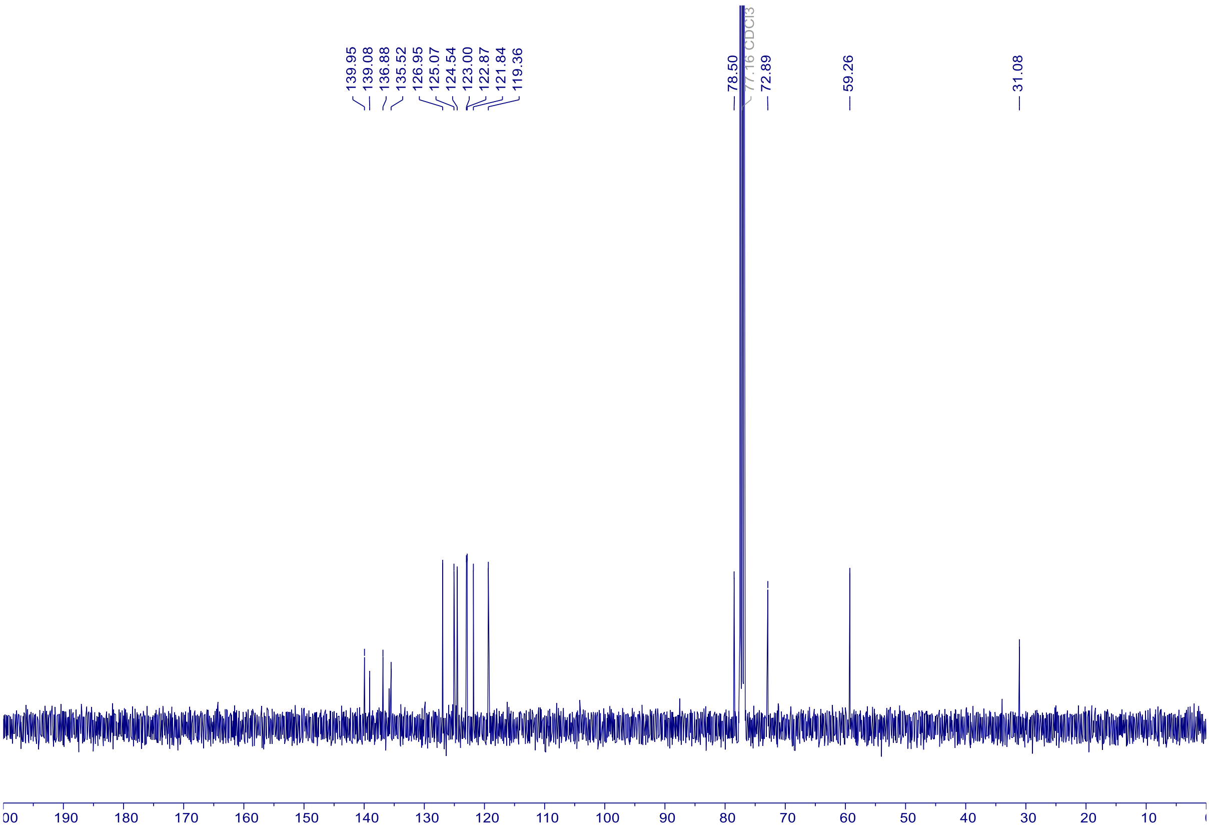

4an – ^{1}H NMR (400 MHz, $CDCl_3$)

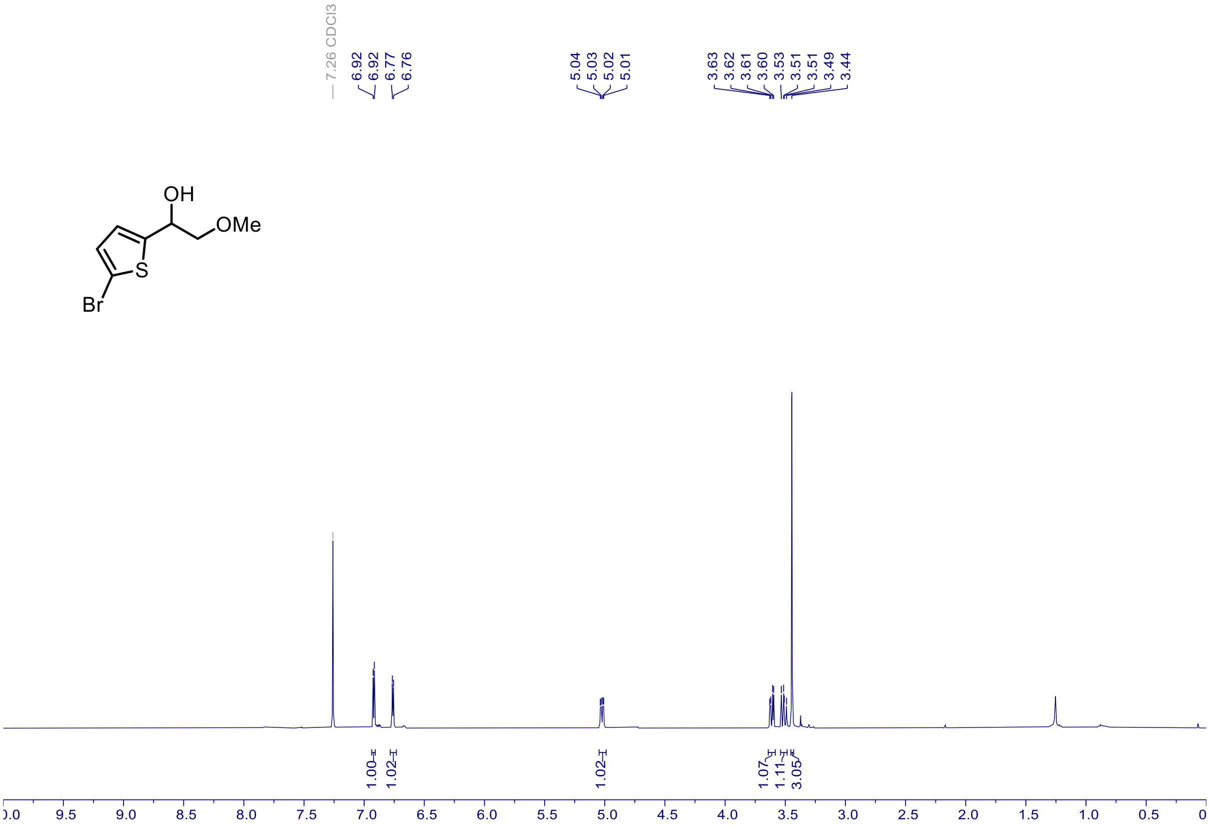

4an – ^{13}C NMR (101 MHz, $CDCl_3$)

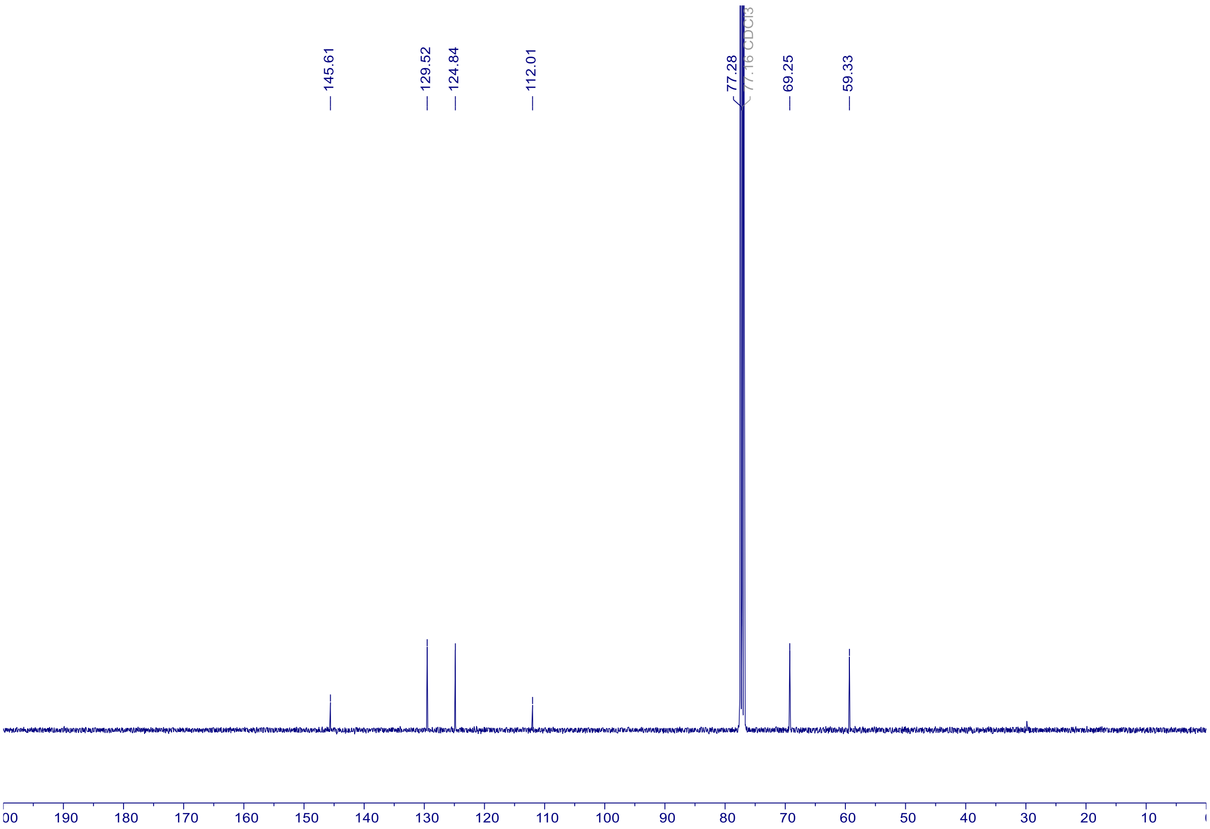

4ao – ^{1}H NMR (400 MHz, $CDCl_3$)

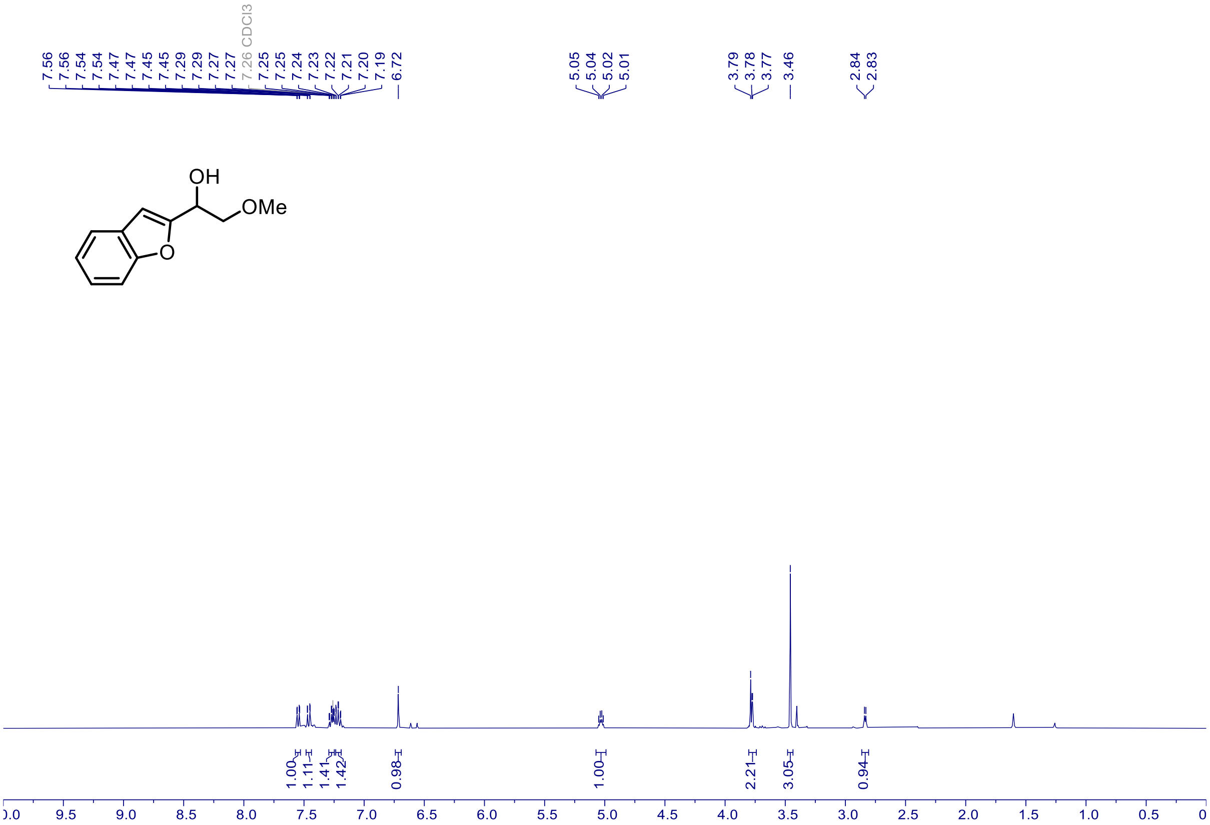

4ao – ^{13}C NMR (101 MHz, $CDCl_3$)

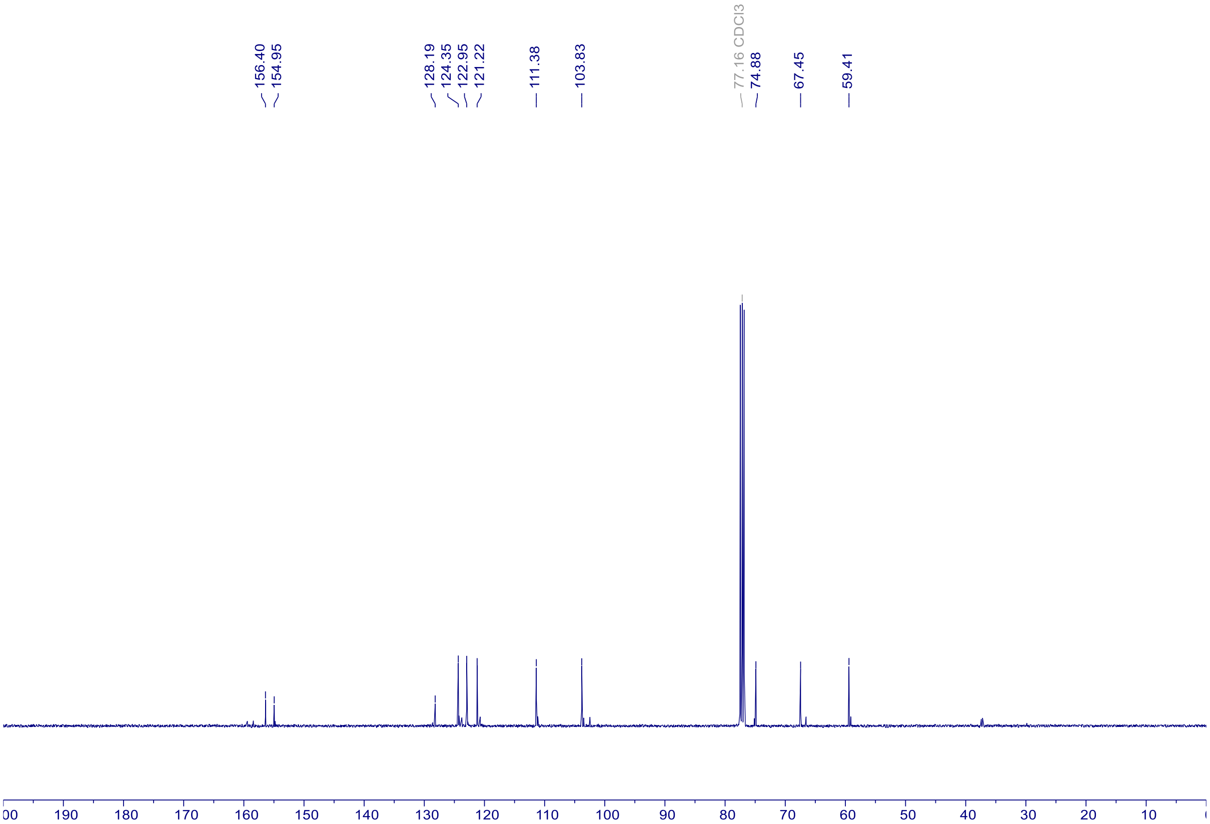

4ap – ^{1}H NMR (400 MHz, $CDCl_3$)

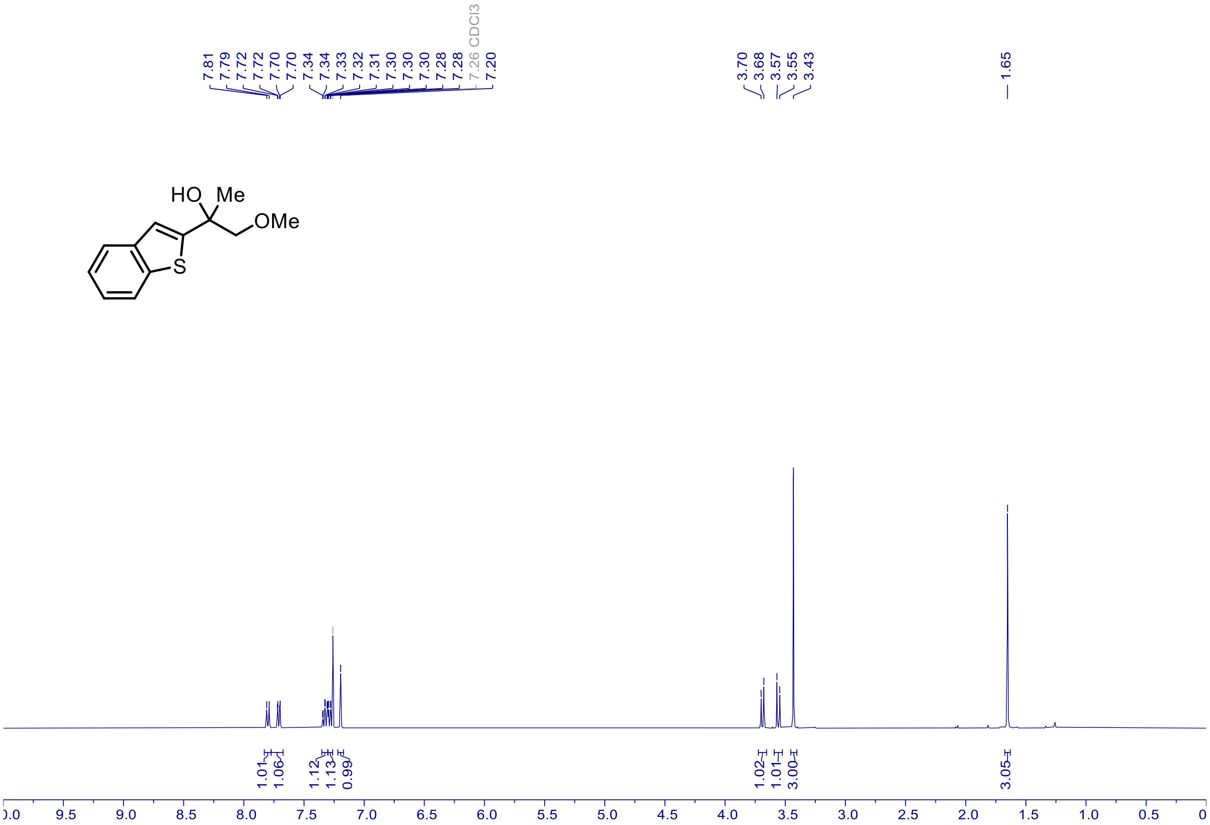

4ap – ^{13}C NMR (101 MHz, $CDCl_3$)

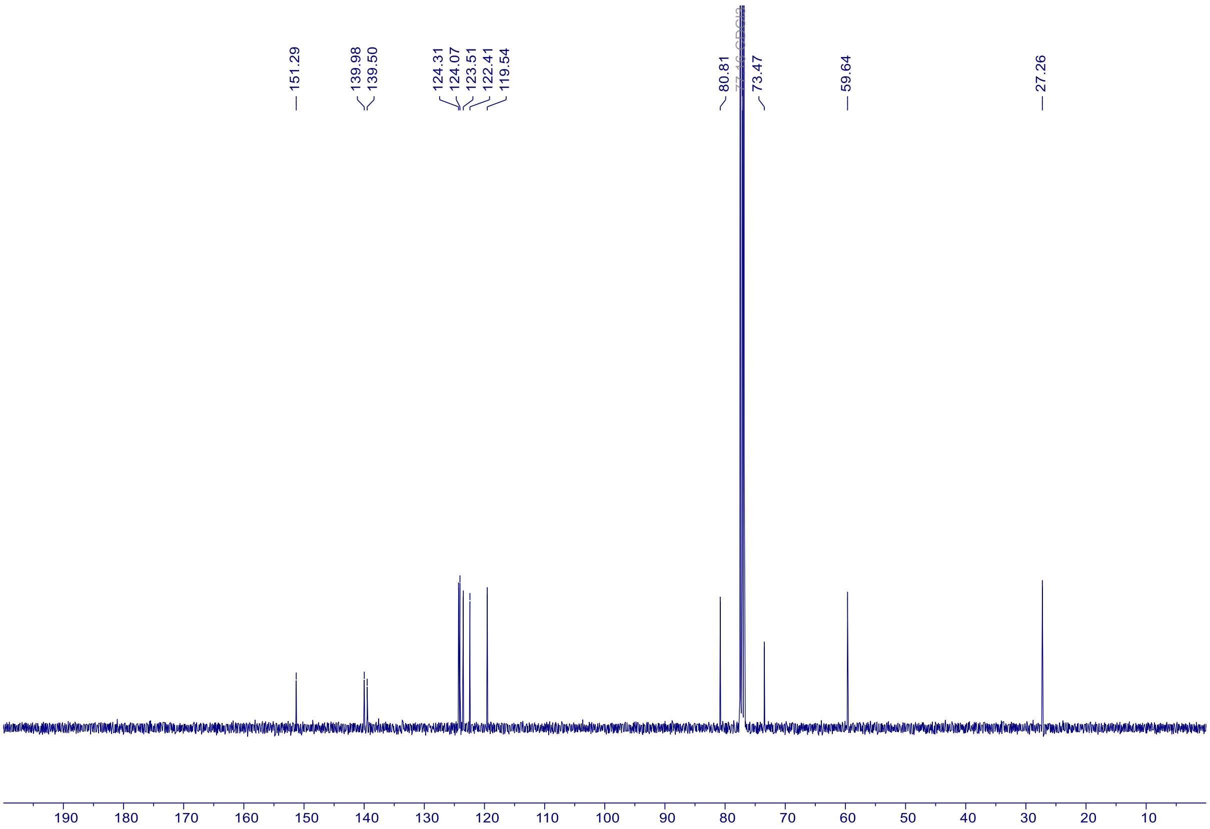

4aq – ^{1}H NMR (400 MHz, $CDCl_3$)

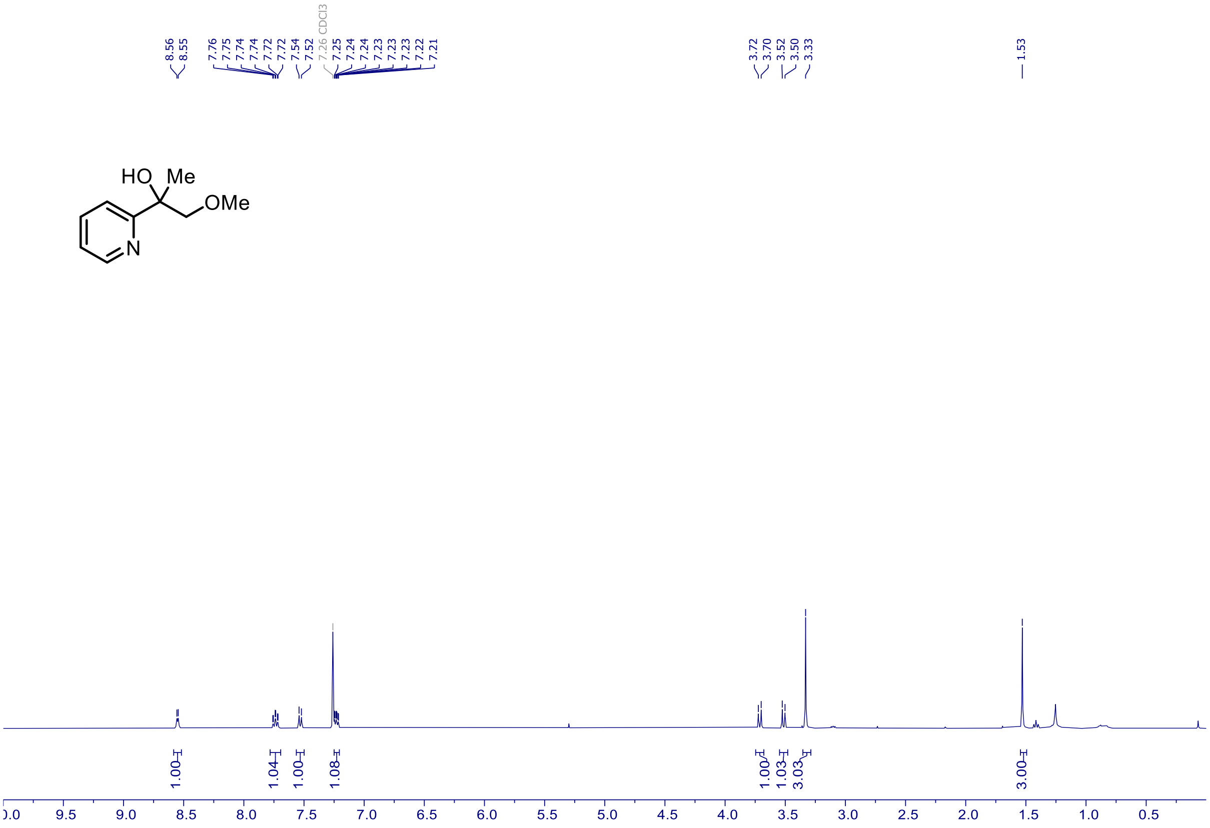

4aq – ^{13}C NMR (101 MHz, $CDCl_3$)

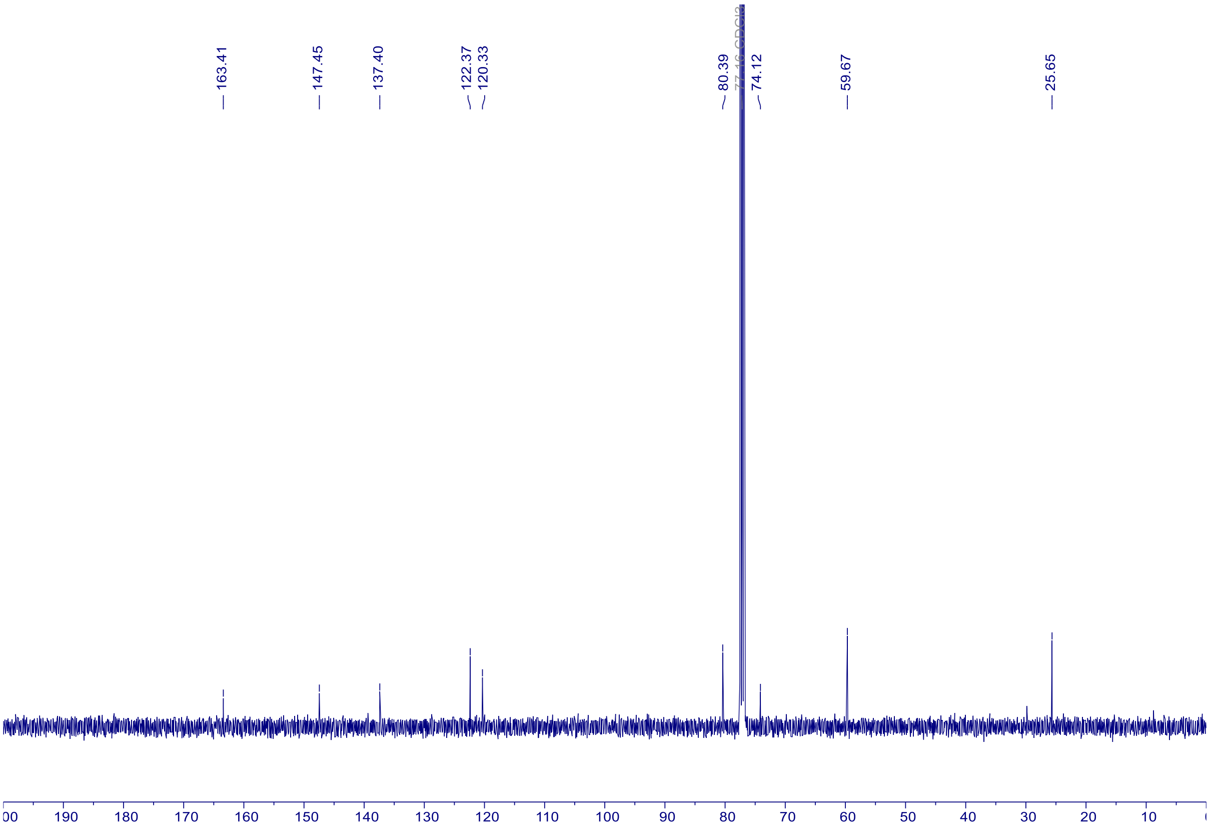

4ar – ^{1}H NMR (400 MHz, $CDCl_3$, 1.3:1 mixture of diastereoisomers)

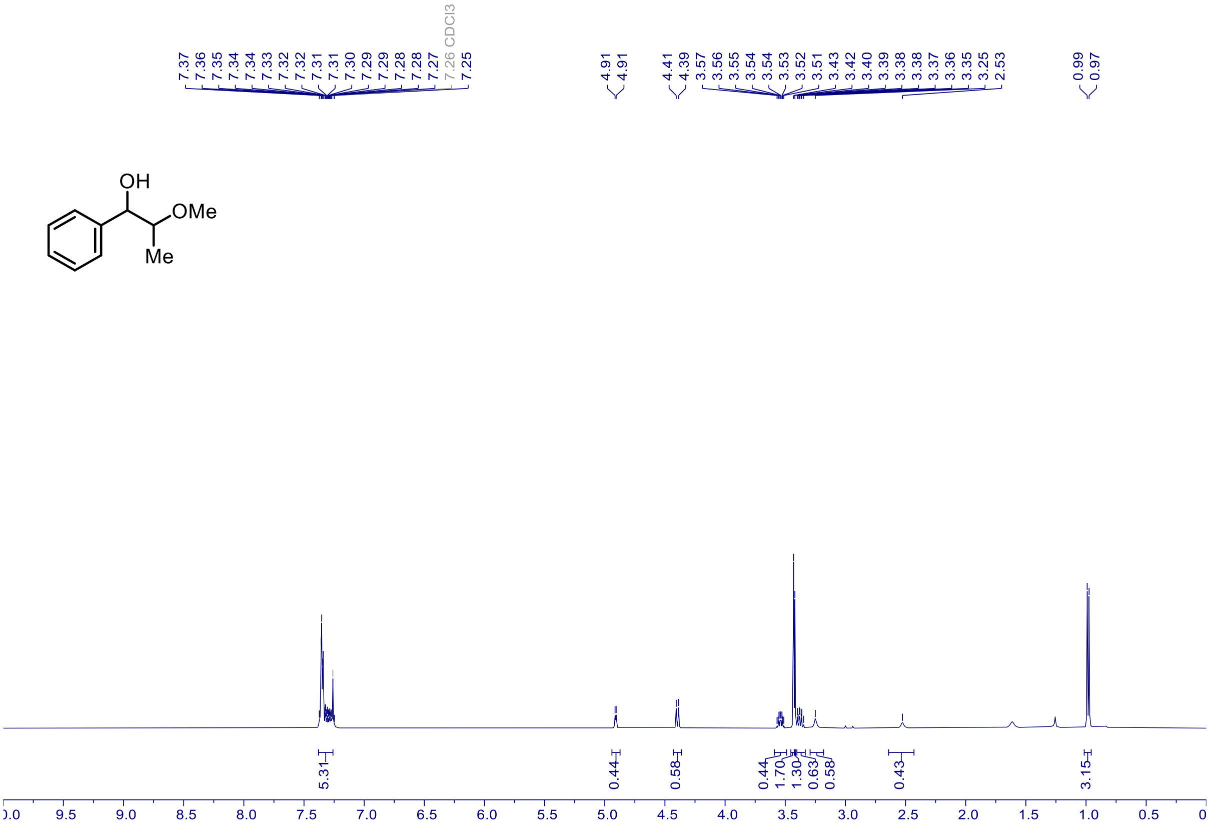

4ar – ^{13}C NMR (101 MHz, $CDCl_3$, 1.3:1 mixture of diastereoisomers)

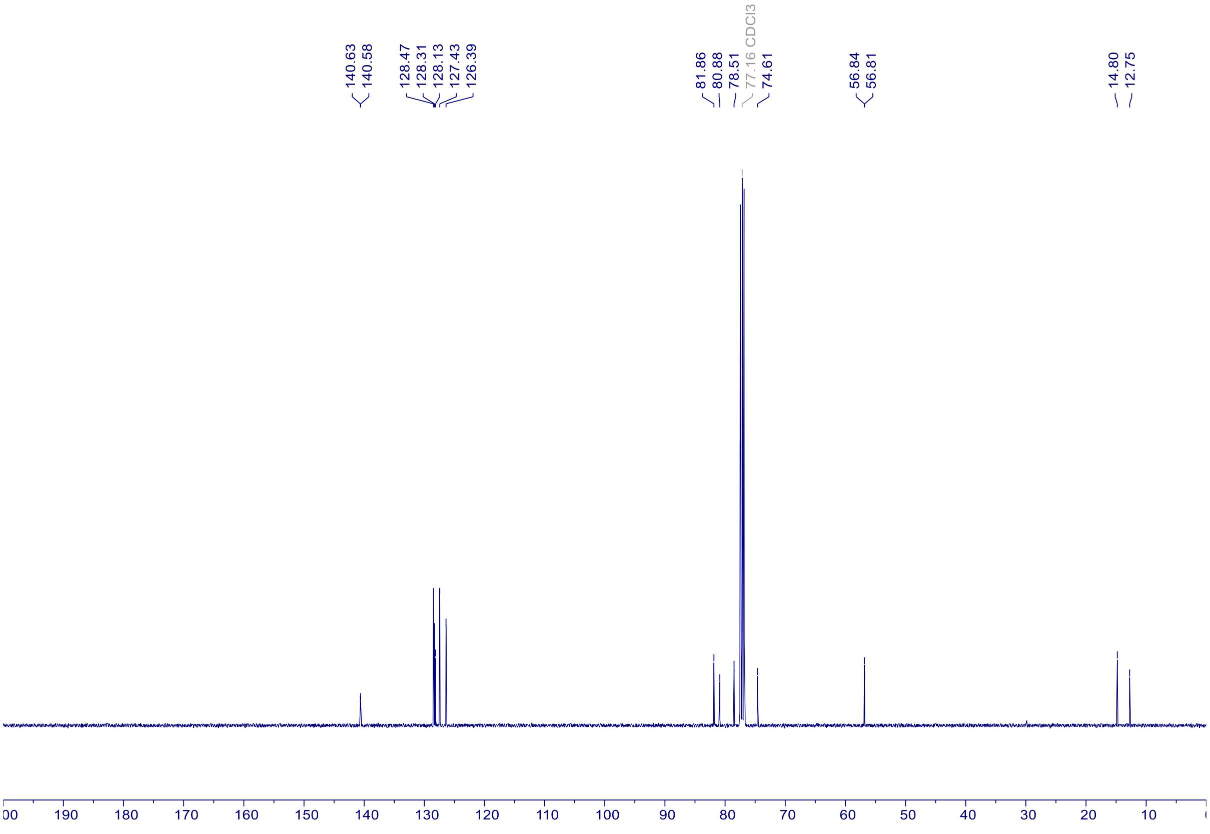

4as – ^{1}H NMR (400 MHz, $CDCl_3$, 1.1:1 mixture of diastereoisomers)

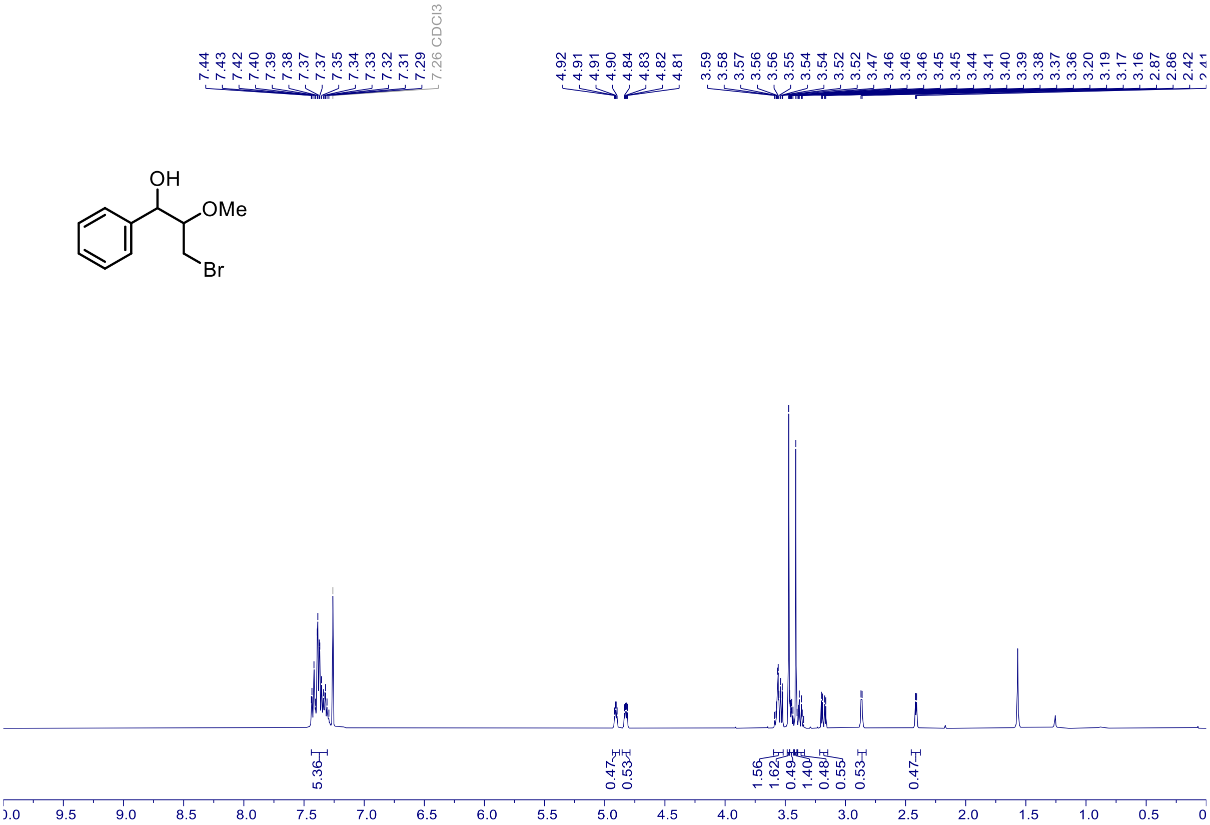

4as – ^{13}C NMR (101 MHz, $CDCl_3$, 1.1:1 mixture of diastereoisomers)

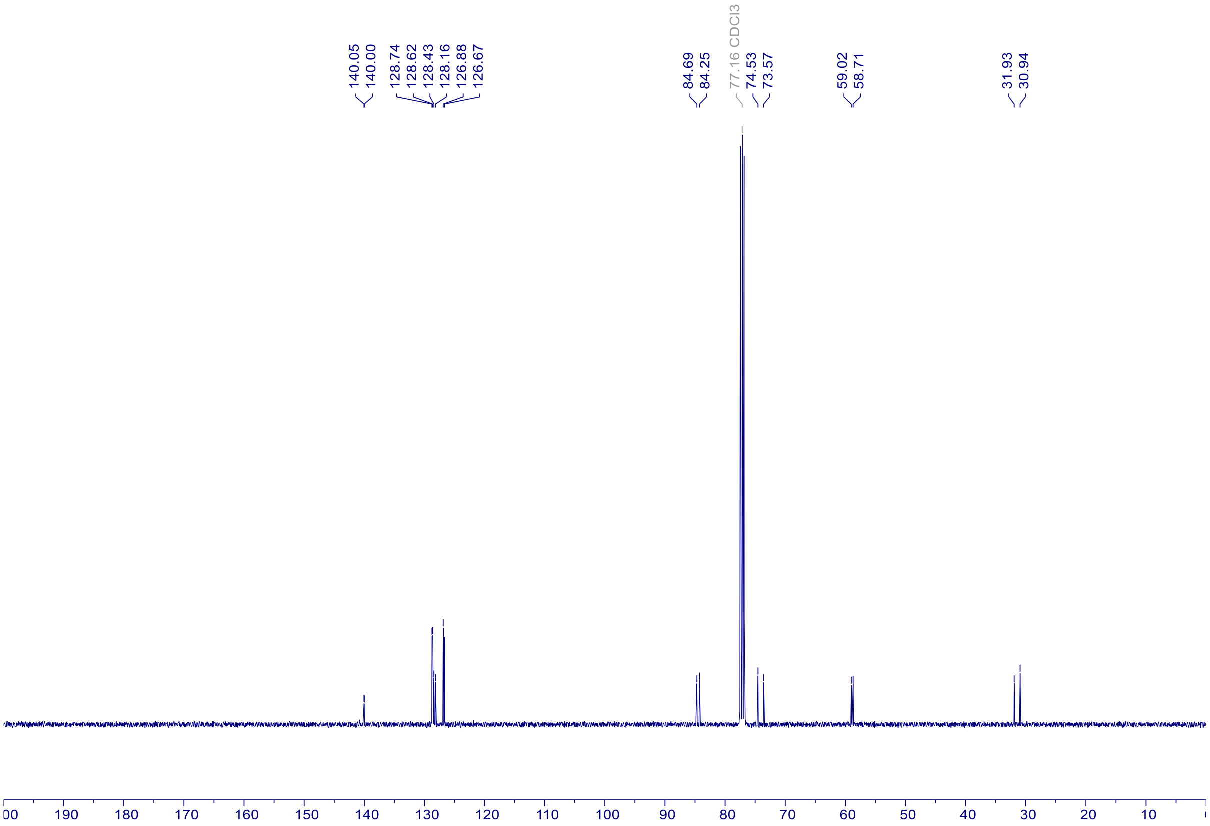

4at – ^{1}H NMR (400 MHz, $CDCl_3$, 1:1 mixture of diastereoisomers)

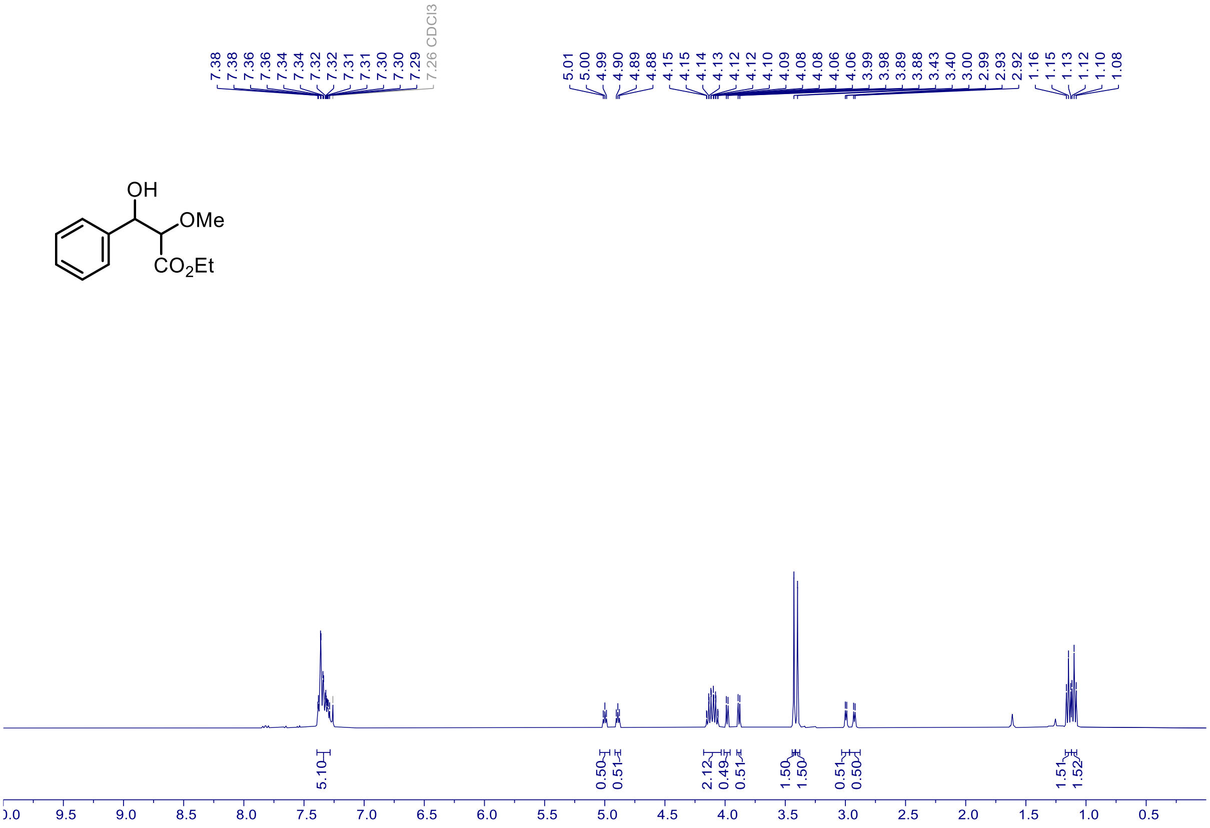

4at – ^{13}C NMR (101 MHz, $CDCl_3$, 1:1 mixture of diastereoisomers)

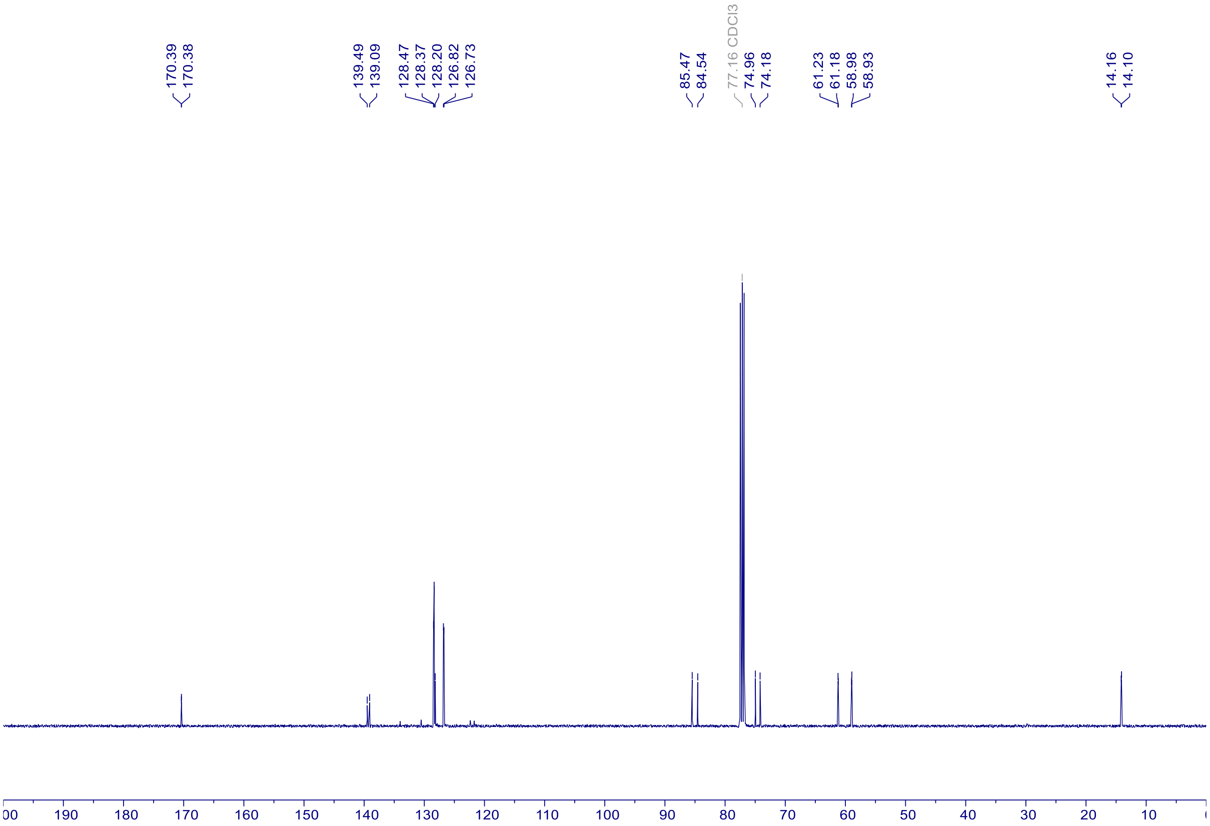

4au – ^{1}H NMR (500 MHz, $CDCl_3$, 2:1 mixture of diastereoisomers)

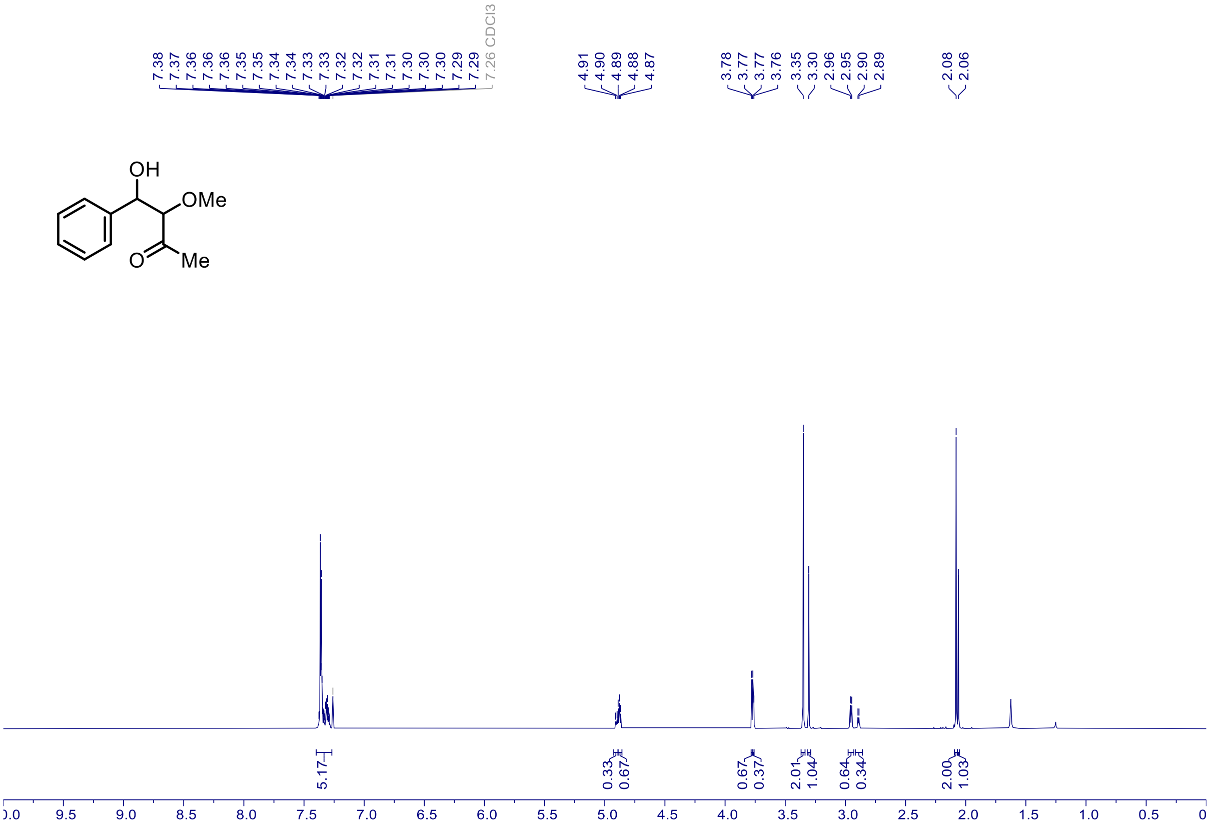

4au – ^{13}C NMR (126 MHz, $CDCl_3$, 2:1 mixture of diastereoisomers)

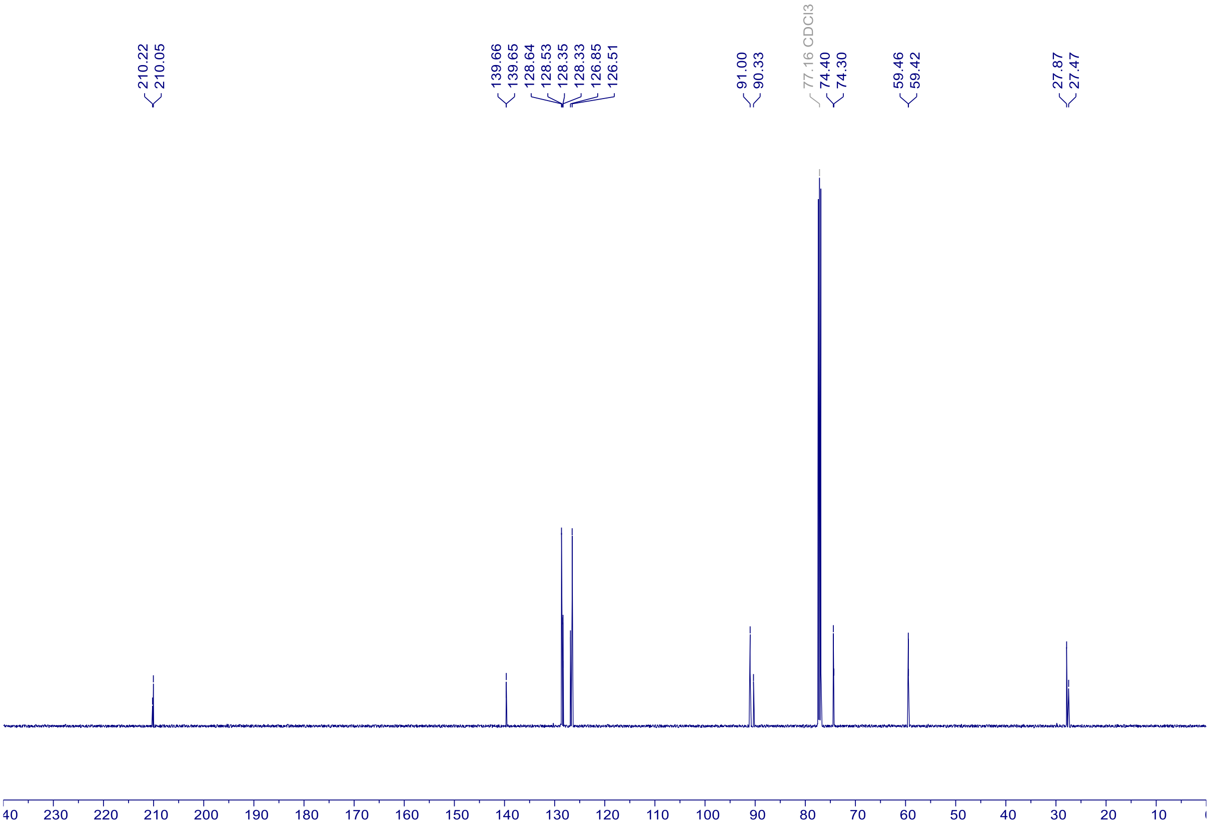

4av – ^{1}H NMR (400 MHz, CDCl$_3$)

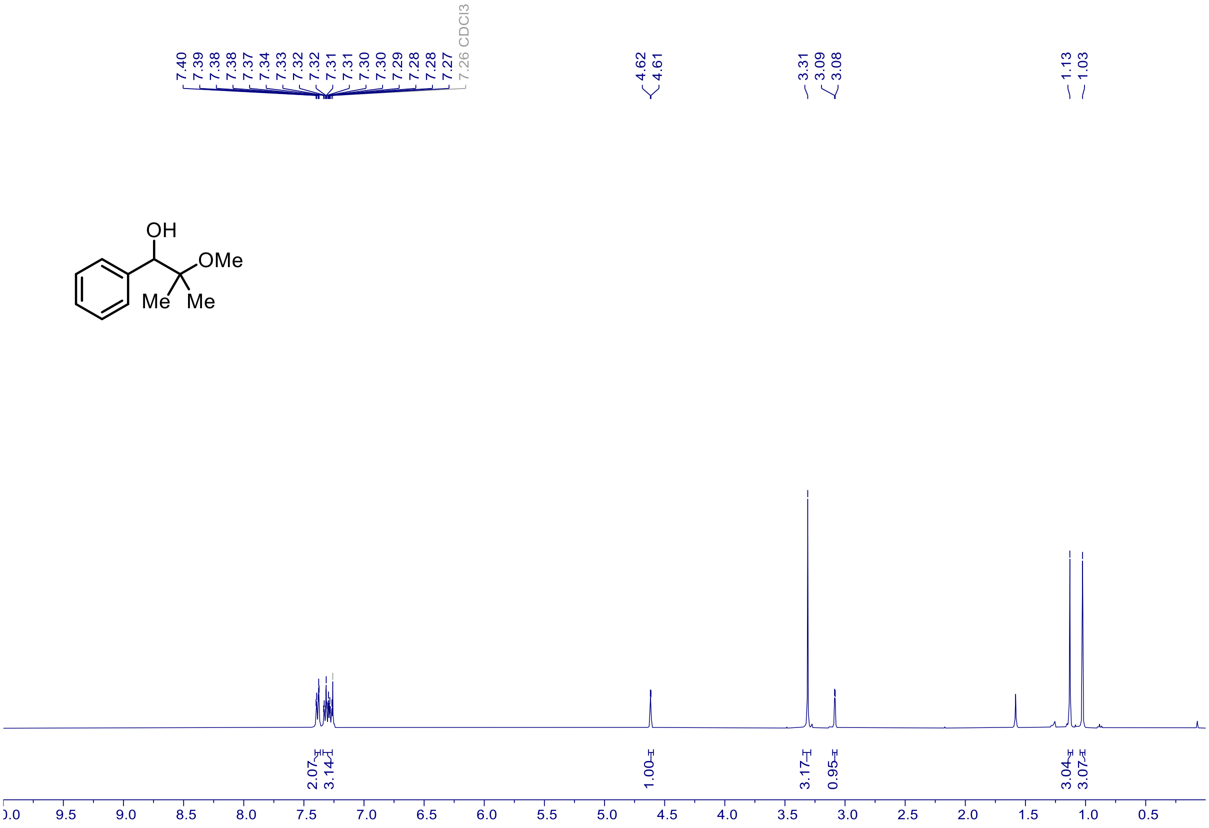

4av – ^{13}C NMR (101 MHz, CDCl$_3$)

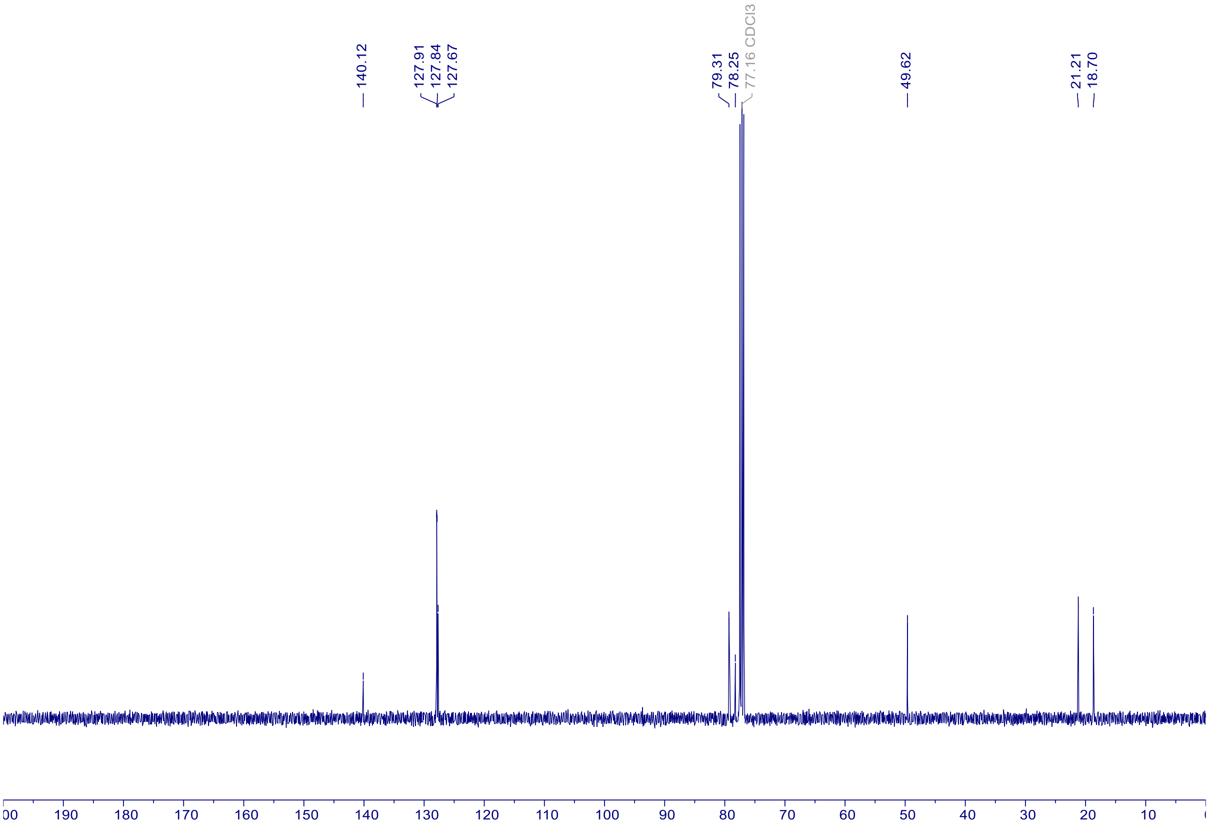

4aw – ^{1}H NMR (500 MHz, $CDCl_3$, 2.8:1 mixture of diastereoisomers)

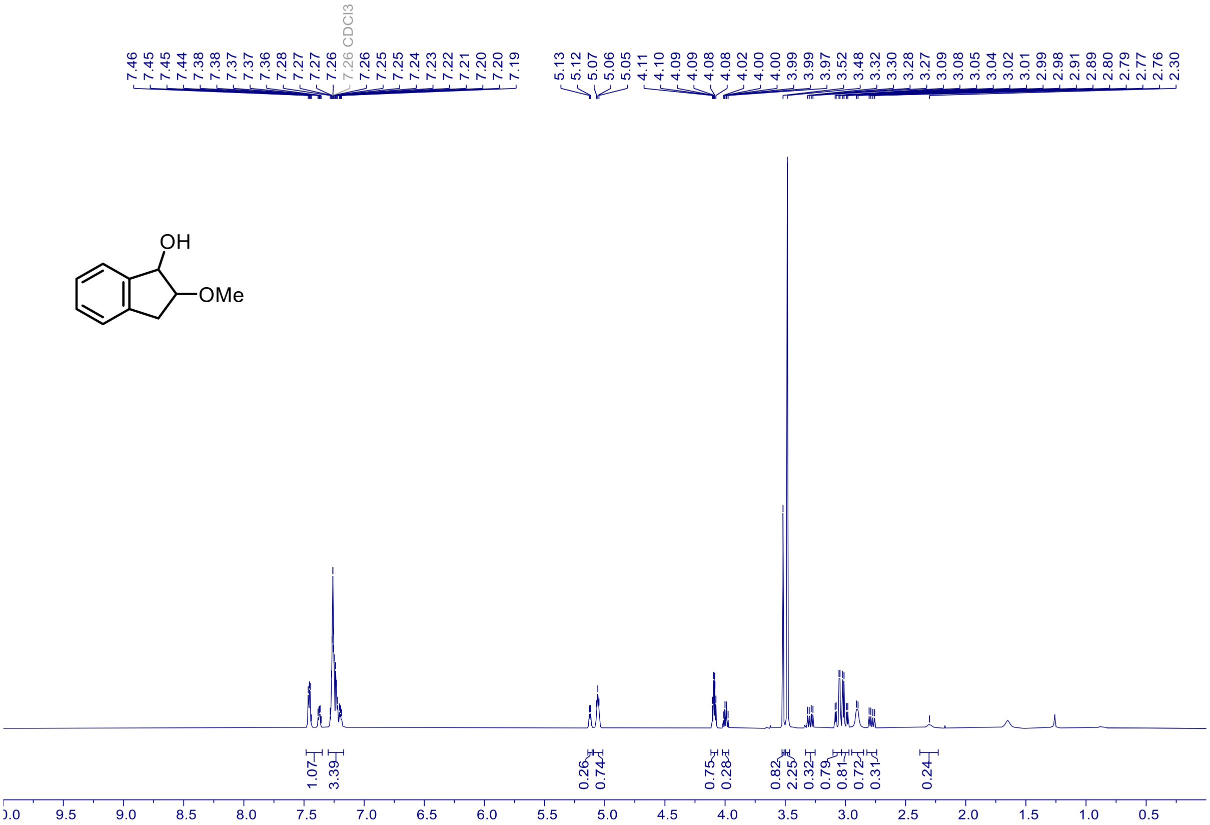

4aw – ^{13}C NMR (126 MHz, $CDCl_3$, 2.8:1 mixture of diastereoisomers)

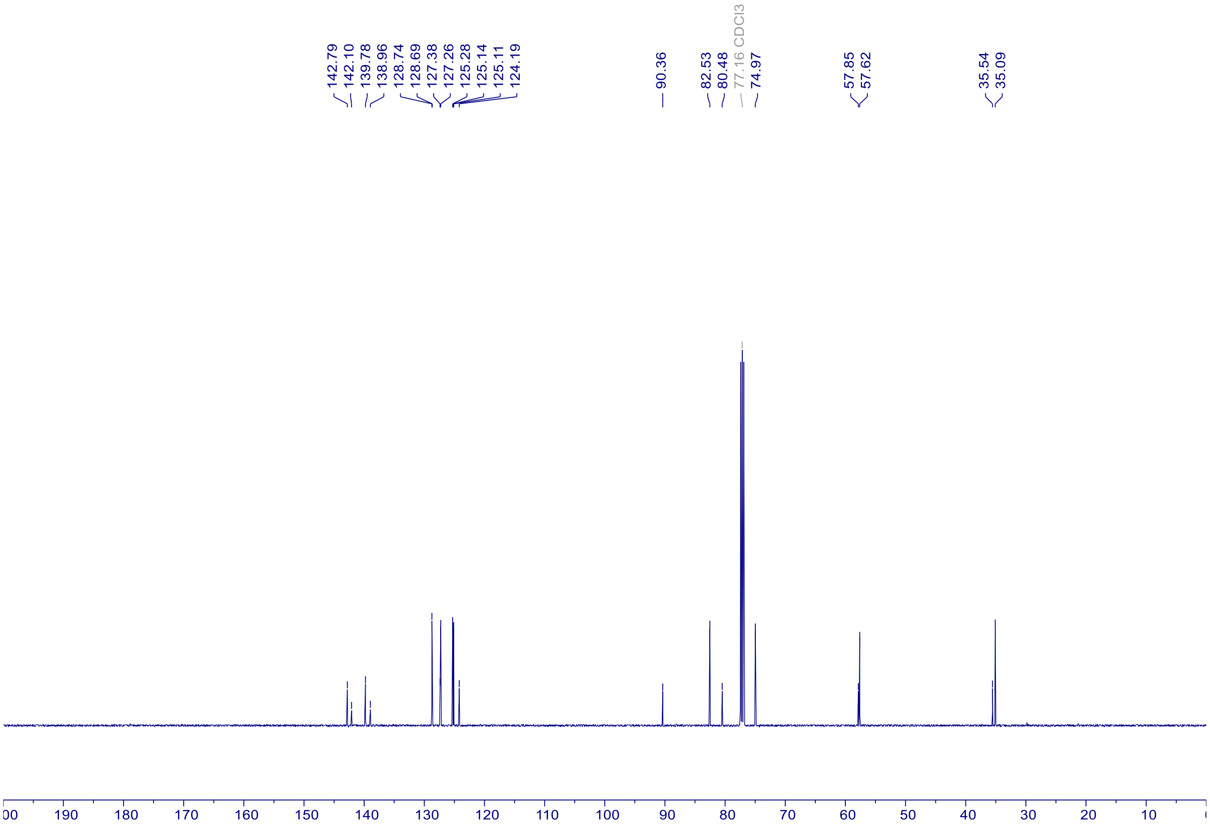

4ax – ^{1}H NMR (400 MHz, $CDCl_3$, 1.3:1 mixture of diastereoisomers)

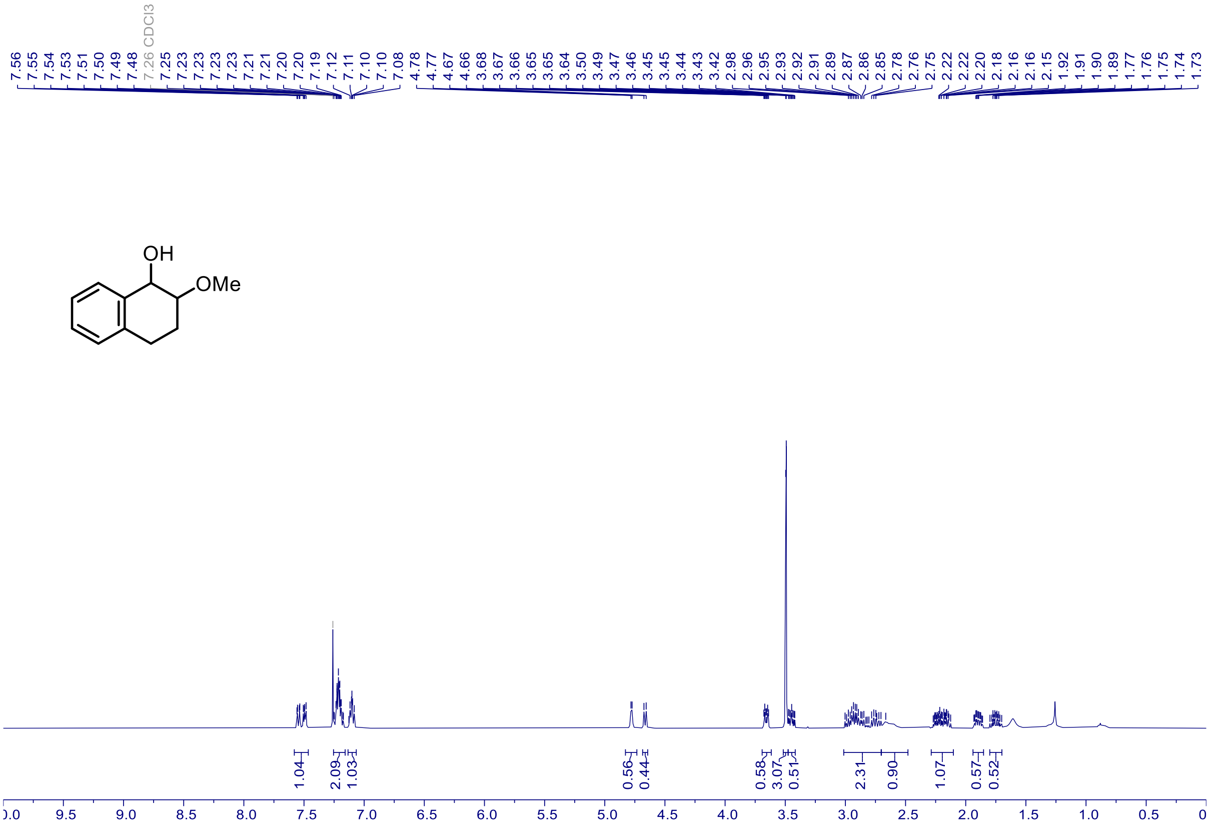

4ax – ^{13}C NMR (101 MHz, $CDCl_3$, 1.3:1 mixture of diastereoisomers)

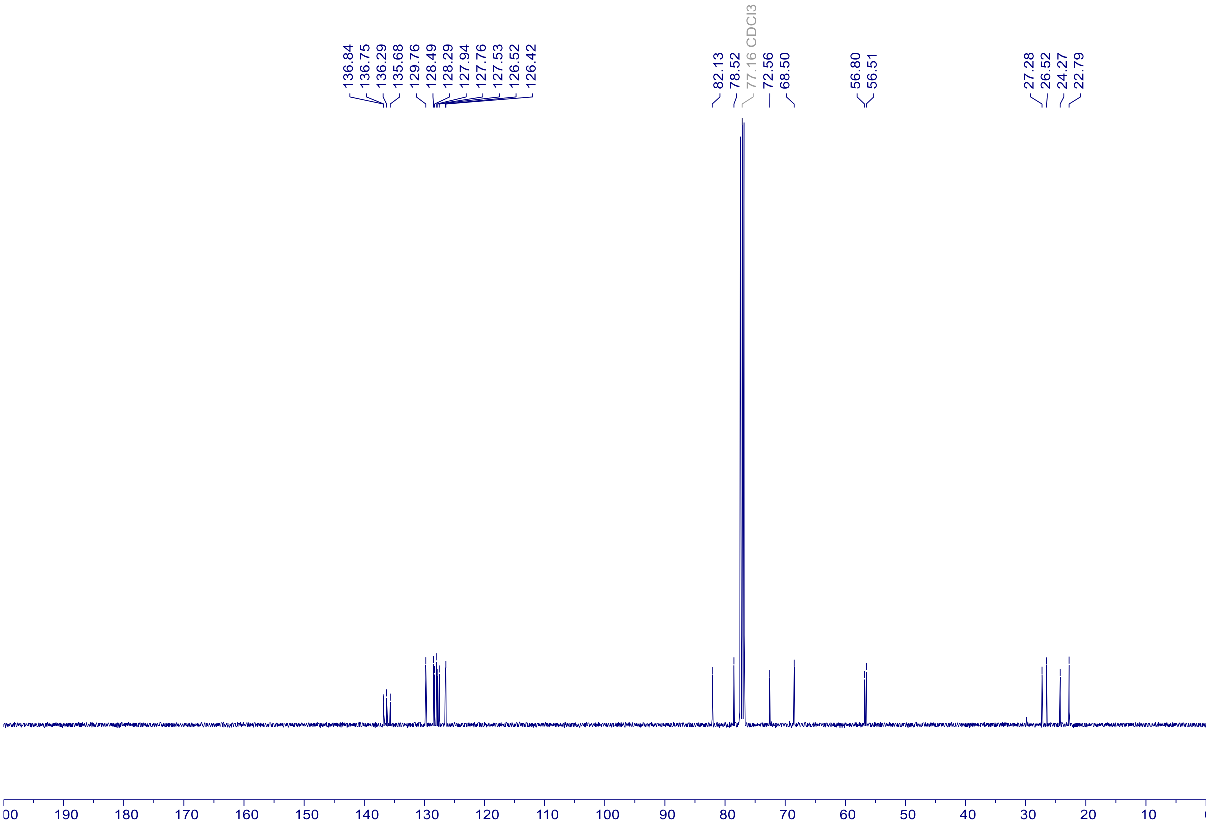

4ay – ^{1}H NMR (400 MHz, $CDCl_3$)

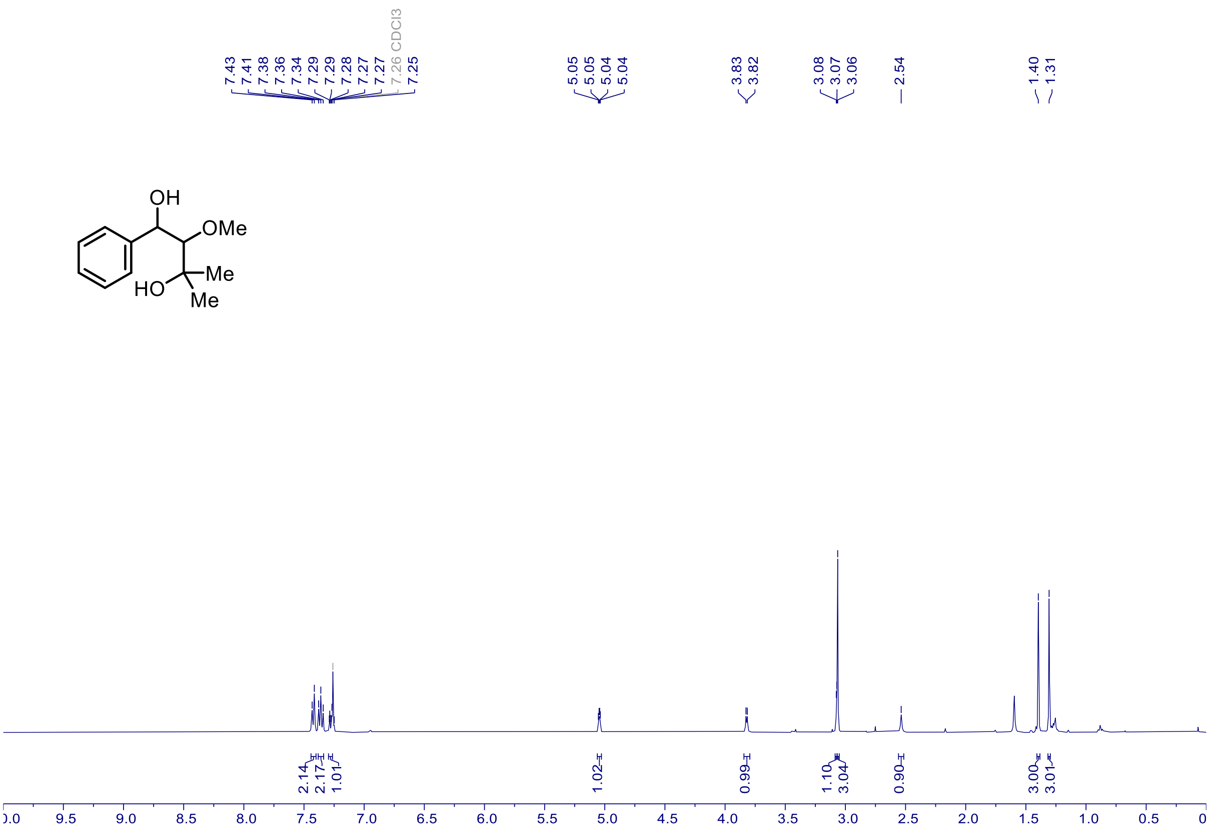

4ay – ^{13}C NMR (101 MHz, $CDCl_3$)

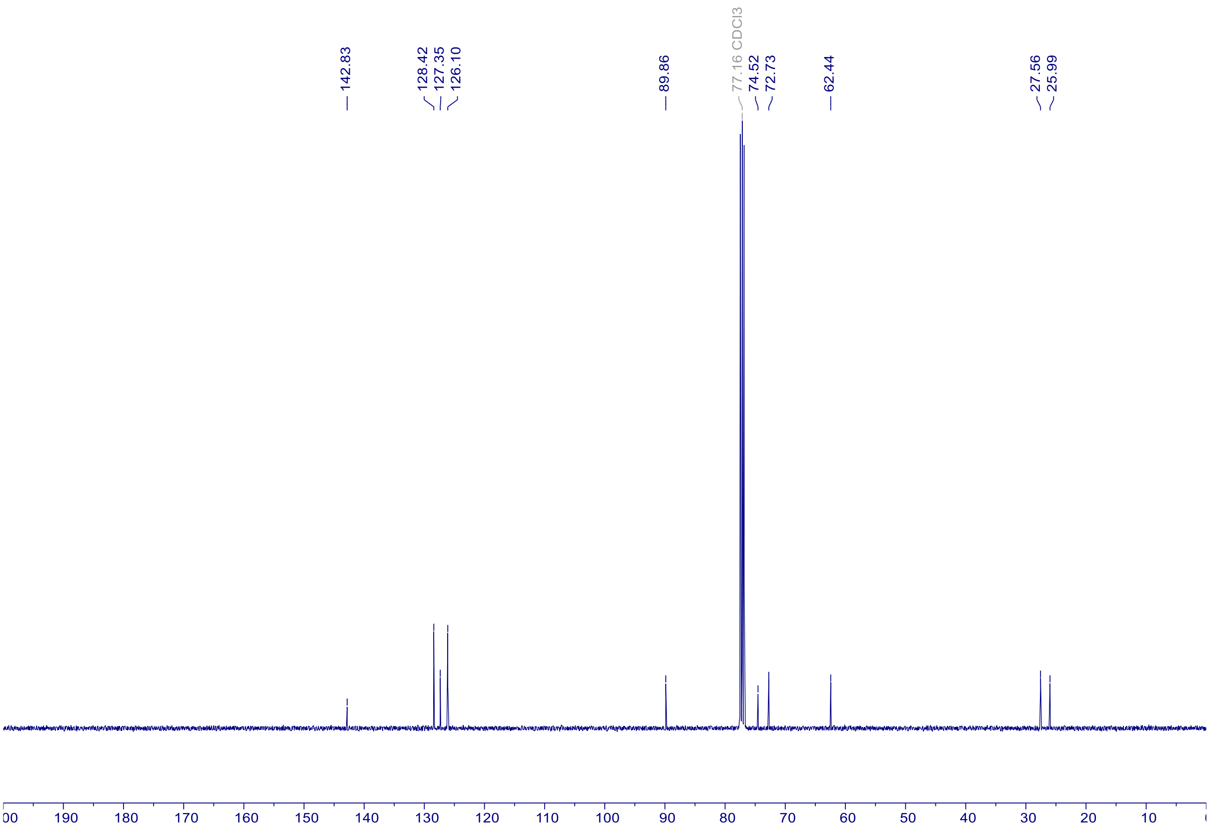

4az – ^{1}H NMR (400 MHz, $CDCl_3$, 1.7:1 mixture of diastereoisomers)

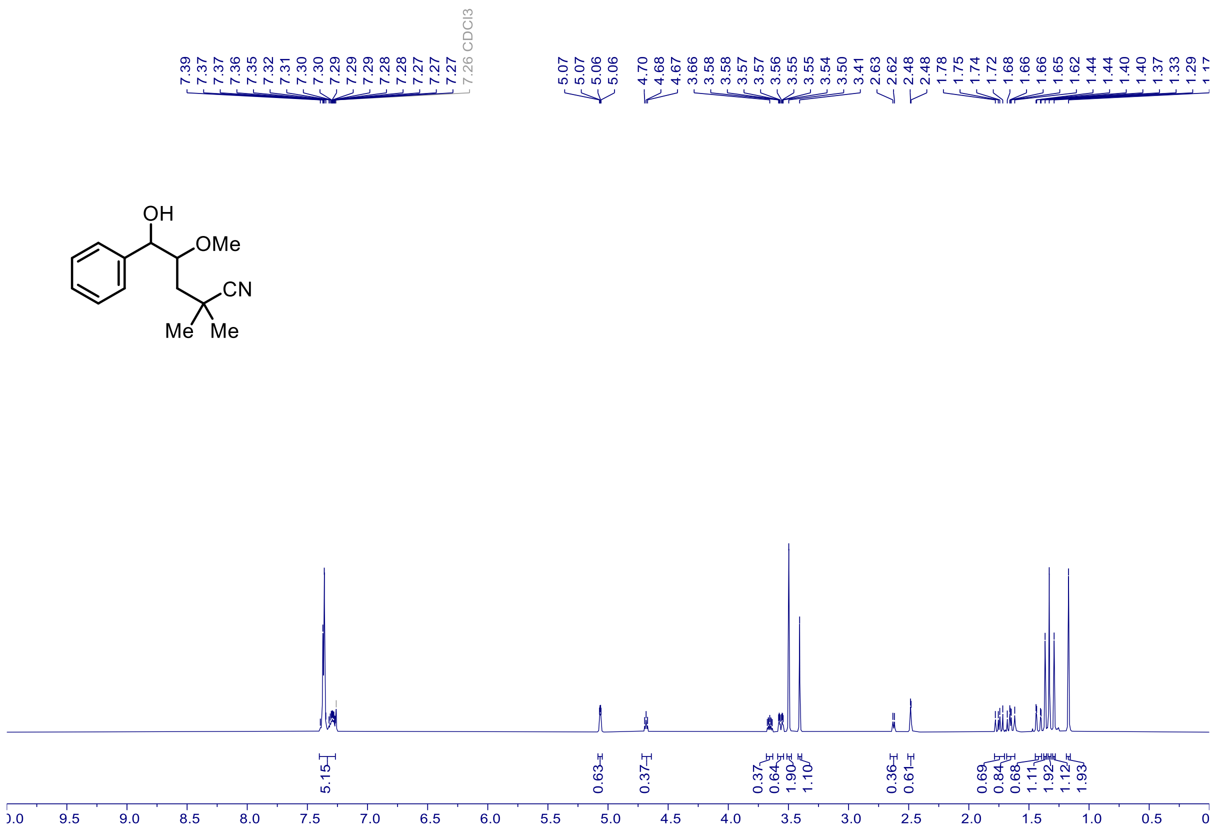

4az – ^{13}C NMR (101 MHz, $CDCl_3$, 1.7:1 mixture of diastereoisomers)

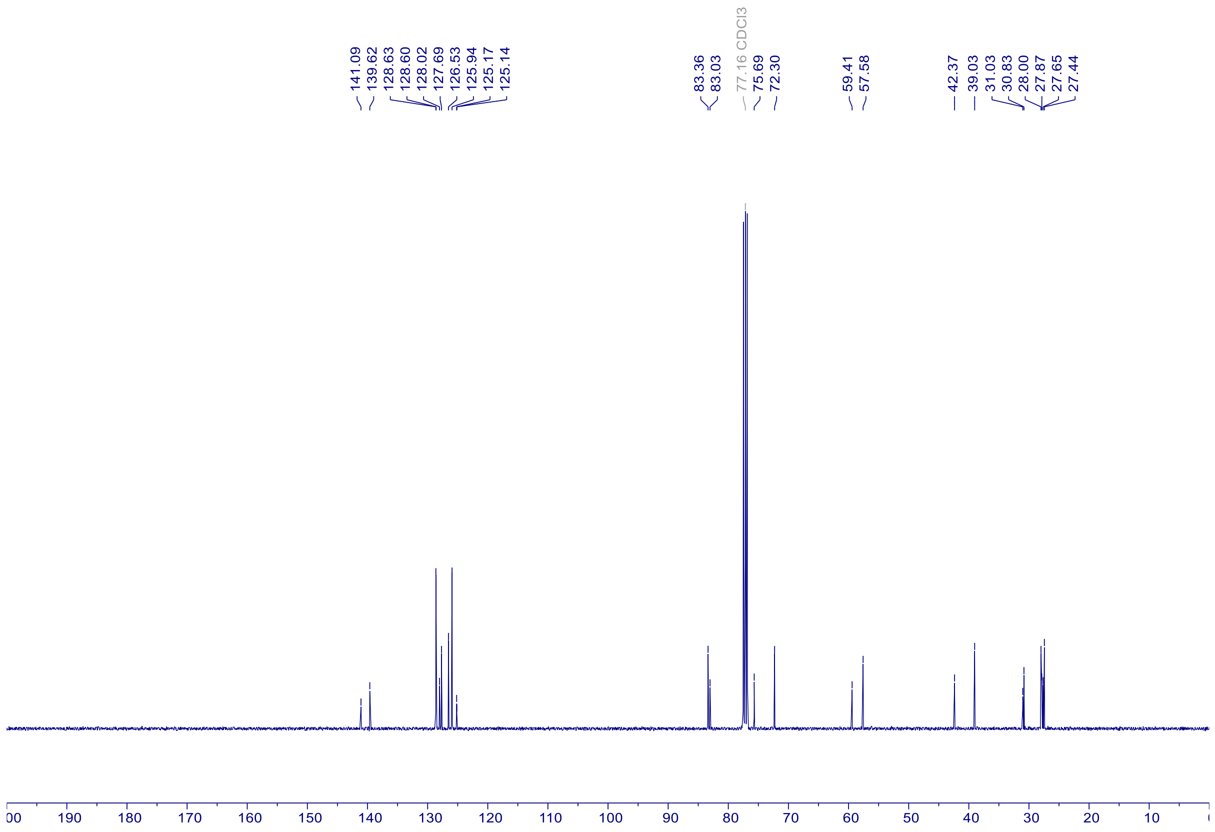

4ba – ^{1}H NMR (400 MHz, $CDCl_3$)

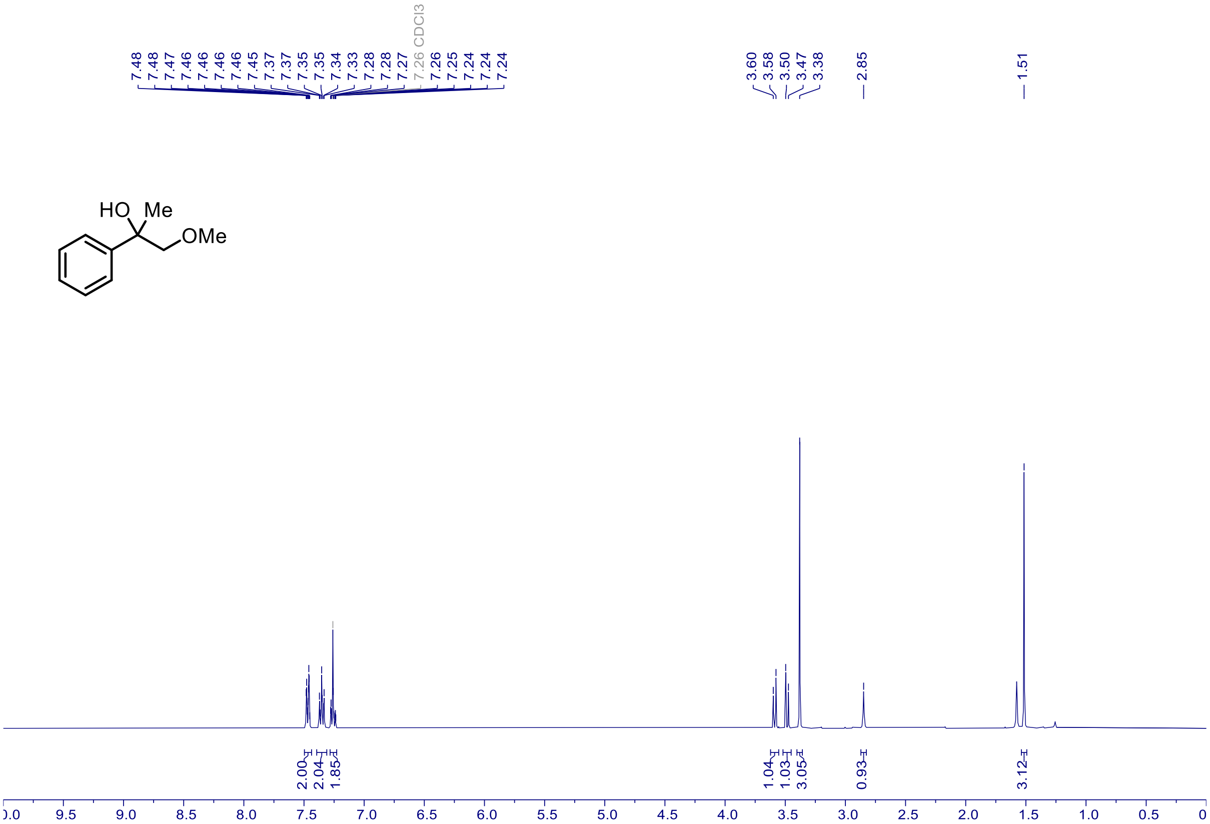

4ba – ^{13}C NMR (101 MHz, $CDCl_3$)

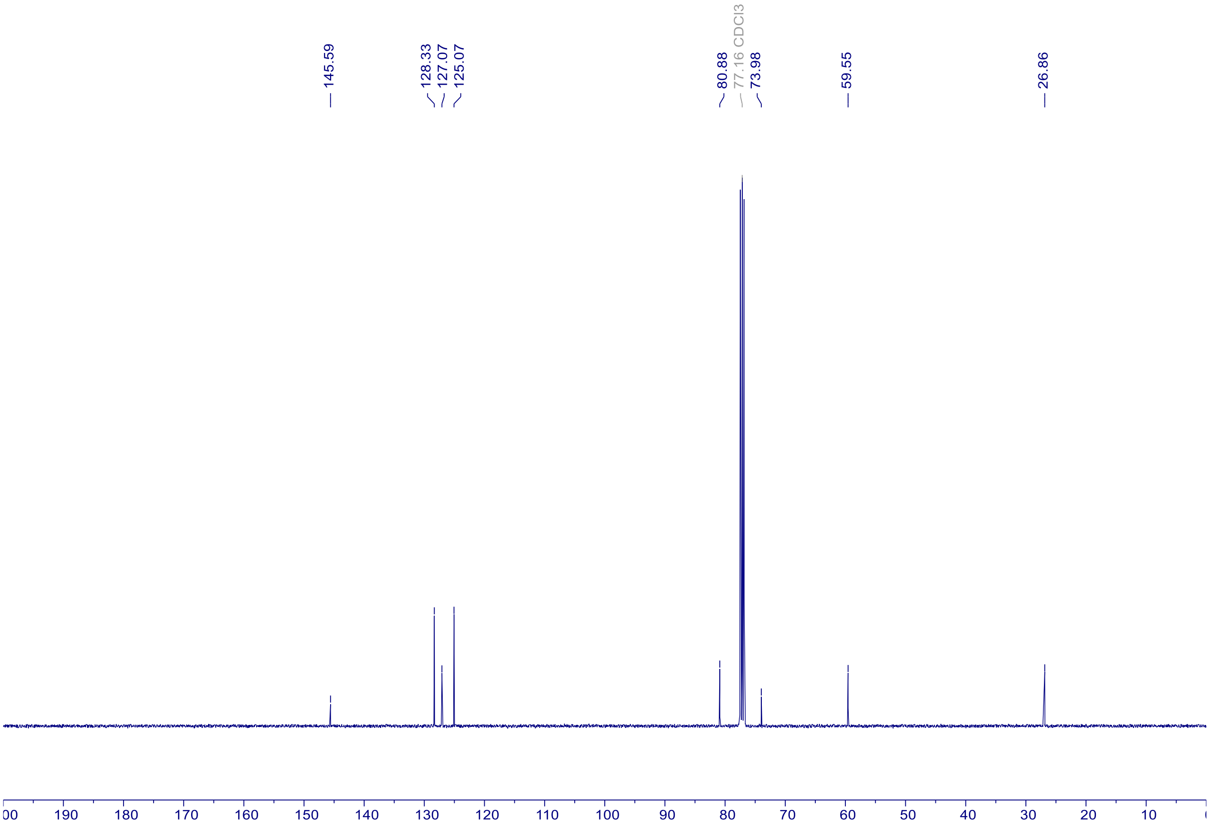

4bb – ^{1}H NMR (400 MHz, $CDCl_3$)

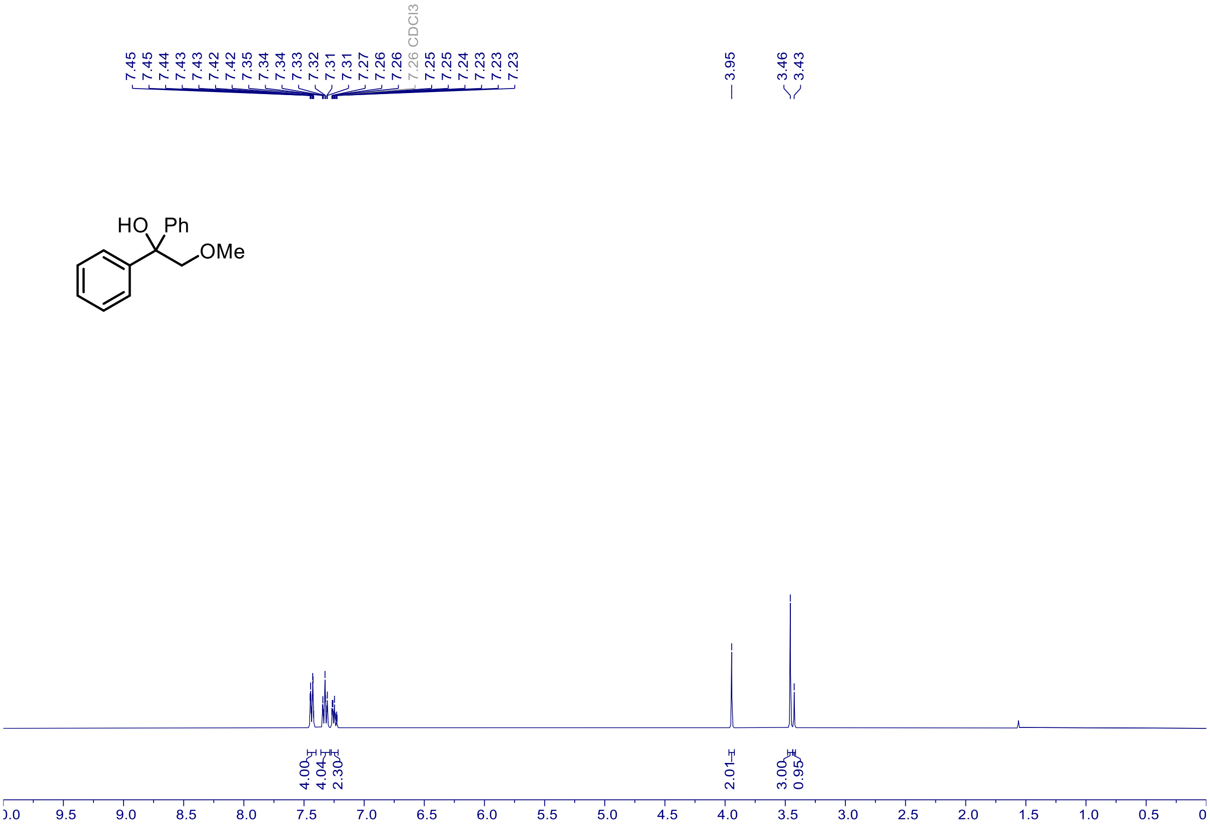

4bb – ^{13}C NMR (101 MHz, $CDCl_3$)

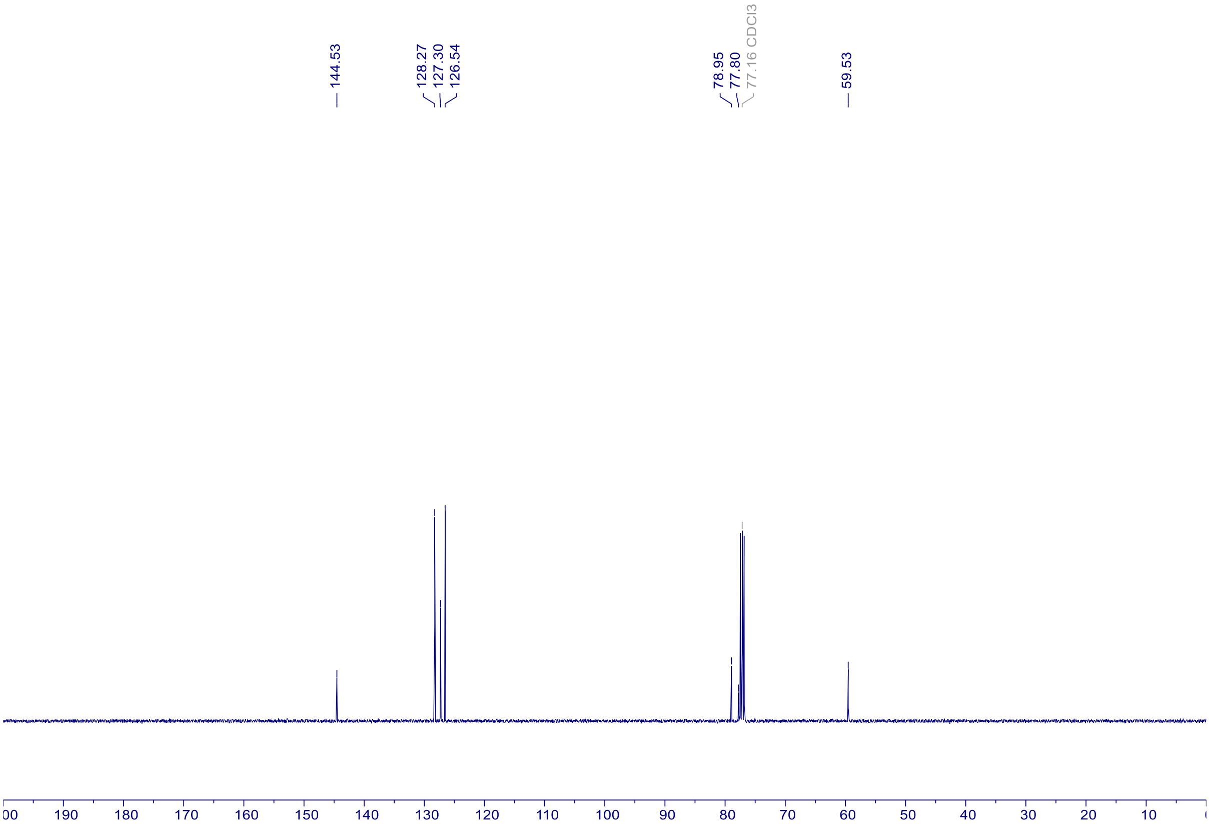

4bc – ^{1}H NMR (400 MHz, $CDCl_3$)

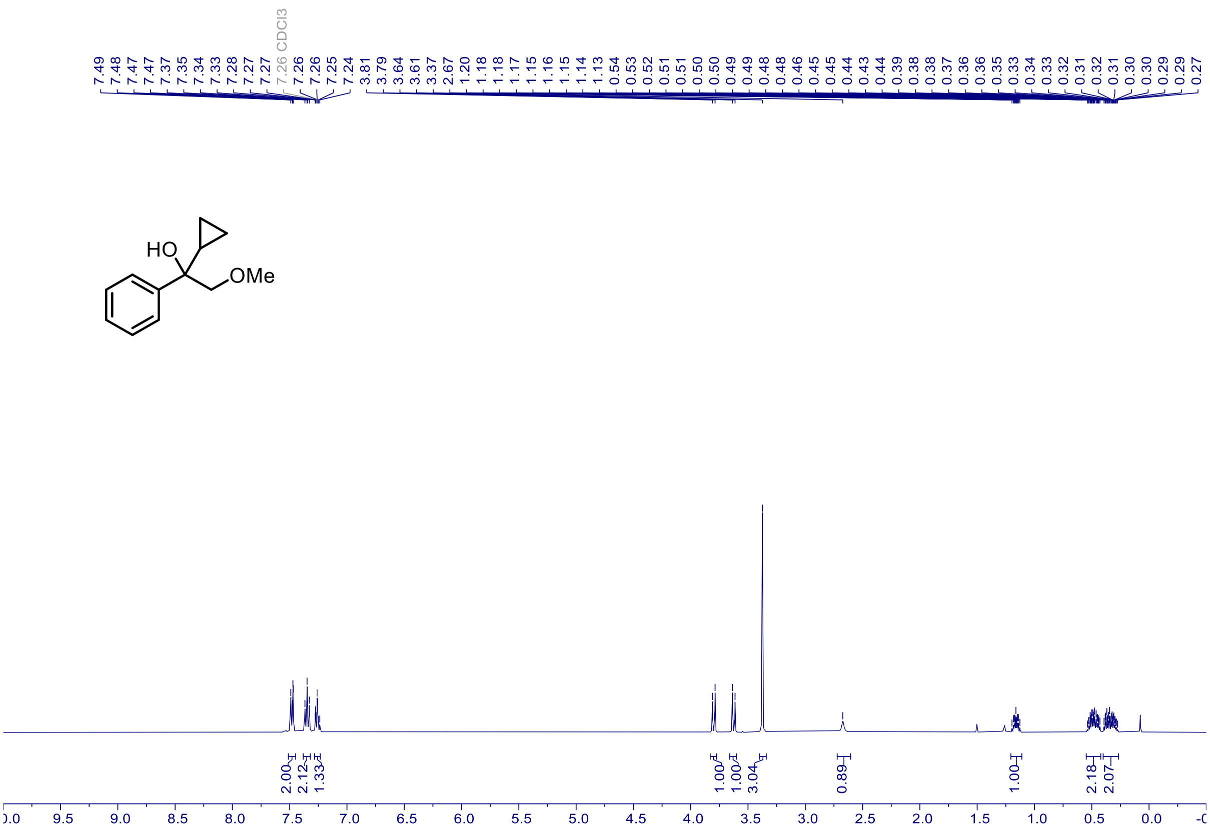

4bc – ^{13}C NMR (101 MHz, $CDCl_3$)

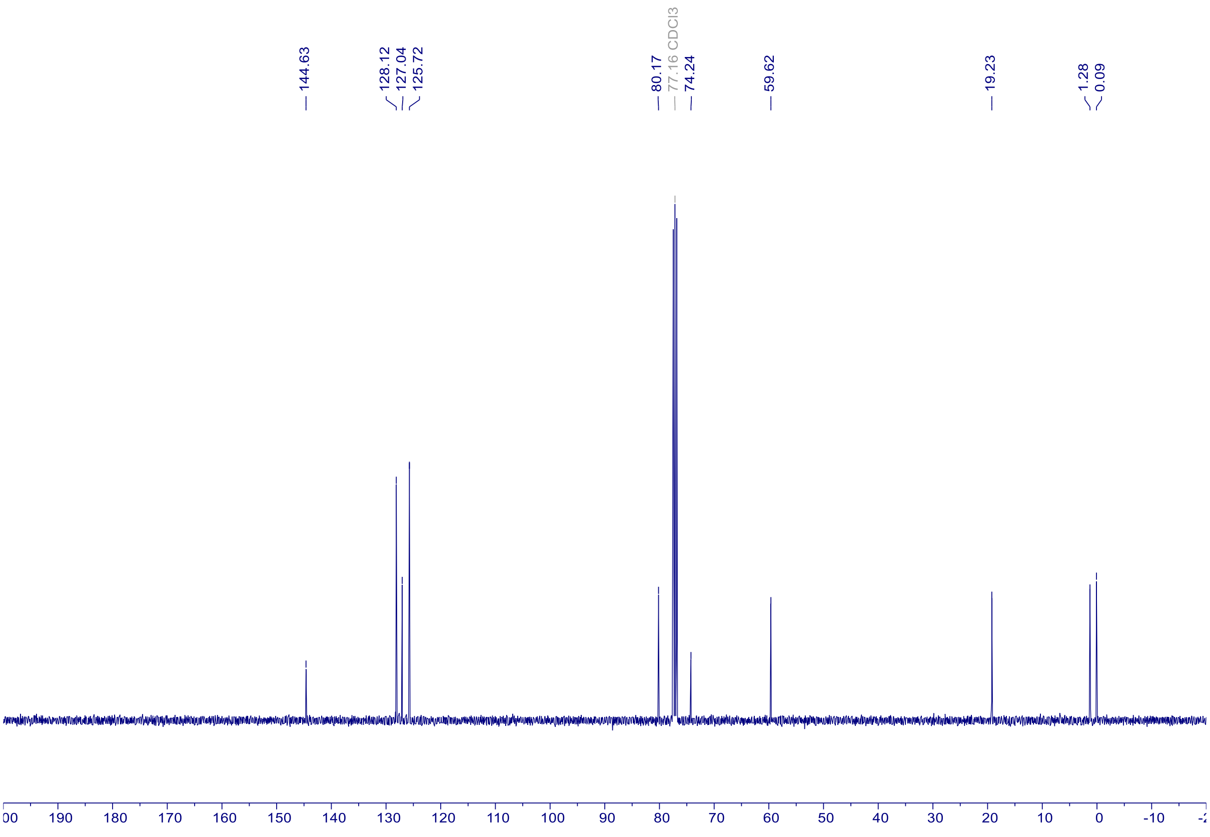

4bd – ^{1}H NMR (400 MHz, $CDCl_3$, 6.6:1 mixture of diastereoisomers)

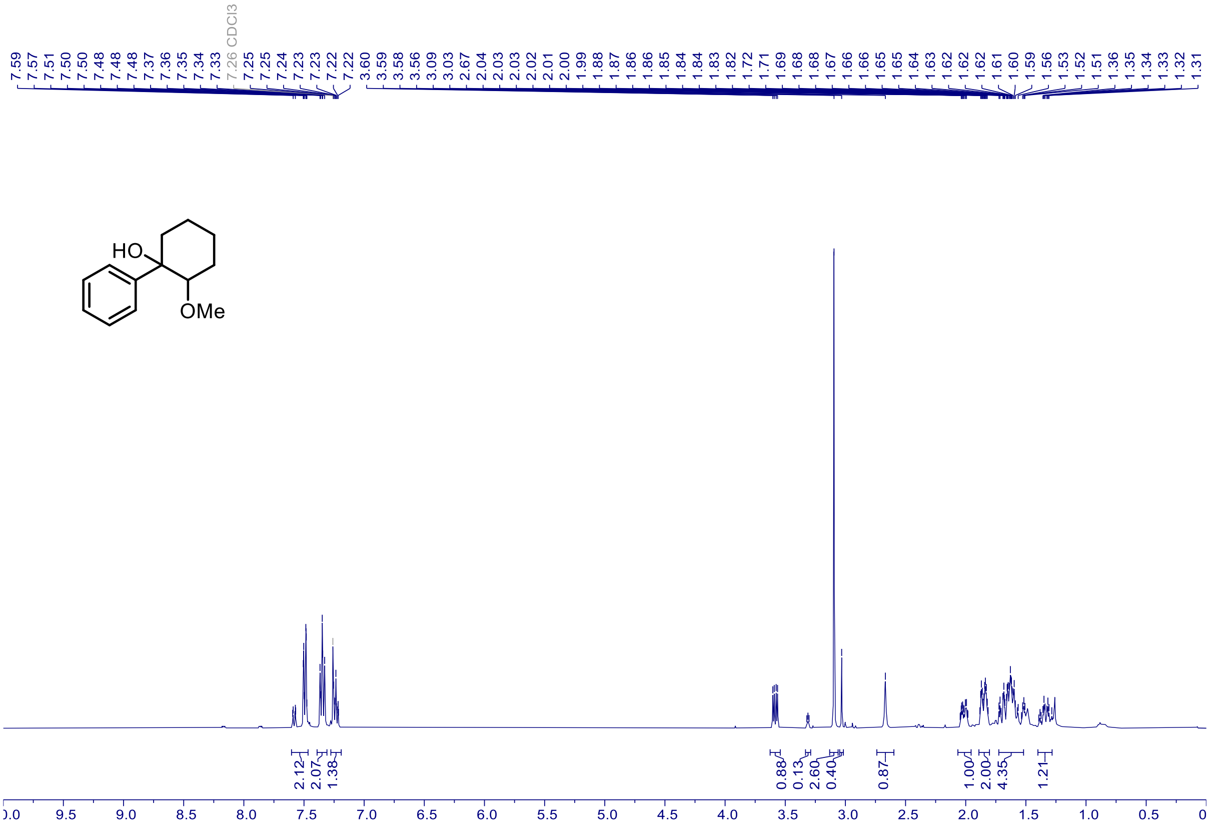

4bd – ^{13}C NMR (101 MHz, $CDCl_3$, 6.6:1 mixture of diastereoisomers)

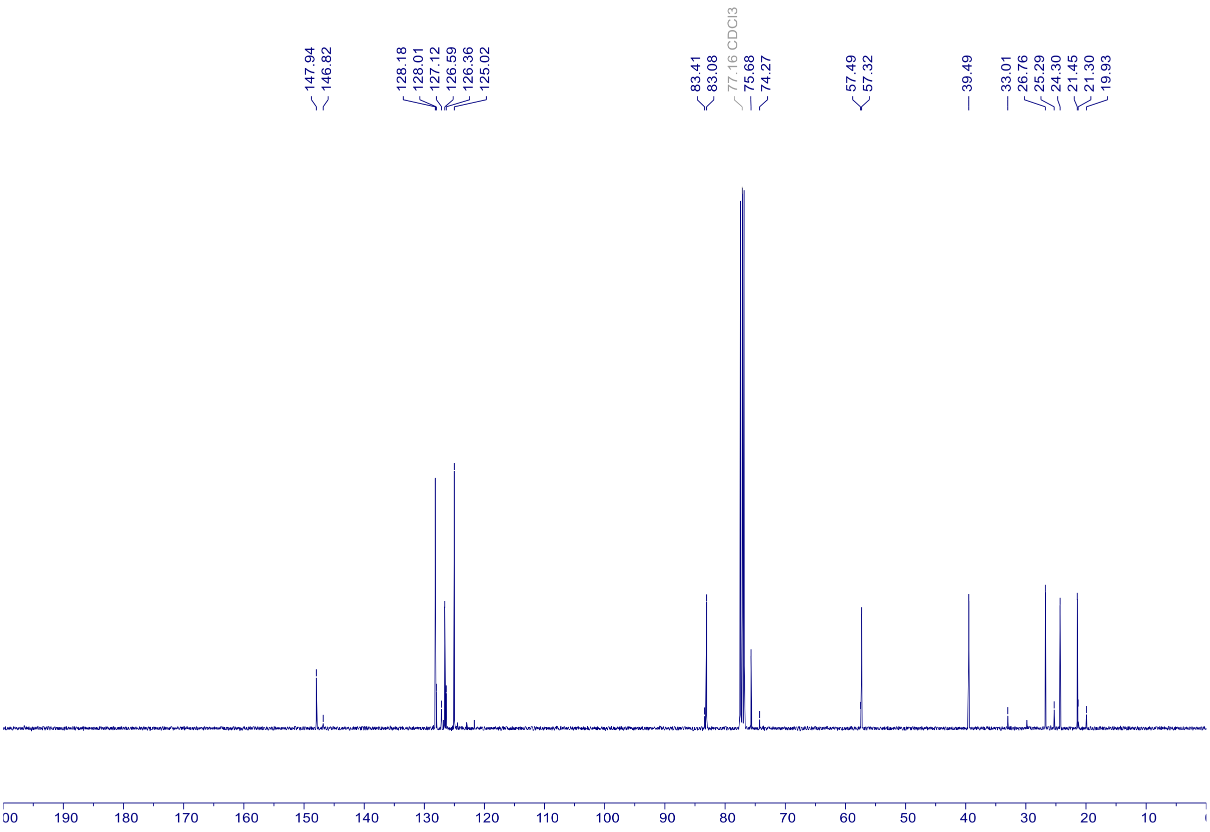

4be – ^{1}H NMR (500 MHz, $CDCl_3$, mixture of rotamers)

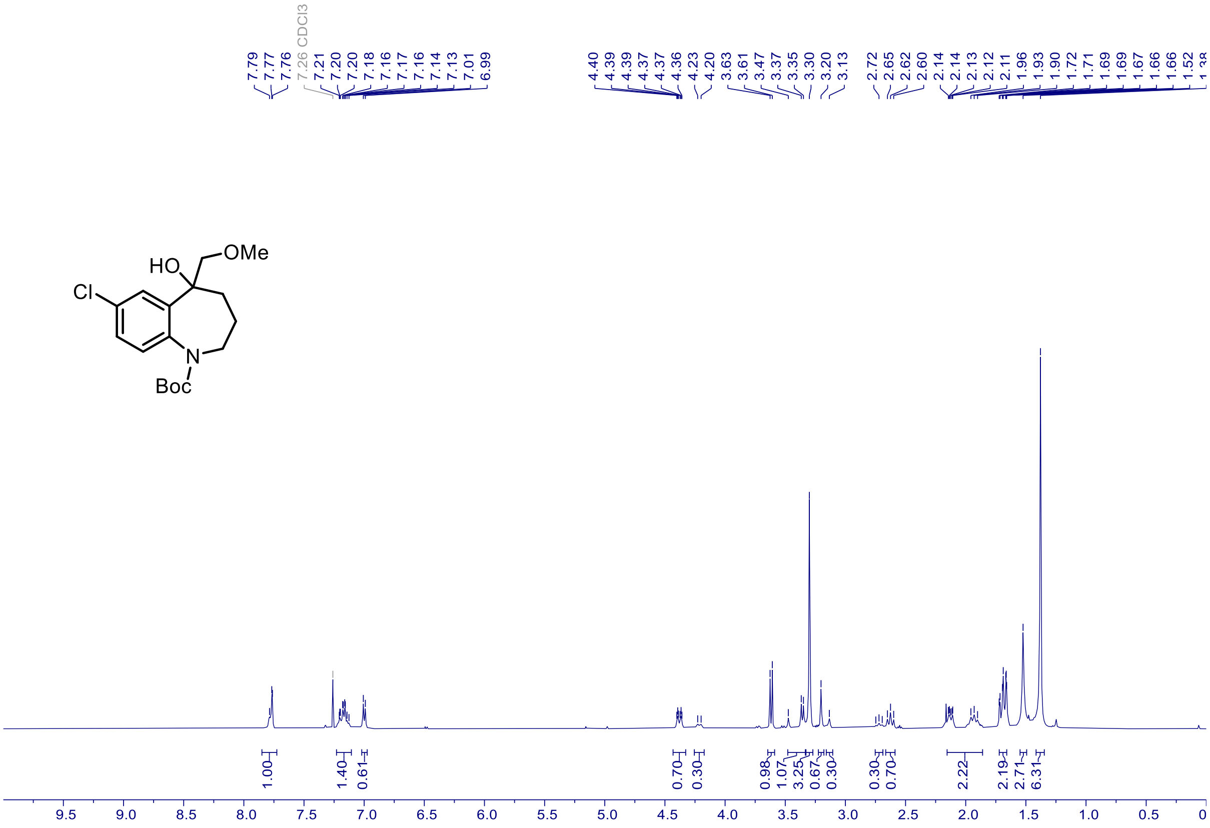

4be – ^{13}C NMR (126 MHz, $CDCl_3$, mixture of rotamers)

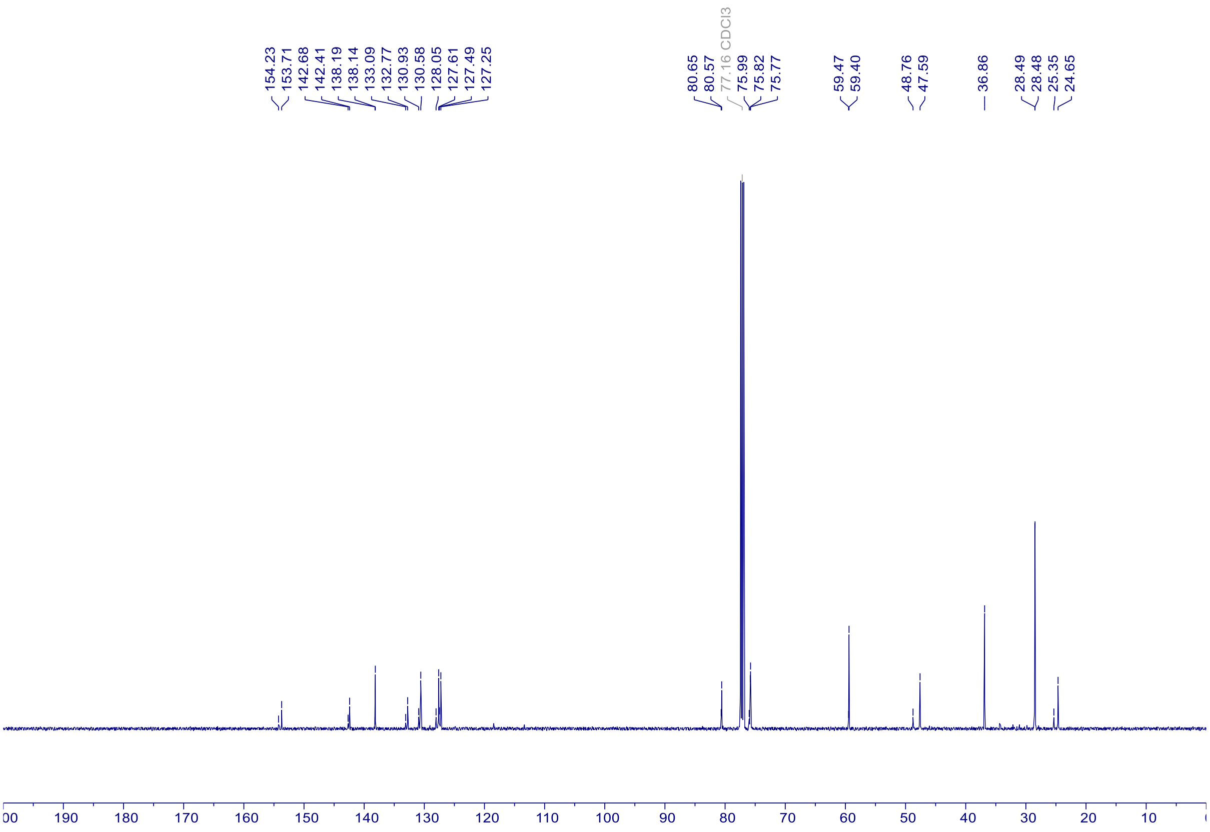

4bf – ^{1}H NMR (400 MHz, $CDCl_3$)

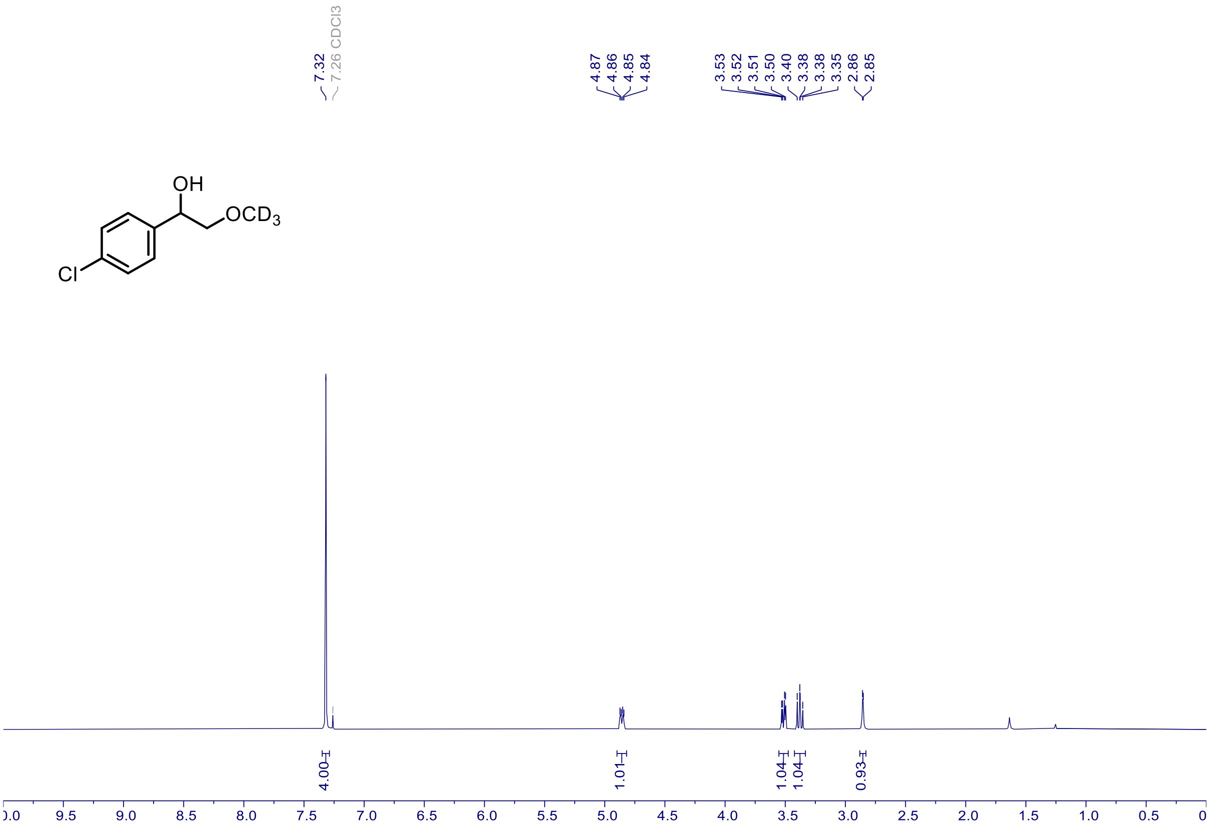

4bf – ^{13}C NMR (101 MHz, $CDCl_3$)

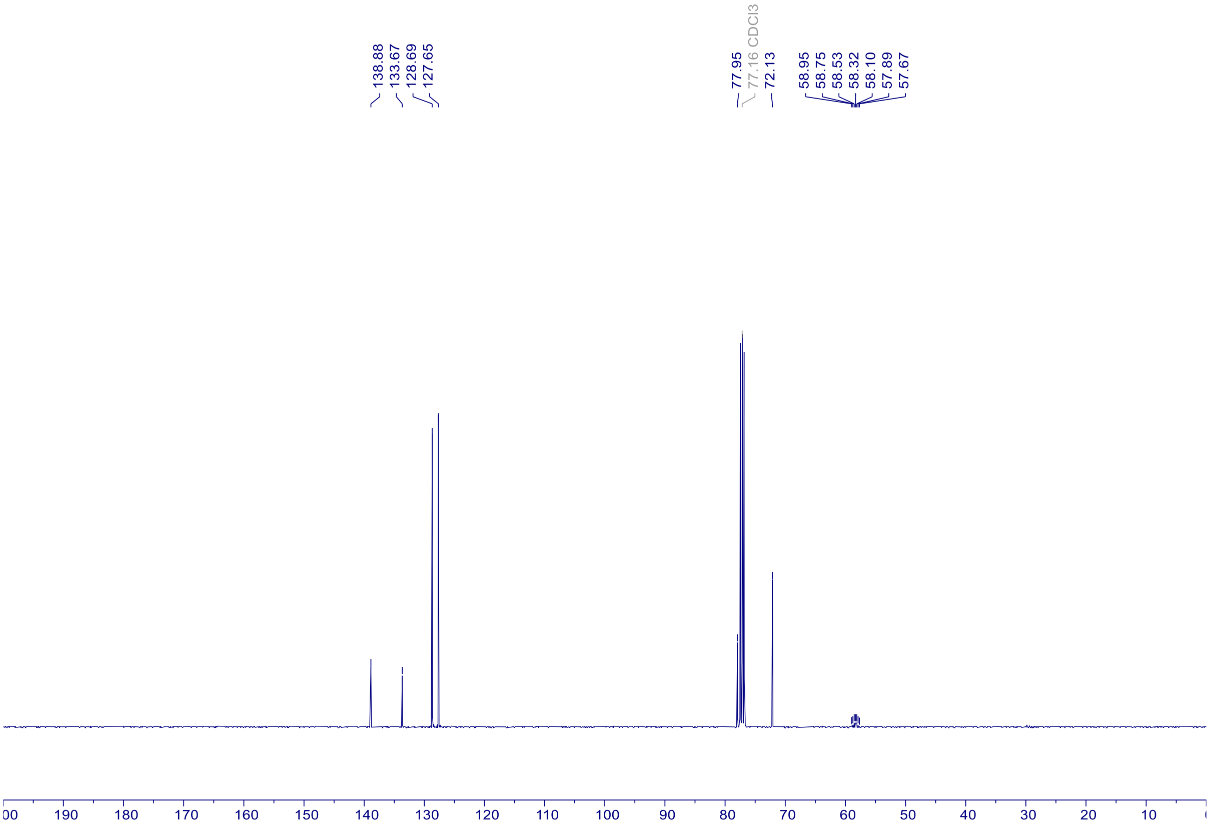

4bf – ^{2}H NMR (61 MHz, $CDCl_3$)

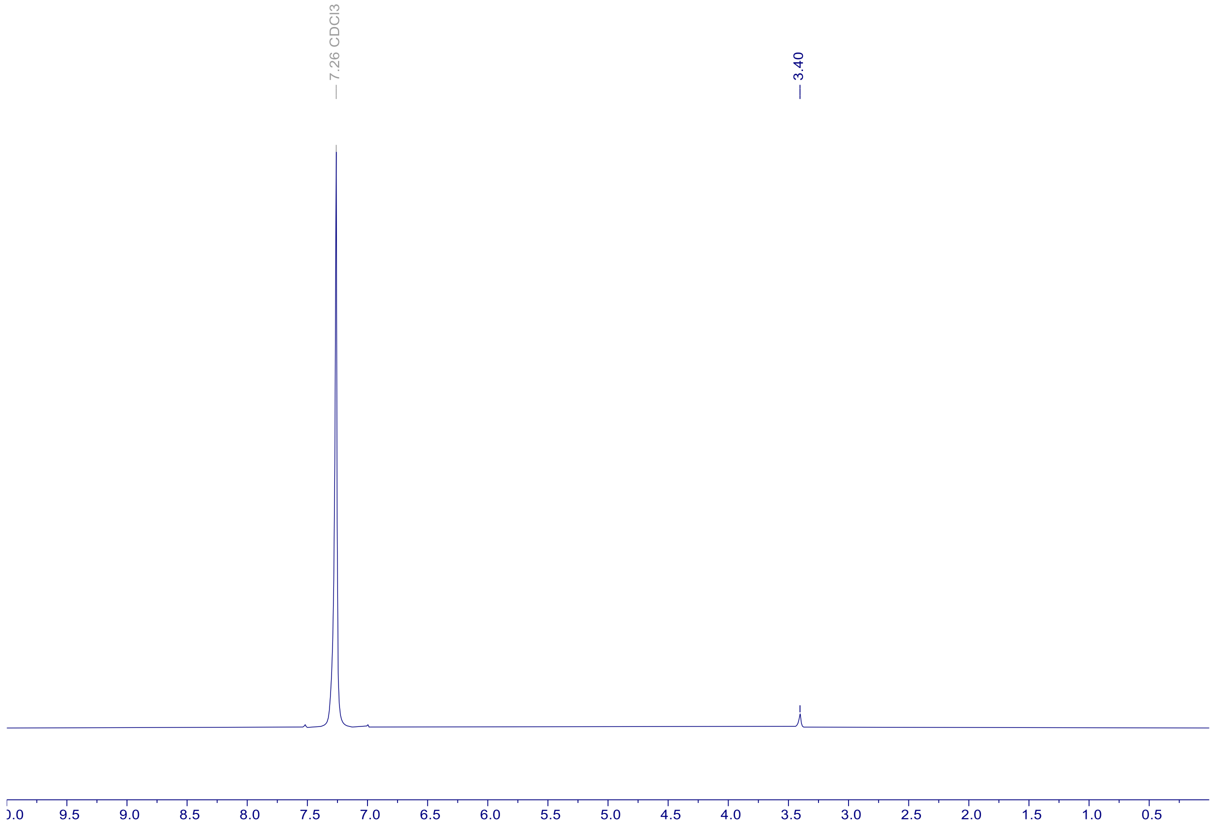

4bg – ^{1}H NMR (500 MHz, $CDCl_3$)

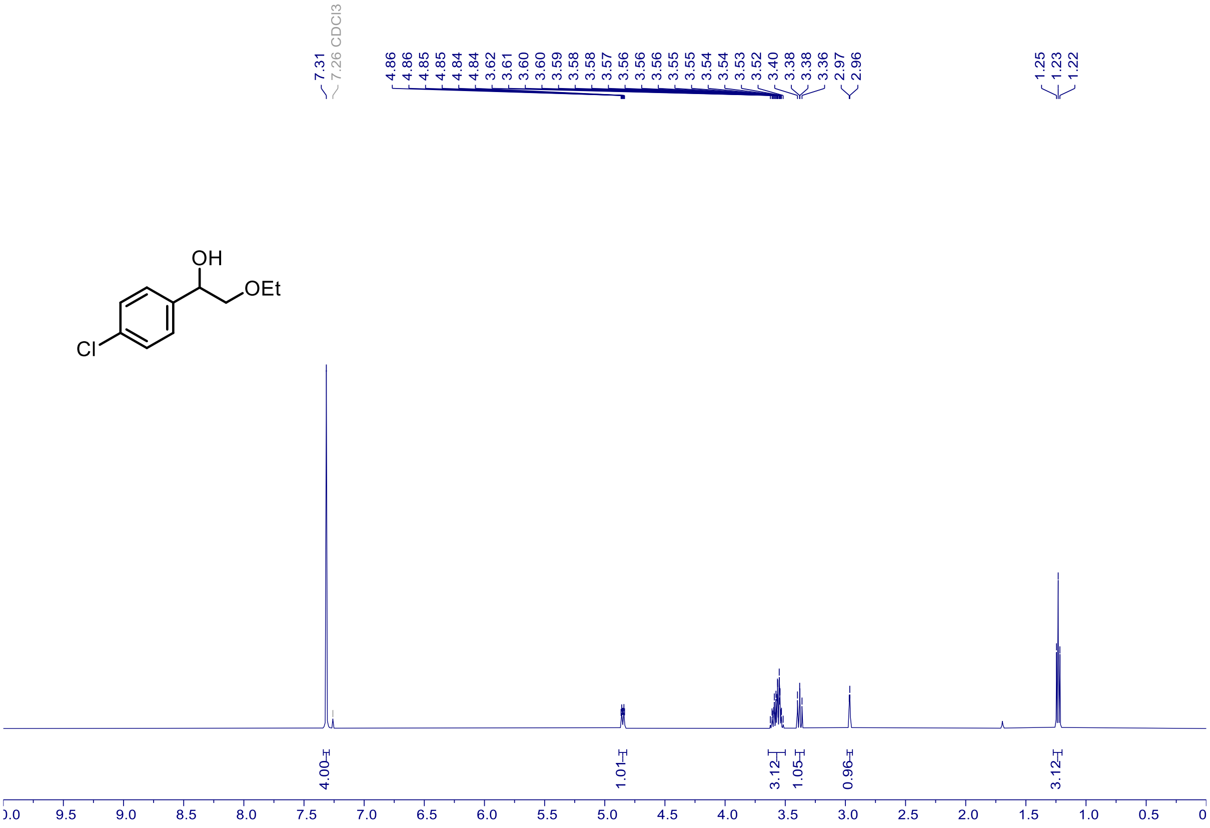

4bg – ^{13}C NMR (126 MHz, $CDCl_3$)

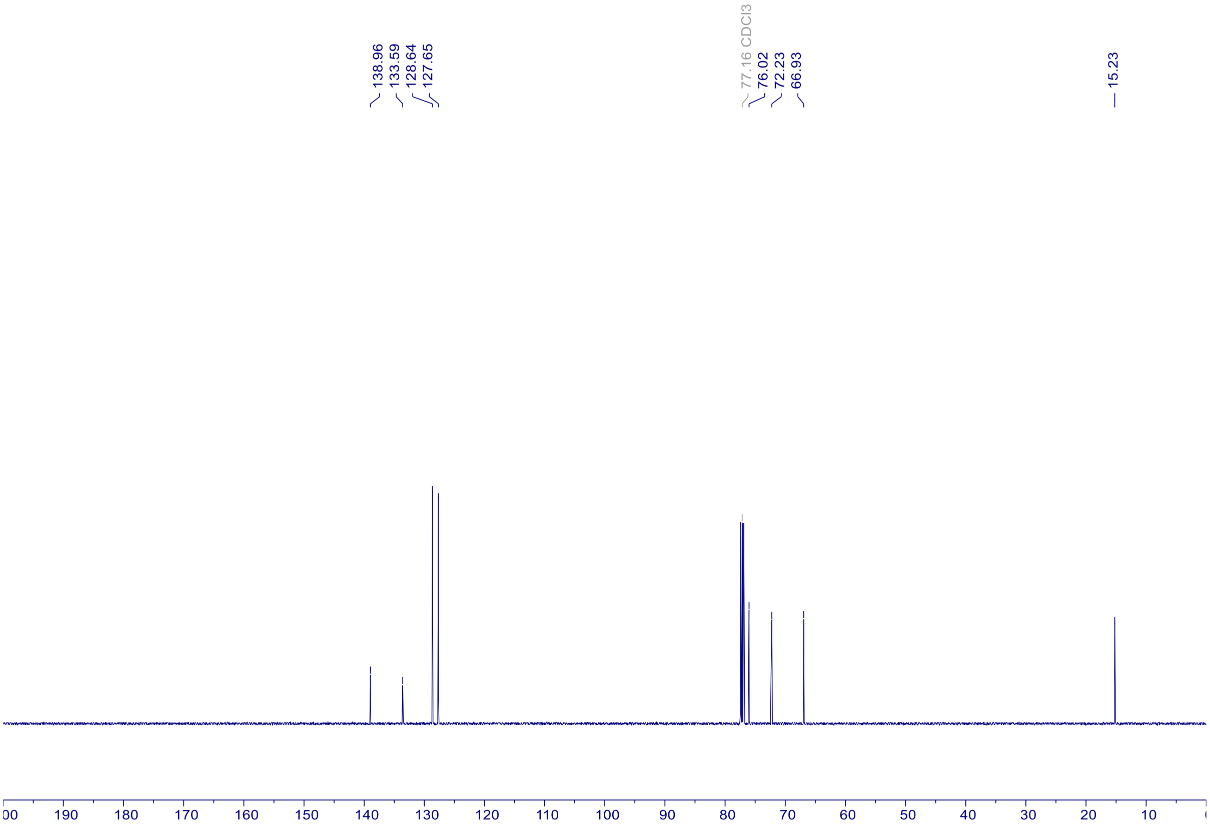

4bh – ^{1}H NMR (400 MHz, $CDCl_3$)

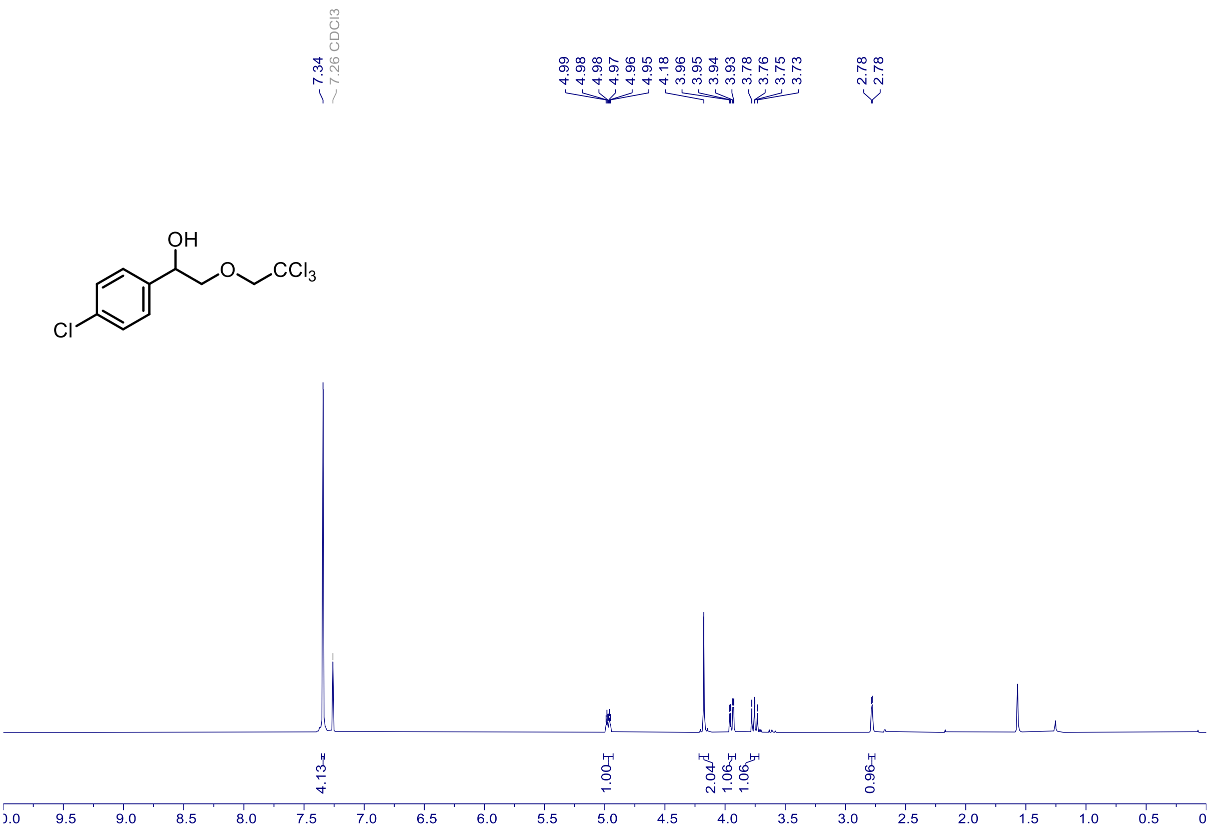

4bh – ^{13}C NMR (101 MHz, $CDCl_3$)

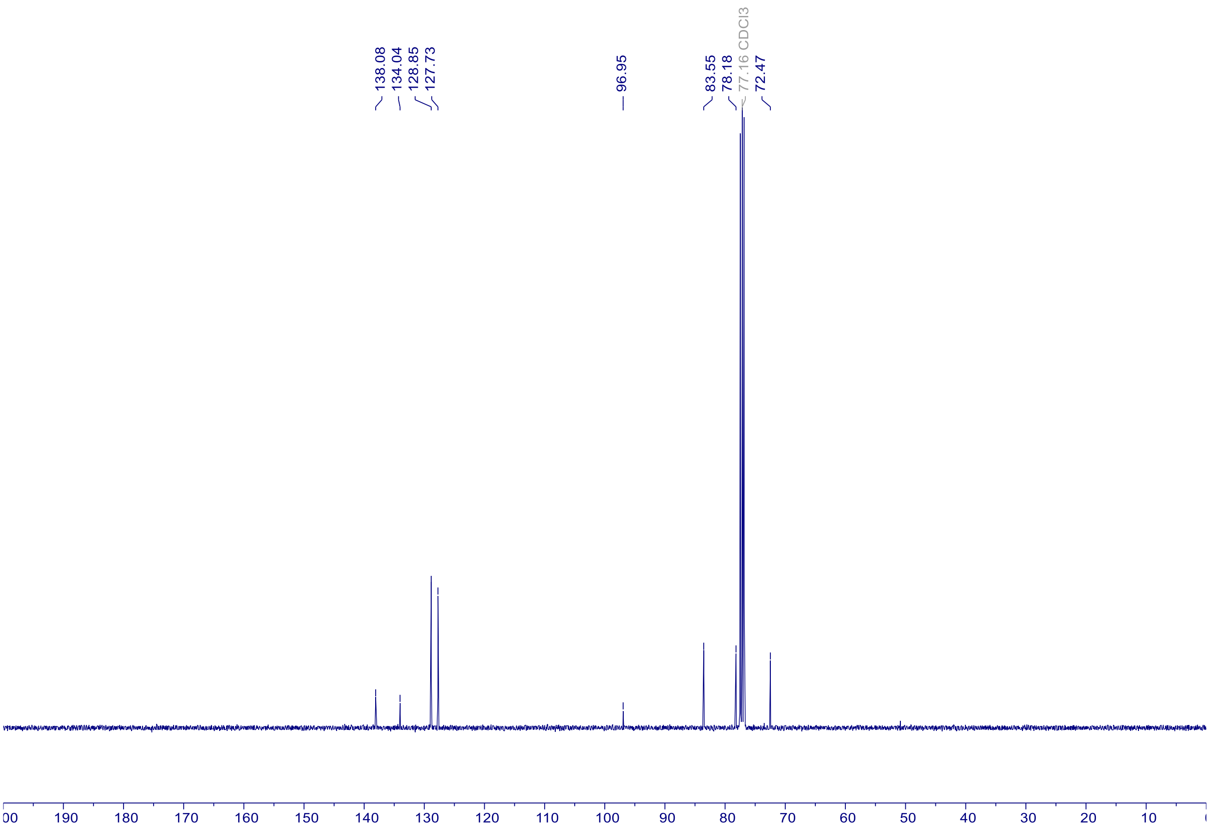

4bi – ^{1}H NMR (400 MHz, $CDCl_3$)

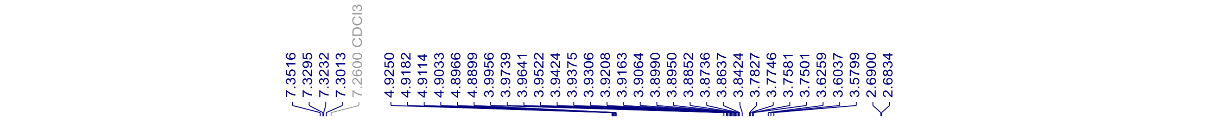

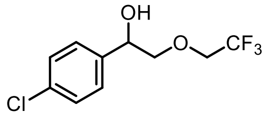

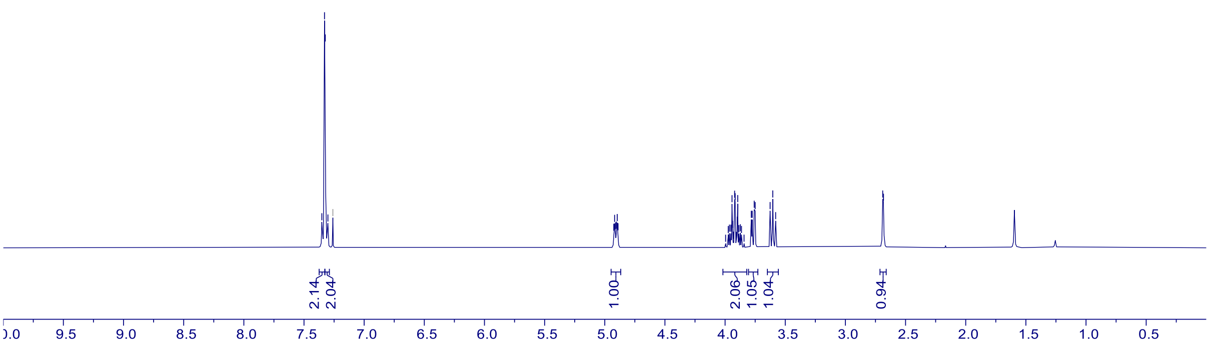

4bi – ^{13}C NMR (101 MHz, $CDCl_3$)

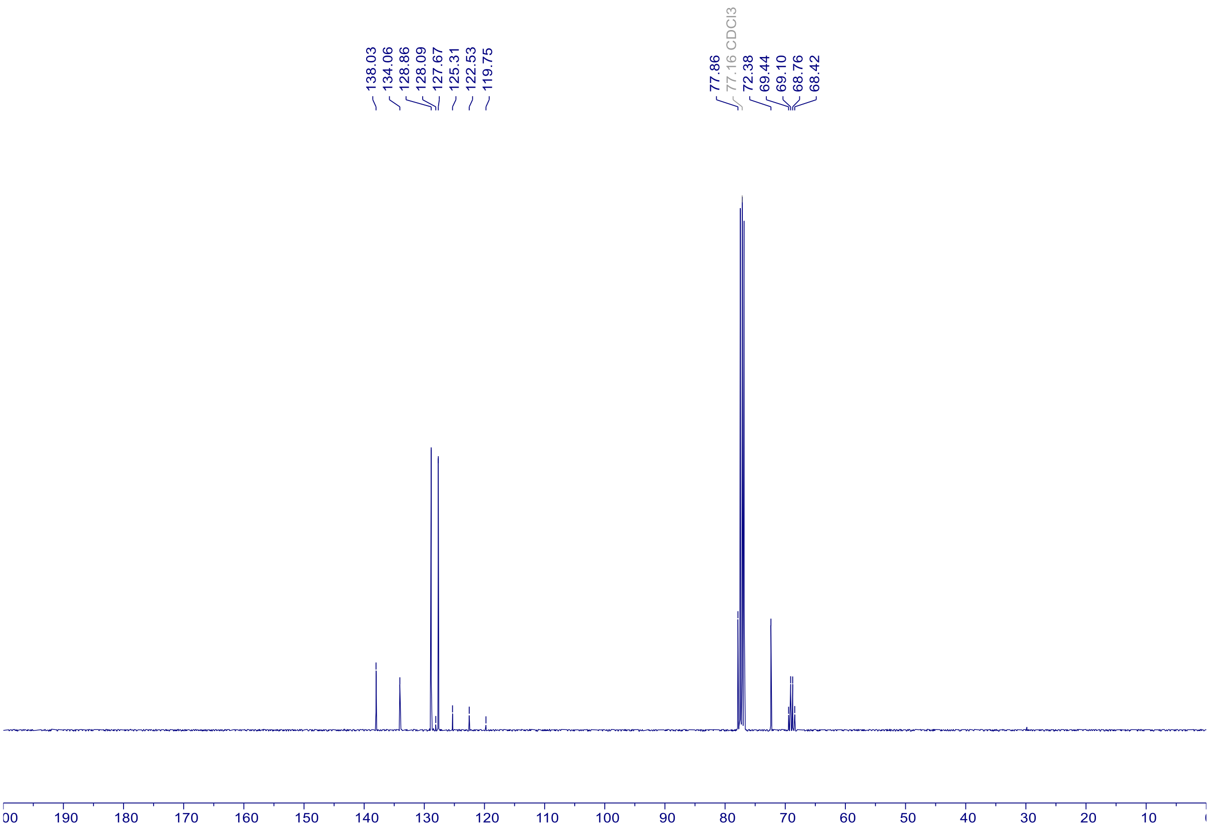

4bi – ^{19}F NMR (376 MHz, $CDCl_3$)

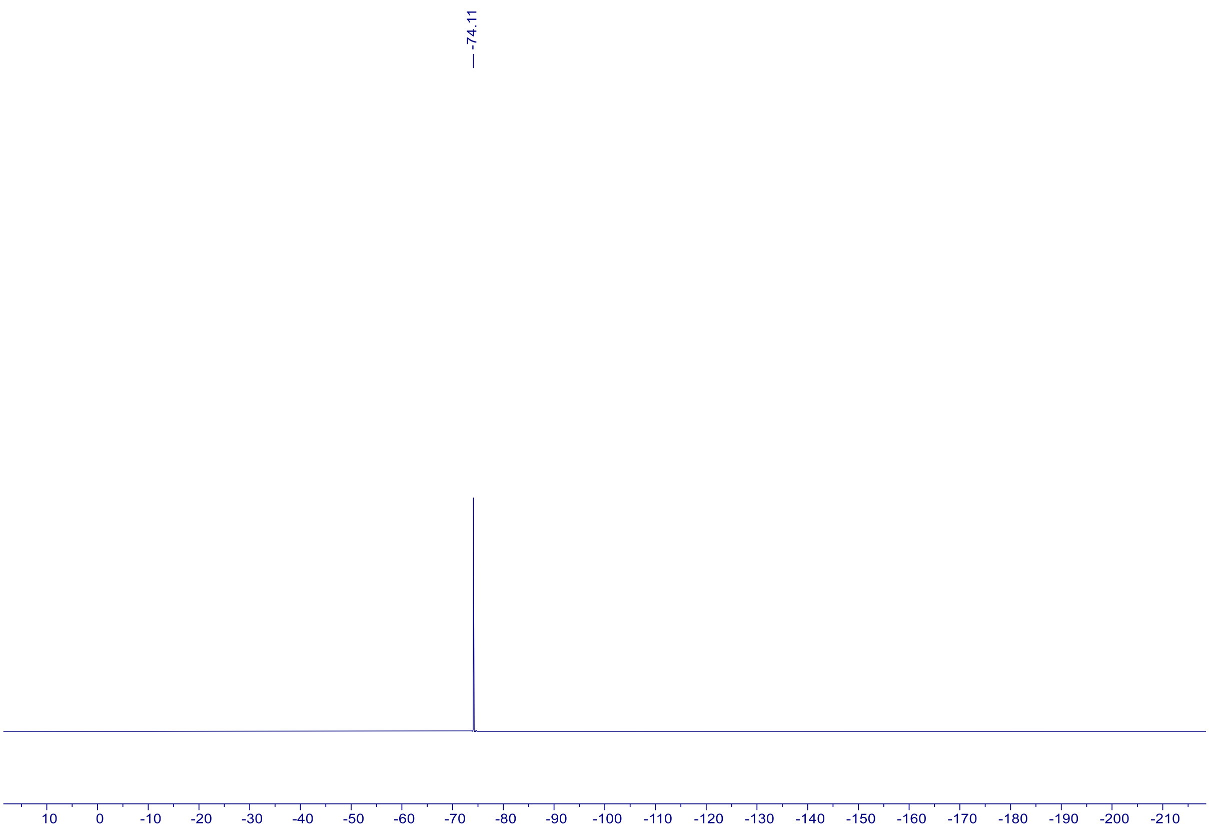

4bj – ^{1}H NMR (400 MHz, $CDCl_3$)

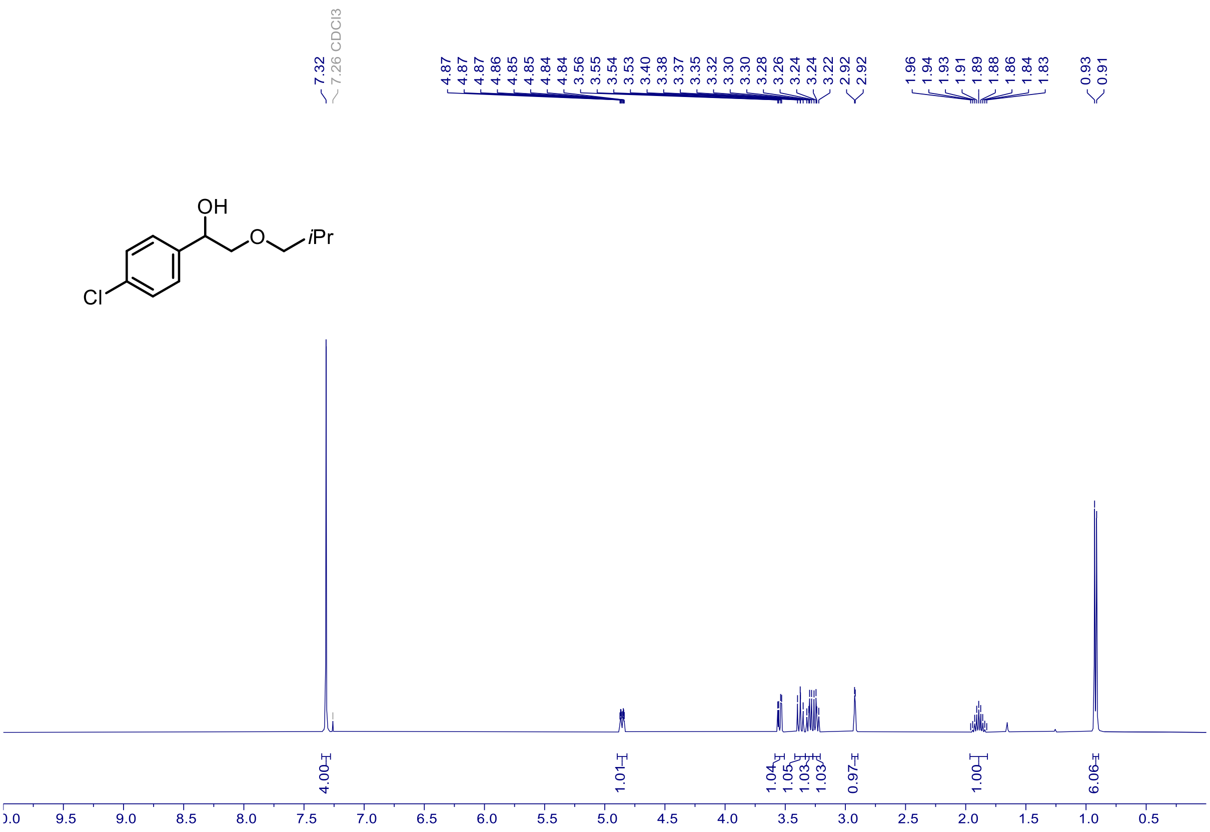

4bj – ^{13}C NMR (101 MHz, $CDCl_3$)

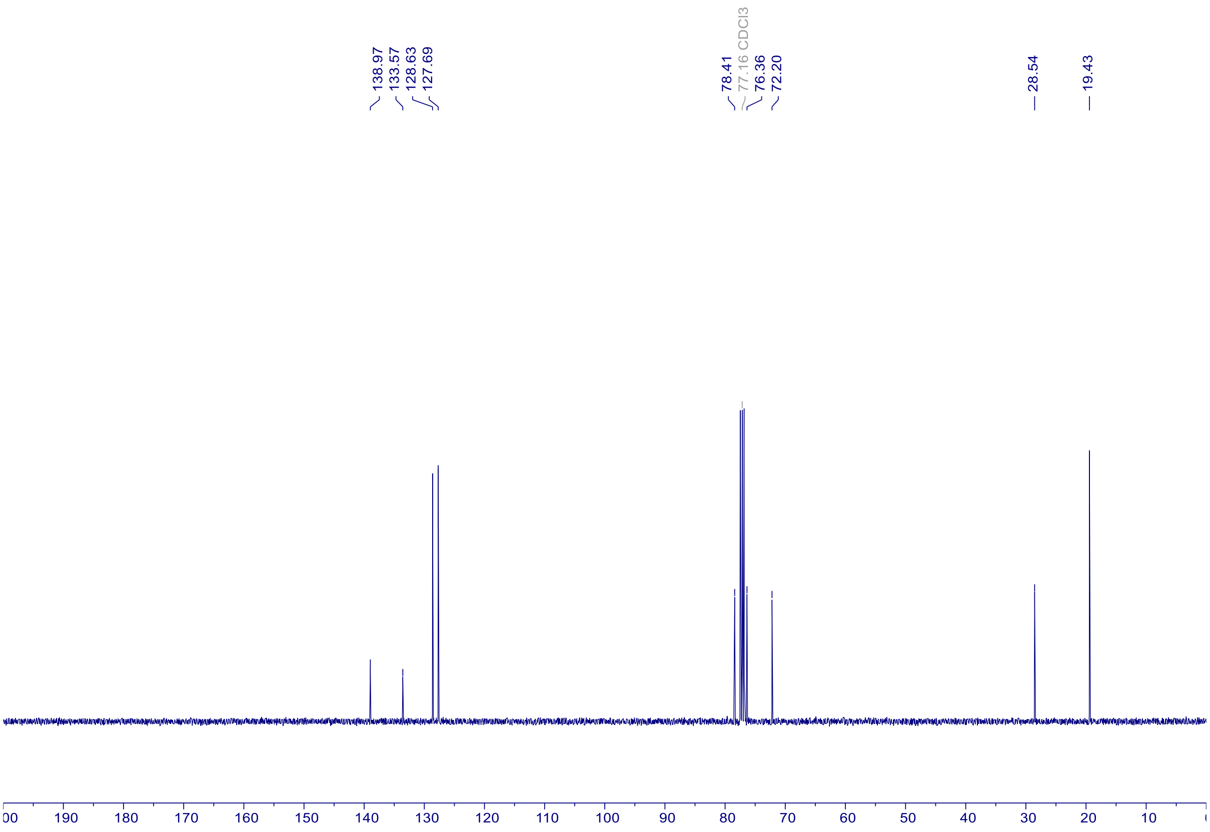

4bk – ^{1}H NMR (400 MHz, $CDCl_3$)

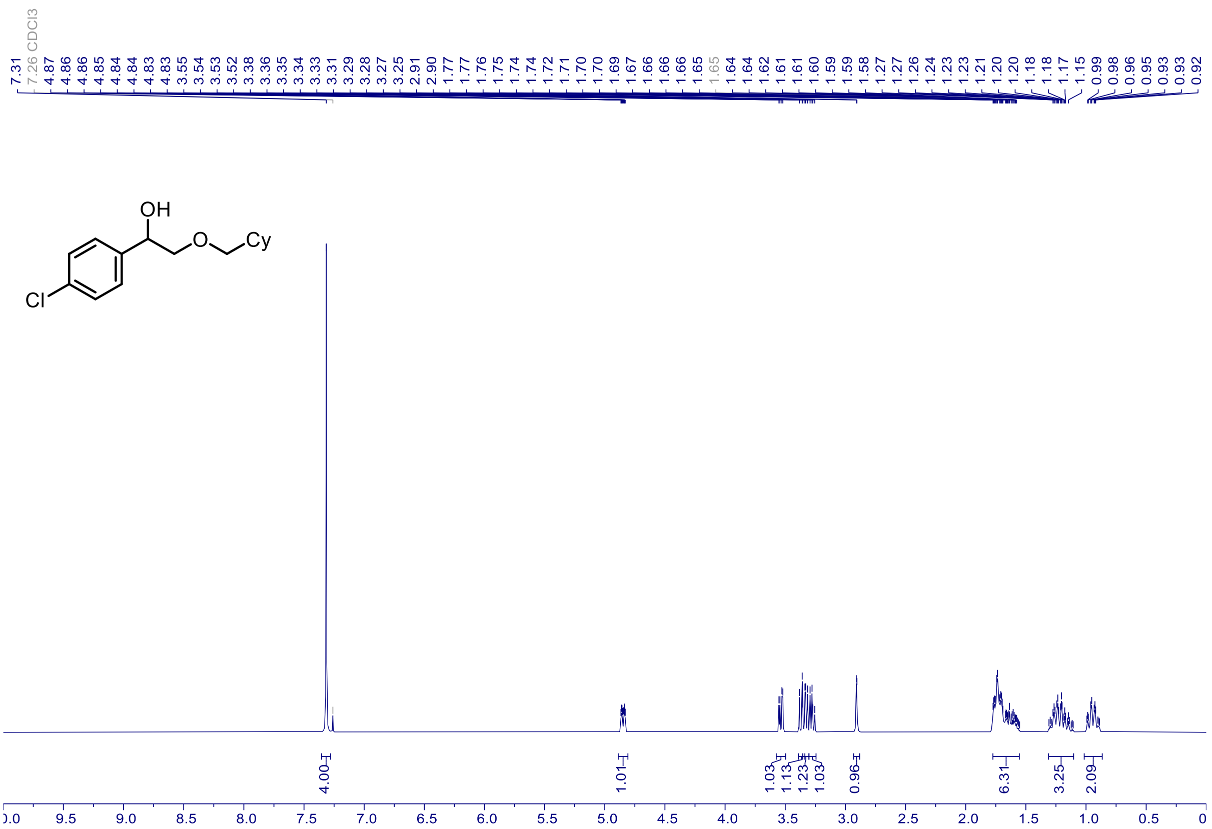

4bk – ^{13}C NMR (101 MHz, $CDCl_3$)

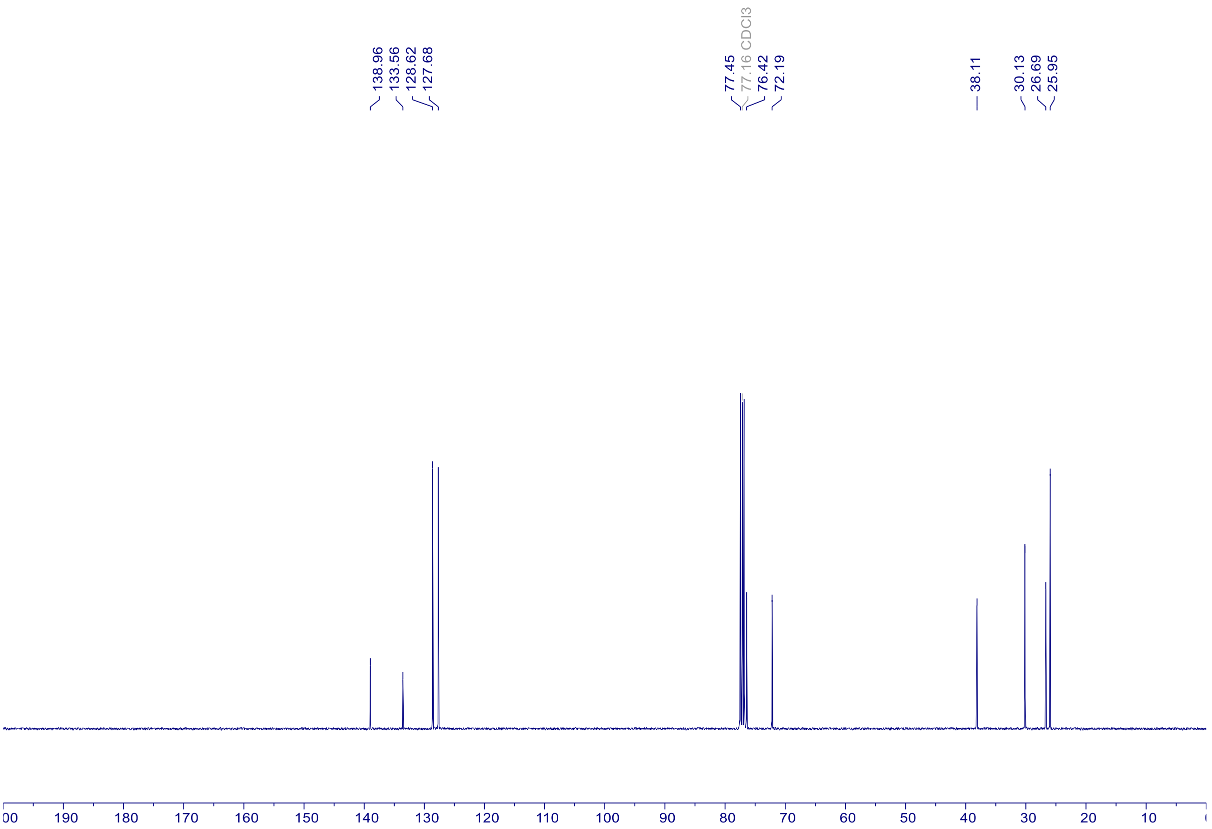

4bl – ^{1}H NMR (400 MHz, $CDCl_3$)

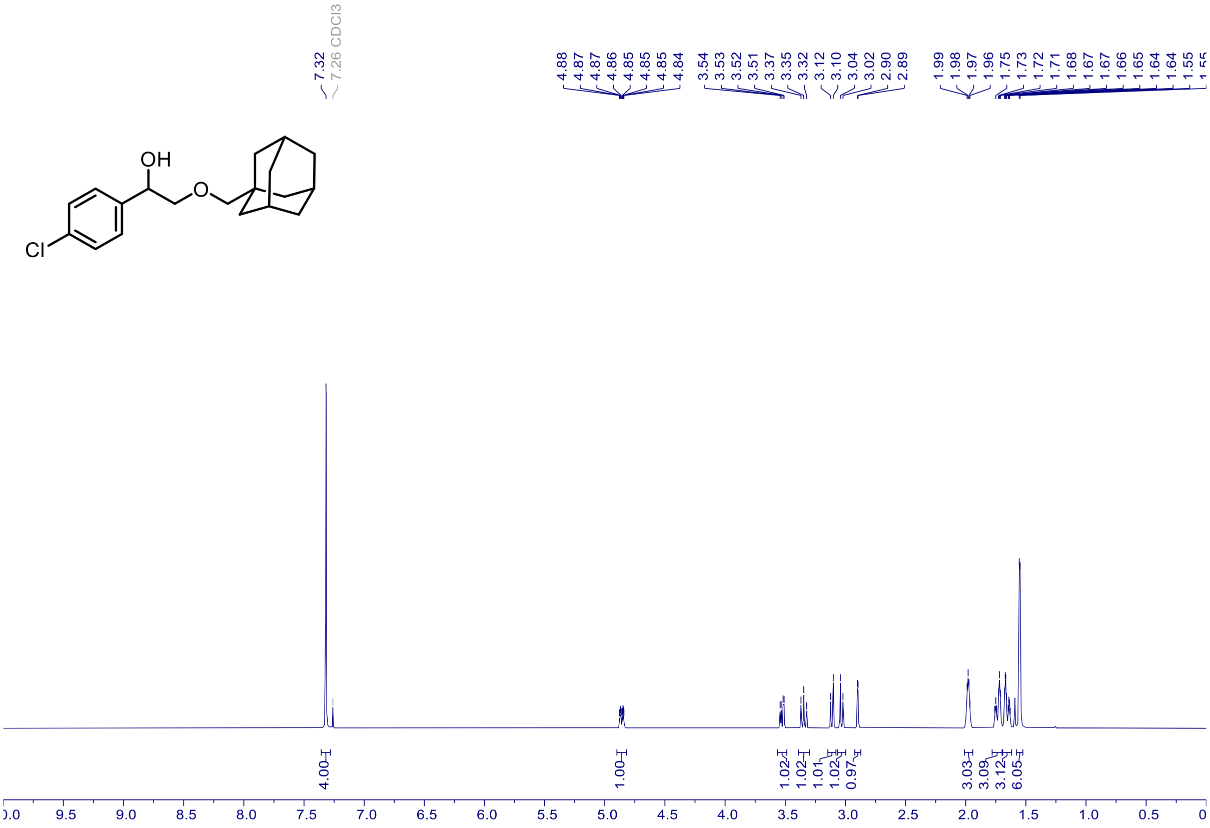

4bl – ^{13}C NMR (101 MHz, $CDCl_3$)

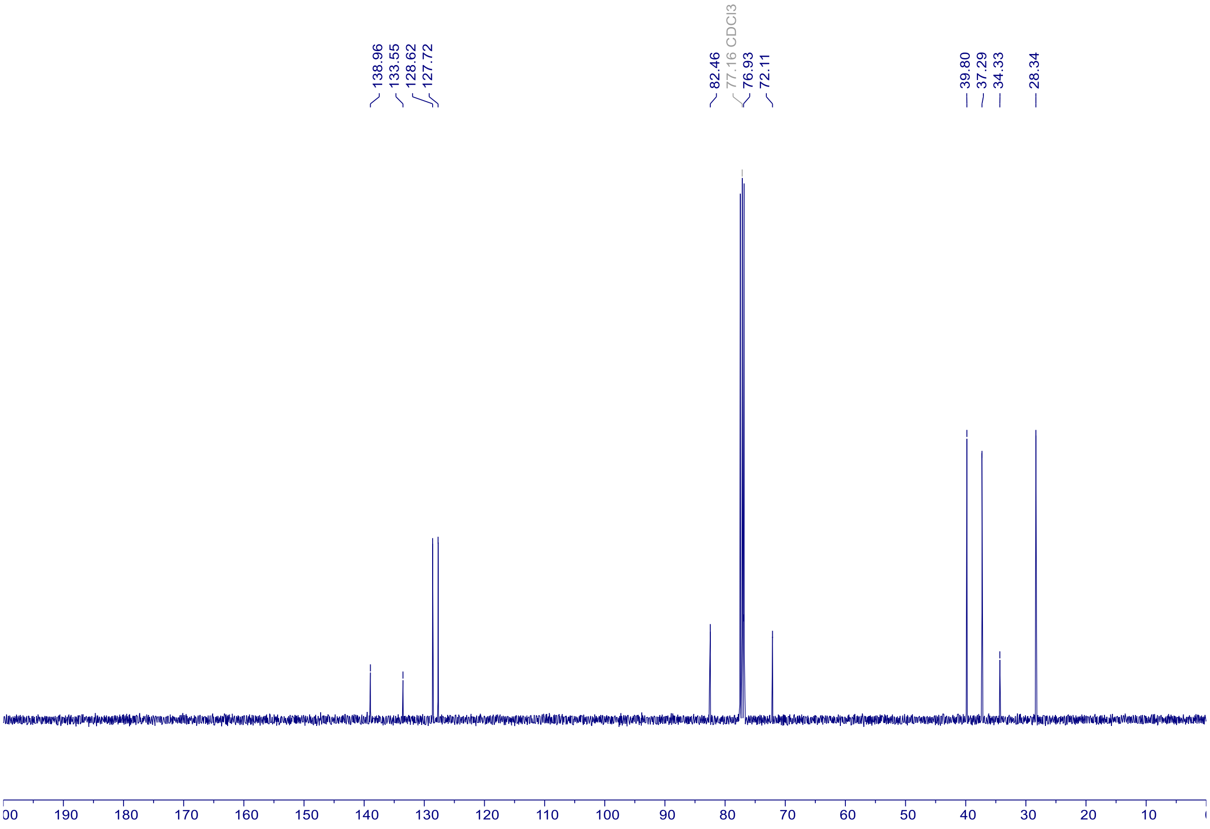

4bm – ^{1}H NMR (400 MHz, $CDCl_3$)

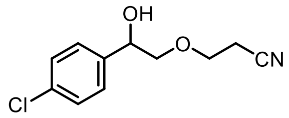

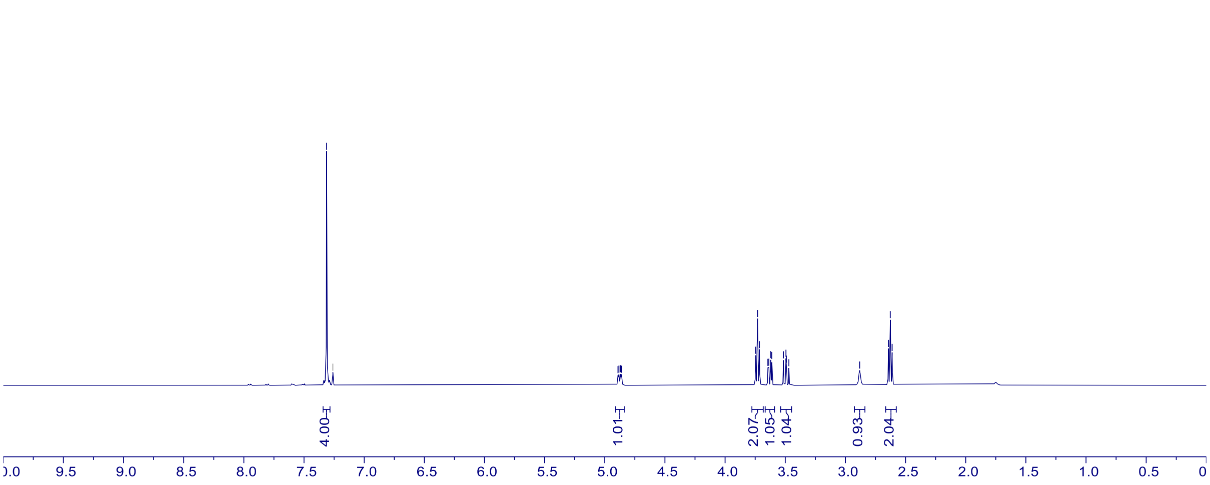

4bm – ^{13}C NMR (101 MHz, $CDCl_3$)

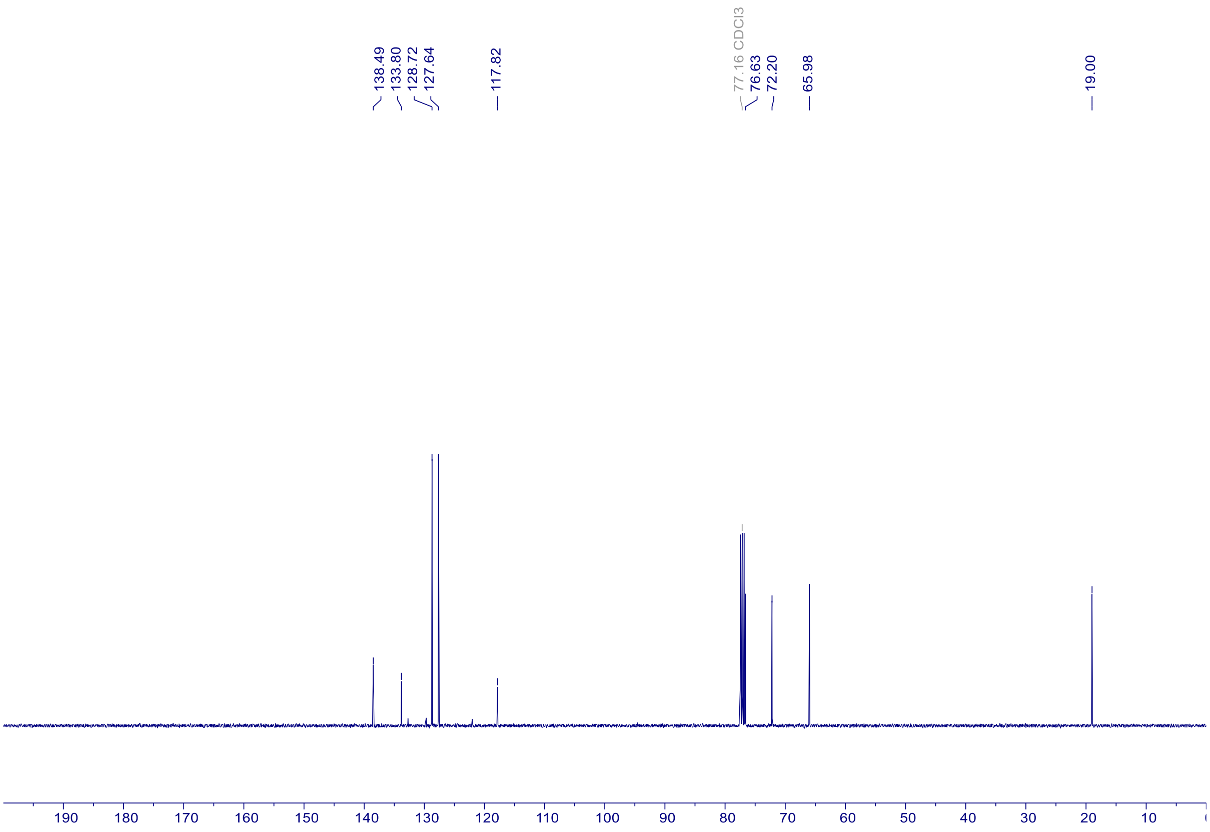

4bn – ^{1}H NMR (500 MHz, CDCl$_3$)

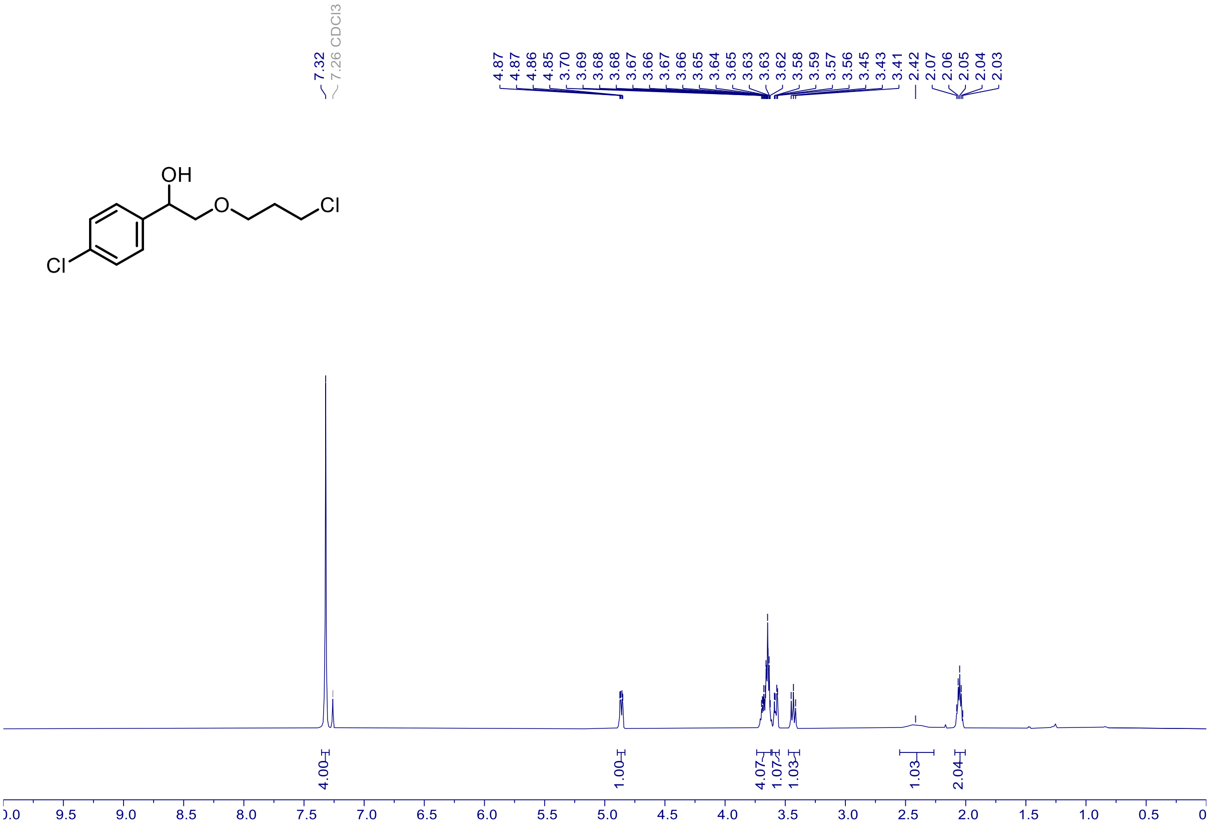

4bn – ^{13}C NMR (126 MHz, CDCl$_3$)

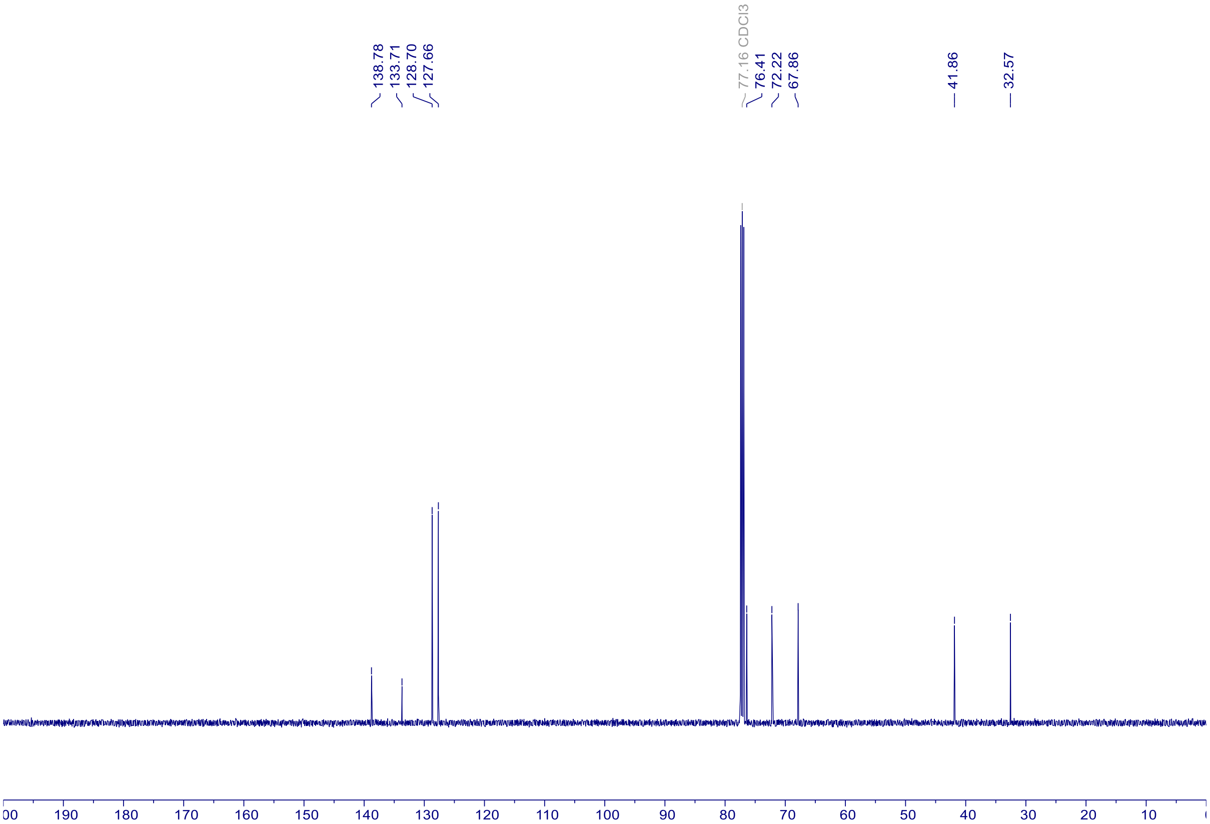

4bo – ^{1}H NMR (500 MHz, $CDCl_3$)

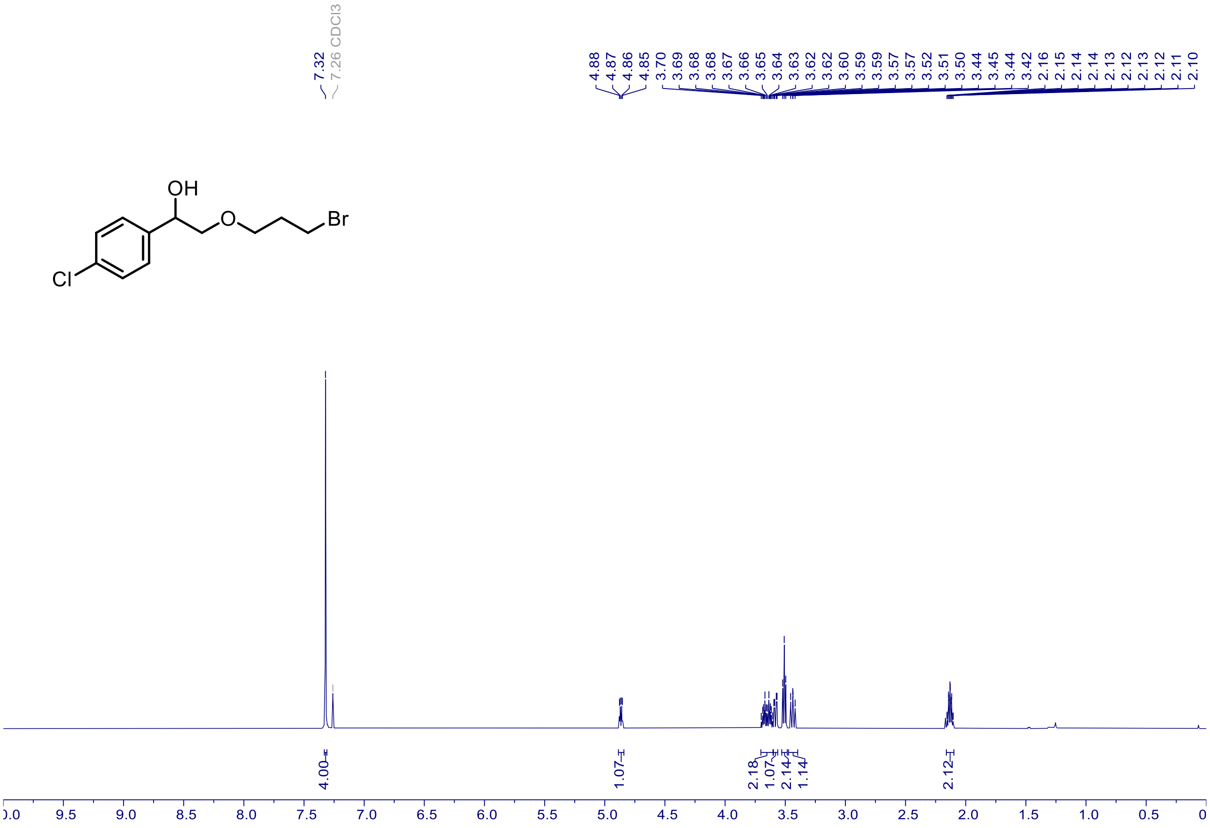

4bo – ^{13}C NMR (126 MHz, $CDCl_3$)

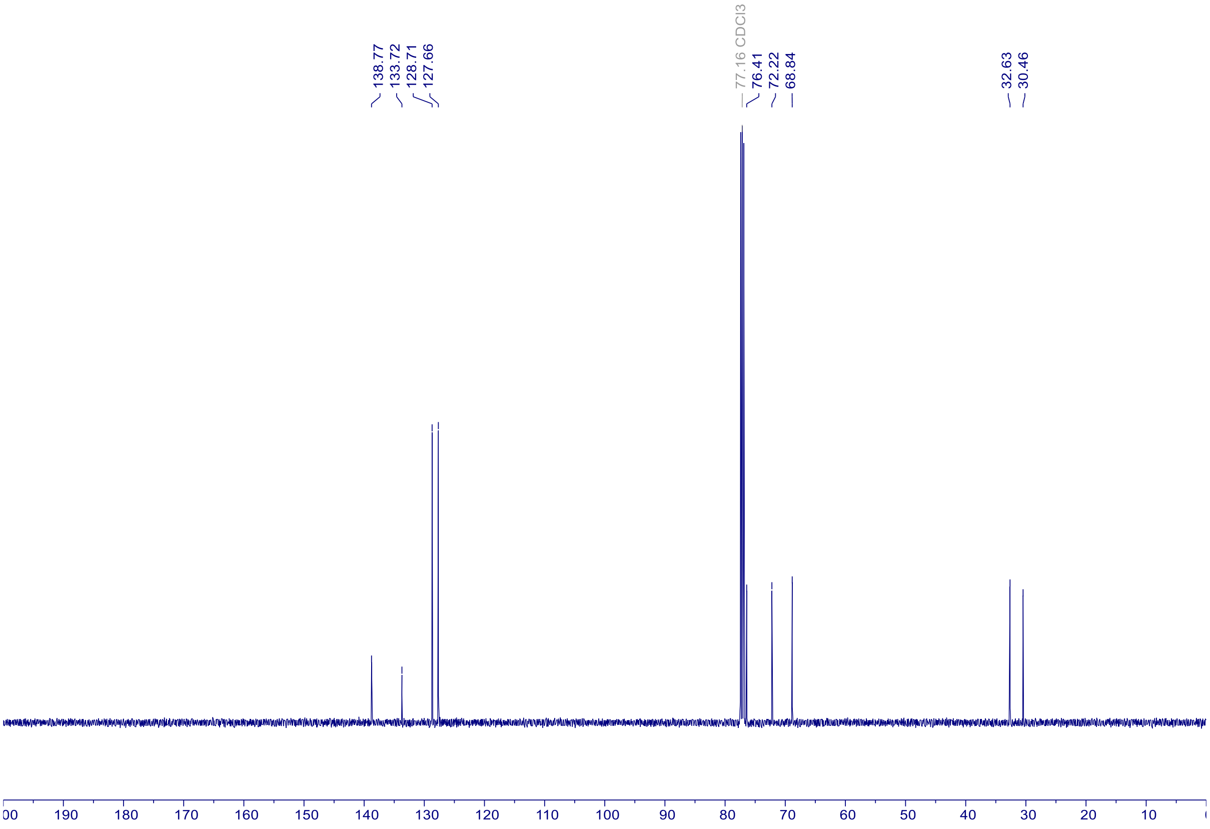

4bp – ¹H NMR (400 MHz, CDCl$_3$)

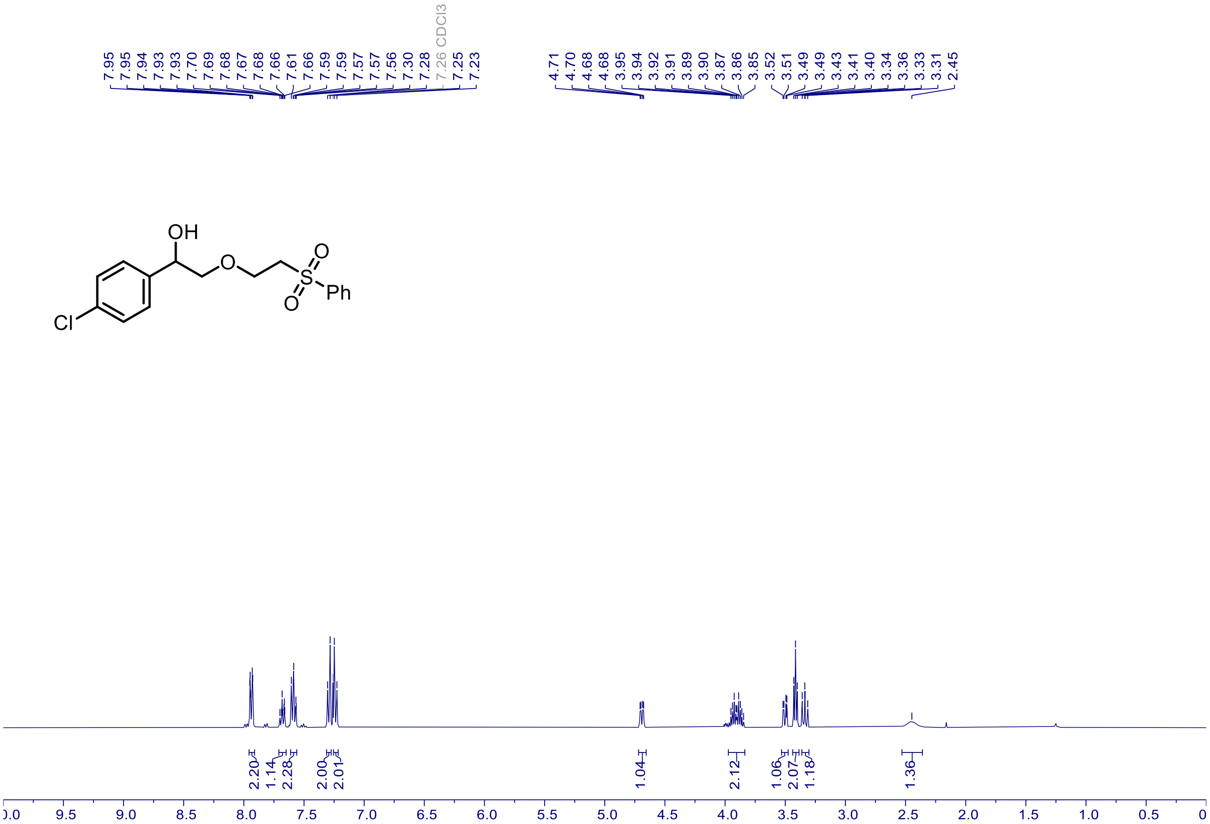

4bp – ¹³C NMR (101 MHz, CDCl$_3$)

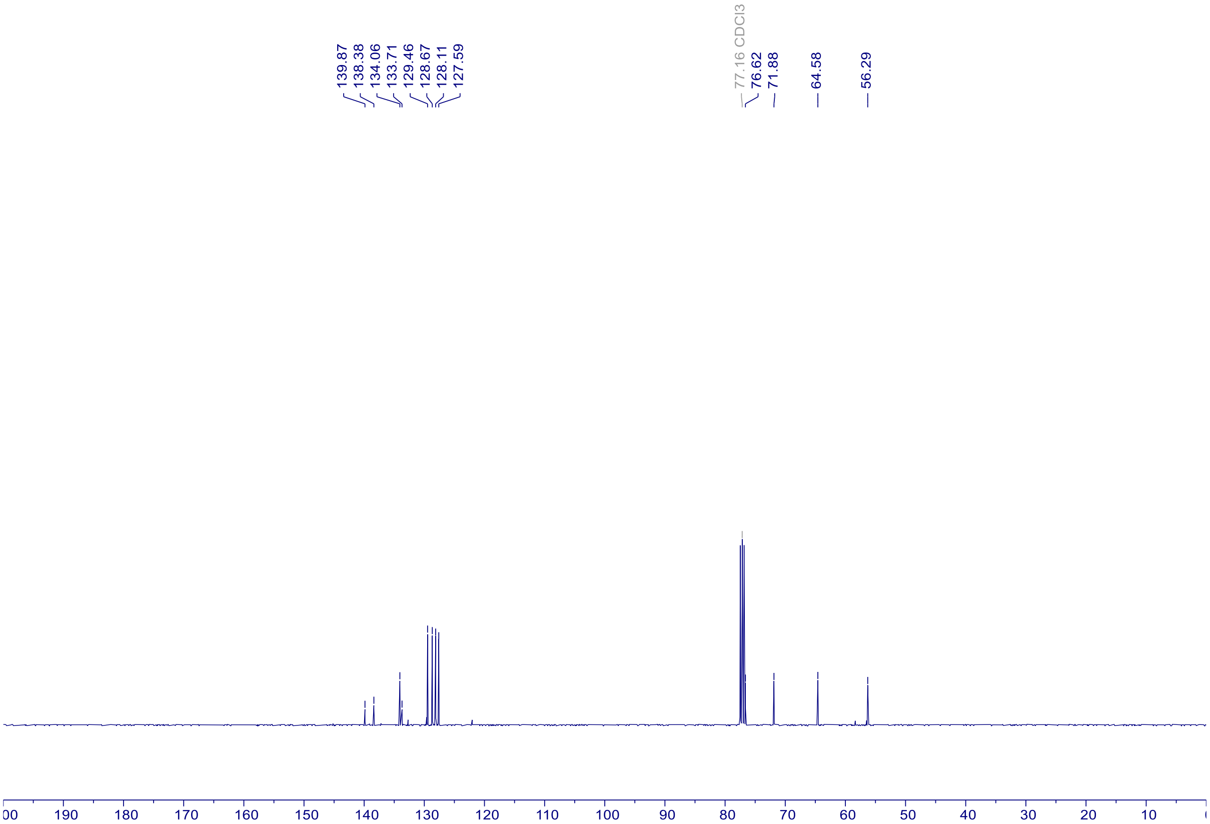

4bq – ^{1}H NMR (400 MHz, CDCl$_3$)

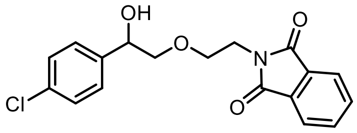

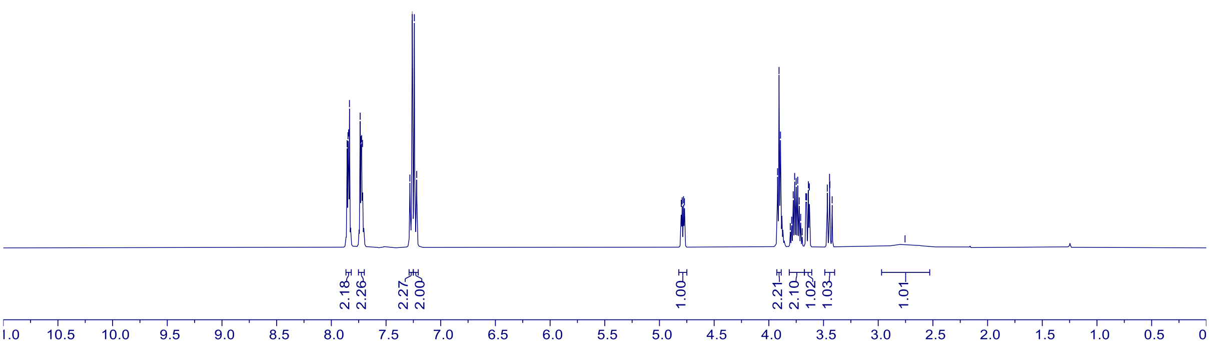

4bq – ^{13}C NMR (101 MHz, CDCl$_3$)

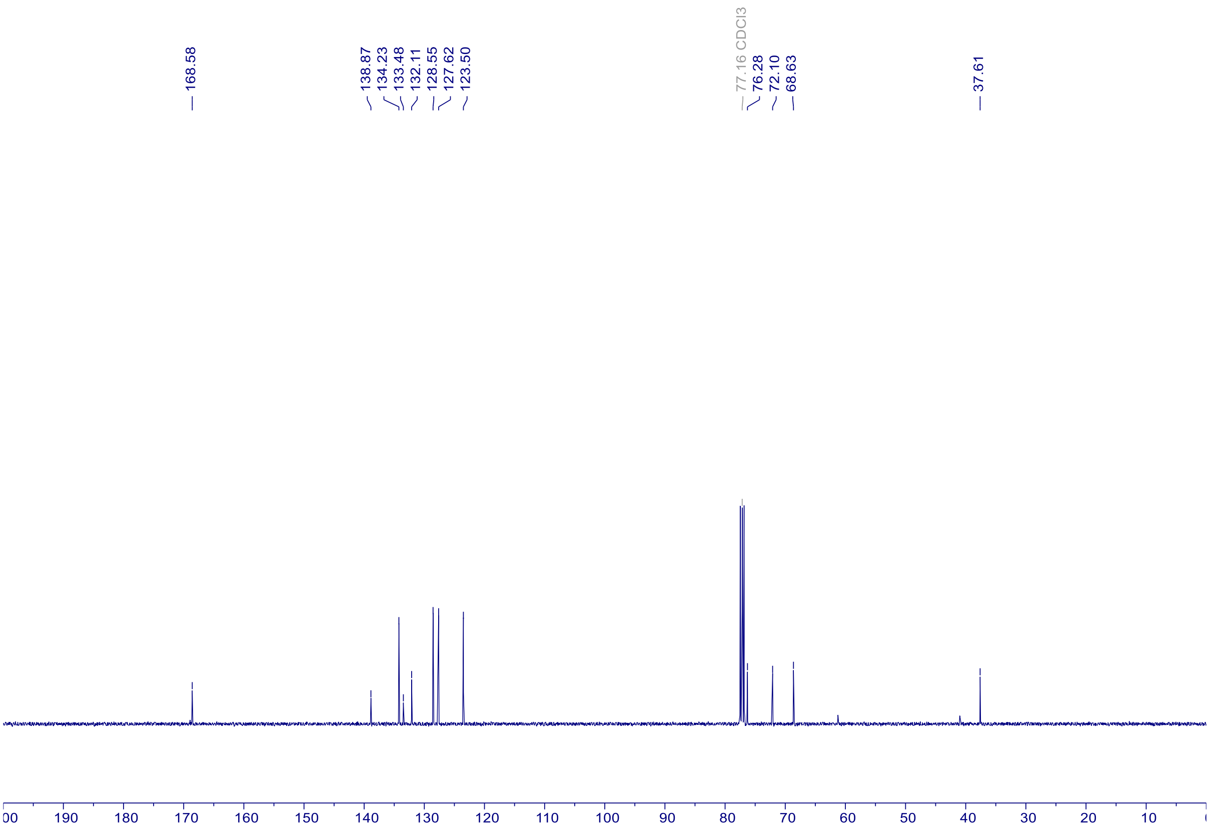

4br – ^{1}H NMR (400 MHz, $CDCl_3$)

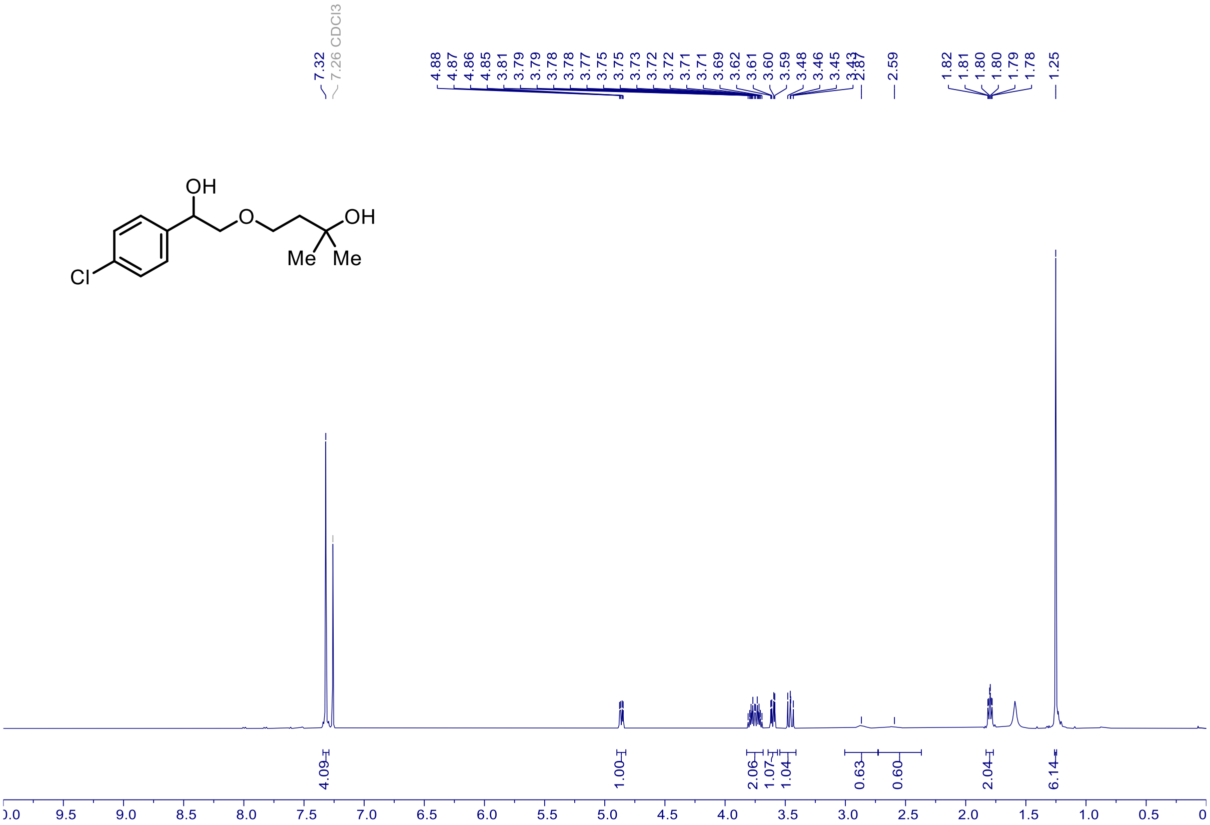

4br – ^{13}C NMR (101 MHz, $CDCl_3$)

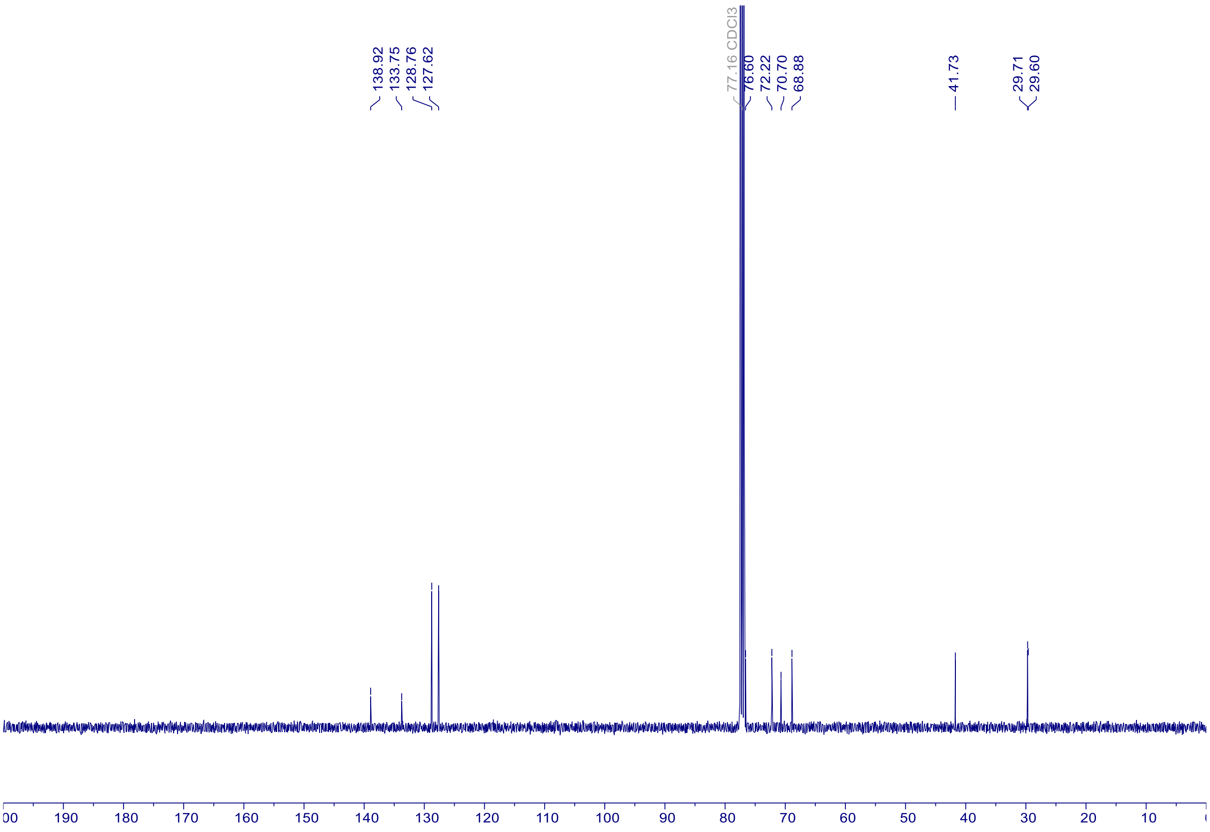

4bs – ^{1}H NMR (400 MHz, $CDCl_3$)

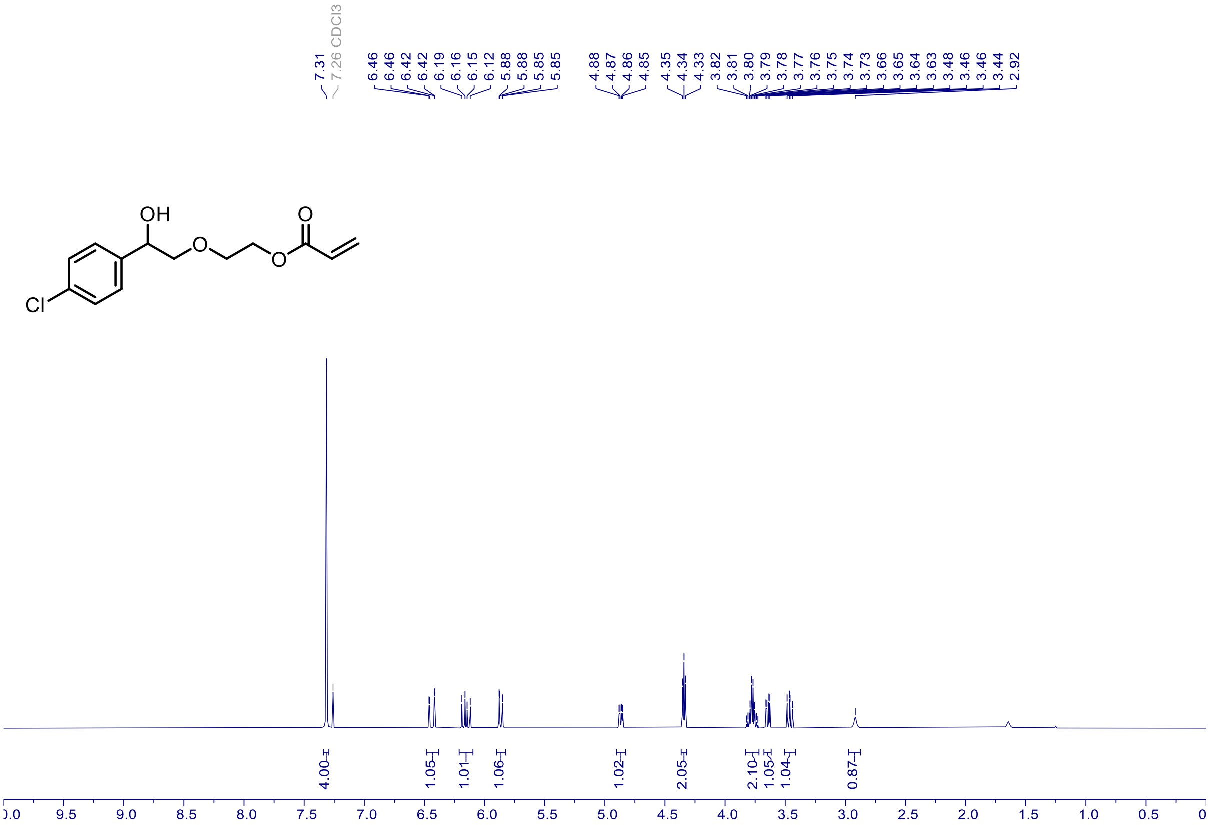

4bs – ^{13}C NMR (101 MHz, $CDCl_3$)

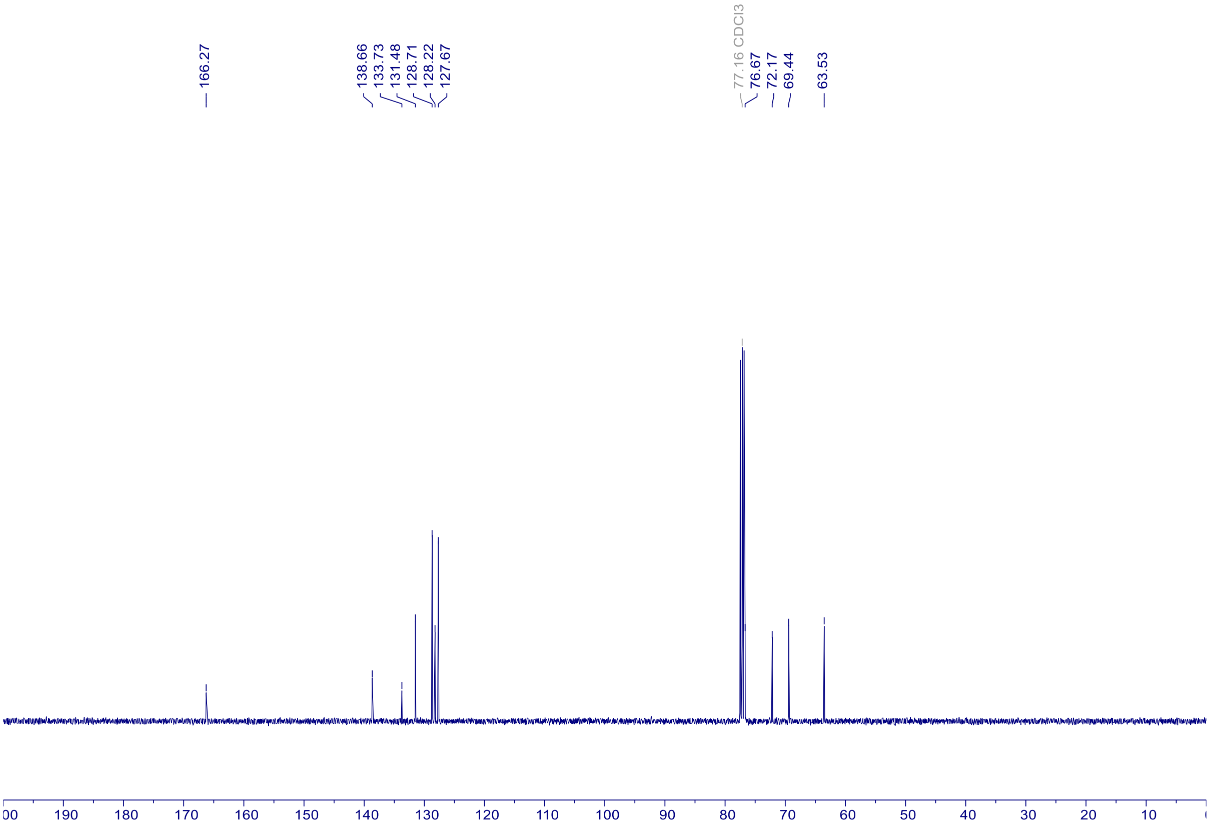

4bt – ^{1}H NMR (400 MHz, $CDCl_3$)

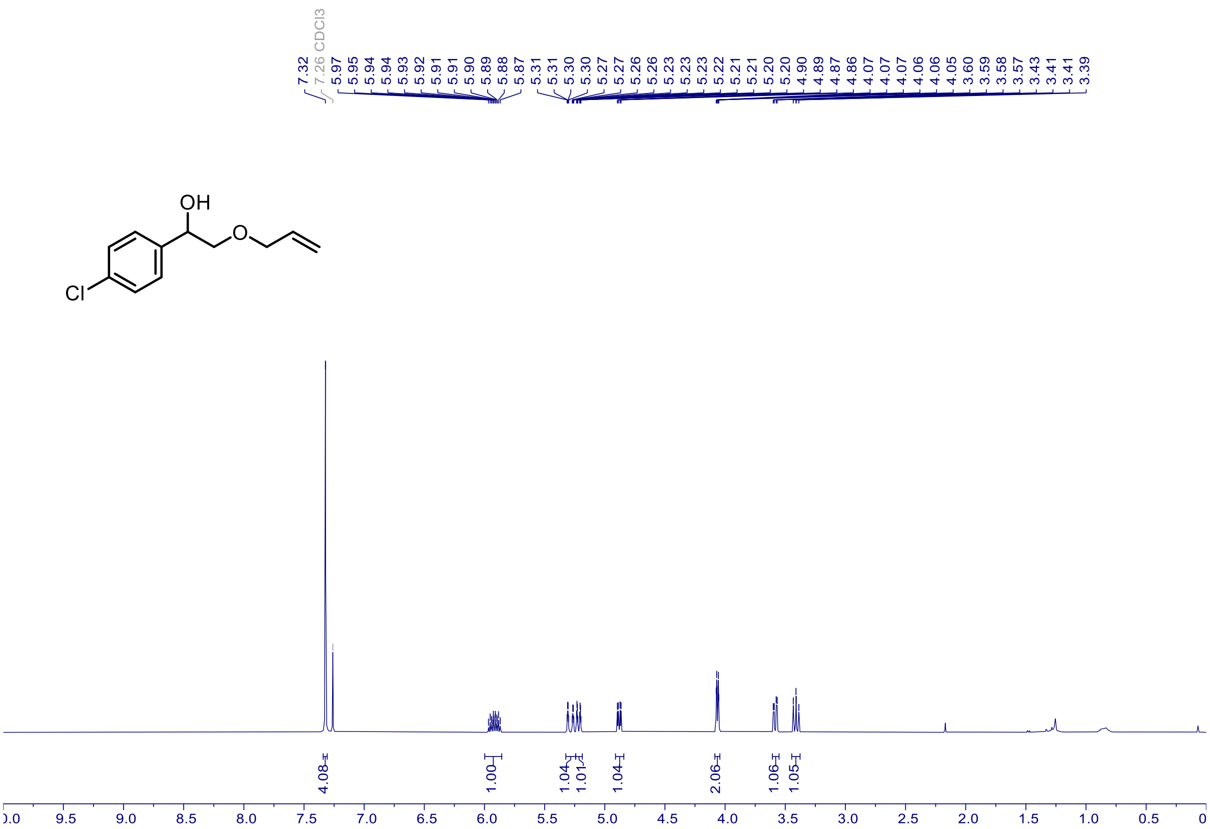

4bt – ^{13}C NMR (101 MHz, $CDCl_3$)

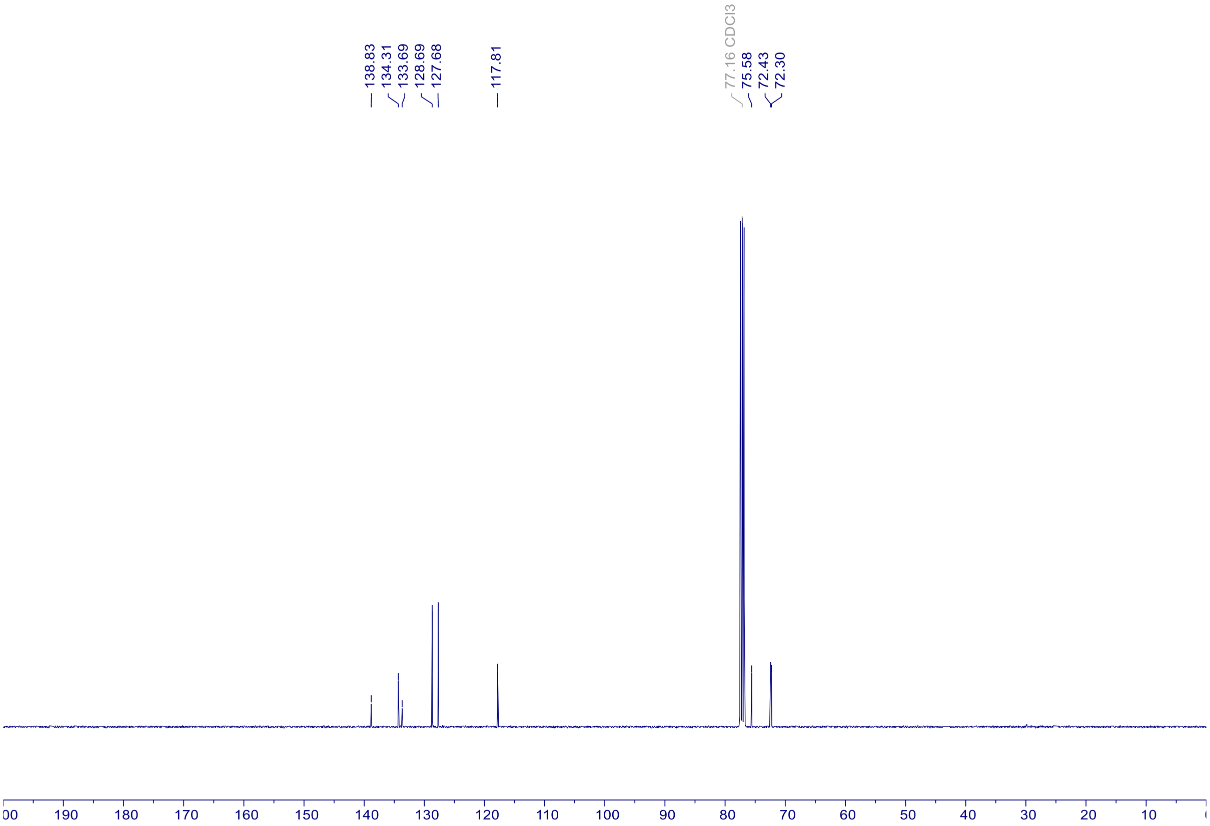

4bu – ^{1}H NMR (400 MHz, $CDCl_3$)

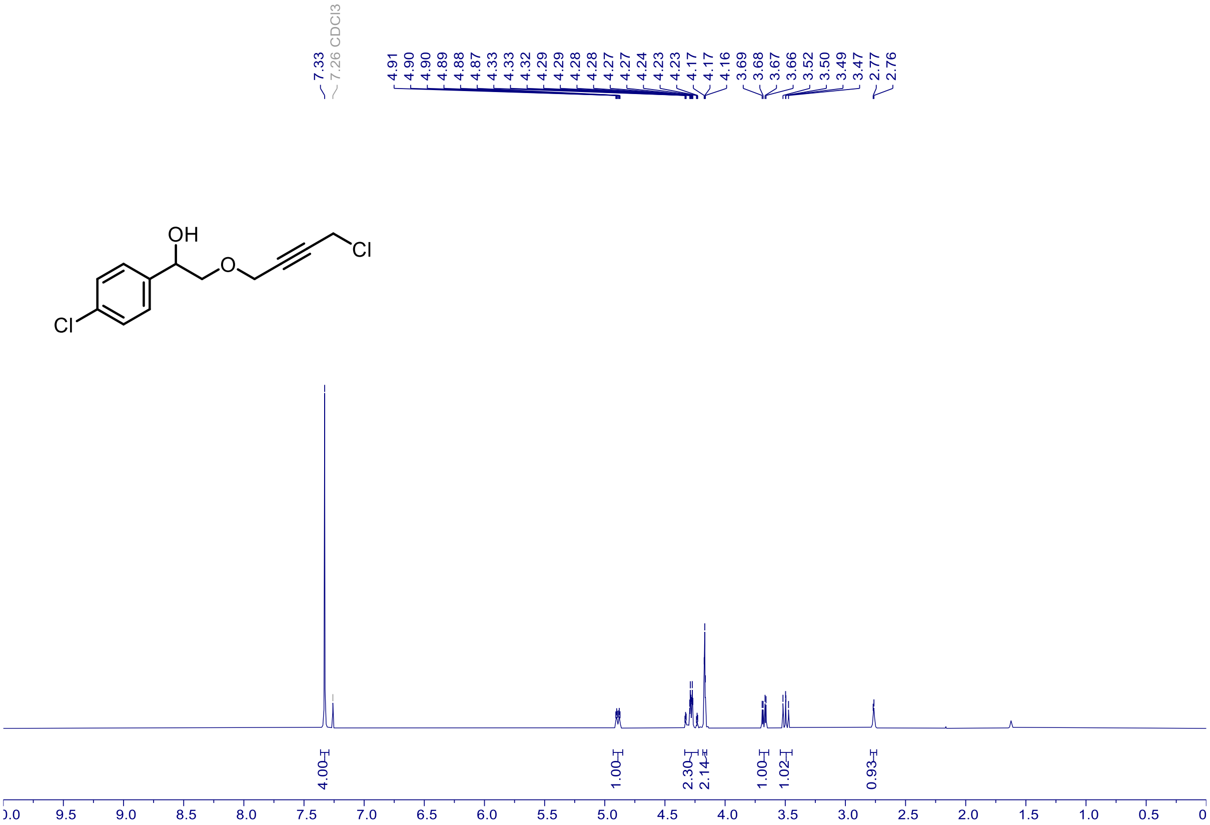

4bu – ^{13}C NMR (101 MHz, $CDCl_3$)

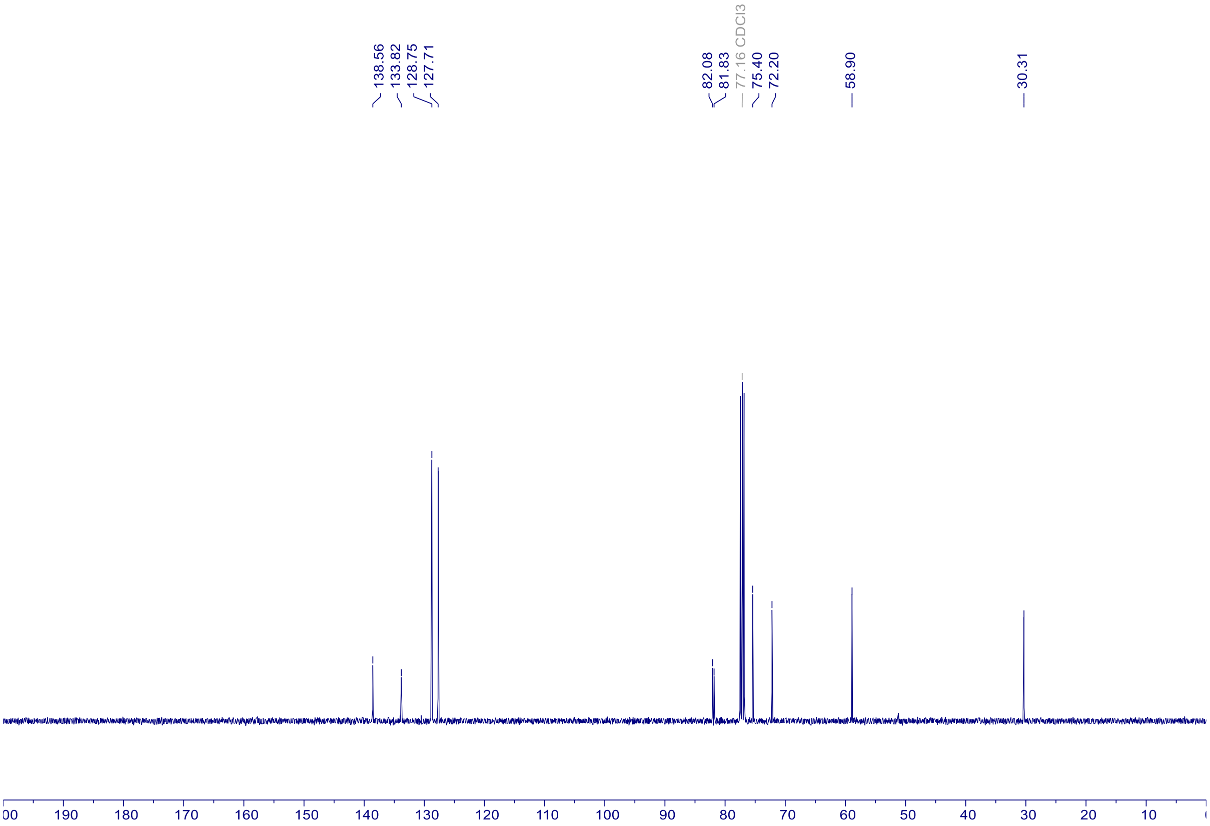

4bv – ^{1}H NMR (400 MHz, $CDCl_3$)

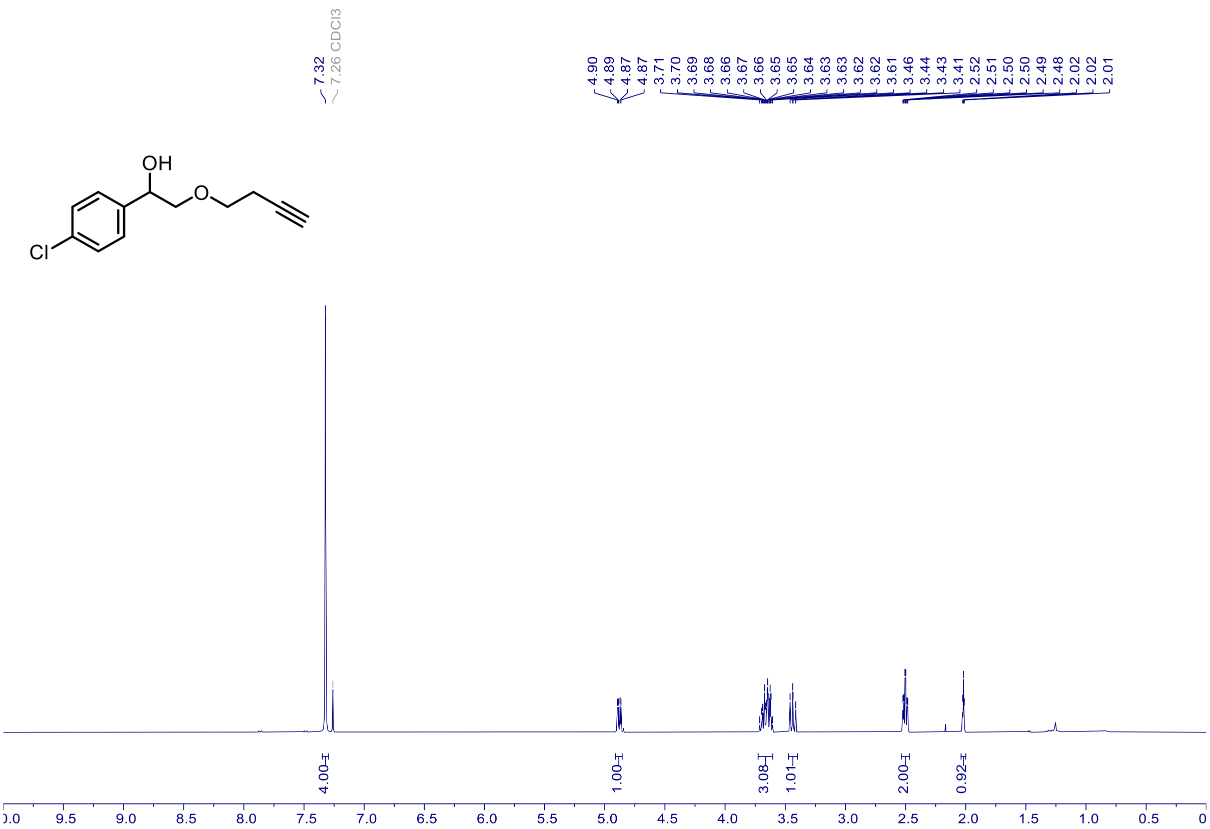

4bv – ^{13}C NMR (101 MHz, $CDCl_3$)

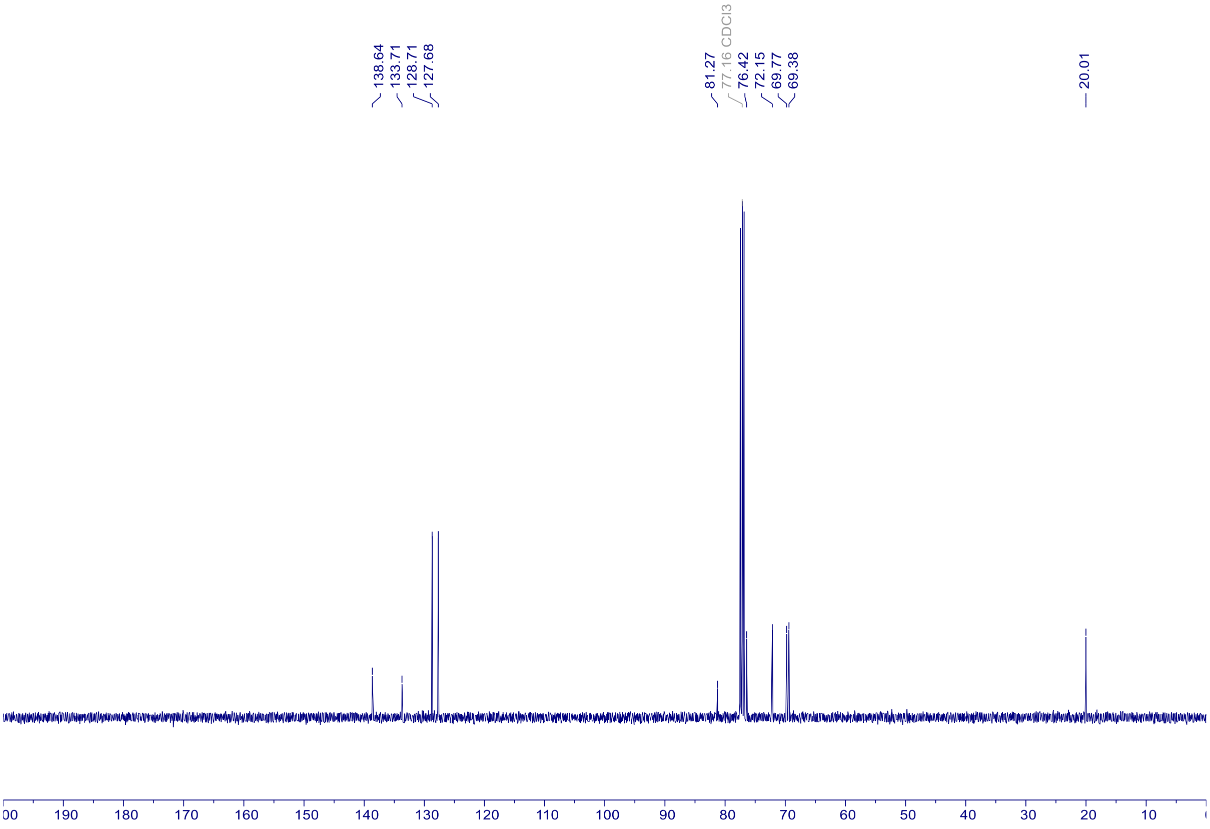

4bw – ^{1}H NMR (400 MHz, $CDCl_3$)

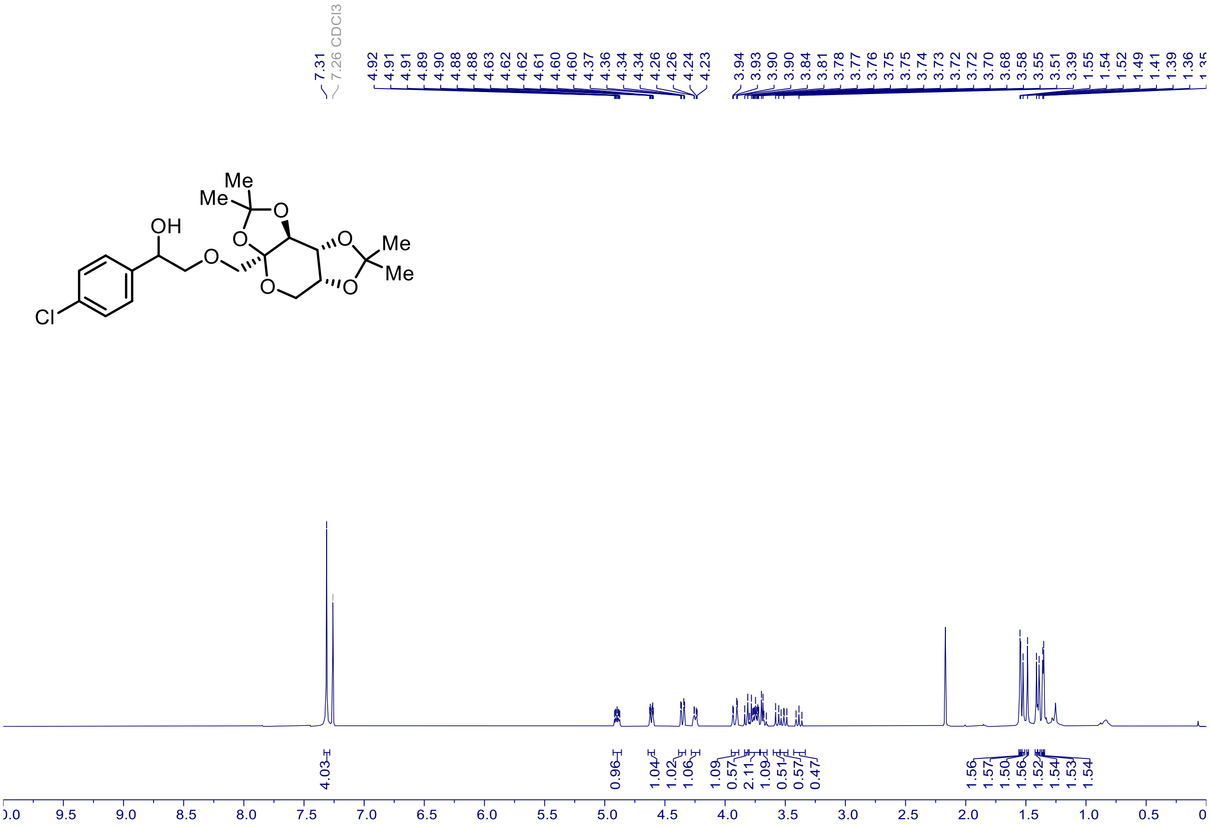

4bw – ^{13}C NMR (101 MHz, $CDCl_3$)

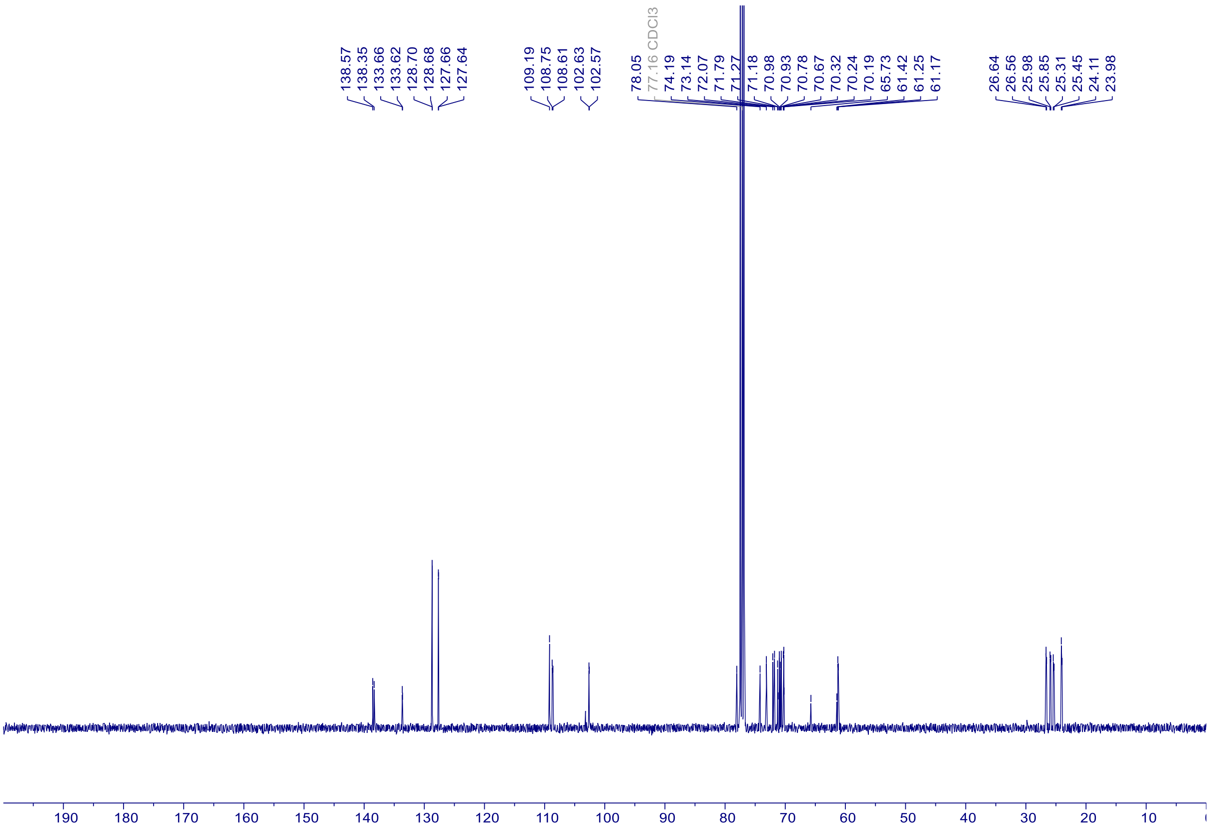

4bx – ^{1}H NMR (400 MHz, $CDCl_3$, 1:1 mixture of diastereoisomers)

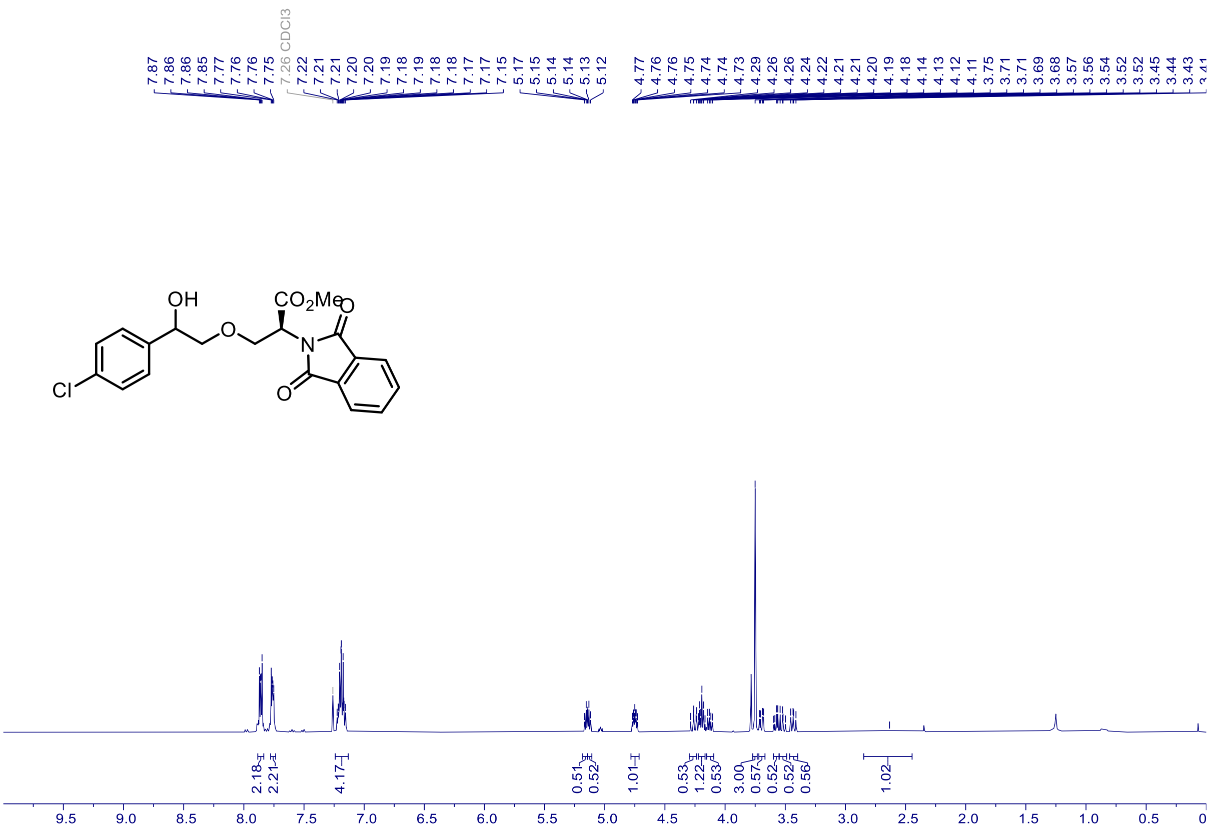

4bx – ^{13}C NMR (101 MHz, $CDCl_3$, 1:1 mixture of diastereoisomers)

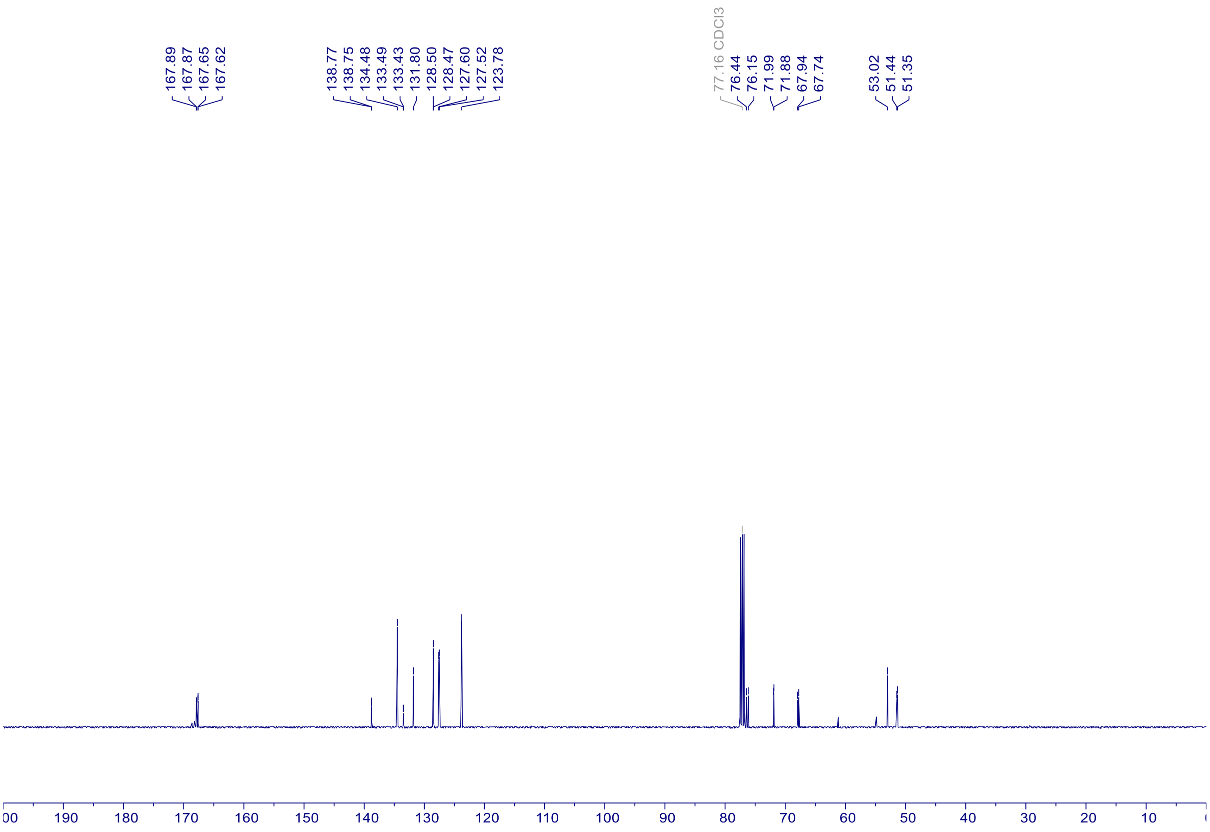

4bx' – ^{1}H NMR (400 MHz, $CDCl_3$, 1:1 mixture of diastereoisomers)

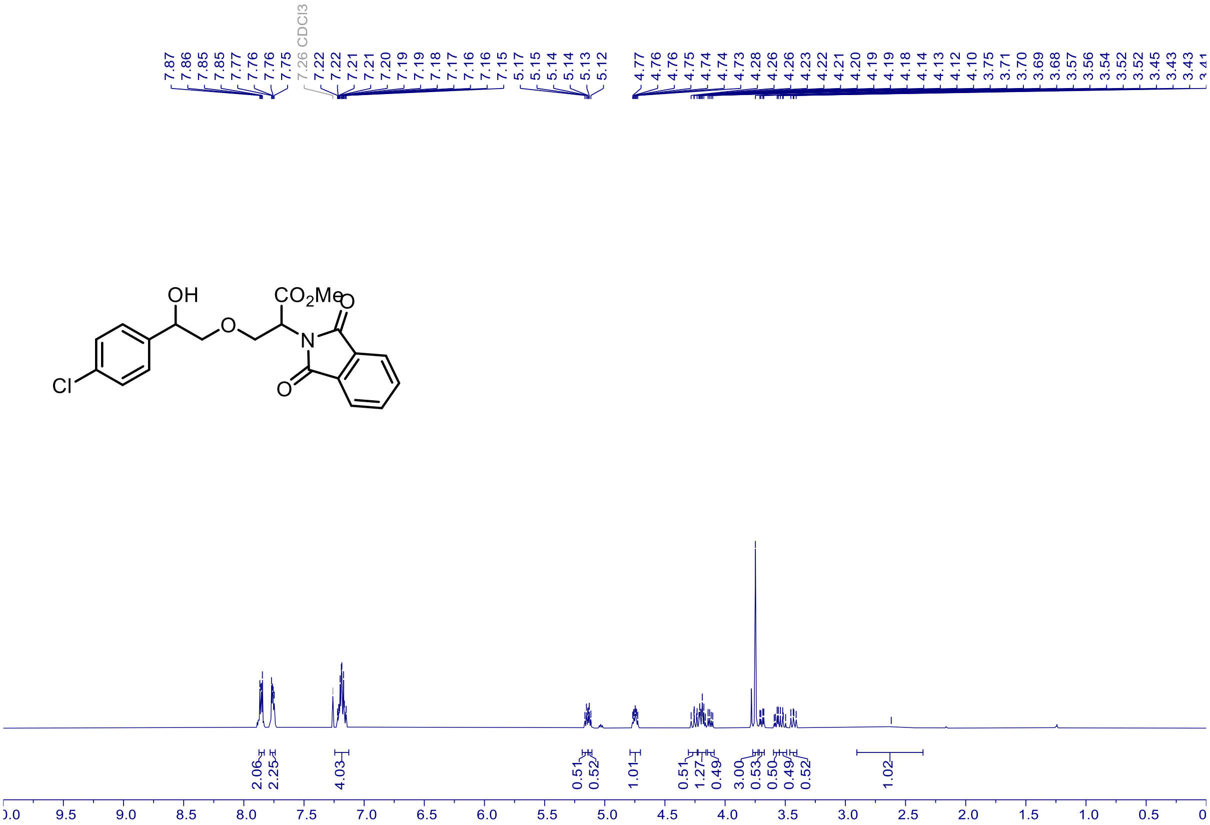

4bx' – ^{13}C NMR (101 MHz, $CDCl_3$, 1:1 mixture of diastereoisomers)

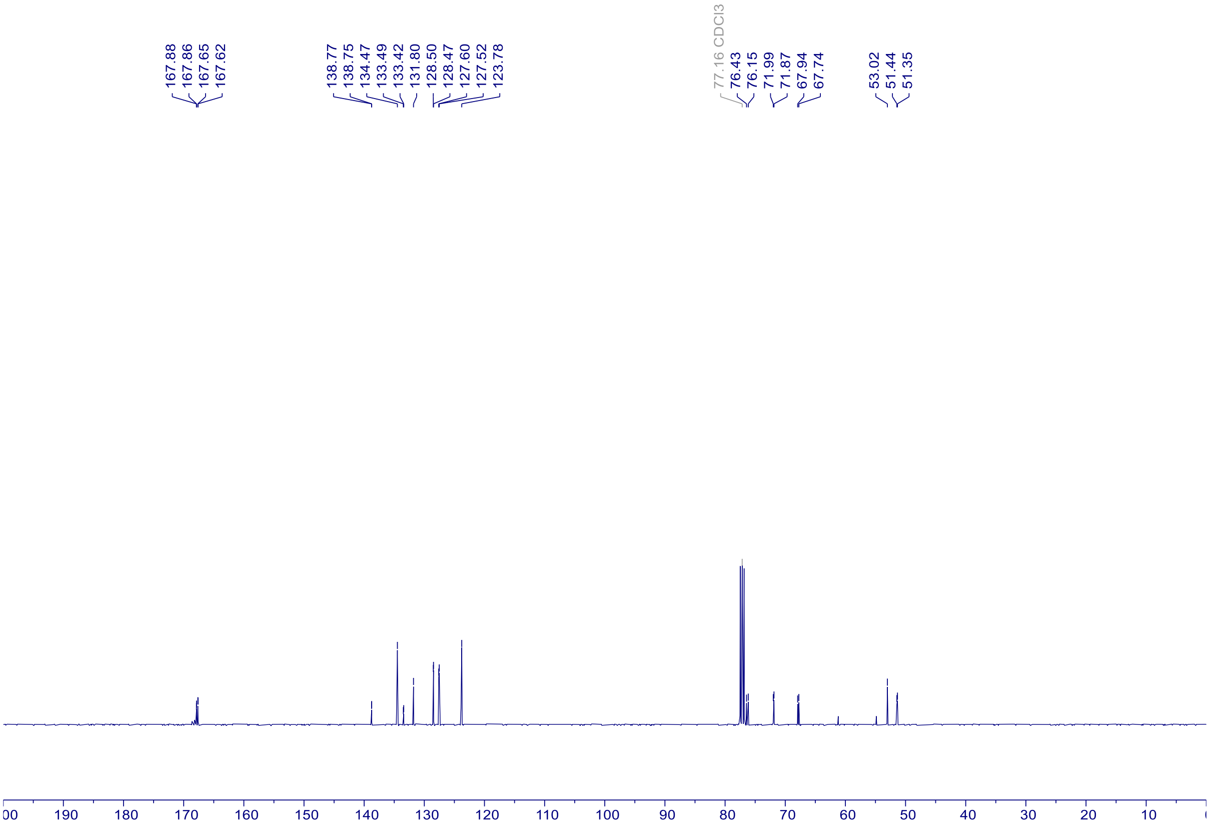

4by – ^{1}H NMR (400 MHz, $CDCl_3$)

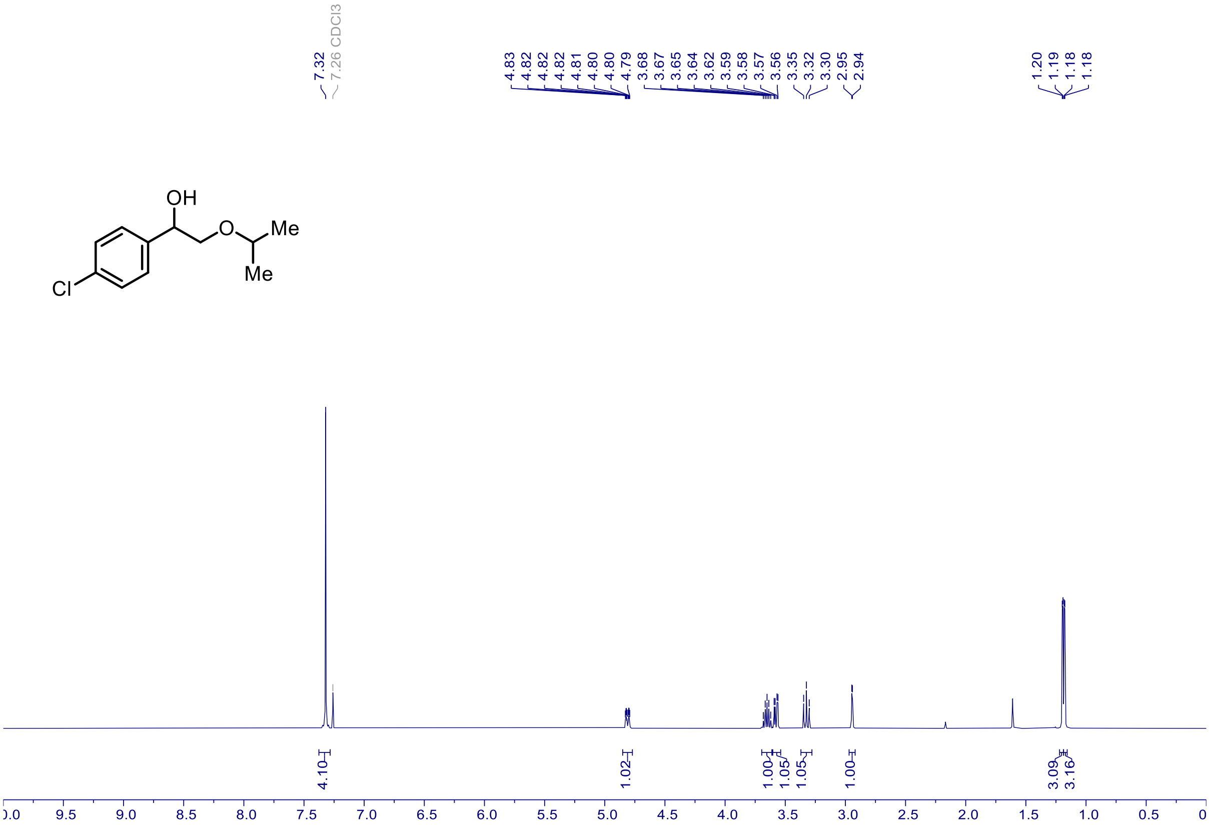

4by – ^{13}C NMR (101 MHz, $CDCl_3$)

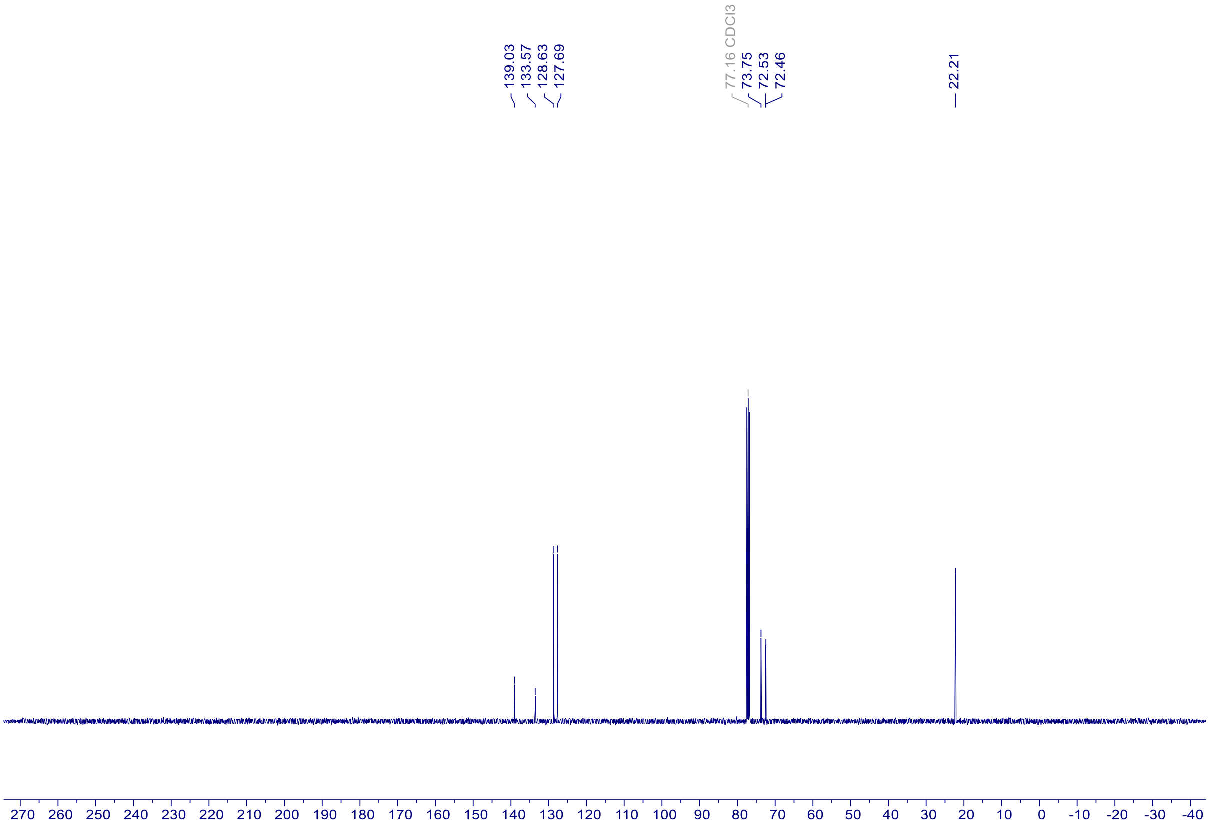

4bz – ^{1}H NMR (400 MHz, $CDCl_3$, 1:1 mixture of diastereoisomers)

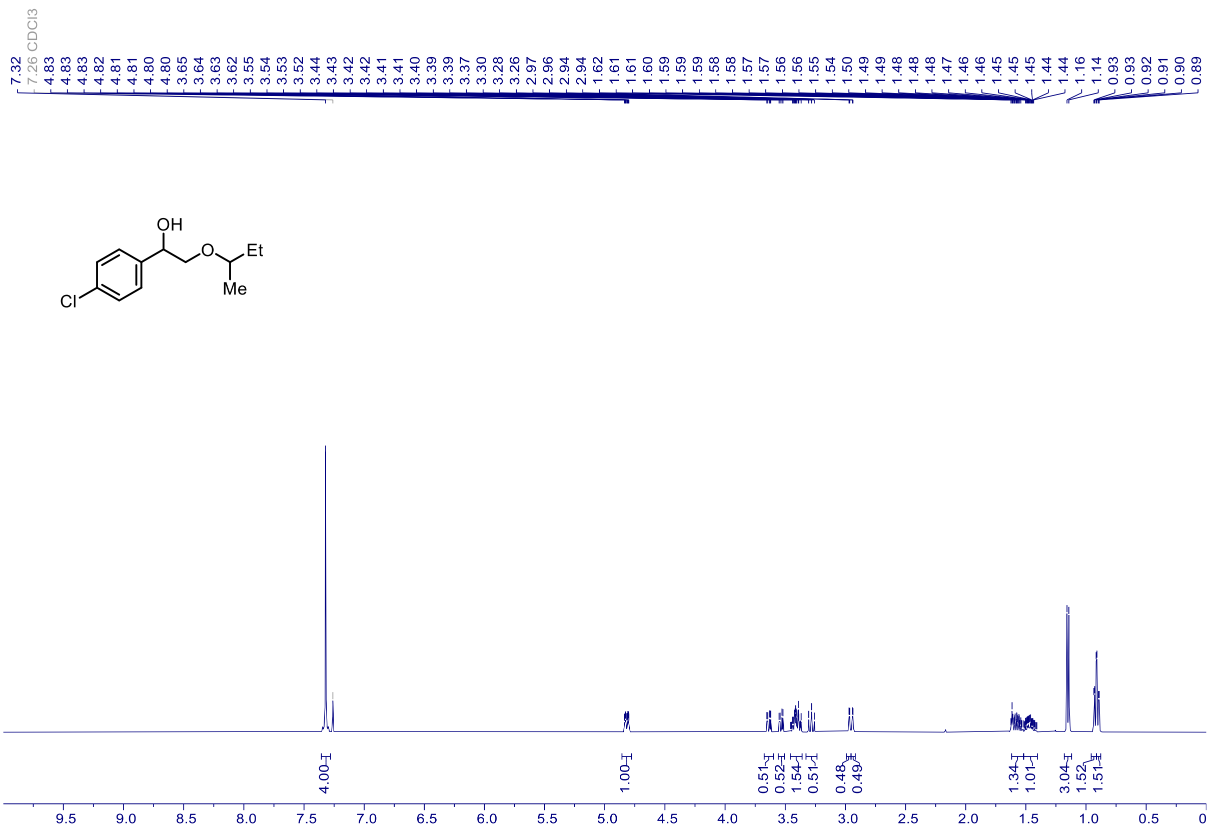

4bz – ^{13}C NMR (101 MHz, $CDCl_3$, 1:1 mixture of diastereoisomers)

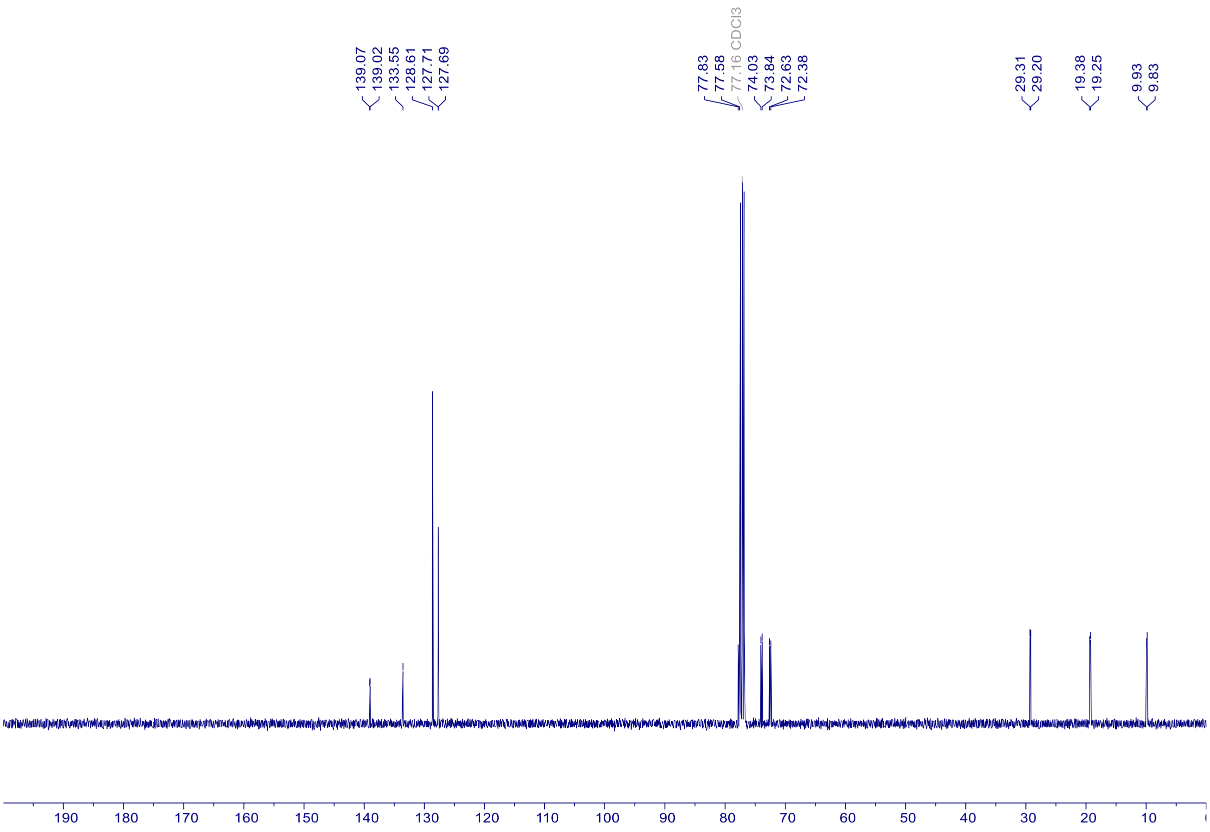

4ca – ^{1}H NMR (400 MHz, $CDCl_3$, 1:1 mixture of diastereoisomers)

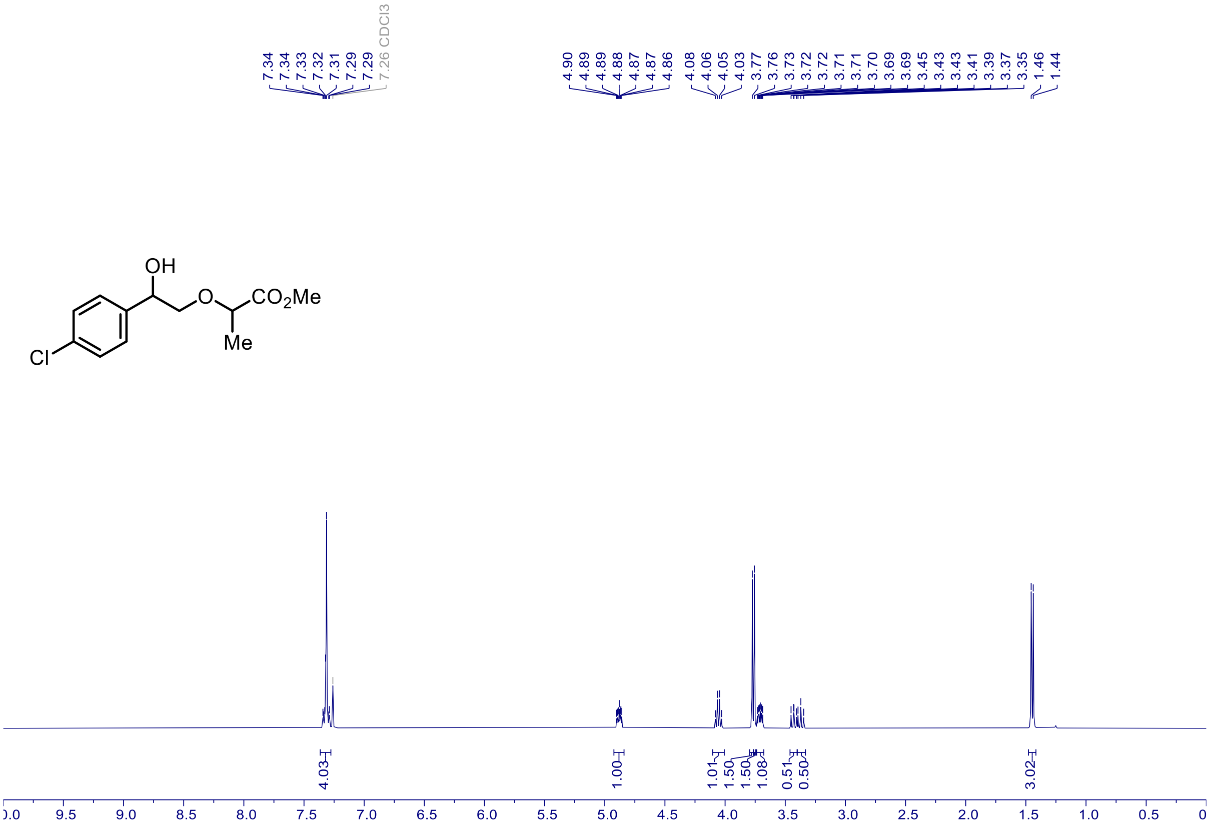

4ca – ^{13}C NMR (101 MHz, $CDCl_3$, 1:1 mixture of diastereoisomers)

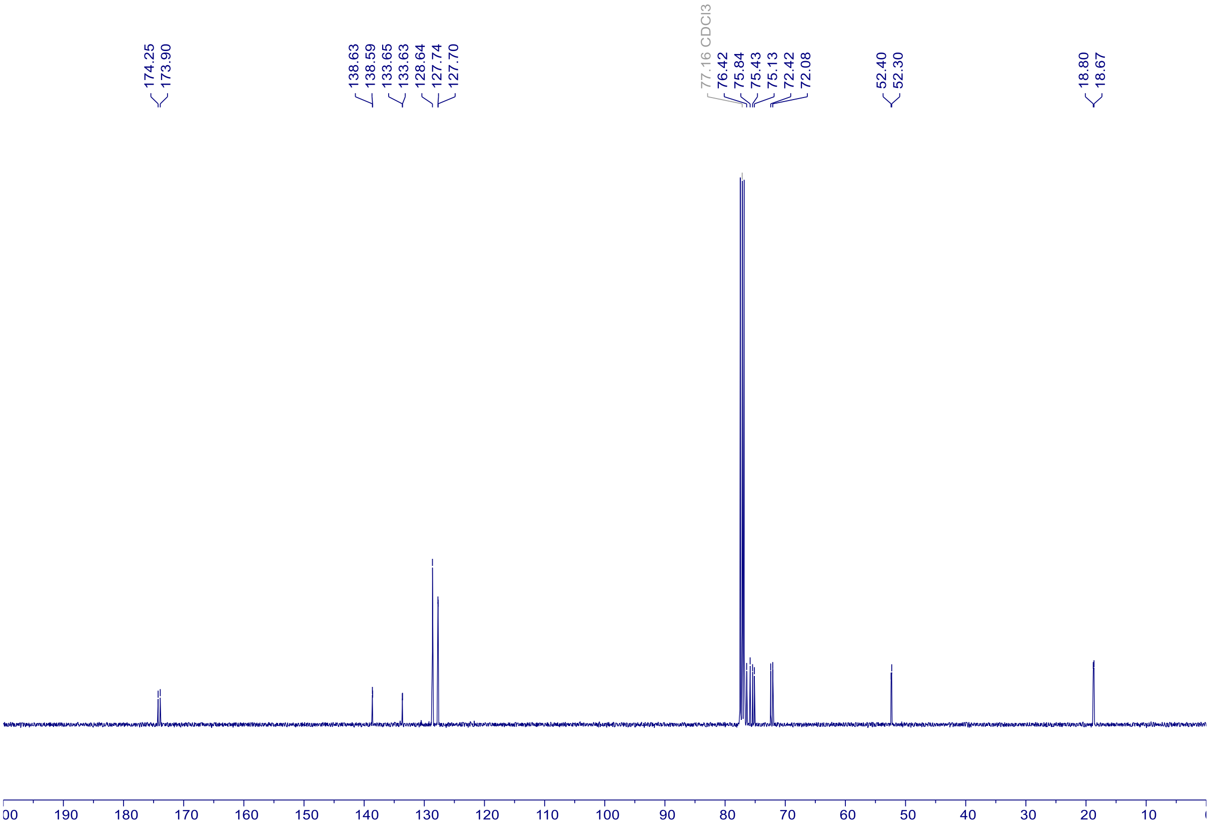

4cb – ^{1}H NMR (400 MHz, $CDCl_3$, 1:1 mixture of diastereoisomers)

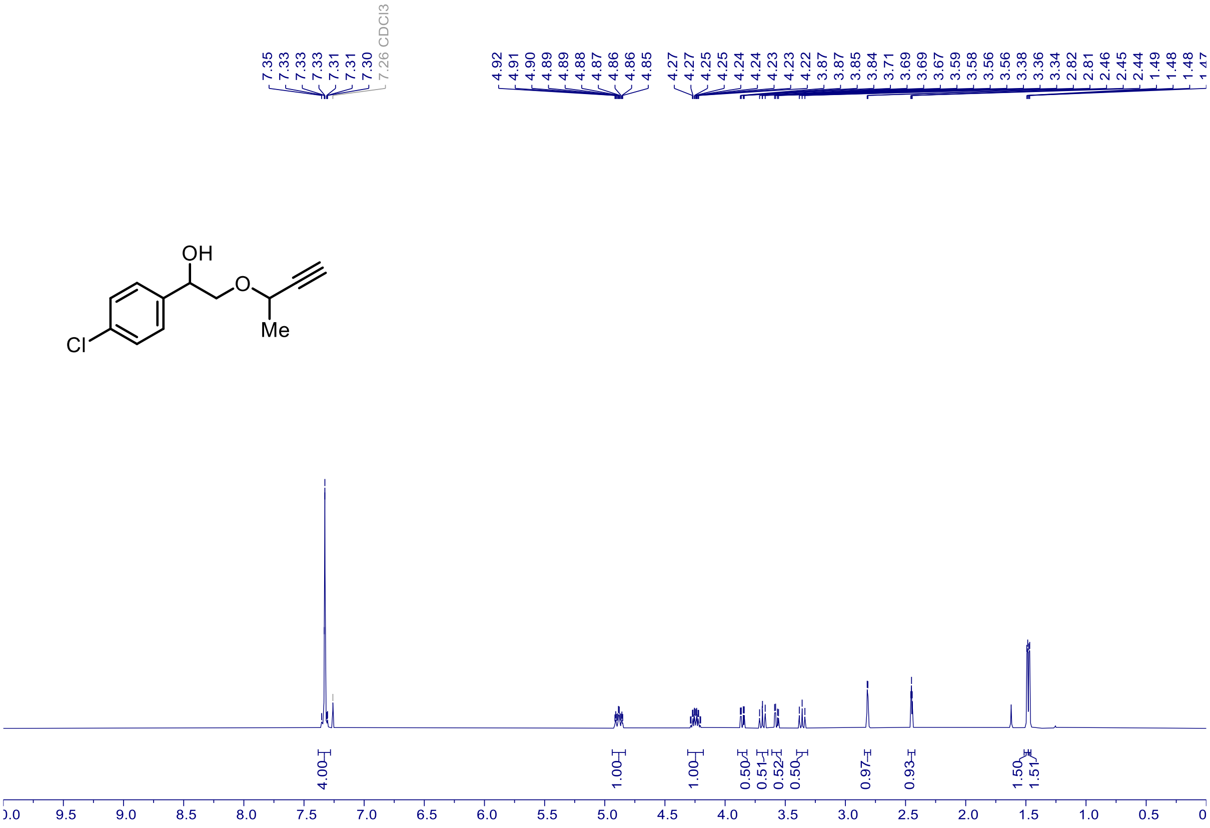

4cb – ^{13}C NMR (101 MHz, $CDCl_3$, 1:1 mixture of diastereoisomers)

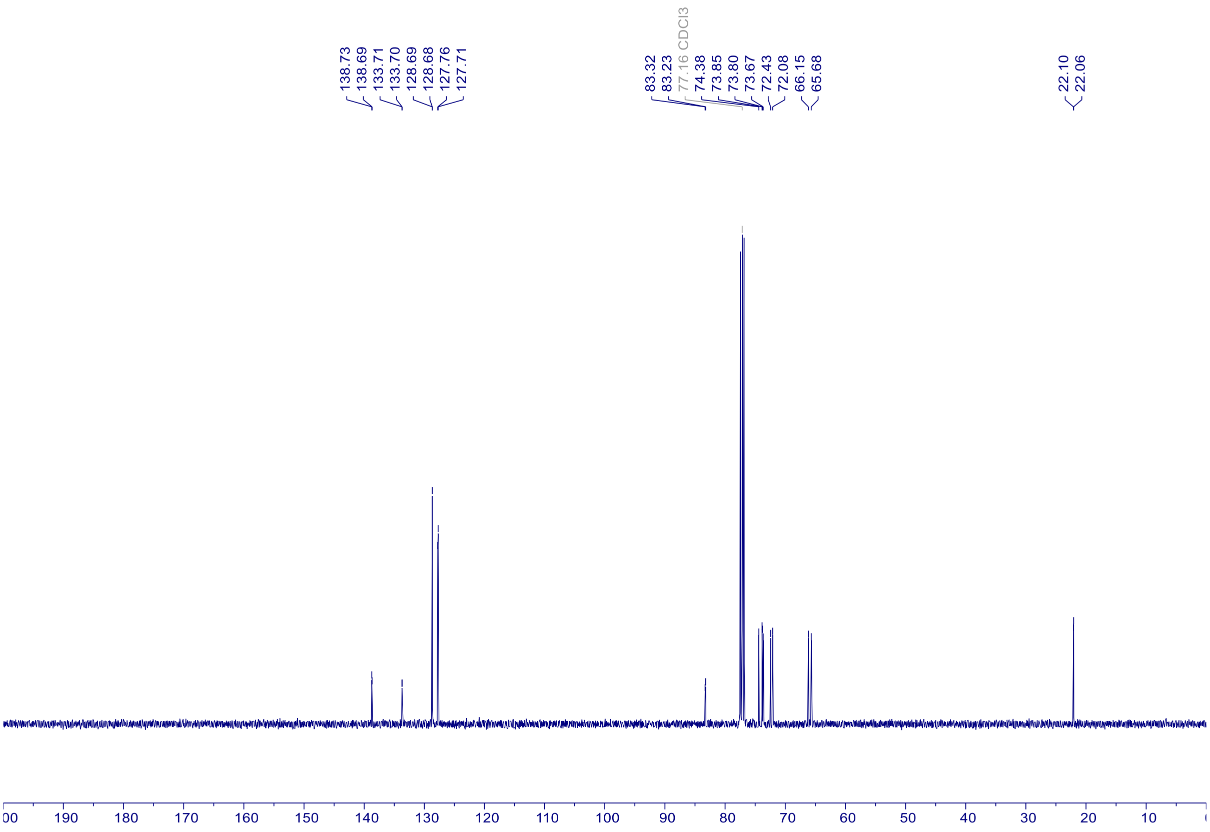

4cc – ^{1}H NMR (400 MHz, $CDCl_3$)

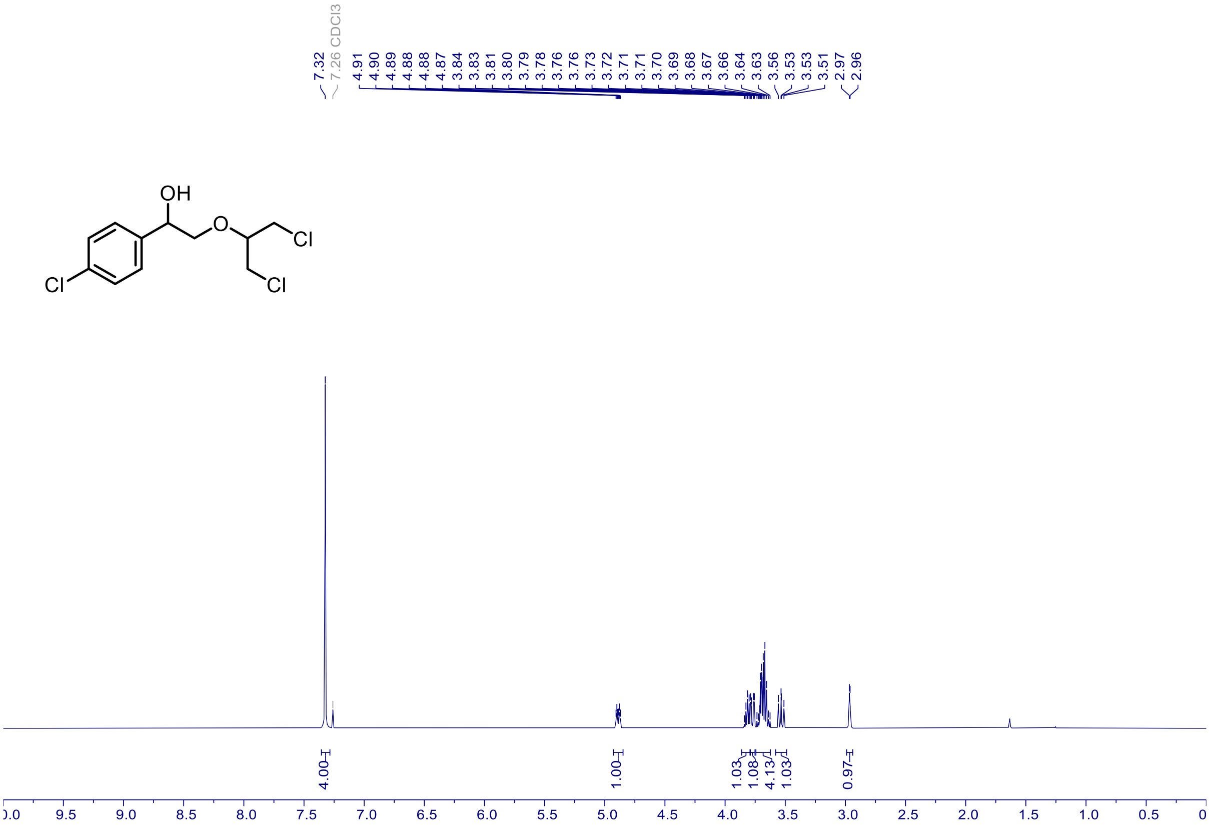

4cc – ^{13}C NMR (101 MHz, $CDCl_3$)

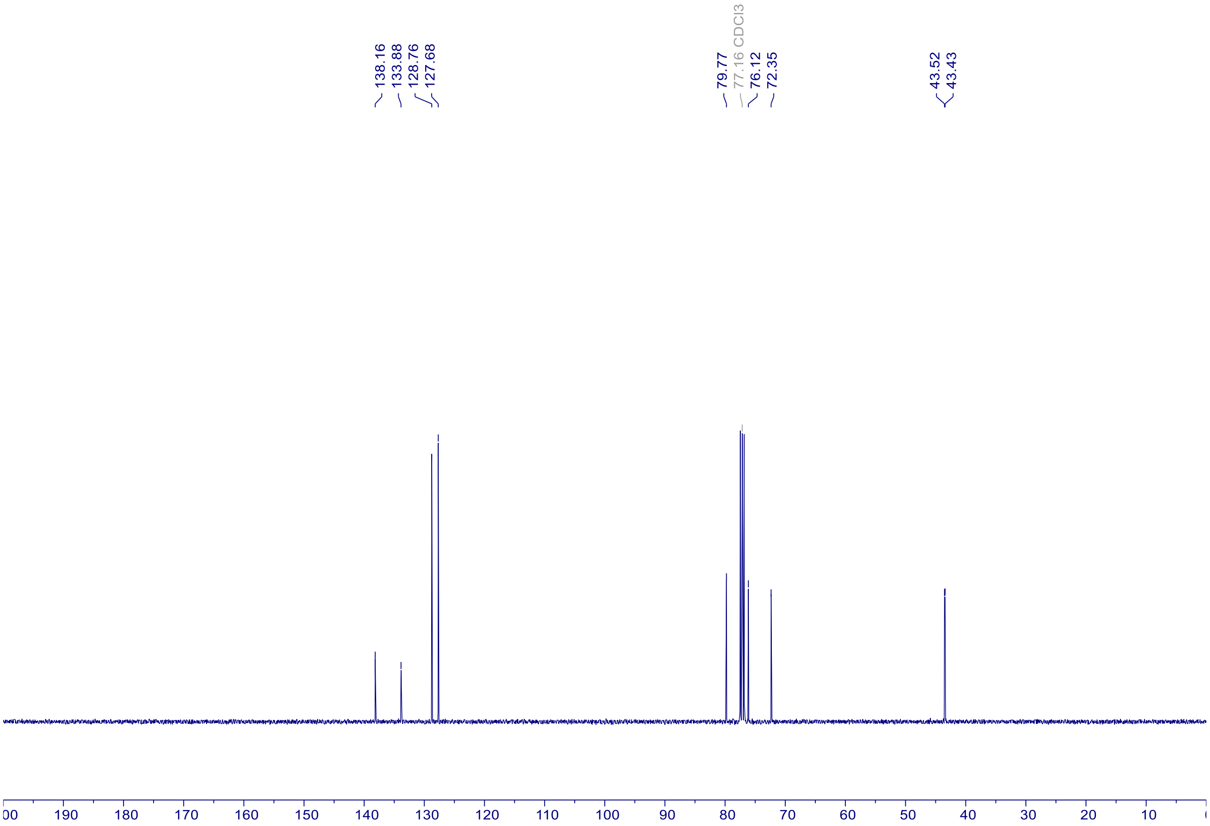

4cd – ^{1}H NMR (400 MHz, $CDCl_3$)

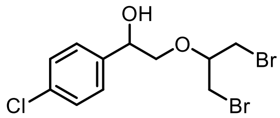

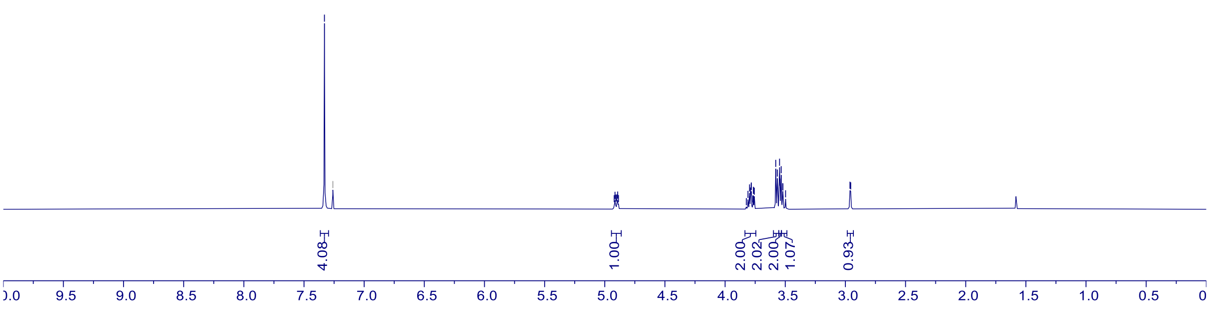

4cd – ^{13}C NMR (101 MHz, $CDCl_3$)

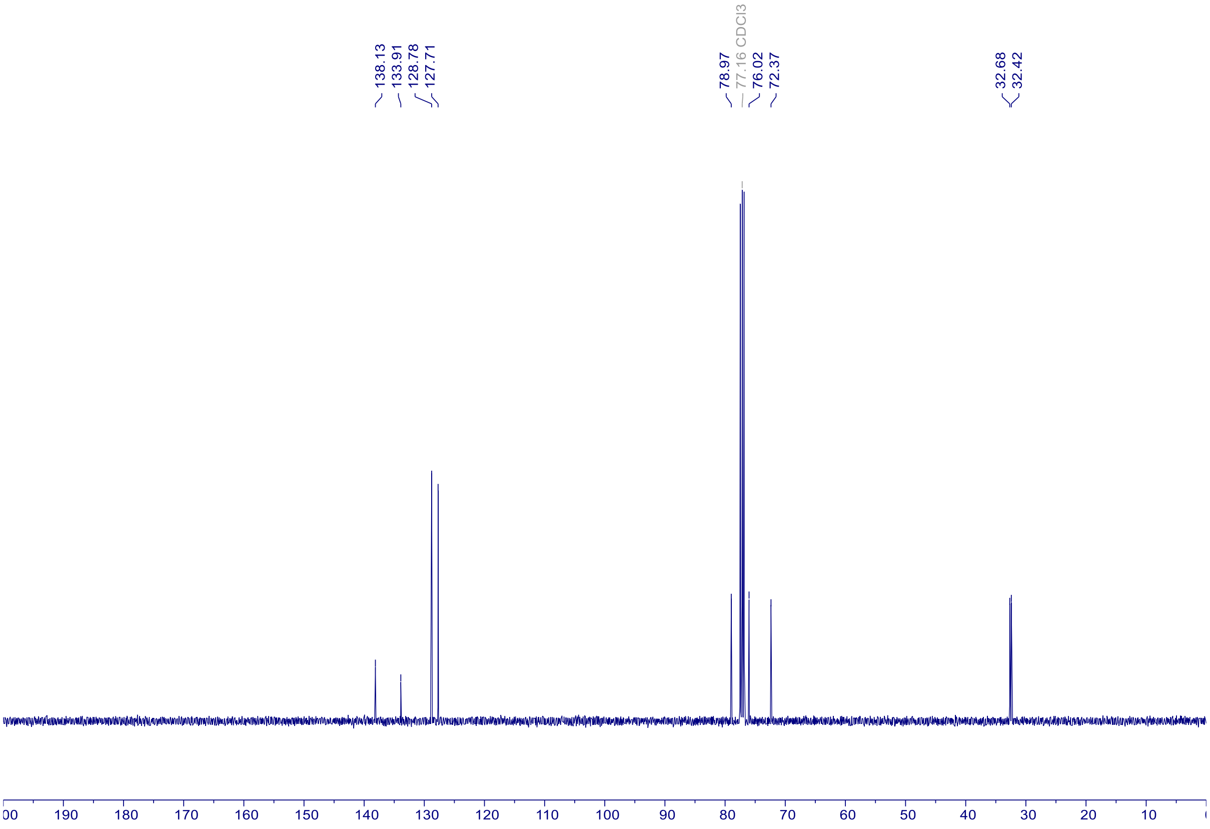

rac-**4ce** – ^{1}H NMR (400 MHz, $CDCl_3$, 1.1:1 mixture of diastereoisomers)

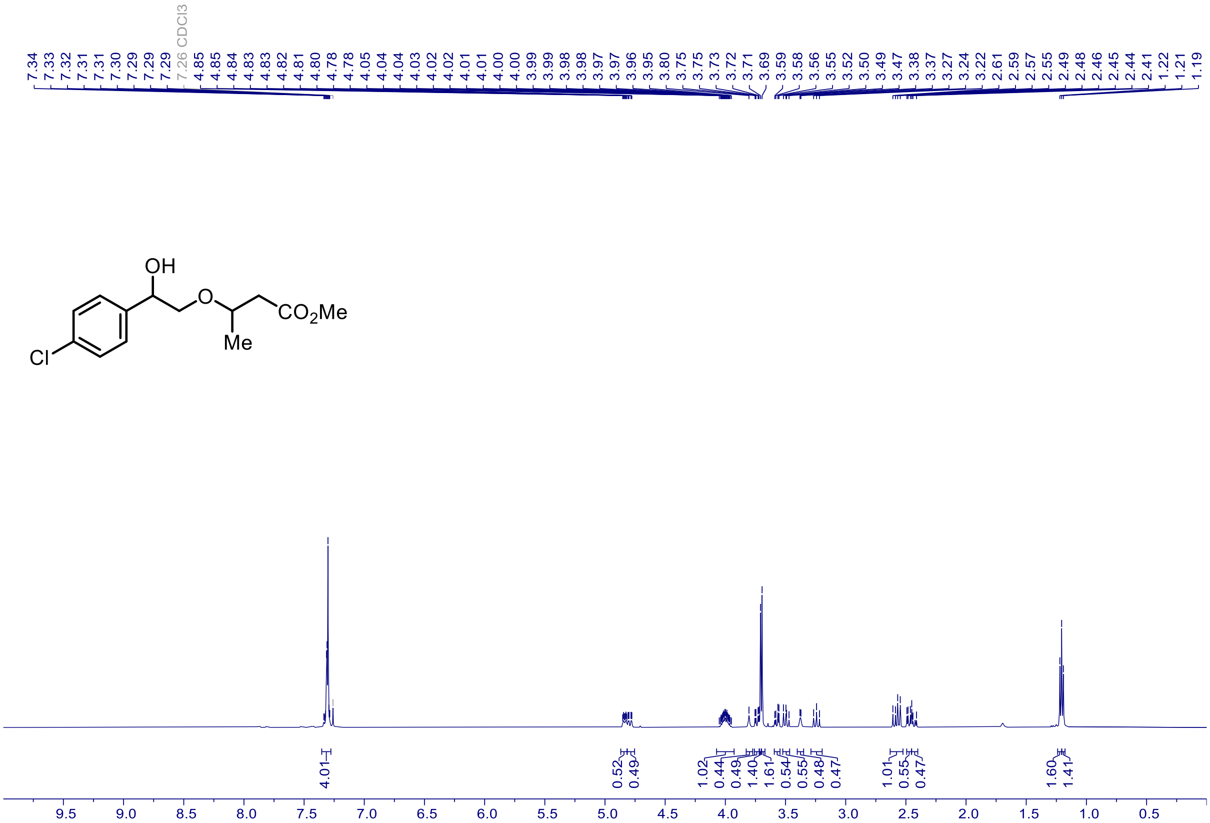

rac-**4ce** – ^{13}C NMR (101 MHz, $CDCl_3$, 1.1:1 mixture of diastereoisomers)

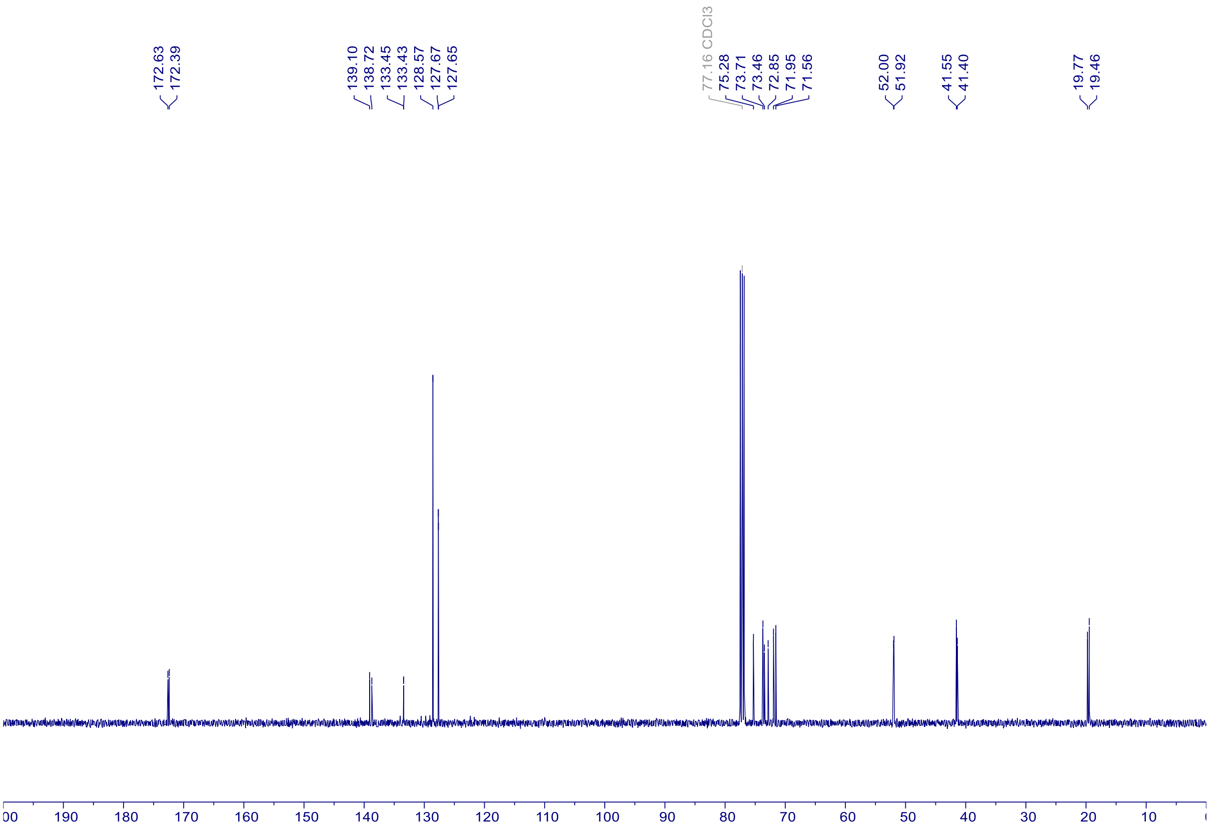

(*S*)-**4ce** – ^{1}H NMR (400 MHz, $CDCl_3$, 1.1:1 mixture of diastereoisomers)

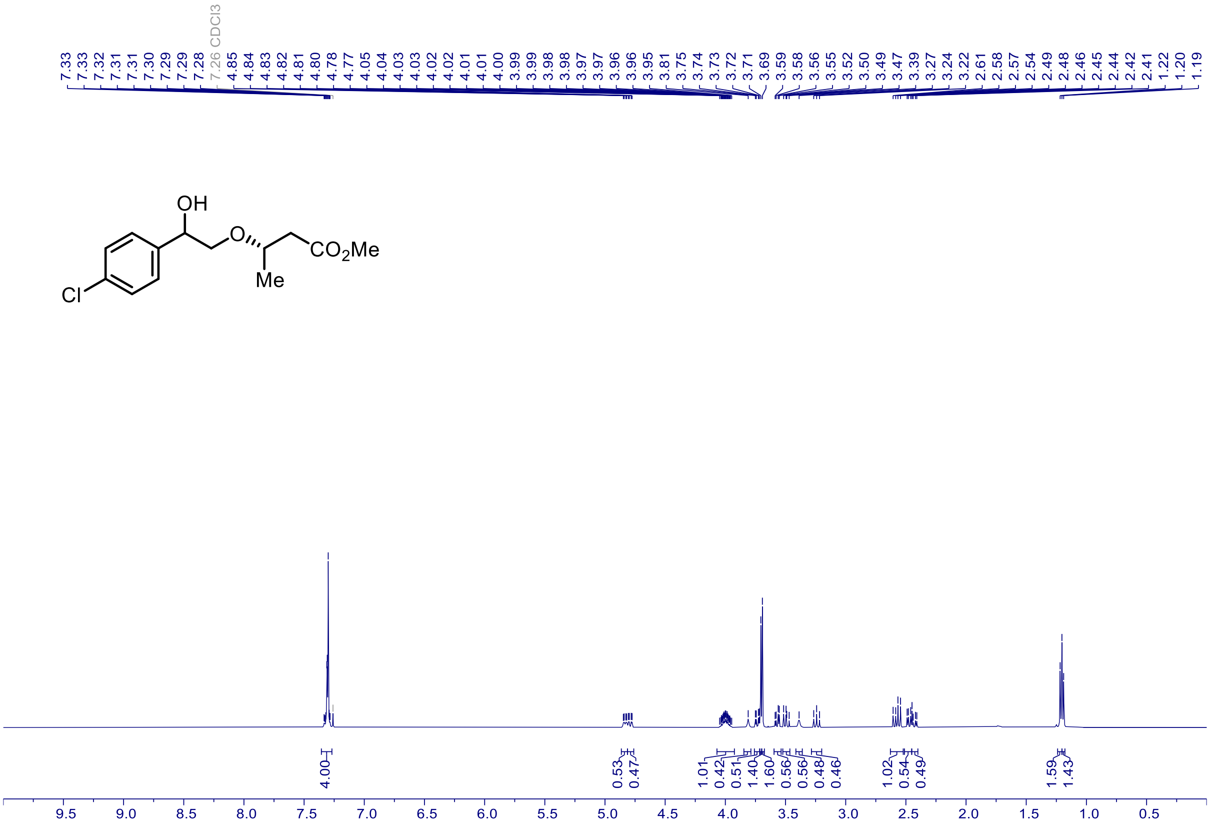

(*S*)-**4ce** – ^{13}C NMR (101 MHz, $CDCl_3$, 1.1:1 mixture of diastereoisomers)

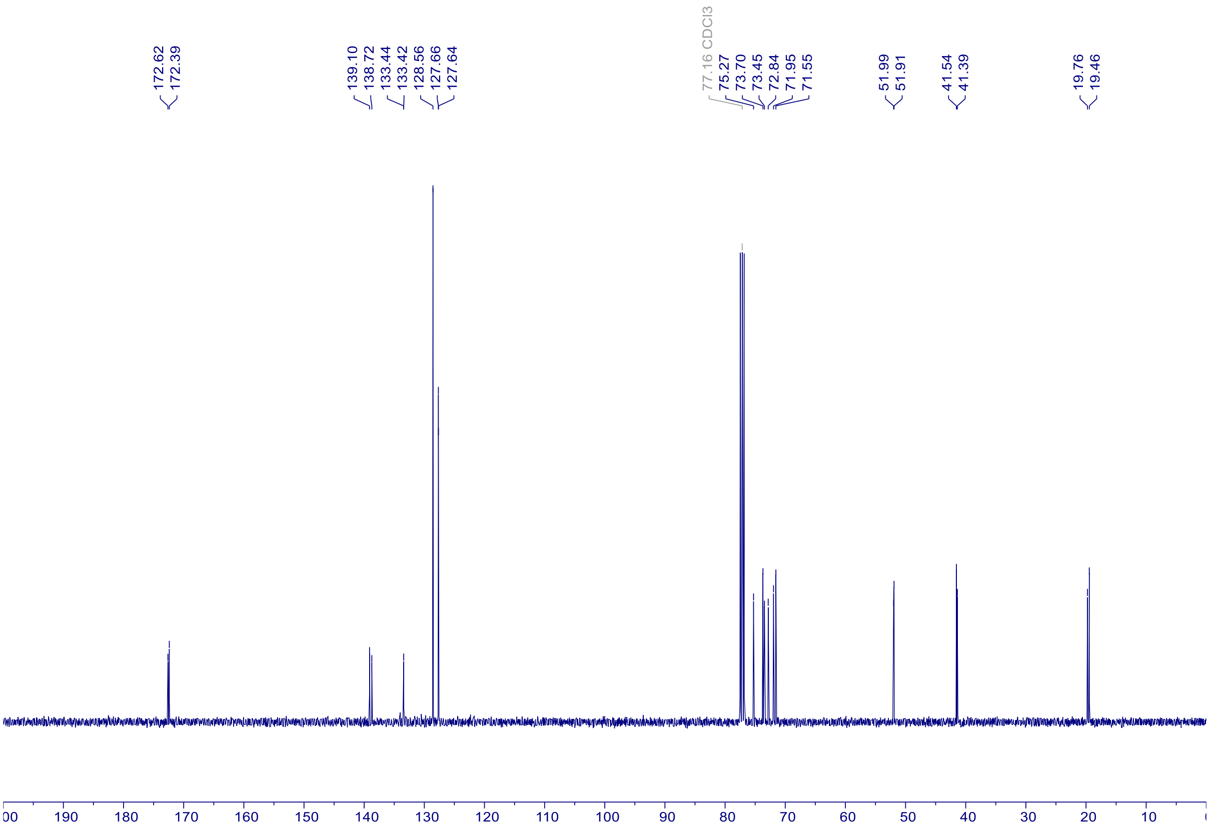

4cf – ^{1}H NMR (400 MHz, $CDCl_3$, 1:1 mixture of diastereoisomers)

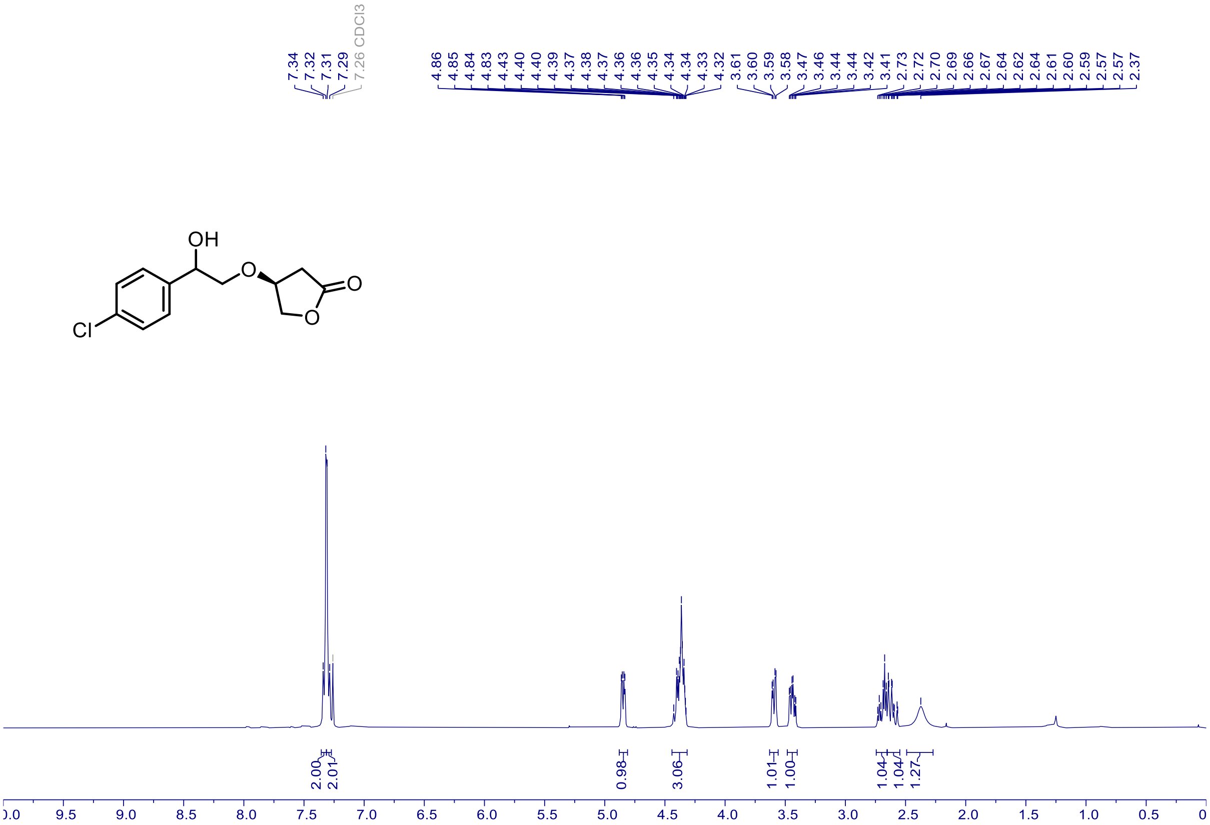

4cf – ^{13}C NMR (101 MHz, $CDCl_3$, 1:1 mixture of diastereoisomers)

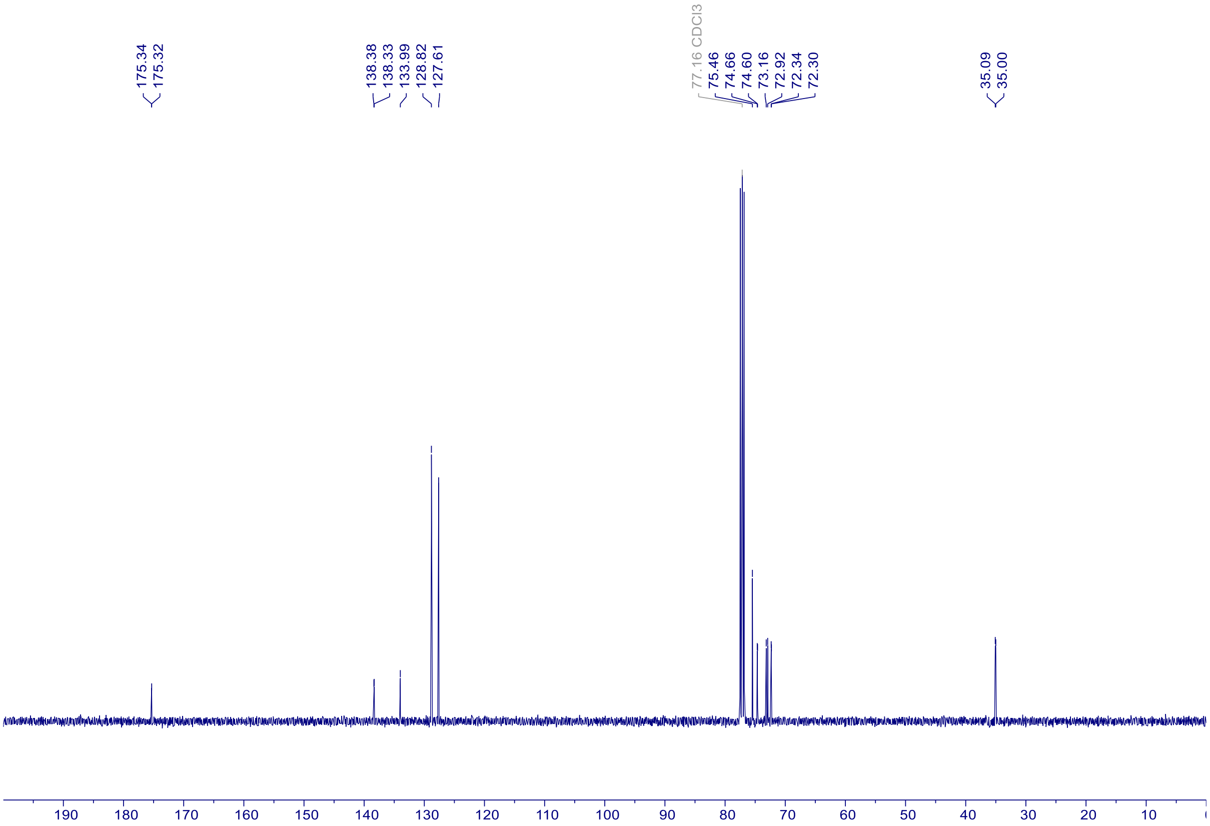

4cg – ^{1}H NMR (500 MHz, $CDCl_3$)

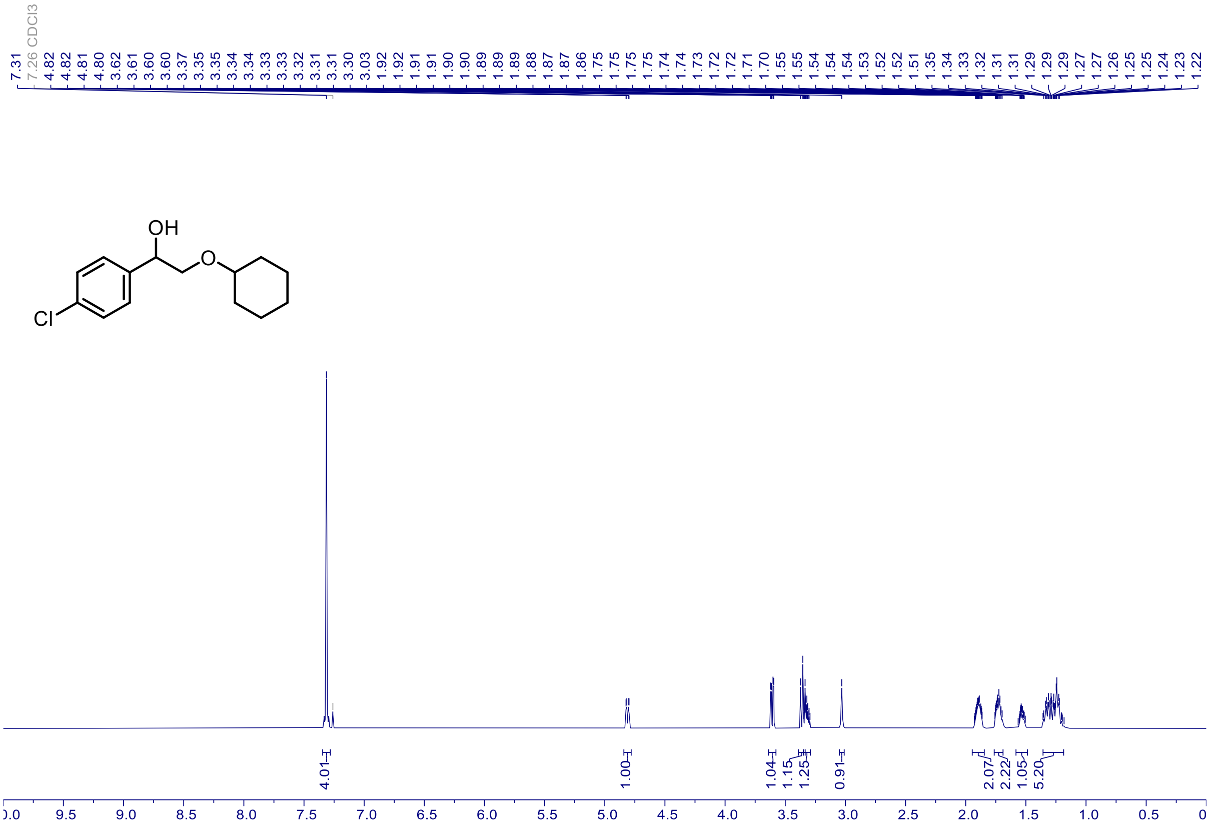

4cg – ^{13}C NMR (126 MHz, $CDCl_3$)

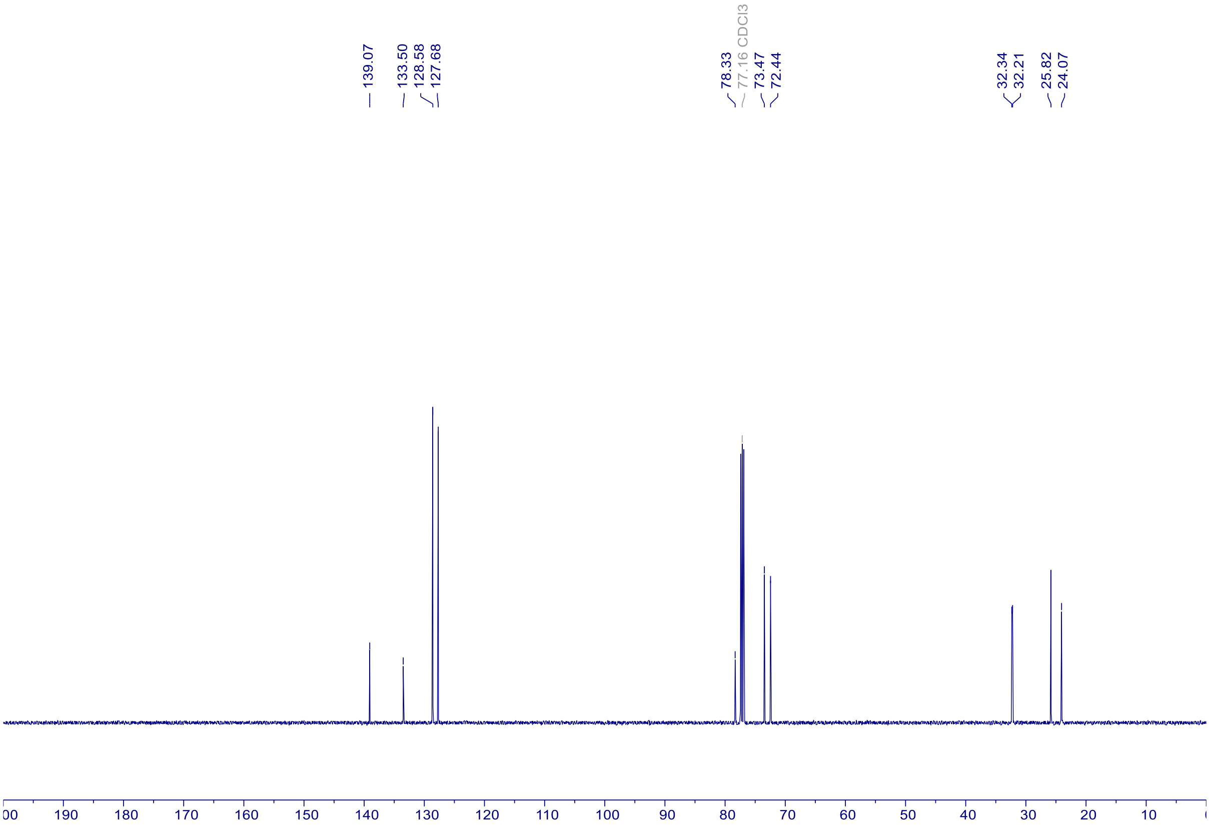

4ch – ^{1}H NMR (400 MHz, $CDCl_3$)

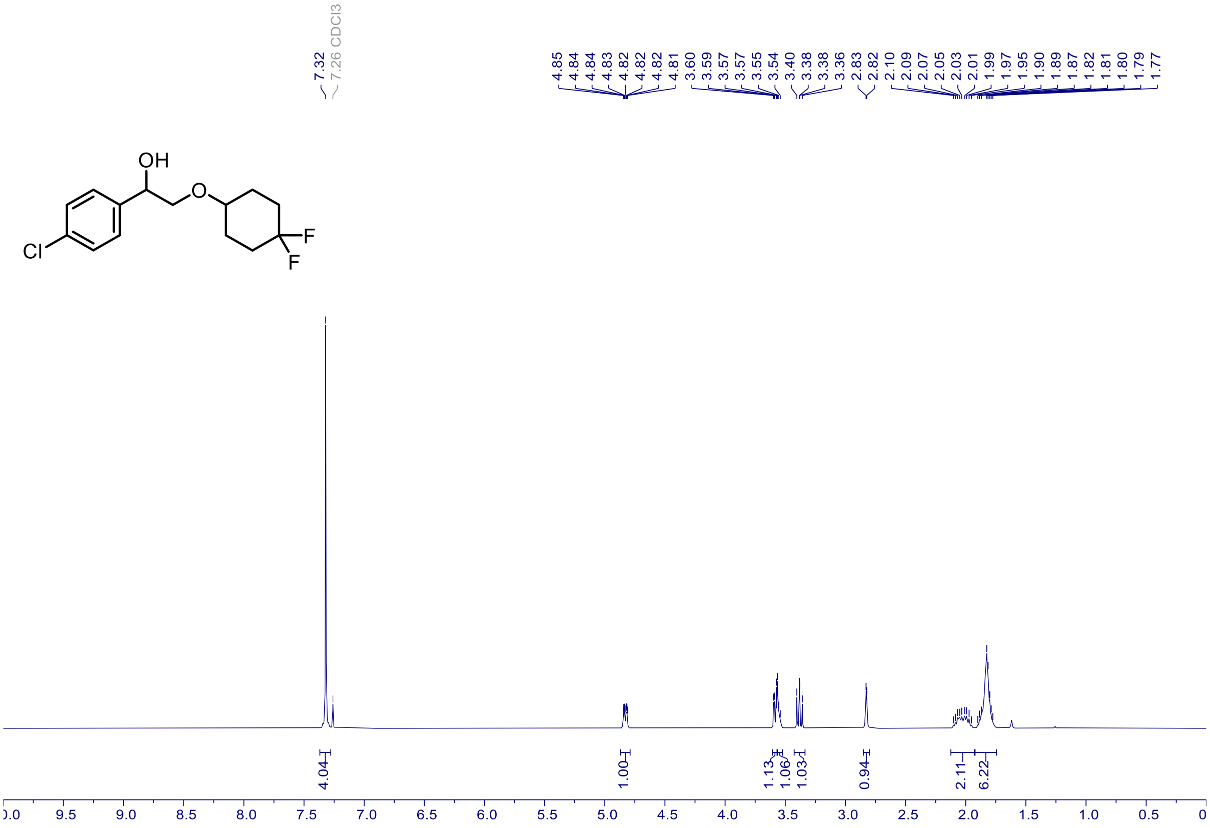

4ch – ^{13}C NMR (101 MHz, $CDCl_3$)

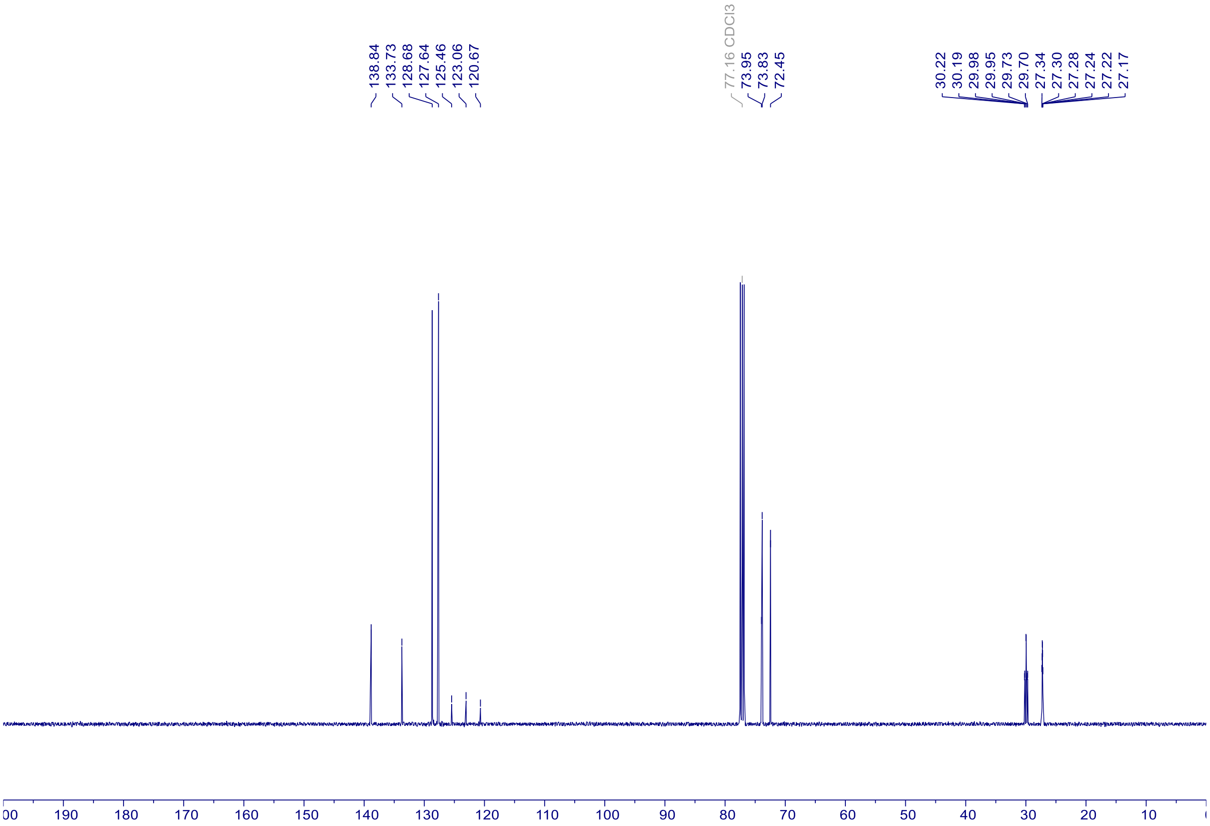

4ch – ^{19}F NMR (376 MHz, $CDCl_3$)

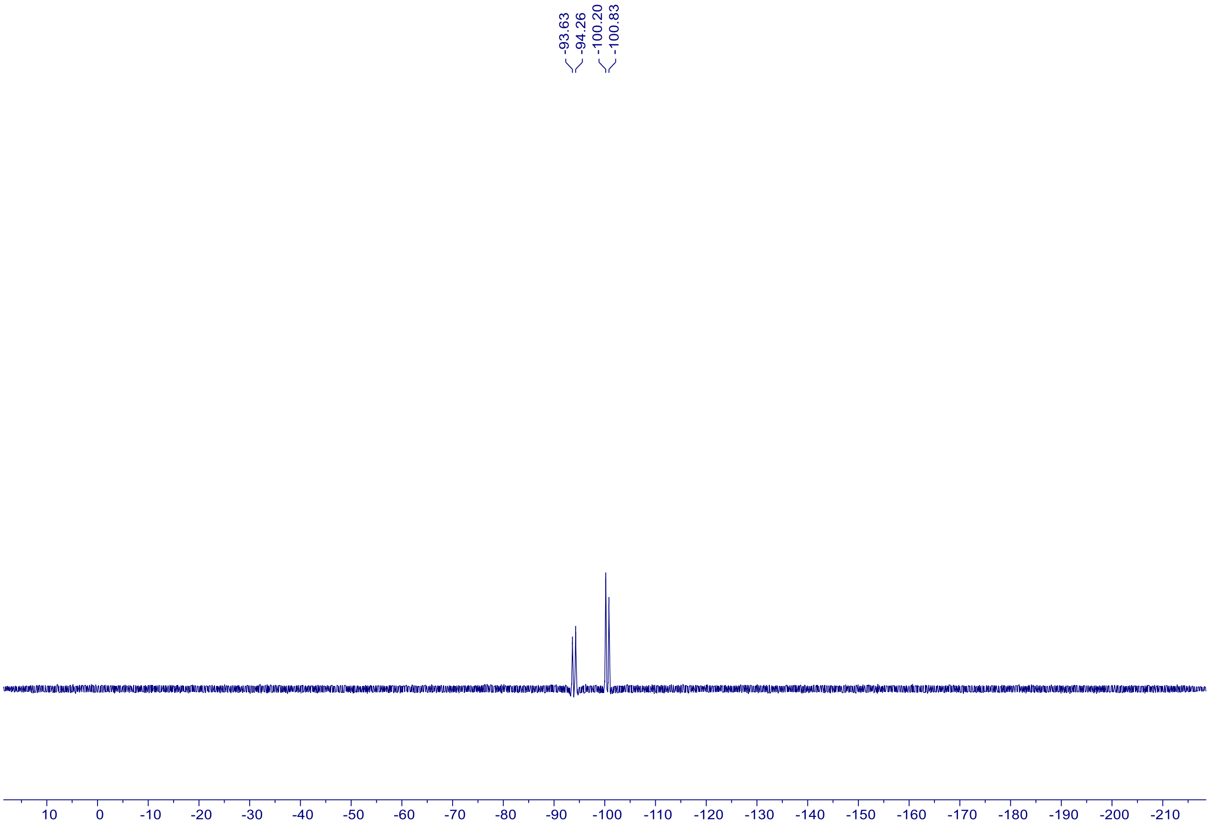

4ci – ^{1}H NMR (400 MHz, CDCl$_3$)

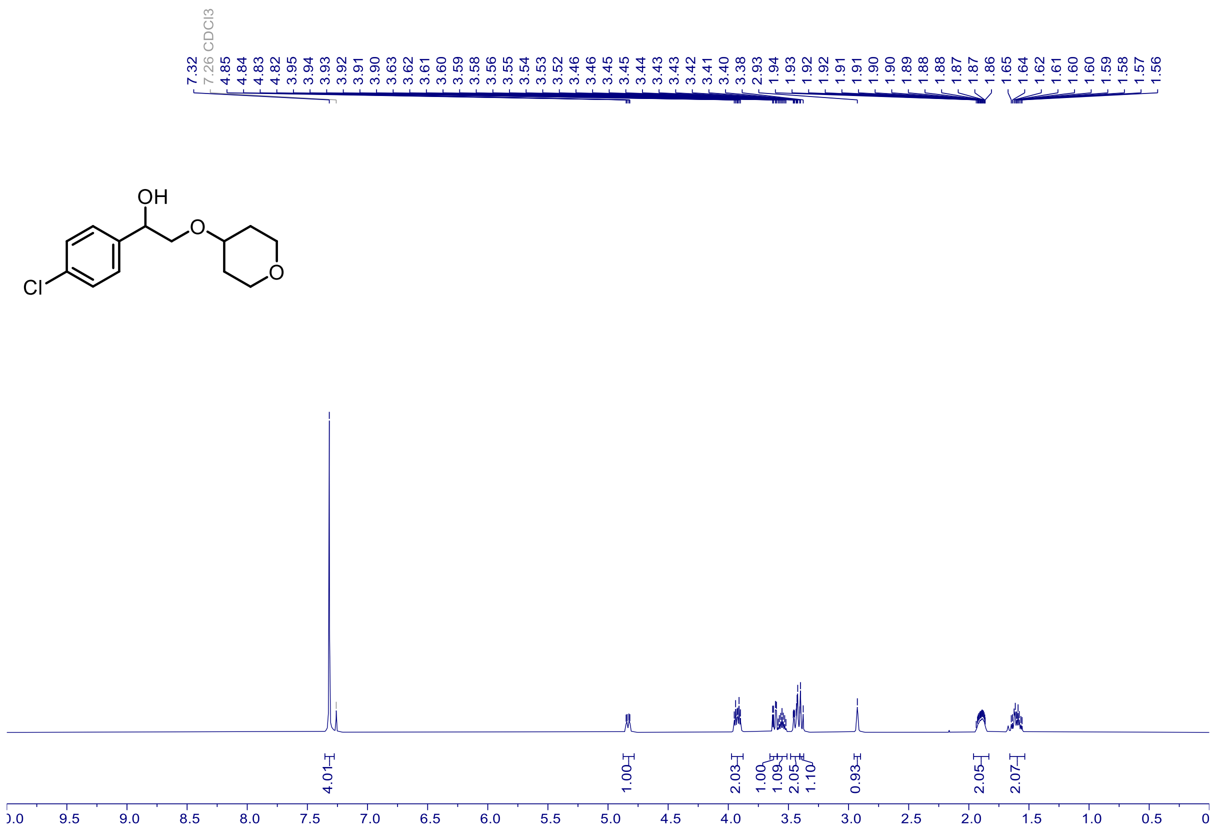

4ci – ^{13}C NMR (101 MHz, CDCl$_3$)

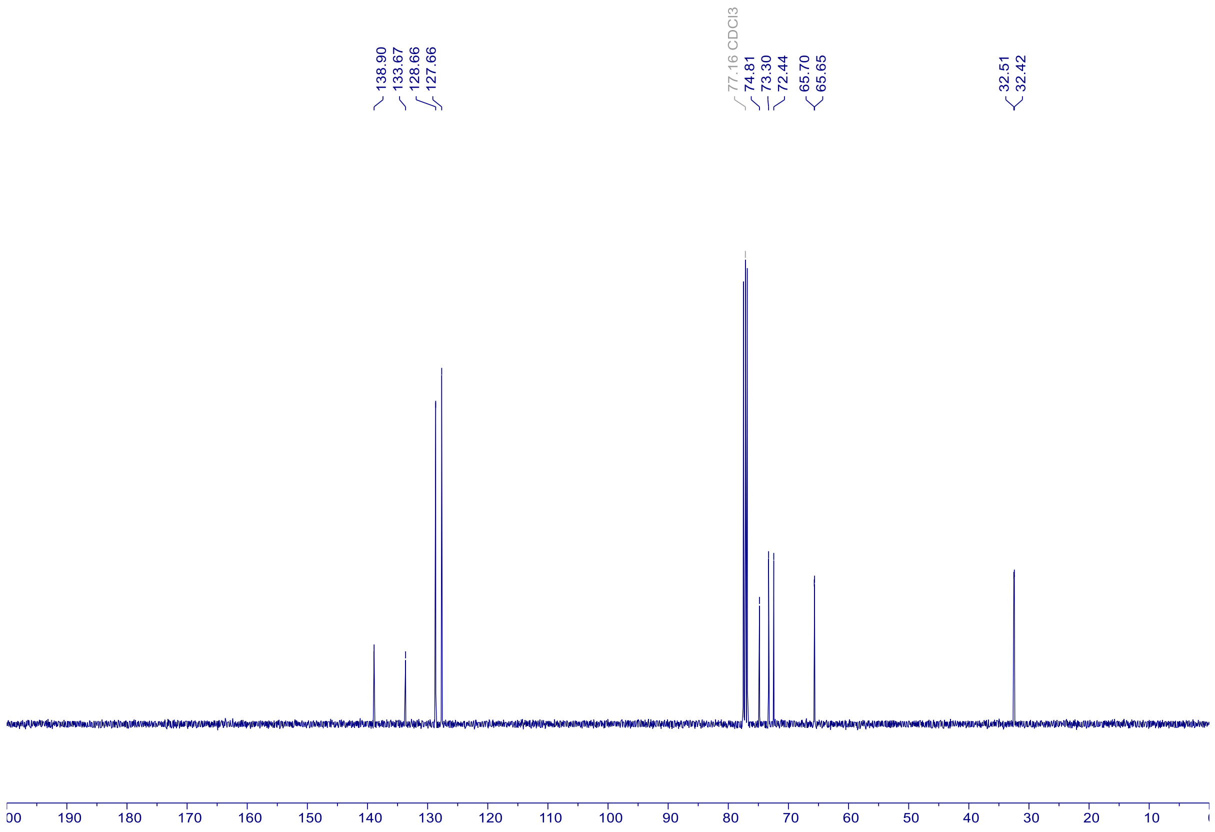

4cj – ^{1}H NMR (400 MHz, $CDCl_3$)

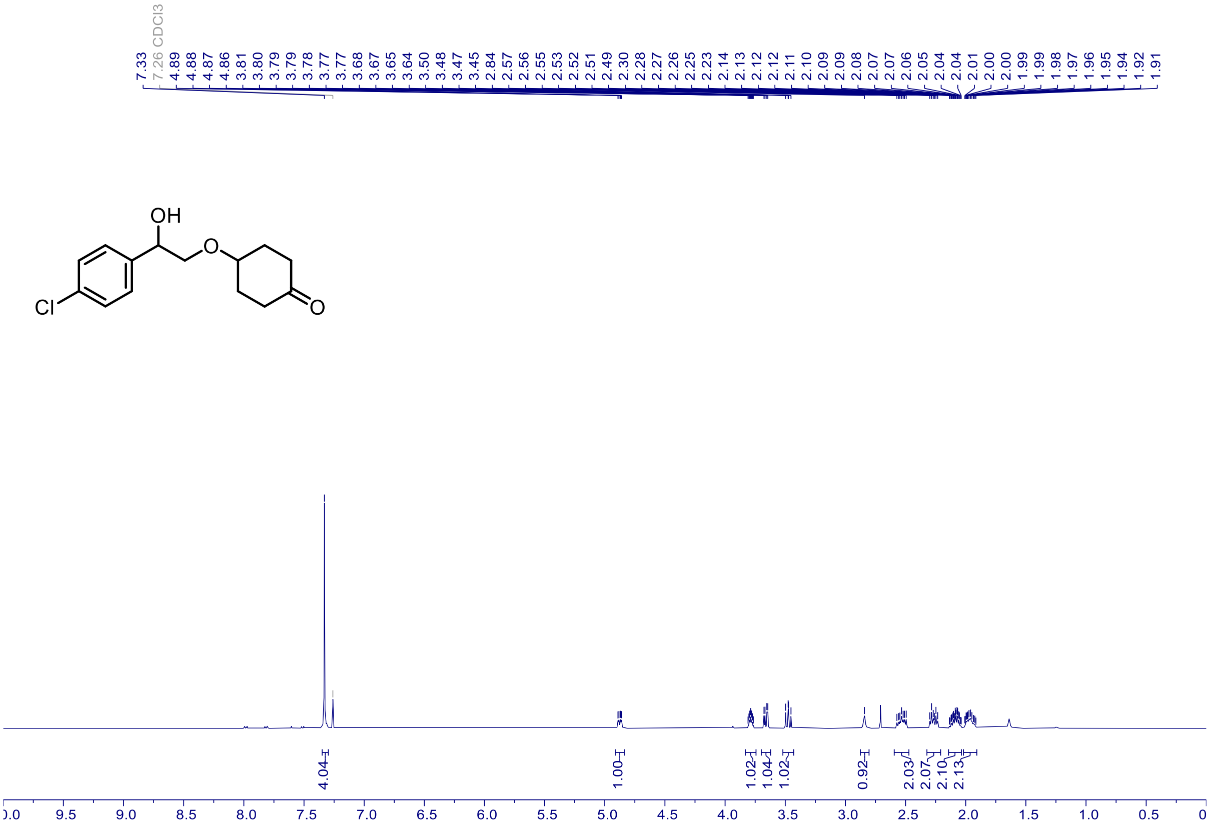

4cj – ^{13}C NMR (101 MHz, $CDCl_3$)

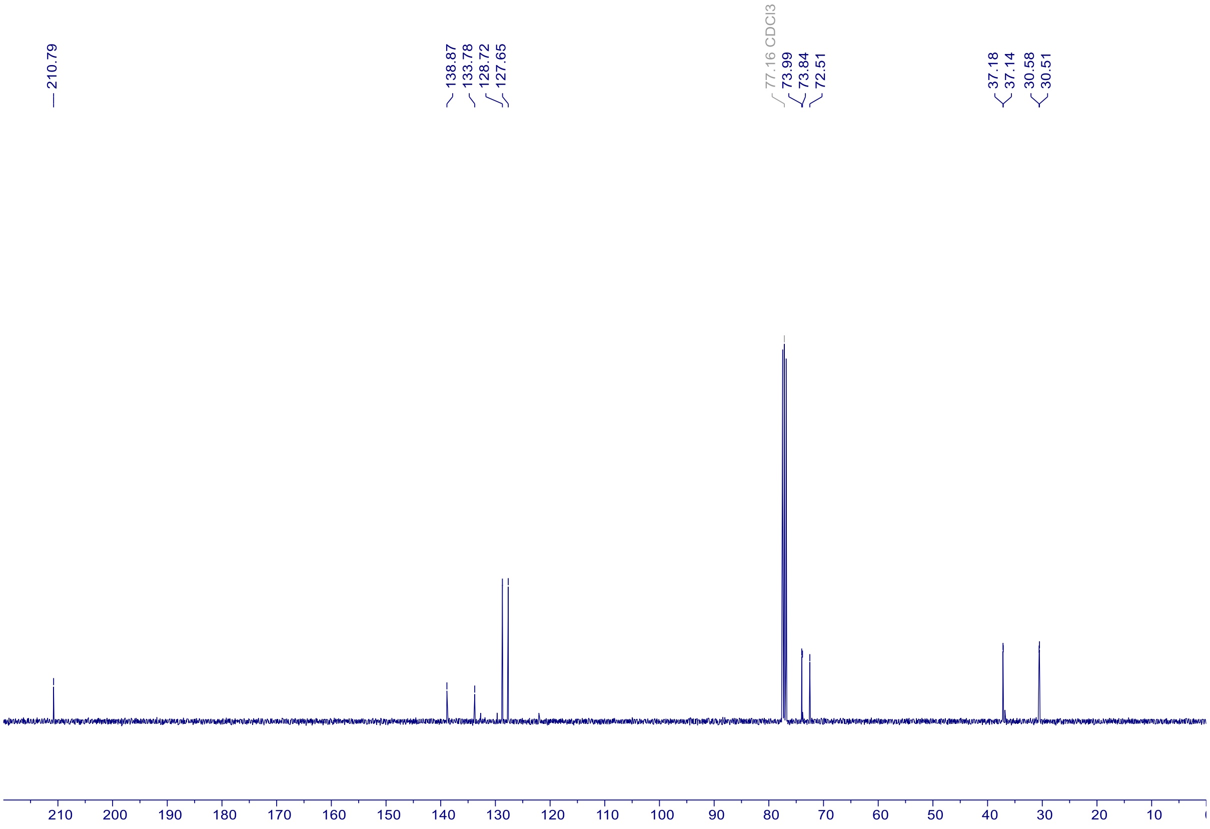

4ck – ^{1}H NMR (400 MHz, $CDCl_3$)

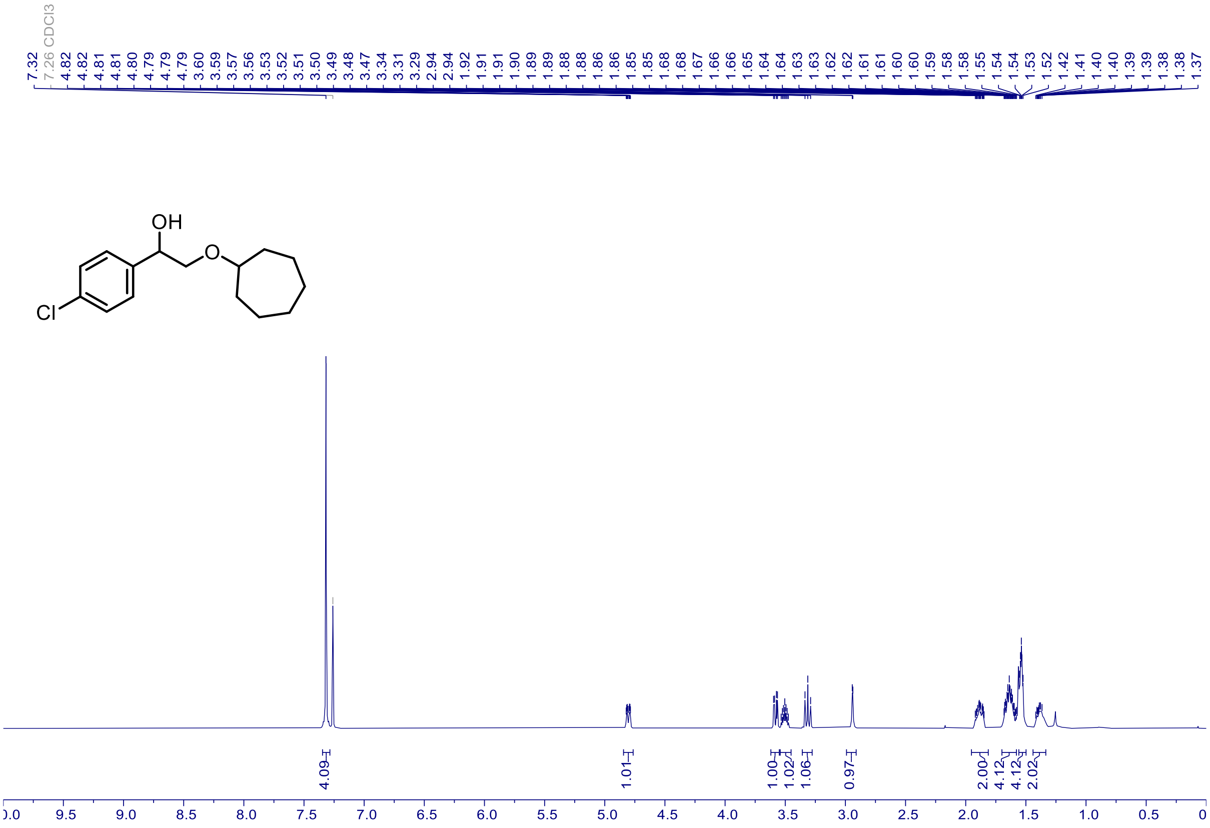

4ck – ^{13}C NMR (101 MHz, $CDCl_3$)

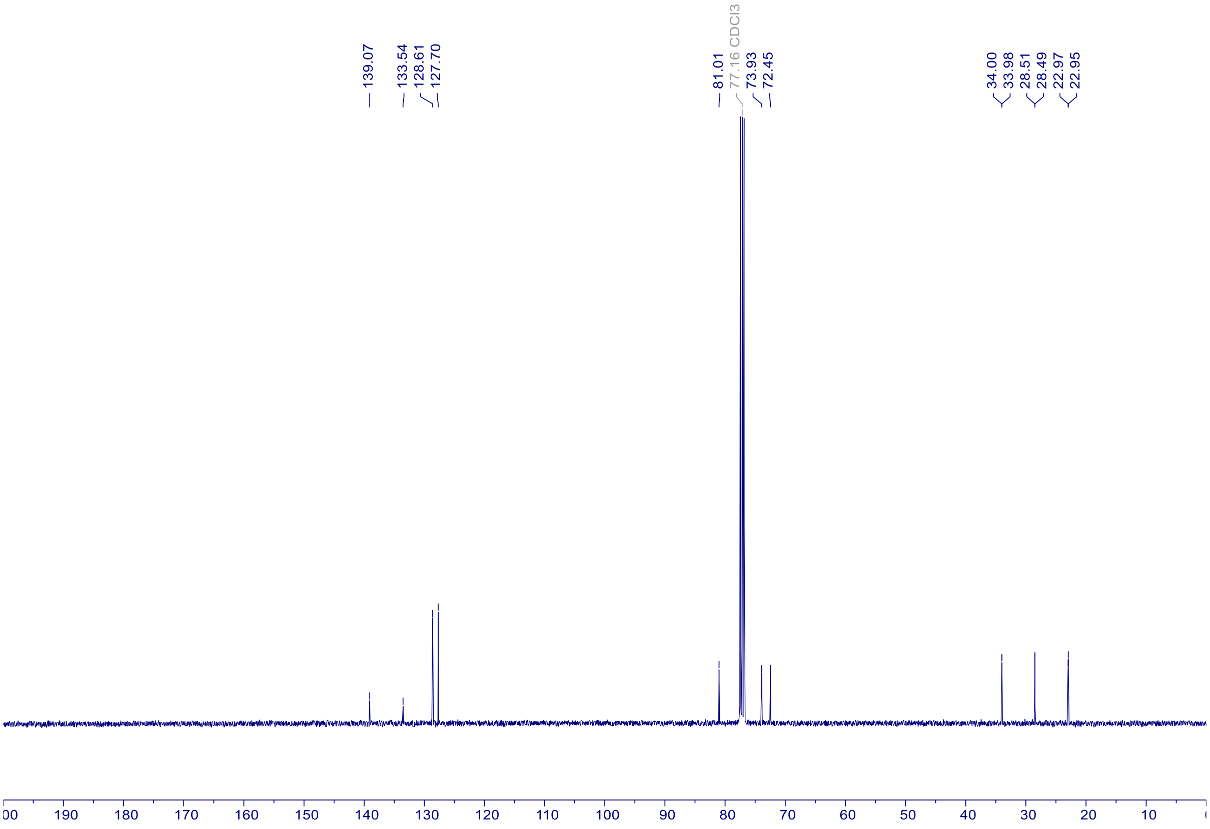

4cl – ^{1}H NMR (400 MHz, $CDCl_3$)

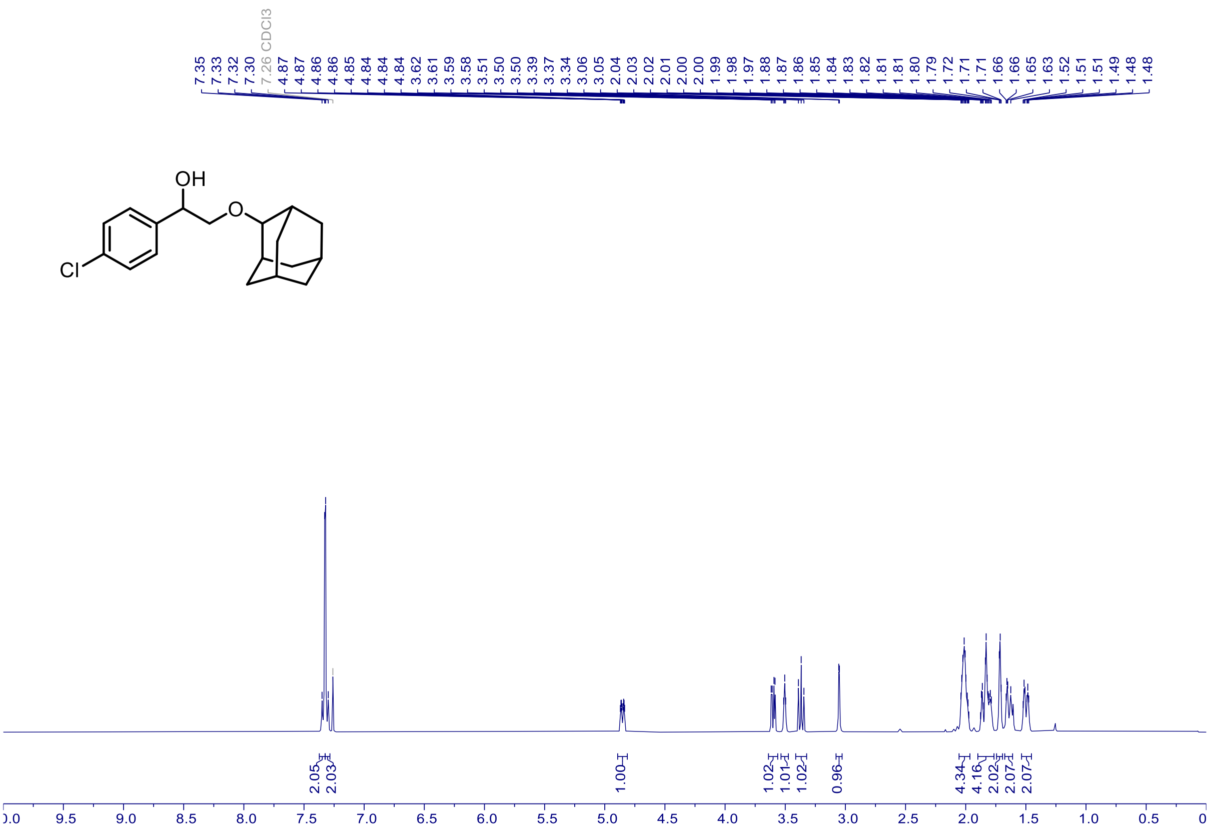

4cl – ^{13}C NMR (101 MHz, $CDCl_3$)

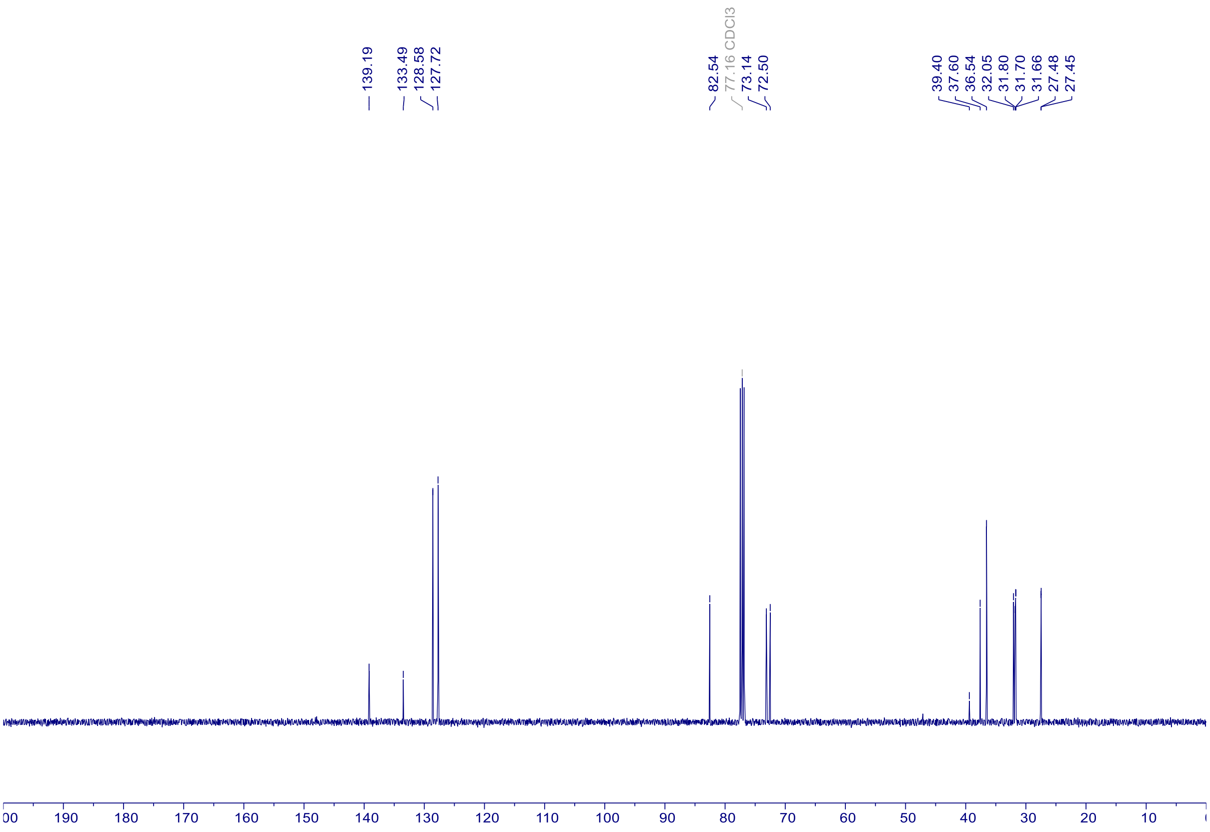

4cm – ^{1}H NMR (400 MHz, $CDCl_3$)

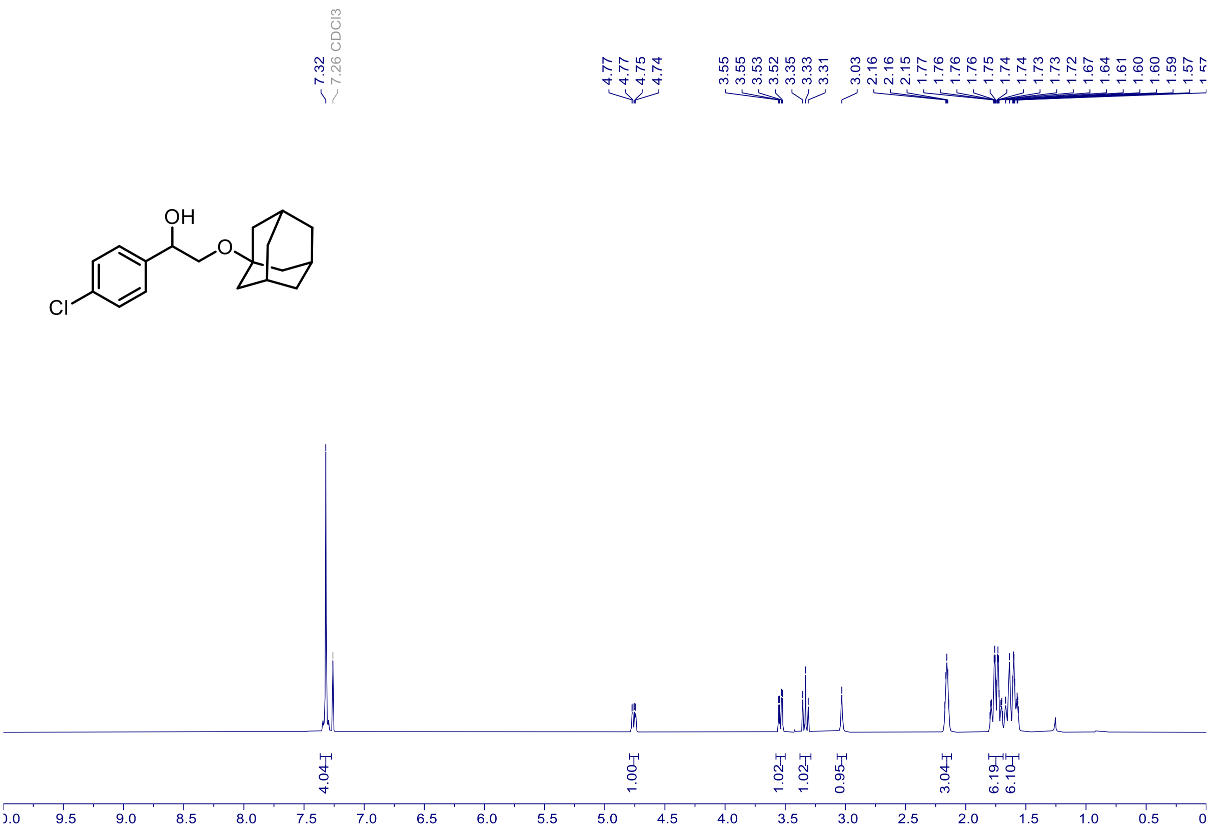

4cm – ^{13}C NMR (101 MHz, $CDCl_3$)

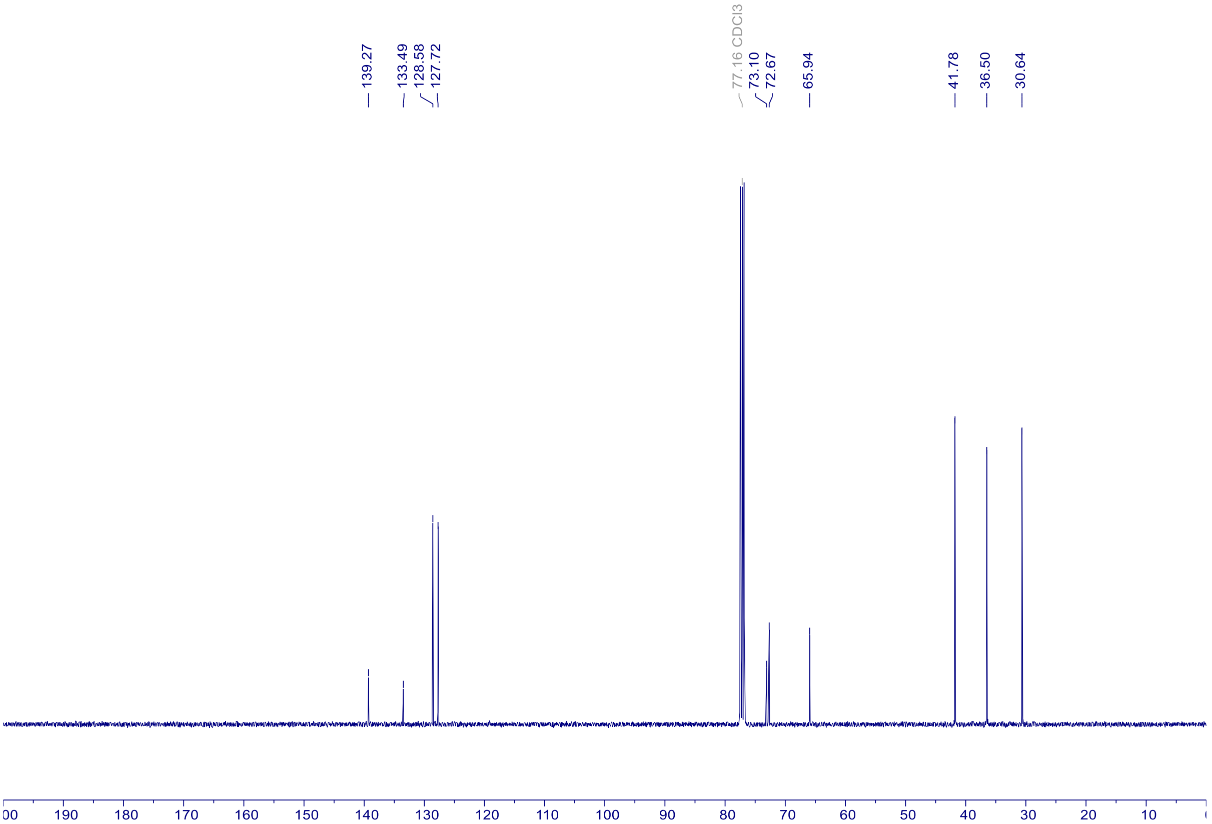

5a – ^{1}H NMR (400 MHz, $CDCl_3$)

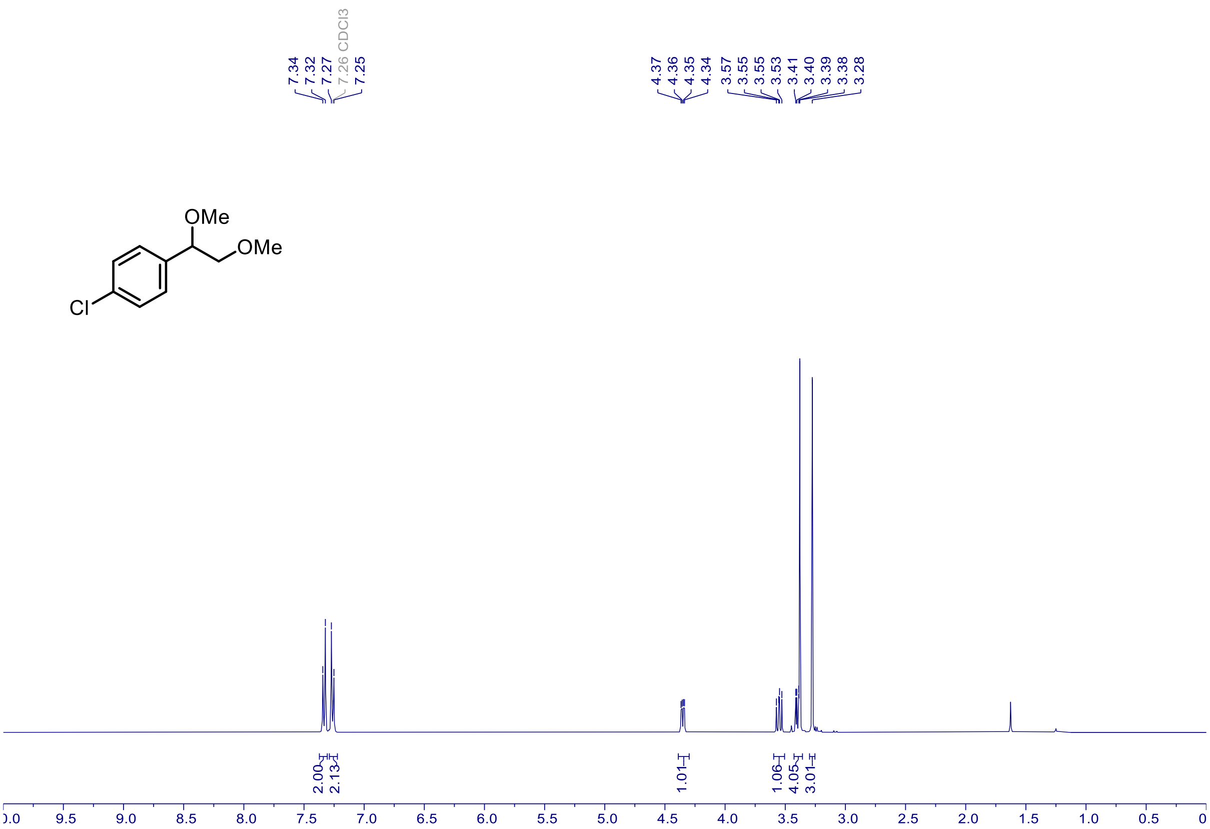

5a – ^{13}C NMR (101 MHz, $CDCl_3$)

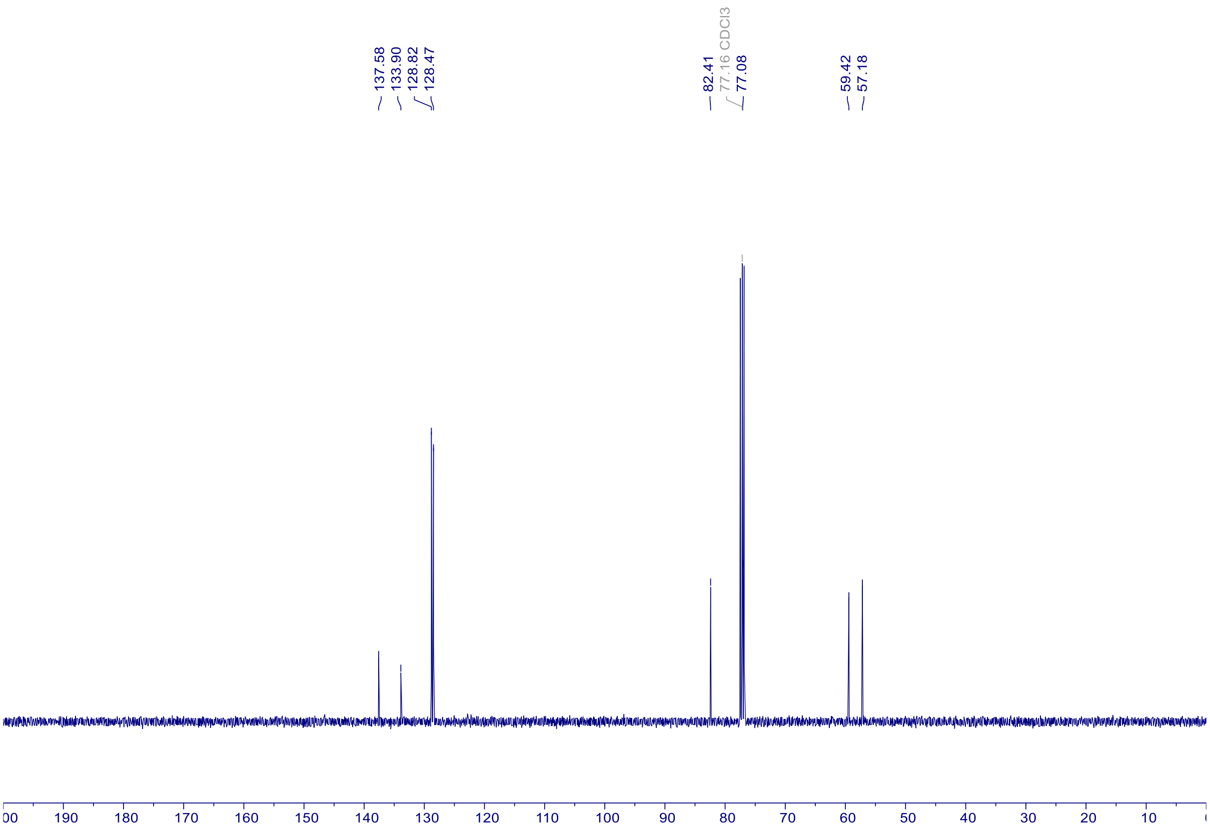

5b – ^{1}H NMR (400 MHz, CDCl$_3$)

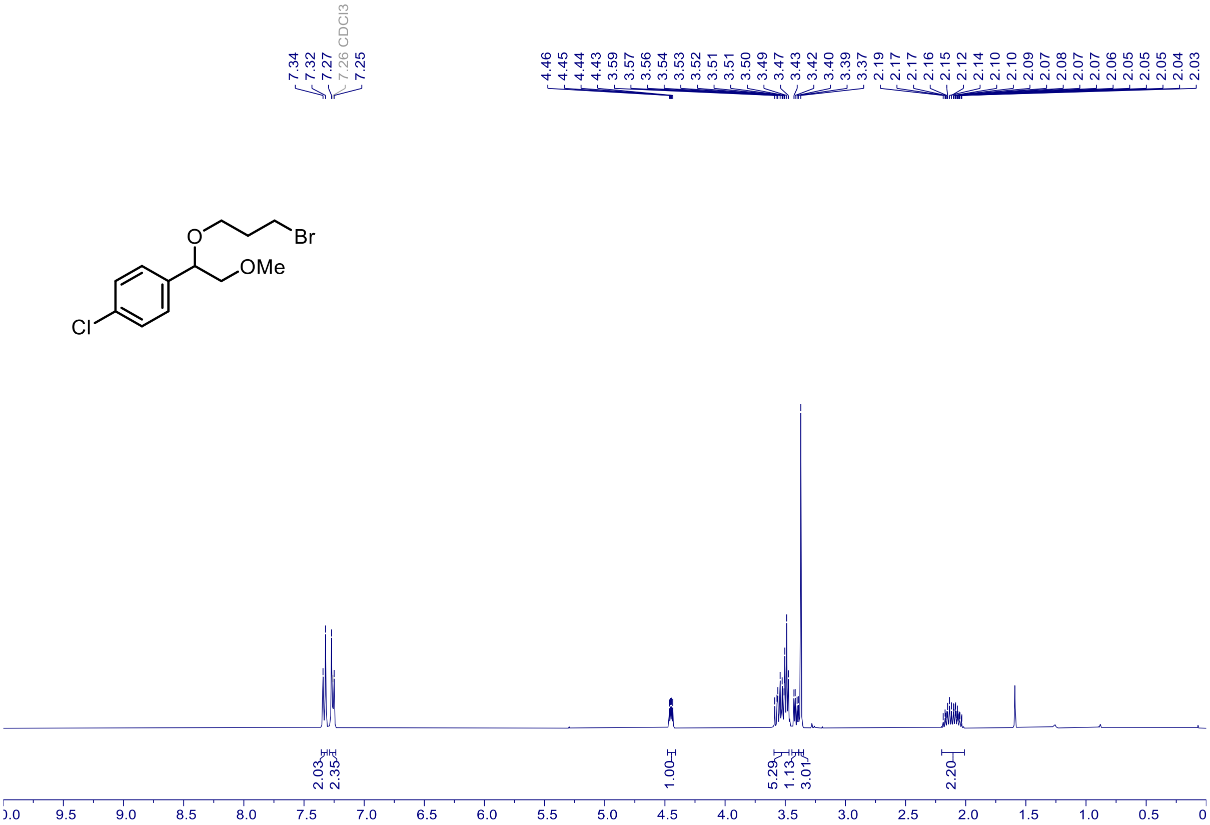

5b – ^{13}C NMR (101 MHz, CDCl$_3$)

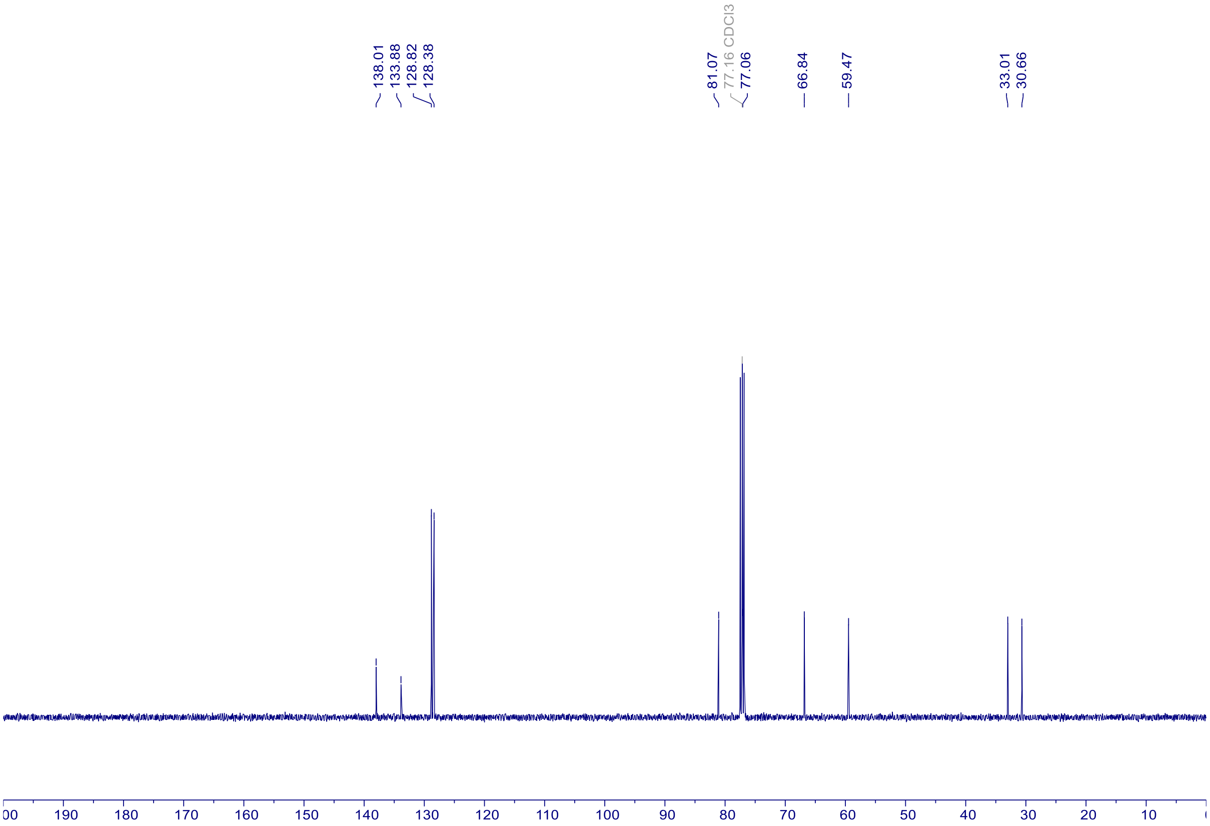

5c – ^{1}H NMR (400 MHz, $CDCl_3$)

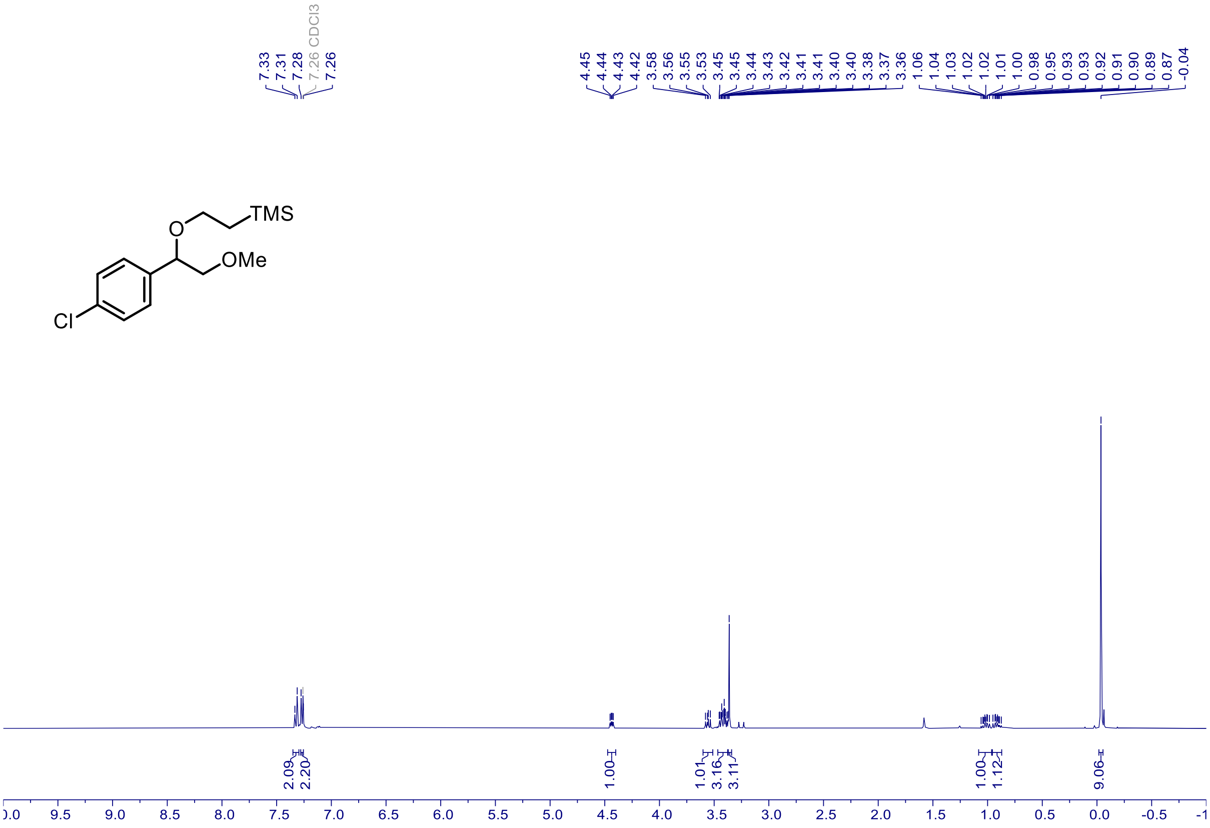

5c – ^{13}C NMR (101 MHz, $CDCl_3$)

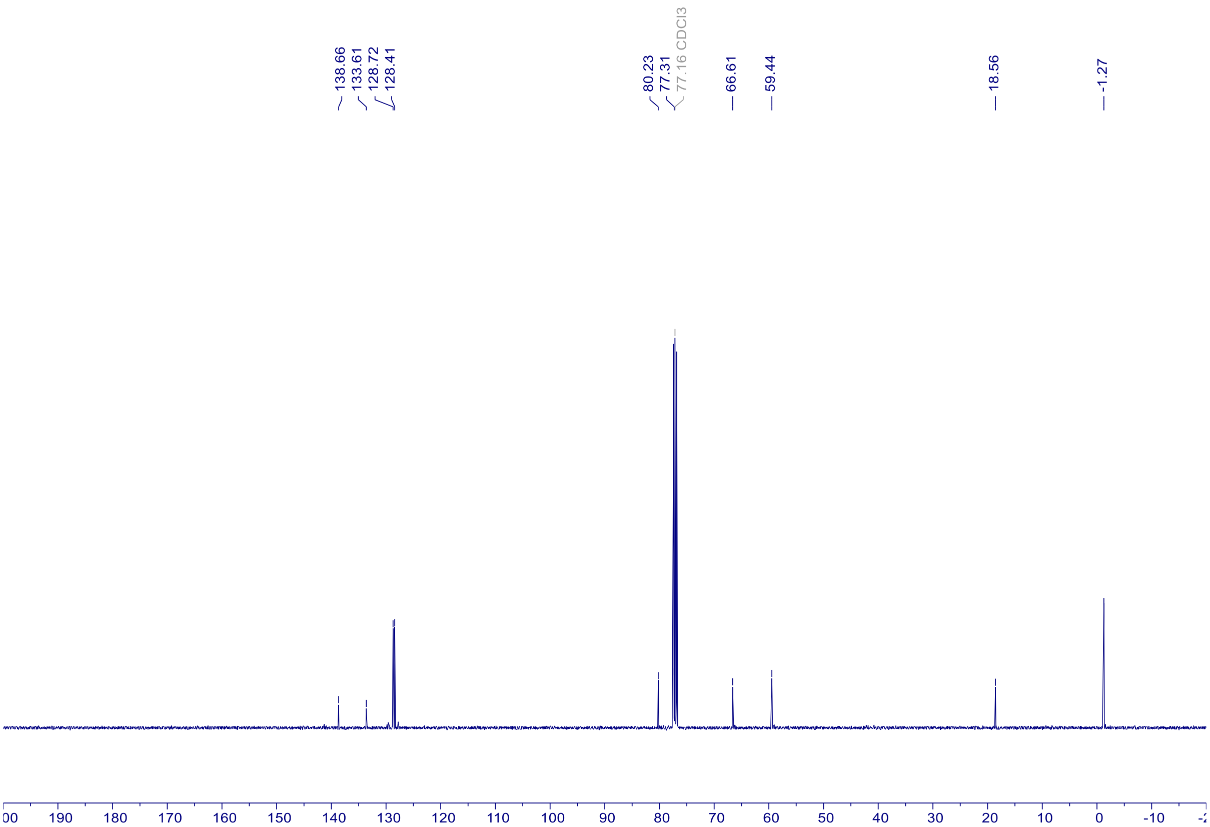

5d – ^{1}H NMR (400 MHz, CDCl$_3$)

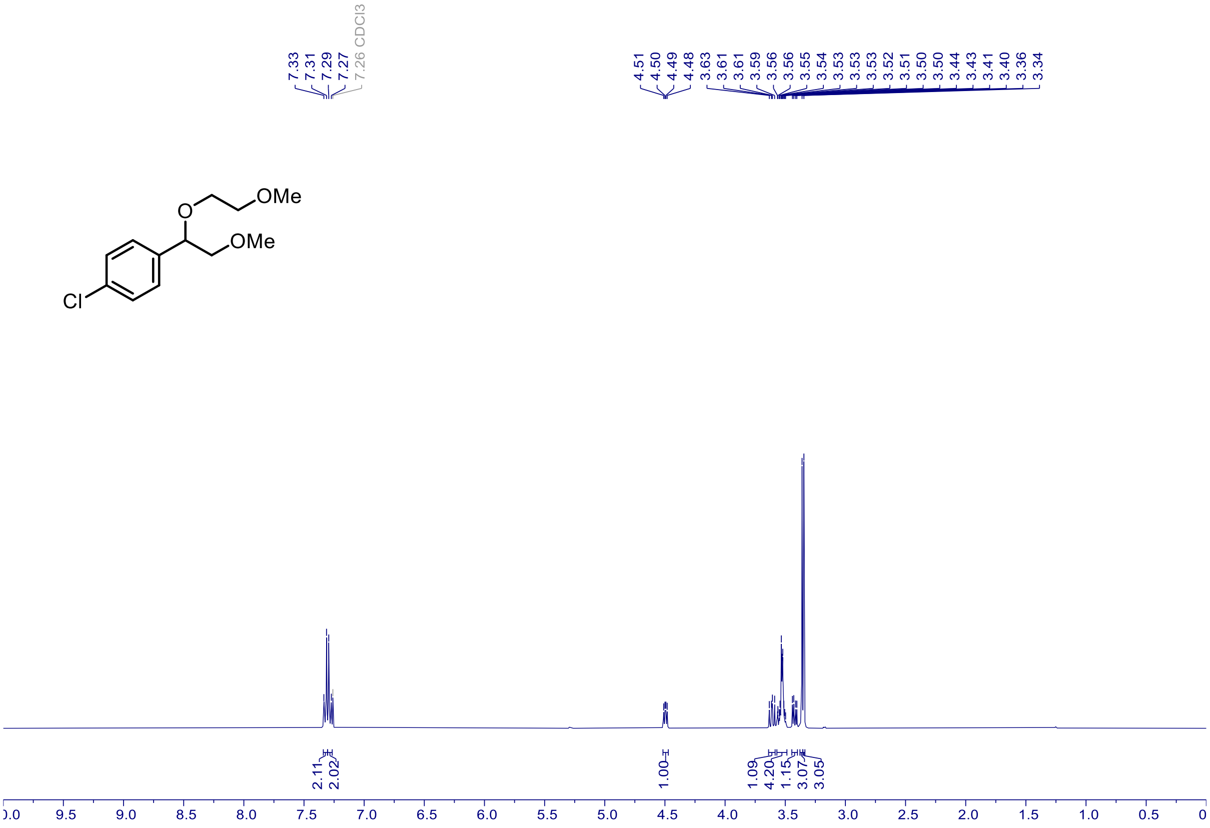

5d – ^{13}C NMR (101 MHz, CDCl$_3$)

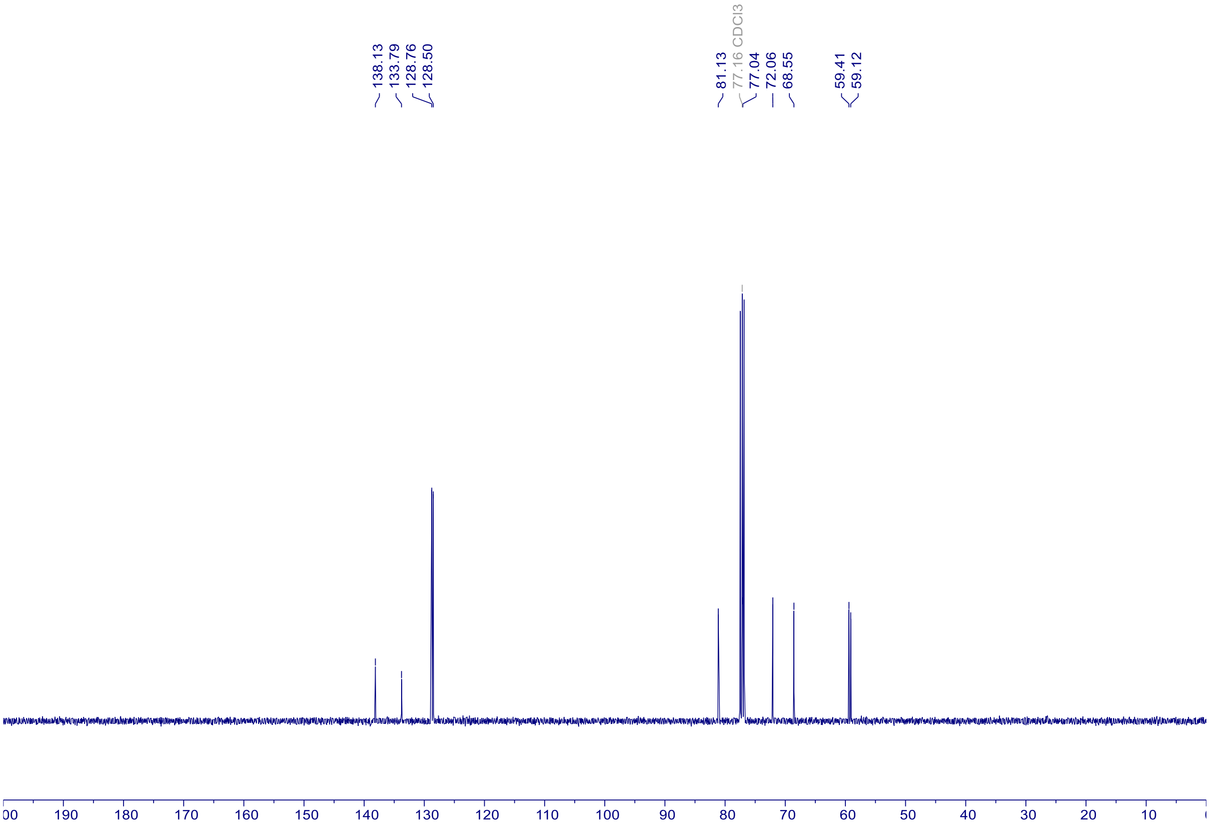

5e – ^{1}H NMR (400 MHz, $CDCl_3$)

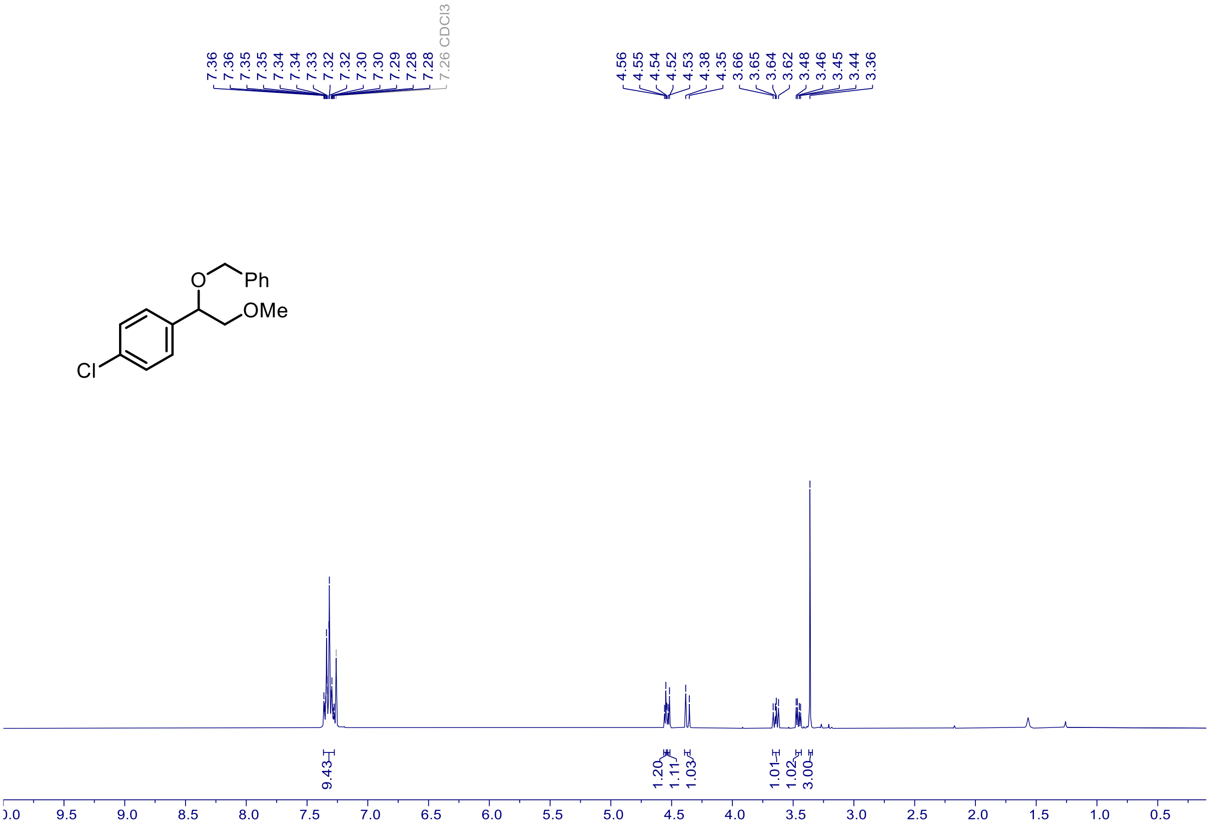

5e – ^{13}C NMR (101 MHz, $CDCl_3$)

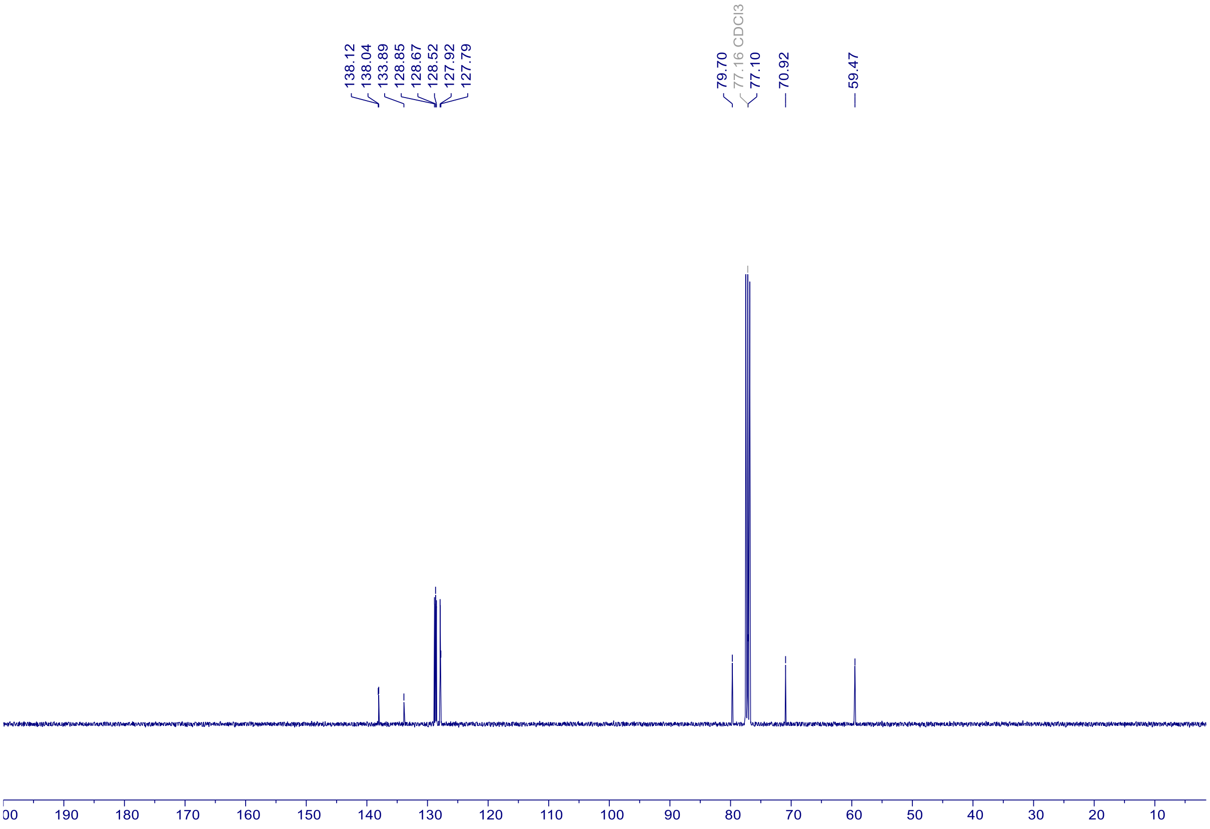

5f – ^{1}H NMR (400 MHz, $CDCl_3$, 1:1 mixture of diastereoisomers)

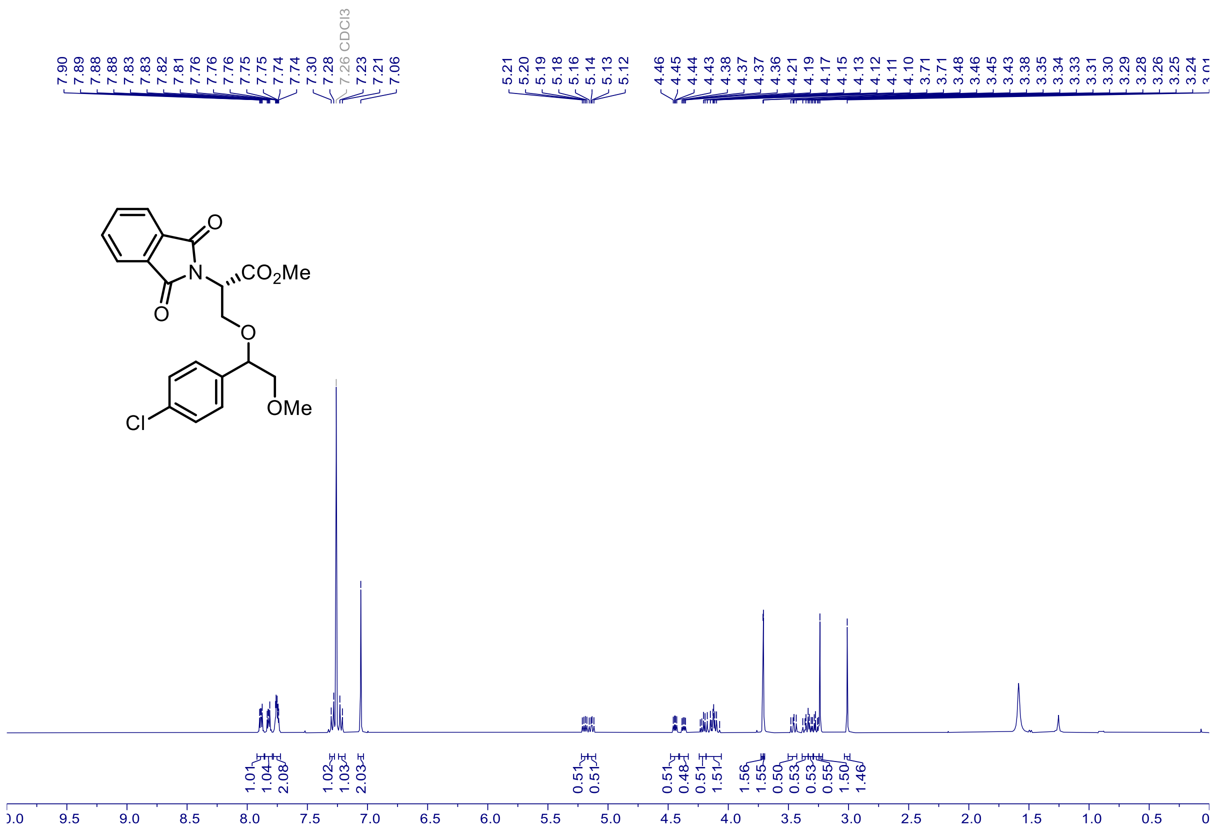

5f – ^{13}C NMR (101 MHz, $CDCl_3$, 1:1 mixture of diastereoisomers)

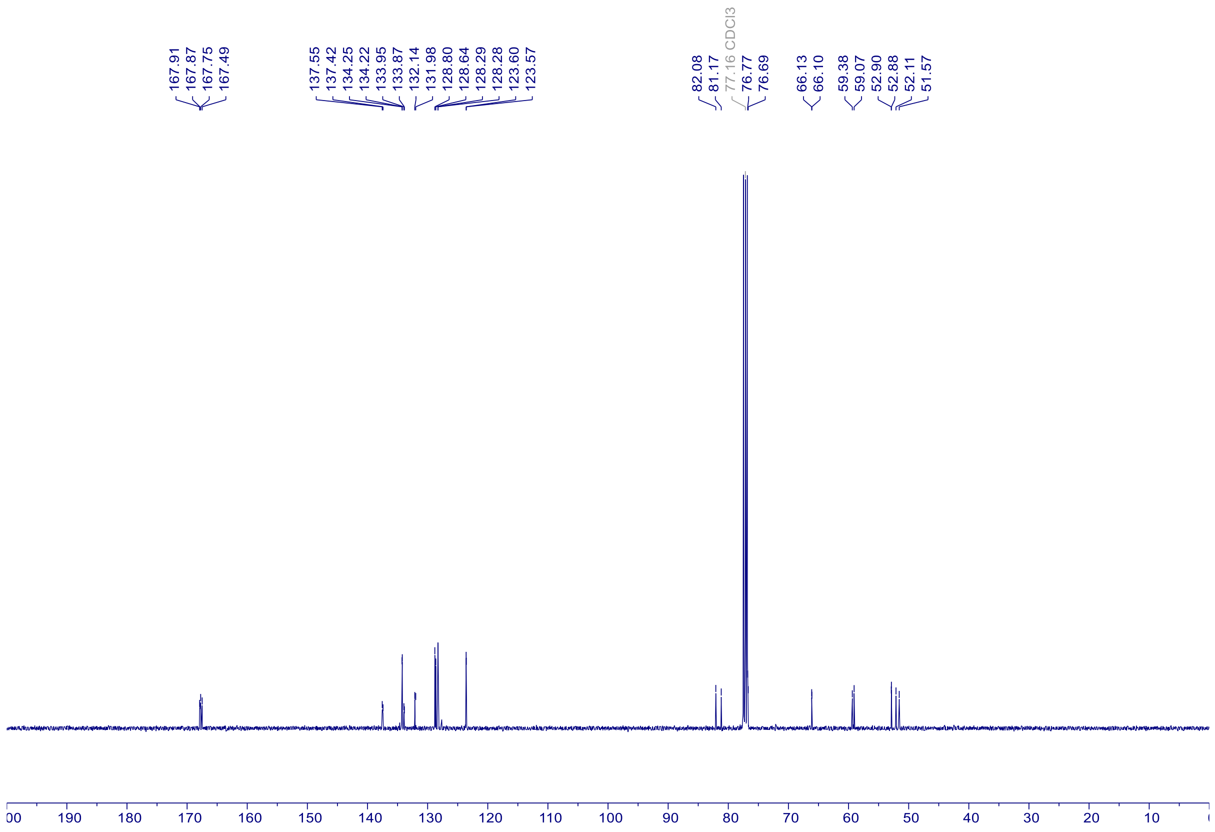

5g – ^{1}H NMR (400 MHz, $CDCl_3$)

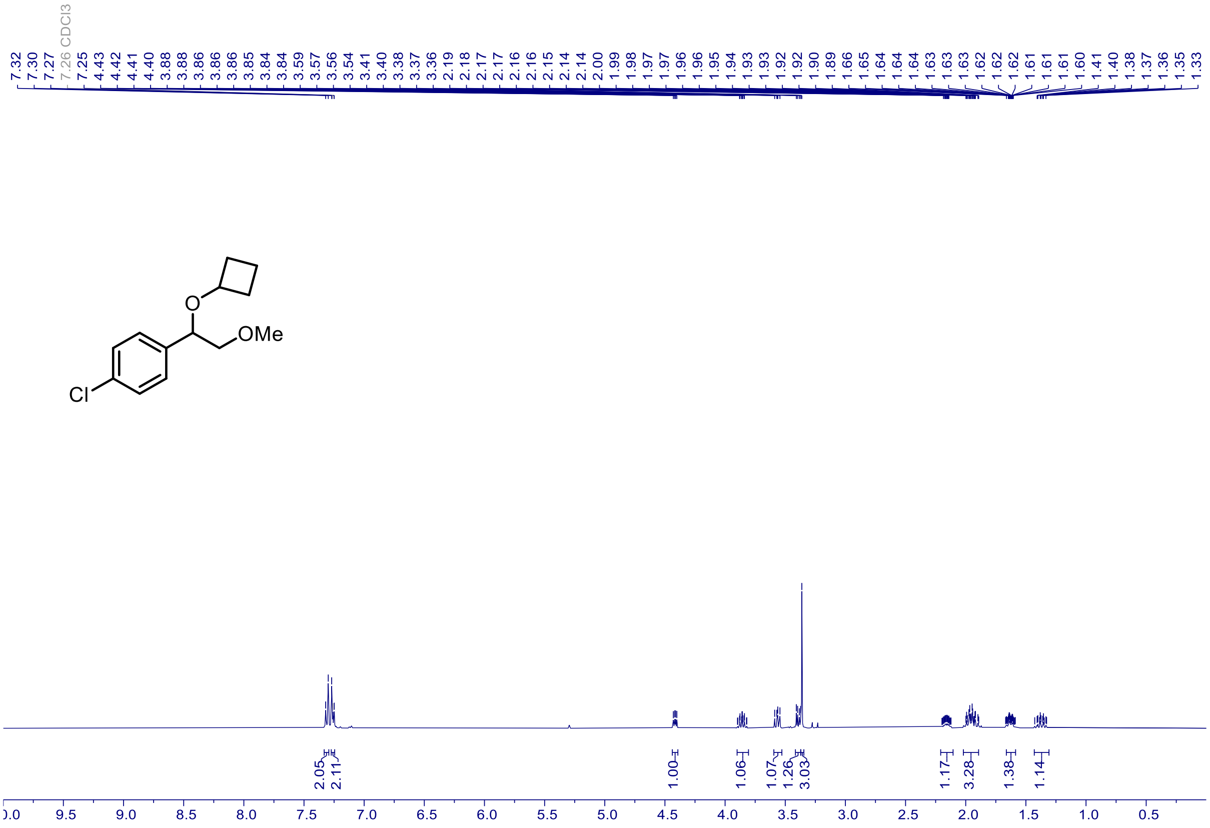

5g – ^{13}C NMR (101 MHz, $CDCl_3$)

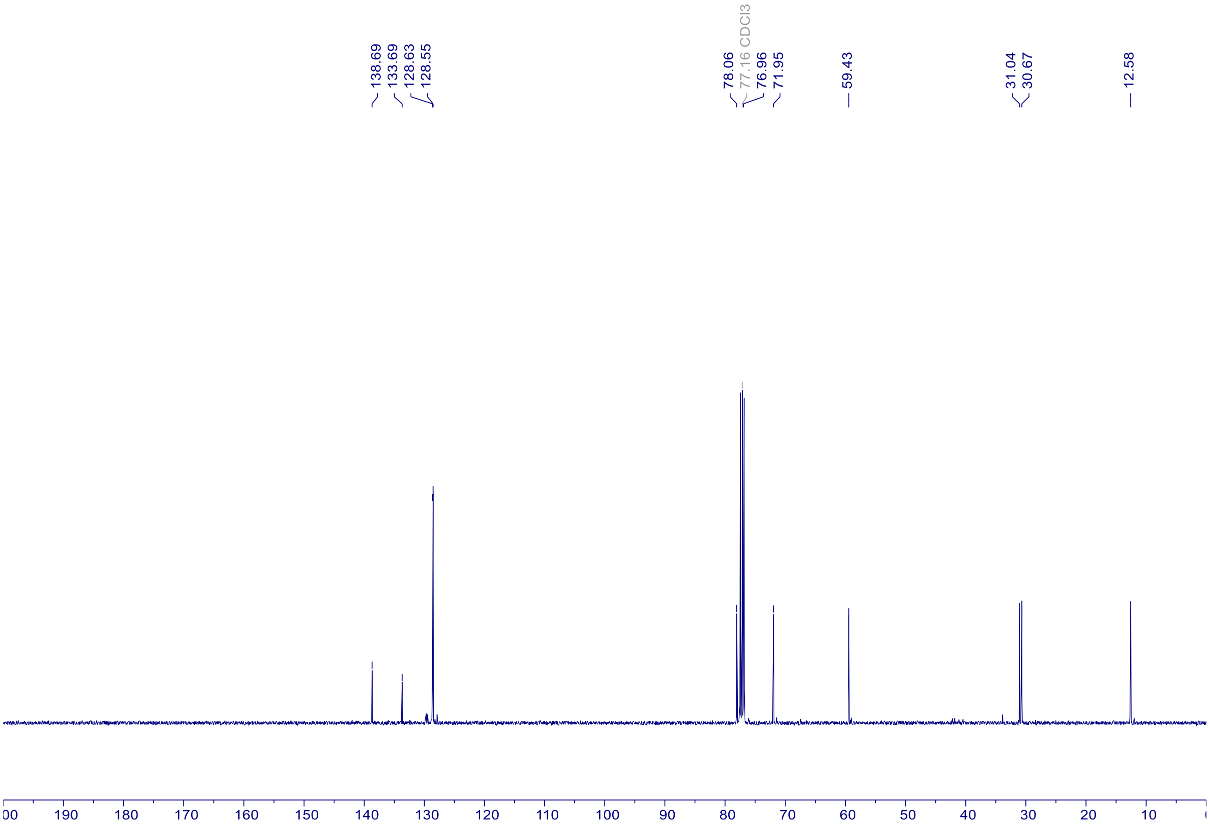

5h – ^{1}H NMR (400 MHz, CDCl$_3$)

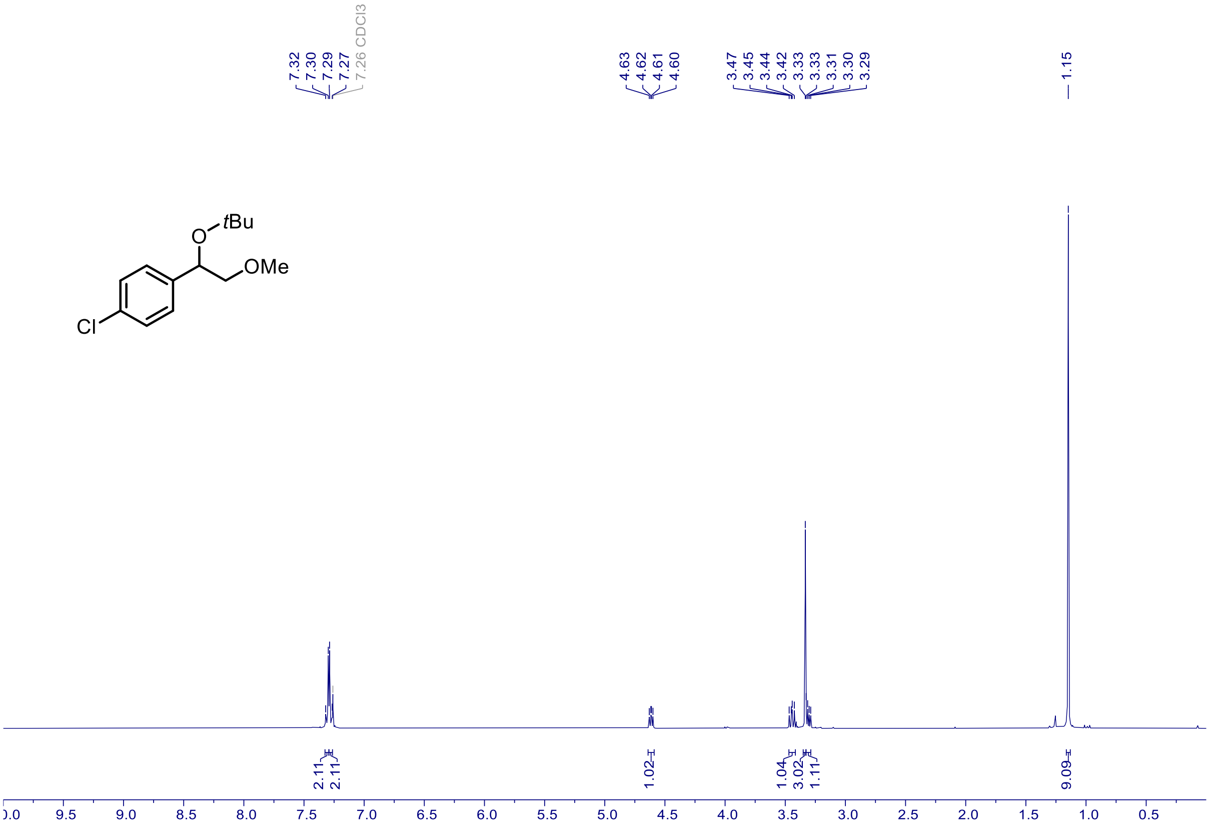

5h – ^{13}C NMR (101 MHz, CDCl$_3$)

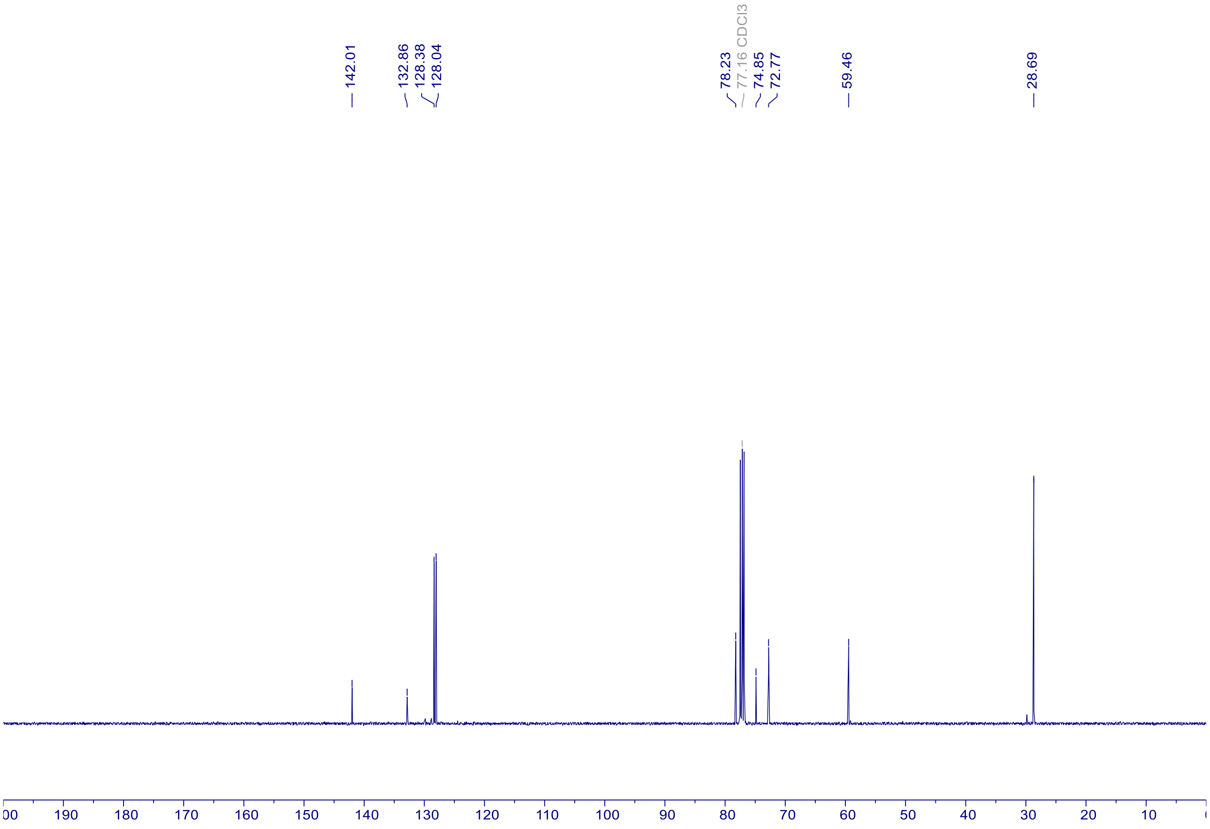

6a – ^{1}H NMR (500 MHz, $CDCl_3$)

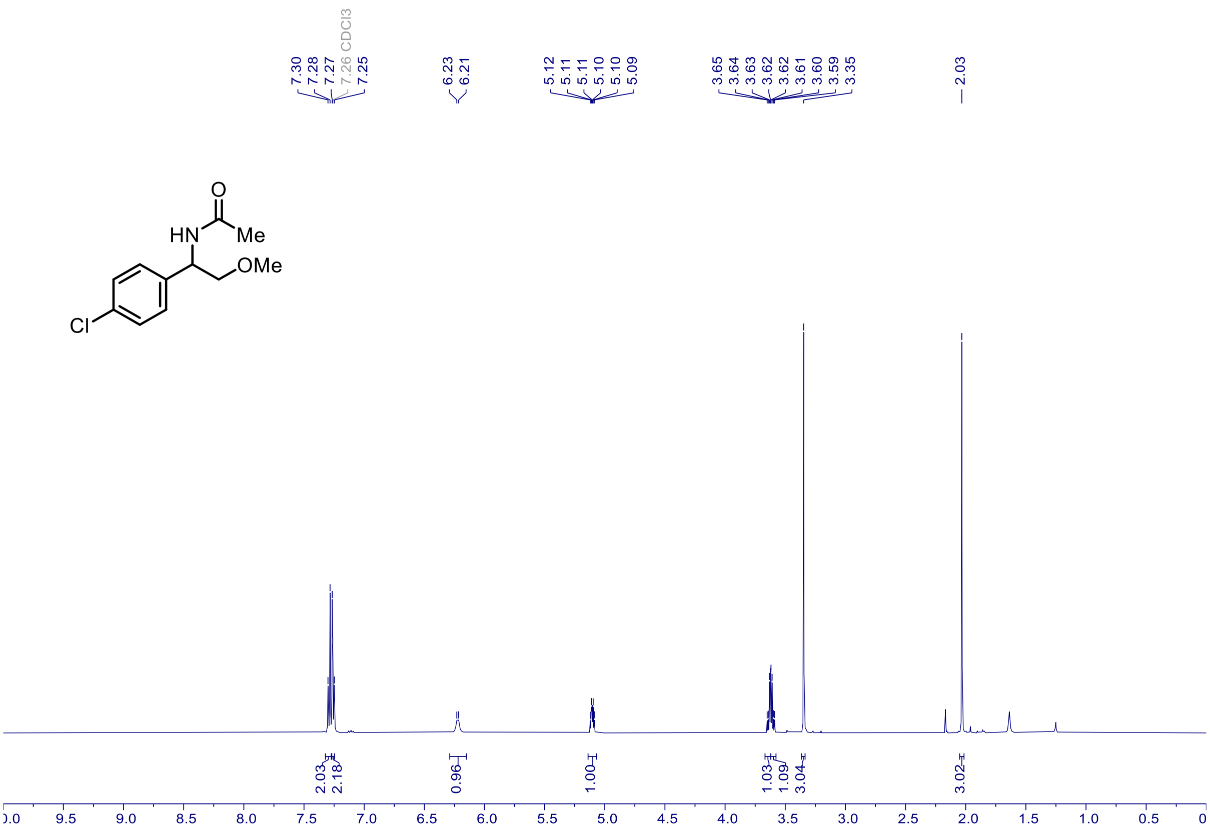

6a – ^{13}C NMR (126 MHz, $CDCl_3$)

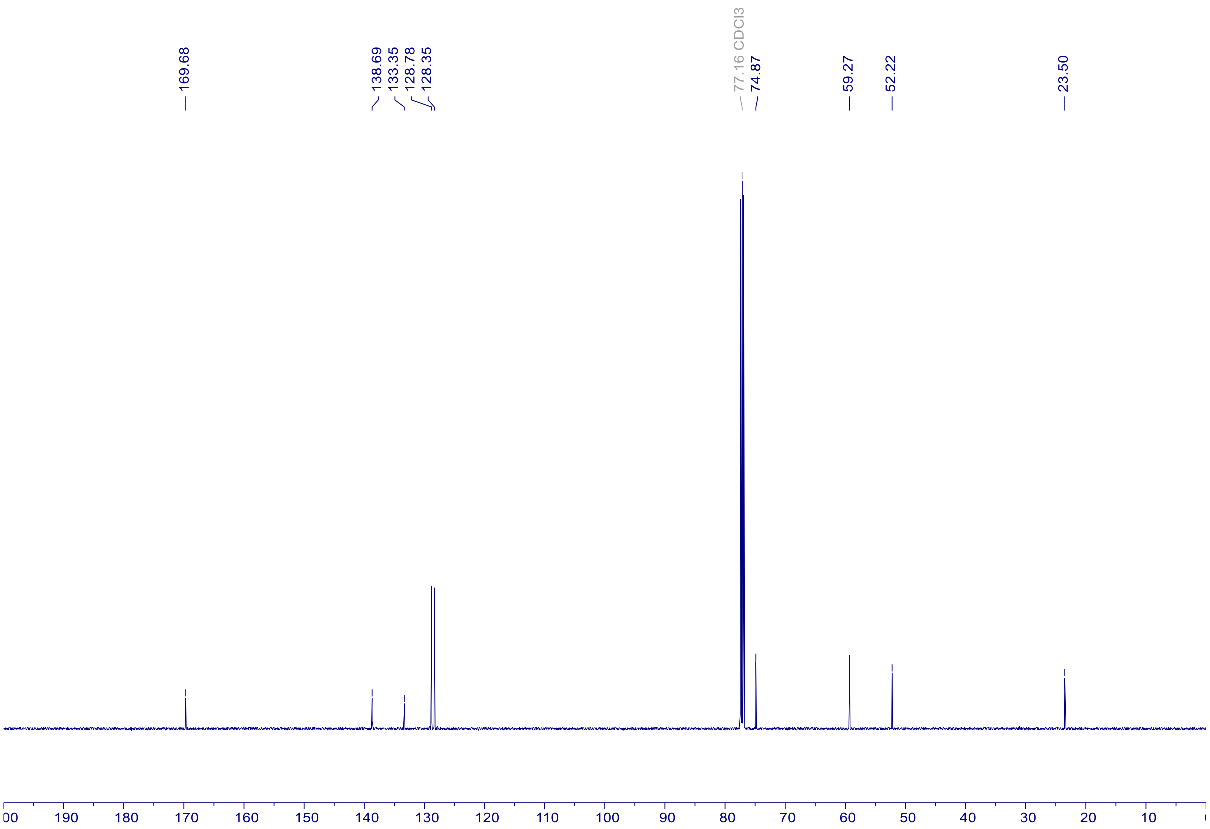

6b – ^{1}H NMR (400 MHz, $CDCl_3$)

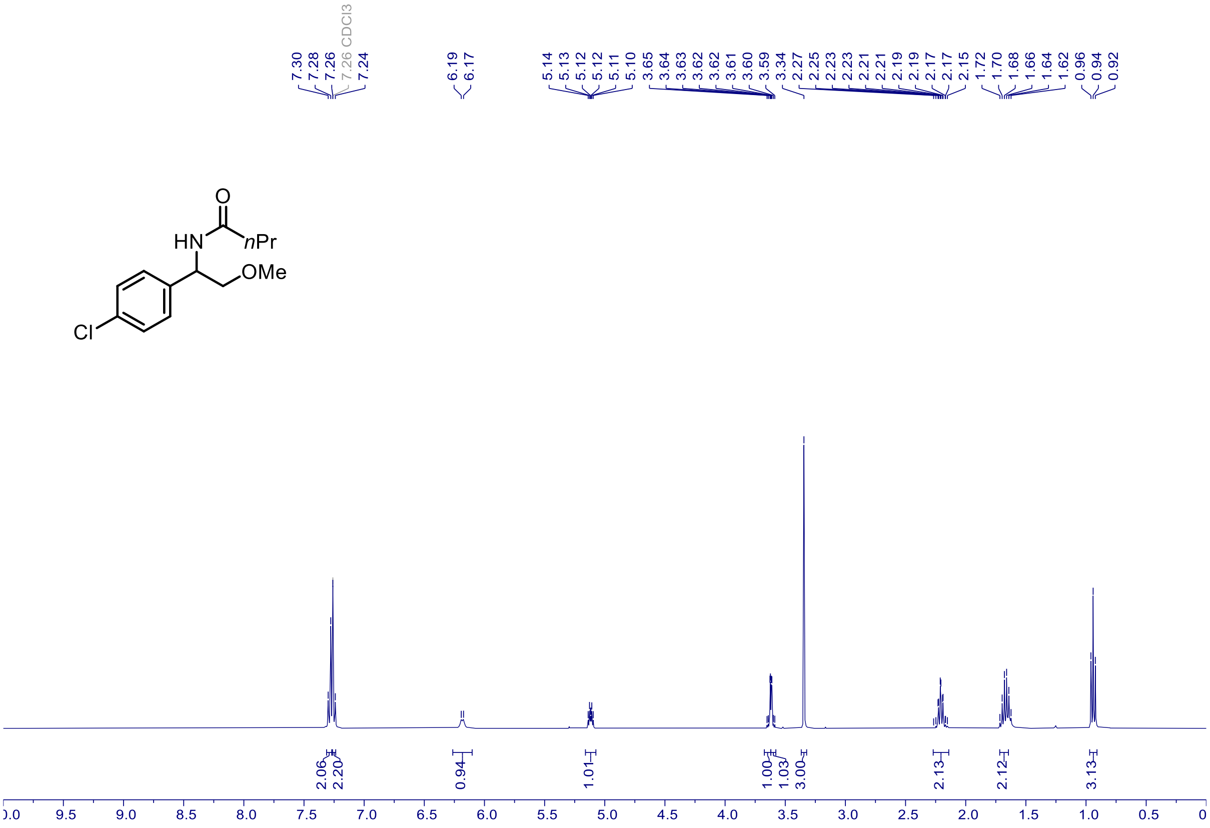

6b – ^{13}C NMR (101 MHz, $CDCl_3$)

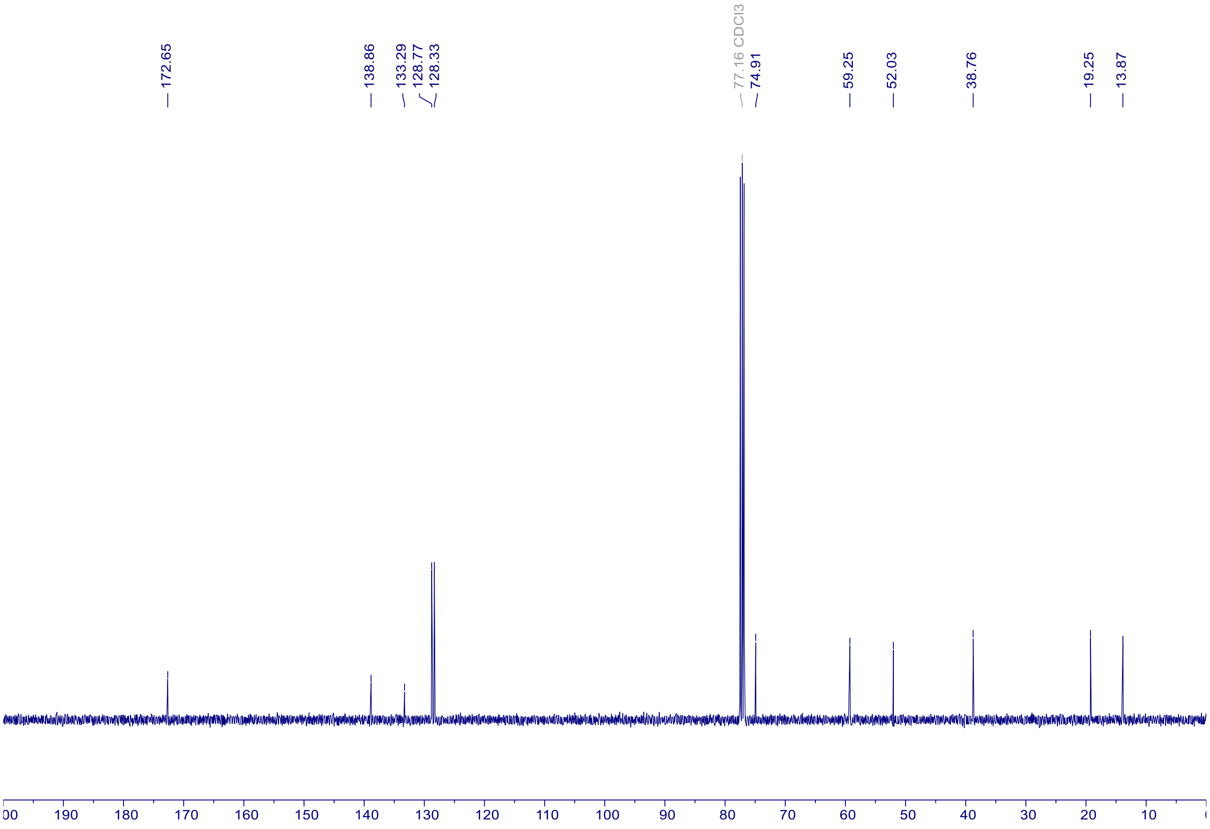

6c – ^{1}H NMR (400 MHz, $CDCl_3$)

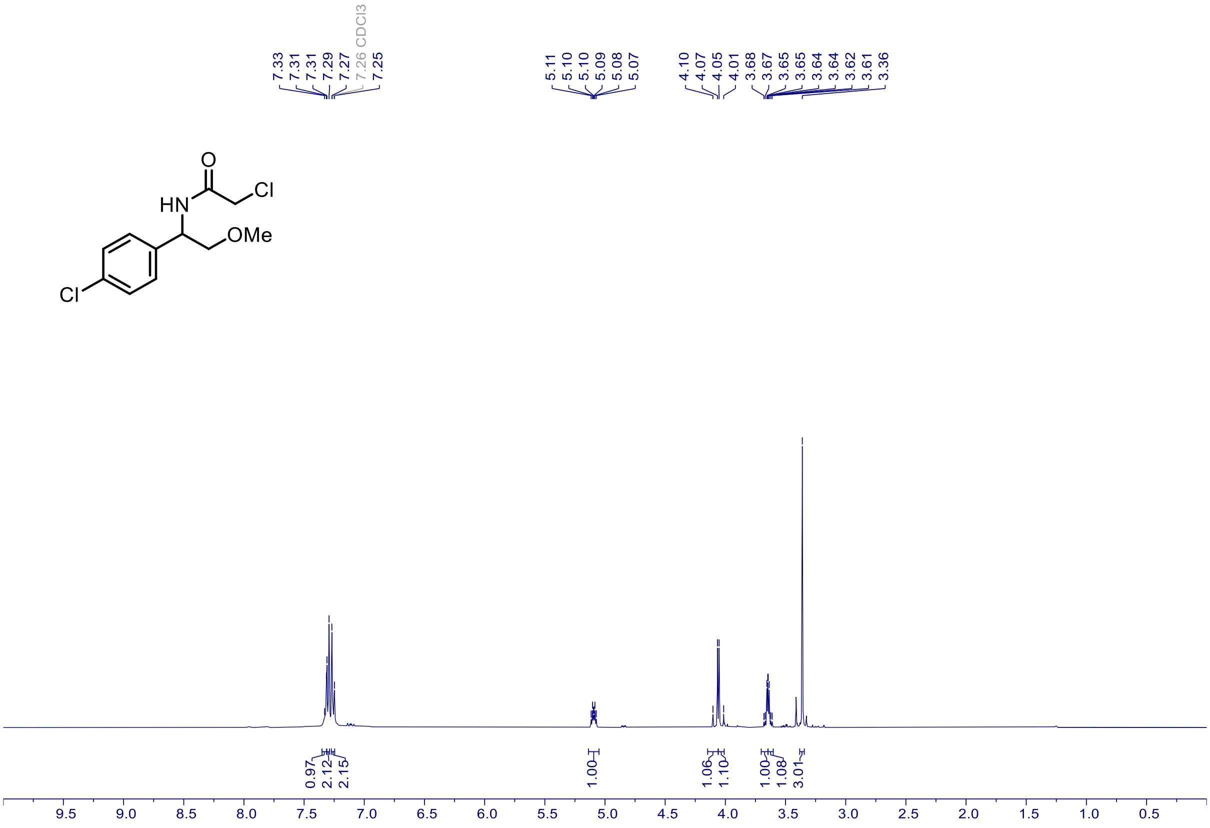

6c – ^{13}C NMR (101 MHz, $CDCl_3$)

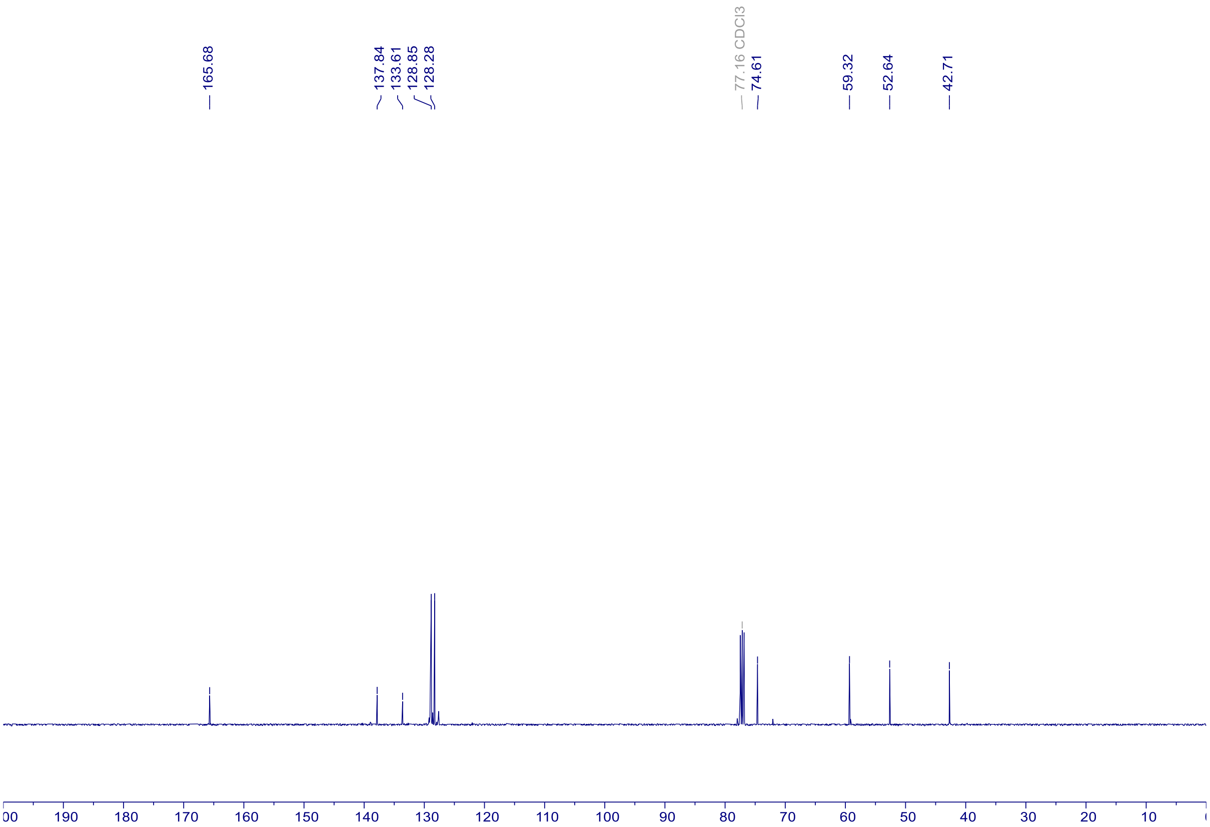

6d – ^{1}H NMR (400 MHz, CDCl$_3$)

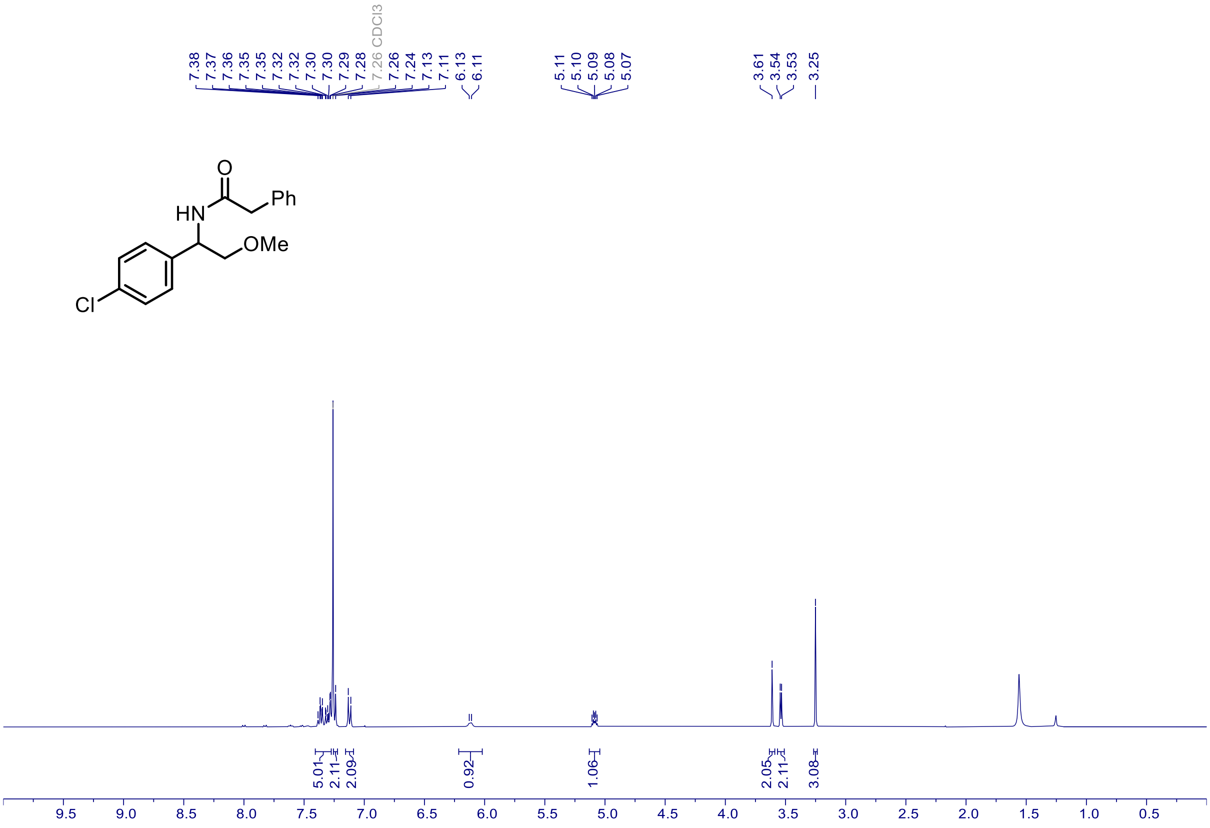

6d – ^{13}C NMR (101 MHz, CDCl$_3$)

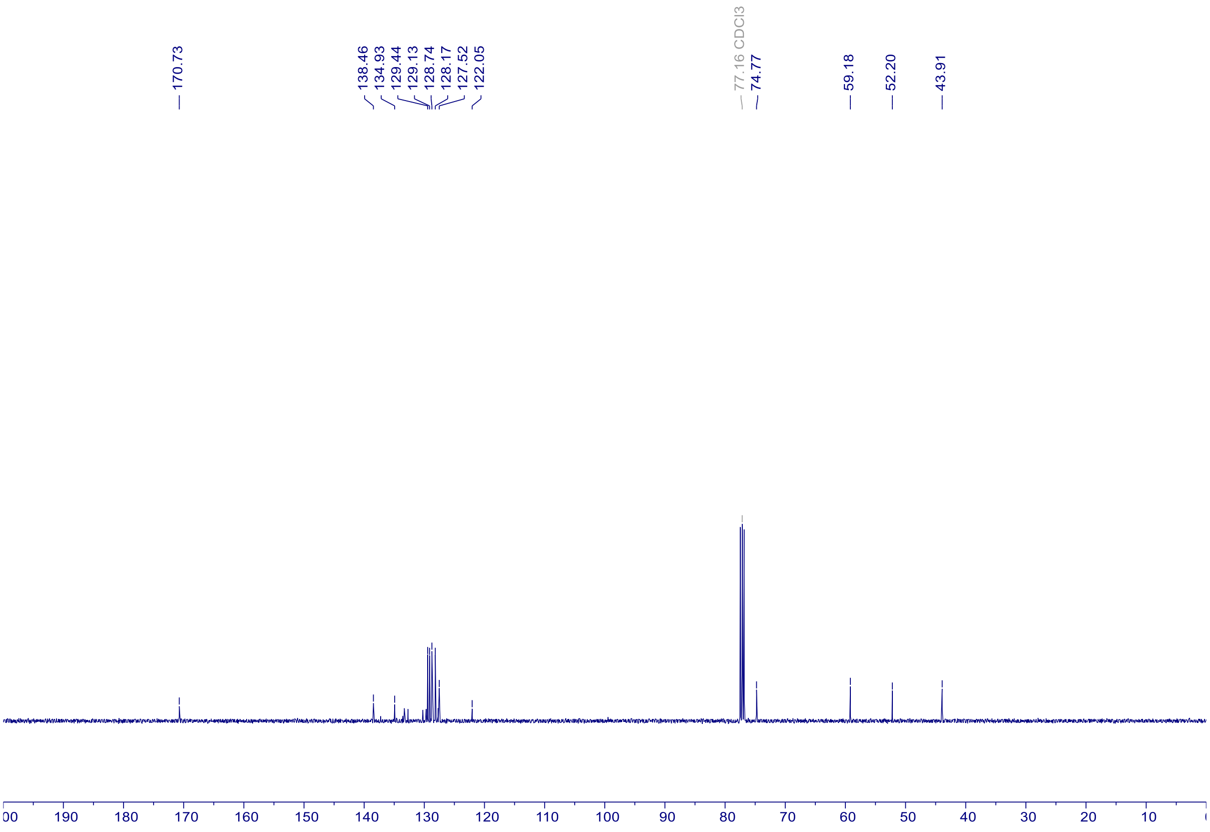

6e – ^{1}H NMR (400 MHz, $CDCl_3$)

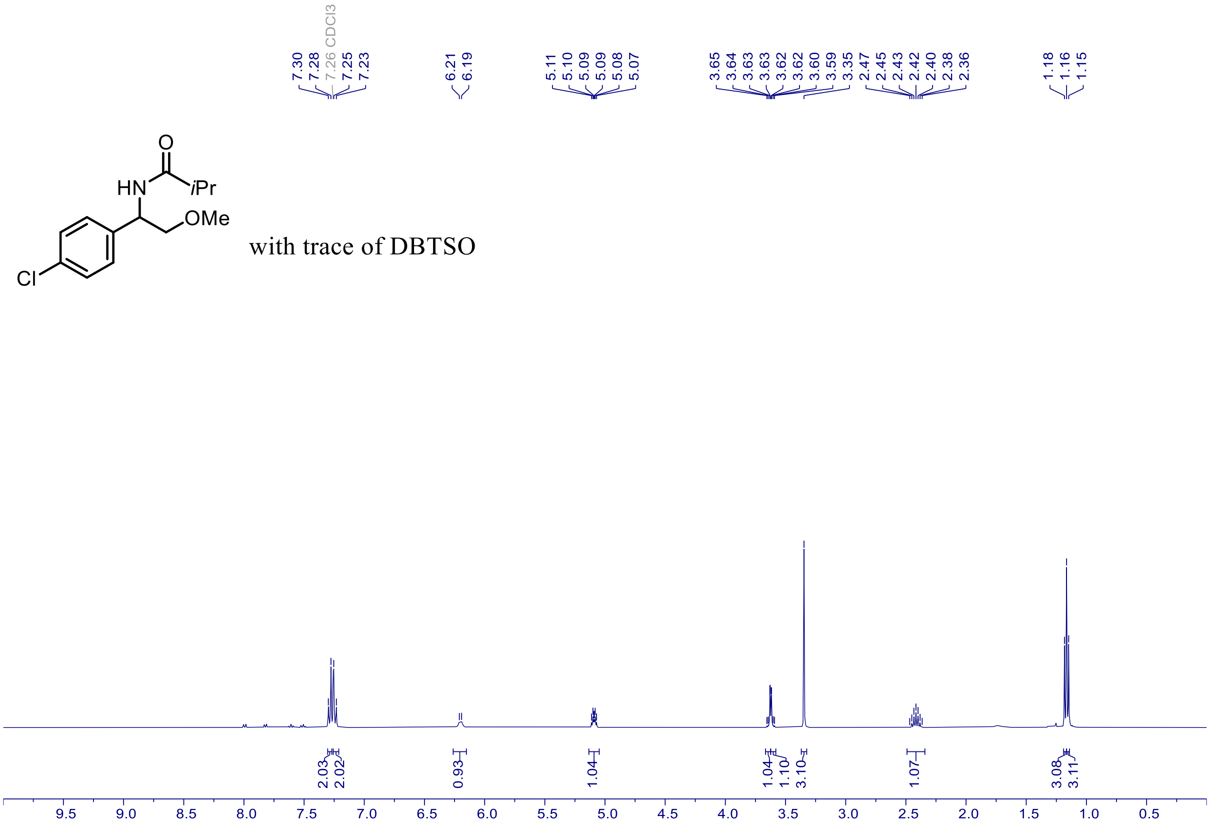

6e – ^{13}C NMR (101 MHz, $CDCl_3$)

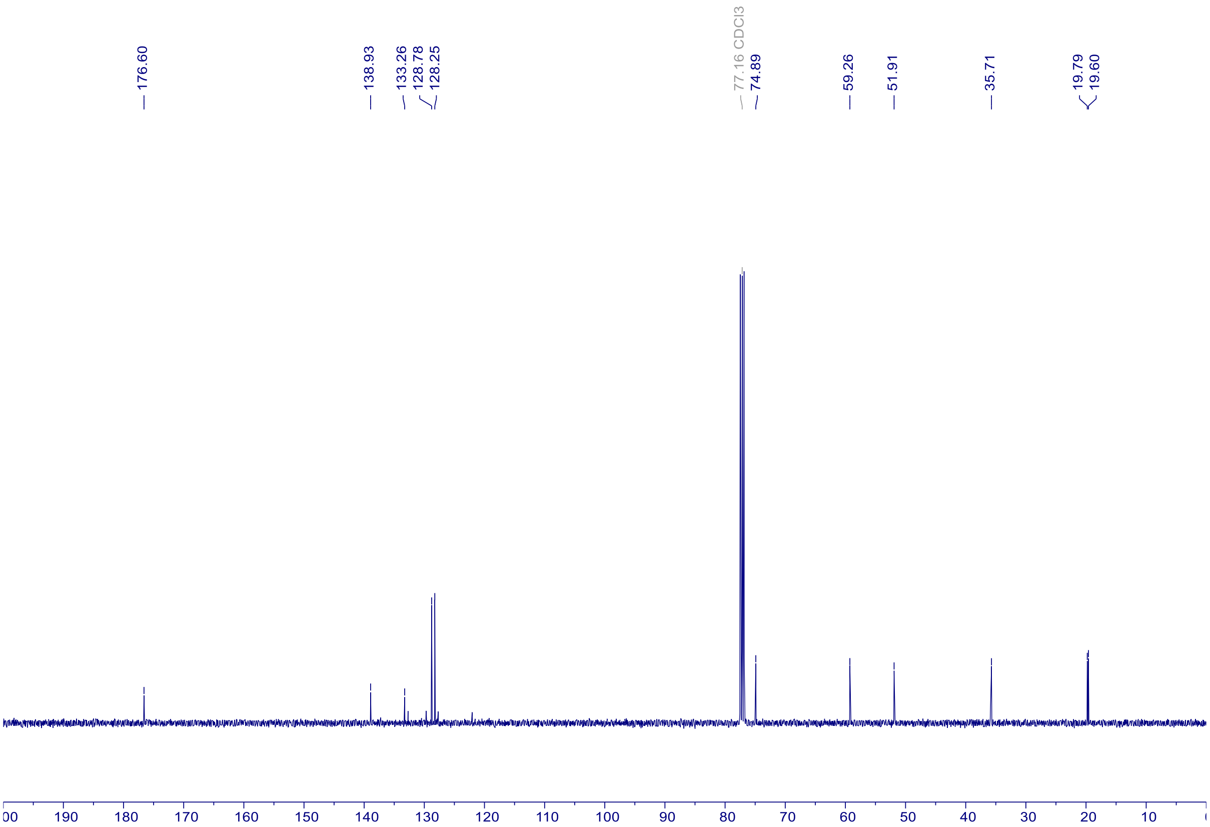

6f – ¹H NMR (400 MHz, CDCl$_3$)

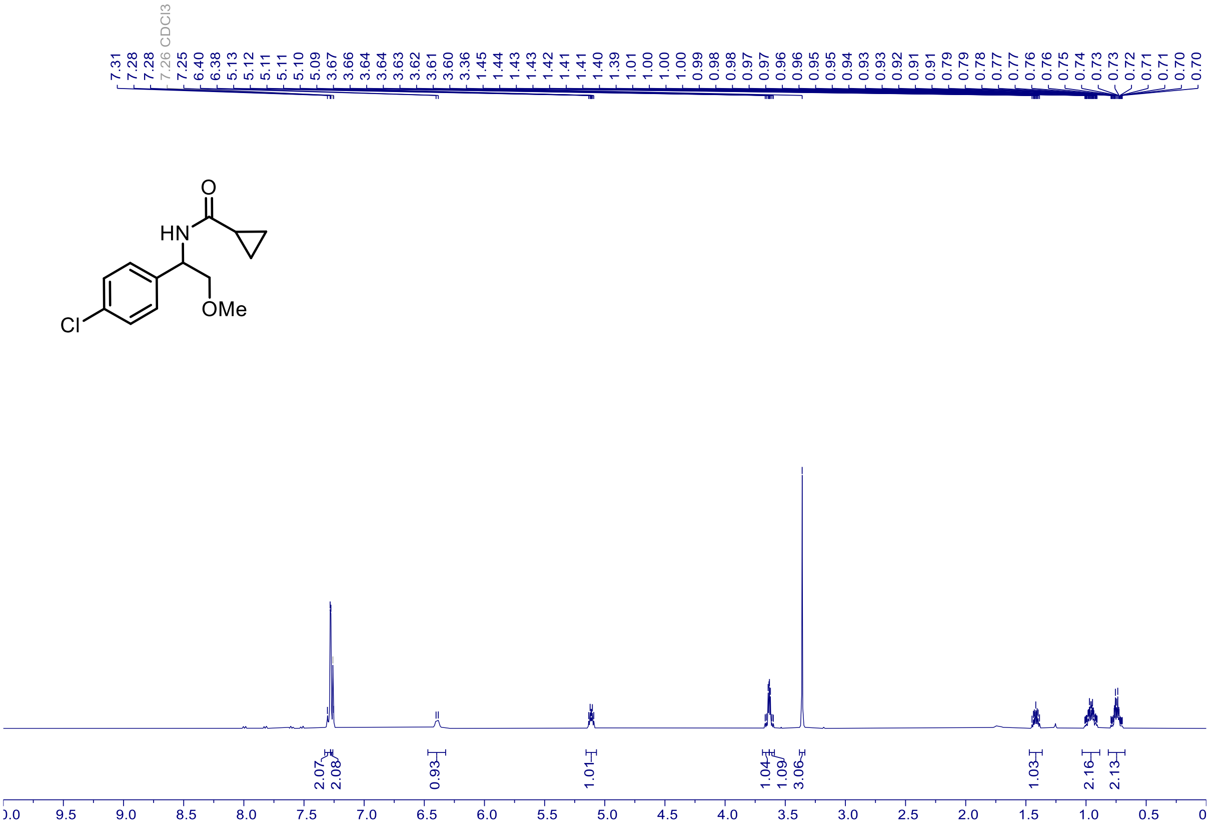

6f – ¹³C NMR (101 MHz, CDCl$_3$)

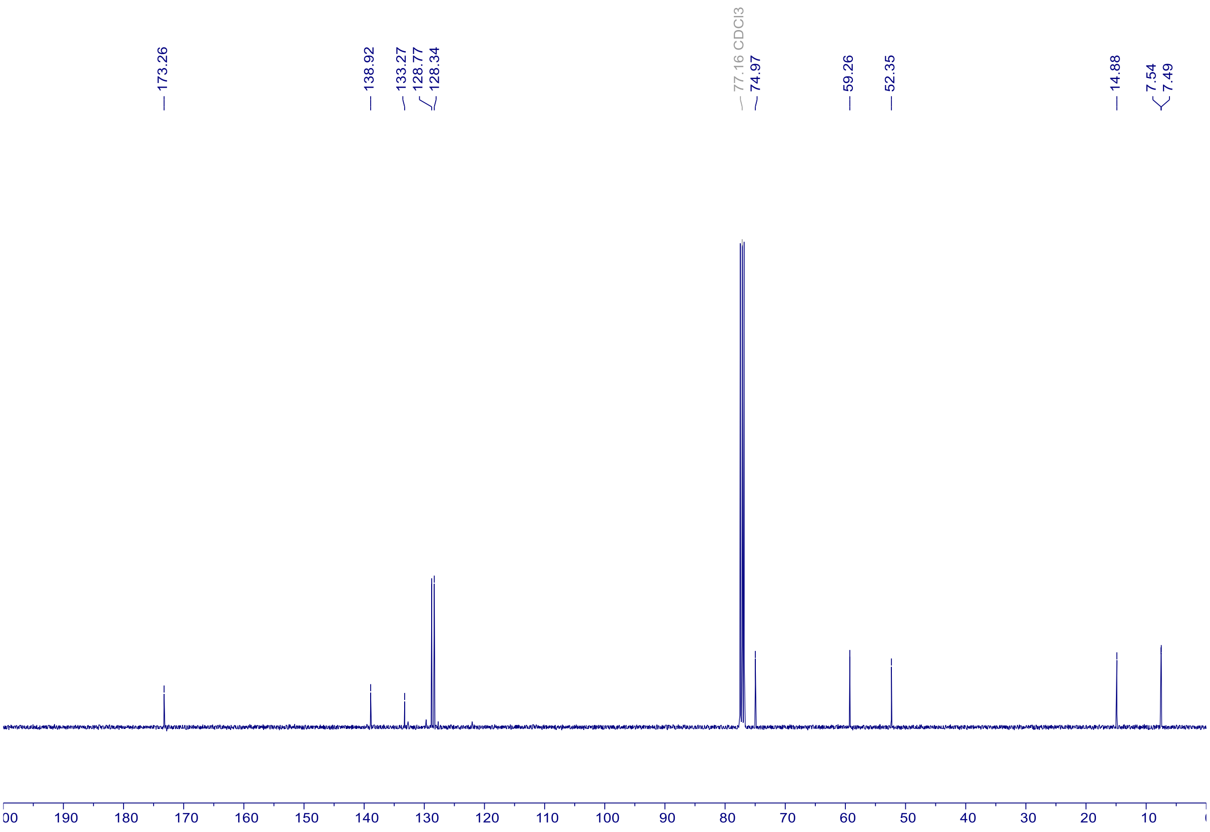

6g – ^{1}H NMR (400 MHz, $CDCl_3$)

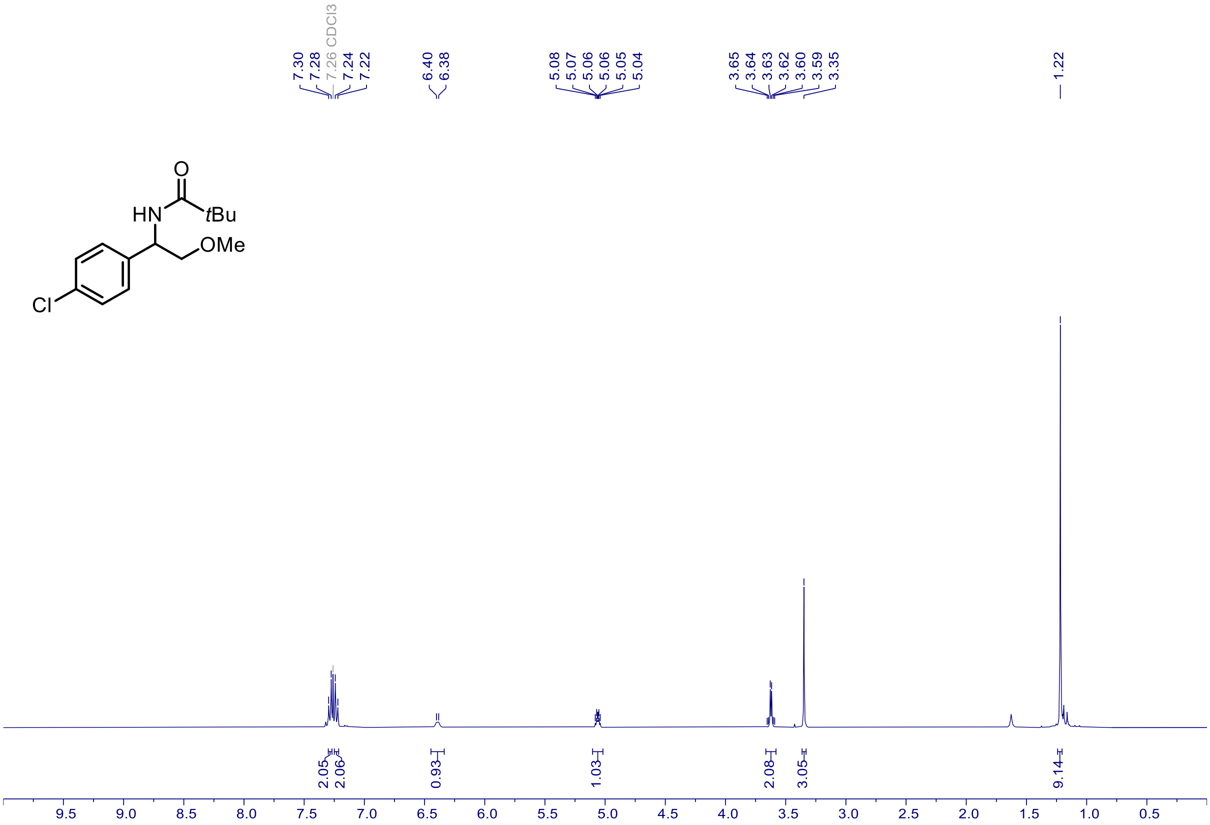

6g – ^{13}C NMR (101 MHz, $CDCl_3$)

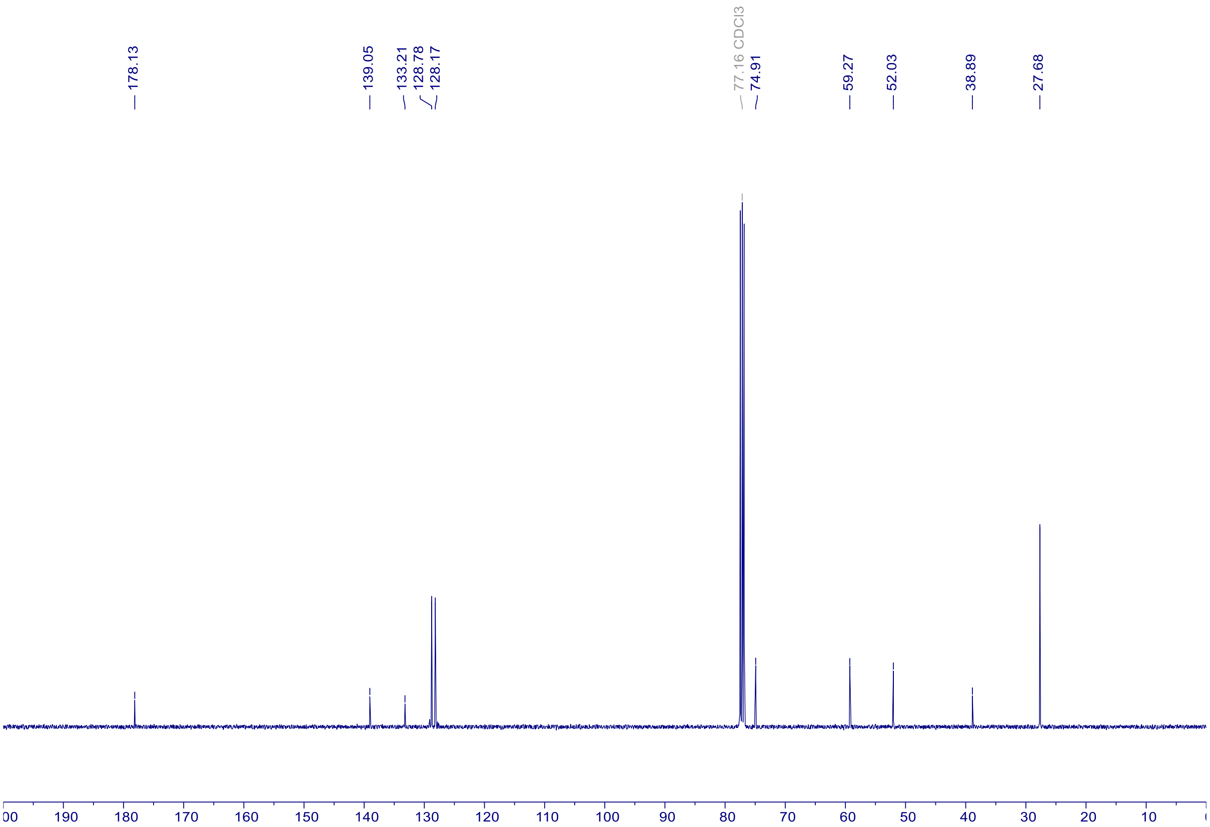

6h – ^{1}H NMR (400 MHz, CDCl$_3$)

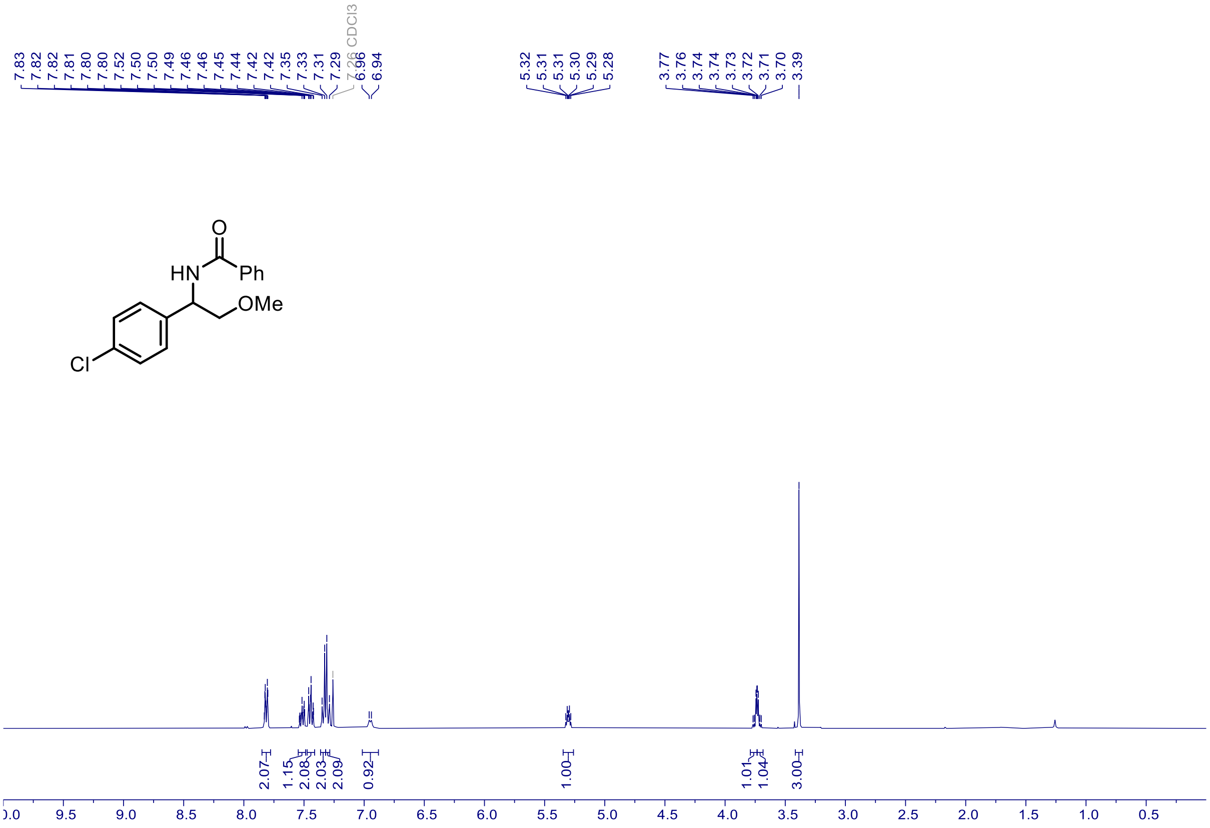

6h – ^{13}C NMR (101 MHz, CDCl$_3$)

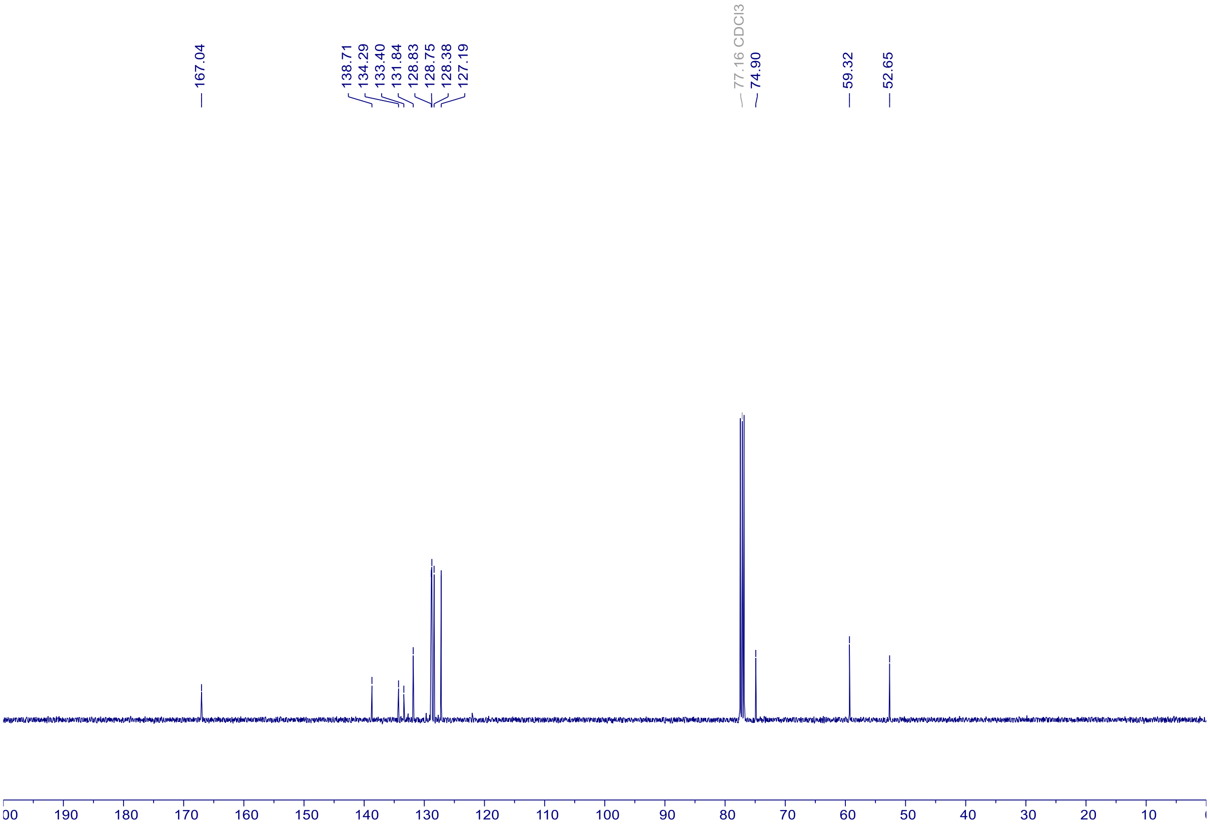

6i – ^{1}H NMR (400 MHz, $CDCl_3$, 5:1 mixture of diastereoisomers)

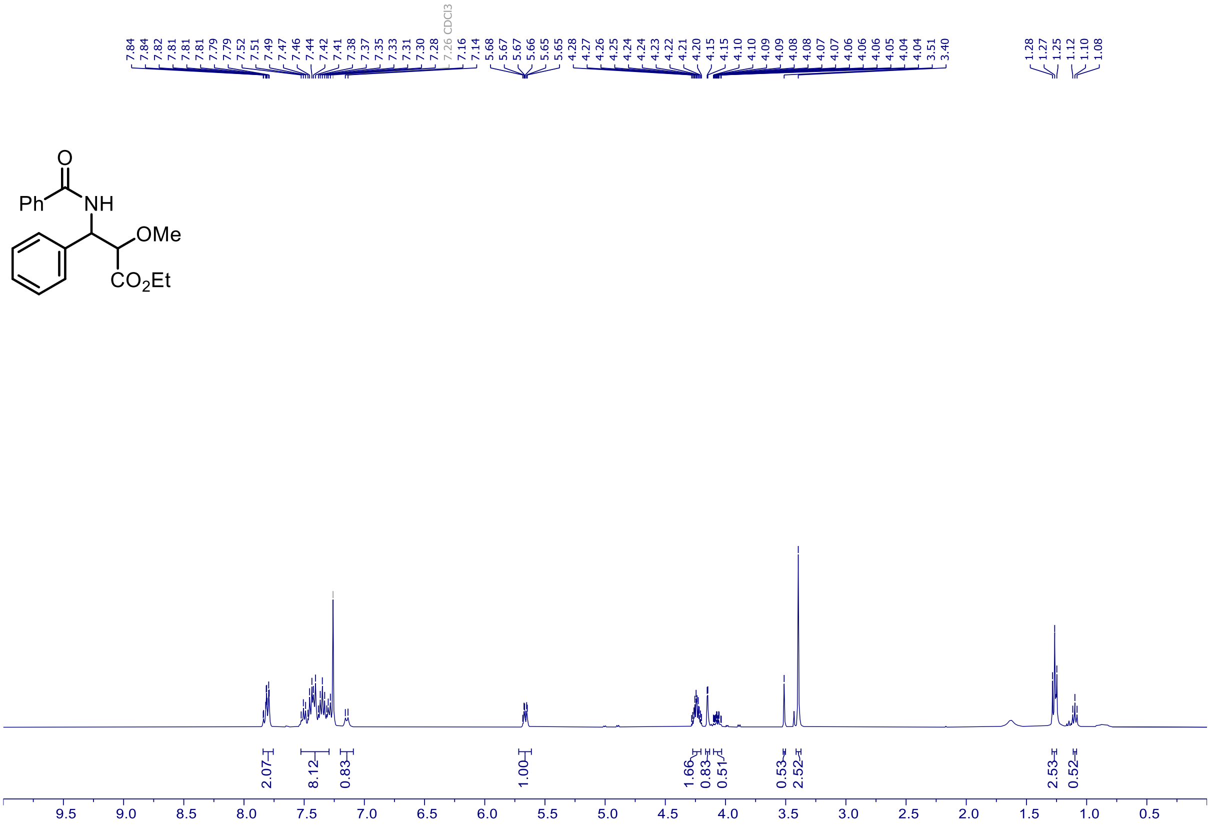

6i – ^{13}C NMR (101 MHz, $CDCl_3$, 5:1 mixture of diastereoisomers)

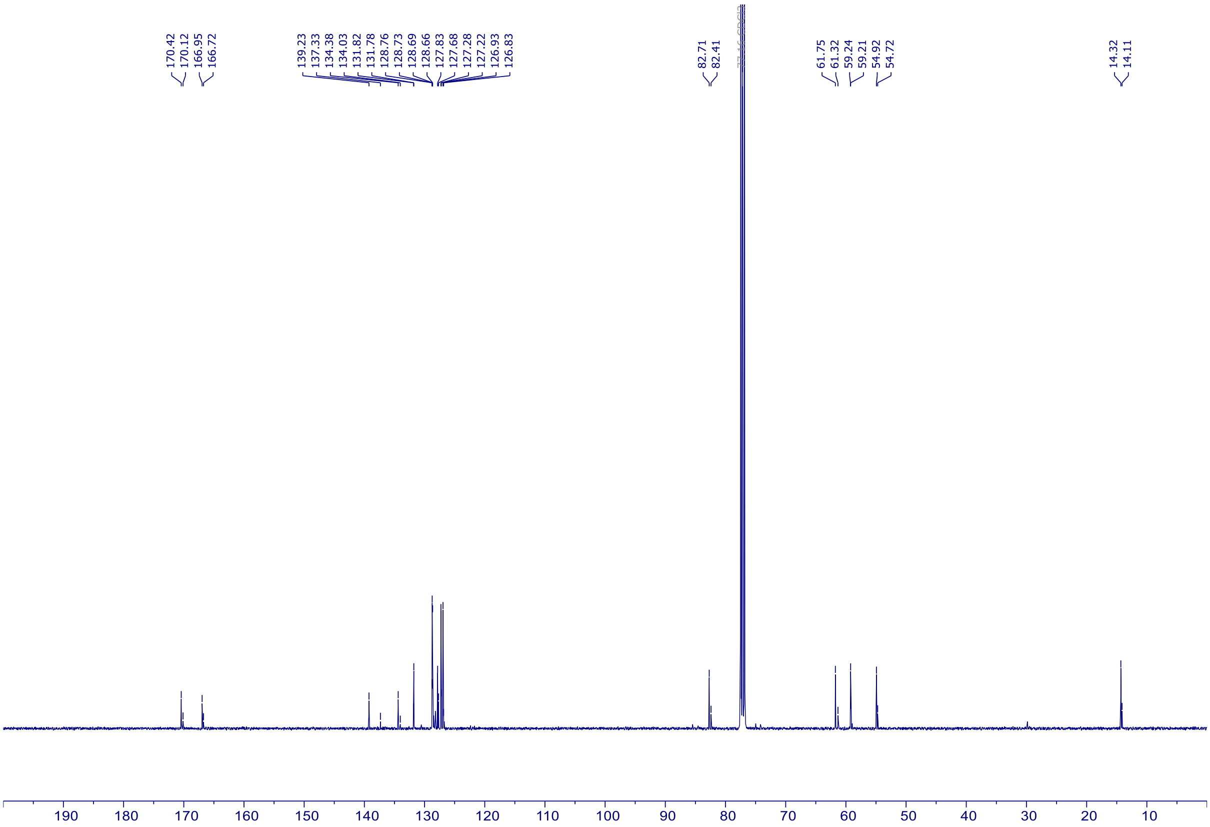

9 – ^{1}H NMR (400 MHz, $CDCl_3$)

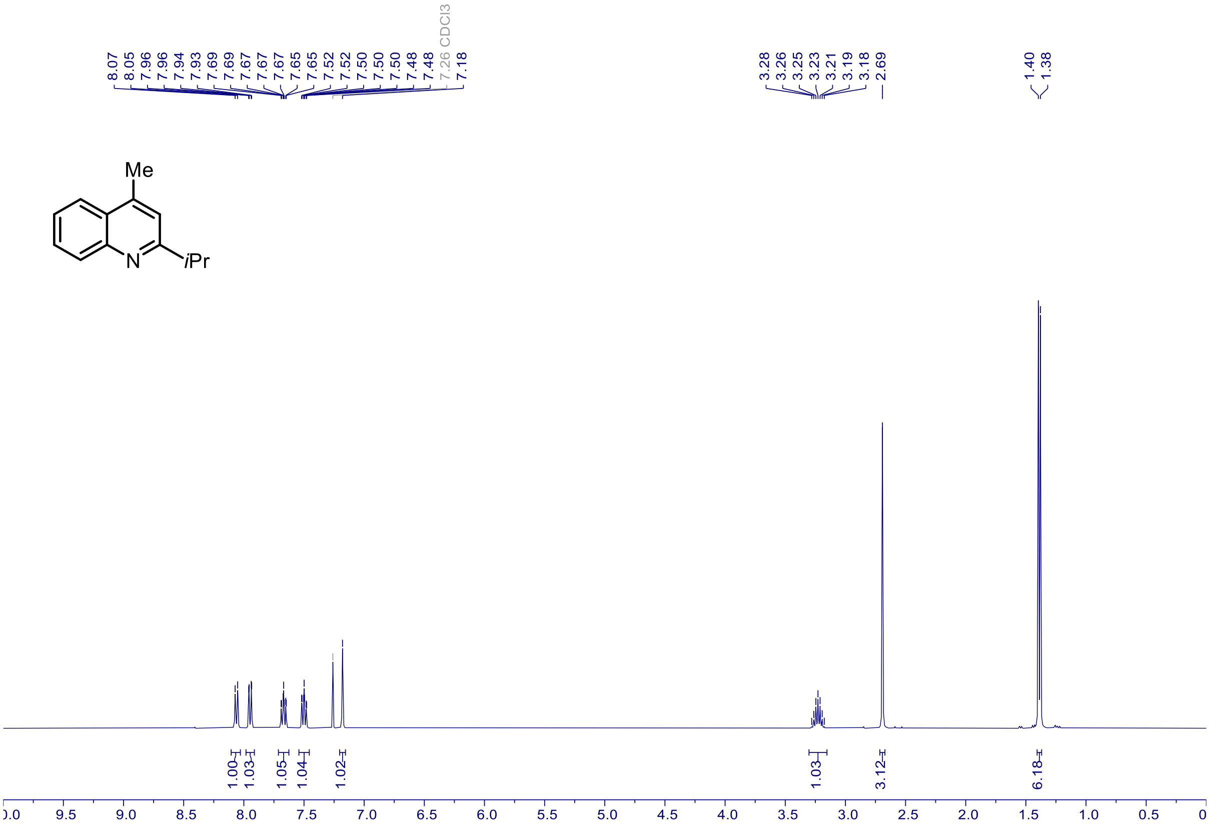

9 – ^{13}C NMR (101 MHz, $CDCl_3$)

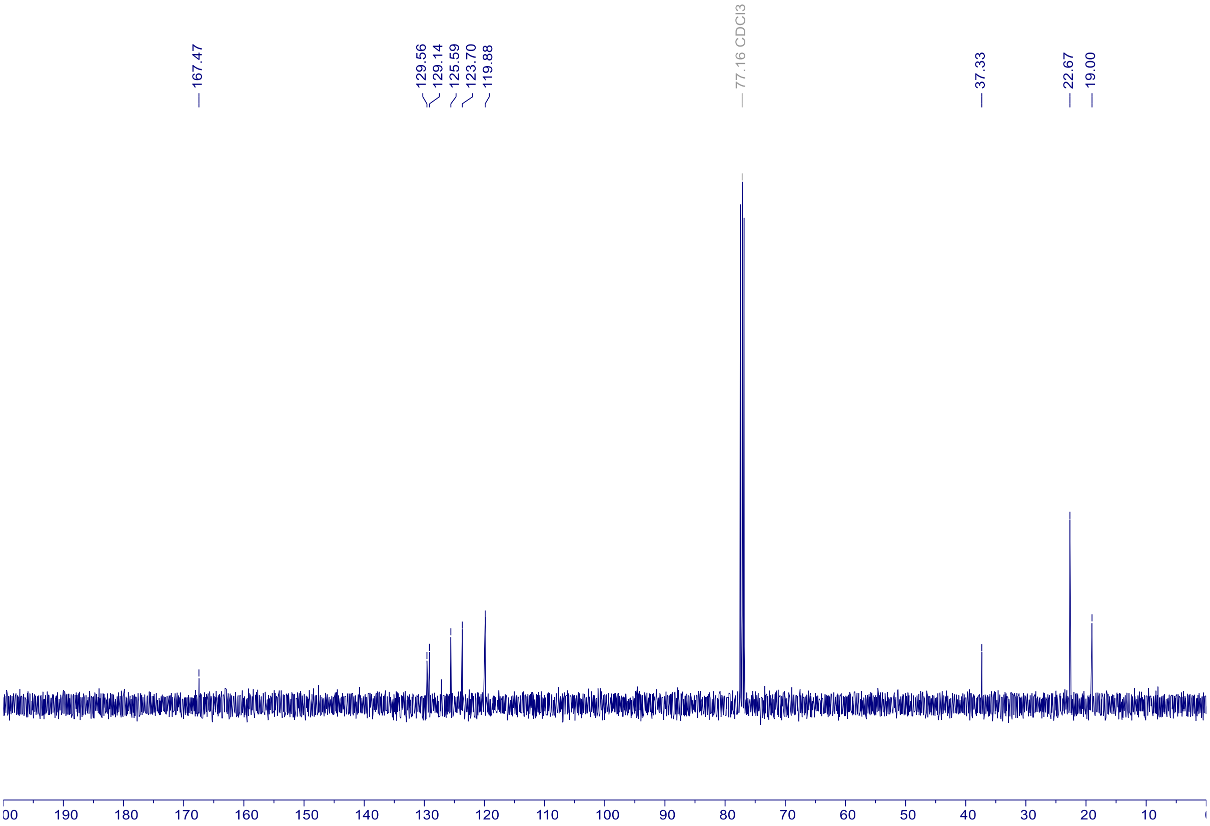

9 References

1 Butcher, T. W. *et al.* Regioselective Copper-Catalyzed Boracarboxylation of Vinyl Arenes. *Org. Lett.* **18**, 6428–6431 (2016).

2 Kwong, C. K.-W., Huang, R., Zhang, M., Shi, M. & Toy, P. H. Bifunctional Polymeric Organocatalysts and Their Application in the Cooperative Catalysis of Morita–Baylis–Hillman Reactions. *Chem. Eur. J.* **13**, 2369–2376 (2007).

3 Granados, A., Dhungana, R. K., Sharique, M., Majhi, J. & Molander, G. A. From Styrenes to Fluorinated Benzyl Bromides: A Photoinduced Difunctionalization via Atom Transfer Radical Addition. *Org. Lett.* **24**, 4750–4755 (2022).

4 Zhang, W. & Lin, S. Electroreductive Carbofunctionalization of Alkenes with Alkyl Bromides via a Radical-Polar Crossover Mechanism. *J. Am. Chem. Soc.* **142**, 20661–20670 (2020).

5 Maity, S. *et al.* Efficient and Stereoselective Nitration of Mono- and Disubstituted Olefins with $AgNO_2$ and TEMPO. *J. Am. Chem. Soc.* **135**, 3355–3358 (2013).

6 Wang, J. *et al.* Visible-Light-Induced Regioselective Radical Oxo-Amination of Alkenes with O_2 as the Oxygen Source. *Org. Lett.* **25**, 5333–5338 (2023).

7 Li, J. *et al.* Visible-Light-Driven Oxidative Cleavage of Alkenes Using Water-Soluble CdSe Quantum Dots. *ChemSusChem* **14**, 4985–4992 (2021).

8 Ebner, C., Müller, C. A., Markert, C. & Pfaltz, A. Determining the Enantioselectivity of Chiral Catalysts by Mass Spectrometric Screening of Their Racemic Forms. *J. Am. Chem. Soc.* **133**, 4710–4713 (2011).

9 Pace, V. *et al.* Highly regioselective control of 1,2-addition of organolithiums to α,β-unsaturated compounds promoted by lithium bromide in 2-methyltetrahydrofuran: a facile and eco-friendly access to allylic alcohols and amines. *Tetrahedron* **67**, 2670–2675 (2011).

10 Peng, X., Tong, B. M. K., Hirao, H. & Chiba, S. Inorganic-Base-Mediated Hydroamination of Alkenyl Oximes for the Synthesis of Cyclic Nitrones. *Angew. Chem. Int. Ed.* **53**, 1959–1962 (2014).

11 Fu, N., Sauer, G. S., Saha, A., Loo, A. & Lin, S. Metal-catalyzed electrochemical diazidation of alkenes. *Science* **357**, 575–579 (2017).

12 Lu, H., Lv, Y., Ye, Y., Peng, J. & Guo, H. Novel benzazepine spiro derivative. WO2022111581 A1 (2022).

13 Stach, T., Dräger, J. & Huy, P. H. Nucleophilic Substitutions of Alcohols in High Levels of Catalytic Efficiency. *Org. Lett.* **20**, 2980–2983 (2018).

14 Liu, H., Pattabiraman, V. R. & Vederas, J. C. Stereoselective Syntheses of 4-Oxa Diaminopimelic Acid and Its Protected Derivatives via Aziridine Ring Opening. *Org. Lett.* **9**, 4211–4214 (2007).

15 Barthelemy, A. L., Tuccio, B., Magnier, E. & Dagousset, G. Alkoxyl Radicals Generated under Photoredox Catalysis: A Strategy for anti-Markovnikov Alkoxylation Reactions. *Angew. Chem. Int. Ed.* **57**, 13790–13794 (2018).

16 Schmidt, T. A., Ciszek, B., Kathe, P. & Fleischer, I. Tandem Acid/Pd-Catalyzed Reductive Rearrangement of Glycol Derivatives. *Chem. Eur. J.* **26**, 3641–3646 (2020).

17 Báez-Santos, Y. M. *et al.* X-ray Structural and Biological Evaluation of a Series of Potent and Highly Selective Inhibitors of Human Coronavirus Papain-like Proteases. *J. Med. Chem.* **57**, 2393–2412 (2014).

18 Li, C., Pei, X.-Q., Yi, D., Li, T.-B. & Wu, Z.-L. Bioreductive dynamic kinetic resolution of ethyl 2-methoxy-3-oxo-3-phenylpropanoate. *Catal. Commun.* **135**, 105865 (2020).

19 Schetter, B., Stosiek, C., Ziemer, B. & Mahrwald, R. Multinuclear enantiopure titanium self-assembly complexes—synthesis, characterization and application to organic synthesis. *Appl. Organometal. Chem.* **21**, 139–145 (2007).

20 Itoh, K., Shino, S., Maekawa, H. & Nishiguchi, I. Novel synthesis of 2-arylcyclohexanones using anodic oxidation of enol esters as the key step. *J. Electroanal. Chem* **507**, 14–21 (2001).

21 Chang, L., An, Q., Duan, L., Feng, K. & Zuo, Z. Alkoxy Radicals See the Light: New Paradigms of Photochemical Synthesis. *Chem. Rec.* **122**, 2429–2486 (2022).

22 Wu, M. *et al.* Enhanced Reactivity of Acridinium Perchlorate: Harnessing Redox Mediators for Trace Chloride Activation in Hydrogen Atom Transfer Photocatalysis. *ACS Catal.* **14**, 9364–9373 (2024).

23 Pavlishchuk, V. V. & Addison, A. W. Conversion constants for redox potentials measured versus different reference electrodes in acetonitrile solutions at 25°C. *Inorganica Chimica Acta* **298**, 97–102 (2000).

24 Hatchard, C. G., Parker, C. A. & Bowen, E. J. A new sensitive chemical actinometer - II. Potassium ferrioxalate as a standard chemical actinometer. *Proc. Math. Phys.* **235**, 518–536 (1956).
